# Association between high dietary intake of live microbes from food and all-cause and cause-specific mortality in cancer patients: A prospective cohort study

**DOI:** 10.1097/MD.0000000000049649

**Published:** 2026-07-10

**Authors:** Jingyang Zeng, Xiaobo Liu, Yingxuan Huang, Yisen Huang, Yubin Wang, Xiaoqiang Liu

**Affiliations:** a Department of Anesthesiology, First Hospital of Quanzhou Affiliated to Fujian Medical University, Quanzhou, Fujian, China; b McConnell Brain Imaging Centre, Montreal Neurological Institute, McGill University, Montreal, Quebec, Canada; c Department of Gastroenterology, First Hospital of Quanzhou Affiliated to Fujian Medical University, Quanzhou, Fujian, China.

**Keywords:** cancer patient survival, live dietary microbes, mortality, NHANES, prospective cohort study

## Abstract

Dietary live microbes have gained attention as potential modulators in chronic disease management, yet their impact on the survival of cancer patients remains underexplored. This study assessed the association between dietary live microbe intake from food and all-cause as well as cause-specific mortality in cancer patients. Data from 3649 adult cancer patients in the 1999 to 2018 National Health and Nutrition Examination Survey were analyzed. Participants were grouped according to their intake of foods with medium-to-high microbial content (MedHi food): G1 (no intake), G2 (below median), and G3 (above median). Kaplan–Meier survival analysis and multivariable Cox regression were used to evaluate mortality outcomes, while restricted cubic spline models assessed dose–response relationships. Over a median follow-up of 7.08 years, 1290 participants died, including 424 cancer-specific deaths. Compared with G1, G3 had 21% lower all-cause mortality and 27% lower noncancer mortality. No significant association was observed with cancer-specific mortality. A nonlinear, inverse relationship was observed for all-cause mortality at intake levels below 489.7 grams, plateauing beyond this threshold. Higher MedHi food intake was associated with lower risks of all-cause and noncancer mortality among adults with self-reported cancer, but not with cancer-specific mortality. Because National Health and Nutrition Examination Survey lacks detailed information on cancer type, stage, treatment, and time since diagnosis, these cancer-specific clinical factors were not adjusted for in the present analyses. These findings should therefore be interpreted as observational and hypothesis-generating, and may inform future prospective studies on dietary live microbes and survival among cancer survivors.

## 1. Introduction

In recent years, the significance of dietary patterns in the prevention and management of chronic diseases has been increasingly recognized, with dietary live microbes emerging as a potential modulating factor of considerable interest.^[1,2]^ Dietary live microbes, primarily including various probiotics and microorganisms that survive fermentation processes, may exert diverse beneficial effects on host health by modulating the gut microbiota, enhancing immune function, and reducing systemic inflammation.^[3]^ Previous studies have demonstrated a significant association between the intake of dietary live microbes and a reduced risk of cardiovascular diseases, metabolic syndrome, and immune-related disorders, but research specifically focusing on cancer patients remains limited.^[4,5]^

Cancer, as one of the leading causes of death worldwide, presents not only the inherent challenges of the disease itself but also the complications and metabolic disturbances induced by treatment, making the improvement of cancer patient outcomes a complex and crucial challenge.^[6–8]^ Although some studies have explored the relationship between dietary factors and cancer prognosis, the role of dietary live microbes in this context has not been thoroughly investigated.^[9,10]^ Given the potential of dietary live microbes in modulating gut health and systemic inflammation, it is particularly important to explore their impact on the survival rates of cancer patients.

This study, utilizing nationally representative data from adult cancer patients participating in the National Health and Nutrition Examination Survey (NHANES) from 1999 to 2018, is the first to systematically investigate the association between dietary live microbe intake and all-cause and cause-specific mortality among cancer patients. Through this research, we aim to fill the gap in understanding the relationship between dietary live microbe intake and mortality in cancer patients, thereby laying the foundation for future in-depth studies in this field.

## 2. Methods

### 2.1. Study population

**Figure 1.**
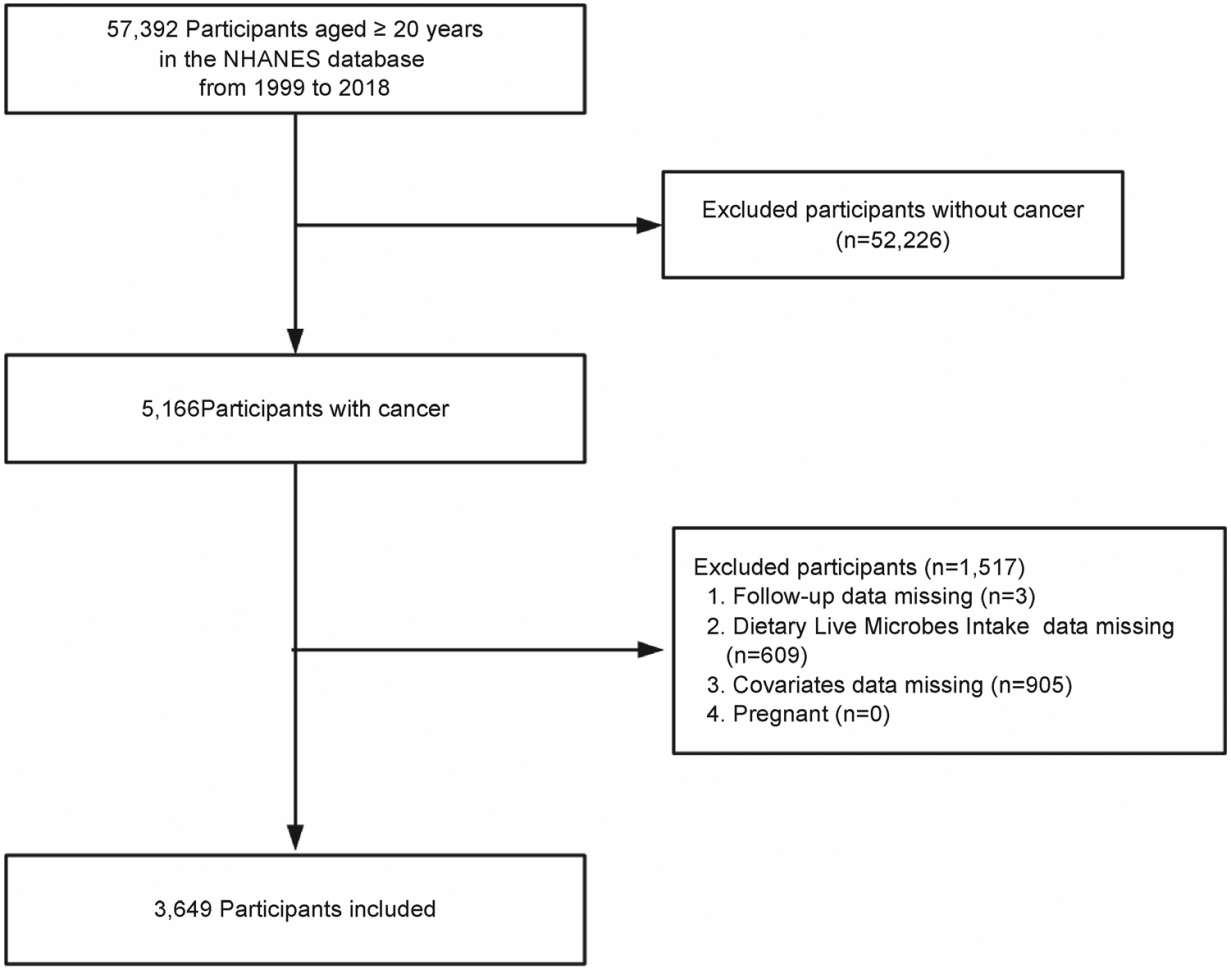
Study flow chart.

The NHANES is a nationally representative survey conducted by the National Center for Health Statistics (NCHS) using a stratified, multistage probability cluster sampling method to assess the health and nutritional status of the non-institutionalized U.S. population. The NHANES research protocols were approved by the NCHS Ethics Review Board, and all participants provided written informed consent. The present study was conducted in full accordance with the ethical principles of the Declaration of Helsinki. This study utilized data from adults who participated in NHANES between 1999 and 2018. Inclusion criteria were adults aged ≥20 years. Cancer status was defined based on participants’ self-reported history of a prior cancer diagnosis in the NHANES questionnaire. Specifically, participants were classified as having cancer if they reported that a doctor or other health professional had ever told them that they had cancer or a malignancy. Participants with nonmalignant tumors, those lost to follow-up, missing data on dietary live microbe intake, or missing covariate data, as well as pregnant women, were excluded. The final sample included 3649 adults with self-reported cancer (Fig. 1).

### 2.2. Assessment of dietary live microbe intake

Energy and nutrient intakes were calculated using 24-hour dietary recall data, linked to the cycle-specific Food and Nutrient Database for Dietary Studies provided by the U.S. Department of Agriculture (USDA). Dietary live microbe intake was estimated using the first-day 24-hour dietary recall, which was available for all NHANES cycles and collected via a standardized in-person interview at the mobile examination center. The use of Day 1 recall ensured methodological consistency across survey cycles (1999–2018), including earlier cycles in which only 1 recall day was available. Four food-microbiology specialists (Maria L. Marco, Mary E. Sanders, Robert Hutkins, and Colin Hill) systematically assigned colony-forming-unit estimates (CFU/g) to all 9388 NHANES food codes, drawing on peer-reviewed studies, authoritative reviews, and evidence of how processing steps such as pasteurization influence microbial survival.^[11]^ Each NHANES food code was assigned to one of 3 viable-microbe categories on the basis of expert judgement and published data on microbial survival during processing: low (<10^4^ CFU/g), medium (10^4^ to 10^7^ CFU/g), or high (>10^7^ CFU/g). Intra- and inter-team discussions determined these classifications, and external expert input from Fred Breidt (USDA Agricultural Research Service) was also incorporated. The complete mapping of NHANES food codes to microbial categories has been previously published and is provided in Supplementary Table 1, Supplemental Digital Content 1 to enhance transparency and reproducibility. Although NHANES food codes are periodically updated across survey cycles, the microbial classification framework was applied consistently according to food processing characteristics and expected microbial survival, without modification of the original categorization principles. Because NHANES dietary recall data are recorded at the food-code level, mixed dishes were classified according to their assigned NHANES food codes without further disaggregation into individual ingredients. This approach is consistent with the original classification methodology and avoids introducing additional assumptions regarding the microbial content of component ingredients. Because routine thermal processing markedly diminishes viable microbes, items such as pasteurized milk, processed meats, pork and poultry products, prepared seafood dishes, as well as sauces and gravies were all classified as “Lo” (<10^4^ CFU/g) category. In contrast, the Med category consisted mainly of fresh vegetables and fruits, which generally carry a moderate microbial load. Fermented dairy products made up most of the Hi category, reflecting their high concentrations of viable microbes.^[12]^ To quantify live microbe exposure, we calculated the total grams per day of foods classified as medium-to-high microbial content (MedHi). Participants were divided into 3 MedHi food intake groups: G1 comprised individuals who consumed no MedHi food, G2 included those whose consumption of MedHi food was greater than zero but below the cohort median, and G3 encompassed individuals whose intake of MedHi food exceeded the median.^[13]^

### 2.3. Determination of mortality

Vital status was ascertained by linking respondents to the publicly available NHANES Linked Mortality File, with follow-up ending on December 31, 2019. Survival time was calculated from the date of the NHANES baseline examination and interview to the date of death or censoring, rather than from the date of cancer diagnosis. Underlying causes of death were classified according to the International Classification of Diseases, 10th Revision (ICD-10). We examined all-cause mortality and cause-specific mortality, including cancer-specific mortality defined by malignant neoplasms as the underlying cause of death (ICD-10: C00–C97). Noncancer mortality was defined as death from all other underlying causes, including cardiovascular, respiratory, metabolic, and external causes.

### 2.4. Covariate assessment

Covariates included age, sex, race, marital status, body mass index (BMI), poverty income ratio (PIR), education level, Healthy Eating Index (HEI-2015), physical activity, smoking status, alcohol intake, and the presence of cardiovascular disease (CVD), hypertension, hyperlipidemia, and diabetes. Age was analyzed as a continuous covariate, using each participant’s exact age (in years). Sex recorded participants’ sex. Race was categorized as non-Hispanic White, others (non-Hispanic Black, Mexican American, other Hispanic, and other races). Marital status was categorized as married/living with a partner and unmarried/other (including widowed, divorced, or separated). BMI was a continuous variable calculated based on participants’ height and weight. The PIR was stratified into 3 groups: 1.00 to 1.30, 1.31 to 3.50, and >3.50.^[14]^ Educational attainment was categorized into 3 tiers: less than high school, completion of high school or an equivalent credential, and education beyond high school. The HEI-2015 aggregates 13 component scores calculated from each participant’s 24-hour dietary recall; higher totals signal superior diet quality and closer adherence to the Dietary Guidelines for Americans, 2015 to 2020.^[15]^ Smoking status was categorized as never smokers (smoked <100 cigarettes in their lifetime), former smokers (smoked more than 100 cigarettes but are currently nonsmokers), and current smokers (smoked more than 100 cigarettes and currently smoke occasionally or daily).^[16]^ Alcohol intake status was classified into 3 groups: never drinkers, who reported fewer than 12 alcoholic beverages over their lifetime; former drinkers, who had consumed at least 12 drinks in any single year but none during the past 12 months; and current drinkers, who had consumed at least 12 drinks in a year and continued to drink within the last year.^[17]^ Weekly physical activity, quantified as total MET-minutes accumulated through walking, cycling, occupational tasks, and leisure exercise, was first retained as a continuous measure and then grouped into 3 strata: inactive (0 MET -min/week), insufficiently active (1–599 MET-min/week), and sufficiently active (≥600 MET-min/week).^[18]^ CVD was defined by self-reported previous diagnoses of coronary heart disease, angina, stroke, heart attack, or congestive heart failure.^[18]^ Hypertension was considered present if a participant had an average systolic blood pressure of ≥ 140 mm Hg, an average diastolic blood pressure of ≥ 90 mm Hg, a self-reported physician diagnosis, or current use of antihypertensive medication.^[16]^ Hyperlipidemia was recorded when any one of the following applied: current use of lipid-lowering medication; fasting triglycerides ≥150 mg/dL; or dyslipidemia, defined as total cholesterol ≥200 mg/dL, LDL cholesterol ≥130 mg/dL, or HDL cholesterol <40 mg/dL.^[19]^ Participants were considered diabetic if they reported a physician diagnosis, had an HbA1c ≥6.5 %, showed a fasting plasma glucose ≥7.0 mmol/L, recorded a random or 2-h OGTT glucose ≥11.1 mmol/L, or were using insulin or other glucose-lowering medications.^[20]^

### 2.5. Statistical analysis

Because the study drew solely on an existing dataset, no prospective power analysis was conducted. Statistical computations were carried out in R (The R Foundation for Statistical Computing, http://www.R-project.org) and Free Statistics version 1.7, with statistical significance defined as a 2-tailed *P*-value <.05. Categorical data were summarized as percentages, whereas continuous data were reported as means with their standard deviations. Group differences were evaluated using 1 way analysis of variance for normally distributed continuous variables and the chi-square test for categorical variables. standardized mean differences were calculated to quantify baseline imbalances between MedHi intake groups, with an absolute SMD ≥0.10 considered indicative of meaningful imbalance.

Kaplan–Meier survival curves were generated to examine the relationship between MedHi food intake categories and all-cause, cancer-specific, and noncancer mortality among participants with cancer. Multivariable Cox proportional hazards regression models were employed to assess the associations between MedHi food intake groups and all-cause mortality, cancer-specific mortality, and noncancer mortality. Model 1 adjusted for age and sex; Model 2 further adjusted for race, marital status, BMI, PIR, education level, HEI-2015, physical activity, smoking status, and alcohol intake; Model 3 further adjusted for CVD, hypertension, hyperlipidemia, and diabetes. After covariate adjustment (Model 3), we fitted a restricted cubic spline (RCS) with knots at the 5th, 35th, 65th, and 95th percentiles of MedHi food intake to examine whether the intake-mortality association was linear and to characterize its dose–response pattern. When nonlinearity was suggested by the RCS analysis, a 2-piecewise Cox proportional hazards model was fitted, adjusted for the covariates in Model 3. The potential inflection point was determined in a data-driven manner by comparing models across a range of candidate cut-points within the observed exposure distribution and selecting the point that maximized the log-likelihood. A likelihood-ratio test was then used to compare the 1-line model with the 2-piecewise model. Formal competing-risk analyses, including Fine–Gray subdistribution hazard models, were not performed; therefore, cause-specific mortality results were interpreted using Cox proportionalhazards models based on the underlying cause of death.

Subgroup analyses were conducted by age (< 65 vs ≥65 years), sex (male vs female), race (White vs other), marital status (married vs unmarried), and BMI (< 30 vs ≥30 kg m^−2^). Heterogeneity and interaction across these strata were evaluated with Cox proportional hazards models and likelihood-ratio tests. To verify the robustness of our findings, we carried out several supplementary analyses. First, we re-ran all models after removing individuals who died during the first 2 years of follow-up to reduce potential reverse causation. Second, we repeated the analyses after imputing missing data using a multiple-imputation procedure. Third, we additionally adjusted the fully multivariable model for total energy intake (kcal/day) to account for potential confounding by overall caloric consumption. Fourth, we conducted survey-weighted Cox proportional hazards models incorporating NHANES sampling weights, strata, and primary sampling units to account for the complex survey design. In each scenario, we reestimated the association between MedHi food intake categories and mortality among cancer participants, and the results were materially consistent with the primary analyses, indicating that the findings are robust.

## 3. Results

### 3.1. Participant characteristics

As shown in Table 1, there were 1105 participants in G1 (no MedHi food intake), 1244 participants in G2 (intake below 132.7 grams), and 1300 participants in G3 (intake equal to or above 132.7 grams). The mean ages of participants in these groups were 65.3 ± 14.5 years, 65.8 ± 14.1 years, and 66.3 ± 13.2 years, respectively. Participants in G3 exhibited higher scores in the Healthy Eating Index, higher levels of physical activity, and higher educational attainment, with lower proportions of smokers and cardiovascular disease compared to the other groups. standardized mean differences indicated notable baseline imbalances (absolute SMD ≥0.10) in several socioeconomic and lifestyle factors, particularly HEI-2015, education, and PIR, whereas age and BMI were relatively balanced (Supplementary Table 2, Supplemental Digital Content 2).

**Table 1:** Characteristics of US cancer patients by 3 levels of MedHi food intake.

Variables	Total	G1 (MedHi = 0)	G2 (0 < MedHi < 132.7)	G3 (MedHi ≥ 132.7)	*P*-value
Number of participants	3649	1105	1244	1300	–
Age, Mean ± SD	65.8 ± 13.9	65.3 ± 14.5	65.8 ± 14.1	66.3 ± 13.2	.213
Sex, n (%)					
Male	1758 (48.2)	583 (52.8)	561 (45.1)	614 (47.2)	<.001
Female	1891 (51.8)	522 (47.2)	683 (54.9)	686 (52.8)	
Race, n (%)					
Non-Hispanic White	2627 (72.0)	719 (65.1)	918 (73.8)	990 (76.2)	<.001
Others	1022 (28.0)	386 (34.9)	326 (26.2)	310 (23.8)	
Marital status, n (%)					
Married/ Living with partner	2265 (62.1)	624 (56.5)	793 (63.7)	848 (65.2)	<.001
Never married/Other	1384 (37.9)	481 (43.5)	451 (36.3)	452 (34.8)	
BMI, kg/m^2^, Mean ± SD	28.9 ± 6.5	29.0 ± 6.7	28.8 ± 6.4	28.9 ± 6.3	.727
PIR group, n (%)					
1–1.3	837 (22.9)	365 (33.0)	263 (21.1)	209 (16.1)	<.001
1.31–3.50	1496 (41.0)	460 (41.6)	530 (42.6)	506 (38.9)	
>3.50	1316 (36.1)	280 (25.3)	451 (36.3)	585 (45.0)	
Education level, n (%)					
Less than high school	776 (21.3)	317 (28.7)	269 (21.6)	190 (14.6)	<.001
High school or equivalent	837 (22.9)	286 (25.9)	280 (22.5)	271 (20.8)	
Above high school	2036 (55.8)	502 (45.4)	695 (55.9)	839 (64.5)	
HEI-2015, Mean ± SD	55.4 ± 13.5	50.1 ± 12.4	54.4 ± 13.1	60.9 ± 12.9	<.001
Physical activity, n (%)					
Inactive	1192 (32.7)	452 (40.9)	405 (32.6)	335 (25.8)	<.001
Insufficiently active	828 (22.7)	230 (20.8)	293 (23.6)	305 (23.5)	
Sufficiently active	1629 (44.6)	423 (38.3)	546 (43.9)	660 (50.8)	
Smoking status, n (%)					
Never	1585 (43.4)	414 (37.5)	544 (43.7)	627 (48.2)	<.001
Former	1529 (41.9)	442 (40)	513 (41.2)	574 (44.2)	
Current	535 (14.7)	249 (22.5)	187 (15.0)	99 (7.6)	
Alcohol intake, n (%)					
Never	448 (12.3)	141 (12.8)	161 (12.9)	146 (11.2)	<.001
Former	1011 (27.7)	377 (34.1)	319 (25.6)	315 (24.2)	
Current	2190 (60.0)	587 (53.1)	764 (61.4)	839 (64.5)	
CVD, n (%)					
No	2754 (75.5)	783 (70.9)	944 (75.9)	1027 (79.0)	<.001
Yes	895 (24.5)	322 (29.1)	300 (24.1)	273 (21.0)	
Hypertension, n (%)					
No	1308 (35.8)	359 (32.5)	449 (36.1)	500 (38.5)	.009
Yes	2341 (64.2)	746 (67.5)	795 (63.9)	800 (61.5)	
Hyperlipidemia, n (%)					
No	775 (21.2)	229 (20.7)	254 (20.4)	292 (22.5)	.399
Yes	2874 (78.8)	876 (79.3)	990 (79.6)	1008 (77.5)	
Diabetes, n (%)					
No	2719 (74.5)	798 (72.2)	938 (75.4)	983 (75.6)	.110
Yes	930 (25.5)	307 (27.8)	306 (24.6)	317 (24.4)	

BMI = body mass index, CVD = cardiovascular disease, HEI-2015 = healthy eating index-2015, MedHi = medium-to-high microbial content food, PIR = poverty income ratio.

### 3.2. Association between MedHi food intake and mortality

**Figure 2.**
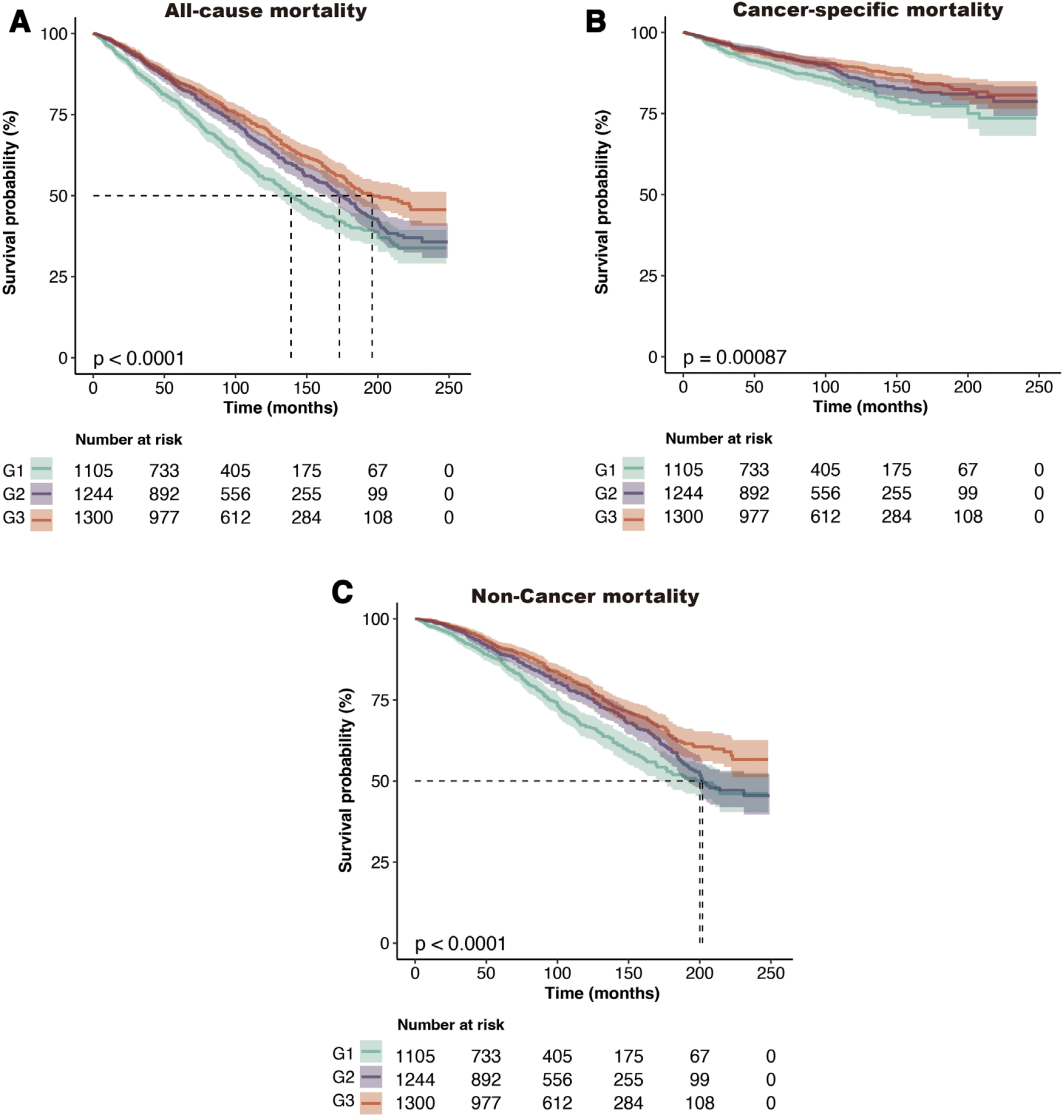
Kaplan–Meier survival curve of all-cause mortality (A), cancer-specific mortality (B), and noncancer mortality (C) according to 3 levels of MedHi food intake among cancer patients.

During a median follow-up period of 7.08 years (interquartile range: 3.25–10.67 years), a total of 1290 participants died, of whom 424 deaths were due to cancer. Participants who consumed more MedHi food exhibited significantly lower cumulative incidences of all-cause mortality, cancer-specific mortality, and noncancer mortality (log-rank test for all comparisons: *P* < .001, Fig. 2).

As shown in Table 2, multivariable Cox regression analysis indicated that, compared to G1, G3 had a 21% reduced risk of all-cause mortality (hazard ratio [HR] = 0.79, 95% confidence interval [CI]: 0.69–0.92, *P* = .002) and a 27% reduced risk of noncancer mortality (HR = 0.73, 95% CI: 0.61–0.87, *P* < .001). However, no significant difference was observed in cancer-specific mortality after adjustment (HR = 0.93, 95% CI: 0.72–1.2, *P* = .587).

**Table 2:** Adjusted hazard ratios of 3 levels of MedHi food intake with risk of all-cause mortality and cause-specific mortality.

Characteristics	Levels of MedHi food intake			*P*-trend
	G1(MedHi = 0)	G2(0 < MedHi < 132.7)	G3(MedHi ≥ 132.7)	
All-cause mortality				
No. deaths/total (%)	448/1105 (40.5%)	441/1224 (35.5%)	401/1300 (30.8%)	–
Person-years	8004.9	10069.3	10054.6	–
Model 1	1 (reference)	0.75 (0.65–0.85)	0.59 (0.52–0.68)	<.001
Model 2	1 (reference)	0.89 (0.78–1.02)	0.78 (0.68–0.91)	.001
Model 3	1 (reference)	0.90 (0.79–1.04)	0.79 (0.69–0.92)	.002
Cancer-specific mortality				
No. deaths/total (%)	151/1105 (13.7%)	140/1244 (11.3%)	133/1300 (10.2%)	–
Person-years	8004.9	10069.3	10054.6	–
Model 1	1 (reference)	0.76 (0.60–0.95)	0.63 (0.50–0.80)	<.001
Model 2	1 (reference)	0.95 (0.75–1.21)	0.95 (0.74–1.23)	.695
Model 3	1 (reference)	0.95 (0.75–1.20)	0.93 (0.72–1.20)	.587
Noncancer mortality				
No. deaths/total (%)	297/1105 (26.9%)	301/1244 (24.2%)	268/1300 (20.6%)	–
Person-years	8004.9	10069.3	10054.6	–
Model 1	1 (reference)	0.74 (0.63–0.87)	0.57 (0.48–0.68)	<.001
Model 2	1 (reference)	0.85 (0.72–1.01)	0.71 (0.59–0.85)	<.001
Model 3	1 (reference)	0.88 (0.74–1.03)	0.73 (0.61–0.87)	<.001

Model 1 was adjusted for age and sex. Model 2 was additionally adjusted for race, marital status, BMI, PIR group, educational level, HEI-2015, physical activity, smoking status, and alcohol intake. Model 3 was additionally adjusted for CVD, hypertension, hyperlipidemia, and diabetes.

BMI = body mass index, CVD = cardiovascular disease, HEI-2015 = healthy eating index-2015, MedHi = medium-to-high microbial content food, PIR = poverty income ratio.

**Figure 3.**
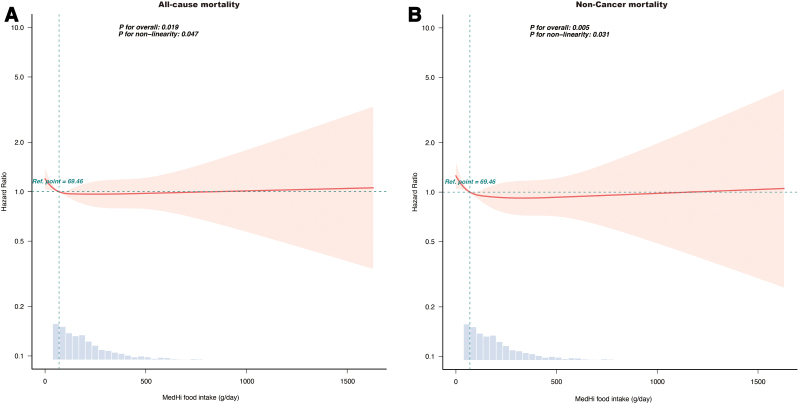
The dose–response association of the MedHi food intake with all-cause mortality (A), and noncancer mortality (B) among cancer patients. This spline model was adjusted for age, sex, race, marital status, BMI, PIR group, educational level, HEI-2015, physical activity, smoking status, and alcohol intake, CVD, hypertension, hyperlipidemia, and diabetes. The reference point shown in the figure indicates the value used for estimating hazard ratios in the RCS model and does not represent the threshold identified in the 2-piecewise Cox regression model. BMI = body mass index, CVD, cardiovascular disease, HEI-2015 = healthy eating index-2015, MedHi = medium-to-high microbial content food, PIR = poverty income ratio, RCS = restricted cubic spline.

Dose–response analysis revealed a nonlinear inverse association between MedHi food intake and both all-cause mortality and noncancer mortality (nonlinear, *P* = .047 and *P* = .031, respectively, Fig. 3). In the 2-piecewise Cox regression model for all-cause mortality, among participants with MedHi food intake < 489.7 g/day, each 100-g/day increment in MedHi food intake was associated with a lower hazard of all-cause mortality (adjusted HR = 0.95, 95% CI: 0.90–0.99; *P* = .045). No significant association was observed among participants with MedHi food intake ≥ 489.7 g/day when the same 100-g/day increment was applied (Table 3).

**Table 3:** Threshold effect analysis of the relationship between MedHi food intake and the incidence of all-cause mortality.

MedHi	HR (95%CI)	*P*-value
**<**489.7 g/d	0.95 (0.90–0.99)	.043
**≥**489.7 g/d	0.69 (0.41–1.18)	.178
Likelihood-ratio test	–	.045

Adjusted for age, sex, race, marital status, BMI, PIR group, educational level, HEI-2015, physical activity, smoking status, and alcohol intake, CVD, hypertension, hyperlipidemia, and diabetes.

BMI = body mass index, CVD = cardiovascular disease, HEI-2015 = healthy eating index-2015, MedHi = medium-to-high microbial content food, PIR = poverty income ratio.

### 3.3. Subgroup analysis

**Figure 4.**
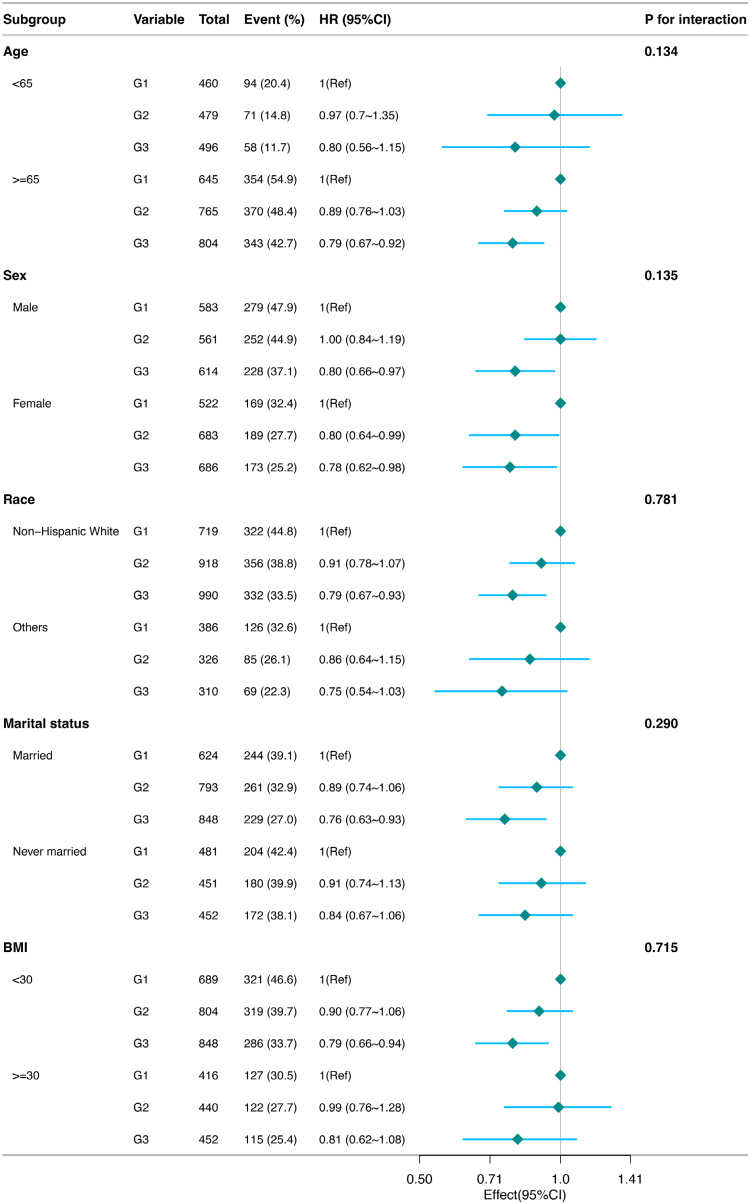
Subgroup analyses of the association of the 3 levels of MedHi food intake with all-cause mortality among cancer patients. The model was adjusted for age, sex, race, marital status, BMI, PIR group, educational level, HEI-2015, physical activity, smoking status, alcohol intake, CVD, hypertension, hyperlipidemia, and diabetes. BMI = body mass index, CVD, cardiovascular disease, HEI-2015 = healthy eating index-2015, MedHi = medium-to-high microbial content food, PIR = poverty income ratio.

As shown in Figure 4, subgroup analyses indicated consistent effects of the 3 MedHi food intake groups on all-cause mortality across different subgroups defined by age, sex, race, marital status, and BMI. Specifically, in all subgroups, the G3 group demonstrated significantly lower all-cause mortality. No significant interactions were observed in any subgroup.

### 3.4. Sensitivity analysis

After excluding participants who died within the first 2 years of follow-up, G3 continued to show a 21% reduction in all-cause mortality risk compared to G1 (HR = 0.79, 95% CI: 0.67–0.93, *P* = .004). The results from the multiple-imputation analysis for missing data were consistent with the primary analysis, further confirming the robustness of the study findings (HR = 0.89, 95% CI: 0.83–0.96, *P* = .002). These results indicate that the observed associations were not materially influenced by energy intake adjustment or accounting for the complex survey design (see Supplementary Table 3, Supplemental Digital Content 3 and Supplementary Table 4, Supplemental Digital Content 4).

## 4. Discussion

This study is the first to systematically investigate the association between dietary live microbe intake from food and all-cause and cause-specific mortality among cancer patients. The results indicate that higher MedHi food intake is significantly associated with reduced all-cause mortality and noncancer mortality, although no significant association was observed with cancer-specific mortality. These findings provide new evidence supporting the potential role of dietary live microbes as an intervention for improving cancer prognosis and indicate important directions for future research.

Recent evidence indicates that regular consumption of probiotic-rich foods, such as yogurt, may confer significant health benefits to both the general population and individuals with cancer. A large, population-based cohort study in the United States demonstrated that higher yogurt intake was associated with a lower risk of all-cause mortality, potentially through mechanisms involving gut microbiota modulation and immune system enhancement.^[21]^ In parallel, a comprehensive meta-analysis by Tutunchi et al revealed that increased yogurt consumption was substantially associated with reduced all-cause and CVD mortality, although its impact on cancer-specific mortality remained less definitive.^[22]^ These observations suggest that the probiotic content of yogurt, along with its favorable nutrient profile, may help improve metabolic health, strengthen the gut epithelial barrier, and mitigate systemic inflammation. Each of these factors plays a crucial role in cancer progression and in optimizing patient outcomes.

Building on these findings, researchers have also explored the broader impact of dietary live microbes on various health outcomes, including cardiovascular disease, metabolic syndrome, and immune function. For instance, Liang et al found that higher MedHi food intake was significantly associated with reduced all-cause mortality and cardiovascular-specific mortality.^[23]^ This relationship was also confirmed in our study, particularly regarding noncancer mortality among cancer patients. However, similar to the study by Liang et al, we did not observe a significant association between higher MedHi food intake and cancer-specific mortality, which may suggest that the causes of mortality in cancer patients are more complex, potentially involving cancer type, treatment modalities, and other unmeasured confounding factors.^[24]^ Notably, although our estimated HR showed a slight protective trend, the relatively low number of cancer-specific deaths in our sample may have limited the statistical power to detect a significant association.

Additionally, other studies have supported the positive effects of dietary live microbes on metabolic health. For example, Fan et al found a significant inverse association between the consumption of microbe-rich foods and the prevalence of nonalcoholic fatty liver disease (NAFLD), highlighting the potential role of dietary live microbes in managing metabolic health, particularly in cancer patients.^[25]^ Similarly, Huo et al reported that high dietary live microbe intake from food was associated with a lower risk of abdominal aortic calcification, further suggesting the broad protective effects of dietary live microbes on cardiovascular health.^[26]^ It is also noteworthy that Wang et al found an association between consumption of foods containing higher levels of live microbes and lower prevalence of depressive symptoms. This finding on mental health could potentially explain the observed reduction in noncancer mortality in our study, as mental health plays a critical role in the overall survival and quality of life of cancer patients.^[13]^

A notable aspect of this study is the use of RCS regression models, which suggested a nonlinear association between MedHi food intake and all-cause mortality. In the 2-piecewise Cox regression model, the association appeared to be stronger below the model-derived threshold of 489.7 g/day, whereas the association plateaued above this point. However, this threshold should be interpreted as an exploratory, data-driven estimate rather than as an optimal intake target or clinical recommendation. In contrast, Liang et al also used RCS regression models to explore the relationship between MedHi food intake and all-cause mortality but did not identify a significant threshold, suggesting a linear relationship.^[23]^ The differences between these findings may stem from various factors, including sample characteristics, study design, exposure distribution, or analytical approaches. Therefore, the observed threshold may help generate hypotheses regarding the potential dose–response pattern of MedHi food intake, but it requires confirmation in future prospective studies and randomized controlled trials before being used to guide dietary recommendations for cancer survivors.

Dietary live microbes may influence survival in cancer patients through several mechanisms. First, dietary live microbes can modulate the gut microbiota, enhance host immune responses, and reduce systemic inflammation, which is particularly crucial in cancer patients.^[27]^ Additionally, metabolites produced by dietary live microbes, such as short-chain fatty acids, help maintain intestinal barrier function, thereby reducing the absorption and dissemination of carcinogenic substances.^[28]^ The gut-brain axis is another potential mechanism by which dietary live microbes might impact cancer prognosis, as improving mental health could indirectly enhance the quality of life and survival rates of cancer patients.^[29,30]^ However, although MedHi food were conceptualized as indicators of higher dietary live microbe exposure, these foods also differ in nutrient composition and may reflect broader dietary patterns or health behaviors. Despite adjustment for HEI-2015 and other lifestyle factors, residual confounding by overall diet quality or unmeasured health-related behaviors cannot be fully excluded. Therefore, the observed associations should not be interpreted as evidence of a direct microbial effect.

This study is based on NHANES data from 1999 to 2018, encompassing a large, nationally representative sample of U.S. adults. This large-scale dataset not only enhances the external validity of our findings but also improves the ability to generalize the association between dietary live microbe intake from food and mortality across different populations. We employed RCS regression models to identify the nonlinear relationship and threshold between MedHi food intake and all-cause mortality among cancer patients, providing important evidence for developing more precise dietary recommendations in clinical and public health settings. The analysis adjusted for a wide range of potential confounders, including age, sex, BMI, and HEI-2015, ensuring the accuracy of the results and the validity of the association between MedHi food intake and mortality.

We acknowledge several limitations in this study. Although we used prospective cohort data, baseline dietary intake was assessed using cross-sectional 24-hour recall surveys, which limits causal inference. Reverse causation is also a potential concern, particularly among cancer survivors, as individuals with more advanced disease may reduce food intake prior to death. To address this issue, we conducted a sensitivity analysis excluding participants who died within the first 2 years of follow-up. The associations remained materially unchanged, suggesting that reverse causation is unlikely to fully account for the observed findings. Additionally, dietary live microbe intake was based on self-reported data and estimated microbial classifications rather than direct microbiological measurements, which may introduce measurement error. Although NHANES is nationally representative, the final analytic sample of cancer survivors may not fully capture the diversity of the broader population, limiting generalizability. Furthermore, participants with missing dietary or covariate data were excluded, which may introduce selection bias if excluded individuals differed systematically from those included. Importantly, NHANES does not provide detailed information on cancer type, stage, treatment modalities, or time since diagnosis. These factors may substantially influence both dietary behavior and survival outcomes. Residual confounding by cancer severity, treatment-related factors, overall diet quality, or unmeasured health behaviors therefore cannot be excluded. In addition, formal competing-risk or subdistribution hazard analyses were not performed, and deaths from other causes may have acted as competing events in the analyses of cause-specific mortality. Therefore, these findings should not be interpreted as a basis for recommending increased intake of MedHi food for improving cancer prognosis without confirmation from randomized clinical trials or prospective studies with repeated exposure assessment.

In addition to the aforementioned limitations, future studies would benefit from prospective interventional designs, such as randomized controlled trials, to more robustly evaluate the causality between dietary live microbes and cancer outcomes. Mechanistic investigations are also warranted (potentially employing multi-omics approaches, including metagenomics and metabolomics) to elucidate how dietary live microbes may modulate gut microbiota, systemic inflammation, and immune responses across different cancer types or treatment modalities. Such studies could provide deeper insights into optimal dietary microbe intake thresholds, refine personalized nutrition strategies, and ultimately improve long-term clinical outcomes for cancer patients.

## 5. Conclusion

This study demonstrates that high dietary live microbe intake from food is significantly associated with lower all-cause and noncancer mortality in cancer patients, although no significant association was observed with cancer-specific mortality. However, causal inference cannot be established, and randomized trials and longitudinal studies are required before clinical recommendations can be made.

## Acknowledgments

We thank Huanxian Liu (Department of Neurology, Chinese PLA General Hospital, Beijing, China) for his valuable comments on the study design and manuscript.

## Author contributions

**Conceptualization:** Jingyang Zeng, Xiaobo Liu, Yingxuan Huang, Yisen Huang.

**Data curation:** Jingyang Zeng, Xiaobo Liu, Yingxuan Huang, Yisen Huang.

**Methodology:** Yingxuan Huang.

**Software:** Yisen Huang.

**Supervision:** Yubin Wang, Xiaoqiang Liu.

**Writing – original draft:** Jingyang Zeng, Xiaobo Liu.

**Writing – review & editing:** Yubin Wang, Xiaoqiang Liu.








