# Supplementary material for: Association between high dietary intake of live microbes from food and all-cause and cause-specific mortality in cancer patients: A prospective cohort study
Source: Medicine (Baltimore). 2026 Jul 10;105(28):e49649. doi: 10.1097/MD.0000000000049649 (PMC13363040; doi:10.1097/MD.0000000000049649)
Supplement: Supplementary file 1 [file medi-105-e49649-s001.pdf]

A classification system for defining and estimating dietary intake of live microbes in US adults and children. Marco et al. Online Supplementary Material

Foodcode	Description	Assigned category	Subgroup
11100000	Milk, NFS	Lo	Milk
11111000	Milk, whole	Lo	Milk
11111100	Milk, low sodium, whole	Lo	Milk
11111150	Milk, calcium fortified, whole	Lo	Milk
11111160	Milk, calcium fortified, low fat (1%)	Lo	Milk
11111170	Milk, calcium fortified, fat free (skim	Lo	Milk
11112000	Milk, cow's, fluid, other than whole,	Lo	Milk
11112110	Milk, reduced fat (2%)	Lo	Milk
11112210	Milk, low fat (1%)	Lo	Milk
11113000	Milk, fat free (skim)	Lo	Milk
11114300	Milk, lactose free, low fat (1%)	Lo	Milk
11114320	Milk, lactose free, fat free (skim)	Lo	Milk
11114330	Milk, lactose free, reduced fat (2%)	Lo	Milk
11114350	Milk, lactose free, whole	Lo	Milk
11116000	Goat's milk, whole	Lo	Milk
11120000	Milk, dry, reconstituted, NS as to fat	Lo	Milk
11121100	Milk, dry, reconstituted, whole	Lo	Milk
11121210	Milk, dry, reconstituted, low fat (1%)	Lo	Milk
11121300	Milk, dry, reconstituted, fat free (ski	Lo	Milk
11210000	Milk, evaporated, NS as to fat conte	Lo	Milk
11210050	Milk, evaporated, NS as to fat conte	Lo	Milk
11210100	Milk, evaporated, NS as to fat conte	Lo	Milk
11211000	Milk, evaporated, whole, NS as to d	Lo	Milk
11211050	Milk, evaporated, whole	Lo	Milk
11211100	Milk, evaporated, whole, undiluted	Lo	Milk
11211200	Milk, evaporated, whole, diluted	Lo	Milk
11211400	Milk, evaporated, reduced fat (2%)	Lo	Milk
11212000	Milk, evaporated, skim, NS as to dil	Lo	Milk
11212050	Milk, evaporated, fat free (skim)	Lo	Milk
11212100	Milk, evaporated, skim, undiluted	Lo	Milk
11220000	Milk, condensed, sweetened	Lo	Flavored Milk
11220100	Milk, condensed, sweetened, undilu	Lo	Flavored Milk
11220200	Milk, condensed, sweetened, dilute	Lo	Flavored Milk
11310000	Milk, imitation, fluid, soy based	Lo	Dairy Drinks and Substitutes
11320000	Soy milk	Lo	Dairy Drinks and Substitutes
11320100	Soy milk, light	Lo	Dairy Drinks and Substitutes
11320200	Soy milk, nonfat	Lo	Dairy Drinks and Substitutes
11321000	Soy milk, chocolate	Lo	Dairy Drinks and Substitutes
11321100	Soy milk, light, chocolate	Lo	Dairy Drinks and Substitutes
11321200	Soy milk, nonfat, chocolate	Lo	Dairy Drinks and Substitutes
11330000	Milk, soy, dry, reconstituted, not bak	Lo	Dairy Drinks and Substitutes
11340000	Imitation milk, non-soy, sweetened	Lo	Dairy Drinks and Substitutes
11350000	Almond milk, sweetened	Lo	Dairy Drinks and Substitutes
11350010	Almond milk, sweetened, chocolate	Lo	Dairy Drinks and Substitutes
11350020	Almond milk, unsweetened	Lo	Dairy Drinks and Substitutes
11350030	Almond milk, unsweetened, chocola	Lo	Dairy Drinks and Substitutes
11360000	Rice milk	Lo	Dairy Drinks and Substitutes
11370000	Coconut milk	Lo	Dairy Drinks and Substitutes
11511000	Chocolate milk, NFS	Lo	Flavored Milk

11511100	Chocolate milk, ready to drink, who	Lo	Flavored Milk
11511200	Chocolate milk, ready to drink, redu	Lo	Flavored Milk
11511300	Chocolate milk, ready to drink, fat fr	Lo	Flavored Milk
11511400	Chocolate milk, ready to drink, low f	Lo	Flavored Milk
11511550	Chocolate milk, ready to drink, redu	Lo	Flavored Milk
11511600	Chocolate milk, ready to drink, low f	Lo	Flavored Milk
11511610	Chocolate milk, ready to drink, fat fr	Lo	Flavored Milk
11511700	Chocolate milk, ready to drink, low f	Lo	Flavored Milk
11512000	Cocoa, hot chocolate, not from dry	Lo	Flavored Milk
11512010	Hot chocolate / Cocoa, ready to drin	Lo	Flavored Milk
11512020	Hot chocolate / Cocoa, ready to drin	Lo	Flavored Milk
11512030	Hot chocolate / Cocoa, ready to drin	Lo	Dairy Drinks and Substitutes
11512100	Hot chocolate / Cocoa, ready to drin	Lo	Flavored Milk
11512110	Hot chocolate / Cocoa, ready to drin	Lo	Flavored Milk
11512120	Hot chocolate / Cocoa, ready to drin	Lo	Dairy Drinks and Substitutes
11512500	Hot chocolate, Puerto Rican style, r	Lo	Flavored Milk
11512510	Hot chocolate, Puerto Rican style, r	Lo	Flavored Milk
11513000	Chocolate milk, made from dry mix,	Lo	Flavored Milk
11513100	Chocolate milk, made from dry mix	Lo	Flavored Milk
11513150	Chocolate milk, made from dry mix	Lo	Flavored Milk
11513200	Chocolate milk, made from dry mix	Lo	Flavored Milk
11513300	Chocolate milk, made from dry mix	Lo	Flavored Milk
11513310	Chocolate milk, made from dry mix	Lo	Dairy Drinks and Substitutes
11513355	Chocolate milk, made from reduced	Lo	Flavored Milk
11513360	Chocolate milk, made from reduced	Lo	Flavored Milk
11513365	Chocolate milk, made from reduced	Lo	Flavored Milk
11513380	Chocolate milk, made from dry mix,	Lo	Flavored Milk
11513381	Chocolate milk, made from dry mix	Lo	Flavored Milk
11513382	Chocolate milk, made from dry mix	Lo	Flavored Milk
11513383	Chocolate milk, made from dry mix	Lo	Flavored Milk
11513384	Chocolate milk, made from dry mix	Lo	Flavored Milk
11513391	Chocolate milk, made from no suga	Lo	Flavored Milk
11513392	Chocolate milk, made from no suga	Lo	Flavored Milk
11513393	Chocolate milk, made from no suga	Lo	Flavored Milk
11513394	Chocolate milk, made from no suga	Lo	Flavored Milk
11513395	Chocolate milk, made from no suga	Lo	Dairy Drinks and Substitutes
11513400	Chocolate milk, made from syrup, N	Lo	Flavored Milk
11513500	Chocolate milk, made from syrup w	Lo	Flavored Milk
11513550	Chocolate milk, made from syrup w	Lo	Flavored Milk
11513600	Chocolate milk, made from syrup w	Lo	Flavored Milk
11513700	Chocolate milk, made from syrup w	Lo	Flavored Milk
11513750	Chocolate milk, made from syrup w	Lo	Dairy Drinks and Substitutes
11513801	Chocolate milk, made from light syr	Lo	Flavored Milk
11513802	Chocolate milk, made from light syr	Lo	Flavored Milk
11513803	Chocolate milk, made from light syr	Lo	Flavored Milk
11513804	Chocolate milk, made from light syr	Lo	Flavored Milk
11513805	Chocolate milk, made from light syr	Lo	Dairy Drinks and Substitutes
11513851	Chocolate milk, made from sugar fr	Lo	Flavored Milk
11513853	Chocolate milk, made from sugar fr	Lo	Flavored Milk
11514100	Hot chocolate / Cocoa, made with d	Lo	Flavored Milk
11514110	Hot chocolate / Cocoa, made with d	Lo	Flavored Milk

11514120	Hot chocolate / Cocoa, made with d	Lo	Flavored Milk
11514130	Hot chocolate / Cocoa, made with d	Lo	Flavored Milk
11514140	Hot chocolate / Cocoa, made with d	Lo	Flavored Milk
11514150	Hot chocolate / Cocoa, made with d	Lo	Dairy Drinks and Substitutes
11514300	Cocoa with nonfat dry milk and low	Lo	Flavored Milk
11514310	Hot chocolate / Cocoa, made with n	Lo	Flavored Milk
11514320	Hot chocolate / Cocoa, made with n	Lo	Flavored Milk
11514330	Hot chocolate / Cocoa, made with n	Lo	Flavored Milk
11514340	Hot chocolate / Cocoa, made with n	Lo	Flavored Milk
11514350	Hot chocolate / Cocoa, made with n	Lo	Flavored Milk
11514360	Hot chocolate / Cocoa, made with n	Lo	Dairy Drinks and Substitutes
11514500	Cocoa, whey, and low calorie swee	Lo	Dairy Drinks and Substitutes
11516000	Cocoa, whey, and low-calorie swee	Lo	Flavored Milk
11518000	Milk beverage with nonfat dry milk a	Lo	Dairy Drinks and Substitutes
11519000	Milk beverage, made with whole mi	Lo	Flavored Milk
11519040	Strawberry milk, NFS	Lo	Flavored Milk
11519050	Strawberry milk, whole	Lo	Flavored Milk
11519105	Strawberry milk, reduced fat	Lo	Flavored Milk
11519200	Strawberry milk, low fat	Lo	Flavored Milk
11519205	Strawberry milk, fat free	Lo	Flavored Milk
11519210	Strawberry milk, reduced sugar	Lo	Flavored Milk
11519215	Strawberry milk, non-dairy	Lo	Dairy Drinks and Substitutes
11520000	Milk, malted, unfortified, NS as to fla	Lo	Flavored Milk
11521000	Milk, malted, unfortified, chocolate,	Lo	Flavored Milk
11522000	Milk, malted, unfortified, natural flav	Lo	Flavored Milk
11525000	Milk, malted, natural flavor, made w	Lo	Flavored Milk
11526000	Milk, malted	Lo	Flavored Milk
11527000	Milk, malted, fortified, NS as to flavo	Lo	Flavored Milk
11531000	Eggnog	Lo	Dairy Drinks and Substitutes
11531500	Eggnog, lowfat / light	Lo	Dairy Drinks and Substitutes
11541000	Milk shake, NS as to flavor or type	Lo	Dairy Drinks and Substitutes
11541100	Milk shake, homemade or fountain-	Lo	Dairy Drinks and Substitutes
11541110	Milk shake, home recipe, chocolate	Lo	Dairy Drinks and Substitutes
11541120	Milk shake, home recipe, flavors oth	Lo	Dairy Drinks and Substitutes
11541130	Milk shake, home recipe, chocolate	Lo	Dairy Drinks and Substitutes
11541135	Milk shake, home recipe, flavors oth	Lo	Dairy Drinks and Substitutes
11541400	Milk shake with malt	Lo	Dairy Drinks and Substitutes
11541500	Milk shake, made with skim milk, ch	Lo	Dairy Drinks and Substitutes
11541510	Milk shake, made with skim milk, fla	Lo	Dairy Drinks and Substitutes
11542100	Milk shake, fast food, chocolate	Lo	Dairy Drinks and Substitutes
11542200	Milk shake, fast food, flavors other t	Lo	Dairy Drinks and Substitutes
11543000	Milk shake, bottled, chocolate	Lo	Dairy Drinks and Substitutes
11543010	Milk shake, bottled, flavors other tha	Lo	Dairy Drinks and Substitutes
11551050	Licuado or Batido	Lo	Sweetened Beverages
11552200	Orange Julius	Lo	Sweetened Beverages
11553130	Fruit smoothie juice drink, with dairy	Lo	Sweetened Beverages
11560000	Chocolate milk drink	Lo	Dairy Drinks and Substitutes
11560020	Flavored milk drink, whey- and milk	Lo	Dairy Drinks and Substitutes
11561000	Cafe con leche	Lo	Coffee and Tea
11561010	Cafe con leche prepared with sugar	Lo	Coffee and Tea
11611000	Instant breakfast, fluid, canned	Lo	Sweetened Beverages

11612000	Instant breakfast, powder, milk add	Lo	Sweetened Beverages
11613000	Instant breakfast, powder, sweeten	Lo	Sweetened Beverages
11623000	Meal supplement or replacement, c	Lo	Sweetened Beverages
11631000	High calorie beverage, canned or p	Lo	Sweetened Beverages
11641000	Meal supplement or replacement, n	Lo	Sweetened Beverages
11641020	Meal replacement or supplement, n	Lo	Sweetened Beverages
11710000	Infant formula, NFS	Lo	Infant Formulas
11710050	Infant formula, NS as to form (Simil	Lo	Infant Formulas
11710051	Infant formula, ready-to-feed (Simila	Lo	Infant Formulas
11710053	Infant formula, powder, made with w	Lo	Infant Formulas
11710054	Infant formula, powder, made with t	Lo	Infant Formulas
11710055	Infant formula, powder, made with p	Lo	Infant Formulas
11710056	Infant formula, powder, made with b	Lo	Infant Formulas
11710061	Similac Alimentum Advance, with ir	Lo	Infant Formulas
11710063	Similac Alimentum Advance, with ir	Lo	Infant Formulas
11710100	Enfamil, low iron, infant formula, NS	Lo	Infant Formulas
11710102	Enfamil, low iron, infant formula, pre	Lo	Infant Formulas
11710103	Enfamil, low iron, infant formula, pre	Lo	Infant Formulas
11710200	Similac, low iron, infant formula, NS	Lo	Infant Formulas
11710201	Similac, low iron, infant formula, rea	Lo	Infant Formulas
11710202	Similac, low iron, infant formula, pre	Lo	Infant Formulas
11710203	Similac, low iron, infant formula, pre	Lo	Infant Formulas
11710250	Similac Natural Care Advance, low	Lo	Infant Formulas
11710350	Infant formula, NS as to form (Simil	Lo	Infant Formulas
11710351	Infant formula, ready-to-feed (Simila	Lo	Infant Formulas
11710352	Infant formula, liquid concentrate, m	Lo	Infant Formulas
11710353	Infant formula, powder, made with w	Lo	Infant Formulas
11710354	Infant formula, liquid concentrate, m	Lo	Infant Formulas
11710355	Infant formula, liquid concentrate, m	Lo	Infant Formulas
11710356	Infant formula, liquid concentrate, m	Lo	Infant Formulas
11710357	Infant formula, powder, made with t	Lo	Infant Formulas
11710358	Infant formula, powder, made with p	Lo	Infant Formulas
11710359	Infant formula, powder, made with b	Lo	Infant Formulas
11710367	Infant formula, powder, made with t	Lo	Infant Formulas
11710369	Infant formula, powder, made with b	Lo	Infant Formulas
11710370	Infant formula, NS as to form (Simil	Lo	Infant Formulas
11710371	Infant formula, ready-to-feed (Simila	Lo	Infant Formulas
11710373	Infant formula, powder, made with w	Lo	Infant Formulas
11710374	Infant formula, liquid concentrate, m	Lo	Infant Formulas
11710376	Infant formula, liquid concentrate, m	Lo	Infant Formulas
11710377	Infant formula, powder, made with t	Lo	Infant Formulas
11710378	Infant formula, powder, made with p	Lo	Infant Formulas
11710379	Infant formula, powder, made with b	Lo	Infant Formulas
11710380	Infant formula, NS as to form (Simil	Lo	Infant Formulas
11710381	Infant formula, ready-to-feed (Simila	Lo	Infant Formulas
11710383	Infant formula, powder, made with w	Lo	Infant Formulas
11710387	Similac Sensitive for Spit-Up, infant	Lo	Infant Formulas
11710388	Similac Sensitive for Spit-Up, infant	Lo	Infant Formulas
11710389	Similac Sensitive for Spit-Up, infant	Lo	Infant Formulas
11710400	Similac, with iron, infant formula, NS	Lo	Infant Formulas
11710401	Similac, with iron, infant formula, re	Lo	Infant Formulas

11710402	Similac, with iron, infant formula, pr	Lo	Infant Formulas
11710403	Similac, with iron, infant formula, pr	Lo	Infant Formulas
11710451	Similac Special Care Advance 24, v	Lo	Infant Formulas
11710470	Infant formula, NS as to form (Simil	Lo	Infant Formulas
11710471	Infant formula, ready-to-feed (Simila	Lo	Infant Formulas
11710473	Infant formula, powder, made with v	Lo	Infant Formulas
11710477	Infant formula, powder, made with t	Lo	Infant Formulas
11710478	Infant formula, powder, made with p	Lo	Infant Formulas
11710479	Infant formula, powder, made with b	Lo	Infant Formulas
11710480	Infant formula, NS as to form (Simil	Lo	Infant Formulas
11710481	Infant formula, powder, made with v	Lo	Infant Formulas
11710482	Similac Go and Grow, infant formula	Lo	Infant Formulas
11710484	Similac Go and Grow, infant formula	Lo	Infant Formulas
11710600	Enfamil, with iron, infant formula, NS	Lo	Infant Formulas
11710601	Enfamil, with iron, infant formula, re	Lo	Infant Formulas
11710602	Enfamil, with iron, infant formula, pr	Lo	Infant Formulas
11710603	Enfamil, with iron, infant formula, pr	Lo	Infant Formulas
11710627	Infant formula, powder, made with t	Lo	Infant Formulas
11710628	Infant formula, powder, made with p	Lo	Infant Formulas
11710629	Infant formula, powder, made with b	Lo	Infant Formulas
11710631	Infant formula, ready-to-feed (Enfar	Lo	Infant Formulas
11710633	Infant formula, liquid concentrate, m	Lo	Infant Formulas
11710634	Infant formula, liquid concentrate, m	Lo	Infant Formulas
11710635	Infant formula, liquid concentrate, m	Lo	Infant Formulas
11710637	Infant formula, powder, made with t	Lo	Infant Formulas
11710638	Infant formula, powder, made with p	Lo	Infant Formulas
11710639	Infant formula, powder, made with b	Lo	Infant Formulas
11710640	Enfamil PREMIUM LIPIL, infant forr	Lo	Infant Formulas
11710643	Enfamil PREMIUM LIPIL, infant forr	Lo	Infant Formulas
11710644	Enfamil PREMIUM LIPIL, infant forr	Lo	Infant Formulas
11710646	Enfamil PREMIUM LIPIL, infant forr	Lo	Infant Formulas
11710647	Enfamil PREMIUM LIPIL, infant forr	Lo	Infant Formulas
11710648	Enfamil PREMIUM LIPIL, infant forr	Lo	Infant Formulas
11710649	Enfamil PREMIUM LIPIL, infant forr	Lo	Infant Formulas
11710650	Enfamil LIPIL, infant formula, NS as	Lo	Infant Formulas
11710651	Enfamil LIPIL, infant formula, ready	Lo	Infant Formulas
11710652	Enfamil LIPIL, infant formula, prepa	Lo	Infant Formulas
11710653	Enfamil LIPIL, infant formula, prepa	Lo	Infant Formulas
11710654	Enfamil LIPIL, infant formula, prepa	Lo	Infant Formulas
11710656	Enfamil LIPIL, infant formula, prepa	Lo	Infant Formulas
11710657	Enfamil LIPIL, infant formula, prepa	Lo	Infant Formulas
11710658	Enfamil LIPIL, infant formula, prepa	Lo	Infant Formulas
11710659	Enfamil LIPIL, infant formula, prepa	Lo	Infant Formulas
11710660	Infant formula, NS as to form (Enfa	Lo	Infant Formulas
11710661	Infant formula, ready-to-feed (Enfar	Lo	Infant Formulas
11710663	Infant formula, powder, made with v	Lo	Infant Formulas
11710664	Infant formula, powder, made with t	Lo	Infant Formulas
11710667	Infant formula, powder, made with v	Lo	Infant Formulas
11710668	Infant formula, powder, made with p	Lo	Infant Formulas
11710669	Infant formula, powder, made with b	Lo	Infant Formulas
11710671	Infant formula, ready-to-feed (Enfar	Lo	Infant Formulas

11710673	Infant formula, powder, made with v	Lo	Infant Formulas
11710674	Infant formula, powder, made with t	Lo	Infant Formulas
11710675	Infant formula, powder, made with p	Lo	Infant Formulas
11710677	Infant formula, powder, made with t	Lo	Infant Formulas
11710678	Infant formula, powder, made with p	Lo	Infant Formulas
11710679	Infant formula, powder, made with b	Lo	Infant Formulas
11710681	Infant formula, ready-to-feed (Enfar	Lo	Infant Formulas
11710683	Infant formula, powder, made with v	Lo	Infant Formulas
11710687	Infant formula, powder, made with t	Lo	Infant Formulas
11710688	Infant formula, powder, made with p	Lo	Infant Formulas
11710689	Infant formula, powder, made with b	Lo	Infant Formulas
11710690	Infant formula, NS as to form (Enfa	Lo	Infant Formulas
11710697	Infant formula, powder, made with t	Lo	Infant Formulas
11710698	Infant formula, powder, made with p	Lo	Infant Formulas
11710699	Infant formula, powder, made with b	Lo	Infant Formulas
11710710	Lactofree, with iron, infant formula,	Lo	Infant Formulas
11710711	Lactofree, with iron, infant formula,	Lo	Infant Formulas
11710712	Lactofree, with iron, infant formula,	Lo	Infant Formulas
11710713	Lactofree, with iron, infant formula,	Lo	Infant Formulas
11710720	Enfamil LactoFree Lipil, with iron, in	Lo	Infant Formulas
11710721	Enfamil LactoFree Lipil, with iron, in	Lo	Infant Formulas
11710722	Enfamil LactoFree Lipil, with iron, in	Lo	Infant Formulas
11710723	Enfamil LactoFree Lipil, with iron, in	Lo	Infant Formulas
11710800	Infant formula, NS as to form (Pedia	Lo	Infant Formulas
11710801	Infant formula, ready-to-feed (Pedia	Lo	Infant Formulas
11710805	Infant formula, with fiber, NS as to f	Lo	Infant Formulas
11710806	Infant formula, with fiber, ready-to-fe	Lo	Infant Formulas
11710900	Good Start Supreme, with iron, infa	Lo	Infant Formulas
11710901	Good Start Supreme, with iron, infa	Lo	Infant Formulas
11710902	Good Start Supreme, with iron, infa	Lo	Infant Formulas
11710903	Good Start Supreme, with iron, infa	Lo	Infant Formulas
11710910	Infant formula, NS as to form (Gerb	Lo	Infant Formulas
11710911	Infant formula, ready-to-feed (Gerbe	Lo	Infant Formulas
11710912	Infant formula, liquid concentrate, m	Lo	Infant Formulas
11710913	Infant formula, powder, made with v	Lo	Infant Formulas
11710916	Infant formula, liquid concentrate, m	Lo	Infant Formulas
11710917	Infant formula, powder, made with t	Lo	Infant Formulas
11710918	Infant formula, powder, made with p	Lo	Infant Formulas
11710919	Infant formula, powder, made with b	Lo	Infant Formulas
11710927	Infant formula, powder, made with t	Lo	Infant Formulas
11710928	Infant formula, powder, made with p	Lo	Infant Formulas
11710929	Infant formula, powder, made with b	Lo	Infant Formulas
11710930	Infant formula, NS as to form (Gerb	Lo	Infant Formulas
11710937	Gerber Good Start 2 Gentle Plus, in	Lo	Infant Formulas
11710938	Gerber Good Start 2 Gentle Plus, in	Lo	Infant Formulas
11710949	Gerber Good Start 2 Protect Plus, i	Lo	Infant Formulas
11710951	Good Start 2 Essentials, with iron, i	Lo	Infant Formulas
11710953	Good Start 2 Essentials, with iron, i	Lo	Infant Formulas
11710962	Infant formula, powder, made with v	Lo	Infant Formulas
11710963	Infant formula, ready-to-feed (Store	Lo	Infant Formulas
11710964	Infant formula, liquid concentrate, m	Lo	Infant Formulas

11710966	Infant formula, liquid concentrate, m	Lo	Infant Formulas
11710967	Infant formula, powder, made with t	Lo	Infant Formulas
11710968	Infant formula, powder, made with p	Lo	Infant Formulas
11710969	Infant formula, powder, made with b	Lo	Infant Formulas
11720300	Enfamil ProSobee, with iron, infant	Lo	Infant Formulas
11720301	Enfamil ProSobee, with iron, infant	Lo	Infant Formulas
11720302	Enfamil ProSobee, with iron, infant	Lo	Infant Formulas
11720303	Enfamil ProSobee, with iron, infant	Lo	Infant Formulas
11720311	Infant formula, ready-to-feed (Enfar	Lo	Infant Formulas
11720312	Infant formula, liquid concentrate, m	Lo	Infant Formulas
11720313	Infant formula, powder, made with w	Lo	Infant Formulas
11720316	Infant formula, liquid concentrate, m	Lo	Infant Formulas
11720317	Infant formula, powder, made with t	Lo	Infant Formulas
11720318	Infant formula, powder, made with p	Lo	Infant Formulas
11720319	Infant formula, powder, made with b	Lo	Infant Formulas
11720323	Infant formula, powder, made with w	Lo	Infant Formulas
11720328	Enfamil Enfagrow Soy Next Step LI	Lo	Infant Formulas
11720329	Enfamil Enfagrow Soy Next Step LI	Lo	Infant Formulas
11720400	Similac Isomil, with iron, infant form	Lo	Infant Formulas
11720401	Similac Isomil, with iron, infant form	Lo	Infant Formulas
11720402	Similac Isomil, with iron, infant form	Lo	Infant Formulas
11720403	Similac Isomil, with iron, infant form	Lo	Infant Formulas
11720411	Infant formula, ready-to-feed (Simila	Lo	Infant Formulas
11720412	Infant formula, liquid concentrate, m	Lo	Infant Formulas
11720413	Infant formula, powder, made with w	Lo	Infant Formulas
11720414	Infant formula, liquid concentrate, m	Lo	Infant Formulas
11720416	Infant formula, liquid concentrate, m	Lo	Infant Formulas
11720417	Infant formula, powder, made with t	Lo	Infant Formulas
11720418	Infant formula, powder, made with p	Lo	Infant Formulas
11720419	Infant formula, powder, made with b	Lo	Infant Formulas
11720430	Infant formula, NS as to form (Simil	Lo	Infant Formulas
11720431	Infant formula, ready-to-feed (Simila	Lo	Infant Formulas
11720600	Good Start Essentials Soy, with iron	Lo	Infant Formulas
11720601	Good Start Essentials Soy, with iron	Lo	Infant Formulas
11720602	Good Start Essentials Soy, with iron	Lo	Infant Formulas
11720603	Good Start Essentials Soy, with iron	Lo	Infant Formulas
11720612	Infant formula, liquid concentrate, m	Lo	Infant Formulas
11720613	Infant formula, powder, made with w	Lo	Infant Formulas
11720615	Infant formula, liquid concentrate, m	Lo	Infant Formulas
11720617	Infant formula, powder, made with t	Lo	Infant Formulas
11720618	Infant formula, powder, made with p	Lo	Infant Formulas
11720619	Infant formula, powder, made with b	Lo	Infant Formulas
11720620	Infant formula, NS as to form (Gerb	Lo	Infant Formulas
11720628	Gerber Good Start 2 Soy Plus, infar	Lo	Infant Formulas
11720629	Gerber Good Start 2 Soy Plus, infar	Lo	Infant Formulas
11720703	Next Step Prosobee, with iron, infar	Lo	Infant Formulas
11720803	Infant formula, powder, made with w	Lo	Infant Formulas
11720807	Infant formula, powder, made with t	Lo	Infant Formulas
11720808	Infant formula, powder, made with p	Lo	Infant Formulas
11720809	Infant formula, powder, made with b	Lo	Infant Formulas
11740301	Nutramigen, with iron, infant formula	Lo	Infant Formulas

11740302	Nutramigen, with iron, infant formula	Lo	Infant Formulas
11740303	Nutramigen, with iron, infant formula	Lo	Infant Formulas
11740310	Infant formula, NS as to form (Enfa	Lo	Infant Formulas
11740311	Infant formula, ready-to-feed (Enfar	Lo	Infant Formulas
11740312	Infant formula, liquid concentrate, m	Lo	Infant Formulas
11740313	Infant formula, powder, made with w	Lo	Infant Formulas
11740317	Enfamil Nutramigen LIPIL, infant fo	Lo	Infant Formulas
11740318	Enfamil Nutramigen LIPIL, infant fo	Lo	Infant Formulas
11740319	Enfamil Nutramigen LIPIL, infant fo	Lo	Infant Formulas
11740323	Infant formula, powder, made with w	Lo	Infant Formulas
11740329	Enfamil Nutramigen AA LIPIL, infan	Lo	Infant Formulas
11740400	Infant formula, NS as to form (Enfa	Lo	Infant Formulas
11740403	Infant formula, powder, made with w	Lo	Infant Formulas
11740407	Enfmail Pregestimil LIPIL, infant for	Lo	Infant Formulas
11740520	Enfamil Premature LIPIL 20, with iro	Lo	Infant Formulas
11810000	Milk, dry, not reconstituted, NS as to	Lo	Other
11811000	Milk, dry, not reconstituted, whole	Lo	Other
11812000	Milk, dry, not reconstituted, low fat (	Lo	Other
11813000	Milk, dry, not reconstituted, fat free	Lo	Other
11825000	Whey, sweet, dry	Lo	Other
11830100	Hot chocolate / Cocoa, dry mix, not	Lo	Other
11830110	Cocoa powder with nonfat dry milk a	Lo	Other
11830115	Hot chocolate / Cocoa, dry mix, no s	Lo	Other
11830120	Cocoa, whey, and low calorie sweet	Lo	Other
11830140	Chocolate, instant, dry mix, fortified	Lo	Other
11830150	Cocoa powder, not reconstituted	Lo	Other
11830160	Chocolate beverage powder, dry mi	Lo	Other
11830165	Chocolate beverage powder, light, d	Lo	Other
11830170	Cocoa (or chocolate) flavored beve	Lo	Other
11830200	Milk, malted, dry mix, unfortified, no	Lo	Other
11830210	Milk, malted, dry mix, fortified, not re	Lo	Other
11830260	Milk, malted, dry mix, not reconstitu	Lo	Other
11830400	Strawberry beverage powder, dry m	Lo	Other
11830500	Milk beverage, powder, with nonfat	Lo	Other
11830550	Milk beverage, powder, with nonfat	Lo	Other
11830800	Instant breakfast, powder, not recon	Lo	Other
11830810	Instant breakfast, powder, sweeten	Lo	Other
11830850	High calorie milk beverage, powder	Lo	Other
11830900	Protein supplement, milk-based, po	Lo	Other
11830940	Meal replacement, high protein, mil	Lo	Other
11830970	Meal replacement, protein type, mil	Lo	Other
11830990	Nutrient supplement, milk-based, p	Lo	Other
11831500	Nutrient supplement, milk-based, hi	Lo	Other
11832000	Meal replacement, protein type, mil	Lo	Other
11836000	Protein supplement, milk-based, Mu	Lo	Other
11836100	Protein supplement, milk-based, Mu	Lo	Other
12100100	Cream, NS as to light, heavy, or ha	Lo	Fats and Oils
12110100	Cream, light	Lo	Fats and Oils
12110300	Cream, light, whipped, unsweetene	Lo	Fats and Oils
12120100	Cream, half and half	Lo	Fats and Oils
12120105	Cream, half and half, low fat	Lo	Fats and Oils

12120106	Cream, half and half, flavored	Lo	Fats and Oils
12120110	Cream, half and half, fat free	Lo	Fats and Oils
12130100	Cream, heavy	Lo	Fats and Oils
12130200	Cream, heavy, whipped, unsweeten	Lo	Fats and Oils
12140000	Cream, whipped	Lo	Fats and Oils
12140100	Cream, whipped, pressurized conta	Lo	Fats and Oils
12140105	Cream, whipped, pressurized conta	Lo	Fats and Oils
12140110	Whipped topping, dairy based, fat fr	Lo	Fats and Oils
12200100	Coffee creamer, NFS	Lo	Fats and Oils
12210100	Cream substitute, frozen	Lo	Fats and Oils
12210200	Coffee creamer, liquid	Lo	Fats and Oils
12210210	Coffee creamer, liquid, flavored	Lo	Fats and Oils
12210250	Cream substitute, light, liquid	Lo	Fats and Oils
12210255	Cream substitute, light, flavored, liq	Lo	Fats and Oils
12210260	Coffee creamer, liquid, fat free	Lo	Fats and Oils
12210270	Coffee creamer, liquid, fat free, flav	Lo	Fats and Oils
12210280	Coffee creamer, liquid, fat free, sug	Lo	Fats and Oils
12210305	Cream substitute, sugar free, liquid	Lo	Fats and Oils
12210310	Coffee creamer, liquid, sugar free, f	Lo	Fats and Oils
12210400	Coffee creamer, powder	Lo	Fats and Oils
12210410	Cream substitute, light, powdered	Lo	Fats and Oils
12210420	Coffee creamer, powder, flavored	Lo	Fats and Oils
12210430	Coffee creamer, powder, fat free	Lo	Fats and Oils
12210440	Coffee creamer,powder, fat free, fla	Lo	Fats and Oils
12210500	Cream substitute, sugar free, powd	Lo	Fats and Oils
12210505	Coffee creamer,powder, sugar free	Lo	Fats and Oils
12210520	Coffee creamer, soy, liquid	Lo	Fats and Oils
12220000	Whipped topping, nondairy, NS as t	Lo	Fats and Oils
12220100	Whipped topping, nondairy, pressur	Lo	Fats and Oils
12220200	Whipped topping	Lo	Fats and Oils
12220250	Whipped topping, nondairy, frozen,	Lo	Fats and Oils
12220270	Whipped topping, fat free	Lo	Fats and Oils
12220280	Whipped topping, sugar free	Lo	Fats and Oils
12220300	Whipped cream substitute, nondairy	Lo	Fats and Oils
12220400	Whipped cream substitute, nondairy	Lo	Fats and Oils
13110000	Ice cream, NFS	Lo	Other Desserts
13110100	Ice cream, vanilla	Lo	Other Desserts
13110102	Ice cream, vanilla, with additional in	Lo	Other Desserts
13110110	Ice cream, chocolate	Lo	Other Desserts
13110112	Ice cream, chocolate, with additiona	Lo	Other Desserts
13110120	Ice cream, rich, flavors other than c	Lo	Other Desserts
13110130	Ice cream, rich, chocolate	Lo	Other Desserts
13110140	Ice cream, rich, NS as to flavor	Lo	Other Desserts
13110200	Ice cream, soft serve, vanilla	Lo	Other Desserts
13110210	Ice cream, soft serve, chocolate	Lo	Other Desserts
13110220	Ice cream, soft serve, NS as to flav	Lo	Other Desserts
13110310	Ice cream, no sugar added, NS as t	Lo	Other Desserts
13110320	Ice cream, no sugar added, flavors	Lo	Other Desserts
13110330	Ice cream, no sugar added, chocola	Lo	Other Desserts
13110460	Gelato, vanilla	Lo	Other Desserts
13110470	Gelato, chocolate	Lo	Other Desserts

13120050	Ice cream bar, vanilla	Lo	Other Desserts
13120100	Ice cream bar, vanilla, chocolate co	Lo	Other Desserts
13120110	Ice cream candy bar	Lo	Other Desserts
13120120	Ice cream bar or stick, rich chocolat	Lo	Other Desserts
13120121	Ice cream bar or stick, rich ice crea	Lo	Other Desserts
13120130	Ice cream bar or stick, rich ice crea	Lo	Other Desserts
13120140	Ice cream bar, chocolate	Lo	Other Desserts
13120300	Ice cream bar, cake covered	Lo	Other Desserts
13120310	Ice cream bar, stick or nugget, with	Lo	Other Desserts
13120400	Ice cream bar or stick with fruit	Lo	Other Desserts
13120500	Ice cream sandwich, vanilla	Lo	Other Desserts
13120510	Ice cream sandwich, chocolate	Lo	Other Desserts
13120550	Ice cream cookie sandwich	Lo	Other Desserts
13120700	Ice cream cone with nuts, flavors ot	Lo	Other Desserts
13120710	Ice cream cone, chocolate covered	Lo	Other Desserts
13120720	Ice cream cone, chocolate covered	Lo	Other Desserts
13120730	Ice cream cone, scooped, vanilla	Lo	Other Desserts
13120735	Ice cream cone, scooped, vanilla, w	Lo	Other Desserts
13120740	Ice cream cone, NFS	Lo	Other Desserts
13120750	Ice cream cone with nuts, chocolate	Lo	Other Desserts
13120760	Ice cream cone, chocolate covered	Lo	Other Desserts
13120770	Ice cream cone, scooped, chocolate	Lo	Other Desserts
13120775	Ice cream cone, scooped, chocolate	Lo	Other Desserts
13120780	Ice cream cone, chocolate covered	Lo	Other Desserts
13120782	Ice cream cone, soft serve, vanilla	Lo	Other Desserts
13120784	Ice cream cone, soft serve, chocola	Lo	Other Desserts
13120786	Ice cream cone, soft serve, vanilla,	Lo	Other Desserts
13120788	Ice cream cone, soft serve, chocola	Lo	Other Desserts
13120790	Ice cream cone, vanilla, prepackage	Lo	Other Desserts
13120792	Ice cream cone, chocolate, prepack	Lo	Other Desserts
13120800	Ice cream soda, flavors other than c	Lo	Dairy Drinks and Substitutes
13120810	Ice cream soda, chocolate	Lo	Dairy Drinks and Substitutes
13121000	Ice cream sundae, NFS	Lo	Other Desserts
13121100	Ice cream sundae, fruit topping	Lo	Other Desserts
13121120	Banana split	Lo	Other Desserts
13121300	Ice cream sundae, hot fudge toppin	Lo	Other Desserts
13121400	Ice cream sundae, caramel topping	Lo	Other Desserts
13122100	Ice cream pie, no crust	Lo	Other Desserts
13122500	Ice cream pie, with cookie crust, fuc	Lo	Other Desserts
13126000	Ice cream, fried	Lo	Other Desserts
13127000	Dippin' Dots, flash frozen ice cream	Lo	Other Desserts
13127010	Dippin' Dots, flash frozen ice cream	Lo	Other Desserts
13130100	Light ice cream, NFS	Lo	Other Desserts
13130300	Light ice cream, vanilla	Lo	Other Desserts
13130310	Light ice cream, chocolate	Lo	Other Desserts
13130320	Light ice cream, no sugar added, N	Lo	Other Desserts
13130330	Light ice cream, no sugar added, fla	Lo	Other Desserts
13130340	Light ice cream, no sugar added, ch	Lo	Other Desserts
13130590	Light ice cream, soft serve, NS as t	Lo	Other Desserts
13130600	Light ice cream, soft serve, flavors c	Lo	Other Desserts
13130610	Light ice cream, soft serve, chocola	Lo	Other Desserts

13130620	Light ice cream, soft serve cone, fla	Lo	Other Desserts
13130630	Light ice cream, soft serve cone, ch	Lo	Other Desserts
13130640	Light ice cream, soft serve cone, NS	Lo	Other Desserts
13130700	Soft serve, blended with candy or co	Lo	Other Desserts
13135000	Light ice cream sandwich, vanilla	Lo	Other Desserts
13135010	Light ice cream sandwich, chocolate	Lo	Other Desserts
13136000	Ice cream sandwich, made with ligh	Lo	Other Desserts
13140000	Light ice cream bar, vanilla	Lo	Other Desserts
13140100	Light ice cream bar, vanilla, chocola	Lo	Other Desserts
13140110	Light ice cream, bar or stick, chocol	Lo	Other Desserts
13140115	Light ice cream bar, chocolate	Lo	Other Desserts
13140450	Light ice cream, cone, NFS	Lo	Other Desserts
13140500	Light ice cream, cone, flavors other	Lo	Other Desserts
13140550	Light ice cream, cone, chocolate	Lo	Other Desserts
13140575	Light ice cream, no sugar added, co	Lo	Other Desserts
13140580	Light ice cream, no sugar added, co	Lo	Other Desserts
13140650	Light ice cream, sundae, soft serve	Lo	Other Desserts
13140660	Light ice cream, sundae, soft serve	Lo	Other Desserts
13140670	Light ice cream, sundae, soft serve	Lo	Other Desserts
13140680	Light ice cream, sundae, soft serve	Lo	Other Desserts
13140700	Creamsicle	Lo	Other Desserts
13140710	Creamsicle, light	Lo	Other Desserts
13140900	Fudgesicle	Lo	Other Desserts
13142000	Milk dessert bar or stick, frozen, wit	Lo	Other Desserts
13142100	Light ice cream cone, vanilla, prepa	Lo	Other Desserts
13142110	Light ice cream cone, chocolate, pre	Lo	Other Desserts
13150000	Sherbet, all flavors	Lo	Other Desserts
13160150	Fat free ice cream, no sugar added	Lo	Other Desserts
13160160	Fat free ice cream, no sugar added	Lo	Other Desserts
13160400	Fat free ice cream, flavors other tha	Lo	Other Desserts
13160410	Fat free ice cream, chocolate	Lo	Other Desserts
13161000	Milk dessert bar, frozen, made from	Lo	Other Desserts
13161500	Milk dessert sandwich bar, frozen, m	Lo	Other Desserts
13161520	Milk dessert sandwich bar, frozen, w	Lo	Other Desserts
13161600	Fudgesicle, light	Lo	Other Desserts
13161630	Light ice cream, bar or stick, with lo	Lo	Other Desserts
13170000	Baked Alaska	Lo	Other Desserts
13200110	Pudding, chocolate, NFS	Lo	Other Desserts
13210110	Pudding, bread	Lo	Other Desserts
13210180	Pudding, Mexican bread	Lo	Other Desserts
13210220	Pudding, chocolate, NS as to from d	Lo	Other Desserts
13210250	Pudding, chocolate, low calorie, con	Lo	Other Desserts
13210270	Custard, Puerto Rican style	Lo	Other Desserts
13210280	Pudding, flavors other than chocola	Lo	Other Desserts
13210290	Pudding, flavors other than chocola	Lo	Other Desserts
13210300	Custard	Lo	Other Desserts
13210350	Flan	Lo	Other Desserts
13210370	Creme brulee	Lo	Other Desserts
13210410	Pudding, rice	Lo	Other Desserts
13210450	Firni, Indian pudding	Lo	Other Desserts
13210500	Pudding, tapioca, made from home	Lo	Other Desserts

13210520	Pudding, tapioca, made from dry m	Lo	Other Desserts
13210610	Pudding, coconut	Lo	Other Desserts
13210710	Pudding, cornmeal, milk, and molas	Lo	Other Desserts
13210750	Pudding, pumpkin	Lo	Other Desserts
13210810	Pumpkin pudding, Puerto Rican sty	Lo	Other Desserts
13210820	Fresh corn custard, Puerto Rican st	Lo	Other Desserts
13220110	Pudding, flavors other than chocola	Lo	Other Desserts
13220120	Pudding, chocolate, made from dry	Lo	Other Desserts
13220210	Pudding, flavors other than chocola	Lo	Other Desserts
13220220	Pudding, chocolate, made from dry	Lo	Other Desserts
13220230	Pudding, ready-to-eat, chocolate, re	Lo	Other Desserts
13220235	Pudding, ready-to-eat, chocolate, fa	Lo	Other Desserts
13220240	Pudding, ready-to-eat, flavors other	Lo	Other Desserts
13220245	Pudding, ready-to-eat, flavors other	Lo	Other Desserts
13230110	Pudding, flavors other than chocola	Lo	Other Desserts
13230120	Pudding, flavors other than chocola	Lo	Other Desserts
13230130	Pudding, chocolate, ready-to-eat	Lo	Other Desserts
13230140	Pudding, chocolate, ready-to-eat, su	Lo	Other Desserts
13230200	Pudding, ready-to-eat, chocolate ar	Lo	Other Desserts
13230500	Pudding, tapioca, ready-to-eat	Lo	Other Desserts
13230510	Pudding, ready-to-eat, tapioca, fat f	Lo	Other Desserts
13241000	Banana pudding	Lo	Other Desserts
13250000	Mousse	Lo	Other Desserts
13250100	Mousse, not chocolate	Lo	Other Desserts
13250200	Mousse, chocolate, lowfat, reduced	Lo	Other Desserts
13252100	Coconut custard, Puerto Rican style	Lo	Other Desserts
13252200	Milk dessert or milk candy, Puerto R	Lo	Other Desserts
13252500	Barfi or Burfi, Indian dessert	Lo	Other Desserts
13252590	Trifle	Lo	Other Desserts
13252600	Tiramisu	Lo	Sweet Bakery Products
13310000	Custard pudding, flavor other than c	Lo	Baby Foods
13311000	Custard pudding, baby food, flavor c	Lo	Baby Foods
13312000	Custard pudding, baby food, flavor c	Lo	Baby Foods
13411000	White sauce or gravy	Lo	Condiments and Sauces
13412000	Milk gravy, quick gravy	Lo	Condiments and Sauces
14120010	Cheese, Mexican blend	Lo	Cheese
14120020	Cheese, Mexican blend, reduced fa	Lo	Cheese
14131000	Queso Anejo, aged Mexican chees	Lo	Cheese
14131500	Queso Asadero	Lo	Cheese
14132000	Queso Chihuahua	Lo	Cheese
14133000	Queso Fresco	Lo	Cheese
14134000	Queso cotija	Lo	Cheese
14200100	Cheese, cottage, NFS	Lo	Cheese
14201010	Cheese, cottage, creamed, large or	Lo	Cheese
14201200	Cottage cheese, farmer's	Lo	Cheese
14201500	Cheese, Ricotta	Lo	Cheese
14202010	Cheese, cottage, with fruit	Lo	Cheese
14202020	Cheese, cottage, with vegetables	Lo	Cheese
14203010	Cheese, cottage, dry curd	Lo	Cheese
14203020	Cheese, cottage, salted, dry curd	Lo	Cheese
14203510	Puerto Rican white cheese	Lo	Cheese

14204010	Cheese, cottage, low fat	Lo	Cheese
14204020	Cheese, cottage, lowfat, with fruit	Lo	Cheese
14206010	Cheese, cottage, lowfat, low sodium	Lo	Cheese
14207010	Cheese, cottage, lowfat, lactose red	Lo	Cheese
14301010	Cream cheese, regular, plain	Lo	Fats and Oils
14301100	Cream cheese, regular, flavored	Lo	Fats and Oils
14303010	Cream cheese, light	Lo	Fats and Oils
14410100	Cheese, American and Swiss blend	Lo	Cheese
14410110	Cheese, American	Lo	Cheese
14410120	Cheese, American, reduced fat	Lo	Cheese
14410130	Cheese, American, nonfat or fat fre	Lo	Cheese
14410200	Cheese, processed, American or C	Lo	Cheese
14410210	Cheese, American, reduced sodium	Lo	Cheese
14410300	Cheese, processed, American or C	Lo	Cheese
14410330	Cheese spread, American or Chedd	Lo	Cheese
14410350	Cheese, processed, American or C	Lo	Cheese
14410380	Cream cheese spread, fat free	Lo	Fats and Oils
14410400	Cheese, processed, Swiss	Lo	Cheese
14410420	Cheese, processed, Swiss, lowfat	Lo	Cheese
14410500	Cheese, processed cheese food	Lo	Cheese
14410600	Cheese, processed, with vegetables	Lo	Cheese
14420000	Cheese spread, NFS	Lo	Cheese
14420100	Cheese spread, American or Chedd	Lo	Cheese
14420160	Cheese spread, Swiss cheese base	Lo	Cheese
14420200	Cheese spread, cream cheese, reg	Lo	Fats and Oils
14420210	Cheese spread, cream cheese, ligh	Lo	Fats and Oils
14420300	Cheese spread, pressurized can	Lo	Cheese
14502000	Imitation cheese	Lo	Cheese
14502010	Imitation cheese, American or ched	Lo	Cheese
14502040	Imitation cheese, American or ched	Lo	Cheese
14504010	Imitation mozzarella cheese	Lo	Cheese
14610200	Cheese, cottage cheese, with gelat	Lo	Cheese
14610210	Cheese, cottage cheese, with gelat	Lo	Cheese
14610250	Cheese, cottage cheese, with gelat	Lo	Cheese
14610520	Cheese ball	Lo	Cheese
14620110	Artichoke dip	Lo	Condiments and Sauces
14620130	Seafood dip	Lo	Condiments and Sauces
14620150	Cheese dip with chili pepper	Lo	Condiments and Sauces
14620200	Cheese dip	Lo	Condiments and Sauces
14620300	Topping from cheese pizza	Lo	Mixed Dishes - Pizza
14620310	Topping from vegetable pizza	Lo	Mixed Dishes - Pizza
14620320	Topping from meat pizza	Lo	Mixed Dishes - Pizza
14620330	Topping from meat and vegetable p	Lo	Mixed Dishes - Pizza
14630100	Cheese fondue	Lo	Condiments and Sauces
14630200	Cheese souffle	Lo	Eggs
14630300	Welsh rarebit	Lo	Condiments and Sauces
14640002	Cheese sandwich, American chees	Lo	Mixed Dishes - Sandwiches (single code)
14640004	Cheese sandwich, American chees	Lo	Mixed Dishes - Sandwiches (single code)
14640006	Cheese sandwich, American chees	Lo	Mixed Dishes - Sandwiches (single code)
14640014	Cheese sandwich, reduced fat Ame	Lo	Mixed Dishes - Sandwiches (single code)
14640018	Cheese sandwich, reduced fat Ame	Lo	Mixed Dishes - Sandwiches (single code)

14640026	Cheese sandwich, American chees	Lo	Mixed Dishes - Sandwiches (single code)
14640028	Cheese sandwich, American chees	Lo	Mixed Dishes - Sandwiches (single code)
14640030	Cheese sandwich, American chees	Lo	Mixed Dishes - Sandwiches (single code)
14640042	Cheese sandwich, reduced fat Ame	Lo	Mixed Dishes - Sandwiches (single code)
14640050	Cheese sandwich, American chees	Lo	Mixed Dishes - Sandwiches (single code)
14640052	Cheese sandwich, American chees	Lo	Mixed Dishes - Sandwiches (single code)
14640054	Cheese sandwich, American chees	Lo	Mixed Dishes - Sandwiches (single code)
14640062	Cheese sandwich, reduced fat Ame	Lo	Mixed Dishes - Sandwiches (single code)
14640100	Grilled cheese sandwich, NFS	Lo	Mixed Dishes - Sandwiches (single code)
14640105	Grilled cheese sandwich, American	Lo	Mixed Dishes - Sandwiches (single code)
14640110	Grilled cheese sandwich, American	Lo	Mixed Dishes - Sandwiches (single code)
14640115	Grilled cheese sandwich, American	Lo	Mixed Dishes - Sandwiches (single code)
14640125	Grilled cheese sandwich, Cheddar	Lo	Mixed Dishes - Sandwiches (single code)
14640130	Grilled cheese sandwich, Cheddar	Lo	Mixed Dishes - Sandwiches (single code)
14640135	Grilled cheese sandwich, Cheddar	Lo	Mixed Dishes - Sandwiches (single code)
14640155	Grilled cheese sandwich, reduced f	Lo	Mixed Dishes - Sandwiches (single code)
14640160	Grilled cheese sandwich, reduced f	Lo	Mixed Dishes - Sandwiches (single code)
14640165	Grilled cheese sandwich, reduced f	Lo	Mixed Dishes - Sandwiches (single code)
14640185	Grilled cheese sandwich, reduced f	Lo	Mixed Dishes - Sandwiches (single code)
14640190	Grilled cheese sandwich, reduced f	Lo	Mixed Dishes - Sandwiches (single code)
14640195	Grilled cheese sandwich, reduced f	Lo	Mixed Dishes - Sandwiches (single code)
14650100	Cheese sauce	Lo	Condiments and Sauces
14650150	Cheese sauce made with lowfat che	Lo	Condiments and Sauces
14650160	Alfredo sauce	Lo	Condiments and Sauces
14660200	Mozzarella sticks, breaded, baked,	Lo	Cheese
14710100	Cheddar cheese soup, home recipe	Lo	Mixed Dishes - Soups
14710200	Beer cheese soup, made with milk	Lo	Mixed Dishes - Soups
20000000	Meat, NFS	Lo	Meats
20000090	Meat sticks, baby food, NS as to typ	Lo	Baby Foods
20000200	Ground meat, NFS	Lo	Meats
21000100	Beef, NS as to cut, cooked, NS as t	Lo	Meats
21000110	Beef, NS as to cut, cooked, lean an	Lo	Meats
21000120	Beef, NS as to cut, cooked, lean on	Lo	Meats
21001000	Steak, NS as to type of meat, cooke	Lo	Meats
21001010	Steak, NS as to type of meat, cooke	Lo	Meats
21001020	Steak, NS as to type of meat, cooke	Lo	Meats
21002000	Beef, pickled	Lo	Meats
21003000	Beef, NS as to cut, fried, NS to fat e	Lo	Meats
21101000	Beef steak, NS as to cooking metho	Lo	Meats
21101010	Beef steak, NS as to cooking metho	Lo	Meats
21101020	Beef steak, NS as to cooking metho	Lo	Meats
21101110	Beef steak, broiled or baked, NS as	Lo	Meats
21101120	Beef steak, broiled or baked, lean a	Lo	Meats
21101130	Beef steak, broiled or baked, lean o	Lo	Meats
21102110	Beef steak, fried, NS as to fat eaten	Lo	Meats
21102120	Beef steak, fried, lean and fat eaten	Lo	Meats
21102130	Beef steak, fried, lean only eaten	Lo	Meats
21103110	Beef steak, breaded or floured, bak	Lo	Meats
21103120	Beef steak, breaded or floured, bak	Lo	Meats
21103130	Beef steak, breaded or floured, bak	Lo	Meats
21104110	Beef steak, battered, fried, NS as to	Lo	Meats

21104120	Beef steak, battered, fried, lean and	Lo	Meats
21104130	Beef steak, battered, fried, lean only	Lo	Meats
21105110	Beef steak, braised, NS as to fat ea	Lo	Meats
21105120	Beef steak, braised, lean and fat ea	Lo	Meats
21105130	Beef steak, braised, lean only eaten	Lo	Meats
21301000	Beef, oxtails, cooked	Lo	Meats
21302000	Beef, neck bones, cooked	Lo	Meats
21304000	Beef, shortribs, cooked, NS as to fa	Lo	Meats
21304110	Beef, shortribs, cooked, lean and fa	Lo	Meats
21304120	Beef, shortribs, cooked, lean only e	Lo	Meats
21304200	Beef, shortribs, barbecued, with sau	Lo	Meats
21304210	Beef, shortribs, barbecued, with sau	Lo	Meats
21304220	Beef, shortribs, barbecued, with sau	Lo	Meats
21305000	Beef, cow head, cooked	Lo	Meats
21401000	Beef, roast, roasted, NS as to fat ea	Lo	Meats
21401110	Beef, roast, roasted, lean and fat ea	Lo	Meats
21401120	Beef, roast, roasted, lean only eaten	Lo	Meats
21401400	Beef, roast, canned	Lo	Meats
21407000	Beef, pot roast, braised or boiled, N	Lo	Meats
21407110	Beef, pot roast, braised or boiled, le	Lo	Meats
21407120	Beef, pot roast, braised or boiled, le	Lo	Meats
21410000	Beef, stew meat, cooked, NS as to	Lo	Meats
21410110	Beef, stew meat, cooked, lean and	Lo	Meats
21410120	Beef, stew meat, cooked, lean only	Lo	Meats
21416000	Corned beef, cooked, NS as to fat e	Lo	Cured Meats/Poultry
21416110	Corned beef, cooked, lean and fat e	Lo	Cured Meats/Poultry
21416120	Corned beef, cooked, lean only eate	Lo	Cured Meats/Poultry
21416150	Corned beef, canned, ready-to-eat	Lo	Cured Meats/Poultry
21417100	Beef brisket, cooked, NS as to fat e	Lo	Meats
21417110	Beef brisket, cooked, lean and fat e	Lo	Meats
21417120	Beef brisket, cooked, lean only eate	Lo	Meats
21420100	Beef, sandwich steak, flaked, forme	Lo	Meats
21500100	Ground beef, cooked	Lo	Meats
21500110	Ground beef, meatballs, meat only,	Lo	Meats
21500200	Ground beef or patty, breaded, coo	Lo	Meats
21500300	Ground beef patty, cooked (for fast	Lo	Meats
21500310	Ground beef patty, cooked	Lo	Meats
21501000	Ground beef, less than 80% lean, c	Lo	Meats
21501200	Ground beef, 80% - 84% lean, cook	Lo	Meats
21501300	Ground beef, 85% - 89% lean, cook	Lo	Meats
21501350	Ground beef, 90% - 94% lean, cook	Lo	Meats
21501360	Ground beef, 95% or more lean, co	Lo	Meats
21540100	Ground beef with textured vegetabl	Lo	Meats
21601000	Beef, bacon, cooked	Lo	Cured Meats/Poultry
21601010	Beef, bacon, reduced sodium, cook	Lo	Cured Meats/Poultry
21601250	Beef, bacon, cooked, lean only eate	Lo	Cured Meats/Poultry
21601500	Beef, bacon, formed, lean meat add	Lo	Cured Meats/Poultry
21602000	Beef, dried, chipped, uncooked	Lo	Cured Meats/Poultry
21602010	Beef, dried, chipped, cooked in fat	Lo	Cured Meats/Poultry
21602100	Beef jerky	Lo	Cured Meats/Poultry
21603000	Beef, pastrami (beef, smoked, spice	Lo	Cured Meats/Poultry

21701010	Beef, baby food, strained	Lo	Baby Foods
21701020	Beef, baby food, junior	Lo	Baby Foods
22000100	Pork, NS as to cut, cooked, NS as t	Lo	Meats
22000110	Pork, NS as to cut, cooked, lean an	Lo	Meats
22000120	Pork, NS as to cut, cooked, lean on	Lo	Meats
22000200	Pork, NS as to cut, fried, NS as to f	Lo	Meats
22000210	Pork, NS as to cut, fried, lean and f	Lo	Meats
22000220	Pork, NS as to cut, fried, lean only e	Lo	Meats
22000300	Pork, NS as to cut, breaded or flour	Lo	Meats
22000310	Pork, NS as to cut, breaded or flour	Lo	Meats
22000320	Pork, NS as to cut, breaded or flour	Lo	Meats
22001000	Pork, pickled, NS as to cut	Lo	Meats
22002000	Pork, ground or patty, cooked	Lo	Meats
22002100	Pork, ground or patty, breaded, coo	Lo	Meats
22002800	Pork jerky	Lo	Cured Meats/Poultry
22101000	Pork chop, NS as to cooking metho	Lo	Meats
22101010	Pork chop, NS as to cooking metho	Lo	Meats
22101020	Pork chop, NS as to cooking metho	Lo	Meats
22101100	Pork chop, broiled or baked, NS as	Lo	Meats
22101110	Pork chop, broiled or baked, lean a	Lo	Meats
22101120	Pork chop, broiled or baked, lean o	Lo	Meats
22101130	Pork chop, breaded or floured, broi	Lo	Meats
22101140	Pork chop, breaded or floured, broi	Lo	Meats
22101150	Pork chop, breaded or floured, broi	Lo	Meats
22101200	Pork chop, fried, NS as to fat eaten	Lo	Meats
22101210	Pork chop, fried, lean and fat eaten	Lo	Meats
22101220	Pork chop, fried, lean only eaten	Lo	Meats
22101300	Pork chop, breaded or floured, fried	Lo	Meats
22101310	Pork chop, breaded or floured, fried	Lo	Meats
22101320	Pork chop, breaded or floured, fried	Lo	Meats
22101400	Pork chop, battered, fried, NS as to	Lo	Meats
22101410	Pork chop, battered, fried, lean and	Lo	Meats
22101420	Pork chop, battered, fried, lean only	Lo	Meats
22101500	Pork chop, stewed, NS as to fat eat	Lo	Meats
22101510	Pork chop, stewed, lean and fat eat	Lo	Meats
22101520	Pork chop, stewed, lean only eaten	Lo	Meats
22107000	Pork chop, smoked or cured, cooke	Lo	Cured Meats/Poultry
22107010	Pork chop, smoked or cured, cooke	Lo	Cured Meats/Poultry
22107020	Pork chop, smoked or cured, cooke	Lo	Cured Meats/Poultry
22201000	Pork steak or cutlet, NS as to cooki	Lo	Meats
22201010	Pork steak or cutlet, NS as to cooki	Lo	Meats
22201020	Pork steak or cutlet, NS as to cooki	Lo	Meats
22201050	Pork steak or cutlet, battered, fried,	Lo	Meats
22201060	Pork steak or cutlet, battered, fried,	Lo	Meats
22201070	Pork steak or cutlet, battered, fried,	Lo	Meats
22201100	Pork steak or cutlet, broiled or bake	Lo	Meats
22201110	Pork steak or cutlet, broiled or bake	Lo	Meats
22201120	Pork steak or cutlet, broiled or bake	Lo	Meats
22201200	Pork steak or cutlet, fried, NS as to	Lo	Meats
22201210	Pork steak or cutlet, fried, lean and	Lo	Meats
22201220	Pork steak or cutlet, fried, lean only	Lo	Meats

22201300	Pork steak or cutlet, breaded or flou	Lo	Meats
22201310	Pork steak or cutlet, breaded or flou	Lo	Meats
22201320	Pork steak or cutlet, breaded or flou	Lo	Meats
22201400	Pork steak or cutlet, breaded or flou	Lo	Meats
22201410	Pork steak or cutlet, breaded or flou	Lo	Meats
22201420	Pork steak or cutlet, breaded or flou	Lo	Meats
22210300	Pork, tenderloin, cooked, NS as to c	Lo	Meats
22210310	Pork, tenderloin, breaded, fried	Lo	Meats
22210350	Pork, tenderloin, braised	Lo	Meats
22210400	Pork, tenderloin, baked	Lo	Meats
22210450	Pork, tenderloin, battered, fried	Lo	Meats
22300120	Ham, fried, NS as to fat eaten	Lo	Cured Meats/Poultry
22300130	Ham, fried, lean and fat eaten	Lo	Cured Meats/Poultry
22300140	Ham, fried, lean only eaten	Lo	Cured Meats/Poultry
22300150	Ham, breaded or floured, fried, NS	Lo	Cured Meats/Poultry
22300160	Ham, breaded or floured, fried, lean	Lo	Cured Meats/Poultry
22300170	Ham, breaded or floured, fried, lean	Lo	Cured Meats/Poultry
22301000	Ham, fresh, cooked, NS as to fat ea	Lo	Meats
22301110	Ham, fresh, cooked, lean and fat ea	Lo	Meats
22301120	Ham, fresh, cooked, lean only eaten	Lo	Meats
22311000	Ham, smoked or cured, cooked, NS	Lo	Cured Meats/Poultry
22311010	Ham, smoked or cured, cooked, lea	Lo	Cured Meats/Poultry
22311020	Ham, smoked or cured, cooked, lea	Lo	Cured Meats/Poultry
22311220	Ham, smoked or cured, low sodium	Lo	Cured Meats/Poultry
22311450	Ham, prosciutto	Lo	Cured Meats/Poultry
22311500	Ham, smoked or cured, canned, NS	Lo	Cured Meats/Poultry
22311510	Ham, smoked or cured, canned, lea	Lo	Cured Meats/Poultry
22311520	Ham, smoked or cured, canned, lea	Lo	Cured Meats/Poultry
22321110	Ham, smoked or cured, ground patt	Lo	Cured Meats/Poultry
22400100	Pork roast, NS as to cut, cooked, N	Lo	Meats
22400110	Pork roast, NS as to cut, cooked, le	Lo	Meats
22400120	Pork roast, NS as to cut, cooked, le	Lo	Meats
22401000	Pork roast, loin, cooked, NS as to fa	Lo	Meats
22401010	Pork roast, loin, cooked, lean and fa	Lo	Meats
22401020	Pork roast, loin, cooked, lean only e	Lo	Meats
22411000	Pork roast, shoulder, cooked, NS as	Lo	Meats
22411010	Pork roast, shoulder, cooked, lean a	Lo	Meats
22411020	Pork roast, shoulder, cooked, lean o	Lo	Meats
22421000	Pork roast, smoked or cured, cooke	Lo	Cured Meats/Poultry
22421010	Pork roast, smoked or cured, cooke	Lo	Cured Meats/Poultry
22421020	Pork roast, smoked or cured, cooke	Lo	Cured Meats/Poultry
22431000	Pork roll, cured, fried	Lo	Cured Meats/Poultry
22501010	Canadian bacon, cooked	Lo	Cured Meats/Poultry
22600100	Bacon, NS as to type of meat, cook	Lo	Cured Meats/Poultry
22600200	Pork bacon, NS as to fresh, smoked	Lo	Cured Meats/Poultry
22600210	Pork bacon, NS as to fresh, smoked	Lo	Cured Meats/Poultry
22601000	Pork bacon, smoked or cured, cook	Lo	Cured Meats/Poultry
22601020	Pork bacon, smoked or cured, cook	Lo	Cured Meats/Poultry
22601040	Bacon or side pork, fresh, cooked	Lo	Cured Meats/Poultry
22602010	Pork bacon, smoked or cured, redu	Lo	Cured Meats/Poultry
22605010	Pork bacon, formed, lean meat add	Lo	Cured Meats/Poultry

22621000	Salt pork, cooked	Lo	Cured Meats/Poultry
22621100	Fat back, cooked	Lo	Fats and Oils
22701000	Pork, spareribs, cooked, NS as to fa	Lo	Meats
22701010	Pork, spareribs, cooked, lean and fa	Lo	Meats
22701020	Pork, spareribs, cooked, lean only e	Lo	Meats
22701030	Pork, spareribs, barbecued, with sa	Lo	Meats
22701040	Pork, spareribs, barbecued, with sa	Lo	Meats
22701050	Pork, spareribs, barbecued, with sa	Lo	Meats
22704010	Pork, cracklings, cooked	Lo	Meats
22705010	Pork ears, tail, head, snout, miscell	Lo	Meats
22706010	Pork, neck bones, cooked	Lo	Meats
22707010	Pork, pig's feet, cooked	Lo	Meats
22707020	Pork, pig's feet, pickled	Lo	Meats
22708010	Pork, pig's hocks, cooked	Lo	Meats
22709010	Pork skin rinds	Lo	Meats
22709110	Pork skin, boiled	Lo	Meats
22810010	Ham, baby food, strained	Lo	Baby Foods
22820000	Meat stick, baby food	Lo	Baby Foods
23000100	Lamb, NS as to cut, cooked	Lo	Meats
23101000	Lamb chop, NS as to cut, cooked, N	Lo	Meats
23101010	Lamb chop, NS as to cut, cooked, l	Lo	Meats
23101020	Lamb chop, NS as to cut, cooked, l	Lo	Meats
23104000	Lamb, loin chop, cooked, NS as to	Lo	Meats
23104010	Lamb, loin chop, cooked, lean and	Lo	Meats
23104020	Lamb, loin chop, cooked, lean only	Lo	Meats
23107020	Lamb, shoulder chop, cooked, lean	Lo	Meats
23108020	Lamb, shoulder, cooked, lean only	Lo	Meats
23110000	Lamb, ribs, cooked, lean only eaten	Lo	Meats
23110050	Lamb, ribs, cooked, lean and fat ea	Lo	Meats
23111010	Lamb hocks, cooked	Lo	Meats
23120100	Lamb, roast, cooked, NS as to fat e	Lo	Meats
23120110	Lamb, roast, cooked, lean and fat e	Lo	Meats
23120120	Lamb, roast, cooked, lean only eate	Lo	Meats
23132000	Lamb, ground or patty, cooked	Lo	Meats
23150100	Goat, boiled	Lo	Meats
23150200	Goat, fried	Lo	Meats
23150250	Goat, baked	Lo	Meats
23150270	Goat head, cooked	Lo	Meats
23150300	Goat ribs, cooked	Lo	Meats
23200100	Veal, NS as to cut, cooked, NS as t	Lo	Meats
23200110	Veal, NS as to cut, cooked, lean an	Lo	Meats
23200120	Veal, NS as to cut, cooked, lean on	Lo	Meats
23201030	Veal chop, NS as to cooking metho	Lo	Meats
23203020	Veal chop, fried, lean and fat eaten	Lo	Meats
23203030	Veal chop, fried, lean only eaten	Lo	Meats
23203110	Veal chop, broiled, lean and fat eate	Lo	Meats
23203120	Veal chop, broiled, lean only eaten	Lo	Meats
23204010	Veal cutlet or steak, NS as to cooki	Lo	Meats
23204030	Veal cutlet or steak, NS as to cooki	Lo	Meats
23204200	Veal cutlet or steak, broiled, NS as	Lo	Meats
23204210	Veal cutlet or steak, broiled, lean ar	Lo	Meats

23204220	Veal cutlet or steak, broiled, lean or	Lo	Meats
23205010	Veal cutlet or steak, fried, NS as to	Lo	Meats
23205020	Veal cutlet or steak, fried, lean and	Lo	Meats
23205030	Veal cutlet or steak, fried, lean only	Lo	Meats
23210030	Veal, roasted, lean only eaten	Lo	Meats
23220010	Veal, ground or patty, cooked	Lo	Meats
23220020	Mock chicken legs, cooked	Lo	Meats
23220030	Veal patty, breaded, cooked	Lo	Meats
23310000	Rabbit, NS as to domestic or wild, c	Lo	Meats
23311120	Rabbit, NS as to domestic or wild, b	Lo	Meats
23321000	Venison/deer, NFS	Lo	Meats
23321100	Venison/deer, roasted	Lo	Meats
23321200	Venison/deer steak, cooked, NS as	Lo	Meats
23321250	Venison/deer steak, breaded or flou	Lo	Meats
23321900	Venison/deer jerky	Lo	Cured Meats/Poultry
23322100	Deer sausage	Lo	Cured Meats/Poultry
23322300	Deer chop, cooked	Lo	Meats
23322350	Venison/deer ribs, cooked	Lo	Meats
23322400	Venison/deer, stewed	Lo	Meats
23323100	Moose, cooked	Lo	Meats
23323500	Bear, cooked	Lo	Meats
23326100	Bison, cooked	Lo	Meats
23333100	Squirrel, cooked	Lo	Meats
23334100	Beaver, cooked	Lo	Meats
23335100	Raccoon, cooked	Lo	Meats
23345100	Wild pig, smoked	Lo	Meats
23420010	Veal, baby food, strained	Lo	Baby Foods
24100000	Chicken, NS as to part and cooking	Lo	Poultry
24100010	Chicken, NS as to part and cooking	Lo	Poultry
24100020	Chicken, NS as to part and cooking	Lo	Poultry
24102000	Chicken, NS as to part, baked, broi	Lo	Poultry
24102010	Chicken, NS as to part, baked, broi	Lo	Poultry
24102020	Chicken, NS as to part, baked, broi	Lo	Poultry
24102050	Chicken, NS as to part, rotisserie, N	Lo	Poultry
24102060	Chicken, NS as to part, rotisserie, s	Lo	Poultry
24102070	Chicken, NS as to part, rotisserie, s	Lo	Poultry
24103000	Chicken, NS as to part, stewed, NS	Lo	Poultry
24103010	Chicken, NS as to part, stewed, ski	Lo	Poultry
24103020	Chicken, NS as to part, stewed, ski	Lo	Poultry
24103050	Chicken, NS as to part, grilled witho	Lo	Poultry
24103060	Chicken, NS as to part, grilled witho	Lo	Poultry
24103070	Chicken, NS as to part, grilled with s	Lo	Poultry
24103075	Chicken, NS as to part, grilled with s	Lo	Poultry
24103080	Chicken, NS as to part, grilled with s	Lo	Poultry
24104000	Chicken, NS as to part, fried, no coa	Lo	Poultry
24104010	Chicken, NS as to part, fried, no coa	Lo	Poultry
24104020	Chicken, NS as to part, fried, no coa	Lo	Poultry
24104051	Chicken, NS as to part, sauteed, sk	Lo	Poultry
24107000	Chicken, NS as to part, coated, bak	Lo	Poultry
24107001	Chicken, NS as to part, coated, bak	Lo	Poultry
24107010	Chicken, NS as to part, coated, bak	Lo	Poultry

24107020	Chicken, NS as to part, coated, bak	Lo	Poultry
24107040	Chicken, NS as to part, coated, bak	Lo	Poultry
24107050	Chicken, NS as to part, coated, bak	Lo	Poultry
24107060	Chicken, NS as to part, coated, bak	Lo	Poultry
24107070	Chicken, NS as to part, fried, coate	Lo	Poultry
24107071	Chicken, NS as to part, fried, coate	Lo	Poultry
24107080	Chicken, NS as to part, baked, coat	Lo	Poultry
24120100	Chicken, breast, NS as to cooking r	Lo	Poultry
24120110	Chicken breast, NS as to cooking m	Lo	Poultry
24120120	Chicken breast, NS as to cooking m	Lo	Poultry
24122100	Chicken, breast, roasted, broiled, or	Lo	Poultry
24122110	Chicken, breast, roasted, broiled, or	Lo	Poultry
24122120	Chicken, breast, roasted, broiled, or	Lo	Poultry
24122130	Chicken breast, baked, broiled, or r	Lo	Poultry
24122131	Chicken breast, baked, broiled, or r	Lo	Poultry
24122140	Chicken breast, baked or broiled, sl	Lo	Poultry
24122141	Chicken breast, baked or broiled, sl	Lo	Poultry
24122150	Chicken breast, baked or broiled, sl	Lo	Poultry
24122151	Chicken breast, baked or broiled, sl	Lo	Poultry
24122160	Chicken breast, baked, broiled, or r	Lo	Poultry
24122161	Chicken breast, baked, broiled, or r	Lo	Poultry
24122170	Chicken breast, rotisserie, skin eate	Lo	Poultry
24122171	Chicken breast, rotisserie, skin not	Lo	Poultry
24123100	Chicken, breast, stewed, NS as to s	Lo	Poultry
24123110	Chicken breast, stewed, skin eaten	Lo	Poultry
24123120	Chicken breast, stewed, skin not ea	Lo	Poultry
24123300	Chicken breast, grilled without sauc	Lo	Poultry
24123301	Chicken breast, grilled without sauc	Lo	Poultry
24123310	Chicken breast, grilled with sauce, s	Lo	Poultry
24123311	Chicken breast, grilled with sauce, s	Lo	Poultry
24124100	Chicken, breast, fried, no coating, N	Lo	Poultry
24124110	Chicken, breast, fried, no coating, s	Lo	Poultry
24124113	Chicken, breast, fried, no coating, s	Lo	Poultry
24124115	Chicken, breast, fried, no coating, s	Lo	Poultry
24124120	Chicken, breast, fried, no coating, s	Lo	Poultry
24124121	Chicken, breast, fried, no coating, s	Lo	Poultry
24124122	Chicken, breast, fried, no coating, s	Lo	Poultry
24124123	Chicken, breast, fried, no coating, s	Lo	Poultry
24124124	Chicken, breast, fried, no coating, s	Lo	Poultry
24124125	Chicken, breast, fried, no coating, s	Lo	Poultry
24124200	Chicken breast, sauteed, skin eaten	Lo	Poultry
24124201	Chicken breast, sauteed, skin not e	Lo	Poultry
24127100	Chicken, breast, coated, baked or f	Lo	Poultry
24127110	Chicken, breast, coated, baked or f	Lo	Poultry
24127112	Chicken, breast, coated, baked or f	Lo	Poultry
24127113	Chicken, breast, coated, baked or f	Lo	Poultry
24127115	Chicken, breast, coated, baked or f	Lo	Poultry
24127120	Chicken, breast, coated, baked or f	Lo	Poultry
24127125	Chicken, breast, from fast food, coa	Lo	Poultry
24127130	Chicken, breast, from fast food, coa	Lo	Poultry
24127135	Chicken, breast, from fast food, coa	Lo	Poultry

24127140	Chicken, breast, coated, baked or f	Lo	Poultry
24127141	Chicken, breast, coated, baked or f	Lo	Poultry
24127150	Chicken, breast, coated, baked or f	Lo	Poultry
24127151	Chicken, breast, coated, baked or f	Lo	Poultry
24127152	Chicken, breast, coated, baked or f	Lo	Poultry
24127153	Chicken, breast, coated, baked or f	Lo	Poultry
24127154	Chicken, breast, coated, baked or f	Lo	Poultry
24127155	Chicken, breast, coated, baked or f	Lo	Poultry
24127160	Chicken, breast, coated, baked or f	Lo	Poultry
24127163	Chicken, breast, coated, baked or f	Lo	Poultry
24127165	Chicken, breast, coated, baked or f	Lo	Poultry
24127200	Chicken breast, fried, coated, skin /	Lo	Poultry
24127201	Chicken breast, fried, coated, skin /	Lo	Poultry
24127202	Chicken breast, fried, coated, prepa	Lo	Poultry
24127210	Chicken breast, fried, coated, skin /	Lo	Poultry
24127211	Chicken breast, fried, coated, skin /	Lo	Poultry
24127220	Chicken breast, fried, coated, skin /	Lo	Poultry
24127221	Chicken breast, fried, coated, skin /	Lo	Poultry
24127500	Chicken breast, baked, coated, skir	Lo	Poultry
24127501	Chicken breast, baked, coated, skir	Lo	Poultry
24130200	Chicken, leg (drumstick and thigh),	Lo	Poultry
24130210	Chicken leg, drumstick and thigh, N	Lo	Poultry
24130220	Chicken leg, drumstick and thigh, N	Lo	Poultry
24132200	Chicken, leg (drumstick and thigh),	Lo	Poultry
24132210	Chicken, leg (drumstick and thigh),	Lo	Poultry
24132220	Chicken, leg (drumstick and thigh),	Lo	Poultry
24132230	Chicken leg, drumstick and thigh, b	Lo	Poultry
24132231	Chicken leg, drumstick and thigh, b	Lo	Poultry
24132240	Chicken leg, drumstick and thigh, ro	Lo	Poultry
24132241	Chicken leg, drumstick and thigh, ro	Lo	Poultry
24133200	Chicken, leg (drumstick and thigh),	Lo	Poultry
24133210	Chicken leg, drumstick and thigh, s	Lo	Poultry
24133220	Chicken leg, drumstick and thigh, s	Lo	Poultry
24134100	Chicken leg, drumstick and thigh, g	Lo	Poultry
24134101	Chicken leg, drumstick and thigh, g	Lo	Poultry
24134150	Chicken leg, drumstick and thigh, g	Lo	Poultry
24134151	Chicken leg, drumstick and thigh, g	Lo	Poultry
24134200	Chicken, leg (drumstick and thigh),	Lo	Poultry
24134210	Chicken, leg (drumstick and thigh),	Lo	Poultry
24134220	Chicken, leg (drumstick and thigh),	Lo	Poultry
24134300	Chicken leg, drumstick and thigh, s	Lo	Poultry
24134301	Chicken leg, drumstick and thigh, s	Lo	Poultry
24137200	Chicken, leg (drumstick and thigh),	Lo	Poultry
24137210	Chicken, leg (drumstick and thigh),	Lo	Poultry
24137211	Chicken, leg (drumstick and thigh),	Lo	Poultry
24137220	Chicken, leg (drumstick and thigh),	Lo	Poultry
24137240	Chicken, leg (drumstick and thigh),	Lo	Poultry
24137250	Chicken, leg (drumstick and thigh),	Lo	Poultry
24137251	Chicken, leg (drumstick and thigh),	Lo	Poultry
24137260	Chicken, leg (drumstick and thigh),	Lo	Poultry
24137300	Chicken leg, drumstick and thigh, fr	Lo	Poultry

24137301	Chicken leg, drumstick and thigh, fr	Lo	Poultry
24137310	Chicken leg, drumstick and thigh, b	Lo	Poultry
24137311	Chicken leg, drumstick and thigh, b	Lo	Poultry
24140200	Chicken, drumstick, NS as to cooki	Lo	Poultry
24140210	Chicken drumstick, NS as to cookin	Lo	Poultry
24140220	Chicken drumstick, NS as to cookin	Lo	Poultry
24142200	Chicken, drumstick, roasted, broiled	Lo	Poultry
24142210	Chicken, drumstick, roasted, broiled	Lo	Poultry
24142220	Chicken, drumstick, roasted, broiled	Lo	Poultry
24142300	Chicken drumstick, baked, broiled,	Lo	Poultry
24142301	Chicken drumstick, baked, broiled,	Lo	Poultry
24142310	Chicken drumstick, baked or broiled	Lo	Poultry
24142311	Chicken drumstick, baked or broiled	Lo	Poultry
24142320	Chicken drumstick, baked or broiled	Lo	Poultry
24142321	Chicken drumstick, baked or broiled	Lo	Poultry
24142400	Chicken drumstick, rotisserie, skin e	Lo	Poultry
24142401	Chicken drumstick, rotisserie, skin n	Lo	Poultry
24142500	Chicken drumstick, grilled without s	Lo	Poultry
24142501	Chicken drumstick, grilled without s	Lo	Poultry
24142510	Chicken drumstick, grilled with sauc	Lo	Poultry
24142511	Chicken drumstick, grilled with sauc	Lo	Poultry
24143200	Chicken, drumstick, stewed, NS as	Lo	Poultry
24143210	Chicken drumstick, stewed, skin ea	Lo	Poultry
24143220	Chicken drumstick, stewed, skin no	Lo	Poultry
24144200	Chicken, drumstick, fried, no coatin	Lo	Poultry
24144210	Chicken, drumstick, fried, no coatin	Lo	Poultry
24144212	Chicken, drumstick, fried, no coatin	Lo	Poultry
24144213	Chicken, drumstick, fried, no coatin	Lo	Poultry
24144215	Chicken, drumstick, fried, no coatin	Lo	Poultry
24144220	Chicken, drumstick, fried, no coatin	Lo	Poultry
24144221	Chicken, drumstick, fried, no coatin	Lo	Poultry
24144222	Chicken, drumstick, fried, no coatin	Lo	Poultry
24144223	Chicken, drumstick, fried, no coatin	Lo	Poultry
24144225	Chicken, drumstick, fried, no coatin	Lo	Poultry
24144300	Chicken drumstick, sauteed, skin ea	Lo	Poultry
24144301	Chicken drumstick, sauteed, skin no	Lo	Poultry
24147200	Chicken, drumstick, coated, baked	Lo	Poultry
24147210	Chicken, drumstick, coated, baked	Lo	Poultry
24147212	Chicken, drumstick, coated, baked	Lo	Poultry
24147213	Chicken, drumstick, coated, baked	Lo	Poultry
24147215	Chicken, drumstick, coated, baked	Lo	Poultry
24147220	Chicken, drumstick, coated, baked	Lo	Poultry
24147223	Chicken, drumstick, coated, baked	Lo	Poultry
24147225	Chicken, drumstick, from fast food,	Lo	Poultry
24147230	Chicken, drumstick, from fast food,	Lo	Poultry
24147235	Chicken, drumstick, from fast food,	Lo	Poultry
24147240	Chicken, drumstick, coated, baked	Lo	Poultry
24147250	Chicken, drumstick, coated, baked	Lo	Poultry
24147251	Chicken, drumstick, coated, baked	Lo	Poultry
24147252	Chicken, drumstick, coated, baked	Lo	Poultry
24147253	Chicken, drumstick, coated, baked	Lo	Poultry

24147255	Chicken, drumstick, coated, baked	Lo	Poultry
24147260	Chicken, drumstick, coated, baked	Lo	Poultry
24147263	Chicken, drumstick, coated, baked	Lo	Poultry
24147265	Chicken, drumstick, coated, baked	Lo	Poultry
24147300	Chicken drumstick, fried, coated, sk	Lo	Poultry
24147301	Chicken drumstick, fried, coated, sk	Lo	Poultry
24147302	Chicken drumstick, fried, coated, pr	Lo	Poultry
24147310	Chicken drumstick, fried, coated, sk	Lo	Poultry
24147311	Chicken drumstick, fried, coated, sk	Lo	Poultry
24147320	Chicken drumstick, fried, coated, sk	Lo	Poultry
24147321	Chicken drumstick, fried, coated, sk	Lo	Poultry
24147400	Chicken drumstick, baked, coated,	Lo	Poultry
24147401	Chicken drumstick, baked, coated,	Lo	Poultry
24150200	Chicken, thigh, NS as to cooking m	Lo	Poultry
24150210	Chicken thigh, NS as to cooking me	Lo	Poultry
24150220	Chicken thigh, NS as to cooking me	Lo	Poultry
24152200	Chicken, thigh, roasted, broiled, or	Lo	Poultry
24152210	Chicken, thigh, roasted, broiled, or	Lo	Poultry
24152220	Chicken, thigh, roasted, broiled, or	Lo	Poultry
24152230	Chicken thigh, baked, broiled, or ro	Lo	Poultry
24152231	Chicken thigh, baked, broiled, or ro	Lo	Poultry
24152240	Chicken thigh, baked or broiled, ski	Lo	Poultry
24152241	Chicken thigh, baked or broiled, ski	Lo	Poultry
24152250	Chicken thigh, baked or broiled, ski	Lo	Poultry
24152251	Chicken thigh, baked or broiled, ski	Lo	Poultry
24152300	Chicken thigh, rotisserie, skin eaten	Lo	Poultry
24152301	Chicken thigh, rotisserie, skin not e	Lo	Poultry
24153200	Chicken, thigh, stewed, NS as to sk	Lo	Poultry
24153210	Chicken thigh, stewed, skin eaten	Lo	Poultry
24153220	Chicken thigh, stewed, skin not eat	Lo	Poultry
24154010	Chicken thigh, grilled without sauce	Lo	Poultry
24154011	Chicken thigh, grilled without sauce	Lo	Poultry
24154020	Chicken thigh, grilled with sauce, sk	Lo	Poultry
24154021	Chicken thigh, grilled with sauce, sk	Lo	Poultry
24154200	Chicken, thigh, fried, no coating, NS	Lo	Poultry
24154210	Chicken, thigh, fried, no coating, sk	Lo	Poultry
24154211	Chicken, thigh, fried, no coating, sk	Lo	Poultry
24154212	Chicken, thigh, fried, no coating, sk	Lo	Poultry
24154213	Chicken, thigh, fried, no coating, sk	Lo	Poultry
24154214	Chicken, thigh, fried, no coating, sk	Lo	Poultry
24154215	Chicken, thigh, fried, no coating, sk	Lo	Poultry
24154220	Chicken, thigh, fried, no coating, sk	Lo	Poultry
24154221	Chicken, thigh, fried, no coating, sk	Lo	Poultry
24154222	Chicken, thigh, fried, no coating, sk	Lo	Poultry
24154223	Chicken, thigh, fried, no coating, sk	Lo	Poultry
24154224	Chicken, thigh, fried, no coating, sk	Lo	Poultry
24154225	Chicken, thigh, fried, no coating, sk	Lo	Poultry
24154300	Chicken thigh, sauteed, skin eaten	Lo	Poultry
24154301	Chicken thigh, sauteed, skin not ea	Lo	Poultry
24157200	Chicken, thigh, coated, baked or fri	Lo	Poultry
24157210	Chicken, thigh, coated, baked or fri	Lo	Poultry

24157213	Chicken, thigh, coated, baked or fri	Lo	Poultry
24157215	Chicken, thigh, coated, baked or fri	Lo	Poultry
24157220	Chicken, thigh, coated, baked or fri	Lo	Poultry
24157223	Chicken, thigh, coated, baked or fri	Lo	Poultry
24157225	Chicken, thigh, from fast food, coat	Lo	Poultry
24157230	Chicken, thigh, from fast food, coat	Lo	Poultry
24157235	Chicken, thigh, from fast food, coat	Lo	Poultry
24157240	Chicken, thigh, coated, baked or fri	Lo	Poultry
24157250	Chicken, thigh, coated, baked or fri	Lo	Poultry
24157253	Chicken, thigh, coated, baked or fri	Lo	Poultry
24157255	Chicken, thigh, coated, baked or fri	Lo	Poultry
24157260	Chicken, thigh, coated, baked or fri	Lo	Poultry
24157263	Chicken, thigh, coated, baked or fri	Lo	Poultry
24157300	Chicken thigh, fried, coated, skin / c	Lo	Poultry
24157301	Chicken thigh, fried, coated, skin / c	Lo	Poultry
24157302	Chicken thigh, fried, coated, prepar	Lo	Poultry
24157310	Chicken thigh, fried, coated, skin / c	Lo	Poultry
24157311	Chicken thigh, fried, coated, skin / c	Lo	Poultry
24157320	Chicken thigh, fried, coated, skin / c	Lo	Poultry
24157321	Chicken thigh, fried, coated, skin / c	Lo	Poultry
24157330	Chicken thigh, fried, coated, skin / c	Lo	Poultry
24157331	Chicken thigh, fried, coated, skin / c	Lo	Poultry
24157400	Chicken thigh, baked, coated, skin /	Lo	Poultry
24157401	Chicken thigh, baked, coated, skin /	Lo	Poultry
24160100	Chicken, wing, NS as to cooking me	Lo	Poultry
24160110	Chicken wing, NS as to cooking me	Lo	Poultry
24160120	Chicken, wing, NS as to cooking me	Lo	Poultry
24162100	Chicken, wing, roasted, broiled, or b	Lo	Poultry
24162110	Chicken, wing, roasted, broiled, or b	Lo	Poultry
24162120	Chicken, wing, roasted, broiled, or b	Lo	Poultry
24162130	Chicken wing, baked, broiled, or roa	Lo	Poultry
24162140	Chicken wing, baked or broiled, fron	Lo	Poultry
24162150	Chicken wing, baked or broiled, fron	Lo	Poultry
24162200	Chicken wing, rotisserie	Lo	Poultry
24163100	Chicken, wing, stewed, NS as to sk	Lo	Poultry
24163110	Chicken wing, stewed	Lo	Poultry
24163120	Chicken, wing, stewed, skin not eat	Lo	Poultry
24164000	Chicken wing, grilled without sauce	Lo	Poultry
24164010	Chicken wing, grilled with sauce	Lo	Poultry
24164100	Chicken, wing, fried, no coating, NS	Lo	Poultry
24164110	Chicken, wing, fried, no coating, ski	Lo	Poultry
24164111	Chicken, wing, fried, no coating, ski	Lo	Poultry
24164112	Chicken, wing, fried, no coating, ski	Lo	Poultry
24164113	Chicken, wing, fried, no coating, ski	Lo	Poultry
24164115	Chicken, wing, fried, no coating, ski	Lo	Poultry
24164120	Chicken, wing, fried, no coating, ski	Lo	Poultry
24164122	Chicken, wing, fried, no coating, ski	Lo	Poultry
24164123	Chicken, wing, fried, no coating, ski	Lo	Poultry
24164125	Chicken, wing, fried, no coating, ski	Lo	Poultry
24164200	Chicken wing, sauteed	Lo	Poultry
24167100	Chicken, wing, coated, baked or frie	Lo	Poultry

24167110	Chicken, wing, coated, baked or frie	Lo	Poultry
24167113	Chicken, wing, coated, baked or frie	Lo	Poultry
24167115	Chicken, wing, coated, baked or frie	Lo	Poultry
24167119	Chicken, wing, coated, baked or frie	Lo	Poultry
24167120	Chicken, wing, coated, baked or frie	Lo	Poultry
24167123	Chicken, wing, coated, baked or frie	Lo	Poultry
24167125	Chicken, wing, from fast food, coate	Lo	Poultry
24167130	Chicken, wing, from fast food, coate	Lo	Poultry
24167135	Chicken, wing, from fast food, coate	Lo	Poultry
24167200	Chicken wing, fried, coated, from ra	Lo	Poultry
24167210	Chicken wing, fried, coated, from pr	Lo	Poultry
24167220	Chicken wing, fried, coated, from fa	Lo	Poultry
24167230	Chicken wing, fried, coated, from re	Lo	Poultry
24167300	Chicken wing, baked, coated	Lo	Poultry
24168000	Chicken "wings" with hot sauce, fro	Lo	Poultry
24168001	Chicken "wings" with other sauces	Lo	Poultry
24168002	Chicken "wings", plain, from fast foo	Lo	Poultry
24168010	Chicken "wings" with hot sauce, fro	Lo	Poultry
24168011	Chicken "wings" with other sauces	Lo	Poultry
24168012	Chicken "wings", plain, from precoo	Lo	Poultry
24168020	Chicken "wings" with hot sauce, fro	Lo	Poultry
24168021	Chicken "wings" with other sauces	Lo	Poultry
24168022	Chicken "wings", plain, from other s	Lo	Poultry
24168030	Chicken "wings", boneless, with hot	Lo	Poultry
24168031	Chicken "wings", boneless, with hot	Lo	Poultry
24170200	Chicken, back	Lo	Poultry
24180200	Chicken, neck or ribs	Lo	Poultry
24198440	Chicken skin	Lo	Poultry
24198500	Chicken feet	Lo	Poultry
24198570	Chicken, canned, meat only	Lo	Poultry
24198670	Chicken, chicken roll, roasted	Lo	Poultry
24198671	Chicken patty, breaded	Lo	Poultry
24198677	Chicken fillet, breaded	Lo	Poultry
24198683	Chicken fillet, grilled	Lo	Poultry
24198690	Chicken patty, fillet, or tenders, brea	Lo	Poultry
24198695	Chicken patty, fillet, or tenders, brea	Lo	Poultry
24198700	Chicken patty, fillet, or tenders, brea	Lo	Poultry
24198710	Chicken patty with cheese, breaded	Lo	Poultry
24198720	Chicken, ground	Lo	Poultry
24198729	Chicken nuggets, NFS	Lo	Poultry
24198730	Chicken nuggets, from fast food / re	Lo	Poultry
24198731	Chicken nuggets, from fast food	Lo	Poultry
24198732	Chicken nuggets, from restaurant	Lo	Poultry
24198735	Chicken nuggets, from school lunch	Lo	Poultry
24198736	Chicken nuggets, from frozen	Lo	Poultry
24198737	Chicken nuggets, from other source	Lo	Poultry
24198739	Chicken tenders or strips, NFS	Lo	Poultry
24198740	Chicken nuggets	Lo	Poultry
24198741	Chicken tenders or strips, breaded,	Lo	Poultry
24198742	Chicken tenders or strips, breaded,	Lo	Poultry
24198745	Chicken tenders or strips, breaded,	Lo	Poultry

24198746	Chicken tenders or strips, breaded,	Lo	Poultry
24198747	Chicken tenders or strips, breaded,	Lo	Poultry
24198840	Fried chicken chunks, Puerto Rican	Lo	Poultry
24201000	Turkey, NFS	Lo	Poultry
24201010	Turkey, light meat, cooked, NS as t	Lo	Poultry
24201020	Turkey, light meat, skin not eaten	Lo	Poultry
24201030	Turkey, light meat, skin eaten	Lo	Poultry
24201050	Turkey, light meat, breaded, baked	Lo	Poultry
24201060	Turkey, light meat, breaded, baked	Lo	Poultry
24201070	Turkey, light meat, breaded, baked	Lo	Poultry
24201110	Turkey, light meat, roasted, NS as t	Lo	Poultry
24201120	Turkey, light meat, roasted, skin no	Lo	Poultry
24201130	Turkey, light meat, roasted, skin ea	Lo	Poultry
24201210	Turkey, dark meat, roasted, NS as	Lo	Poultry
24201220	Turkey, dark meat, roasted, skin no	Lo	Poultry
24201230	Turkey, dark meat, roasted, skin ea	Lo	Poultry
24201310	Turkey, light and dark meat, roaste	Lo	Poultry
24201320	Turkey, light and dark meat, roaste	Lo	Poultry
24201330	Turkey, light and dark meat, roaste	Lo	Poultry
24201350	Turkey, light or dark meat, battered	Lo	Poultry
24201360	Turkey, light or dark meat, fried, co	Lo	Poultry
24201370	Turkey, light or dark meat, fried, co	Lo	Poultry
24201400	Turkey, light or dark meat, stewed,	Lo	Poultry
24201410	Turkey, light or dark meat, stewed,	Lo	Poultry
24201420	Turkey light or dark meat, stewed, s	Lo	Poultry
24201500	Turkey, light or dark meat, smoked,	Lo	Poultry
24201510	Turkey, light or dark meat, smoked,	Lo	Poultry
24201520	Turkey, light or dark meat, smoked,	Lo	Poultry
24202000	Turkey, drumstick, cooked, NS as t	Lo	Poultry
24202010	Turkey, drumstick, cooked, skin not	Lo	Poultry
24202020	Turkey, drumstick, cooked, skin eat	Lo	Poultry
24202050	Turkey, drumstick, roasted, NS as t	Lo	Poultry
24202060	Turkey, drumstick, roasted, skin no	Lo	Poultry
24202070	Turkey, drumstick, roasted, skin ea	Lo	Poultry
24202120	Turkey, drumstick, smoked, skin ea	Lo	Poultry
24202450	Turkey, thigh, cooked, NS as to ski	Lo	Poultry
24202460	Turkey, thigh, cooked, skin eaten	Lo	Poultry
24202500	Turkey, thigh, cooked, skin not eate	Lo	Poultry
24202600	Turkey, neck	Lo	Poultry
24203000	Turkey, wing, cooked, NS as to skir	Lo	Poultry
24203010	Turkey, wing, cooked, skin not eate	Lo	Poultry
24203020	Turkey, wing, cooked, skin eaten	Lo	Poultry
24203120	Turkey, wing, smoked, skin eaten	Lo	Poultry
24204000	Turkey, rolled roast, light or dark m	Lo	Poultry
24205000	Turkey, tail	Lo	Poultry
24207000	Turkey, ground	Lo	Poultry
24208000	Turkey, nuggets	Lo	Poultry
24208500	Turkey bacon, cooked	Lo	Cured Meats/Poultry
24208510	Turkey bacon, reduced sodium, coo	Lo	Cured Meats/Poultry
24209000	Turkey with barbecue sauce, skin e	Lo	Mixed Dishes - Meat, Poultry, Seafood
24209001	Turkey with barbecue sauce, skin n	Lo	Mixed Dishes - Meat, Poultry, Seafood

24300100	Duck, cooked, NS as to skin eaten	Lo	Poultry
24300110	Duck, cooked, skin eaten	Lo	Poultry
24300120	Duck, cooked, skin not eaten	Lo	Poultry
24301000	Duck, roasted, NS as to skin eaten	Lo	Poultry
24301010	Duck, roasted, skin eaten	Lo	Poultry
24301020	Duck, roasted, skin not eaten	Lo	Poultry
24301210	Duck, coated, fried	Lo	Poultry
24302010	Duck, pressed, Chinese	Lo	Poultry
24400000	Cornish game hen, cooked, NS as t	Lo	Poultry
24400010	Cornish game hen, cooked, skin ea	Lo	Poultry
24400020	Cornish game hen, cooked, skin no	Lo	Poultry
24401010	Cornish game hen, roasted, skin ea	Lo	Poultry
24401020	Cornish game hen, roasted, skin no	Lo	Poultry
24402100	Dove, cooked, NS as to cooking me	Lo	Poultry
24403100	Quail, cooked	Lo	Poultry
24404100	Pheasant, cooked	Lo	Poultry
24701000	Chicken, baby food, NS as to strain	Lo	Baby Foods
24701010	Chicken, baby food, strained	Lo	Baby Foods
24701020	Chicken, baby food, junior	Lo	Baby Foods
24703000	Turkey, baby food, NS as to straine	Lo	Baby Foods
24703010	Turkey, baby food, strained	Lo	Baby Foods
24703020	Turkey, baby food, junior	Lo	Baby Foods
24705010	Chicken stick, baby food	Lo	Baby Foods
24706010	Turkey stick, baby food	Lo	Baby Foods
25110120	Beef liver, braised	Lo	Meats
25110140	Beef liver, fried	Lo	Meats
25110420	Chicken liver, braised	Lo	Meats
25110450	Chicken liver, fried	Lo	Meats
25112200	Liver paste or pate, chicken	Lo	Meats
25120000	Heart, cooked	Lo	Meats
25130000	Kidney, cooked	Lo	Meats
25150000	Brains, cooked	Lo	Meats
25160000	Tongue, cooked	Lo	Meats
25160110	Tongue, smoked, cured, or pickled,	Lo	Cured Meats/Poultry
25170110	Tripe, cooked	Lo	Meats
25170210	Chitterlings, cooked	Lo	Meats
25170310	Hog maws, cooked	Lo	Meats
25170420	Gizzard, cooked	Lo	Meats
25210110	Frankfurter or hot dog, NFS	Lo	Cured Meats/Poultry
25210150	Frankfurter or hot dog, cheese-fillec	Lo	Cured Meats/Poultry
25210170	Frankfurter or hot dog, chili-filled	Lo	Cured Meats/Poultry
25210210	Frankfurter or hot dog, beef	Lo	Cured Meats/Poultry
25210220	Frankfurter or hot dog, beef and po	Lo	Cured Meats/Poultry
25210230	Frankfurter or hot dog, beef and po	Lo	Cured Meats/Poultry
25210240	Frankfurter or hot dog, beef and po	Lo	Cured Meats/Poultry
25210250	Frankfurter or hot dog, meat and po	Lo	Cured Meats/Poultry
25210280	Frankfurter or hot dog, meat and po	Lo	Cured Meats/Poultry
25210290	Frankfurter or hot dog, meat and po	Lo	Cured Meats/Poultry
25210310	Frankfurter or hot dog, chicken	Lo	Cured Meats/Poultry
25210410	Frankfurter or hot dog, turkey	Lo	Cured Meats/Poultry
25210510	Frankfurter or hot dog, low salt	Lo	Cured Meats/Poultry

25210610	Frankfurter or hot dog, beef, lowfat	Lo	Cured Meats/Poultry
25210620	Frankfurter or hot dog, beef, reduce	Lo	Cured Meats/Poultry
25210700	Frankfurter or hot dog, meat & poul	Lo	Cured Meats/Poultry
25220010	Cold cut, NFS	Lo	Cured Meats/Poultry
25220100	Beef sausage, NFS	Lo	Cured Meats/Poultry
25220105	Beef sausage	Lo	Cured Meats/Poultry
25220106	Beef sausage, reduced fat	Lo	Cured Meats/Poultry
25220108	Beef sausage, reduced sodium	Lo	Cured Meats/Poultry
25220110	Beef sausage, brown and serve, lin	Lo	Cured Meats/Poultry
25220120	Beef sausage, smoked, stick	Lo	Cured Meats/Poultry
25220130	Beef sausage, smoked	Lo	Cured Meats/Poultry
25220140	Beef sausage, fresh, bulk, patty or l	Lo	Cured Meats/Poultry
25220150	Beef sausage with cheese	Lo	Cured Meats/Poultry
25220210	Blood sausage	Lo	Cured Meats/Poultry
25220350	Bratwurst	Lo	Cured Meats/Poultry
25220360	Bratwurst, with cheese	Lo	Cured Meats/Poultry
25220370	Bratwurst, beef, cooked	Lo	Cured Meats/Poultry
25220390	Bologna, beef, lowfat	Lo	Cured Meats/Poultry
25220400	Bologna, pork and beef	Lo	Cured Meats/Poultry
25220410	Bologna, NFS	Lo	Cured Meats/Poultry
25220420	Bologna, Lebanon	Lo	Cured Meats/Poultry
25220425	Bologna, made from any kind of me	Lo	Cured Meats/Poultry
25220430	Bologna, beef	Lo	Cured Meats/Poultry
25220435	Bologna, made from any kind of me	Lo	Cured Meats/Poultry
25220440	Bologna, turkey	Lo	Cured Meats/Poultry
25220445	Bologna, made from any kind of me	Lo	Cured Meats/Poultry
25220460	Bologna, pork	Lo	Cured Meats/Poultry
25220470	Bologna, beef, lower sodium	Lo	Cured Meats/Poultry
25220480	Bologna, chicken, beef, and pork	Lo	Cured Meats/Poultry
25220500	Bologna, beef and pork, lowfat	Lo	Cured Meats/Poultry
25220510	Capicola	Lo	Cured Meats/Poultry
25220650	Turkey or chicken and beef sausag	Lo	Cured Meats/Poultry
25220710	Chorizo	Lo	Cured Meats/Poultry
25220910	Head cheese	Lo	Cured Meats/Poultry
25221110	Knockwurst	Lo	Cured Meats/Poultry
25221210	Mortadella	Lo	Cured Meats/Poultry
25221215	Pastrami, NFS	Lo	Cured Meats/Poultry
25221220	Pastrami, made from any kind of m	Lo	Cured Meats/Poultry
25221250	Pepperoni, NFS	Lo	Cured Meats/Poultry
25221255	Pepperoni, reduced fat	Lo	Cured Meats/Poultry
25221260	Pepperoni, reduced sodium	Lo	Cured Meats/Poultry
25221310	Polish sausage	Lo	Cured Meats/Poultry
25221350	Italian sausage	Lo	Cured Meats/Poultry
25221400	Sausage, NFS	Lo	Cured Meats/Poultry
25221405	Pork sausage	Lo	Cured Meats/Poultry
25221406	Pork sausage, reduced fat	Lo	Cured Meats/Poultry
25221408	Pork sausage, reduced sodium	Lo	Cured Meats/Poultry
25221410	Pork sausage, fresh, bulk, patty or l	Lo	Cured Meats/Poultry
25221420	Pork sausage, brown and serve, co	Lo	Cured Meats/Poultry
25221430	Pork sausage, country style, fresh,	Lo	Cured Meats/Poultry
25221450	Pork sausage rice links	Lo	Cured Meats/Poultry

25221460	Pork and beef sausage	Lo	Cured Meats/Poultry
25221470	Pork and beef sausage, brown and	Lo	Cured Meats/Poultry
25221500	Salami, NFS	Lo	Cured Meats/Poultry
25221505	Salami, made from any type of mea	Lo	Cured Meats/Poultry
25221510	Salami, soft, cooked	Lo	Cured Meats/Poultry
25221515	Salami, made from any type of mea	Lo	Cured Meats/Poultry
25221520	Salami, dry or hard	Lo	Cured Meats/Poultry
25221530	Salami, beef	Lo	Cured Meats/Poultry
25221610	Scrapple, cooked	Lo	Cured Meats/Poultry
25221650	Smoked link sausage, pork	Lo	Cured Meats/Poultry
25221660	Smoked link sausage, pork and bee	Lo	Cured Meats/Poultry
25221680	Smoked sausage, pork	Lo	Cured Meats/Poultry
25221810	Thuringer	Lo	Cured Meats/Poultry
25221830	Turkey or chicken sausage	Lo	Cured Meats/Poultry
25221840	Turkey breakfast sausage, bulk, pa	Lo	Cured Meats/Poultry
25221850	Turkey sausage, smoked	Lo	Cured Meats/Poultry
25221855	Turkey or chicken sausage, reduce	Lo	Cured Meats/Poultry
25221860	Turkey or chicken sausage, reduce	Lo	Cured Meats/Poultry
25221870	Turkey or chicken and pork sausag	Lo	Cured Meats/Poultry
25221875	Turkey or chicken, pork, and beef s	Lo	Cured Meats/Poultry
25221880	Turkey or chicken, pork, and beef s	Lo	Cured Meats/Poultry
25221890	Turkey, pork, and beef sausage, lov	Lo	Cured Meats/Poultry
25221910	Vienna sausage, canned	Lo	Cured Meats/Poultry
25221920	Vienna sausage, chicken, canned	Lo	Cured Meats/Poultry
25221950	Pickled sausage	Lo	Cured Meats/Poultry
25230110	Luncheon meat, NFS	Lo	Cured Meats/Poultry
25230210	Ham, prepackaged or deli, luncheo	Lo	Cured Meats/Poultry
25230220	Ham, prepackaged or deli, luncheo	Lo	Cured Meats/Poultry
25230230	Ham, sliced, extra lean, prepackage	Lo	Cured Meats/Poultry
25230235	Ham, sliced, extra lean, lower sodiu	Lo	Cured Meats/Poultry
25230310	Chicken or turkey loaf, prepackage	Lo	Cured Meats/Poultry
25230320	Chicken, prepackaged or deli, lunch	Lo	Cured Meats/Poultry
25230340	Chicken, prepackaged or deli, lunch	Lo	Cured Meats/Poultry
25230410	Ham loaf, luncheon meat	Lo	Cured Meats/Poultry
25230420	Ham luncheon meat, loaf type	Lo	Cured Meats/Poultry
25230430	Ham and cheese loaf	Lo	Cured Meats/Poultry
25230450	Honey loaf	Lo	Cured Meats/Poultry
25230510	Ham, luncheon meat, chopped, mir	Lo	Cured Meats/Poultry
25230520	Ham, luncheon meat, chopped, mir	Lo	Cured Meats/Poultry
25230530	Ham and pork, canned luncheon m	Lo	Cured Meats/Poultry
25230540	Ham, pork and chicken, canned lun	Lo	Cured Meats/Poultry
25230550	Ham, pork, and chicken, canned lu	Lo	Cured Meats/Poultry
25230560	Liverwurst	Lo	Cured Meats/Poultry
25230610	Luncheon meat, loaf type	Lo	Cured Meats/Poultry
25230710	Sandwich loaf, luncheon meat	Lo	Cured Meats/Poultry
25230780	Turkey, prepackaged or deli, lunche	Lo	Cured Meats/Poultry
25230785	Turkey, prepackaged or deli, lunche	Lo	Cured Meats/Poultry
25230790	Turkey ham, sliced, extra lean, prep	Lo	Cured Meats/Poultry
25230800	Turkey ham, prepackaged or deli, lu	Lo	Cured Meats/Poultry
25230810	Veal loaf	Lo	Cured Meats/Poultry
25230820	Turkey pastrami	Lo	Cured Meats/Poultry

25230840	Turkey salami	Lo	Cured Meats/Poultry
25230900	Turkey or chicken breast, prepacka	Lo	Cured Meats/Poultry
25230905	Turkey or chicken breast, low salt, p	Lo	Cured Meats/Poultry
25231110	Beef, prepackaged or deli, luncheon	Lo	Cured Meats/Poultry
25231120	Beef, prepackaged or deli, luncheon	Lo	Cured Meats/Poultry
25231150	Corned beef, pressed	Lo	Cured Meats/Poultry
25240000	Meat spread or potted meat, NFS	Lo	Cured Meats/Poultry
25240110	Chicken salad spread	Lo	Cured Meats/Poultry
25240220	Ham salad spread	Lo	Cured Meats/Poultry
25240310	Roast beef spread	Lo	Cured Meats/Poultry
26100100	Fish, NS as to type, raw	Lo	Seafood
26100110	Fish, NS as to type, cooked, NS as	Lo	Seafood
26100120	Fish, NS as to type, baked or broile	Lo	Seafood
26100121	Fish, NS as to type, baked or broile	Lo	Seafood
26100122	Fish, NS as to type, baked or broile	Lo	Seafood
26100123	Fish, NS as to type, baked or broile	Lo	Seafood
26100130	Fish, NS as to type, coated, baked	Lo	Seafood
26100133	Fish, NS as to type, coated, baked	Lo	Seafood
26100140	Fish, NS as to type, coated, fried, m	Lo	Seafood
26100142	Fish, NS as to type, coated, fried, m	Lo	Seafood
26100143	Fish, NS as to type, coated, fried, n	Lo	Seafood
26100150	Fish, NS as to type, battered, fried	Lo	Seafood
26100160	Fish, NS as to type, steamed	Lo	Seafood
26100170	Fish, NS as to type, dried	Lo	Seafood
26100180	Fish, NS as to type, canned	Lo	Seafood
26100190	Fish, NS as to type, smoked	Lo	Seafood
26100200	Fish, NS as to type, from fast food	Lo	Seafood
26100210	Fish stick, patty, or fillet, NS as to ty	Lo	Seafood
26100220	Fish stick, patty, or fillet, NS as to ty	Lo	Seafood
26100230	Fish stick, patty, or fillet, NS as to ty	Lo	Seafood
26100240	Fish stick, patty, or fillet, NS as to ty	Lo	Seafood
26100250	Fish stick, patty, or fillet, NS as to ty	Lo	Seafood
26100260	Fish stick, patty or nugget from fast	Lo	Seafood
26100270	Fish stick, patty or nugget from rest	Lo	Seafood
26101110	Anchovy, cooked, NS as to cooking	Lo	Seafood
26101180	Anchovy, canned	Lo	Seafood
26105110	Carp, cooked, NS as to cooking me	Lo	Seafood
26105120	Carp, baked or broiled, fat added	Lo	Seafood
26105140	Carp, coated, fried	Lo	Seafood
26105160	Carp, steamed or poached	Lo	Seafood
26107110	Catfish, cooked, NS as to cooking r	Lo	Seafood
26107120	Catfish, baked or broiled, made with	Lo	Seafood
26107121	Catfish, baked or broiled, made with	Lo	Seafood
26107123	Catfish, baked or broiled, no added	Lo	Seafood
26107124	Catfish, baked or broiled, made with	Lo	Seafood
26107130	Catfish, coated, baked or broiled, m	Lo	Seafood
26107131	Catfish, coated, baked or broiled, m	Lo	Seafood
26107133	Catfish, coated, baked or broiled, no	Lo	Seafood
26107140	Catfish, coated, fried, made with oil	Lo	Seafood
26107143	Catfish, coated, fried, no added fat	Lo	Seafood
26107144	Catfish, coated, fried, made with co	Lo	Seafood

26107150	Catfish, battered, fried	Lo	Seafood
26107160	Catfish, steamed or poached	Lo	Seafood
26109110	Cod, cooked, NS as to cooking met	Lo	Seafood
26109120	Cod, baked or broiled, made with oi	Lo	Seafood
26109121	Cod, baked or broiled, made with bu	Lo	Seafood
26109122	Cod, baked or broiled, made with m	Lo	Seafood
26109123	Cod, baked or broiled, no added fat	Lo	Seafood
26109124	Cod, baked or broiled, made with co	Lo	Seafood
26109130	Cod, coated, baked or broiled, mad	Lo	Seafood
26109133	Cod, coated, baked or broiled, no a	Lo	Seafood
26109134	Cod, coated, baked or broiled, mad	Lo	Seafood
26109140	Cod, coated, fried, made with oil	Lo	Seafood
26109141	Cod, coated, fried, made with butte	Lo	Seafood
26109143	Cod, coated, fried, no added fat	Lo	Seafood
26109144	Cod, coated, fried, made with cooki	Lo	Seafood
26109150	Cod, battered, fried	Lo	Seafood
26109160	Cod, steamed or poached	Lo	Seafood
26109170	Cod, dried, salted	Lo	Seafood
26109180	Cod, dried, salted, salt removed in w	Lo	Seafood
26111110	Croaker, cooked, NS as to cooking	Lo	Seafood
26111120	Croaker, baked or broiled, fat added	Lo	Seafood
26111121	Croaker, baked or broiled, no added	Lo	Seafood
26111130	Croaker, coated, baked or broiled, f	Lo	Seafood
26111140	Croaker, coated, fried	Lo	Seafood
26111160	Croaker, steamed or poached	Lo	Seafood
26113110	Eel, cooked, NS as to cooking meth	Lo	Seafood
26113160	Eel, steamed or poached	Lo	Seafood
26113190	Eel, smoked	Lo	Seafood
26115110	Flounder, cooked, NS as to cooking	Lo	Seafood
26115120	Flounder, baked or broiled, made w	Lo	Seafood
26115121	Flounder, baked or broiled, made w	Lo	Seafood
26115122	Flounder, baked or broiled, made w	Lo	Seafood
26115123	Flounder, baked or broiled, no adde	Lo	Seafood
26115124	Flounder, baked or broiled, made w	Lo	Seafood
26115130	Flounder, coated, baked or broiled,	Lo	Seafood
26115132	Flounder, coated, baked or broiled,	Lo	Seafood
26115133	Flounder, coated, baked or broiled,	Lo	Seafood
26115140	Flounder, coated, fried, made with o	Lo	Seafood
26115141	Flounder, coated, fried, made with b	Lo	Seafood
26115150	Flounder, battered, fried	Lo	Seafood
26115160	Flounder, steamed or poached	Lo	Seafood
26115190	Flounder, smoked	Lo	Seafood
26117110	Haddock, cooked, NS as to cooking	Lo	Seafood
26117120	Haddock, baked or broiled, fat adde	Lo	Seafood
26117121	Haddock, baked or broiled, no adde	Lo	Seafood
26117130	Haddock, coated, baked or broiled,	Lo	Seafood
26117131	Haddock, coated, baked or broiled,	Lo	Seafood
26117140	Haddock, coated, fried	Lo	Seafood
26117150	Haddock, battered, fried	Lo	Seafood
26117160	Haddock, steamed or poached	Lo	Seafood
26118020	Halibut, baked or broiled, made with	Lo	Seafood

26118023	Halibut, baked or broiled, no added	Lo	Seafood
26118024	Halibut, baked or broiled, made with	Lo	Seafood
26118030	Halibut, coated, baked or broiled, m	Lo	Seafood
26118050	Halibut, steamed or poached	Lo	Seafood
26119100	Herring, raw	Lo	Seafood
26119110	Herring, cooked, NS as to cooking r	Lo	Seafood
26119120	Herring, baked or broiled, fat added	Lo	Seafood
26119121	Herring, baked or broiled, no added	Lo	Seafood
26119130	Herring, coated, baked or broiled, fa	Lo	Seafood
26119131	Herring, coated, baked or broiled, n	Lo	Seafood
26119140	Herring, coated, fried	Lo	Seafood
26119160	Herring, pickled, in cream sauce	Lo	Seafood
26119180	Herring, pickled	Lo	Seafood
26119190	Herring, smoked, kippered	Lo	Seafood
26121100	Mackerel, raw	Lo	Seafood
26121110	Mackerel, cooked, NS as to cooking	Lo	Seafood
26121120	Mackerel, baked or broiled, fat adde	Lo	Seafood
26121121	Mackerel, baked or broiled, no adde	Lo	Seafood
26121140	Mackerel, coated, fried	Lo	Seafood
26121160	Mackerel, pickled	Lo	Seafood
26121180	Mackerel, canned	Lo	Seafood
26123120	Mullet, baked or broiled, fat added	Lo	Seafood
26123121	Mullet, baked or broiled, no added f	Lo	Seafood
26123140	Mullet, coated, fried	Lo	Seafood
26123160	Mullet, steamed or poached	Lo	Seafood
26125110	Ocean perch, cooked, NS as to coo	Lo	Seafood
26125120	Ocean perch, baked or broiled, fat a	Lo	Seafood
26125121	Ocean perch, baked or broiled, no a	Lo	Seafood
26125130	Ocean perch, coated, baked or broi	Lo	Seafood
26125140	Ocean perch, coated, fried	Lo	Seafood
26125150	Ocean perch, battered, fried	Lo	Seafood
26125160	Ocean perch, steamed or poached	Lo	Seafood
26127110	Perch, cooked, NS as to cooking m	Lo	Seafood
26127120	Perch, baked or broiled, made with	Lo	Seafood
26127121	Perch, baked or broiled, made with	Lo	Seafood
26127123	Perch, baked or broiled, no added f	Lo	Seafood
26127130	Perch, coated, baked or broiled, ma	Lo	Seafood
26127133	Perch, coated, baked or broiled, no	Lo	Seafood
26127140	Perch, coated, fried, made with oil	Lo	Seafood
26127141	Perch, coated, fried, made with butt	Lo	Seafood
26127143	Perch, coated, fried, no added fat	Lo	Seafood
26127150	Perch, battered, fried	Lo	Seafood
26127160	Perch, steamed or poached	Lo	Seafood
26129120	Pike, baked or broiled, fat added	Lo	Seafood
26129140	Pike, coated, fried	Lo	Seafood
26131100	Pompano, raw	Lo	Seafood
26131110	Pompano, cooked, NS as to cookin	Lo	Seafood
26131120	Pompano, baked or broiled, fat add	Lo	Seafood
26131121	Pompano, baked or broiled, no add	Lo	Seafood
26131131	Pompano, coated, baked or broiled	Lo	Seafood
26131140	Pompano, coated, fried	Lo	Seafood

26131150	Pompano, battered, fried	Lo	Seafood
26131160	Pompano, steamed or poached	Lo	Seafood
26131190	Pompano, smoked	Lo	Seafood
26133110	Porgy, cooked, NS as to cooking m	Lo	Seafood
26133120	Porgy, baked or broiled, fat added	Lo	Seafood
26133121	Porgy, baked or broiled, no added f	Lo	Seafood
26133130	Porgy, coated, baked or broiled, fat	Lo	Seafood
26133140	Porgy, coated, fried	Lo	Seafood
26133150	Porgy, battered, fried	Lo	Seafood
26133160	Porgy, steamed or poached	Lo	Seafood
26135120	Ray, baked or broiled, fat added	Lo	Seafood
26137100	Salmon, raw	Lo	Seafood
26137110	Salmon, cooked, NS as to cooking	Lo	Seafood
26137120	Salmon, baked or broiled, made wit	Lo	Seafood
26137121	Salmon, baked or broiled, made wit	Lo	Seafood
26137122	Salmon, baked or broiled, made wit	Lo	Seafood
26137123	Salmon, baked or broiled, no addec	Lo	Seafood
26137124	Salmon, baked or broiled, made wit	Lo	Seafood
26137130	Salmon, coated, baked or broiled, r	Lo	Seafood
26137131	Salmon, coated, baked or broiled, r	Lo	Seafood
26137133	Salmon, coated, baked or broiled, r	Lo	Seafood
26137134	Salmon, coated, baked or broiled, r	Lo	Seafood
26137140	Salmon, coated, fried, made with oi	Lo	Seafood
26137141	Salmon, coated, fried, made with bu	Lo	Seafood
26137142	Salmon, coated, fried, made with m	Lo	Seafood
26137143	Salmon, coated, fried, no added fat	Lo	Seafood
26137150	Salmon, battered, fried	Lo	Seafood
26137160	Salmon, steamed or poached	Lo	Seafood
26137170	Salmon, dried	Lo	Seafood
26137180	Salmon, canned	Lo	Seafood
26137190	Salmon, smoked	Lo	Seafood
26139110	Sardines, cooked	Lo	Seafood
26139170	Sardines, dried	Lo	Seafood
26139180	Sardines, canned in oil	Lo	Seafood
26139190	Sardines, skinless, boneless, packe	Lo	Seafood
26141110	Sea bass, cooked, NS as to cookin	Lo	Seafood
26141120	Sea bass, baked or broiled, fat add	Lo	Seafood
26141121	Sea bass, baked or broiled, no add	Lo	Seafood
26141130	Sea bass, coated, baked or broiled,	Lo	Seafood
26141140	Sea bass, coated, fried	Lo	Seafood
26141160	Sea bass, steamed or poached	Lo	Seafood
26143110	Shark, cooked, NS as to cooking m	Lo	Seafood
26143120	Shark, baked or broiled, fat added	Lo	Seafood
26143160	Shark, steamed or poached	Lo	Seafood
26145140	Smelt, floured or breaded, fried	Lo	Seafood
26147110	Sturgeon, cooked, NS as to cookin	Lo	Seafood
26149110	Swordfish, cooked, NS as to cookin	Lo	Seafood
26149120	Swordfish, baked or broiled, fat add	Lo	Seafood
26149121	Swordfish, baked or broiled, no add	Lo	Seafood
26149130	Swordfish, coated, baked or broiled	Lo	Seafood
26149140	Swordfish, coated, fried	Lo	Seafood

26149160	Swordfish, steamed or poached	Lo	Seafood
26151110	Trout, cooked, NS as to cooking me	Lo	Seafood
26151120	Trout, baked or broiled, made with	Lo	Seafood
26151121	Trout, baked or broiled, made with	Lo	Seafood
26151122	Trout, baked or broiled, made with	Lo	Seafood
26151123	Trout, baked or broiled, no added fa	Lo	Seafood
26151130	Trout, coated, baked or broiled, ma	Lo	Seafood
26151133	Trout, coated, baked or broiled, no	Lo	Seafood
26151140	Trout, coated, fried, made with oil	Lo	Seafood
26151142	Trout, coated, fried, made with mar	Lo	Seafood
26151143	Trout, coated, fried, no added fat	Lo	Seafood
26151150	Trout, battered, fried	Lo	Seafood
26151160	Trout, steamed or poached	Lo	Seafood
26151190	Trout, smoked	Lo	Seafood
26153110	Tuna, fresh, cooked, NS as to cook	Lo	Seafood
26153120	Tuna, fresh, baked or broiled, fat ac	Lo	Seafood
26153122	Tuna, fresh, baked or broiled, no ac	Lo	Seafood
26153130	Tuna, fresh, coated, baked or broile	Lo	Seafood
26153131	Tuna, fresh, coated, baked or broile	Lo	Seafood
26153140	Tuna, fresh, coated, fried	Lo	Seafood
26153160	Tuna, fresh, steamed or poached	Lo	Seafood
26155110	Tuna, canned, NS as to oil or water	Lo	Seafood
26155180	Tuna, canned, oil pack	Lo	Seafood
26155190	Tuna, canned, water pack	Lo	Seafood
26157110	Whiting, cooked, NS as to cooking	Lo	Seafood
26157120	Whiting, baked or broiled, made wit	Lo	Seafood
26157121	Whiting, baked or broiled, made wit	Lo	Seafood
26157122	Whiting, baked or broiled, made wit	Lo	Seafood
26157123	Whiting, baked or broiled, no added	Lo	Seafood
26157124	Whiting, baked or broiled, made wit	Lo	Seafood
26157130	Whiting, coated, baked or broiled, n	Lo	Seafood
26157132	Whiting, coated, baked or broiled, n	Lo	Seafood
26157133	Whiting, coated, baked or broiled, n	Lo	Seafood
26157140	Whiting, coated, fried, made with oi	Lo	Seafood
26157150	Whiting, battered, fried	Lo	Seafood
26157160	Whiting, steamed or poached	Lo	Seafood
26158000	Tilapia, cooked, NS as to cooking m	Lo	Seafood
26158010	Tilapia, baked or broiled, made with	Lo	Seafood
26158011	Tilapia, baked or broiled, made with	Lo	Seafood
26158012	Tilapia, baked or broiled, made with	Lo	Seafood
26158013	Tilapia, baked or broiled, no added	Lo	Seafood
26158014	Tilapia, baked or broiled, made with	Lo	Seafood
26158020	Tilapia, coated, baked or broiled, m	Lo	Seafood
26158021	Tilapia, coated, baked or broiled, m	Lo	Seafood
26158023	Tilapia, coated, baked or broiled, no	Lo	Seafood
26158024	Tilapia, coated, baked or broiled, m	Lo	Seafood
26158030	Tilapia, coated, fried, made with oil	Lo	Seafood
26158031	Tilapia, coated, fried, made with but	Lo	Seafood
26158032	Tilapia, coated, fried, made with ma	Lo	Seafood
26158033	Tilapia, coated, fried, no added fat	Lo	Seafood
26158034	Tilapia, coated, fried, made with coo	Lo	Seafood

26158040	Tilapia, battered, fried	Lo	Seafood
26158050	Tilapia, steamed or poached	Lo	Seafood
26203110	Frog legs, NS as to cooking methoc	Lo	Seafood
26205110	Octopus, cooked, NS as to cooking	Lo	Seafood
26205160	Octopus, steamed	Lo	Seafood
26207110	Roe, shad, cooked	Lo	Seafood
26211100	Roe, sturgeon	Lo	Seafood
26213100	Squid, raw	Lo	Seafood
26213120	Squid, baked or broiled, fat added	Lo	Seafood
26213140	Squid, coated, fried	Lo	Seafood
26213160	Squid, steamed or boiled	Lo	Seafood
26213170	Squid, dried	Lo	Seafood
26213190	Squid, canned	Lo	Seafood
26215120	Turtle, cooked, NS as to cooking m	Lo	Seafood
26301110	Abalone, cooked, NS as to cooking	Lo	Seafood
26301140	Abalone, floured or breaded, fried	Lo	Seafood
26303100	Clams, raw	Lo	Seafood
26303110	Clams, cooked, NS as to cooking m	Lo	Seafood
26303120	Clams, baked or broiled, fat added	Lo	Seafood
26303121	Clams, baked or broiled, no added	Lo	Seafood
26303140	Clams, coated, fried	Lo	Seafood
26303150	Clams, battered, fried	Lo	Seafood
26303160	Clams, steamed or boiled	Lo	Seafood
26303180	Clams, canned	Lo	Seafood
26305110	Crab, cooked, NS as to cooking me	Lo	Seafood
26305120	Crab, baked or broiled, fat added	Lo	Seafood
26305121	Crab, baked or broiled, no added fa	Lo	Seafood
26305130	Crab, coated, baked or broiled, fat a	Lo	Seafood
26305160	Crab, hard shell, steamed	Lo	Seafood
26305180	Crab, canned	Lo	Seafood
26307140	Crab, soft shell, coated, fried	Lo	Seafood
26309140	Crayfish, coated, fried	Lo	Seafood
26309160	Crayfish, boiled or steamed	Lo	Seafood
26311110	Lobster, cooked, NS as to cooking	Lo	Seafood
26311120	Lobster, baked or broiled, fat addec	Lo	Seafood
26311121	Lobster, baked or broiled, no addec	Lo	Seafood
26311140	Lobster, coated, fried	Lo	Seafood
26311160	Lobster, steamed or boiled	Lo	Seafood
26313100	Mussels, raw	Lo	Seafood
26313110	Mussels, cooked, NS as to cooking	Lo	Seafood
26313160	Mussels, steamed or poached	Lo	Seafood
26315110	Oysters, cooked, NS as to cooking	Lo	Seafood
26315120	Oysters, baked or broiled, fat addec	Lo	Seafood
26315121	Oysters, baked or broiled, no addec	Lo	Seafood
26315130	Oysters, steamed	Lo	Seafood
26315140	Oysters, coated, fried	Lo	Seafood
26315150	Oysters, battered, fried	Lo	Seafood
26315160	Oysters, coated, baked or broiled, f	Lo	Seafood
26315180	Oysters, canned	Lo	Seafood
26315190	Oysters, smoked	Lo	Seafood
26317110	Scallops, cooked, NS as to cooking	Lo	Seafood

26317120	Scallops, baked or broiled, fat adde	Lo	Seafood
26317121	Scallops, baked or broiled, no adde	Lo	Seafood
26317130	Scallops, steamed or boiled	Lo	Seafood
26317140	Scallops, coated, fried	Lo	Seafood
26317150	Scallops, battered, fried	Lo	Seafood
26317160	Scallops, coated, baked or broiled,	Lo	Seafood
26319110	Shrimp, cooked, NS as to cooking r	Lo	Seafood
26319120	Shrimp, baked or broiled, made witl	Lo	Seafood
26319121	Shrimp, baked or broiled, made witl	Lo	Seafood
26319122	Shrimp, baked or broiled, made witl	Lo	Seafood
26319123	Shrimp, baked or broiled, no added	Lo	Seafood
26319124	Shrimp, baked or broiled, made witl	Lo	Seafood
26319130	Shrimp, steamed or boiled	Lo	Seafood
26319140	Shrimp, coated, fried, made with oil	Lo	Seafood
26319141	Shrimp, coated, fried, made with bu	Lo	Seafood
26319142	Shrimp, coated, fried, made with ma	Lo	Seafood
26319143	Shrimp, coated, fried, no added fat	Lo	Seafood
26319145	Shrimp, coated, fried, from fast foo	Lo	Seafood
26319160	Shrimp, coated, baked or broiled, m	Lo	Seafood
26319161	Shrimp, coated, baked or broiled, m	Lo	Seafood
26319163	Shrimp, coated, baked or broiled, n	Lo	Seafood
26319164	Shrimp, coated, baked or broiled, m	Lo	Seafood
26319170	Shrimp, dried	Lo	Seafood
26319180	Shrimp, canned	Lo	Seafood
26321110	Snails, cooked, NS as to cooking m	Lo	Seafood
27111000	Beef with tomato-based sauce	Lo	Mixed Dishes - Meat, Poultry, Seafood
27111050	Spaghetti sauce with beef or meat c	Lo	Condiments and Sauces
27111100	Beef goulash	Lo	Mixed Dishes - Meat, Poultry, Seafood
27111200	Beef burgundy	Lo	Mixed Dishes - Meat, Poultry, Seafood
27111300	Beef stew, no potatoes, tomato-bas	Lo	Mixed Dishes - Meat, Poultry, Seafood
27111310	Beef stew, no potatoes, tomato-bas	Lo	Mixed Dishes - Meat, Poultry, Seafood
27111400	Chili con carne, NS as to beans	Lo	Mixed Dishes - Meat, Poultry, Seafood
27111405	Chili con carne with beans, from res	Lo	Mixed Dishes - Meat, Poultry, Seafood
27111406	Chili con carne with beans, home re	Lo	Mixed Dishes - Meat, Poultry, Seafood
27111407	Chili con carne with beans, canned	Lo	Mixed Dishes - Meat, Poultry, Seafood
27111410	Chili con carne with beans	Lo	Mixed Dishes - Meat, Poultry, Seafood
27111420	Chili con carne without beans	Lo	Mixed Dishes - Meat, Poultry, Seafood
27111430	Chili con carne, NS as to beans, wit	Lo	Mixed Dishes - Meat, Poultry, Seafood
27111440	Chili con carne with beans and che	Lo	Mixed Dishes - Meat, Poultry, Seafood
27111500	Beef sloppy joe, no bun	Lo	Mixed Dishes - Meat, Poultry, Seafood
27112000	Beef with gravy	Lo	Mixed Dishes - Meat, Poultry, Seafood
27112010	Salisbury steak with gravy	Lo	Mixed Dishes - Meat, Poultry, Seafood
27113000	Beef with cream or white sauce	Lo	Mixed Dishes - Meat, Poultry, Seafood
27113100	Beef stroganoff	Lo	Mixed Dishes - Meat, Poultry, Seafood
27113200	Creamed chipped or dried beef	Lo	Mixed Dishes - Meat, Poultry, Seafood
27113300	Swedish meatballs with cream or w	Lo	Mixed Dishes - Meat, Poultry, Seafood
27114000	Beef with mushroom sauce	Lo	Mixed Dishes - Meat, Poultry, Seafood
27115000	Beef with soy-based sauce	Lo	Mixed Dishes - Asian
27115100	Steak teriyaki	Lo	Mixed Dishes - Asian
27116100	Beef curry	Lo	Mixed Dishes - Meat, Poultry, Seafood
27116110	Beef curry with rice	Lo	Mixed Dishes - Meat, Poultry, Seafood

27116200	Beef with barbecue sauce	Lo	Mixed Dishes - Meat, Poultry, Seafood
27116300	Beef with sweet and sour sauce	Lo	Mixed Dishes - Asian
27116350	Stewed seasoned ground beef, Me	Lo	Mixed Dishes - Meat, Poultry, Seafood
27118110	Meatballs, Puerto Rican style	Lo	Mixed Dishes - Meat, Poultry, Seafood
27118120	Stewed seasoned ground beef, Pue	Lo	Mixed Dishes - Meat, Poultry, Seafood
27118130	Stewed dried beef, Puerto Rican st	Lo	Mixed Dishes - Meat, Poultry, Seafood
27118180	Beef stew, meat with gravy, no pota	Lo	Mixed Dishes - Meat, Poultry, Seafood
27120020	Ham or pork with gravy	Lo	Mixed Dishes - Meat, Poultry, Seafood
27120030	Ham or pork with barbecue sauce	Lo	Mixed Dishes - Meat, Poultry, Seafood
27120060	Sweet and sour pork	Lo	Mixed Dishes - Asian
27120080	Ham stroganoff	Lo	Mixed Dishes - Meat, Poultry, Seafood
27120090	Ham or pork with mushroom sauce	Lo	Mixed Dishes - Meat, Poultry, Seafood
27120100	Ham or pork with tomato-based sa	Lo	Mixed Dishes - Meat, Poultry, Seafood
27120110	Sausage with tomato-based sauce	Lo	Mixed Dishes - Meat, Poultry, Seafood
27120120	Sausage gravy	Lo	Mixed Dishes - Meat, Poultry, Seafood
27120130	Pork stew, no potatoes, tomato-bas	Lo	Mixed Dishes - Meat, Poultry, Seafood
27120150	Pork or ham with soy-based sauce	Lo	Mixed Dishes - Asian
27120160	Pork curry	Lo	Mixed Dishes - Meat, Poultry, Seafood
27120210	Frankfurter or hot dog, with chili, no	Lo	Mixed Dishes - Meat, Poultry, Seafood
27120250	Frankfurters or hot dogs with tomat	Lo	Mixed Dishes - Meat, Poultry, Seafood
27121000	Pork with chili and tomatoes	Lo	Mixed Dishes - Meat, Poultry, Seafood
27121010	Stewed pork, Puerto Rican style	Lo	Mixed Dishes - Meat, Poultry, Seafood
27121410	Chili con carne with beans, made w	Lo	Mixed Dishes - Meat, Poultry, Seafood
27130010	Lamb or mutton with gravy	Lo	Mixed Dishes - Meat, Poultry, Seafood
27130040	Spaghetti sauce with lamb or mutto	Lo	Condiments and Sauces
27130100	Lamb or mutton curry	Lo	Mixed Dishes - Meat, Poultry, Seafood
27133010	Stewed goat, Puerto Rican style	Lo	Mixed Dishes - Meat, Poultry, Seafood
27135010	Veal with gravy	Lo	Mixed Dishes - Meat, Poultry, Seafood
27135040	Veal with butter sauce	Lo	Mixed Dishes - Meat, Poultry, Seafood
27135050	Veal Marsala	Lo	Mixed Dishes - Meat, Poultry, Seafood
27135110	Veal parmigiana	Lo	Mixed Dishes - Meat, Poultry, Seafood
27136050	Venison or deer with tomato-based	Lo	Mixed Dishes - Meat, Poultry, Seafood
27136080	Venison or deer with gravy	Lo	Mixed Dishes - Meat, Poultry, Seafood
27136100	Chili con carne with venison/deer a	Lo	Mixed Dishes - Meat, Poultry, Seafood
27141000	Chicken or turkey cacciatore	Lo	Mixed Dishes - Meat, Poultry, Seafood
27141030	Spaghetti sauce with poultry	Lo	Condiments and Sauces
27141035	Spaghetti sauce with poultry and ad	Lo	Condiments and Sauces
27141050	Stewed chicken with tomato-based	Lo	Mixed Dishes - Meat, Poultry, Seafood
27141500	Chili con carne with chicken or turk	Lo	Mixed Dishes - Meat, Poultry, Seafood
27142000	Chicken with gravy	Lo	Mixed Dishes - Meat, Poultry, Seafood
27142100	Chicken or turkey fricassee	Lo	Mixed Dishes - Meat, Poultry, Seafood
27142200	Turkey with gravy	Lo	Mixed Dishes - Meat, Poultry, Seafood
27143000	Chicken or turkey with cream sauce	Lo	Mixed Dishes - Meat, Poultry, Seafood
27144000	Chicken or turkey with mushroom s	Lo	Mixed Dishes - Meat, Poultry, Seafood
27145000	Chicken or turkey with teriyaki	Lo	Mixed Dishes - Asian
27146000	Chicken or turkey with barbecue sa	Lo	Poultry
27146010	Chicken or turkey with barbecue sa	Lo	Poultry
27146011	Chicken, shredded or pulled, with b	Lo	Mixed Dishes - Meat, Poultry, Seafood
27146050	Chicken wing with hot pepper sauce	Lo	Poultry
27146100	Sweet and sour chicken or turkey	Lo	Mixed Dishes - Asian
27146110	Sweet and sour chicken or turkey, v	Lo	Mixed Dishes - Asian

27146150	Chicken curry	Lo	Mixed Dishes - Meat, Poultry, Seafood
27146155	Chicken curry with rice	Lo	Mixed Dishes - Meat, Poultry, Seafood
27146160	Chicken with mole sauce	Lo	Mixed Dishes - Meat, Poultry, Seafood
27146200	Chicken or turkey with cheese sauc	Lo	Mixed Dishes - Meat, Poultry, Seafood
27146250	Chicken or turkey cordon bleu	Lo	Mixed Dishes - Meat, Poultry, Seafood
27146300	Chicken or turkey parmigiana	Lo	Mixed Dishes - Meat, Poultry, Seafood
27146350	Orange chicken	Lo	Mixed Dishes - Asian
27146360	Sesame chicken	Lo	Mixed Dishes - Asian
27146400	Chicken kiev	Lo	Mixed Dishes - Meat, Poultry, Seafood
27148010	Stuffed chicken, drumstick or breas	Lo	Mixed Dishes - Meat, Poultry, Seafood
27150010	Fish with cream or white sauce, not	Lo	Mixed Dishes - Meat, Poultry, Seafood
27150020	Crab, deviled	Lo	Mixed Dishes - Meat, Poultry, Seafood
27150030	Crab imperial	Lo	Mixed Dishes - Meat, Poultry, Seafood
27150060	Lobster newburg	Lo	Mixed Dishes - Meat, Poultry, Seafood
27150070	Lobster with butter sauce	Lo	Seafood
27150100	Shrimp curry	Lo	Mixed Dishes - Meat, Poultry, Seafood
27150110	Shrimp cocktail	Lo	Seafood
27150120	Tuna with cream or white sauce	Lo	Mixed Dishes - Meat, Poultry, Seafood
27150130	Seafood newburg	Lo	Mixed Dishes - Meat, Poultry, Seafood
27150140	Seafood sauce	Lo	Seafood
27150151	Spaghetti sauce with seafood	Lo	Condiments and Sauces
27150155	Spaghetti sauce with seafood and a	Lo	Condiments and Sauces
27150160	Shrimp with lobster sauce	Lo	Mixed Dishes - Meat, Poultry, Seafood
27150170	Sweet and sour shrimp	Lo	Mixed Dishes - Asian
27150190	Lobster sauce	Lo	Condiments and Sauces
27150200	Oyster sauce	Lo	Condiments and Sauces
27150210	Fish sauce	Lo	Condiments and Sauces
27150230	Shrimp scampi	Lo	Seafood
27150250	Fish moochim	Lo	Mixed Dishes - Meat, Poultry, Seafood
27150310	Fish with tomato-based sauce	Lo	Mixed Dishes - Meat, Poultry, Seafood
27150320	Fish curry	Lo	Mixed Dishes - Meat, Poultry, Seafood
27150325	Fish curry with rice	Lo	Mixed Dishes - Meat, Poultry, Seafood
27150330	Mussels with tomato-based sauce	Lo	Mixed Dishes - Meat, Poultry, Seafood
27150350	Sardines with tomato-based sauce	Lo	Mixed Dishes - Meat, Poultry, Seafood
27150370	Sardines with mustard sauce	Lo	Mixed Dishes - Meat, Poultry, Seafood
27150410	Shrimp teriyaki	Lo	Mixed Dishes - Asian
27150510	Scallops with cheese sauce	Lo	Mixed Dishes - Meat, Poultry, Seafood
27151040	Crabs in tomato-based sauce, Puer	Lo	Mixed Dishes - Meat, Poultry, Seafood
27151050	Shrimp in garlic sauce, Puerto Rica	Lo	Mixed Dishes - Meat, Poultry, Seafood
27151070	Stewed codfish, no potatoes, Puert	Lo	Mixed Dishes - Meat, Poultry, Seafood
27160010	Meat with barbecue sauce, NS as t	Lo	Mixed Dishes - Meat, Poultry, Seafood
27160100	Meatballs, NS as to type of meat, w	Lo	Mixed Dishes - Meat, Poultry, Seafood
27162010	Meat with tomato-based sauce	Lo	Mixed Dishes - Meat, Poultry, Seafood
27162040	Spaghetti sauce with meat	Lo	Condiments and Sauces
27162050	Spaghetti sauce with combination o	Lo	Condiments and Sauces
27162060	Spaghetti sauce with meat and add	Lo	Condiments and Sauces
27162500	Stewed, seasoned, ground beef and	Lo	Mixed Dishes - Meat, Poultry, Seafood
27163010	Meat with gravy, NS as to type of m	Lo	Mixed Dishes - Meat, Poultry, Seafood
27211000	Beef and potatoes, no sauce	Lo	Mixed Dishes - Meat, Poultry, Seafood
27211100	Beef stew with potatoes, tomato-ba	Lo	Mixed Dishes - Meat, Poultry, Seafood
27211110	Beef stew with potatoes, tomato-ba	Lo	Mixed Dishes - Meat, Poultry, Seafood

27211150	Beef goulash with potatoes	Lo	Mixed Dishes - Meat, Poultry, Seafood
27211190	Beef and potatoes with cream sauc	Lo	Mixed Dishes - Meat, Poultry, Seafood
27211200	Beef stew with potatoes, gravy	Lo	Mixed Dishes - Meat, Poultry, Seafood
27211300	Beef, roast, hash	Lo	Mixed Dishes - Meat, Poultry, Seafood
27211400	Corned beef hash	Lo	Mixed Dishes - Meat, Poultry, Seafood
27211500	Beef and potatoes with cheese sau	Lo	Mixed Dishes - Meat, Poultry, Seafood
27211550	Stewed, seasoned, ground beef wit	Lo	Mixed Dishes - Meat, Poultry, Seafood
27212000	Beef and noodles, no sauce	Lo	Mixed Dishes - Meat, Poultry, Seafood
27212050	Beef and macaroni with cheese sau	Lo	Mixed Dishes - Meat, Poultry, Seafood
27212100	Beef and noodles with tomato-base	Lo	Mixed Dishes - Meat, Poultry, Seafood
27212120	Chili con carne with beans and mac	Lo	Mixed Dishes - Meat, Poultry, Seafood
27212150	Beef goulash with noodles	Lo	Mixed Dishes - Meat, Poultry, Seafood
27212200	Beef and noodles with gravy	Lo	Mixed Dishes - Meat, Poultry, Seafood
27212300	Beef and noodles with cream or wh	Lo	Mixed Dishes - Meat, Poultry, Seafood
27212350	Beef stroganoff with noodles	Lo	Mixed Dishes - Meat, Poultry, Seafood
27212400	Beef and noodles with mushroom s	Lo	Mixed Dishes - Meat, Poultry, Seafood
27212500	Beef and noodles with soy-based sa	Lo	Mixed Dishes - Asian
27213000	Beef and rice, no sauce	Lo	Mixed Dishes - Meat, Poultry, Seafood
27213010	Biryani with meat	Lo	Mixed Dishes - Grain-based
27213100	Beef and rice with tomato-based sa	Lo	Mixed Dishes - Meat, Poultry, Seafood
27213120	Porcupine balls with tomato-based	Lo	Mixed Dishes - Meat, Poultry, Seafood
27213150	Chili con carne with beans and rice	Lo	Mixed Dishes - Meat, Poultry, Seafood
27213200	Beef and rice with gravy	Lo	Mixed Dishes - Meat, Poultry, Seafood
27213300	Beef and rice with cream sauce	Lo	Mixed Dishes - Meat, Poultry, Seafood
27213400	Beef and rice with mushroom sauce	Lo	Mixed Dishes - Meat, Poultry, Seafood
27213420	Porcupine balls with mushroom sau	Lo	Mixed Dishes - Meat, Poultry, Seafood
27213500	Beef and rice with soy-based sauce	Lo	Mixed Dishes - Asian
27213600	Beef and rice with cheese sauce	Lo	Mixed Dishes - Meat, Poultry, Seafood
27214100	Meat loaf made with beef	Lo	Mixed Dishes - Meat, Poultry, Seafood
27214110	Meat loaf made with beef, with toma	Lo	Mixed Dishes - Meat, Poultry, Seafood
27218210	Beef stew with potatoes, Puerto Ric	Lo	Mixed Dishes - Meat, Poultry, Seafood
27218310	Stewed corned beef, Puerto Rican s	Lo	Mixed Dishes - Meat, Poultry, Seafood
27220010	Meat loaf made with ham	Lo	Mixed Dishes - Meat, Poultry, Seafood
27220020	Ham and noodles with cream or wh	Lo	Mixed Dishes - Meat, Poultry, Seafood
27220030	Ham and rice with mushroom sauce	Lo	Mixed Dishes - Meat, Poultry, Seafood
27220050	Ham or pork with stuffing	Lo	Mixed Dishes - Meat, Poultry, Seafood
27220080	Ham croquette	Lo	Mixed Dishes - Meat, Poultry, Seafood
27220110	Pork and rice with tomato-based sa	Lo	Mixed Dishes - Meat, Poultry, Seafood
27220120	Sausage and rice with tomato-base	Lo	Mixed Dishes - Meat, Poultry, Seafood
27220150	Sausage and rice with mushroom s	Lo	Mixed Dishes - Meat, Poultry, Seafood
27220170	Sausage and rice with cheese sauc	Lo	Mixed Dishes - Meat, Poultry, Seafood
27220190	Sausage and noodles with cream o	Lo	Mixed Dishes - Meat, Poultry, Seafood
27220210	Ham and noodles, no sauce	Lo	Mixed Dishes - Meat, Poultry, Seafood
27220310	Ham or pork and rice, no sauce	Lo	Mixed Dishes - Meat, Poultry, Seafood
27220510	Ham or pork and potatoes with grav	Lo	Mixed Dishes - Meat, Poultry, Seafood
27220520	Ham or pork and potatoes with che	Lo	Mixed Dishes - Meat, Poultry, Seafood
27221100	Stewed pig's feet, Puerto Rican styl	Lo	Mixed Dishes - Meat, Poultry, Seafood
27221150	Pork stew, with potatoes, tomato-ba	Lo	Mixed Dishes - Meat, Poultry, Seafood
27230010	Lamb or mutton loaf	Lo	Mixed Dishes - Meat, Poultry, Seafood
27231000	Lamb or mutton and potatoes with g	Lo	Mixed Dishes - Meat, Poultry, Seafood
27232000	Lamb or mutton and potatoes with t	Lo	Mixed Dishes - Meat, Poultry, Seafood

27235000	Meat loaf made with venison/deer	Lo	Mixed Dishes - Meat, Poultry, Seafood
27236000	Venison or deer and noodles with c	Lo	Mixed Dishes - Meat, Poultry, Seafood
27241010	Chicken or turkey and potatoes with	Lo	Mixed Dishes - Meat, Poultry, Seafood
27242000	Chicken or turkey and noodles, no s	Lo	Mixed Dishes - Meat, Poultry, Seafood
27242200	Chicken or turkey and noodles with	Lo	Mixed Dishes - Meat, Poultry, Seafood
27242250	Chicken or turkey and noodles with	Lo	Mixed Dishes - Meat, Poultry, Seafood
27242300	Chicken or turkey and noodles with	Lo	Mixed Dishes - Meat, Poultry, Seafood
27242310	Chicken or turkey and noodles with	Lo	Mixed Dishes - Meat, Poultry, Seafood
27242350	Chicken or turkey tetrazzini	Lo	Mixed Dishes - Meat, Poultry, Seafood
27242400	Chicken or turkey and noodles with	Lo	Mixed Dishes - Meat, Poultry, Seafood
27242500	Chicken or turkey and noodles with	Lo	Mixed Dishes - Asian
27243000	Chicken or turkey and rice, no sauc	Lo	Mixed Dishes - Meat, Poultry, Seafood
27243100	Biryani with chicken	Lo	Mixed Dishes - Grain-based
27243300	Chicken or turkey and rice with crea	Lo	Mixed Dishes - Meat, Poultry, Seafood
27243400	Chicken or turkey and rice with mus	Lo	Mixed Dishes - Meat, Poultry, Seafood
27243500	Chicken or turkey and rice with tom	Lo	Mixed Dishes - Meat, Poultry, Seafood
27243600	Chicken or turkey and rice with soy-	Lo	Mixed Dishes - Asian
27246100	Chicken or turkey with dumplings	Lo	Mixed Dishes - Meat, Poultry, Seafood
27246200	Chicken or turkey with stuffing	Lo	Mixed Dishes - Meat, Poultry, Seafood
27246300	Chicken or turkey cake, patty, or cro	Lo	Mixed Dishes - Meat, Poultry, Seafood
27246400	Chicken or turkey souffle	Lo	Mixed Dishes - Meat, Poultry, Seafood
27246500	Meat loaf made with chicken or turk	Lo	Mixed Dishes - Meat, Poultry, Seafood
27246505	Meat loaf made with chicken or turk	Lo	Mixed Dishes - Meat, Poultry, Seafood
27250020	Clams, stuffed	Lo	Mixed Dishes - Meat, Poultry, Seafood
27250030	Codfish ball or cake	Lo	Mixed Dishes - Meat, Poultry, Seafood
27250040	Crab cake	Lo	Mixed Dishes - Meat, Poultry, Seafood
27250050	Fish cake or patty, NS as to fish	Lo	Mixed Dishes - Meat, Poultry, Seafood
27250060	Gefilte fish	Lo	Mixed Dishes - Meat, Poultry, Seafood
27250070	Salmon cake or patty	Lo	Mixed Dishes - Meat, Poultry, Seafood
27250080	Salmon loaf	Lo	Mixed Dishes - Meat, Poultry, Seafood
27250110	Scallops and noodles with cheese s	Lo	Mixed Dishes - Meat, Poultry, Seafood
27250120	Shrimp and noodles, no sauce	Lo	Mixed Dishes - Meat, Poultry, Seafood
27250122	Shrimp and noodles with gravy	Lo	Mixed Dishes - Meat, Poultry, Seafood
27250124	Shrimp and noodles with mushroom	Lo	Mixed Dishes - Meat, Poultry, Seafood
27250126	Shrimp and noodles with cream or w	Lo	Mixed Dishes - Meat, Poultry, Seafood
27250128	Shrimp and noodles with soy-based	Lo	Mixed Dishes - Asian
27250130	Shrimp and noodles with cheese sa	Lo	Mixed Dishes - Meat, Poultry, Seafood
27250132	Shrimp and noodles with tomato sa	Lo	Mixed Dishes - Meat, Poultry, Seafood
27250150	Tuna loaf	Lo	Mixed Dishes - Meat, Poultry, Seafood
27250160	Tuna cake or patty	Lo	Mixed Dishes - Meat, Poultry, Seafood
27250210	Clam cake or patty	Lo	Mixed Dishes - Meat, Poultry, Seafood
27250220	Oyster fritter	Lo	Mixed Dishes - Meat, Poultry, Seafood
27250250	Flounder with crab stuffing	Lo	Mixed Dishes - Meat, Poultry, Seafood
27250260	Lobster with bread stuffing, baked	Lo	Mixed Dishes - Meat, Poultry, Seafood
27250300	Mackerel cake or patty	Lo	Mixed Dishes - Meat, Poultry, Seafood
27250400	Shrimp cake or patty	Lo	Mixed Dishes - Meat, Poultry, Seafood
27250410	Shrimp with crab stuffing	Lo	Mixed Dishes - Meat, Poultry, Seafood
27250450	Shrimp toast, fried	Lo	Mixed Dishes - Meat, Poultry, Seafood
27250510	Fish cake (Kamaboko) tempura	Lo	Mixed Dishes - Meat, Poultry, Seafood
27250520	Seafood restructured	Lo	Seafood
27250610	Tuna noodle casserole with cream o	Lo	Mixed Dishes - Meat, Poultry, Seafood

27250630	Tuna noodle casserole with mushro	Lo	Mixed Dishes - Meat, Poultry, Seafood
27250710	Tuna and rice with mushroom sauc	Lo	Mixed Dishes - Meat, Poultry, Seafood
27250810	Fish and rice with tomato-based sa	Lo	Mixed Dishes - Meat, Poultry, Seafood
27250820	Fish and rice with cream sauce	Lo	Mixed Dishes - Meat, Poultry, Seafood
27250830	Fish and rice with mushroom sauce	Lo	Mixed Dishes - Meat, Poultry, Seafood
27250900	Fish and noodles with mushroom sa	Lo	Mixed Dishes - Meat, Poultry, Seafood
27250950	Shellfish and noodles with tomato-b	Lo	Mixed Dishes - Meat, Poultry, Seafood
27260010	Meat loaf, NS as to type of meat	Lo	Mixed Dishes - Meat, Poultry, Seafood
27260050	Meatballs, with breading, NS as to t	Lo	Mixed Dishes - Meat, Poultry, Seafood
27260080	Meat loaf made with beef and pork	Lo	Mixed Dishes - Meat, Poultry, Seafood
27260090	Meat loaf made with beef, veal and	Lo	Mixed Dishes - Meat, Poultry, Seafood
27260100	Meat loaf made with beef and pork,	Lo	Mixed Dishes - Meat, Poultry, Seafood
27260110	Hash, NS as to type of meat	Lo	Mixed Dishes - Meat, Poultry, Seafood
27260500	Vienna sausages stewed with potat	Lo	Mixed Dishes - Meat, Poultry, Seafood
27260510	Liver dumpling	Lo	Mixed Dishes - Meat, Poultry, Seafood
27261500	Stewed, seasoned, ground beef an	Lo	Mixed Dishes - Meat, Poultry, Seafood
27311110	Beef, potatoes, and vegetables incl	Lo	Mixed Dishes - Meat, Poultry, Seafood
27311120	Beef, potatoes, and vegetables, exc	Lo	Mixed Dishes - Meat, Poultry, Seafood
27311210	Corned beef, potatoes, and vegetab	Lo	Mixed Dishes - Meat, Poultry, Seafood
27311220	Corned beef, potatoes, and vegetab	Lo	Mixed Dishes - Meat, Poultry, Seafood
27311310	Beef stew with potatoes and vegeta	Lo	Mixed Dishes - Meat, Poultry, Seafood
27311320	Beef stew with potatoes and vegeta	Lo	Mixed Dishes - Meat, Poultry, Seafood
27311410	Beef stew with potatoes and vegeta	Lo	Mixed Dishes - Meat, Poultry, Seafood
27311420	Beef stew with potatoes and vegeta	Lo	Mixed Dishes - Meat, Poultry, Seafood
27311510	Shepherd's pie with beef	Lo	Mixed Dishes - Meat, Poultry, Seafood
27311600	Beef, potatoes, and vegetables incl	Lo	Mixed Dishes - Meat, Poultry, Seafood
27311605	Beef, potatoes, and vegetables exc	Lo	Mixed Dishes - Meat, Poultry, Seafood
27311610	Beef, potatoes, and vegetables incl	Lo	Mixed Dishes - Meat, Poultry, Seafood
27311620	Beef, potatoes, and vegetables exc	Lo	Mixed Dishes - Meat, Poultry, Seafood
27311625	Beef, potatoes, and vegetables incl	Lo	Mixed Dishes - Meat, Poultry, Seafood
27311630	Beef, potatoes, and vegetables exc	Lo	Mixed Dishes - Meat, Poultry, Seafood
27311635	Beef, potatoes, and vegetables incl	Lo	Mixed Dishes - Meat, Poultry, Seafood
27311640	Beef, potatoes, and vegetables exc	Lo	Mixed Dishes - Meat, Poultry, Seafood
27311645	Beef, potatoes, and vegetables incl	Lo	Mixed Dishes - Asian
27311650	Beef, potatoes, and vegetables exc	Lo	Mixed Dishes - Asian
27313010	Beef, noodles, and vegetables inclu	Lo	Mixed Dishes - Meat, Poultry, Seafood
27313020	Beef, noodles, and vegetables exclu	Lo	Mixed Dishes - Meat, Poultry, Seafood
27313110	Beef chow mein or chop suey with n	Lo	Mixed Dishes - Asian
27313150	Beef, noodles, and vegetables inclu	Lo	Mixed Dishes - Asian
27313160	Beef, noodles, and vegetables exclu	Lo	Mixed Dishes - Asian
27313210	Beef, noodles, and vegetables inclu	Lo	Mixed Dishes - Meat, Poultry, Seafood
27313220	Beef, noodles, and vegetables exclu	Lo	Mixed Dishes - Meat, Poultry, Seafood
27313310	Beef, noodles, and vegetables inclu	Lo	Mixed Dishes - Meat, Poultry, Seafood
27313320	Beef, noodles, and vegetables exclu	Lo	Mixed Dishes - Meat, Poultry, Seafood
27313410	Beef, noodles, and vegetables inclu	Lo	Mixed Dishes - Meat, Poultry, Seafood
27313420	Beef, noodles, and vegetables exclu	Lo	Mixed Dishes - Meat, Poultry, Seafood
27315010	Beef, rice, and vegetables including	Lo	Mixed Dishes - Meat, Poultry, Seafood
27315020	Beef, rice, and vegetables excludin	Lo	Mixed Dishes - Meat, Poultry, Seafood
27315210	Beef, rice, and vegetables including	Lo	Mixed Dishes - Meat, Poultry, Seafood
27315220	Beef, rice, and vegetables excludin	Lo	Mixed Dishes - Meat, Poultry, Seafood
27315250	Stuffed cabbage rolls with beef and	Lo	Mixed Dishes - Meat, Poultry, Seafood

27315270	Stuffed grape leaves with beef and	Lo	Mixed Dishes - Meat, Poultry, Seafood
27315310	Beef, rice, and vegetables including	Lo	Mixed Dishes - Meat, Poultry, Seafood
27315320	Beef, rice, and vegetables excluding	Lo	Mixed Dishes - Meat, Poultry, Seafood
27315340	Beef, rice, and vegetables excluding	Lo	Mixed Dishes - Meat, Poultry, Seafood
27315410	Beef, rice, and vegetables including	Lo	Mixed Dishes - Meat, Poultry, Seafood
27315420	Beef, rice, and vegetables excluding	Lo	Mixed Dishes - Meat, Poultry, Seafood
27315510	Beef, rice, and vegetables including	Lo	Mixed Dishes - Asian
27315520	Beef, rice, and vegetables excluding	Lo	Mixed Dishes - Asian
27317010	Beef pot pie	Lo	Mixed Dishes - Meat, Poultry, Seafood
27319010	Stuffed green pepper, Puerto Rican	Lo	Mixed Dishes - Meat, Poultry, Seafood
27320020	Ham pot pie	Lo	Mixed Dishes - Meat, Poultry, Seafood
27320025	Ham or pork, noodles and vegetabl	Lo	Mixed Dishes - Meat, Poultry, Seafood
27320027	Ham or pork, noodles, and vegetab	Lo	Mixed Dishes - Meat, Poultry, Seafood
27320030	Ham or pork, noodles and vegetabl	Lo	Mixed Dishes - Meat, Poultry, Seafood
27320040	Pork, potatoes, and vegetables incl	Lo	Mixed Dishes - Meat, Poultry, Seafood
27320070	Ham or pork, noodles, and vegetab	Lo	Mixed Dishes - Meat, Poultry, Seafood
27320080	Sausage, noodles, and vegetables	Lo	Mixed Dishes - Meat, Poultry, Seafood
27320090	Sausage, noodles, and vegetables	Lo	Mixed Dishes - Meat, Poultry, Seafood
27320100	Pork, potatoes, and vegetables incl	Lo	Mixed Dishes - Meat, Poultry, Seafood
27320110	Pork, potatoes, and vegetables exc	Lo	Mixed Dishes - Meat, Poultry, Seafood
27320120	Sausage, potatoes, and vegetables	Lo	Mixed Dishes - Meat, Poultry, Seafood
27320130	Sausage, potatoes, and vegetables	Lo	Mixed Dishes - Meat, Poultry, Seafood
27320140	Pork, potatoes, and vegetables incl	Lo	Mixed Dishes - Meat, Poultry, Seafood
27320150	Pork, potatoes, and vegetables exc	Lo	Mixed Dishes - Meat, Poultry, Seafood
27320210	Pork, potatoes, and vegetables exc	Lo	Mixed Dishes - Meat, Poultry, Seafood
27320310	Pork chow mein or chop suey with r	Lo	Mixed Dishes - Asian
27320320	Pork, rice, and vegetables including	Lo	Mixed Dishes - Asian
27320330	Pork, rice, and vegetables excluding	Lo	Mixed Dishes - Asian
27320340	Pork, rice, and vegetables including	Lo	Mixed Dishes - Meat, Poultry, Seafood
27320350	Pork, rice, and vegetables excluding	Lo	Mixed Dishes - Meat, Poultry, Seafood
27320410	Ham, potatoes, and vegetables exc	Lo	Mixed Dishes - Meat, Poultry, Seafood
27320450	Ham, potatoes, and vegetables incl	Lo	Mixed Dishes - Meat, Poultry, Seafood
27320500	Sweet and sour pork with rice	Lo	Mixed Dishes - Asian
27330010	Shepherd's pie with lamb	Lo	Mixed Dishes - Meat, Poultry, Seafood
27330030	Lamb or mutton stew with potatoes	Lo	Mixed Dishes - Meat, Poultry, Seafood
27330050	Lamb or mutton, rice, and vegetable	Lo	Mixed Dishes - Meat, Poultry, Seafood
27330060	Lamb or mutton, rice, and vegetable	Lo	Mixed Dishes - Meat, Poultry, Seafood
27330080	Lamb or mutton, rice, and vegetable	Lo	Mixed Dishes - Meat, Poultry, Seafood
27330110	Lamb or mutton stew with potatoes	Lo	Mixed Dishes - Meat, Poultry, Seafood
27330170	Stuffed grape leaves with lamb and	Lo	Mixed Dishes - Meat, Poultry, Seafood
27330210	Lamb or mutton stew with potatoes	Lo	Mixed Dishes - Meat, Poultry, Seafood
27330220	Lamb or mutton stew with potatoes	Lo	Mixed Dishes - Meat, Poultry, Seafood
27332100	Veal stew with potatoes and vegeta	Lo	Mixed Dishes - Meat, Poultry, Seafood
27335100	Rabbit stew with potatoes and vege	Lo	Mixed Dishes - Meat, Poultry, Seafood
27336100	Venison or deer stew with potatoes	Lo	Mixed Dishes - Meat, Poultry, Seafood
27336150	Venison or deer stew with potatoes	Lo	Mixed Dishes - Meat, Poultry, Seafood
27336200	Venison or deer, potatoes, and veg	Lo	Mixed Dishes - Meat, Poultry, Seafood
27336250	Venison or deer, potatoes, and veg	Lo	Mixed Dishes - Meat, Poultry, Seafood
27336310	Venison or deer, noodles, and vege	Lo	Mixed Dishes - Meat, Poultry, Seafood
27341000	Chicken or turkey, potatoes, corn, a	Lo	Mixed Dishes - Meat, Poultry, Seafood
27341010	Chicken or turkey, potatoes, and ve	Lo	Mixed Dishes - Meat, Poultry, Seafood

27341020	Chicken or turkey, potatoes, and ve	Lo	Mixed Dishes - Meat, Poultry, Seafood
27341025	Chicken or turkey, potatoes, and ve	Lo	Mixed Dishes - Meat, Poultry, Seafood
27341030	Chicken or turkey, potatoes, and ve	Lo	Mixed Dishes - Meat, Poultry, Seafood
27341035	Chicken or turkey, potatoes, and ve	Lo	Mixed Dishes - Meat, Poultry, Seafood
27341040	Chicken or turkey, potatoes, and ve	Lo	Mixed Dishes - Meat, Poultry, Seafood
27341045	Chicken or turkey, potatoes, and ve	Lo	Mixed Dishes - Meat, Poultry, Seafood
27341050	Chicken or turkey, potatoes, and ve	Lo	Mixed Dishes - Meat, Poultry, Seafood
27341055	Chicken or turkey, potatoes, and ve	Lo	Mixed Dishes - Meat, Poultry, Seafood
27341060	Chicken or turkey, potatoes, and ve	Lo	Mixed Dishes - Meat, Poultry, Seafood
27341310	Chicken or turkey stew with potatoe	Lo	Mixed Dishes - Meat, Poultry, Seafood
27341320	Chicken or turkey stew with potatoe	Lo	Mixed Dishes - Meat, Poultry, Seafood
27341510	Chicken or turkey stew with potatoe	Lo	Mixed Dishes - Meat, Poultry, Seafood
27341520	Chicken or turkey stew with potatoe	Lo	Mixed Dishes - Meat, Poultry, Seafood
27343010	Chicken or turkey, noodles, and veg	Lo	Mixed Dishes - Meat, Poultry, Seafood
27343020	Chicken or turkey, noodles, and veg	Lo	Mixed Dishes - Meat, Poultry, Seafood
27343410	Chicken or turkey, noodles, and veg	Lo	Mixed Dishes - Meat, Poultry, Seafood
27343420	Chicken or turkey, noodles, and veg	Lo	Mixed Dishes - Meat, Poultry, Seafood
27343470	Chicken or turkey, noodles, and veg	Lo	Mixed Dishes - Meat, Poultry, Seafood
27343480	Chicken or turkey, noodles, and veg	Lo	Mixed Dishes - Meat, Poultry, Seafood
27343510	Chicken or turkey, noodles, and veg	Lo	Mixed Dishes - Meat, Poultry, Seafood
27343520	Chicken or turkey, noodles, and veg	Lo	Mixed Dishes - Meat, Poultry, Seafood
27343910	Chicken or turkey chow mein or cho	Lo	Mixed Dishes - Asian
27343950	Chicken or turkey, noodles, and veg	Lo	Mixed Dishes - Meat, Poultry, Seafood
27343960	Chicken or turkey, noodles, and veg	Lo	Mixed Dishes - Meat, Poultry, Seafood
27345010	Chicken or turkey, rice, and vegetab	Lo	Mixed Dishes - Meat, Poultry, Seafood
27345020	Chicken or turkey, rice, and vegetab	Lo	Mixed Dishes - Meat, Poultry, Seafood
27345210	Chicken or turkey, rice, and vegetab	Lo	Mixed Dishes - Meat, Poultry, Seafood
27345220	Chicken or turkey, rice, and vegetab	Lo	Mixed Dishes - Meat, Poultry, Seafood
27345230	Chicken or turkey, rice, corn, and c	Lo	Mixed Dishes - Meat, Poultry, Seafood
27345310	Chicken or turkey, rice, and vegetab	Lo	Mixed Dishes - Asian
27345320	Chicken or turkey, rice, and vegetab	Lo	Mixed Dishes - Asian
27345410	Chicken or turkey, rice, and vegetab	Lo	Mixed Dishes - Meat, Poultry, Seafood
27345420	Chicken or turkey, rice, and vegetab	Lo	Mixed Dishes - Meat, Poultry, Seafood
27345440	Chicken or turkey, rice, and vegetab	Lo	Mixed Dishes - Meat, Poultry, Seafood
27345450	Chicken or turkey, rice, and vegetab	Lo	Mixed Dishes - Meat, Poultry, Seafood
27345510	Chicken or turkey, rice, and vegetab	Lo	Mixed Dishes - Meat, Poultry, Seafood
27345520	Chicken or turkey, rice, and vegetab	Lo	Mixed Dishes - Meat, Poultry, Seafood
27347100	Chicken or turkey pot pie	Lo	Mixed Dishes - Meat, Poultry, Seafood
27347200	Chicken or turkey, stuffing, and veg	Lo	Mixed Dishes - Meat, Poultry, Seafood
27347210	Chicken or turkey,stuffing, and vege	Lo	Mixed Dishes - Meat, Poultry, Seafood
27347220	Chicken or turkey, stuffing, and veg	Lo	Mixed Dishes - Meat, Poultry, Seafood
27347230	Chicken or turkey, stuffing, and veg	Lo	Mixed Dishes - Meat, Poultry, Seafood
27347240	Chicken or turkey, dumplings, and v	Lo	Mixed Dishes - Meat, Poultry, Seafood
27347250	Chicken or turkey, dumplings, and v	Lo	Mixed Dishes - Meat, Poultry, Seafood
27348100	Chicken fricassee, Puerto Rican sty	Lo	Mixed Dishes - Meat, Poultry, Seafood
27350020	Paella with seafood	Lo	Mixed Dishes - Grain-based
27350030	Seafood stew with potatoes and veg	Lo	Mixed Dishes - Meat, Poultry, Seafood
27350050	Shrimp chow mein or chop suey wit	Lo	Mixed Dishes - Asian
27350060	Shrimp creole, with rice	Lo	Mixed Dishes - Meat, Poultry, Seafood
27350070	Tuna pot pie	Lo	Mixed Dishes - Meat, Poultry, Seafood
27350080	Tuna noodle casserole with vegetab	Lo	Mixed Dishes - Meat, Poultry, Seafood

27350090	Fish, noodles, and vegetables inclu	Lo	Mixed Dishes - Meat, Poultry, Seafood
27350100	Fish, noodles, and vegetables exclu	Lo	Mixed Dishes - Meat, Poultry, Seafood
27350110	Bouillabaisse	Lo	Mixed Dishes - Meat, Poultry, Seafood
27350310	Seafood stew with potatoes and veg	Lo	Mixed Dishes - Meat, Poultry, Seafood
27350410	Tuna noodle casserole with vegetab	Lo	Mixed Dishes - Meat, Poultry, Seafood
27351030	Stewed codfish, Puerto Rican style	Lo	Mixed Dishes - Meat, Poultry, Seafood
27351040	Biscayne codfish, Puerto Rican styl	Lo	Mixed Dishes - Meat, Poultry, Seafood
27360000	Stew, NFS	Lo	Mixed Dishes - Meat, Poultry, Seafood
27360010	Goulash, NFS	Lo	Mixed Dishes - Meat, Poultry, Seafood
27360050	Meat pie, NFS	Lo	Mixed Dishes - Meat, Poultry, Seafood
27360080	Chow mein or chop suey, NS as to	Lo	Mixed Dishes - Asian
27360090	Paella, NFS	Lo	Mixed Dishes - Grain-based
27360100	Brunswick stew	Lo	Mixed Dishes - Meat, Poultry, Seafood
27360120	Chow mein or chop suey, various ty	Lo	Mixed Dishes - Asian
27361010	Stewed variety meats, mostly liver,	Lo	Mixed Dishes - Meat, Poultry, Seafood
27362000	Stewed tripe, with potatoes, Puerto	Lo	Mixed Dishes - Meat, Poultry, Seafood
27363000	Gumbo with rice	Lo	Mixed Dishes - Meat, Poultry, Seafood
27363100	Jambalaya with meat and rice	Lo	Mixed Dishes - Grain-based
27410210	Beef and vegetables including carro	Lo	Mixed Dishes - Meat, Poultry, Seafood
27410220	Beef and vegetables excluding carr	Lo	Mixed Dishes - Meat, Poultry, Seafood
27410250	Beef shish kabob with vegetables, e	Lo	Mixed Dishes - Meat, Poultry, Seafood
27411100	Beef with vegetables including carro	Lo	Mixed Dishes - Meat, Poultry, Seafood
27411120	Swiss steak	Lo	Mixed Dishes - Meat, Poultry, Seafood
27411150	Beef rolls, stuffed with vegetables o	Lo	Mixed Dishes - Meat, Poultry, Seafood
27411200	Beef with vegetables excluding carr	Lo	Mixed Dishes - Meat, Poultry, Seafood
27414100	Beef with vegetables including carro	Lo	Mixed Dishes - Meat, Poultry, Seafood
27414200	Beef with vegetables excluding carr	Lo	Mixed Dishes - Meat, Poultry, Seafood
27415100	Beef and vegetables including carro	Lo	Mixed Dishes - Asian
27415110	Beef and broccoli	Lo	Mixed Dishes - Asian
27415120	Beef, tofu, and vegetables including	Lo	Mixed Dishes - Asian
27415140	Hunan beef	Lo	Mixed Dishes - Asian
27415150	Beef chow mein or chop suey, no n	Lo	Mixed Dishes - Asian
27415170	Kung Pao beef	Lo	Mixed Dishes - Asian
27415200	Beef and vegetables excluding carr	Lo	Mixed Dishes - Asian
27415220	Beef, tofu, and vegetables excludin	Lo	Mixed Dishes - Asian
27416150	Pepper steak	Lo	Mixed Dishes - Meat, Poultry, Seafood
27416200	Beef, ground, with egg and onion	Lo	Mixed Dishes - Meat, Poultry, Seafood
27416250	Beef salad	Lo	Mixed Dishes - Meat, Poultry, Seafood
27416300	Beef taco filling: beef, cheese, toma	Lo	Mixed Dishes - Mexican
27416400	Stir fried beef and vegetables in soy	Lo	Mixed Dishes - Asian
27416450	Beef and vegetables including carro	Lo	Mixed Dishes - Meat, Poultry, Seafood
27416500	Beef and vegetables excluding carr	Lo	Mixed Dishes - Meat, Poultry, Seafood
27418110	Seasoned shredded soup meat	Lo	Mixed Dishes - Meat, Poultry, Seafood
27418210	Beef stew with vegetables excluding	Lo	Mixed Dishes - Meat, Poultry, Seafood
27418310	Corned beef with tomato sauce and	Lo	Mixed Dishes - Meat, Poultry, Seafood
27418410	Beef steak with onions, Puerto Rica	Lo	Mixed Dishes - Meat, Poultry, Seafood
27420010	Cabbage with ham hocks	Lo	Mixed Dishes - Meat, Poultry, Seafood
27420020	Ham or pork salad	Lo	Mixed Dishes - Meat, Poultry, Seafood
27420040	Frankfurters or hot dogs and sauerk	Lo	Mixed Dishes - Meat, Poultry, Seafood
27420060	Pork and vegetables including carro	Lo	Mixed Dishes - Meat, Poultry, Seafood
27420080	Greens with ham or pork	Lo	Vegetables, excluding Potatoes

27420100	Pork, tofu, and vegetables including	Lo	Mixed Dishes - Asian
27420110	Pork and vegetables, Hawaiian style	Lo	Mixed Dishes - Asian
27420150	Kung Pao pork	Lo	Mixed Dishes - Asian
27420160	Moo Shu pork, without Chinese pan	Lo	Mixed Dishes - Asian
27420170	Pork and onions with soy-based sau	Lo	Mixed Dishes - Asian
27420200	Pork hash	Lo	Mixed Dishes - Meat, Poultry, Seafood
27420250	Ham and vegetables including carro	Lo	Mixed Dishes - Meat, Poultry, Seafood
27420270	Ham and vegetables excluding carr	Lo	Mixed Dishes - Meat, Poultry, Seafood
27420350	Pork and vegetables excluding carr	Lo	Mixed Dishes - Meat, Poultry, Seafood
27420370	Pork, tofu, and vegetables, excludin	Lo	Mixed Dishes - Asian
27420390	Pork chow mein or chop suey, no n	Lo	Mixed Dishes - Asian
27420400	Pork and vegetables including carro	Lo	Mixed Dishes - Meat, Poultry, Seafood
27420410	Pork and vegetables excluding car	Lo	Mixed Dishes - Meat, Poultry, Seafood
27420450	Sausage and vegetables including	Lo	Mixed Dishes - Meat, Poultry, Seafood
27420460	Sausage and vegetables, excluding	Lo	Mixed Dishes - Meat, Poultry, Seafood
27420470	Sausage and peppers, no sauce	Lo	Mixed Dishes - Meat, Poultry, Seafood
27420500	Pork and vegetables including carro	Lo	Mixed Dishes - Asian
27420510	Pork and vegetables excluding carr	Lo	Mixed Dishes - Asian
27420520	Pork shish kabob with vegetables, e	Lo	Mixed Dishes - Meat, Poultry, Seafood
27421010	Stuffed christophine, Puerto Rican s	Lo	Mixed Dishes - Meat, Poultry, Seafood
27422010	Pork chop stewed with vegetables,	Lo	Mixed Dishes - Meat, Poultry, Seafood
27430400	Lamb or mutton stew with vegetable	Lo	Mixed Dishes - Meat, Poultry, Seafood
27430410	Lamb or mutton stew with vegetable	Lo	Mixed Dishes - Meat, Poultry, Seafood
27430500	Veal goulash with vegetables exclu	Lo	Mixed Dishes - Meat, Poultry, Seafood
27430610	Lamb shish kabob with vegetables,	Lo	Mixed Dishes - Meat, Poultry, Seafood
27440110	Chicken or turkey and vegetables in	Lo	Mixed Dishes - Meat, Poultry, Seafood
27440120	Chicken or turkey and vegetables e	Lo	Mixed Dishes - Meat, Poultry, Seafood
27440130	Chicken or turkey shish kabob with	Lo	Mixed Dishes - Meat, Poultry, Seafood
27442110	Chicken or turkey and vegetables in	Lo	Mixed Dishes - Meat, Poultry, Seafood
27442120	Chicken or turkey and vegetables e	Lo	Mixed Dishes - Meat, Poultry, Seafood
27443110	Chicken or turkey a la king with veg	Lo	Mixed Dishes - Meat, Poultry, Seafood
27443120	Chicken or turkey a la king with veg	Lo	Mixed Dishes - Meat, Poultry, Seafood
27443150	Chicken or turkey divan	Lo	Mixed Dishes - Meat, Poultry, Seafood
27445110	Chicken or turkey and vegetables in	Lo	Mixed Dishes - Asian
27445120	Chicken or turkey and vegetables e	Lo	Mixed Dishes - Asian
27445125	Chicken or turkey and vegetables in	Lo	Mixed Dishes - Meat, Poultry, Seafood
27445130	Chicken or turkey and vegetables e	Lo	Mixed Dishes - Meat, Poultry, Seafood
27445150	General Tso chicken	Lo	Mixed Dishes - Asian
27445180	Moo Goo Gai Pan	Lo	Mixed Dishes - Asian
27445220	Kung pao chicken	Lo	Mixed Dishes - Asian
27445250	Almond chicken	Lo	Mixed Dishes - Asian
27446100	Chicken or turkey chow mein or cho	Lo	Mixed Dishes - Asian
27446200	Chicken or turkey salad, made with	Lo	Mixed Dishes - Meat, Poultry, Seafood
27446205	Chicken or turkey salad with nuts ar	Lo	Mixed Dishes - Meat, Poultry, Seafood
27446220	Chicken or turkey salad with egg	Lo	Mixed Dishes - Meat, Poultry, Seafood
27446225	Chicken or turkey salad, made with	Lo	Mixed Dishes - Meat, Poultry, Seafood
27446230	Chicken or turkey salad, made with	Lo	Mixed Dishes - Meat, Poultry, Seafood
27446235	Chicken or turkey salad, made with	Lo	Mixed Dishes - Meat, Poultry, Seafood
27446240	Chicken or turkey salad, made with	Lo	Mixed Dishes - Meat, Poultry, Seafood
27446245	Chicken or turkey salad, made with	Lo	Mixed Dishes - Meat, Poultry, Seafood
27446260	Chicken or turkey salad, made with	Lo	Mixed Dishes - Meat, Poultry, Seafood

27446400	Chicken or turkey and vegetables ir	Lo	Mixed Dishes - Meat, Poultry, Seafood
27446410	Chicken or turkey and vegetables e	Lo	Mixed Dishes - Meat, Poultry, Seafood
27448020	Chicken or turkey fricassee, with sa	Lo	Mixed Dishes - Meat, Poultry, Seafood
27450010	Crab salad	Lo	Mixed Dishes - Meat, Poultry, Seafood
27450020	Lobster salad	Lo	Mixed Dishes - Meat, Poultry, Seafood
27450040	Shrimp chow mein or chop suey, nc	Lo	Mixed Dishes - Asian
27450060	Tuna salad, made with mayonnaise	Lo	Mixed Dishes - Meat, Poultry, Seafood
27450061	Tuna salad, made with light mayonr	Lo	Mixed Dishes - Meat, Poultry, Seafood
27450062	Tuna salad, made with mayonnaise	Lo	Mixed Dishes - Meat, Poultry, Seafood
27450063	Tuna salad, made with light mayonr	Lo	Mixed Dishes - Meat, Poultry, Seafood
27450064	Tuna salad, made with creamy dres	Lo	Mixed Dishes - Meat, Poultry, Seafood
27450065	Tuna salad, made with light creamy	Lo	Mixed Dishes - Meat, Poultry, Seafood
27450066	Tuna salad, made with Italian dress	Lo	Mixed Dishes - Meat, Poultry, Seafood
27450067	Tuna salad, made with light Italian d	Lo	Mixed Dishes - Meat, Poultry, Seafood
27450068	Tuna salad, made with any type of f	Lo	Mixed Dishes - Meat, Poultry, Seafood
27450070	Shrimp salad	Lo	Mixed Dishes - Meat, Poultry, Seafood
27450080	Seafood salad	Lo	Mixed Dishes - Meat, Poultry, Seafood
27450130	Crab salad made with imitation crab	Lo	Mixed Dishes - Meat, Poultry, Seafood
27450150	Fish, tofu, and vegetables, tempura	Lo	Mixed Dishes - Meat, Poultry, Seafood
27450250	Oysters Rockefeller	Lo	Mixed Dishes - Meat, Poultry, Seafood
27450400	Shrimp and vegetables including ca	Lo	Mixed Dishes - Meat, Poultry, Seafood
27450405	Shrimp and vegetables excluding ca	Lo	Mixed Dishes - Meat, Poultry, Seafood
27450410	Shrimp and vegetables including ca	Lo	Mixed Dishes - Asian
27450420	Shrimp and vegetables excluding ca	Lo	Mixed Dishes - Asian
27450430	Shrimp shish kabob with vegetables	Lo	Mixed Dishes - Meat, Poultry, Seafood
27450450	Shrimp creole, no rice	Lo	Mixed Dishes - Meat, Poultry, Seafood
27450470	Kung Pao shrimp	Lo	Mixed Dishes - Asian
27450510	Tuna casserole with vegetables and	Lo	Mixed Dishes - Meat, Poultry, Seafood
27450600	Shellfish mixture and vegetables in	Lo	Mixed Dishes - Asian
27450610	Shellfish mixture and vegetables ex	Lo	Mixed Dishes - Asian
27450650	Shellfish mixture and vegetables in	Lo	Mixed Dishes - Meat, Poultry, Seafood
27450660	Shellfish mixture and vegetables ex	Lo	Mixed Dishes - Meat, Poultry, Seafood
27450700	Fish and vegetables including carro	Lo	Mixed Dishes - Meat, Poultry, Seafood
27450710	Fish and vegetables excluding carr	Lo	Mixed Dishes - Meat, Poultry, Seafood
27450740	Fish and vegetables including carro	Lo	Mixed Dishes - Asian
27450750	Fish and vegetables excluding carr	Lo	Mixed Dishes - Asian
27451030	Lobster with sauce, Puerto Rican st	Lo	Mixed Dishes - Meat, Poultry, Seafood
27460010	Chow mein or chop suey, NS as to	Lo	Mixed Dishes - Asian
27460510	Antipasto with ham, fish, cheese, ve	Lo	Mixed Dishes - Meat, Poultry, Seafood
27460710	Livers, chicken, chopped, with eggs	Lo	Mixed Dishes - Meat, Poultry, Seafood
27460750	Liver, beef or calves, and onions	Lo	Mixed Dishes - Meat, Poultry, Seafood
27461010	Stewed seasoned ground beef, Pue	Lo	Mixed Dishes - Meat, Poultry, Seafood
27464000	Gumbo, no rice	Lo	Mixed Dishes - Meat, Poultry, Seafood
27500050	Sandwich, NFS	Lo	Mixed Dishes - Sandwiches (single code)
27500100	Meat sandwich, NFS	Lo	Mixed Dishes - Sandwiches (single code)
27500200	Wrap sandwich, filled with meat, po	Lo	Mixed Dishes - Sandwiches (single code)
27500300	Wrap sandwich, NFS	Lo	Mixed Dishes - Sandwiches (single code)
27510000	Beef sandwich, NFS	Lo	Mixed Dishes - Sandwiches (single code)
27510110	Beef barbecue sandwich or Sloppy	Lo	Mixed Dishes - Sandwiches (single code)
27510130	Beef barbecue submarine sandwich	Lo	Mixed Dishes - Sandwiches (single code)
27510140	Cheeseburger slider, from fast food	Lo	Mixed Dishes - Sandwiches (single code)

27510145	Cheeseburger, 1 miniature patty, w	Lo	Mixed Dishes - Sandwiches (single code)
27510150	Cheeseburger, 1 miniature patty, or	Lo	Mixed Dishes - Sandwiches (single code)
27510155	Cheeseburger, NFS	Lo	Mixed Dishes - Sandwiches (single code)
27510160	Cheeseburger, from fast food, 1 sm	Lo	Mixed Dishes - Sandwiches (single code)
27510165	Cheeseburger, 1 small patty, with c	Lo	Mixed Dishes - Sandwiches (single code)
27510170	Cheeseburger (Burger King)	Lo	Mixed Dishes - Sandwiches (single code)
27510171	Whopper Jr with cheese (Burger Ki	Lo	Mixed Dishes - Sandwiches (single code)
27510172	Cheeseburger (McDonalds)	Lo	Mixed Dishes - Sandwiches (single code)
27510173	Cheeseburger, 1 small patty, with c	Lo	Mixed Dishes - Sandwiches (single code)
27510174	Cheeseburger, 1 small patty, with c	Lo	Mixed Dishes - Sandwiches (single code)
27510175	Cheeseburger, 1 small patty, with c	Lo	Mixed Dishes - Sandwiches (single code)
27510190	Cheeseburger, from school cafeteri	Lo	Mixed Dishes - Sandwiches (single code)
27510191	Cheeseburger slider	Lo	Mixed Dishes - Sandwiches (single code)
27510195	Cheeseburger, on white bun, 1 sma	Lo	Mixed Dishes - Sandwiches (single code)
27510196	Cheeseburger, on wheat bun, 1 sm	Lo	Mixed Dishes - Sandwiches (single code)
27510205	Cheeseburger, 1 small patty, with c	Lo	Mixed Dishes - Sandwiches (single code)
27510206	Cheeseburger, 1 small patty, with c	Lo	Mixed Dishes - Sandwiches (single code)
27510207	Cheeseburger, 1 small patty, with c	Lo	Mixed Dishes - Sandwiches (single code)
27510210	Cheeseburger, plain, on bun	Lo	Mixed Dishes - Sandwiches (single code)
27510215	Cheeseburger, from fast food, 1 me	Lo	Mixed Dishes - Sandwiches (single code)
27510220	Cheeseburger, with mayonnaise or	Lo	Mixed Dishes - Sandwiches (single code)
27510225	Cheeseburger, 1 medium patty, wit	Lo	Mixed Dishes - Sandwiches (single code)
27510229	Quarter Pounder (McDonalds)	Lo	Mixed Dishes - Sandwiches (single code)
27510230	Cheeseburger, with mayonnaise or	Lo	Mixed Dishes - Sandwiches (single code)
27510231	Whopper with cheese (Burger King	Lo	Mixed Dishes - Sandwiches (single code)
27510232	Quarter Pounder with cheese (McD	Lo	Mixed Dishes - Sandwiches (single code)
27510233	Cheeseburger, 1 medium patty, wit	Lo	Mixed Dishes - Sandwiches (single code)
27510235	Cheeseburger submarine sandwich	Lo	Mixed Dishes - Sandwiches (single code)
27510240	Cheeseburger, 1/4 lb meat, plain, o	Lo	Mixed Dishes - Sandwiches (single code)
27510241	Cheeseburger, on white bun, 1 med	Lo	Mixed Dishes - Sandwiches (single code)
27510242	Cheeseburger, on wheat bun, 1 me	Lo	Mixed Dishes - Sandwiches (single code)
27510243	Cheeseburger, 1 medium patty, pla	Lo	Mixed Dishes - Sandwiches (single code)
27510245	Cheeseburger, on white bun, 1 larg	Lo	Mixed Dishes - Sandwiches (single code)
27510246	Cheeseburger, on wheat bun, 1 larg	Lo	Mixed Dishes - Sandwiches (single code)
27510250	Cheeseburger, 1/4 lb meat, with ma	Lo	Mixed Dishes - Sandwiches (single code)
27510251	Cheeseburger, 1 medium patty, wit	Lo	Mixed Dishes - Sandwiches (single code)
27510252	Cheeseburger, 1 medium patty, wit	Lo	Mixed Dishes - Sandwiches (single code)
27510253	Cheeseburger, 1 medium patty, wit	Lo	Mixed Dishes - Sandwiches (single code)
27510254	Double cheeseburger, on white bun	Lo	Mixed Dishes - Sandwiches (single code)
27510257	Double cheeseburger, on white bun	Lo	Mixed Dishes - Sandwiches (single code)
27510258	Double cheeseburger, on wheat bu	Lo	Mixed Dishes - Sandwiches (single code)
27510260	Cheeseburger, 1/4 lb meat, with mu	Lo	Mixed Dishes - Sandwiches (single code)
27510261	Cheeseburger, from fast food, 1 lar	Lo	Mixed Dishes - Sandwiches (single code)
27510262	Double cheeseburger, on white bun	Lo	Mixed Dishes - Sandwiches (single code)
27510265	Double cheeseburger, (2 patties, 1	Lo	Mixed Dishes - Sandwiches (single code)
27510266	Cheeseburger, 1 large patty, with c	Lo	Mixed Dishes - Sandwiches (single code)
27510270	Double cheeseburger (2 patties), pl	Lo	Mixed Dishes - Sandwiches (single code)
27510276	Bacon cheeseburger, 1 small patty,	Lo	Mixed Dishes - Sandwiches (single code)
27510280	Double cheeseburger (2 patties), w	Lo	Mixed Dishes - Sandwiches (single code)
27510281	Bacon cheeseburger, 1 small patty,	Lo	Mixed Dishes - Sandwiches (single code)
27510290	Double cheeseburger (2 patties), pl	Lo	Mixed Dishes - Sandwiches (single code)

27510300	Double cheeseburger (2 patties), w	Lo	Mixed Dishes - Sandwiches (single code)
27510305	Bacon cheeseburger, 1 medium pa	Lo	Mixed Dishes - Sandwiches (single code)
27510310	Cheeseburger with tomato and/or c	Lo	Mixed Dishes - Sandwiches (single code)
27510311	Cheeseburger, 1 oz meat, plain, on	Lo	Mixed Dishes - Sandwiches (single code)
27510312	Bacon cheeseburger, 1 medium pa	Lo	Mixed Dishes - Sandwiches (single code)
27510320	Cheeseburger, 1/4 lb meat, with tor	Lo	Mixed Dishes - Sandwiches (single code)
27510330	Double cheeseburger (2 patties), w	Lo	Mixed Dishes - Sandwiches (single code)
27510331	Bacon cheeseburger, 1 medium pa	Lo	Mixed Dishes - Sandwiches (single code)
27510340	Double cheeseburger (2 patties), w	Lo	Mixed Dishes - Sandwiches (single code)
27510341	Bacon cheeseburger, 1 medium pa	Lo	Mixed Dishes - Sandwiches (single code)
27510342	Bacon cheeseburger, 1 medium pa	Lo	Mixed Dishes - Sandwiches (single code)
27510343	Bacon cheeseburger, 1 medium pa	Lo	Mixed Dishes - Sandwiches (single code)
27510346	Bacon cheeseburger, 1 large patty,	Lo	Mixed Dishes - Sandwiches (single code)
27510350	Cheeseburger, 1/4 lb meat, with ma	Lo	Mixed Dishes - Sandwiches (single code)
27510355	Cheeseburger, 1/3 lb meat, with ma	Lo	Mixed Dishes - Sandwiches (single code)
27510359	Cheeseburger, 1/3 lb meat, with ma	Lo	Mixed Dishes - Sandwiches (single code)
27510360	Bacon cheeseburger, with mayonna	Lo	Mixed Dishes - Sandwiches (single code)
27510370	Double cheeseburger (2 patties, 1/4	Lo	Mixed Dishes - Sandwiches (single code)
27510371	Double cheeseburger, from fast foo	Lo	Mixed Dishes - Sandwiches (single code)
27510375	Double cheeseburger (2 patties, 1/4	Lo	Mixed Dishes - Sandwiches (single code)
27510376	Double cheeseburger, 2 small patti	Lo	Mixed Dishes - Sandwiches (single code)
27510380	Triple cheeseburger (3 patties, 1/4	Lo	Mixed Dishes - Sandwiches (single code)
27510385	Double bacon cheeseburger (2 patt	Lo	Mixed Dishes - Sandwiches (single code)
27510386	Double cheeseburger (Burger King)	Lo	Mixed Dishes - Sandwiches (single code)
27510387	Double cheeseburger (McDonalds)	Lo	Mixed Dishes - Sandwiches (single code)
27510388	McDouble (McDonalds)	Lo	Mixed Dishes - Sandwiches (single code)
27510389	Big Mac (McDonalds)	Lo	Mixed Dishes - Sandwiches (single code)
27510390	Double bacon cheeseburger (2 patt	Lo	Mixed Dishes - Sandwiches (single code)
27510391	Double cheeseburger, 2 small patti	Lo	Mixed Dishes - Sandwiches (single code)
27510400	Bacon cheeseburger, 1/4 lb meat, v	Lo	Mixed Dishes - Sandwiches (single code)
27510401	Double cheeseburger, from fast foo	Lo	Mixed Dishes - Sandwiches (single code)
27510405	Double cheeseburger, from fast foo	Lo	Mixed Dishes - Sandwiches (single code)
27510406	Double cheeseburger, 2 medium pa	Lo	Mixed Dishes - Sandwiches (single code)
27510410	Chiliburger, on bun	Lo	Mixed Dishes - Sandwiches (single code)
27510412	Double cheeseburger, 2 medium pa	Lo	Mixed Dishes - Sandwiches (single code)
27510413	Double cheeseburger, 2 medium pa	Lo	Mixed Dishes - Sandwiches (single code)
27510420	Taco burger, on bun	Lo	Mixed Dishes - Sandwiches (single code)
27510425	Double bacon cheeseburger (2 patt	Lo	Mixed Dishes - Sandwiches (single code)
27510430	Double bacon cheeseburger (2 patt	Lo	Mixed Dishes - Sandwiches (single code)
27510431	Double bacon cheeseburger, 2 sma	Lo	Mixed Dishes - Sandwiches (single code)
27510435	Double bacon cheeseburger (2 patt	Lo	Mixed Dishes - Sandwiches (single code)
27510440	Bacon cheeseburger, 1/4 lb meat, v	Lo	Mixed Dishes - Sandwiches (single code)
27510445	Bacon cheeseburger, 1/3 lb meat, v	Lo	Mixed Dishes - Sandwiches (single code)
27510446	Double bacon cheeseburger, 2 mec	Lo	Mixed Dishes - Sandwiches (single code)
27510450	Cheeseburger, 1/4 lb meat, with ha	Lo	Mixed Dishes - Sandwiches (single code)
27510451	Double bacon cheeseburger, 2 mec	Lo	Mixed Dishes - Sandwiches (single code)
27510465	Double bacon cheeseburger, 2 mec	Lo	Mixed Dishes - Sandwiches (single code)
27510475	Double bacon cheeseburger, 2 larg	Lo	Mixed Dishes - Sandwiches (single code)
27510480	Cheeseburger (hamburger with che	Lo	Mixed Dishes - Sandwiches (single code)
27510486	Triple cheeseburger, 3 medium pat	Lo	Mixed Dishes - Sandwiches (single code)
27510500	Hamburger, plain, on bun	Lo	Mixed Dishes - Sandwiches (single code)

27510501	Hamburger slider, from fast food	Lo	Mixed Dishes - Sandwiches (single code)
27510506	Hamburger, 1 miniature patty, with (	Lo	Mixed Dishes - Sandwiches (single code)
27510510	Hamburger, with tomato and/or cats	Lo	Mixed Dishes - Sandwiches (single code)
27510511	Hamburger, 1 miniature patty, on m	Lo	Mixed Dishes - Sandwiches (single code)
27510520	Hamburger, with mayonnaise or sal	Lo	Mixed Dishes - Sandwiches (single code)
27510530	Hamburger, 1/4 lb meat, plain, on b	Lo	Mixed Dishes - Sandwiches (single code)
27510531	Hamburger, from fast food, 1 small	Lo	Mixed Dishes - Sandwiches (single code)
27510536	Hamburger, 1 small patty, with cond	Lo	Mixed Dishes - Sandwiches (single code)
27510540	Double hamburger (2 patties), with	Lo	Mixed Dishes - Sandwiches (single code)
27510550	Double hamburger (2 patties), with	Lo	Mixed Dishes - Sandwiches (single code)
27510551	Hamburger (Burger King)	Lo	Mixed Dishes - Sandwiches (single code)
27510552	Whopper Jr (Burger King)	Lo	Mixed Dishes - Sandwiches (single code)
27510553	Hamburger (McDonalds)	Lo	Mixed Dishes - Sandwiches (single code)
27510555	Hamburger, 1 small patty, with cond	Lo	Mixed Dishes - Sandwiches (single code)
27510560	Hamburger, 1/4 lb meat, with mayo	Lo	Mixed Dishes - Sandwiches (single code)
27510565	Hamburger, from school cafeteria	Lo	Mixed Dishes - Sandwiches (single code)
27510570	Hamburger, 2-1/2 oz meat, with ma	Lo	Mixed Dishes - Sandwiches (single code)
27510573	Hamburger slider	Lo	Mixed Dishes - Sandwiches (single code)
27510575	Hamburger, on white bun, 1 small p	Lo	Mixed Dishes - Sandwiches (single code)
27510576	Hamburger, on wheat bun, 1 small	Lo	Mixed Dishes - Sandwiches (single code)
27510577	Hamburger, 1 small patty, plain, on	Lo	Mixed Dishes - Sandwiches (single code)
27510585	Hamburger, 1 small patty, with cond	Lo	Mixed Dishes - Sandwiches (single code)
27510587	Hamburger, 1 small patty, with cond	Lo	Mixed Dishes - Sandwiches (single code)
27510590	Hamburger, with mayonnaise or sal	Lo	Mixed Dishes - Sandwiches (single code)
27510600	Hamburger, 1 oz meat, plain, on mi	Lo	Mixed Dishes - Sandwiches (single code)
27510601	Hamburger, from fast food, 1 mediu	Lo	Mixed Dishes - Sandwiches (single code)
27510605	Hamburger, from fast food, 1 large	Lo	Mixed Dishes - Sandwiches (single code)
27510606	Hamburger, 1 medium patty, with co	Lo	Mixed Dishes - Sandwiches (single code)
27510610	Hamburger, 1 oz meat, with tomato	Lo	Mixed Dishes - Sandwiches (single code)
27510615	Whopper (Burger King)	Lo	Mixed Dishes - Sandwiches (single code)
27510616	Hamburger, 1 medium patty, with co	Lo	Mixed Dishes - Sandwiches (single code)
27510620	Hamburger, 1/4 lb meat, with tomat	Lo	Mixed Dishes - Sandwiches (single code)
27510631	Hamburger, on white bun, 1 mediur	Lo	Mixed Dishes - Sandwiches (single code)
27510632	Hamburger, on wheat bun, 1 mediu	Lo	Mixed Dishes - Sandwiches (single code)
27510633	Hamburger, 1 medium patty, plain,	Lo	Mixed Dishes - Sandwiches (single code)
27510635	Hamburger, on white bun, 1 large p	Lo	Mixed Dishes - Sandwiches (single code)
27510636	Hamburger, on wheat bun, 1 large	Lo	Mixed Dishes - Sandwiches (single code)
27510641	Hamburger, 1 medium patty, with co	Lo	Mixed Dishes - Sandwiches (single code)
27510642	Hamburger, 1 medium patty, with co	Lo	Mixed Dishes - Sandwiches (single code)
27510643	Hamburger, 1 medium patty, with co	Lo	Mixed Dishes - Sandwiches (single code)
27510649	Double hamburger, on white bun, 2	Lo	Mixed Dishes - Sandwiches (single code)
27510655	Double hamburger, on white bun, 2	Lo	Mixed Dishes - Sandwiches (single code)
27510657	Double hamburger, on wheat bun, 2	Lo	Mixed Dishes - Sandwiches (single code)
27510658	Double hamburger, on white bun, 2	Lo	Mixed Dishes - Sandwiches (single code)
27510661	Double hamburger, from fast food,	Lo	Mixed Dishes - Sandwiches (single code)
27510667	Double hamburger, 2 small patties,	Lo	Mixed Dishes - Sandwiches (single code)
27510670	Double hamburger (2 patties), with	Lo	Mixed Dishes - Sandwiches (single code)
27510671	Double hamburger, from fast food,	Lo	Mixed Dishes - Sandwiches (single code)
27510675	Double hamburger, from fast food,	Lo	Mixed Dishes - Sandwiches (single code)
27510676	Double hamburger, 2 medium pattie	Lo	Mixed Dishes - Sandwiches (single code)
27510680	Double hamburger (2 patties, 1/4 lb	Lo	Mixed Dishes - Sandwiches (single code)

27510681	Double hamburger, 2 medium pattie	Lo	Mixed Dishes - Sandwiches (single code)
27510682	Double hamburger, 2 medium pattie	Lo	Mixed Dishes - Sandwiches (single code)
27510690	Double hamburger (2 patties, 1/4 lb	Lo	Mixed Dishes - Sandwiches (single code)
27510700	Meatball and spaghetti sauce subm	Lo	Mixed Dishes - Sandwiches (single code)
27510705	Chiliburger, with or without cheese,	Lo	Mixed Dishes - Sandwiches (single code)
27510910	Corned beef sandwich	Lo	Mixed Dishes - Sandwiches (single code)
27510950	Reuben sandwich, corned beef san	Lo	Mixed Dishes - Sandwiches (single code)
27511010	Pastrami sandwich	Lo	Mixed Dishes - Sandwiches (single code)
27513010	Roast beef sandwich	Lo	Mixed Dishes - Sandwiches (single code)
27513040	Roast beef submarine sandwich, wi	Lo	Mixed Dishes - Sandwiches (single code)
27513041	Roast beef submarine sandwich, wi	Lo	Mixed Dishes - Sandwiches (single code)
27513050	Roast beef sandwich with cheese	Lo	Mixed Dishes - Sandwiches (single code)
27513060	Roast beef sandwich with bacon an	Lo	Mixed Dishes - Sandwiches (single code)
27513070	Roast beef submarine sandwich, or	Lo	Mixed Dishes - Sandwiches (single code)
27515000	Steak submarine sandwich with lett	Lo	Mixed Dishes - Sandwiches (single code)
27515010	Steak sandwich, plain, on roll	Lo	Mixed Dishes - Sandwiches (single code)
27515020	Steak and cheese submarine sandw	Lo	Mixed Dishes - Sandwiches (single code)
27515030	Steak and cheese sandwich, plain,	Lo	Mixed Dishes - Sandwiches (single code)
27515040	Steak and cheese submarine sandw	Lo	Mixed Dishes - Sandwiches (single code)
27515050	Fajita-style beef sandwich with chee	Lo	Mixed Dishes - Sandwiches (single code)
27515070	Steak and cheese submarine sandw	Lo	Mixed Dishes - Sandwiches (single code)
27515080	Steak sandwich, plain, on biscuit	Lo	Mixed Dishes - Sandwiches (single code)
27516010	Gyro sandwich (pita bread, beef, lar	Lo	Mixed Dishes - Sandwiches (single code)
27517000	Hamburger wrap sandwich, from fa	Lo	Mixed Dishes - Sandwiches (single code)
27517010	Wrap sandwich filled with beef patty	Lo	Mixed Dishes - Sandwiches (single code)
27520130	Bacon, chicken, and tomato club sa	Lo	Mixed Dishes - Sandwiches (single code)
27520135	Bacon, chicken, and tomato club sa	Lo	Mixed Dishes - Sandwiches (single code)
27520140	Bacon and egg sandwich	Lo	Mixed Dishes - Sandwiches (single code)
27520150	Bacon, lettuce, and tomato sandwic	Lo	Mixed Dishes - Sandwiches (single code)
27520155	Bacon, lettuce, and tomato submar	Lo	Mixed Dishes - Sandwiches (single code)
27520156	Bacon, lettuce, tomato, and cheese	Lo	Mixed Dishes - Sandwiches (single code)
27520160	Bacon, chicken, and tomato club sa	Lo	Mixed Dishes - Sandwiches (single code)
27520165	Bacon, breaded fried chicken fillet,	Lo	Mixed Dishes - Sandwiches (single code)
27520166	Bacon, breaded fried chicken fillet,	Lo	Mixed Dishes - Sandwiches (single code)
27520170	Bacon on biscuit	Lo	Mixed Dishes - Sandwiches (single code)
27520250	Ham on biscuit	Lo	Mixed Dishes - Sandwiches (single code)
27520300	Ham sandwich, with spread	Lo	Mixed Dishes - Sandwiches (single code)
27520310	Ham sandwich with lettuce and spre	Lo	Mixed Dishes - Sandwiches (single code)
27520320	Ham and cheese sandwich, with let	Lo	Mixed Dishes - Sandwiches (single code)
27520330	Ham and egg sandwich	Lo	Mixed Dishes - Sandwiches (single code)
27520350	Ham and cheese sandwich, with sp	Lo	Mixed Dishes - Sandwiches (single code)
27520360	Ham and cheese sandwich, on bun	Lo	Mixed Dishes - Sandwiches (single code)
27520370	Hot ham and cheese sandwich, on	Lo	Mixed Dishes - Sandwiches (single code)
27520380	Ham and cheese on English muffin	Lo	Mixed Dishes - Sandwiches (single code)
27520390	Ham and cheese submarine sandw	Lo	Mixed Dishes - Sandwiches (single code)
27520410	Cuban sandwich, with spread	Lo	Mixed Dishes - Sandwiches (single code)
27520500	Pork sandwich, on white roll, with o	Lo	Mixed Dishes - Sandwiches (single code)
27520510	Pork barbecue sandwich or Sloppy	Lo	Mixed Dishes - Sandwiches (single code)
27520520	Pork sandwich	Lo	Mixed Dishes - Sandwiches (single code)
27540110	Sliced chicken sandwich, with sprea	Lo	Mixed Dishes - Sandwiches (single code)
27540111	Sliced chicken sandwich, with chee	Lo	Mixed Dishes - Sandwiches (single code)

27540120	Chicken salad or chicken spread sa	Lo	Mixed Dishes - Sandwiches (single code)
27540130	Chicken barbecue sandwich	Lo	Mixed Dishes - Sandwiches (single code)
27540132	Chicken fillet sandwich, NFS	Lo	Mixed Dishes - Sandwiches (single code)
27540139	Chicken fillet sandwich, from schoo	Lo	Mixed Dishes - Sandwiches (single code)
27540140	Chicken fillet, breaded, fried, sandw	Lo	Mixed Dishes - Sandwiches (single code)
27540145	Chicken fillet biscuit, from fast food	Lo	Mixed Dishes - Sandwiches (single code)
27540146	Chicken fillet sandwich, fried, from f	Lo	Mixed Dishes - Sandwiches (single code)
27540147	Chicken fillet sandwich, fried, from f	Lo	Mixed Dishes - Sandwiches (single code)
27540150	Chicken fillet, breaded, fried, sandw	Lo	Mixed Dishes - Sandwiches (single code)
27540151	Chicken fillet, breaded, fried, sandw	Lo	Mixed Dishes - Sandwiches (single code)
27540152	Chicken fillet sandwich, grilled, from	Lo	Mixed Dishes - Sandwiches (single code)
27540153	Chicken fillet sandwich, grilled, from	Lo	Mixed Dishes - Sandwiches (single code)
27540160	Chicken fillet sandwich, NS as to fri	Lo	Mixed Dishes - Sandwiches (single code)
27540170	Chicken patty sandwich, miniature,	Lo	Mixed Dishes - Sandwiches (single code)
27540175	Chicken fillet sandwich, fried, on wh	Lo	Mixed Dishes - Sandwiches (single code)
27540176	Chicken fillet sandwich, fried, on wh	Lo	Mixed Dishes - Sandwiches (single code)
27540180	Chicken patty sandwich or biscuit	Lo	Mixed Dishes - Sandwiches (single code)
27540185	Chicken fillet sandwich, fried, on wh	Lo	Mixed Dishes - Sandwiches (single code)
27540186	Chicken fillet sandwich, fried, on wh	Lo	Mixed Dishes - Sandwiches (single code)
27540190	Chicken patty sandwich, with lettuce	Lo	Mixed Dishes - Sandwiches (single code)
27540195	Chicken fillet sandwich, grilled, on v	Lo	Mixed Dishes - Sandwiches (single code)
27540196	Chicken fillet sandwich, grilled, on v	Lo	Mixed Dishes - Sandwiches (single code)
27540200	Fajita-style chicken sandwich with c	Lo	Mixed Dishes - Sandwiches (single code)
27540205	Chicken fillet sandwich, grilled, on v	Lo	Mixed Dishes - Sandwiches (single code)
27540206	Chicken fillet sandwich, grilled, on v	Lo	Mixed Dishes - Sandwiches (single code)
27540210	Chicken fillet wrap sandwich, fried,	Lo	Mixed Dishes - Sandwiches (single code)
27540230	Chicken patty sandwich with cheese	Lo	Mixed Dishes - Sandwiches (single code)
27540235	Chicken fillet, broiled, sandwich with	Lo	Mixed Dishes - Sandwiches (single code)
27540240	Chicken fillet, broiled, sandwich, on	Lo	Mixed Dishes - Sandwiches (single code)
27540250	Chicken fillet, broiled, sandwich with	Lo	Mixed Dishes - Sandwiches (single code)
27540260	Chicken fillet, broiled, sandwich, on	Lo	Mixed Dishes - Sandwiches (single code)
27540270	Chicken fillet, broiled, sandwich, wit	Lo	Mixed Dishes - Sandwiches (single code)
27540280	Chicken fillet, broiled, sandwich with	Lo	Mixed Dishes - Sandwiches (single code)
27540285	Chicken, bacon, and tomato club sa	Lo	Mixed Dishes - Sandwiches (single code)
27540290	Chicken submarine sandwich, with	Lo	Mixed Dishes - Sandwiches (single code)
27540291	Chicken submarine sandwich, with	Lo	Mixed Dishes - Sandwiches (single code)
27540295	Buffalo chicken submarine sandwic	Lo	Mixed Dishes - Sandwiches (single code)
27540296	Buffalo chicken submarine sandwic	Lo	Mixed Dishes - Sandwiches (single code)
27540300	Chicken fillet wrap sandwich, grilled	Lo	Mixed Dishes - Sandwiches (single code)
27540310	Turkey sandwich, with spread	Lo	Mixed Dishes - Sandwiches (single code)
27540350	Turkey submarine sandwich, with c	Lo	Mixed Dishes - Sandwiches (single code)
27540360	Turkey and bacon submarine sandw	Lo	Mixed Dishes - Sandwiches (single code)
27540361	Turkey and bacon submarine sandw	Lo	Mixed Dishes - Sandwiches (single code)
27541000	Turkey, ham, and roast beef club sa	Lo	Mixed Dishes - Sandwiches (single code)
27541001	Turkey, ham, and roast beef club sa	Lo	Mixed Dishes - Sandwiches (single code)
27545000	Turkey or chicken burger, plain, on	Lo	Mixed Dishes - Sandwiches (single code)
27545010	Turkey or chicken burger, with cond	Lo	Mixed Dishes - Sandwiches (single code)
27545100	Turkey or chicken burger, on white	Lo	Mixed Dishes - Sandwiches (single code)
27545110	Turkey or chicken burger, on wheat	Lo	Mixed Dishes - Sandwiches (single code)
27545200	Turkey or chicken burger, with cond	Lo	Mixed Dishes - Sandwiches (single code)
27545210	Turkey or chicken burger, with cond	Lo	Mixed Dishes - Sandwiches (single code)

27545220	Turkey or chicken burger, with cond	Lo	Mixed Dishes - Sandwiches (single code)
27550000	Fish sandwich, fried, from fast food	Lo	Mixed Dishes - Sandwiches (single code)
27550100	Fish sandwich, fried, from fast food	Lo	Mixed Dishes - Sandwiches (single code)
27550110	Crab cake sandwich	Lo	Mixed Dishes - Sandwiches (single code)
27550120	Salmon cake sandwich	Lo	Mixed Dishes - Sandwiches (single code)
27550150	Fried seafood sandwich	Lo	Mixed Dishes - Sandwiches (single code)
27550200	Fish sandwich, from school cafeteri	Lo	Mixed Dishes - Sandwiches (single code)
27550300	Fish sandwich, NFS	Lo	Mixed Dishes - Sandwiches (single code)
27550400	Fish sandwich, fried, on white bun	Lo	Mixed Dishes - Sandwiches (single code)
27550405	Fish sandwich, fried, on white bun,	Lo	Mixed Dishes - Sandwiches (single code)
27550410	Fish sandwich, fried, on wheat bun	Lo	Mixed Dishes - Sandwiches (single code)
27550420	Fish sandwich, grilled	Lo	Mixed Dishes - Sandwiches (single code)
27550425	Fish wrap sandwich	Lo	Mixed Dishes - Sandwiches (single code)
27550510	Sardine sandwich	Lo	Mixed Dishes - Sandwiches (single code)
27550720	Tuna salad sandwich, on bread	Lo	Mixed Dishes - Sandwiches (single code)
27550730	Tuna salad sandwich, on bread, wit	Lo	Mixed Dishes - Sandwiches (single code)
27550740	Tuna salad sandwich, on bun	Lo	Mixed Dishes - Sandwiches (single code)
27550745	Tuna salad sandwich, on bun, with	Lo	Mixed Dishes - Sandwiches (single code)
27550750	Tuna salad submarine sandwich, w	Lo	Mixed Dishes - Sandwiches (single code)
27550751	Tuna salad submarine sandwich, w	Lo	Mixed Dishes - Sandwiches (single code)
27550755	Tuna salad wrap sandwich	Lo	Mixed Dishes - Sandwiches (single code)
27550800	Seafood salad sandwich	Lo	Mixed Dishes - Sandwiches (single code)
27560110	Bologna sandwich, with spread	Lo	Mixed Dishes - Sandwiches (single code)
27560120	Bologna and cheese sandwich, with	Lo	Mixed Dishes - Sandwiches (single code)
27560300	Corn dog, frankfurter or hot dog wit	Lo	Mixed Dishes - Sandwiches (single code)
27560320	Frankfurter or hot dog, plain, on bur	Lo	Mixed Dishes - Sandwiches (single code)
27560330	Frankfurter or hot dog, with cheese	Lo	Mixed Dishes - Sandwiches (single code)
27560340	Frankfurter or hot dog, with catsup	Lo	Mixed Dishes - Sandwiches (single code)
27560350	Pig in a blanket, frankfurter or hot d	Lo	Mixed Dishes - Sandwiches (single code)
27560360	Frankfurter or hot dog, with chili, on	Lo	Mixed Dishes - Sandwiches (single code)
27560370	Frankfurter or hot dog with chili and	Lo	Mixed Dishes - Sandwiches (single code)
27560400	Chicken frankfurter or hot dog, plair	Lo	Mixed Dishes - Sandwiches (single code)
27560500	Pepperoni and salami submarine sa	Lo	Mixed Dishes - Sandwiches (single code)
27560510	Salami sandwich, with spread	Lo	Mixed Dishes - Sandwiches (single code)
27560650	Sausage on biscuit	Lo	Mixed Dishes - Sandwiches (single code)
27560660	Sausage griddle cake sandwich	Lo	Mixed Dishes - Sandwiches (single code)
27560670	Sausage and cheese on English mu	Lo	Mixed Dishes - Sandwiches (single code)
27560705	Sausage balls, made with biscuit m	Lo	Mixed Dishes - Meat, Poultry, Seafood
27560710	Sausage sandwich	Lo	Mixed Dishes - Sandwiches (single code)
27560910	Cold cut sumarine sandwich, with c	Lo	Mixed Dishes - Sandwiches (single code)
27564000	Frankfurter or hot dog sandwich, NI	Lo	Mixed Dishes - Sandwiches (single code)
27564001	Frankfurter or hot dog sandwich, NI	Lo	Mixed Dishes - Sandwiches (single code)
27564002	Frankfurter or hot dog sandwich, NI	Lo	Mixed Dishes - Sandwiches (single code)
27564010	Frankfurter or hot dog sandwich, NI	Lo	Mixed Dishes - Sandwiches (single code)
27564020	Frankfurter or hot dog sandwich, NI	Lo	Mixed Dishes - Sandwiches (single code)
27564030	Frankfurter or hot dog sandwich, NI	Lo	Mixed Dishes - Sandwiches (single code)
27564060	Frankfurter or hot dog sandwich, be	Lo	Mixed Dishes - Sandwiches (single code)
27564061	Frankfurter or hot dog sandwich, be	Lo	Mixed Dishes - Sandwiches (single code)
27564062	Frankfurter or hot dog sandwich, be	Lo	Mixed Dishes - Sandwiches (single code)
27564063	Frankfurter or hot dog sandwich, be	Lo	Mixed Dishes - Sandwiches (single code)
27564064	Frankfurter or hot dog sandwich, be	Lo	Mixed Dishes - Sandwiches (single code)

27564070	Frankfurter or hot dog sandwich, be	Lo	Mixed Dishes - Sandwiches (single code)
27564080	Frankfurter or hot dog sandwich, be	Lo	Mixed Dishes - Sandwiches (single code)
27564090	Frankfurter or hot dog sandwich, be	Lo	Mixed Dishes - Sandwiches (single code)
27564100	Frankfurter or hot dog sandwich, be	Lo	Mixed Dishes - Sandwiches (single code)
27564110	Frankfurter or hot dog sandwich, be	Lo	Mixed Dishes - Sandwiches (single code)
27564120	Frankfurter or hot dog sandwich, be	Lo	Mixed Dishes - Sandwiches (single code)
27564121	Frankfurter or hot dog sandwich, be	Lo	Mixed Dishes - Sandwiches (single code)
27564122	Frankfurter or hot dog sandwich, be	Lo	Mixed Dishes - Sandwiches (single code)
27564130	Frankfurter or hot dog sandwich, be	Lo	Mixed Dishes - Sandwiches (single code)
27564140	Frankfurter or hot dog sandwich, be	Lo	Mixed Dishes - Sandwiches (single code)
27564150	Frankfurter or hot dog sandwich, be	Lo	Mixed Dishes - Sandwiches (single code)
27564160	Frankfurter or hot dog sandwich, be	Lo	Mixed Dishes - Sandwiches (single code)
27564180	Frankfurter or hot dog sandwich, m	Lo	Mixed Dishes - Sandwiches (single code)
27564181	Frankfurter or hot dog sandwich, m	Lo	Mixed Dishes - Sandwiches (single code)
27564182	Frankfurter or hot dog sandwich, m	Lo	Mixed Dishes - Sandwiches (single code)
27564190	Frankfurter or hot dog sandwich, m	Lo	Mixed Dishes - Sandwiches (single code)
27564200	Frankfurter or hot dog sandwich, m	Lo	Mixed Dishes - Sandwiches (single code)
27564210	Frankfurter or hot dog sandwich, m	Lo	Mixed Dishes - Sandwiches (single code)
27564220	Frankfurter or hot dog sandwich, m	Lo	Mixed Dishes - Sandwiches (single code)
27564230	Frankfurter or hot dog sandwich, m	Lo	Mixed Dishes - Sandwiches (single code)
27564240	Frankfurter or hot dog sandwich, ch	Lo	Mixed Dishes - Sandwiches (single code)
27564241	Frankfurter or hot dog sandwich, ch	Lo	Mixed Dishes - Sandwiches (single code)
27564242	Frankfurter or hot dog sandwich, ch	Lo	Mixed Dishes - Sandwiches (single code)
27564243	Frankfurter or hot dog sandwich, ch	Lo	Mixed Dishes - Sandwiches (single code)
27564250	Frankfurter or hot dog sandwich, ch	Lo	Mixed Dishes - Sandwiches (single code)
27564260	Frankfurter or hot dog sandwich, ch	Lo	Mixed Dishes - Sandwiches (single code)
27564270	Frankfurter or hot dog sandwich, ch	Lo	Mixed Dishes - Sandwiches (single code)
27564280	Frankfurter or hot dog sandwich, ch	Lo	Mixed Dishes - Sandwiches (single code)
27564290	Frankfurter or hot dog sandwich, ch	Lo	Mixed Dishes - Sandwiches (single code)
27564300	Frankfurter or hot dog sandwich, re	Lo	Mixed Dishes - Sandwiches (single code)
27564301	Frankfurter or hot dog sandwich, re	Lo	Mixed Dishes - Sandwiches (single code)
27564303	Frankfurter or hot dog sandwich, re	Lo	Mixed Dishes - Sandwiches (single code)
27564310	Frankfurter or hot dog sandwich, re	Lo	Mixed Dishes - Sandwiches (single code)
27564330	Frankfurter or hot dog sandwich, re	Lo	Mixed Dishes - Sandwiches (single code)
27564360	Frankfurter or hot dog sandwich, fa	Lo	Mixed Dishes - Sandwiches (single code)
27564361	Frankfurter or hot dog sandwich, fa	Lo	Mixed Dishes - Sandwiches (single code)
27564362	Frankfurter or hot dog sandwich, fa	Lo	Mixed Dishes - Sandwiches (single code)
27564364	Frankfurter or hot dog sandwich, fa	Lo	Mixed Dishes - Sandwiches (single code)
27564370	Frankfurter or hot dog sandwich, fa	Lo	Mixed Dishes - Sandwiches (single code)
27564380	Frankfurter or hot dog sandwich, fa	Lo	Mixed Dishes - Sandwiches (single code)
27564418	Frankfurter or hot dog sandwich, re	Lo	Mixed Dishes - Sandwiches (single code)
27564420	Frankfurter or hot dog sandwich, m	Lo	Mixed Dishes - Sandwiches (single code)
27564430	Frankfurter or hot dog sandwich, m	Lo	Mixed Dishes - Sandwiches (single code)
27564440	Frankfurter or hot dog sandwich, wi	Lo	Mixed Dishes - Sandwiches (single code)
27564441	Frankfurter or hot dog sandwich, wi	Lo	Mixed Dishes - Sandwiches (single code)
27564442	Frankfurter or hot dog sandwich, wi	Lo	Mixed Dishes - Sandwiches (single code)
27564443	Frankfurter or hot dog sandwich, wi	Lo	Mixed Dishes - Sandwiches (single code)
27564450	Frankfurter or hot dog sandwich, wi	Lo	Mixed Dishes - Sandwiches (single code)
27564460	Frankfurter or hot dog sandwich, wi	Lo	Mixed Dishes - Sandwiches (single code)
27564500	Frankfurter or hot dog sandwich, wi	Lo	Mixed Dishes - Sandwiches (single code)
27564510	Frankfurter or hot dog sandwich, wi	Lo	Mixed Dishes - Sandwiches (single code)

27564520	Frankfurter or hot dog sandwich, wi	Lo	Mixed Dishes - Sandwiches (single code)
27570310	Hors d'oeuvres, with spread	Lo	Mixed Dishes - Sandwiches (single code)
27601000	Beef stew, baby food, toddler	Lo	Baby Foods
27610100	Beef and egg noodles, baby food, N	Lo	Baby Foods
27610110	Beef and egg noodles, baby food, s	Lo	Baby Foods
27610120	Beef and egg noodles, baby food, ju	Lo	Baby Foods
27610710	Beef with vegetables, baby food, str	Lo	Baby Foods
27610730	Beef with vegetables, baby food, to	Lo	Baby Foods
27640050	Chicken and rice dinner, baby food,	Lo	Baby Foods
27640100	Chicken noodle dinner, baby food, N	Lo	Baby Foods
27640110	Chicken noodle dinner, baby food, s	Lo	Baby Foods
27640120	Chicken noodle dinner, baby food, j	Lo	Baby Foods
27640810	Chicken, noodles, and vegetables,	Lo	Baby Foods
27641000	Chicken stew, baby food, toddler	Lo	Baby Foods
27642100	Turkey, rice and vegetables, baby fo	Lo	Baby Foods
27642110	Turkey, rice and vegetables, baby fo	Lo	Baby Foods
27642120	Turkey, rice and vegetables, baby fo	Lo	Baby Foods
27642130	Turkey, rice, and vegetables, baby f	Lo	Baby Foods
27644110	Chicken soup, baby food	Lo	Baby Foods
28101000	Frozen dinner, NFS	Lo	Mixed Dishes - Meat, Poultry, Seafood
28110000	Beef dinner, NFS, frozen meal	Lo	Mixed Dishes - Meat, Poultry, Seafood
28110110	Beef with potatoes (frozen meal)	Lo	Mixed Dishes - Meat, Poultry, Seafood
28110120	Beef with potatoes (frozen meal, lar	Lo	Mixed Dishes - Meat, Poultry, Seafood
28110150	Beef with vegetable, diet frozen me	Lo	Mixed Dishes - Meat, Poultry, Seafood
28110220	Sirloin, chopped, with gravy, mashe	Lo	Mixed Dishes - Meat, Poultry, Seafood
28110230	Sirloin, chopped, or swiss steak with	Lo	Mixed Dishes - Meat, Poultry, Seafood
28110250	Sirloin tips, with gravy, potatoes, ve	Lo	Mixed Dishes - Meat, Poultry, Seafood
28110270	Sirloin beef, with gravy, potatoes, ve	Lo	Mixed Dishes - Meat, Poultry, Seafood
28110300	Salisbury steak dinner, NFS, frozen	Lo	Mixed Dishes - Meat, Poultry, Seafood
28110310	Salisbury steak with gravy, potatoes	Lo	Mixed Dishes - Meat, Poultry, Seafood
28110330	Salisbury steak with gravy, whipped	Lo	Mixed Dishes - Meat, Poultry, Seafood
28110350	Salisbury steak with gravy, potatoes	Lo	Mixed Dishes - Meat, Poultry, Seafood
28110370	Salisbury steak with gravy, macaron	Lo	Mixed Dishes - Meat, Poultry, Seafood
28110380	Salisbury steak with gravy, macaron	Lo	Mixed Dishes - Meat, Poultry, Seafood
28110390	Salisbury steak, potatoes, vegetable	Lo	Mixed Dishes - Meat, Poultry, Seafood
28110500	Beef, sliced, with gravy, barley and	Lo	Mixed Dishes - Meat, Poultry, Seafood
28110510	Beef, sliced, with gravy, potatoes, v	Lo	Mixed Dishes - Meat, Poultry, Seafood
28110620	Beef short ribs, boneless, with barb	Lo	Mixed Dishes - Meat, Poultry, Seafood
28110640	Meatballs, Swedish, in sauce, with n	Lo	Mixed Dishes - Meat, Poultry, Seafood
28110660	Meatballs, Swedish, in gravy, with n	Lo	Mixed Dishes - Meat, Poultry, Seafood
28113110	Salisbury steak, baked, with tomato	Lo	Mixed Dishes - Meat, Poultry, Seafood
28113140	Beef with spaetzle or rice, vegetable	Lo	Mixed Dishes - Meat, Poultry, Seafood
28120230	Pork, sliced, with gravy, mashed po	Lo	Mixed Dishes - Meat, Poultry, Seafood
28120310	Pork with rice, vegetable, in soy-bas	Lo	Mixed Dishes - Asian
28133110	Veal, breaded, with spaghetti, in tom	Lo	Mixed Dishes - Meat, Poultry, Seafood
28140100	Chicken dinner, NFS, frozen meal	Lo	Mixed Dishes - Meat, Poultry, Seafood
28140150	Chicken divan, frozen meal	Lo	Mixed Dishes - Meat, Poultry, Seafood
28140320	Chicken and noodles with vegetable	Lo	Mixed Dishes - Meat, Poultry, Seafood
28140710	Chicken, fried, with potatoes, veget	Lo	Mixed Dishes - Meat, Poultry, Seafood
28140720	Chicken patty, or nuggets, boneless	Lo	Mixed Dishes - Meat, Poultry, Seafood
28140730	Chicken patty, breaded, with tomato	Lo	Mixed Dishes - Meat, Poultry, Seafood

28140740	Chicken patty or nuggets, boneless	Lo	Mixed Dishes - Meat, Poultry, Seafood
28140810	Chicken, fried, with potatoes, veget	Lo	Mixed Dishes - Meat, Poultry, Seafood
28141010	Chicken, fried, with potatoes, veget	Lo	Mixed Dishes - Meat, Poultry, Seafood
28141050	Chicken patty parmigiana, breaded,	Lo	Mixed Dishes - Meat, Poultry, Seafood
28141060	Chicken patty with vegetable (diet fr	Lo	Mixed Dishes - Meat, Poultry, Seafood
28141200	Chicken teriyaki with rice, vegetable	Lo	Mixed Dishes - Meat, Poultry, Seafood
28141201	Teriyaki chicken with rice and veget	Lo	Mixed Dishes - Asian
28141250	Chicken with rice and vegetable, die	Lo	Mixed Dishes - Meat, Poultry, Seafood
28141300	Chicken with rice and vegetable, re	Lo	Mixed Dishes - Meat, Poultry, Seafood
28141600	Chicken a la king with rice, frozen m	Lo	Mixed Dishes - Meat, Poultry, Seafood
28141610	Chicken and vegetables in cream o	Lo	Mixed Dishes - Meat, Poultry, Seafood
28141650	Chicken and vegetables au gratin w	Lo	Mixed Dishes - Meat, Poultry, Seafood
28143010	Chicken and vegetable entree with	Lo	Mixed Dishes - Meat, Poultry, Seafood
28143020	Chicken and vegetable entree with	Lo	Mixed Dishes - Meat, Poultry, Seafood
28143030	Chicken and vegetable entree, orie	Lo	Mixed Dishes - Meat, Poultry, Seafood
28143040	Chicken chow mein with rice, diet fr	Lo	Mixed Dishes - Asian
28143080	Chicken with noodles and cheese s	Lo	Mixed Dishes - Meat, Poultry, Seafood
28143110	Chicken cacciatore with noodles, di	Lo	Mixed Dishes - Meat, Poultry, Seafood
28143130	Chicken and vegetable entree with	Lo	Mixed Dishes - Meat, Poultry, Seafood
28143150	Chicken and vegetable entree with	Lo	Mixed Dishes - Meat, Poultry, Seafood
28143170	Chicken in cream sauce with noodle	Lo	Mixed Dishes - Meat, Poultry, Seafood
28143180	Chicken in butter sauce with potato	Lo	Mixed Dishes - Meat, Poultry, Seafood
28143190	Chicken in mushroom sauce, white	Lo	Mixed Dishes - Meat, Poultry, Seafood
28143200	Chicken in soy-based sauce, rice a	Lo	Mixed Dishes - Asian
28143210	Chicken in orange sauce with almo	Lo	Mixed Dishes - Asian
28143220	Chicken in barbecue sauce, with ric	Lo	Mixed Dishes - Meat, Poultry, Seafood
28144100	Chicken and vegetable entree with	Lo	Mixed Dishes - Meat, Poultry, Seafood
28145000	Turkey dinner, NFS, frozen meal	Lo	Mixed Dishes - Meat, Poultry, Seafood
28145010	Turkey with dressing, gravy, potato	Lo	Mixed Dishes - Meat, Poultry, Seafood
28145100	Turkey with gravy, dressing, vegeta	Lo	Mixed Dishes - Meat, Poultry, Seafood
28145110	Turkey with vegetable, stuffing, diet	Lo	Mixed Dishes - Meat, Poultry, Seafood
28145210	Turkey with gravy, dressing, potato	Lo	Mixed Dishes - Meat, Poultry, Seafood
28145610	Turkey with gravy, dressing, potato	Lo	Mixed Dishes - Meat, Poultry, Seafood
28145710	Turkey tetrazzini, frozen meal	Lo	Mixed Dishes - Meat, Poultry, Seafood
28150000	Fish dinner, NFS (frozen meal)	Lo	Mixed Dishes - Meat, Poultry, Seafood
28150210	Haddock with chopped spinach, die	Lo	Mixed Dishes - Meat, Poultry, Seafood
28150220	Flounder with chopped broccoli, die	Lo	Mixed Dishes - Meat, Poultry, Seafood
28150510	Fish in lemon-butter sauce with sta	Lo	Mixed Dishes - Meat, Poultry, Seafood
28150650	Fish, breaded, or fish sticks, with pa	Lo	Mixed Dishes - Meat, Poultry, Seafood
28152030	Seafood newburg with rice, vegetab	Lo	Mixed Dishes - Meat, Poultry, Seafood
28152050	Shrimp with rice, vegetable (frozen	Lo	Mixed Dishes - Meat, Poultry, Seafood
28153010	Shrimp and clams in tomato-based	Lo	Mixed Dishes - Meat, Poultry, Seafood
28154010	Shrimp and vegetables in sauce wit	Lo	Mixed Dishes - Meat, Poultry, Seafood
28160300	Meat loaf dinner, NFS, frozen meal	Lo	Mixed Dishes - Meat, Poultry, Seafood
28160310	Meat loaf with potatoes, vegetable,	Lo	Mixed Dishes - Meat, Poultry, Seafood
28160650	Stuffed green pepper, frozen meal	Lo	Mixed Dishes - Bean/Vegetable-based
28160710	Stuffed cabbage, with meat and ton	Lo	Mixed Dishes - Meat, Poultry, Seafood
28310110	Beef, broth, bouillon, or consomme	Lo	Mixed Dishes - Soups
28310150	Oxtail soup	Lo	Mixed Dishes - Soups
28310160	Beef broth, with tomato, home recip	Lo	Mixed Dishes - Soups
28310170	Beef broth, without tomato, home re	Lo	Mixed Dishes - Soups

28310210	Chili beef soup	Lo	Mixed Dishes - Soups
28310220	Chili beef soup, chunky style	Lo	Mixed Dishes - Soups
28310230	Meatball soup, home recipe, Mexica	Lo	Mixed Dishes - Soups
28310320	Beef noodle soup, Puerto Rican sty	Lo	Mixed Dishes - Soups
28310330	Pho	Lo	Mixed Dishes - Soups
28310420	Beef and rice soup, Puerto Rican st	Lo	Mixed Dishes - Soups
28311010	Pepperpot soup	Lo	Mixed Dishes - Soups
28311020	Menudo soup, home recipe	Lo	Mixed Dishes - Soups
28311030	Menudo soup, canned, prepared wi	Lo	Mixed Dishes - Soups
28315050	Beef vegetable soup with potato, pa	Lo	Mixed Dishes - Soups
28315100	Beef vegetable soup with potato, st	Lo	Mixed Dishes - Soups
28315120	Beef vegetable soup with noodles, s	Lo	Mixed Dishes - Soups
28315130	Beef vegetable soup with rice, stew	Lo	Mixed Dishes - Soups
28315140	Beef vegetable soup, home recipe,	Lo	Mixed Dishes - Soups
28315150	Meat and corn hominy soup, home	Lo	Mixed Dishes - Soups
28315160	Italian Wedding Soup	Lo	Mixed Dishes - Soups
28316020	Beef and mushroom soup, canned,	Lo	Mixed Dishes - Soups
28317010	Beef stroganoff soup, chunky style,	Lo	Mixed Dishes - Soups
28320110	Pork and rice soup, stew type, chur	Lo	Mixed Dishes - Soups
28320120	Pork vegetable soup with noodles, s	Lo	Mixed Dishes - Soups
28320130	Ham, rice, and potato soup, Puerto	Lo	Mixed Dishes - Soups
28320140	Ham, noodle, and vegetable soup,	Lo	Mixed Dishes - Soups
28320150	Pork, vegetable soup with potatoes	Lo	Mixed Dishes - Soups
28320160	Pork vegetable soup with potato, pa	Lo	Mixed Dishes - Soups
28320300	Pork with vegetable excluding carro	Lo	Mixed Dishes - Soups
28321130	Bacon soup, cream of, prepared wi	Lo	Mixed Dishes - Soups
28330110	Scotch broth (lamb, vegetables, and	Lo	Mixed Dishes - Soups
28331110	Lamb, pasta, and vegetable soup, P	Lo	Mixed Dishes - Soups
28340110	Chicken or turkey broth, bouillon, or	Lo	Mixed Dishes - Soups
28340120	Chicken or turkey broth, without tom	Lo	Mixed Dishes - Soups
28340130	Chicken or turkey broth, with tomato	Lo	Mixed Dishes - Soups
28340140	Chicken broth, bouillion, or consom	Lo	Other
28340150	Mexican style chicken broth soup st	Lo	Mixed Dishes - Soups
28340170	Chicken broth, canned, low sodium	Lo	Mixed Dishes - Soups
28340179	Beef broth, less or reduced sodium	Lo	Mixed Dishes - Soups
28340180	Chicken or turkey broth, less or red	Lo	Mixed Dishes - Soups
28340210	Chicken rice soup, Puerto Rican sty	Lo	Mixed Dishes - Soups
28340220	Chicken soup with noodles and pota	Lo	Mixed Dishes - Soups
28340310	Chicken or turkey gumbo soup, hor	Lo	Mixed Dishes - Soups
28340510	Chicken or turkey noodle soup, chu	Lo	Mixed Dishes - Soups
28340520	Chicken soup, canned, undiluted	Lo	Mixed Dishes - Soups
28340530	Chicken soup	Lo	Mixed Dishes - Soups
28340550	Sweet and sour soup	Lo	Mixed Dishes - Soups
28340580	Chicken or turkey soup with vegeta	Lo	Mixed Dishes - Soups
28340590	Chicken or turkey corn soup with no	Lo	Mixed Dishes - Soups
28340600	Chicken or turkey vegetable soup, c	Lo	Mixed Dishes - Soups
28340610	Chicken or turkey vegetable soup, s	Lo	Mixed Dishes - Soups
28340630	Chicken or turkey vegetable soup w	Lo	Mixed Dishes - Soups
28340640	Chicken or turkey vegetable soup w	Lo	Mixed Dishes - Soups
28340660	Chicken or turkey vegetable soup, h	Lo	Mixed Dishes - Soups
28340670	Chicken or turkey vegetable soup w	Lo	Mixed Dishes - Soups

28340680	Chicken or turkey and corn hominy	Lo	Mixed Dishes - Soups
28340690	Chicken or turkey vegetable soup w	Lo	Mixed Dishes - Soups
28340700	Bird's nest soup	Lo	Mixed Dishes - Soups
28340750	Hot and sour soup	Lo	Mixed Dishes - Soups
28340800	Chicken or turkey soup with vegeta	Lo	Mixed Dishes - Soups
28345010	Chicken or turkey soup, cream of, c	Lo	Mixed Dishes - Soups
28345020	Chicken or turkey soup, cream of, c	Lo	Mixed Dishes - Soups
28345030	Chicken or turkey soup, cream of, c	Lo	Mixed Dishes - Soups
28345040	Chicken or turkey soup, cream of, c	Lo	Mixed Dishes - Soups
28345110	Chicken or turkey soup, cream of, N	Lo	Mixed Dishes - Soups
28345120	Chicken or turkey soup, cream of, p	Lo	Mixed Dishes - Soups
28345130	Chicken or turkey soup, cream of, p	Lo	Mixed Dishes - Soups
28345140	Chicken or turkey soup, cream of, c	Lo	Mixed Dishes - Soups
28345160	Chicken or turkey mushroom soup,	Lo	Mixed Dishes - Soups
28345170	Duck soup	Lo	Mixed Dishes - Soups
28350040	Fish stock, home recipe	Lo	Mixed Dishes - Soups
28350050	Fish chowder	Lo	Mixed Dishes - Soups
28350110	Crab soup, NS as to tomato-base o	Lo	Mixed Dishes - Soups
28350120	Crab soup, tomato-base	Lo	Mixed Dishes - Soups
28350210	Clam chowder, NS as to Manhattan	Lo	Mixed Dishes - Soups
28350220	Clam chowder, Manhattan	Lo	Mixed Dishes - Soups
28351110	Fish and vegetable soup, no potato	Lo	Mixed Dishes - Soups
28351120	Fish soup with potatoes, Mexican st	Lo	Mixed Dishes - Soups
28351160	Codfish, rice, and vegetable soup, F	Lo	Mixed Dishes - Soups
28355110	Clam chowder, New England, NS a	Lo	Mixed Dishes - Soups
28355120	Clam chowder, New England, prepa	Lo	Mixed Dishes - Soups
28355130	Clam chowder, New England, prepa	Lo	Mixed Dishes - Soups
28355140	Clam chowder, New England, reduc	Lo	Mixed Dishes - Soups
28355210	Crab soup, cream of, prepared with	Lo	Mixed Dishes - Soups
28355250	Lobster bisque	Lo	Mixed Dishes - Soups
28355260	Lobster gumbo	Lo	Mixed Dishes - Meat, Poultry, Seafood
28355310	Oyster stew	Lo	Mixed Dishes - Soups
28355350	Salmon soup, cream style	Lo	Mixed Dishes - Soups
28355410	Shrimp soup, cream of, NS as to pr	Lo	Mixed Dishes - Soups
28355420	Shrimp soup, cream of, prepared w	Lo	Mixed Dishes - Soups
28355430	Shrimp soup, cream of, prepared w	Lo	Mixed Dishes - Soups
28355440	Shrimp gumbo	Lo	Mixed Dishes - Meat, Poultry, Seafood
28355450	Seafood soup with potatoes and ve	Lo	Mixed Dishes - Soups
28355460	Seafood soup with potatoes, and ve	Lo	Mixed Dishes - Soups
28355470	Seafood soup with vegetables inclu	Lo	Mixed Dishes - Soups
28355480	Seafood soup with vegetables exclu	Lo	Mixed Dishes - Soups
28360100	Meat broth, Puerto Rican style	Lo	Mixed Dishes - Soups
28401200	Gelatin drink, powder, flavored, with	Lo	Diet Beverages
28410000	Bee pollen	Lo	Other
28500000	Gravy, poultry	Lo	Condiments and Sauces
28500010	Gravy, meat or poultry, with wine	Lo	Condiments and Sauces
28500020	Gravy, meat, with fruit	Lo	Condiments and Sauces
28500030	Gravy, poultry, low sodium	Lo	Condiments and Sauces
28500040	Gravy, beef	Lo	Condiments and Sauces
28500050	Gravy, giblet	Lo	Condiments and Sauces
28500070	Gravy, beef or meat, home recipe	Lo	Condiments and Sauces

28500080	Gravy, poultry, home recipe	Lo	Condiments and Sauces
28500100	Gravy, mushroom	Lo	Condiments and Sauces
28501010	Gravy, beef, fat free	Lo	Condiments and Sauces
28501110	Gravy, poultry, fat free	Lo	Condiments and Sauces
28510010	Gravy or sauce, poultry-based from	Lo	Condiments and Sauces
28510030	Gravy, meat-based, from Puerto-Ri	Lo	Condiments and Sauces
28520000	Gravy, made with soy sauce	Lo	Condiments and Sauces
28520010	Gravy, NFS	Lo	Condiments and Sauces
28520100	Oyster sauce	Lo	Condiments and Sauces
28522000	Mole sauce	Lo	Condiments and Sauces
28522050	Mole verde sauce	Lo	Condiments and Sauces
31101010	Egg, whole, raw	Lo	Eggs
31102000	Egg, whole, cooked, NS as to cooki	Lo	Eggs
31103000	Egg, whole, boiled	Lo	Eggs
31103010	Egg, whole, boiled or poached	Lo	Eggs
31104000	Egg, whole, poached	Lo	Eggs
31105000	Egg, whole, fried	Lo	Eggs
31105005	Egg, whole, fried, NS as to fat	Lo	Eggs
31105010	Egg, whole, fried no added fat	Lo	Eggs
31105020	Egg, whole, fried with margarine	Lo	Eggs
31105030	Egg, whole, fried with oil	Lo	Eggs
31105040	Egg, whole, fried with butter	Lo	Eggs
31105060	Egg, whole, fried with animal fat or	Lo	Eggs
31105080	Egg, whole, fried with cooking spray	Lo	Eggs
31105085	Egg, whole, fried, NS as to fat type	Lo	Eggs
31105090	Egg, whole, fried, from fast food / re	Lo	Eggs
31106000	Egg, whole, baked, NS as to fat	Lo	Eggs
31106010	Egg, whole, baked, no added fat	Lo	Eggs
31106020	Egg, whole, baked, fat added	Lo	Eggs
31107000	Egg, whole, pickled	Lo	Eggs
31108010	Egg, white only, raw	Lo	Eggs
31108100	Egg, white, cooked, NS as to fat	Lo	Eggs
31108110	Egg, white, cooked, no added fat	Lo	Eggs
31108120	Egg, white, cooked, fat added	Lo	Eggs
31109000	Egg, white only, cooked, NS as to fa	Lo	Eggs
31109010	Egg, white only, cooked, fat not add	Lo	Eggs
31109020	Egg, white only, cooked, fat added i	Lo	Eggs
31110010	Egg, yolk only, raw	Lo	Eggs
31111000	Egg, yolk only, cooked, NS as to fat	Lo	Eggs
31111010	Egg, yolk only, cooked, no added fa	Lo	Eggs
31111020	Egg, yolk only, cooked, fat added	Lo	Eggs
31201000	Duck egg, cooked	Lo	Eggs
31202000	Goose egg, cooked	Lo	Eggs
31203000	Quail egg, canned	Lo	Eggs
32101500	Egg, Benedict	Lo	Eggs
32101530	Egg curry	Lo	Mixed Dishes - Bean/Vegetable-based
32102000	Egg, deviled	Lo	Eggs
32103000	Egg salad, made with mayonnaise	Lo	Eggs
32103015	Egg salad, made with light mayonna	Lo	Eggs
32103020	Egg salad, made with mayonnaise-t	Lo	Eggs
32103025	Egg salad, made with light mayonna	Lo	Eggs

32103035	Egg salad, made with light creamy d	Lo	Eggs
32103050	Egg Salad, made with any type of fa	Lo	Eggs
32104900	Egg omelet or scrambled egg, NS a	Lo	Eggs
32104950	Egg omelet or scrambled egg, fat n	Lo	Eggs
32105000	Egg omelet or scrambled egg, fat a	Lo	Eggs
32105010	Egg omelet or scrambled egg, with	Lo	Eggs
32105013	Egg omelet or scrambled egg, with	Lo	Eggs
32105020	Egg omelet or scrambled egg, with	Lo	Eggs
32105030	Egg omelet or scrambled egg, with	Lo	Eggs
32105040	Egg omelet or scrambled egg, with	Lo	Eggs
32105045	Egg omelet or scrambled egg, with	Lo	Eggs
32105048	Egg omelet or scrambled egg, with	Lo	Eggs
32105050	Egg omelet or scrambled egg, with	Lo	Eggs
32105055	Egg omelet or scrambled egg, with	Lo	Eggs
32105060	Egg omelet or scrambled egg, with	Lo	Eggs
32105070	Egg omelet or scrambled egg, with	Lo	Eggs
32105080	Egg omelet or scrambled egg, with	Lo	Eggs
32105081	Egg omelet or scrambled egg, with	Lo	Eggs
32105082	Egg omelet or scrambled egg, with	Lo	Eggs
32105085	Egg omelet or scrambled egg, with	Lo	Eggs
32105100	Egg omelet or scrambled egg, with	Lo	Eggs
32105110	Egg omelet or scrambled egg, with	Lo	Eggs
32105118	Egg omelet or scrambled egg, with	Lo	Eggs
32105119	Egg omelet or scrambled egg, with	Lo	Eggs
32105120	Egg omelet or scrambled egg, with	Lo	Eggs
32105121	Egg omelet or scrambled egg, with	Lo	Eggs
32105122	Egg omelet or scrambled egg, with	Lo	Eggs
32105125	Egg omelet or scrambled egg, with	Lo	Eggs
32105126	Egg omelet or scrambled egg, with	Lo	Eggs
32105130	Egg omelet or scrambled egg, Spar	Lo	Eggs
32105150	Egg omelet or scrambled egg, with	Lo	Eggs
32105160	Egg omelet or scrambled egg, with	Lo	Eggs
32105161	Egg omelet or scrambled egg, with	Lo	Eggs
32105170	Egg omelet or scrambled egg, with	Lo	Eggs
32105180	Huevos rancheros	Lo	Eggs
32105190	Egg casserole with bread, cheese,	Lo	Eggs
32105200	Egg foo yung, NFS	Lo	Mixed Dishes - Asian
32105210	Chicken egg foo yung	Lo	Mixed Dishes - Asian
32105220	Pork egg foo yung	Lo	Mixed Dishes - Asian
32105230	Shrimp egg foo yung	Lo	Mixed Dishes - Asian
32105240	Beef egg foo yung	Lo	Mixed Dishes - Asian
32105310	Ripe plantain omelet, Puerto Rican	Lo	Eggs
32105330	Scrambled eggs with jerked beef, P	Lo	Eggs
32110150	Shrimp-egg patty	Lo	Eggs
32129990	Egg omelet or scrambled egg, NS a	Lo	Eggs
32130000	Egg omelet or scrambled egg, mad	Lo	Eggs
32130010	Egg omelet or scrambled egg, mad	Lo	Eggs
32130020	Egg omelet or scrambled egg, mad	Lo	Eggs
32130040	Egg omelet or scrambled egg, mad	Lo	Eggs
32130060	Egg omelet or scrambled egg, mad	Lo	Eggs
32130065	Egg omelet or scrambled egg, NS a	Lo	Eggs

32130070	Egg omelet or scrambled egg, no a	Lo	Eggs
32130080	Egg omelet or scrambled egg, from	Lo	Eggs
32130100	Egg omelet or scrambled egg, with	Lo	Eggs
32130110	Egg omelet or scrambled egg, with	Lo	Eggs
32130120	Egg omelet or scrambled egg, with	Lo	Eggs
32130140	Egg omelet or scrambled egg, with	Lo	Eggs
32130160	Egg omelet or scrambled egg, with	Lo	Eggs
32130170	Egg omelet or scrambled egg, with	Lo	Eggs
32130190	Egg omelet or scrambled egg, with	Lo	Eggs
32130200	Egg omelet or scrambled egg, with	Lo	Eggs
32130210	Egg omelet or scrambled egg, with	Lo	Eggs
32130220	Egg omelet or scrambled egg, with	Lo	Eggs
32130240	Egg omelet or scrambled egg, with	Lo	Eggs
32130260	Egg omelet or scrambled egg, with	Lo	Eggs
32130265	Egg omelet or scrambled egg, with	Lo	Eggs
32130270	Egg omelet or scrambled egg, with	Lo	Eggs
32130290	Egg omelet or scrambled egg, with	Lo	Eggs
32130300	Egg omelet or scrambled egg, with	Lo	Eggs
32130310	Egg omelet or scrambled egg, with	Lo	Eggs
32130320	Egg omelet or scrambled egg, with	Lo	Eggs
32130360	Egg omelet or scrambled egg, with	Lo	Eggs
32130365	Egg omelet or scrambled egg, with	Lo	Eggs
32130370	Egg omelet or scrambled egg, with	Lo	Eggs
32130400	Egg omelet or scrambled egg, with	Lo	Eggs
32130410	Egg omelet or scrambled egg, with	Lo	Eggs
32130420	Egg omelet or scrambled egg, with	Lo	Eggs
32130430	Egg omelet or scrambled egg, with	Lo	Eggs
32130440	Egg omelet or scrambled egg, with	Lo	Eggs
32130450	Egg omelet or scrambled egg, with	Lo	Eggs
32130460	Egg omelet or scrambled egg, with	Lo	Eggs
32130480	Egg omelet or scrambled egg, with	Lo	Eggs
32130490	Egg omelet or scrambled egg, with	Lo	Eggs
32130500	Egg omelet or scrambled egg, with	Lo	Eggs
32130510	Egg omelet or scrambled egg, with	Lo	Eggs
32130600	Egg omelet or scrambled egg, with	Lo	Eggs
32130610	Egg omelet or scrambled egg, with	Lo	Eggs
32130620	Egg omelet or scrambled egg, with	Lo	Eggs
32130630	Egg omelet or scrambled egg, with	Lo	Eggs
32130640	Egg omelet or scrambled egg, with	Lo	Eggs
32130650	Egg omelet or scrambled egg, with	Lo	Eggs
32130660	Egg omelet or scrambled egg, with	Lo	Eggs
32130680	Egg omelet or scrambled egg, with	Lo	Eggs
32130690	Egg omelet or scrambled egg, with	Lo	Eggs
32130700	Egg omelet or scrambled egg, with	Lo	Eggs
32130710	Egg omelet or scrambled egg, with	Lo	Eggs
32130800	Egg omelet or scrambled egg, with	Lo	Eggs
32130810	Egg omelet or scrambled egg, with	Lo	Eggs
32130820	Egg omelet or scrambled egg, with	Lo	Eggs
32130830	Egg omelet or scrambled egg, with	Lo	Eggs
32130840	Egg omelet or scrambled egg, with	Lo	Eggs
32130850	Egg omelet or scrambled egg, with	Lo	Eggs

32130890	Egg omelet or scrambled egg, with	Lo	Eggs
32130900	Egg omelet or scrambled egg, with	Lo	Eggs
32130910	Egg omelet or scrambled egg, with	Lo	Eggs
32131000	Egg omelet or scrambled egg, with	Lo	Eggs
32131010	Egg omelet or scrambled egg, with	Lo	Eggs
32131020	Egg omelet or scrambled egg, with	Lo	Eggs
32131030	Egg omelet or scrambled egg, with	Lo	Eggs
32131040	Egg omelet or scrambled egg, with	Lo	Eggs
32131050	Egg omelet or scrambled egg, with	Lo	Eggs
32131060	Egg omelet or scrambled egg, with	Lo	Eggs
32131070	Egg omelet or scrambled egg, with	Lo	Eggs
32131080	Egg omelet or scrambled egg, with	Lo	Eggs
32131090	Egg omelet or scrambled egg, with	Lo	Eggs
32131100	Egg omelet or scrambled egg, with	Lo	Eggs
32131110	Egg omelet or scrambled egg, with	Lo	Eggs
32131200	Egg omelet or scrambled egg, with	Lo	Eggs
32131210	Egg omelet or scrambled egg, with	Lo	Eggs
32131220	Egg omelet or scrambled egg, with	Lo	Eggs
32201000	Fried egg sandwich	Lo	Mixed Dishes - Sandwiches (single code)
32202000	Egg, cheese, ham, and bacon on bu	Lo	Mixed Dishes - Sandwiches (single code)
32202010	Egg, cheese, and ham on English m	Lo	Mixed Dishes - Sandwiches (single code)
32202020	Egg, cheese, and ham on biscuit	Lo	Mixed Dishes - Sandwiches (single code)
32202025	Egg, cheese and ham on bagel	Lo	Mixed Dishes - Sandwiches (single code)
32202030	Egg, cheese, and sausage on Engli	Lo	Mixed Dishes - Sandwiches (single code)
32202034	Egg, cheese, and sausage on bun	Lo	Mixed Dishes - Sandwiches (single code)
32202035	Egg, extra cheese, and extra sausa	Lo	Mixed Dishes - Sandwiches (single code)
32202040	Egg, cheese, and beef on English M	Lo	Mixed Dishes - Sandwiches (single code)
32202045	Egg, cheese, and steak on bagel	Lo	Mixed Dishes - Sandwiches (single code)
32202050	Egg, cheese, and sausage on biscu	Lo	Mixed Dishes - Sandwiches (single code)
32202055	Egg, cheese, and sausage griddle c	Lo	Mixed Dishes - Sandwiches (single code)
32202060	Egg and sausage on biscuit	Lo	Mixed Dishes - Sandwiches (single code)
32202070	Egg, cheese, and bacon on biscuit	Lo	Mixed Dishes - Sandwiches (single code)
32202075	Egg, cheese, and bacon griddle cak	Lo	Mixed Dishes - Sandwiches (single code)
32202080	Egg, cheese, and bacon on English	Lo	Mixed Dishes - Sandwiches (single code)
32202085	Egg, cheese and bacon on bagel	Lo	Mixed Dishes - Sandwiches (single code)
32202090	Egg and bacon on biscuit	Lo	Mixed Dishes - Sandwiches (single code)
32202110	Egg and ham on biscuit	Lo	Mixed Dishes - Sandwiches (single code)
32202120	Egg, cheese and sausage on bagel	Lo	Mixed Dishes - Sandwiches (single code)
32202130	Egg and steak on biscuit	Lo	Mixed Dishes - Sandwiches (single code)
32202200	Egg and cheese on biscuit	Lo	Mixed Dishes - Sandwiches (single code)
32204010	Scrambled egg sandwich	Lo	Mixed Dishes - Sandwiches (single code)
32300100	Egg drop soup	Lo	Mixed Dishes - Soups
32301100	Garlic egg soup, Puerto Rican style	Lo	Mixed Dishes - Soups
32400010	Egg white omelet or scrambled egg	Lo	Eggs
32400011	Egg white omelet or scrambled egg	Lo	Eggs
32400012	Egg white omelet or scrambled egg	Lo	Eggs
32400050	Egg white omelet or scrambled egg	Lo	Eggs
32400055	Egg white omelet, scrambled, or frie	Lo	Eggs
32400060	Egg white omelet, scrambled, or frie	Lo	Eggs
32400065	Egg white omelet, scrambled, or frie	Lo	Eggs
32400070	Egg white omelet, scrambled, or frie	Lo	Eggs

32400075	Egg white omelet, scrambled, or frie	Lo	Eggs
32400078	Egg white omelet, scrambled, or frie	Lo	Eggs
32400080	Egg white omelet, scrambled, or frie	Lo	Eggs
32400100	Egg white, omelet, scrambled, or fri	Lo	Eggs
32400110	Egg white, omelet, scrambled, or fri	Lo	Eggs
32400120	Egg white, omelet, scrambled, or fri	Lo	Eggs
32400200	Egg white, omelet, scrambled, or fri	Lo	Eggs
32400210	Egg white, omelet, scrambled, or fri	Lo	Eggs
32400220	Egg white, omelet, scrambled, or fri	Lo	Eggs
32400300	Egg white, omelet, scrambled, or fri	Lo	Eggs
32400310	Egg white, omelet, scrambled, or fri	Lo	Eggs
32400320	Egg white, omelet, scrambled, or fri	Lo	Eggs
32400400	Egg white, omelet, scrambled, or fri	Lo	Eggs
32400410	Egg white, omelet, scrambled, or fri	Lo	Eggs
32400420	Egg white, omelet, scrambled, or fri	Lo	Eggs
32400500	Egg white, omelet, scrambled, or fri	Lo	Eggs
32400510	Egg white, omelet, scrambled, or fri	Lo	Eggs
32400520	Egg white, omelet, scrambled, or fri	Lo	Eggs
32400600	Egg white, omelet, scrambled, or fri	Lo	Eggs
32400610	Egg white, omelet, scrambled, or fri	Lo	Eggs
32400620	Egg white, omelet, scrambled, or fri	Lo	Eggs
32400700	Egg white, omelet, scrambled, or fri	Lo	Eggs
32400710	Egg white, omelet, scrambled, or fri	Lo	Eggs
32400720	Egg white, omelet, scrambled, or fri	Lo	Eggs
32401000	Meringues	Lo	Other Desserts
33000100	Egg substitute, NS as to powdered,	Lo	Eggs
33000990	Egg substitute, omelet, scrambled,	Lo	Eggs
33001000	Egg substitute, omelet, scrambled,	Lo	Eggs
33001010	Egg substitute, omelet, scrambled,	Lo	Eggs
33001020	Egg substitute, omelet, scrambled,	Lo	Eggs
33001040	Egg substitute, omelet, scrambled,	Lo	Eggs
33001050	Egg substitute, omelet, scrambled,	Lo	Eggs
33001200	Egg substitute, vegetable flavored,	Lo	Eggs
33001210	Egg substitute, vegetable flavored,	Lo	Eggs
33102010	Scrambled egg, made from powder	Lo	Eggs
33201010	Scrambled egg, made from cholest	Lo	Eggs
33201110	Scrambled egg, made from cholest	Lo	Eggs
33201500	Scrambled egg, made from cholest	Lo	Eggs
33202010	Scrambled egg, made from frozen	Lo	Eggs
33301010	Scrambled egg, made from packag	Lo	Eggs
33401000	Egg substitute, omelet, scrambled,	Lo	Eggs
33401020	Egg substitute, omelet, scrambled,	Lo	Eggs
33401100	Egg substitute, omelet, scrambled,	Lo	Eggs
33401110	Egg substitute, omelet, scrambled,	Lo	Eggs
33401200	Egg substitute, omelet, scrambled,	Lo	Eggs
33401220	Egg substitute, omelet, scrambled,	Lo	Eggs
33401300	Egg substitute, omelet, scrambled,	Lo	Eggs
33401310	Egg substitute, omelet, scrambled,	Lo	Eggs
33401400	Egg substitute, omelet, scrambled,	Lo	Eggs
33401420	Egg substitute, omelet, scrambled,	Lo	Eggs
33401500	Egg substitute, omelet, scrambled,	Lo	Eggs

33401510	Egg substitute, omelet, scrambled,	Lo	Eggs
33401600	Egg substitute, omelet, scrambled,	Lo	Eggs
33401610	Egg substitute, omelet, scrambled,	Lo	Eggs
33401620	Egg substitute, omelet, scrambled,	Lo	Eggs
35001000	Scrambled eggs, sausage, hash bro	Lo	Eggs
35002000	Scrambled eggs, bacon, home fried	Lo	Eggs
41100990	Beans, NFS	Lo	Plant-based Protein Foods
41101000	Beans, dry, cooked, NS as to type a	Lo	Plant-based Protein Foods
41101010	Beans, from dried, NS as to type, fa	Lo	Plant-based Protein Foods
41101020	Beans, from dried, NS as to type, n	Lo	Plant-based Protein Foods
41101050	Beans, canned, drained, NS as to ty	Lo	Plant-based Protein Foods
41101060	Beans, from canned, NS as to type,	Lo	Plant-based Protein Foods
41101070	Beans, from canned, NS as to type,	Lo	Plant-based Protein Foods
41101080	Beans, from fast food / restaurant, I	Lo	Plant-based Protein Foods
41101090	White beans, NFS	Lo	Plant-based Protein Foods
41101100	White beans, dry, cooked, NS as to	Lo	Plant-based Protein Foods
41101110	White beans, from dried, fat added	Lo	Plant-based Protein Foods
41101111	White beans, dry, cooked, made wi	Lo	Plant-based Protein Foods
41101112	White beans, dry, cooked, made wi	Lo	Plant-based Protein Foods
41101113	White beans, dry, cooked, made wi	Lo	Plant-based Protein Foods
41101120	White beans, from dried, no added	Lo	Plant-based Protein Foods
41101140	White beans, from canned, fat adde	Lo	Plant-based Protein Foods
41101150	White beans, canned, drained, mad	Lo	Plant-based Protein Foods
41101180	White beans, from canned, no adde	Lo	Plant-based Protein Foods
41101990	Black beans, NFS	Lo	Plant-based Protein Foods
41102000	Black, brown, or Bayo beans, dry, c	Lo	Plant-based Protein Foods
41102010	Black beans, from dried, fat added	Lo	Plant-based Protein Foods
41102011	Black, brown, or Bayo beans, dry, c	Lo	Plant-based Protein Foods
41102012	Black, brown, or Bayo beans, dry, c	Lo	Plant-based Protein Foods
41102013	Black, brown, or Bayo beans, dry, c	Lo	Plant-based Protein Foods
41102020	Black beans, from dried, no added f	Lo	Plant-based Protein Foods
41102030	Black, brown, or Bayo beans, canne	Lo	Plant-based Protein Foods
41102040	Black beans, from canned, fat adde	Lo	Plant-based Protein Foods
41102050	Black, brown, or Bayo beans, canne	Lo	Plant-based Protein Foods
41102060	Black, brown, or Bayo beans, canne	Lo	Plant-based Protein Foods
41102080	Black beans, from canned, no adde	Lo	Plant-based Protein Foods
41102100	Black, brown, or Bayo beans, canne	Lo	Plant-based Protein Foods
41102110	Black beans, from canned, reduced	Lo	Plant-based Protein Foods
41102120	Black, brown, or Bayo beans, canne	Lo	Plant-based Protein Foods
41102150	Black beans, from fast food / restau	Lo	Plant-based Protein Foods
41102170	Black beans with meat	Lo	Plant-based Protein Foods
41102200	Fava beans, dry, cooked, NS as to	Lo	Plant-based Protein Foods
41102210	Fava beans, cooked	Lo	Plant-based Protein Foods
41102220	Fava beans, dry, cooked, fat not ad	Lo	Plant-based Protein Foods
41102260	Fava beans, canned, drained, fat ad	Lo	Plant-based Protein Foods
41102990	Lima beans, NFS	Lo	Plant-based Protein Foods
41103000	Lima beans, dry, cooked, NS as to	Lo	Plant-based Protein Foods
41103010	Lima beans, from dried	Lo	Plant-based Protein Foods
41103011	Lima beans, dry, cooked, made with	Lo	Plant-based Protein Foods
41103012	Lima beans, dry, cooked, made with	Lo	Plant-based Protein Foods
41103013	Lima beans, dry, cooked, made with	Lo	Plant-based Protein Foods

41103020	Lima beans, dry, cooked, fat not ad	Lo	Plant-based Protein Foods
41103050	Pink beans, dry, cooked, NS as to f	Lo	Plant-based Protein Foods
41103060	Pink beans, dry, cooked, fat not add	Lo	Plant-based Protein Foods
41103070	Pink beans, cooked	Lo	Plant-based Protein Foods
41103090	Pink beans, canned, drained, fat ad	Lo	Plant-based Protein Foods
41103100	Pink beans, canned, drained, fat no	Lo	Plant-based Protein Foods
41103990	Pinto beans, NFS	Lo	Plant-based Protein Foods
41104000	Pinto, calico, or red Mexican beans	Lo	Plant-based Protein Foods
41104010	Pinto beans, from dried, fat added	Lo	Plant-based Protein Foods
41104011	Pinto, calico, or red Mexican beans	Lo	Plant-based Protein Foods
41104012	Pinto, calico, or red Mexican beans	Lo	Plant-based Protein Foods
41104013	Pinto, calico, or red Mexican beans	Lo	Plant-based Protein Foods
41104020	Pinto beans, from dried, no added f	Lo	Plant-based Protein Foods
41104030	Pinto, calico, or red Mexican beans	Lo	Plant-based Protein Foods
41104040	Pinto beans, from canned, fat adde	Lo	Plant-based Protein Foods
41104050	Pinto, calico, or red Mexican beans	Lo	Plant-based Protein Foods
41104060	Pinto, calico, or red Mexican beans	Lo	Plant-based Protein Foods
41104080	Pinto beans, from canned, no adde	Lo	Plant-based Protein Foods
41104110	Pinto beans, from canned, reduced	Lo	Plant-based Protein Foods
41104120	Pinto, calico, or red Mexican beans	Lo	Plant-based Protein Foods
41104200	Pinto beans, from fast food / restau	Lo	Plant-based Protein Foods
41104250	Pinto beans with meat	Lo	Plant-based Protein Foods
41105990	Kidney beans, NFS	Lo	Plant-based Protein Foods
41106000	Red kidney beans, dry, cooked, NS	Lo	Plant-based Protein Foods
41106010	Kidney beans, from dried, fat addec	Lo	Plant-based Protein Foods
41106011	Red kidney beans, dry, cooked, ma	Lo	Plant-based Protein Foods
41106012	Red kidney beans, dry, cooked, ma	Lo	Plant-based Protein Foods
41106013	Red kidney beans, dry, cooked, ma	Lo	Plant-based Protein Foods
41106020	Kidney beans, from dried, no addec	Lo	Plant-based Protein Foods
41106040	Kidney beans, from canned, fat add	Lo	Plant-based Protein Foods
41106050	Red kidney beans, canned, drained	Lo	Plant-based Protein Foods
41106080	Kidney beans, from canned, no add	Lo	Plant-based Protein Foods
41106100	Red kidney beans, canned, drained	Lo	Plant-based Protein Foods
41106110	Kidney beans, from canned, reduce	Lo	Plant-based Protein Foods
41106120	Red kidney beans, canned, drained	Lo	Plant-based Protein Foods
41106150	Kidney beans, from fast food / resta	Lo	Plant-based Protein Foods
41106170	Kidney beans with meat	Lo	Plant-based Protein Foods
41106500	Yellow, canary, or Peruvian beans,	Lo	Plant-based Protein Foods
41106510	Peruvian beans, from dried	Lo	Plant-based Protein Foods
41106511	Yellow, canary, or Peruvian beans,	Lo	Plant-based Protein Foods
41106520	Yellow, canary, or Peruvian beans,	Lo	Plant-based Protein Foods
41107000	Soybeans, dry, cooked, fat not adde	Lo	Plant-based Protein Foods
41107010	Soybeans, cooked	Lo	Plant-based Protein Foods
41107020	Soybeans, dry, cooked, NS as to fa	Lo	Plant-based Protein Foods
41108000	Mung beans, dry, cooked, fat not ac	Lo	Plant-based Protein Foods
41108010	Mung beans, cooked	Lo	Plant-based Protein Foods
41108020	Mung beans, dry, cooked, NS as to	Lo	Plant-based Protein Foods
41108030	Mung beans, canned, drained, NS a	Lo	Plant-based Protein Foods
41109000	Mungo beans, cooked, fat not adde	Lo	Plant-based Protein Foods
41201010	Baked beans	Lo	Plant-based Protein Foods
41201020	Baked beans, vegetarian	Lo	Plant-based Protein Foods

41201050	Baked beans from fast food / restau	Lo	Plant-based Protein Foods
41202020	Chili beans, barbecue beans, ranch	Lo	Plant-based Protein Foods
41202500	Beans and tomatoes, NS as to fat a	Lo	Plant-based Protein Foods
41202505	Beans and tomatoes, no added fat	Lo	Plant-based Protein Foods
41202510	Beans and tomatoes, fat added	Lo	Plant-based Protein Foods
41203030	Black bean salad	Lo	Mixed Dishes - Bean/Vegetable-based
41204020	Boston baked beans	Lo	Plant-based Protein Foods
41205010	Refried beans	Lo	Plant-based Protein Foods
41205011	Refried beans, made with oil	Lo	Plant-based Protein Foods
41205012	Refried beans, made with animal fa	Lo	Plant-based Protein Foods
41205013	Refried beans, made with margarin	Lo	Plant-based Protein Foods
41205015	Refried beans, fat not added in coo	Lo	Plant-based Protein Foods
41205016	Refried beans, NS as to fat added i	Lo	Plant-based Protein Foods
41205017	Refried beans, from fast food / resta	Lo	Plant-based Protein Foods
41205020	Refried beans with cheese	Lo	Plant-based Protein Foods
41205030	Refried beans with meat	Lo	Plant-based Protein Foods
41205040	Refried beans, from canned, reduce	Lo	Plant-based Protein Foods
41205050	Bean dip, made with refried beans	Lo	Condiments and Sauces
41205055	Layer dip	Lo	Condiments and Sauces
41205070	Hummus, plain	Lo	Condiments and Sauces
41205075	Hummus, flavored	Lo	Condiments and Sauces
41205100	Black bean sauce	Lo	Condiments and Sauces
41206030	Beans and franks	Lo	Plant-based Protein Foods
41207030	Beans, dry, cooked with ground bee	Lo	Plant-based Protein Foods
41208030	Pork and beans	Lo	Plant-based Protein Foods
41208100	Beans with meat, NS as to type	Lo	Plant-based Protein Foods
41209000	Falafel	Lo	Mixed Dishes - Bean/Vegetable-based
41210000	Bean cake	Lo	Mixed Dishes - Bean/Vegetable-based
41210090	Stewed beans with pork, tomatoes,	Lo	Mixed Dishes - Bean/Vegetable-based
41210100	Stewed red beans, Puerto Rican sty	Lo	Mixed Dishes - Bean/Vegetable-based
41210150	Stewed pink beans with white potat	Lo	Mixed Dishes - Bean/Vegetable-based
41210190	Stewed red beans with pig's feet an	Lo	Mixed Dishes - Bean/Vegetable-based
41210200	Black beans, Cuban style	Lo	Plant-based Protein Foods
41221000	Baked beans, reduced sodium	Lo	Plant-based Protein Foods
41221010	Baked beans, low sodium	Lo	Plant-based Protein Foods
41221020	Chili with beans, without meat	Lo	Mixed Dishes - Bean/Vegetable-based
41300990	Blackeyed peas, NFS	Lo	Plant-based Protein Foods
41301000	Cowpeas, dry, cooked, NS as to fat	Lo	Plant-based Protein Foods
41301010	Blackeyed peas, from dried	Lo	Plant-based Protein Foods
41301020	Cowpeas, dry, cooked, fat not adde	Lo	Plant-based Protein Foods
41301990	Chickpeas, NFS	Lo	Plant-based Protein Foods
41302000	Chickpeas, dry, cooked, NS as to fa	Lo	Plant-based Protein Foods
41302010	Chickpeas, from dried, fat added	Lo	Plant-based Protein Foods
41302011	Chickpeas, dry, cooked, made with	Lo	Plant-based Protein Foods
41302012	Chickpeas, dry, cooked, made with	Lo	Plant-based Protein Foods
41302020	Chickpeas, from dried, no added fa	Lo	Plant-based Protein Foods
41302030	Chickpeas, canned, drained, NS as	Lo	Plant-based Protein Foods
41302040	Chickpeas, from canned, fat added	Lo	Plant-based Protein Foods
41302050	Chickpeas, canned, drained, made	Lo	Plant-based Protein Foods
41302080	Chickpeas, from canned, no added	Lo	Plant-based Protein Foods
41302100	Chickpeas, canned, drained, low so	Lo	Plant-based Protein Foods

41302110	Chickpeas, from canned, reduced s	Lo	Plant-based Protein Foods
41303000	Split peas, from dried, no added fat	Lo	Plant-based Protein Foods
41303010	Split peas, from dried, fat added	Lo	Plant-based Protein Foods
41303011	Green or yellow split peas, dry, coo	Lo	Plant-based Protein Foods
41303012	Green or yellow split peas, dry, coo	Lo	Plant-based Protein Foods
41303013	Green or yellow split peas, dry, coo	Lo	Plant-based Protein Foods
41303020	Green or yellow split peas, dry, coo	Lo	Plant-based Protein Foods
41304000	Wasabi peas	Lo	Plant-based Protein Foods
41304130	Cowpeas, dry, cooked with pork	Lo	Plant-based Protein Foods
41304970	Lentils, NFS	Lo	Plant-based Protein Foods
41304980	Lentils, dry, cooked, NS as to fat ad	Lo	Plant-based Protein Foods
41304990	Lentils, from dried, fat added	Lo	Plant-based Protein Foods
41304991	Lentils, dry, cooked, made with oil	Lo	Plant-based Protein Foods
41304992	Lentils, dry, cooked, made with anir	Lo	Plant-based Protein Foods
41305000	Lentils, from dried, no added fat	Lo	Plant-based Protein Foods
41305020	Lentils, from canned	Lo	Plant-based Protein Foods
41306000	Loaf, lentil	Lo	Plant-based Protein Foods
41310100	Stewed pigeon peas, Puerto Rican	Lo	Mixed Dishes - Bean/Vegetable-based
41310150	Stewed chickpeas, Puerto Rican sty	Lo	Mixed Dishes - Bean/Vegetable-based
41310160	Stewed chickpeas, with potatoes, P	Lo	Mixed Dishes - Bean/Vegetable-based
41310200	Chickpeas stewed with pig's feet, P	Lo	Mixed Dishes - Bean/Vegetable-based
41310220	Fried chickpeas with bacon, Puerto	Lo	Plant-based Protein Foods
41310900	Bean chips	Lo	Savory Snacks
41311000	Papad, grilled or broiled	Lo	Plant-based Protein Foods
41311020	Sambar, vegetable stew	Lo	Mixed Dishes - Bean/Vegetable-based
41311030	Lentil curry	Lo	Mixed Dishes - Bean/Vegetable-based
41311040	Lentil curry with rice	Lo	Mixed Dishes - Bean/Vegetable-based
41410010	Soy nuts	Lo	Plant-based Protein Foods
41410015	Soy chips	Lo	Savory Snacks
41420010	Soybean curd	Lo	Plant-based Protein Foods
41420020	Edamame, cooked	Lo	Plant-based Protein Foods
41420050	Soybean curd cheese	Lo	Plant-based Protein Foods
41420250	Hoisin sauce	Lo	Condiments and Sauces
41420300	Soy sauce	Lo	Condiments and Sauces
41420350	Soy sauce, reduced sodium	Lo	Condiments and Sauces
41420400	Teriyaki sauce	Lo	Condiments and Sauces
41420410	Teriyaki sauce, reduced sodium	Lo	Condiments and Sauces
41420450	Worcestershire sauce	Lo	Condiments and Sauces
41421010	Soybean curd, deep fried	Lo	Plant-based Protein Foods
41421020	Soybean curd, breaded, fried	Lo	Plant-based Protein Foods
41422010	Soybean meal	Lo	Plant-based Protein Foods
41425010	Vermicelli, made from soybeans	Lo	Plant-based Protein Foods
41430000	Protein powder, NFS	Lo	Other
41430010	Protein supplement, powdered	Lo	Other
41430200	Meal replacement or supplement, s	Lo	Sweetened Beverages
41430310	Protein diet powder with soy and ca	Lo	Other
41435110	High protein bar, candy-like, soy an	Lo	Snack/Meal Bars
41435120	Zone Perfect Classic Crunch nutritic	Lo	Snack/Meal Bars
41435300	Balance Original Bar	Lo	Snack/Meal Bars
41435500	Clif Bar	Lo	Snack/Meal Bars
41435700	South Beach Living High Protein Ce	Lo	Snack/Meal Bars

41435710	South Beach Living Meal Replacem	Lo	Snack/Meal Bars
41436000	Nutritional supplement for people w	Lo	Sweetened Beverages
41440000	Textured vegetable protein, dry	Lo	Plant-based Protein Foods
41440010	Ensure liquid nutrition	Lo	Sweetened Beverages
41440020	Ensure with fiber, liquid	Lo	Sweetened Beverages
41440050	Ensure Plus liquid nutrition	Lo	Sweetened Beverages
41440100	Meal replacement or supplement, li	Lo	Sweetened Beverages
41480000	Tofu, frozen dessert, flavors other t	Lo	Other Desserts
41480010	Tofu, frozen dessert, chocolate	Lo	Other Desserts
41480020	Frozen dessert, non-dairy	Lo	Plant-based Protein Foods
41601010	Bean soup, NFS	Lo	Mixed Dishes - Soups
41601020	Bean with bacon or ham soup, cann	Lo	Mixed Dishes - Soups
41601030	Black bean soup, home recipe, can	Lo	Mixed Dishes - Soups
41601040	Lima bean soup, home recipe, cann	Lo	Mixed Dishes - Soups
41601060	Bean soup, with macaroni and mea	Lo	Mixed Dishes - Soups
41601070	Soybean soup, miso broth	Lo	Mixed Dishes - Soups
41601080	Pinto bean soup, home recipe, cann	Lo	Mixed Dishes - Soups
41601090	Bean soup, with macaroni, home re	Lo	Mixed Dishes - Soups
41601100	Portuguese bean soup, home recip	Lo	Mixed Dishes - Soups
41601110	Bean and ham soup, chunky style,	Lo	Mixed Dishes - Soups
41601120	Bean soup with vegetables, rice, an	Lo	Mixed Dishes - Soups
41601130	Bean soup, mixed beans, home rec	Lo	Mixed Dishes - Soups
41601140	Bean soup, home recipe	Lo	Mixed Dishes - Soups
41601160	Bean and ham soup, canned, reduc	Lo	Mixed Dishes - Soups
41601170	Bean and rice soup	Lo	Mixed Dishes - Soups
41601180	Bean and ham soup, home recipe	Lo	Mixed Dishes - Soups
41601200	Liquid from stewed kidney beans, P	Lo	Mixed Dishes - Soups
41602010	Pea and ham soup, chunky style, ca	Lo	Mixed Dishes - Soups
41602020	Garbanzo bean or chickpea soup, h	Lo	Mixed Dishes - Soups
41602030	Split pea and ham soup	Lo	Mixed Dishes - Soups
41602050	Split pea soup	Lo	Mixed Dishes - Soups
41602070	Split pea soup, canned, reduced so	Lo	Mixed Dishes - Soups
41602090	Split pea and ham soup, canned, re	Lo	Mixed Dishes - Soups
41603010	Lentil soup, home recipe, canned, c	Lo	Mixed Dishes - Soups
41610100	White bean soup, Puerto Rican styl	Lo	Mixed Dishes - Soups
41810200	Bacon strip, meatless	Lo	Plant-based Protein Foods
41810250	Bacon bits	Lo	Cured Meats/Poultry
41810400	Breakfast link, pattie, or slice, meat	Lo	Plant-based Protein Foods
41810600	Chicken, meatless, NFS	Lo	Plant-based Protein Foods
41810610	Chicken, meatless, breaded, fried	Lo	Plant-based Protein Foods
41811200	Fish stick, meatless	Lo	Plant-based Protein Foods
41811400	Frankfurter or hot dog, meatless	Lo	Plant-based Protein Foods
41811600	Luncheon slice, meatless-beef, chic	Lo	Plant-based Protein Foods
41811800	Meatball, meatless	Lo	Plant-based Protein Foods
41811890	Vegetarian burger or patty, meatles	Lo	Plant-based Protein Foods
41811900	Soyburger, meatless, no bun	Lo	Plant-based Protein Foods
41811910	Vegetable burger or patty, meatless	Lo	Plant-based Protein Foods
41811950	Swiss steak, with gravy, meatless	Lo	Mixed Dishes - Bean/Vegetable-based
41812000	Sandwich spread, meat substitute t	Lo	Plant-based Protein Foods
41812400	Vegetarian pot pie	Lo	Mixed Dishes - Bean/Vegetable-based
41812450	Vegetarian chili, made with meat su	Lo	Mixed Dishes - Bean/Vegetable-based

41812500	Tofu and vegetables including carro	Lo	Mixed Dishes - Asian
41812510	Tofu and vegetables excluding carr	Lo	Mixed Dishes - Asian
41812600	Vegetarian, fillet	Lo	Plant-based Protein Foods
41812800	Vegetarian stew	Lo	Mixed Dishes - Bean/Vegetable-based
41812850	Vegetarian stroganoff	Lo	Mixed Dishes - Bean/Vegetable-based
41812900	Vegetarian meat loaf	Lo	Mixed Dishes - Bean/Vegetable-based
41901020	Soyburger, meatless, with cheese o	Lo	Plant-based Protein Foods
42100050	Nuts, NFS	Lo	Plant-based Protein Foods
42100100	Almonds, NFS	Lo	Plant-based Protein Foods
42101000	Almonds, unroasted	Lo	Plant-based Protein Foods
42101100	Almonds, roasted	Lo	Plant-based Protein Foods
42101110	Almonds, salted	Lo	Plant-based Protein Foods
42101120	Almonds, lightly salted	Lo	Plant-based Protein Foods
42101130	Almonds, unsalted	Lo	Plant-based Protein Foods
42101200	Almonds, dry roasted, salted	Lo	Plant-based Protein Foods
42101210	Almonds, dry roasted, without salt	Lo	Plant-based Protein Foods
42101300	Almonds, flavored	Lo	Plant-based Protein Foods
42101350	Almonds, honey roasted	Lo	Plant-based Protein Foods
42102000	Brazil nuts	Lo	Plant-based Protein Foods
42104000	Cashews, NFS	Lo	Plant-based Protein Foods
42104050	Cashews, unroasted	Lo	Plant-based Protein Foods
42104100	Cashews, salted	Lo	Plant-based Protein Foods
42104105	Cashews, lightly salted	Lo	Plant-based Protein Foods
42104110	Cashews, unsalted	Lo	Plant-based Protein Foods
42104200	Cashew nuts, dry roasted, salted	Lo	Plant-based Protein Foods
42104205	Cashew nuts, dry roasted, without s	Lo	Plant-based Protein Foods
42104500	Cashews, honey roasted	Lo	Plant-based Protein Foods
42105000	Chestnuts	Lo	Plant-based Protein Foods
42106000	Coconut, fresh	Lo	Plant-based Protein Foods
42106020	Coconut, packaged	Lo	Plant-based Protein Foods
42107000	Hazelnuts	Lo	Plant-based Protein Foods
42109000	Macadamia nuts, unroasted	Lo	Plant-based Protein Foods
42109100	Macadamia nuts	Lo	Plant-based Protein Foods
42109105	Macadamia nuts, roasted, without s	Lo	Plant-based Protein Foods
42110000	Mixed nuts, NFS	Lo	Plant-based Protein Foods
42110015	Mixed nuts, salted	Lo	Plant-based Protein Foods
42110020	Mixed nuts, without salt	Lo	Plant-based Protein Foods
42110050	Mixed nuts, unroasted	Lo	Plant-based Protein Foods
42110100	Mixed nuts, with peanuts, salted	Lo	Plant-based Protein Foods
42110110	Mixed nuts, with peanuts, lightly sal	Lo	Plant-based Protein Foods
42110120	Mixed nuts, with peanuts, unsalted	Lo	Plant-based Protein Foods
42110150	Mixed nuts, without peanuts, salted	Lo	Plant-based Protein Foods
42110160	Mixed nuts, without peanuts, unsalt	Lo	Plant-based Protein Foods
42110200	Mixed nuts, dry roasted	Lo	Plant-based Protein Foods
42110300	Mixed nuts, honey roasted	Lo	Plant-based Protein Foods
42110500	Mixed nuts, in shell	Lo	Plant-based Protein Foods
42111000	Peanuts, NFS	Lo	Plant-based Protein Foods
42111010	Peanuts, in shell, NFS (shell not ea	Lo	Plant-based Protein Foods
42111030	Peanuts, boiled	Lo	Plant-based Protein Foods
42111040	Peanuts, unroasted	Lo	Plant-based Protein Foods
42111100	Peanuts, roasted, salted	Lo	Plant-based Protein Foods

42111110	Peanuts, roasted, unsalted	Lo	Plant-based Protein Foods
42111200	Peanuts, dry roasted, salted	Lo	Plant-based Protein Foods
42111205	Peanuts, dry roasted, lightly salted	Lo	Plant-based Protein Foods
42111210	Peanuts, dry roasted, unsalted	Lo	Plant-based Protein Foods
42111500	Peanuts, honey roasted	Lo	Plant-based Protein Foods
42112000	Pecans, NFS	Lo	Plant-based Protein Foods
42112100	Pecans, unroasted	Lo	Plant-based Protein Foods
42112200	Pecans, salted	Lo	Plant-based Protein Foods
42112210	Pecans, unsalted	Lo	Plant-based Protein Foods
42112300	Pecans, honey roasted	Lo	Plant-based Protein Foods
42113000	Pine nuts	Lo	Plant-based Protein Foods
42114130	Pistachio nuts, NFS	Lo	Plant-based Protein Foods
42114140	Pistachio nuts, salted	Lo	Plant-based Protein Foods
42114142	Pistachio nuts, lightly salted	Lo	Plant-based Protein Foods
42114145	Pistachio nuts, unsalted	Lo	Plant-based Protein Foods
42116000	Walnuts, excluding honey roasted	Lo	Plant-based Protein Foods
42116050	Walnuts	Lo	Plant-based Protein Foods
42116055	Walnuts, roasted, without salt	Lo	Plant-based Protein Foods
42116100	Walnuts, honey roasted	Lo	Plant-based Protein Foods
42200500	Almond butter	Lo	Plant-based Protein Foods
42200510	Almond butter, lower sodium	Lo	Plant-based Protein Foods
42200600	Almond paste	Lo	Plant-based Protein Foods
42201000	Cashew butter	Lo	Plant-based Protein Foods
42202000	Peanut butter	Lo	Plant-based Protein Foods
42202010	Peanut butter, lower sodium	Lo	Plant-based Protein Foods
42202100	Peanut butter, lower sodium and lo	Lo	Plant-based Protein Foods
42202130	Peanut butter, lower sugar	Lo	Plant-based Protein Foods
42202150	Peanut butter, reduced fat	Lo	Plant-based Protein Foods
42202200	Peanut butter, vitamin and mineral	Lo	Plant-based Protein Foods
42203000	Peanut butter and jelly	Lo	Plant-based Protein Foods
42203100	Peanut butter and chocolate spread	Lo	Plant-based Protein Foods
42203200	Soy nut butter	Lo	Plant-based Protein Foods
42204050	Peanut sauce	Lo	Condiments and Sauces
42204100	Gravy, vegetarian	Lo	Condiments and Sauces
42301010	Peanut butter sandwich, NFS	Lo	Mixed Dishes - Sandwiches (single code)
42301015	Peanut butter sandwich, with regula	Lo	Mixed Dishes - Sandwiches (single code)
42301020	Peanut butter sandwich, with regula	Lo	Mixed Dishes - Sandwiches (single code)
42301025	Peanut butter sandwich, with regula	Lo	Mixed Dishes - Sandwiches (single code)
42301115	Peanut butter sandwich, with reduc	Lo	Mixed Dishes - Sandwiches (single code)
42301120	Peanut butter sandwich, with reduc	Lo	Mixed Dishes - Sandwiches (single code)
42301125	Peanut butter sandwich, with reduc	Lo	Mixed Dishes - Sandwiches (single code)
42302010	Peanut butter and jelly sandwich, N	Lo	Mixed Dishes - Sandwiches (single code)
42302015	Peanut butter and jelly sandwich, w	Lo	Mixed Dishes - Sandwiches (single code)
42302020	Peanut butter and jelly sandwich, w	Lo	Mixed Dishes - Sandwiches (single code)
42302025	Peanut butter and jelly sandwich, w	Lo	Mixed Dishes - Sandwiches (single code)
42302055	Peanut butter and jelly sandwich, w	Lo	Mixed Dishes - Sandwiches (single code)
42302060	Peanut butter and jelly sandwich, w	Lo	Mixed Dishes - Sandwiches (single code)
42302065	Peanut butter and jelly sandwich, w	Lo	Mixed Dishes - Sandwiches (single code)
42302105	Peanut butter and jelly sandwich, w	Lo	Mixed Dishes - Sandwiches (single code)
42302110	Peanut butter and jelly sandwich, w	Lo	Mixed Dishes - Sandwiches (single code)
42302115	Peanut butter and jelly sandwich, w	Lo	Mixed Dishes - Sandwiches (single code)

42302155	Peanut butter and jelly sandwich, w	Lo	Mixed Dishes - Sandwiches (single code)
42302160	Peanut butter and jelly sandwich, w	Lo	Mixed Dishes - Sandwiches (single code)
42302165	Peanut butter and jelly sandwich, w	Lo	Mixed Dishes - Sandwiches (single code)
42303100	Peanut butter and jelly sandwich, fr	Lo	Mixed Dishes - Sandwiches (single code)
42401010	Coconut milk, used in cooking	Lo	Dairy Drinks and Substitutes
42401100	Yogurt, coconut milk	Lo	Yogurt
42402010	Coconut cream, canned, sweetened	Lo	Dairy Drinks and Substitutes
42403010	Coconut water, unsweetened	Lo	Sweetened Beverages
42404010	Coconut water, sweetened	Lo	Sweetened Beverages
42502000	Nut mixture with seeds	Lo	Plant-based Protein Foods
42502100	Trail mix with pretzels, cereal, or gr	Lo	Plant-based Protein Foods
43101000	Pumpkin and/or squash seeds, hull	Lo	Plant-based Protein Foods
43101050	Pumpkin seeds, NFS	Lo	Plant-based Protein Foods
43101100	Pumpkin seeds, salted	Lo	Plant-based Protein Foods
43101150	Pumpkin seeds, unsalted	Lo	Plant-based Protein Foods
43102000	Sunflower seeds, plain, unsalted	Lo	Plant-based Protein Foods
43102100	Sunflower seeds, plain, salted	Lo	Plant-based Protein Foods
43102110	Sunflower seeds, hulled, roasted, w	Lo	Plant-based Protein Foods
43102200	Sunflower seeds, hulled, dry roaste	Lo	Plant-based Protein Foods
43102300	Sunflower seeds, flavored	Lo	Plant-based Protein Foods
43102400	Sunflower seeds, NFS	Lo	Plant-based Protein Foods
43103000	Sesame seeds	Lo	Plant-based Protein Foods
43103050	Sesame seeds, whole seed	Lo	Plant-based Protein Foods
43103100	Sesame sauce	Lo	Condiments and Sauces
43104000	Flax seeds	Lo	Plant-based Protein Foods
43105200	Psyllium seed, husks	Lo	Plant-based Protein Foods
43107000	Mixed seeds	Lo	Plant-based Protein Foods
43108010	Chia seeds	Lo	Plant-based Protein Foods
44201000	Carob chips	Lo	Candy
44202000	Carob syrup	Lo	Sugars
50010000	Flour, white	Lo	Other
51000100	Bread, NS as to major flour	Lo	Breads, Rolls, Tortillas
51000110	Bread, NS as to major flour, toasted	Lo	Breads, Rolls, Tortillas
51000180	Bread, made from home recipe or p	Lo	Breads, Rolls, Tortillas
51000190	Bread, made from home recipe or p	Lo	Breads, Rolls, Tortillas
51000200	Roll, NS as to major flour	Lo	Breads, Rolls, Tortillas
51000230	Roll, NS as to major flour, toasted	Lo	Breads, Rolls, Tortillas
51000250	Roll, made from home recipe or pu	Lo	Breads, Rolls, Tortillas
51000300	Roll, hard, NS as to major flour	Lo	Breads, Rolls, Tortillas
51000400	Roll, bran, NS as to type of bran	Lo	Breads, Rolls, Tortillas
51101000	Bread, white	Lo	Breads, Rolls, Tortillas
51101010	Bread, white, toasted	Lo	Breads, Rolls, Tortillas
51101050	Bread, white, made from home reci	Lo	Breads, Rolls, Tortillas
51101060	Bread, white, made from home reci	Lo	Breads, Rolls, Tortillas
51102010	Bread, white with whole wheat swirl	Lo	Breads, Rolls, Tortillas
51102020	Bread, white with whole wheat swirl	Lo	Breads, Rolls, Tortillas
51105010	Bread, Cuban	Lo	Breads, Rolls, Tortillas
51105040	Bread, Cuban, toasted	Lo	Breads, Rolls, Tortillas
51106010	Bread, native, water, Puerto Rican s	Lo	Breads, Rolls, Tortillas
51107010	Bread, French or Vienna	Lo	Breads, Rolls, Tortillas
51107040	Bread, French or Vienna, toasted	Lo	Breads, Rolls, Tortillas

51108010	Focaccia, Italian flatbread, plain	Lo	Breads, Rolls, Tortillas
51108100	Naan, Indian flatbread	Lo	Breads, Rolls, Tortillas
51109010	Bread, Italian, Grecian, Armenian	Lo	Breads, Rolls, Tortillas
51109040	Bread, Italian, Grecian, Armenian, t	Lo	Breads, Rolls, Tortillas
51109100	Bread, pita	Lo	Breads, Rolls, Tortillas
51109110	Bread, pita, toasted	Lo	Breads, Rolls, Tortillas
51109150	Bread, pita with fruit	Lo	Breads, Rolls, Tortillas
51110010	Bread, batter	Lo	Breads, Rolls, Tortillas
51111010	Bread, cheese	Lo	Breads, Rolls, Tortillas
51111040	Bread, cheese, toasted	Lo	Breads, Rolls, Tortillas
51113010	Bread, cinnamon	Lo	Breads, Rolls, Tortillas
51113100	Bread, cinnamon, toasted	Lo	Breads, Rolls, Tortillas
51115010	Bread, cornmeal and molasses	Lo	Breads, Rolls, Tortillas
51115020	Bread, cornmeal and molasses, toa	Lo	Breads, Rolls, Tortillas
51119010	Bread, egg, Challah	Lo	Breads, Rolls, Tortillas
51119040	Bread, egg, Challah, toasted	Lo	Breads, Rolls, Tortillas
51119100	Bread, lowfat, 98% fat free	Lo	Breads, Rolls, Tortillas
51121010	Bread, garlic	Lo	Breads, Rolls, Tortillas
51121015	Garlic bread, NFS	Lo	Breads, Rolls, Tortillas
51121025	Garlic bread, from fast food / restau	Lo	Breads, Rolls, Tortillas
51121035	Garlic bread, from frozen	Lo	Breads, Rolls, Tortillas
51121040	Bread, garlic, toasted	Lo	Breads, Rolls, Tortillas
51121045	Garlic bread, with parmesan chees	Lo	Breads, Rolls, Tortillas
51121055	Garlic bread, with parmesan chees	Lo	Breads, Rolls, Tortillas
51121065	Garlic bread, with melted cheese, fr	Lo	Breads, Rolls, Tortillas
51121075	Garlic bread, with melted cheese, fr	Lo	Breads, Rolls, Tortillas
51121110	Bread, onion	Lo	Breads, Rolls, Tortillas
51122000	Bread, reduced calorie and/or high	Lo	Breads, Rolls, Tortillas
51122010	Bread, reduced calorie and/or high	Lo	Breads, Rolls, Tortillas
51122050	Bread, reduced calorie and/or high	Lo	Breads, Rolls, Tortillas
51122100	Bread, reduced calorie and/or high	Lo	Breads, Rolls, Tortillas
51122110	Bread, reduced calorie and/or high	Lo	Breads, Rolls, Tortillas
51122300	Bread, white, special formula, adde	Lo	Breads, Rolls, Tortillas
51123010	Bread, high protein	Lo	Breads, Rolls, Tortillas
51123020	Bread, high protein, toasted	Lo	Breads, Rolls, Tortillas
51126010	Bread, milk and honey	Lo	Breads, Rolls, Tortillas
51126020	Bread, milk and honey, toasted	Lo	Breads, Rolls, Tortillas
51127010	Bread, potato	Lo	Breads, Rolls, Tortillas
51127020	Bread, potato, toasted	Lo	Breads, Rolls, Tortillas
51129010	Bread, raisin	Lo	Breads, Rolls, Tortillas
51129020	Bread, raisin, toasted	Lo	Breads, Rolls, Tortillas
51133010	Bread, sour dough	Lo	Breads, Rolls, Tortillas
51133020	Bread, sour dough, toasted	Lo	Breads, Rolls, Tortillas
51134000	Bread, sweet potato	Lo	Breads, Rolls, Tortillas
51135000	Bread, vegetable	Lo	Breads, Rolls, Tortillas
51135010	Bread, vegetable, toasted	Lo	Breads, Rolls, Tortillas
51136000	Bruschetta	Lo	Mixed Dishes - Sandwiches (single code)
51140100	Bread, dough, fried	Lo	Breads, Rolls, Tortillas
51150000	Roll, white, soft	Lo	Breads, Rolls, Tortillas
51150100	Roll, white, soft, toasted	Lo	Breads, Rolls, Tortillas
51151060	Roll, white, soft, made from home r	Lo	Breads, Rolls, Tortillas

51152000	Roll, white, soft, reduced calorie and	Lo	Breads, Rolls, Tortillas
51153000	Roll, white, hard	Lo	Breads, Rolls, Tortillas
51153010	Roll, white, hard, toasted	Lo	Breads, Rolls, Tortillas
51154010	Roll, white, hot dog bun	Lo	Breads, Rolls, Tortillas
51154100	Roll, white, hamburger bun	Lo	Breads, Rolls, Tortillas
51154510	Roll, diet	Lo	Breads, Rolls, Tortillas
51154550	Roll, egg bread	Lo	Breads, Rolls, Tortillas
51154600	Roll, cheese	Lo	Breads, Rolls, Tortillas
51155000	Roll, French or Vienna	Lo	Breads, Rolls, Tortillas
51155010	Roll, French or Vienna, toasted	Lo	Breads, Rolls, Tortillas
51156500	Roll, garlic	Lo	Breads, Rolls, Tortillas
51157000	Roll, white, hoagie, submarine	Lo	Breads, Rolls, Tortillas
51158100	Roll, Mexican, bolillo	Lo	Breads, Rolls, Tortillas
51159000	Roll, sour dough	Lo	Breads, Rolls, Tortillas
51160000	Roll, sweet, no frosting	Lo	Sweet Bakery Products
51160010	Roll, sweet, toasted	Lo	Sweet Bakery Products
51160100	Roll, sweet, cinnamon bun, no frost	Lo	Sweet Bakery Products
51160110	Roll, sweet, cinnamon bun, frosted	Lo	Sweet Bakery Products
51161000	Pan Dulce, with fruit, no frosting	Lo	Sweet Bakery Products
51161020	Roll, sweet, with fruit, frosted	Lo	Sweet Bakery Products
51161030	Roll, sweet, with fruit, frosted, diet	Lo	Sweet Bakery Products
51161050	Roll, sweet, frosted	Lo	Sweet Bakery Products
51161070	Roll, sweet, with fruit, frosted, fat fre	Lo	Sweet Bakery Products
51161100	Roll, sweet, with fruit and nuts, no fr	Lo	Sweet Bakery Products
51161150	Roll, sweet, with fruit and nuts, frost	Lo	Sweet Bakery Products
51161200	Roll, sweet, with nuts, no frosting	Lo	Sweet Bakery Products
51161250	Pan Dulce, no topping	Lo	Sweet Bakery Products
51161260	Roll, sweet, crumb topping, Mexican	Lo	Sweet Bakery Products
51161270	Pan Dulce, with sugar topping	Lo	Sweet Bakery Products
51161280	Pan Dulce, with raisins and icing	Lo	Sweet Bakery Products
51165000	Coffee cake, yeast type	Lo	Sweet Bakery Products
51165060	Coffee cake, yeast type, made from	Lo	Sweet Bakery Products
51165100	Coffee cake, yeast type, fat free, ch	Lo	Sweet Bakery Products
51166000	Croissant	Lo	Sweet Bakery Products
51166100	Croissant, cheese	Lo	Sweet Bakery Products
51166200	Croissant, chocolate	Lo	Sweet Bakery Products
51166500	Croissant, fruit	Lo	Sweet Bakery Products
51167000	Brioche	Lo	Sweet Bakery Products
51168000	Bread, Spanish coffee	Lo	Sweet Bakery Products
51180010	Bagel	Lo	Breads, Rolls, Tortillas
51180020	Bagel, toasted	Lo	Breads, Rolls, Tortillas
51180030	Bagel, with raisins	Lo	Breads, Rolls, Tortillas
51180040	Bagel, with raisins, toasted	Lo	Breads, Rolls, Tortillas
51180080	Bagel, with fruit other than raisins	Lo	Breads, Rolls, Tortillas
51180090	Bagel, with fruit other than raisins, t	Lo	Breads, Rolls, Tortillas
51182010	Bread stuffing	Lo	Mixed Dishes - Grain-based
51182020	Bread stuffing made with egg	Lo	Mixed Dishes - Grain-based
51183990	Breadsticks, NFS	Lo	Breads, Rolls, Tortillas
51184000	Breadsticks, hard, NFS	Lo	Crackers
51184010	Bread stick, soft	Lo	Breads, Rolls, Tortillas
51184020	Bread stick, NS as to hard or soft	Lo	Breads, Rolls, Tortillas

51184030	Bread stick, soft, prepared with garl	Lo	Breads, Rolls, Tortillas
51184100	Breadsticks, hard, reduced sodium	Lo	Crackers
51184200	Breadsticks, soft, NFS	Lo	Breads, Rolls, Tortillas
51184210	Breadsticks, soft, from fast food / re	Lo	Breads, Rolls, Tortillas
51184220	Breadsticks, soft, from frozen	Lo	Breads, Rolls, Tortillas
51184230	Breadsticks, soft, with parmesan ch	Lo	Breads, Rolls, Tortillas
51184240	Breadsticks, soft, with parmesan ch	Lo	Breads, Rolls, Tortillas
51184250	Breadsticks, soft, topped with melte	Lo	Breads, Rolls, Tortillas
51184260	Breadsticks, soft, stuffed with melte	Lo	Breads, Rolls, Tortillas
51185000	Croutons	Lo	Crackers
51186010	Muffin, English	Lo	Breads, Rolls, Tortillas
51186020	Muffin, English, toasted	Lo	Breads, Rolls, Tortillas
51186100	Muffin, English, with raisins	Lo	Breads, Rolls, Tortillas
51186120	Muffin, English, with raisins, toasted	Lo	Breads, Rolls, Tortillas
51186160	Muffin, English, with fruit other than	Lo	Breads, Rolls, Tortillas
51187000	Melba toast	Lo	Crackers
51187020	Anisette toast	Lo	Sweet Bakery Products
51188100	Pannetone	Lo	Sweet Bakery Products
51188500	Zwieback toast	Lo	Crackers
51201010	Bread, whole wheat, 100%	Lo	Breads, Rolls, Tortillas
51201020	Bread, whole wheat, 100%, toasted	Lo	Breads, Rolls, Tortillas
51201060	Bread, whole wheat, 100%, made fr	Lo	Breads, Rolls, Tortillas
51201070	Bread, whole wheat, 100%, made fr	Lo	Breads, Rolls, Tortillas
51201110	Bread, whole wheat, 100%, with rai	Lo	Breads, Rolls, Tortillas
51201120	Bread, whole wheat, 100%, with rai	Lo	Breads, Rolls, Tortillas
51201150	Bread, pita, whole wheat, 100%	Lo	Breads, Rolls, Tortillas
51202000	Muffin, English, whole wheat, 100%	Lo	Breads, Rolls, Tortillas
51204010	Bread, wheat germ	Lo	Breads, Rolls, Tortillas
51207010	Bread, sprouted wheat	Lo	Breads, Rolls, Tortillas
51207020	Bread, sprouted wheat, toasted	Lo	Breads, Rolls, Tortillas
51208000	Bagel, whole wheat, 100%	Lo	Breads, Rolls, Tortillas
51208010	Bagel, whole wheat, 100%, toasted	Lo	Breads, Rolls, Tortillas
51208100	Bagel, whole wheat, 100%, with rais	Lo	Breads, Rolls, Tortillas
51208110	Bagel, whole wheat, 100%, with rais	Lo	Breads, Rolls, Tortillas
51220000	Roll, whole wheat, 100%	Lo	Breads, Rolls, Tortillas
51300050	Bread, whole grain white	Lo	Breads, Rolls, Tortillas
51300060	Bread, whole grain white, toasted	Lo	Breads, Rolls, Tortillas
51300100	Bagel, whole grain white	Lo	Breads, Rolls, Tortillas
51300110	Bread, whole wheat	Lo	Breads, Rolls, Tortillas
51300120	Bread, whole wheat, toasted	Lo	Breads, Rolls, Tortillas
51300140	Bread, whole wheat, made from hon	Lo	Breads, Rolls, Tortillas
51300150	Bread, whole wheat, made from hon	Lo	Breads, Rolls, Tortillas
51300175	Bread, chappatti or roti, wheat	Lo	Breads, Rolls, Tortillas
51300180	Bread, puri, wheat	Lo	Breads, Rolls, Tortillas
51300185	Bread, paratha, wheat	Lo	Breads, Rolls, Tortillas
51300210	Bread, whole wheat, with raisins	Lo	Breads, Rolls, Tortillas
51300220	Bread, whole wheat, with raisins, to	Lo	Breads, Rolls, Tortillas
51300300	Bread, sprouted wheat	Lo	Breads, Rolls, Tortillas
51300310	Bread, sprouted wheat, toasted	Lo	Breads, Rolls, Tortillas
51301010	Bread, wheat or cracked wheat	Lo	Breads, Rolls, Tortillas
51301020	Bread, wheat or cracked wheat, toa	Lo	Breads, Rolls, Tortillas

51301040	Bread, wheat or cracked wheat, ma	Lo	Breads, Rolls, Tortillas
51301050	Bread, wheat or cracked wheat, ma	Lo	Breads, Rolls, Tortillas
51301120	Bread, wheat or cracked wheat, wit	Lo	Breads, Rolls, Tortillas
51301130	Bread, wheat or cracked wheat, wit	Lo	Breads, Rolls, Tortillas
51301510	Bread, wheat or cracked wheat, red	Lo	Breads, Rolls, Tortillas
51301520	Bread, wheat or cracked wheat, red	Lo	Breads, Rolls, Tortillas
51301540	Bread, French or Vienna, whole wh	Lo	Breads, Rolls, Tortillas
51301550	Bread, French or Vienna, whole wh	Lo	Breads, Rolls, Tortillas
51301600	Bread, pita, whole wheat	Lo	Breads, Rolls, Tortillas
51301610	Bread, pita, whole wheat, toasted	Lo	Breads, Rolls, Tortillas
51301620	Bread, pita, wheat or cracked whea	Lo	Breads, Rolls, Tortillas
51301630	Bread, pita, wheat or cracked whea	Lo	Breads, Rolls, Tortillas
51301700	Bagel, wheat	Lo	Breads, Rolls, Tortillas
51301710	Bagel, wheat, toasted	Lo	Breads, Rolls, Tortillas
51301750	Bagel, whole wheat	Lo	Breads, Rolls, Tortillas
51301760	Bagel, whole wheat, NS as to 100%	Lo	Breads, Rolls, Tortillas
51301800	Bagel, wheat, with raisins	Lo	Breads, Rolls, Tortillas
51301805	Bagel, whole wheat, with raisins	Lo	Breads, Rolls, Tortillas
51301820	Bagel, wheat, with fruit and nuts	Lo	Breads, Rolls, Tortillas
51301830	Bagel, wheat, with fruit and nuts, to	Lo	Breads, Rolls, Tortillas
51301900	Bagel, wheat bran	Lo	Breads, Rolls, Tortillas
51302010	Bread, wheat bran	Lo	Breads, Rolls, Tortillas
51302020	Bread, wheat bran, toasted	Lo	Breads, Rolls, Tortillas
51302050	Bread, wheat bran, with raisins	Lo	Breads, Rolls, Tortillas
51302060	Bread, wheat bran, with raisins, toa	Lo	Breads, Rolls, Tortillas
51302500	Muffin, English, wheat bran	Lo	Breads, Rolls, Tortillas
51302520	Muffin, English, wheat bran, with ra	Lo	Breads, Rolls, Tortillas
51303010	Muffin, English, wheat or cracked w	Lo	Breads, Rolls, Tortillas
51303020	Muffin, English, wheat or cracked w	Lo	Breads, Rolls, Tortillas
51303030	Muffin, English, whole wheat	Lo	Breads, Rolls, Tortillas
51303040	Muffin, English, whole wheat, NS as	Lo	Breads, Rolls, Tortillas
51303050	Muffin, English, wheat or cracked w	Lo	Breads, Rolls, Tortillas
51303070	Muffin, English, whole wheat, with r	Lo	Breads, Rolls, Tortillas
51303100	Muffin, English, whole grain white	Lo	Breads, Rolls, Tortillas
51306000	Breadsticks, hard, whole wheat	Lo	Crackers
51320010	Roll, wheat or cracked wheat	Lo	Breads, Rolls, Tortillas
51320020	Roll, wheat or cracked wheat, toast	Lo	Breads, Rolls, Tortillas
51320040	Roll, wheat or cracked wheat, made	Lo	Breads, Rolls, Tortillas
51320060	Roll, wheat or cracked wheat, hot d	Lo	Breads, Rolls, Tortillas
51320070	Roll, wheat or cracked wheat, hamb	Lo	Breads, Rolls, Tortillas
51320500	Roll, whole wheat	Lo	Breads, Rolls, Tortillas
51320510	Roll, whole wheat, NS as to 100%,	Lo	Breads, Rolls, Tortillas
51320530	Roll, whole wheat, NS as to 100%,	Lo	Breads, Rolls, Tortillas
51320550	Roll, whole wheat, hot dog bun	Lo	Breads, Rolls, Tortillas
51320560	Roll, whole wheat, hamburger bun	Lo	Breads, Rolls, Tortillas
51320700	Roll, whole grain white	Lo	Breads, Rolls, Tortillas
51320710	Roll, whole grain white, hot dog bun	Lo	Breads, Rolls, Tortillas
51320720	Roll, whole grain white, hamburger	Lo	Breads, Rolls, Tortillas
51401010	Bread, rye	Lo	Breads, Rolls, Tortillas
51401020	Bread, rye, toasted	Lo	Breads, Rolls, Tortillas
51401030	Bread, marble rye and pumpernicke	Lo	Breads, Rolls, Tortillas

51401040	Bread, marble rye and pumpernicke	Lo	Breads, Rolls, Tortillas
51401060	Bread, rye, reduced calorie and/or h	Lo	Breads, Rolls, Tortillas
51401070	Bread, rye, reduced calorie and/or h	Lo	Breads, Rolls, Tortillas
51404010	Bread, pumpernickel	Lo	Breads, Rolls, Tortillas
51404020	Bread, pumpernickel, toasted	Lo	Breads, Rolls, Tortillas
51404500	Bagel, pumpernickel	Lo	Breads, Rolls, Tortillas
51404550	Muffin, English, pumpernickel	Lo	Breads, Rolls, Tortillas
51407010	Bread, black	Lo	Breads, Rolls, Tortillas
51407020	Bread, black, toasted	Lo	Breads, Rolls, Tortillas
51420000	Roll, rye	Lo	Breads, Rolls, Tortillas
51421000	Roll, pumpernickel	Lo	Breads, Rolls, Tortillas
51501010	Bread, oatmeal	Lo	Breads, Rolls, Tortillas
51501020	Bread, oatmeal, toasted	Lo	Breads, Rolls, Tortillas
51501040	Bread, oat bran	Lo	Breads, Rolls, Tortillas
51501050	Bread, oat bran, toasted	Lo	Breads, Rolls, Tortillas
51501060	Bread, oat bran, reduced calorie an	Lo	Breads, Rolls, Tortillas
51501070	Bread, oat bran, reduced calorie an	Lo	Breads, Rolls, Tortillas
51501080	Bagel, oat bran	Lo	Breads, Rolls, Tortillas
51502010	Roll, oatmeal	Lo	Breads, Rolls, Tortillas
51502100	Roll, oat bran	Lo	Breads, Rolls, Tortillas
51503000	Muffin, English, oat bran	Lo	Breads, Rolls, Tortillas
51601010	Bread, multigrain, toasted	Lo	Breads, Rolls, Tortillas
51601020	Bread, multigrain	Lo	Breads, Rolls, Tortillas
51601210	Bread, multigrain, with raisins	Lo	Breads, Rolls, Tortillas
51601220	Bread, multigrain, with raisins, toast	Lo	Breads, Rolls, Tortillas
51602010	Bread, multigrain, reduced calorie a	Lo	Breads, Rolls, Tortillas
51602020	Bread, multigrain, reduced calorie a	Lo	Breads, Rolls, Tortillas
51620000	Roll, multigrain	Lo	Breads, Rolls, Tortillas
51620020	Roll, multigrain, hot dog bun	Lo	Breads, Rolls, Tortillas
51620030	Roll, multigrain, hamburger bun	Lo	Breads, Rolls, Tortillas
51630000	Bagel, multigrain	Lo	Breads, Rolls, Tortillas
51630100	Bagel, multigrain, with raisins	Lo	Breads, Rolls, Tortillas
51630110	Bagel, multigrain, with raisins, toast	Lo	Breads, Rolls, Tortillas
51630200	Muffin, English, multigrain	Lo	Breads, Rolls, Tortillas
51801010	Bread, barley	Lo	Breads, Rolls, Tortillas
51801020	Bread, barley, toasted	Lo	Breads, Rolls, Tortillas
51804010	Bread, soy	Lo	Breads, Rolls, Tortillas
51804020	Bread, soy, toasted	Lo	Breads, Rolls, Tortillas
51805010	Bread, sunflower meal	Lo	Breads, Rolls, Tortillas
51805020	Bread, sunflower meal, toasted	Lo	Breads, Rolls, Tortillas
51806010	Bread, rice	Lo	Breads, Rolls, Tortillas
51806020	Bread, rice, toasted	Lo	Breads, Rolls, Tortillas
51807000	Injera, Ethiopian bread	Lo	Breads, Rolls, Tortillas
51808000	Bread, gluten free	Lo	Breads, Rolls, Tortillas
51808010	Bread, gluten free, toasted	Lo	Breads, Rolls, Tortillas
51808050	Breadsticks, hard, gluten free	Lo	Crackers
51808100	Roll, gluten free	Lo	Breads, Rolls, Tortillas
52101000	Biscuit, NFS	Lo	Quick Breads and Bread Products
52101030	Biscuit dough, fried	Lo	Quick Breads and Bread Products
52101040	Crumpet	Lo	Quick Breads and Bread Products
52101100	Biscuit, baking powder or buttermilk	Lo	Quick Breads and Bread Products

52101150	Biscuit, baking powder or buttermilk	Lo	Quick Breads and Bread Products
52102040	Biscuit, from refrigerated dough	Lo	Quick Breads and Bread Products
52103000	Biscuit, from fast food / restaurant	Lo	Quick Breads and Bread Products
52104010	Biscuit, home recipe	Lo	Quick Breads and Bread Products
52104040	Biscuit, wheat	Lo	Quick Breads and Bread Products
52104100	Biscuit, cheese	Lo	Quick Breads and Bread Products
52104200	Biscuit with fruit	Lo	Quick Breads and Bread Products
52105100	Scone	Lo	Quick Breads and Bread Products
52105200	Scone, with fruit	Lo	Quick Breads and Bread Products
52201000	Cornbread, prepared from mix	Lo	Quick Breads and Bread Products
52202060	Cornbread, made from home recipe	Lo	Quick Breads and Bread Products
52204000	Cornbread stuffing	Lo	Mixed Dishes - Grain-based
52206010	Cornbread muffin, stick, round	Lo	Quick Breads and Bread Products
52206060	Cornbread muffin, stick, round, mad	Lo	Quick Breads and Bread Products
52207010	Corn flour patty or tart, fried	Lo	Quick Breads and Bread Products
52208010	Corn pone, baked	Lo	Quick Breads and Bread Products
52208020	Corn pone, fried	Lo	Quick Breads and Bread Products
52208750	Gordita/sope shell, plain, no filling, g	Lo	Breads, Rolls, Tortillas
52208760	Gordita/sope shell, plain, no filling	Lo	Breads, Rolls, Tortillas
52209010	Hush puppy	Lo	Quick Breads and Bread Products
52215000	Tortilla, NFS	Lo	Breads, Rolls, Tortillas
52215100	Tortilla, corn	Lo	Breads, Rolls, Tortillas
52215200	Tortilla, flour	Lo	Breads, Rolls, Tortillas
52215260	Tortilla, whole wheat	Lo	Breads, Rolls, Tortillas
52215300	Taco shell, corn	Lo	Breads, Rolls, Tortillas
52215350	Taco shell, flour	Lo	Breads, Rolls, Tortillas
52220110	Arepa Dominicana	Lo	Quick Breads and Bread Products
52301000	Muffin, NFS	Lo	Quick Breads and Bread Products
52302010	Muffin, fruit	Lo	Quick Breads and Bread Products
52302020	Muffin, fruit, low fat	Lo	Quick Breads and Bread Products
52302100	Muffin, fruit, fat free, cholesterol free	Lo	Quick Breads and Bread Products
52302500	Muffin, chocolate chip	Lo	Quick Breads and Bread Products
52302600	Muffin, chocolate	Lo	Quick Breads and Bread Products
52302610	Muffin, chocolate, lowfat	Lo	Quick Breads and Bread Products
52303010	Muffin, whole wheat	Lo	Quick Breads and Bread Products
52303500	Muffin, wheat	Lo	Quick Breads and Bread Products
52304000	Muffin, whole grain	Lo	Quick Breads and Bread Products
52304010	Muffin, wheat bran	Lo	Quick Breads and Bread Products
52304040	Muffin, bran with fruit, lowfat	Lo	Quick Breads and Bread Products
52304060	Muffin, bran with fruit, no fat, no cho	Lo	Quick Breads and Bread Products
52304100	Muffin, oatmeal	Lo	Quick Breads and Bread Products
52304150	Muffin, oat bran	Lo	Quick Breads and Bread Products
52304200	Muffin, oat bran with fruit and/or nut	Lo	Quick Breads and Bread Products
52306010	Muffin, plain	Lo	Quick Breads and Bread Products
52306300	Muffin, cheese	Lo	Quick Breads and Bread Products
52306500	Muffin, pumpkin	Lo	Quick Breads and Bread Products
52306550	Muffin, zucchini	Lo	Quick Breads and Bread Products
52306700	Muffin, carrot	Lo	Quick Breads and Bread Products
52307120	Muffin, multigrain, with fruit	Lo	Quick Breads and Bread Products
52308010	Matzo, fritters	Lo	Quick Breads and Bread Products
52308020	Matzo ball	Lo	Quick Breads and Bread Products

52311010	Popover	Lo	Quick Breads and Bread Products
52320110	Toaster muffin, fruit, toasted	Lo	Quick Breads and Bread Products
52401000	Bread, Boston Brown	Lo	Quick Breads and Bread Products
52403000	Bread, nut	Lo	Quick Breads and Bread Products
52404060	Bread, pumpkin	Lo	Quick Breads and Bread Products
52405010	Bread, fruit	Lo	Quick Breads and Bread Products
52405100	Bread, fruit and nut	Lo	Quick Breads and Bread Products
52406010	Bread, whole wheat, with nuts	Lo	Quick Breads and Bread Products
52407000	Bread, zucchini	Lo	Quick Breads and Bread Products
52408000	Bread, Irish soda	Lo	Quick Breads and Bread Products
53100050	Cake batter, raw, chocolate	Lo	Sweet Bakery Products
53100070	Cake batter, raw, not chocolate	Lo	Sweet Bakery Products
53100100	Cake or cupcake, NS as to type	Lo	Sweet Bakery Products
53101100	Cake, angel food, without icing or fi	Lo	Sweet Bakery Products
53101200	Cake, angel food, with icing or filling	Lo	Sweet Bakery Products
53101250	Cake, angel food, with fruit and icin	Lo	Sweet Bakery Products
53101300	Cake, angel food, chocolate, withou	Lo	Sweet Bakery Products
53102000	Cake, applesauce, NS as to icing	Lo	Sweet Bakery Products
53102100	Cake or cupcake, applesauce, with	Lo	Sweet Bakery Products
53102200	Cake or cupcake, applesauce, with	Lo	Sweet Bakery Products
53102300	Cake, applesauce, diet, without icin	Lo	Sweet Bakery Products
53102500	Cake, banana, NS as to icing	Lo	Sweet Bakery Products
53102600	Cake or cupcake, banana, without i	Lo	Sweet Bakery Products
53102700	Cake or cupcake, banana, with icin	Lo	Sweet Bakery Products
53102800	Cake or cupcake, Black Forest	Lo	Sweet Bakery Products
53103000	Cake, Boston cream pie	Lo	Sweet Bakery Products
53103550	Cake, butter, without icing	Lo	Sweet Bakery Products
53103600	Cake, butter, with icing	Lo	Sweet Bakery Products
53104000	Cake, carrot, NS as to icing	Lo	Sweet Bakery Products
53104100	Cake or cupcake, carrot, without ici	Lo	Sweet Bakery Products
53104260	Cake or cupcake, carrot, with icing	Lo	Sweet Bakery Products
53104300	Cake, carrot, diet	Lo	Sweet Bakery Products
53104400	Cake or cupcake, coconut, with icin	Lo	Sweet Bakery Products
53104500	Cheesecake	Lo	Sweet Bakery Products
53104520	Cheesecake, diet	Lo	Sweet Bakery Products
53104550	Cheesecake with fruit	Lo	Sweet Bakery Products
53104570	Cheesecake, diet, with fruit	Lo	Sweet Bakery Products
53104580	Cheesecake -type dessert, made w	Lo	Sweet Bakery Products
53104600	Cheesecake, chocolate	Lo	Sweet Bakery Products
53105050	Cake, chocolate, devil's food, or fud	Lo	Sweet Bakery Products
53105100	Cake, chocolate, devil's food, or fud	Lo	Sweet Bakery Products
53105160	Cake, chocolate, devil's food, or fud	Lo	Sweet Bakery Products
53105200	Cake, chocolate, devil's food, or fud	Lo	Sweet Bakery Products
53105260	Cake, chocolate, devil's food, or fud	Lo	Sweet Bakery Products
53105270	Cake or cupcake, chocolate, devil's	Lo	Sweet Bakery Products
53105275	Cake or cupcake, chocolate, devil's	Lo	Sweet Bakery Products
53105300	Cake or cupcake, German chocolat	Lo	Sweet Bakery Products
53105500	Cake, chocolate, with icing, diet	Lo	Sweet Bakery Products
53105600	Cake, chocolate, devil's food, or fud	Lo	Sweet Bakery Products
53105700	Cake, chocolate, devil's food, or fud	Lo	Sweet Bakery Products
53105750	Cake, chocolate, devil's food, or fud	Lo	Sweet Bakery Products

53106000	Cake, chocolate, devil's food, or fud	Lo	Sweet Bakery Products
53106050	Cake, chocolate, devil's food, or fud	Lo	Sweet Bakery Products
53106500	Cake, cream, without icing or toppir	Lo	Sweet Bakery Products
53107000	Cake, cupcake, NS as to type or ici	Lo	Sweet Bakery Products
53107100	Cake, cupcake, NS as to type, with	Lo	Sweet Bakery Products
53107200	Cake, cupcake, NS as to type, with	Lo	Sweet Bakery Products
53108000	Cake, cupcake, chocolate, NS as to	Lo	Sweet Bakery Products
53108100	Cake, cupcake, chocolate, without i	Lo	Sweet Bakery Products
53108200	Snack cake, chocolate, with icing or	Lo	Sweet Bakery Products
53108220	Snack cake, chocolate, with icing or	Lo	Sweet Bakery Products
53109000	Cake, cupcake, not chocolate, NS a	Lo	Sweet Bakery Products
53109100	Cake, cupcake, not chocolate, with	Lo	Sweet Bakery Products
53109200	Snack cake, not chocolate, with icir	Lo	Sweet Bakery Products
53109210	Cake, cupcake, not chocolate, with	Lo	Sweet Bakery Products
53109220	Snack cake, not chocolate, with icir	Lo	Sweet Bakery Products
53109250	Cake, cupcake, not chocolate, with	Lo	Sweet Bakery Products
53109270	Cake, cupcake, chocolate, with or w	Lo	Sweet Bakery Products
53109300	Cake, Dobos Torte	Lo	Sweet Bakery Products
53110000	Cake, fruit cake, light or dark, holida	Lo	Sweet Bakery Products
53111000	Cake or cupcake, gingerbread	Lo	Sweet Bakery Products
53111500	Cake, graham cracker, without icing	Lo	Sweet Bakery Products
53112000	Cake, ice cream and cake roll, choc	Lo	Sweet Bakery Products
53112100	Ice cream cake	Lo	Sweet Bakery Products
53113000	Cake, jelly roll	Lo	Sweet Bakery Products
53114000	Cake or cupcake, lemon, without ic	Lo	Sweet Bakery Products
53114100	Cake or cupcake, lemon, with icing	Lo	Sweet Bakery Products
53114200	Cake, lemon, lowfat, without icing	Lo	Sweet Bakery Products
53114250	Cake, lemon, lowfat, with icing	Lo	Sweet Bakery Products
53115100	Cake or cupcake, marble, without ic	Lo	Sweet Bakery Products
53115200	Cake or cupcake, marble, with icing	Lo	Sweet Bakery Products
53115310	Cake or cupcake, nut, without icing	Lo	Sweet Bakery Products
53115320	Cake or cupcake, nut, with icing or	Lo	Sweet Bakery Products
53115400	Cake, oatmeal, without icing	Lo	Sweet Bakery Products
53115410	Cake or cupcake, oatmeal	Lo	Sweet Bakery Products
53115450	Cake or cupcake, peanut butter	Lo	Sweet Bakery Products
53115600	Cake, poppyseed, without icing	Lo	Sweet Bakery Products
53116000	Cake, pound, without icing or filling	Lo	Sweet Bakery Products
53116020	Cake, pound, with icing or filling	Lo	Sweet Bakery Products
53116270	Cake, pound, chocolate	Lo	Sweet Bakery Products
53116350	Cake, pound, Puerto Rican style	Lo	Sweet Bakery Products
53116380	Cake, pound, fat free, cholesterol fr	Lo	Sweet Bakery Products
53116390	Cake, pound, reduced fat, choleste	Lo	Sweet Bakery Products
53116490	Cake, pumpkin, NS as to icing	Lo	Sweet Bakery Products
53116500	Cake or cupcake, pumpkin, without	Lo	Sweet Bakery Products
53116510	Cake or cupcake, pumpkin, with icir	Lo	Sweet Bakery Products
53116550	Cake or cupcake, raisin-nut	Lo	Sweet Bakery Products
53116560	Cake, raisin-nut, with icing	Lo	Sweet Bakery Products
53116570	Cake, Ravani	Lo	Sweet Bakery Products
53116600	Cake, rice flour, without icing or fillir	Lo	Sweet Bakery Products
53116650	Cake, Quezadilla, El Salvadorian st	Lo	Sweet Bakery Products
53117100	Cake or cupcake, spice, without icir	Lo	Sweet Bakery Products

53117200	Cake or cupcake, spice, with icing o	Lo	Sweet Bakery Products
53118100	Cake, sponge, without icing or filling	Lo	Sweet Bakery Products
53118200	Cake, sponge, with icing or filling	Lo	Sweet Bakery Products
53118300	Cake, sponge, chocolate	Lo	Sweet Bakery Products
53118310	Cake, sponge, chocolate, with icing	Lo	Sweet Bakery Products
53118350	Cake, sweetpotato, with icing	Lo	Sweet Bakery Products
53118410	Rum cake, without icing	Lo	Sweet Bakery Products
53118500	Cake, torte	Lo	Sweet Bakery Products
53118550	Cake, tres leche	Lo	Sweet Bakery Products
53119000	Cake, pineapple, upside down	Lo	Sweet Bakery Products
53120000	Cake, white, standard-type mix (egg	Lo	Sweet Bakery Products
53120060	Cake, white, made from home recip	Lo	Sweet Bakery Products
53120100	Cake, white, standard-type mix (egg	Lo	Sweet Bakery Products
53120160	Cake, white, without icing, made fro	Lo	Sweet Bakery Products
53120200	Cake, white, standard-type mix (egg	Lo	Sweet Bakery Products
53120260	Cake, white, with icing, made from	Lo	Sweet Bakery Products
53120270	Cake or cupcake, white, with icing o	Lo	Sweet Bakery Products
53120275	Cake or cupcake, white, without icin	Lo	Sweet Bakery Products
53120330	Cake, white, pudding-type mix (oil,	Lo	Sweet Bakery Products
53120350	Cake, white, pudding-type mix (oil,	Lo	Sweet Bakery Products
53120400	Cake, white, eggless, lowfat	Lo	Sweet Bakery Products
53120500	Cake, whole wheat, with fruit and nu	Lo	Sweet Bakery Products
53121060	Cake, yellow, made from home reci	Lo	Sweet Bakery Products
53121100	Cake, yellow, standard-type mix (eg	Lo	Sweet Bakery Products
53121160	Cake, yellow, without icing, made fr	Lo	Sweet Bakery Products
53121200	Cake, yellow, standard-type mix (eg	Lo	Sweet Bakery Products
53121260	Cake, yellow, with icing, made from	Lo	Sweet Bakery Products
53121270	Cake or cupcake, yellow, with icing	Lo	Sweet Bakery Products
53121275	Cake or cupcake, yellow, without ici	Lo	Sweet Bakery Products
53121280	Cake, yellow, pudding-type mix (oil,	Lo	Sweet Bakery Products
53121300	Cake, yellow, pudding-type mix (oil,	Lo	Sweet Bakery Products
53121330	Cake, yellow, pudding-type mix (oil,	Lo	Sweet Bakery Products
53122070	Cake, shortcake, biscuit type, with w	Lo	Sweet Bakery Products
53122080	Cake, shortcake, biscuit type, with f	Lo	Sweet Bakery Products
53123070	Cake, shortcake, sponge type, with	Lo	Sweet Bakery Products
53123080	Cake, shortcake, sponge type, with	Lo	Sweet Bakery Products
53123500	Cake, shortcake, with whipped topp	Lo	Sweet Bakery Products
53124110	Cake or cupcake, zucchini	Lo	Sweet Bakery Products
53124120	Cake, zucchini, with icing	Lo	Sweet Bakery Products
53200100	Cookie, batter or dough, raw	Lo	Sweet Bakery Products
53201000	Cookie, NFS	Lo	Sweet Bakery Products
53202000	Cookie, almond	Lo	Sweet Bakery Products
53203000	Cookie, applesauce	Lo	Sweet Bakery Products
53203050	Cookie, fruit, baby	Lo	Baby Foods
53203100	Cookie, baby	Lo	Baby Foods
53203500	Cookie, biscotti	Lo	Sweet Bakery Products
53204000	Cookie, brownie, NS as to icing	Lo	Sweet Bakery Products
53204010	Cookie, brownie, without icing	Lo	Sweet Bakery Products
53204100	Cookie, brownie, with icing or filling	Lo	Sweet Bakery Products
53204500	Cookie, brownie, with cream chees	Lo	Sweet Bakery Products
53204600	Cookie, brownie, with peanut butter	Lo	Sweet Bakery Products

53204800	Cookie, brownie, diet, NS as to icin	Lo	Sweet Bakery Products
53204830	Cookie, brownie, lowfat, with icing	Lo	Sweet Bakery Products
53204840	Cookie, brownie, reduced fat, NS a	Lo	Sweet Bakery Products
53204850	Cookie, brownie, fat free, choleste	Lo	Sweet Bakery Products
53204860	Cookie, brownie, fat free, NS as to	Lo	Sweet Bakery Products
53205250	Cookie, butterscotch, brownie	Lo	Sweet Bakery Products
53205260	Cookie, bar, with chocolate	Lo	Sweet Bakery Products
53205500	Cookie, butterscotch chip	Lo	Sweet Bakery Products
53205600	Cookie, caramel coated, with nuts	Lo	Sweet Bakery Products
53206000	Cookie, chocolate chip	Lo	Sweet Bakery Products
53206010	Cookie, chocolate chip, with raisins	Lo	Sweet Bakery Products
53206020	Cookie, chocolate chip, made from	Lo	Sweet Bakery Products
53206030	Cookie, chocolate chip, reduced fat	Lo	Sweet Bakery Products
53206050	Cookie, rich, chocolate chip, with ch	Lo	Sweet Bakery Products
53206100	Cookie, chocolate chip sandwich	Lo	Sweet Bakery Products
53206500	Cookie, chocolate, made with rice c	Lo	Sweet Bakery Products
53206550	Cookie, chocolate, made with oatm	Lo	Sweet Bakery Products
53207000	Cookie, chocolate or fudge	Lo	Sweet Bakery Products
53207020	Cookie, chocolate or fudge, reduce	Lo	Sweet Bakery Products
53207050	Cookie, chocolate, with chocolate fi	Lo	Sweet Bakery Products
53208000	Cookie, marshmallow, chocolate-co	Lo	Sweet Bakery Products
53208200	Cookie, marshmallow pie, chocolate	Lo	Sweet Bakery Products
53209000	Cookie, chocolate, chocolate sandw	Lo	Sweet Bakery Products
53209005	Cookie, chocolate, with icing or coa	Lo	Sweet Bakery Products
53209010	Cookie, sugar wafer, chocolate-cov	Lo	Sweet Bakery Products
53209015	Cookie, chocolate sandwich	Lo	Sweet Bakery Products
53209020	Cookie, chocolate sandwich, reduce	Lo	Sweet Bakery Products
53209050	Cookie, chocolate-covered, chocola	Lo	Sweet Bakery Products
53209100	Cookie, chocolate, sandwich, with e	Lo	Sweet Bakery Products
53209500	Cookie, chocolate and vanilla sandw	Lo	Sweet Bakery Products
53210000	Cookie, chocolate wafer	Lo	Sweet Bakery Products
53210900	Cookie, graham cracker with choco	Lo	Sweet Bakery Products
53210910	Cookie, graham cracker with marsh	Lo	Sweet Bakery Products
53211000	Cookie bar, with chocolate, nuts, an	Lo	Sweet Bakery Products
53215500	Cookie, coconut	Lo	Sweet Bakery Products
53216000	Cookie, coconut and nut	Lo	Sweet Bakery Products
53220000	Cookie, fruit-filled bar	Lo	Sweet Bakery Products
53220010	Cookie, fruit-filled bar, fat free	Lo	Sweet Bakery Products
53220020	Cookie, date bar	Lo	Sweet Bakery Products
53220030	Cookie, fig bar	Lo	Sweet Bakery Products
53220040	Cookie, fig bar, fat free	Lo	Sweet Bakery Products
53222010	Cookie, fortune	Lo	Sweet Bakery Products
53222020	Cookie, cone shell, ice cream type,	Lo	Sweet Bakery Products
53222100	Cookie, cone shell, ice cream type,	Lo	Sweet Bakery Products
53223000	Cookie, gingersnaps	Lo	Sweet Bakery Products
53223100	Cookie, granola	Lo	Sweet Bakery Products
53224000	Cookie, ladyfinger	Lo	Sweet Bakery Products
53224250	Cookie, lemon bar	Lo	Sweet Bakery Products
53225000	Cookie, macaroon	Lo	Sweet Bakery Products
53226000	Cookie, marshmallow, with coconut	Lo	Sweet Bakery Products
53226500	Cookie, marshmallow, with rice cere	Lo	Sweet Bakery Products

53226550	Cookie, marshmallow, with rice cer	Lo	Sweet Bakery Products
53226600	Cookie, marshmallow and peanut b	Lo	Sweet Bakery Products
53227000	Cookie, marshmallow pies, non-cho	Lo	Sweet Bakery Products
53228000	Cookie, meringue	Lo	Sweet Bakery Products
53230000	Cookie, molasses	Lo	Sweet Bakery Products
53231000	Cookie, Lebkuchen	Lo	Sweet Bakery Products
53231400	Cookie, multigrain, high fiber	Lo	Sweet Bakery Products
53233000	Cookie, oatmeal	Lo	Sweet Bakery Products
53233010	Cookie, oatmeal, with raisins	Lo	Sweet Bakery Products
53233020	Cookie, oatmeal, with fruit filling	Lo	Sweet Bakery Products
53233030	Cookie, oatmeal, fat free, with raisi	Lo	Sweet Bakery Products
53233040	Cookie, oatmeal, reduced fat, NS a	Lo	Sweet Bakery Products
53233050	Cookie, oatmeal sandwich, with cre	Lo	Sweet Bakery Products
53233060	Cookie, oatmeal, with chocolate chi	Lo	Sweet Bakery Products
53233080	Cookie, oatmeal sandwich, with pea	Lo	Sweet Bakery Products
53233100	Cookie, oatmeal, with chocolate an	Lo	Sweet Bakery Products
53233500	Cookie, oat bran	Lo	Sweet Bakery Products
53234000	Cookie, peanut butter	Lo	Sweet Bakery Products
53234010	Cookie, peanut butter, with oatmea	Lo	Sweet Bakery Products
53234100	Cookie, peanut butter, with chocola	Lo	Sweet Bakery Products
53234250	Cookie, peanut butter with rice cere	Lo	Sweet Bakery Products
53235000	Cookie, peanut butter sandwich	Lo	Sweet Bakery Products
53235500	Cookie, with peanut butter filling, ch	Lo	Sweet Bakery Products
53235600	Cookie, Pfeffernusse	Lo	Sweet Bakery Products
53236000	Cookie, Pizzelle	Lo	Sweet Bakery Products
53236100	Cookie, pumpkin	Lo	Sweet Bakery Products
53237000	Cookie, raisin	Lo	Sweet Bakery Products
53237010	Cookie, raisin sandwich, cream-fille	Lo	Sweet Bakery Products
53237500	Cookie, rum ball, no bake	Lo	Sweet Bakery Products
53238000	Cookie, sandwich-type, not chocola	Lo	Sweet Bakery Products
53239000	Cookie, shortbread	Lo	Sweet Bakery Products
53239010	Cookie, shortbread, reduced fat	Lo	Sweet Bakery Products
53239050	Cookie, shortbread, with icing or filli	Lo	Sweet Bakery Products
53239100	Pocky	Lo	Sweet Bakery Products
53240000	Cookie, animal	Lo	Sweet Bakery Products
53240010	Cookie, animal, with frosting or icin	Lo	Sweet Bakery Products
53241500	Cookie, butter or sugar	Lo	Sweet Bakery Products
53241510	Marie biscuit	Lo	Sweet Bakery Products
53241600	Cookie, butter or sugar, with fruit ar	Lo	Sweet Bakery Products
53242000	Cookie, sugar wafer	Lo	Sweet Bakery Products
53242250	Cookie, teething, baby food	Lo	Baby Foods
53242500	Cookie, toffee bar	Lo	Sweet Bakery Products
53243000	Cookie, vanilla sandwich	Lo	Sweet Bakery Products
53243010	Cookie, vanilla sandwich, extra fillin	Lo	Sweet Bakery Products
53243050	Cookie, vanilla sandwich, reduced f	Lo	Sweet Bakery Products
53243100	Cookie, rich, all chocolate, with cho	Lo	Sweet Bakery Products
53244010	Cookie, butter or sugar, with chocol	Lo	Sweet Bakery Products
53244020	Cookie, butter or sugar, with icing o	Lo	Sweet Bakery Products
53245000	Cookie, vanilla waffle creme	Lo	Sweet Bakery Products
53246000	Cookie, tea, Japanese	Lo	Sweet Bakery Products
53247000	Cookie, vanilla wafer	Lo	Sweet Bakery Products

53247050	Cookie, vanilla wafer, reduced fat	Lo	Sweet Bakery Products
53247500	Cookie, vanilla with caramel, cocon	Lo	Sweet Bakery Products
53248000	Cookie, whole wheat, dried fruit, nu	Lo	Sweet Bakery Products
53251100	Cookie, rugelach	Lo	Sweet Bakery Products
53260030	Cookie, chocolate chip, sugar free	Lo	Sweet Bakery Products
53260150	Cookie, lemon wafer, lowfat	Lo	Sweet Bakery Products
53260200	Cookie, oatmeal, sugar free	Lo	Sweet Bakery Products
53260300	Cookie, sandwich, sugar free	Lo	Sweet Bakery Products
53260400	Cookie, sugar or plain, sugar free	Lo	Sweet Bakery Products
53260500	Cookie, sugar wafer, sugar free	Lo	Sweet Bakery Products
53260600	Cookie, peanut butter, sugar free	Lo	Sweet Bakery Products
53261000	Cookie, gluten free	Lo	Sweet Bakery Products
53270100	Cookies, Puerto Rican style	Lo	Sweet Bakery Products
53300100	Pie, NFS	Lo	Sweet Bakery Products
53300170	Pie, individual size or tart, NFS	Lo	Sweet Bakery Products
53301000	Pie, apple, two crust	Lo	Sweet Bakery Products
53301070	Pie, apple, individual size or tart	Lo	Sweet Bakery Products
53301080	Pie, apple, fried pie	Lo	Sweet Bakery Products
53301500	Pie, apple, one crust	Lo	Sweet Bakery Products
53301750	Pie, apple, diet	Lo	Sweet Bakery Products
53302000	Pie, apricot, two crust	Lo	Sweet Bakery Products
53302070	Pie, apricot, individual size or tart	Lo	Sweet Bakery Products
53303000	Pie, blackberry, two crust	Lo	Sweet Bakery Products
53303070	Pie, blackberry, individual size or ta	Lo	Sweet Bakery Products
53303500	Pie, berry, not blackberry, blueberry	Lo	Sweet Bakery Products
53303510	Pie, berry, not blackberry, blueberry	Lo	Sweet Bakery Products
53303570	Pie, berry, not blackberry, blueberry	Lo	Sweet Bakery Products
53304000	Pie, blueberry, two crust	Lo	Sweet Bakery Products
53304050	Pie, blueberry, one crust	Lo	Sweet Bakery Products
53304070	Pie, blueberry, individual size or tart	Lo	Sweet Bakery Products
53305000	Pie, cherry, two crust	Lo	Sweet Bakery Products
53305010	Pie, cherry, one crust	Lo	Sweet Bakery Products
53305070	Pie, cherry, individual size or tart	Lo	Sweet Bakery Products
53305080	Pie, cherry, fried pie	Lo	Sweet Bakery Products
53305700	Pie, lemon, not cream or meringue	Lo	Sweet Bakery Products
53305720	Pie, lemon, not cream or meringue,	Lo	Sweet Bakery Products
53306000	Pie, mince, two crust	Lo	Sweet Bakery Products
53306070	Pie, mince, individual size or tart	Lo	Sweet Bakery Products
53307000	Pie, peach, two crust	Lo	Sweet Bakery Products
53307050	Pie, peach, one crust	Lo	Sweet Bakery Products
53307070	Pie, peach, individual size or tart	Lo	Sweet Bakery Products
53307080	Pie, peach, fried pie	Lo	Sweet Bakery Products
53307500	Pie, pear, two crust	Lo	Sweet Bakery Products
53308000	Pie, pineapple, two crust	Lo	Sweet Bakery Products
53308070	Pie, pineapple, individual size or tar	Lo	Sweet Bakery Products
53308500	Pie, prune, one crust	Lo	Sweet Bakery Products
53309000	Pie, raisin, two crust	Lo	Sweet Bakery Products
53309070	Pie, raisin, individual size or tart	Lo	Sweet Bakery Products
53310000	Pie, raspberry, one crust	Lo	Sweet Bakery Products
53310050	Pie, raspberry, two crust	Lo	Sweet Bakery Products
53311000	Pie, rhubarb, two crust	Lo	Sweet Bakery Products

53311050	Pie, rhubarb, one crust	Lo	Sweet Bakery Products
53311070	Pie, rhubarb, individual size or tart	Lo	Sweet Bakery Products
53312000	Pie, strawberry, one crust	Lo	Sweet Bakery Products
53313000	Pie, strawberry-rhubarb, two crust	Lo	Sweet Bakery Products
53314000	Pie, strawberry, individual size or ta	Lo	Sweet Bakery Products
53340500	Pie, cherry, made with cream chees	Lo	Sweet Bakery Products
53341000	Pie, banana cream	Lo	Sweet Bakery Products
53341070	Pie, banana cream, individual size c	Lo	Sweet Bakery Products
53341500	Pie, buttermilk	Lo	Sweet Bakery Products
53341750	Pie, chess	Lo	Sweet Bakery Products
53342000	Pie, chocolate cream	Lo	Sweet Bakery Products
53342070	Pie, chocolate cream, individual size	Lo	Sweet Bakery Products
53343000	Pie, coconut cream	Lo	Sweet Bakery Products
53343070	Pie, coconut cream, individual size	Lo	Sweet Bakery Products
53344000	Pie, custard	Lo	Sweet Bakery Products
53344070	Pie, custard, individual size or tart	Lo	Sweet Bakery Products
53344200	Mixed fruit tart filled with custard or	Lo	Sweet Bakery Products
53344300	Dessert pizza	Lo	Sweet Bakery Products
53345000	Pie, lemon cream	Lo	Sweet Bakery Products
53345070	Pie, lemon cream, individual size or	Lo	Sweet Bakery Products
53346000	Pie, peanut butter cream	Lo	Sweet Bakery Products
53346500	Pie, pineapple cream	Lo	Sweet Bakery Products
53347000	Pie, pumpkin	Lo	Sweet Bakery Products
53347070	Pie, pumpkin, individual size or tart	Lo	Sweet Bakery Products
53347100	Pie, raspberry cream	Lo	Sweet Bakery Products
53347600	Pie, squash	Lo	Sweet Bakery Products
53348000	Pie, strawberry cream	Lo	Sweet Bakery Products
53348070	Pie, strawberry cream, individual siz	Lo	Sweet Bakery Products
53360000	Pie, sweet potato	Lo	Sweet Bakery Products
53365000	Pie, vanilla cream	Lo	Sweet Bakery Products
53366000	Pie, yogurt, frozen	Lo	Sweet Bakery Products
53370000	Pie, chiffon, not chocolate	Lo	Sweet Bakery Products
53381000	Pie, lemon meringue	Lo	Sweet Bakery Products
53381070	Pie, lemon meringue, individual size	Lo	Sweet Bakery Products
53382000	Pie, chocolate-marshmallow	Lo	Sweet Bakery Products
53385000	Pie, pecan	Lo	Sweet Bakery Products
53385070	Pie, pecan, individual size or tart	Lo	Sweet Bakery Products
53385500	Pie, oatmeal	Lo	Sweet Bakery Products
53386000	Pie, pudding, flavors other than cho	Lo	Sweet Bakery Products
53386050	Pie, pudding, flavors other than cho	Lo	Sweet Bakery Products
53386250	Pie, pudding, chocolate, with choco	Lo	Sweet Bakery Products
53386500	Pie, pudding, flavors other than cho	Lo	Sweet Bakery Products
53387000	Pie, Toll house chocolate chip	Lo	Sweet Bakery Products
53390000	Pie, shoo-fly	Lo	Sweet Bakery Products
53391000	Pie shell	Lo	Sweet Bakery Products
53391100	Pie shell, graham cracker	Lo	Sweet Bakery Products
53391150	Pie shell, chocolate wafer	Lo	Sweet Bakery Products
53400200	Blintz, cheese-filled	Lo	Quick Breads and Bread Products
53400300	Blintz, fruit-filled	Lo	Quick Breads and Bread Products
53410100	Cobbler, apple	Lo	Sweet Bakery Products
53410300	Cobbler, berry	Lo	Sweet Bakery Products

53410500	Cobbler, cherry	Lo	Sweet Bakery Products
53410800	Cobbler, peach	Lo	Sweet Bakery Products
53410850	Cobbler, pear	Lo	Sweet Bakery Products
53410860	Cobbler, pineapple	Lo	Sweet Bakery Products
53410900	Cobbler, rhubarb	Lo	Sweet Bakery Products
53415100	Crisp, apple, apple dessert	Lo	Sweet Bakery Products
53415120	Fritter, apple	Lo	Sweet Bakery Products
53415200	Fritter, banana	Lo	Sweet Bakery Products
53415220	Fritter, berry	Lo	Sweet Bakery Products
53415300	Crisp, blueberry	Lo	Sweet Bakery Products
53415400	Crisp, cherry	Lo	Sweet Bakery Products
53415500	Crisp, peach	Lo	Sweet Bakery Products
53415600	Crisp, rhubarb	Lo	Sweet Bakery Products
53420000	Cream puff, eclair, custard or crean	Lo	Sweet Bakery Products
53420100	Cream puff, eclair, custard or crean	Lo	Sweet Bakery Products
53420200	Cream puff, eclair, custard or crean	Lo	Sweet Bakery Products
53420210	Cream puff, eclair, custard or crean	Lo	Sweet Bakery Products
53420250	Cream puff, no filling or icing	Lo	Sweet Bakery Products
53420300	Air filled fritter or fried puff, without s	Lo	Sweet Bakery Products
53420310	Wheat flour fritter, without syrup	Lo	Sweet Bakery Products
53420400	Sopaipilla, without syrup or honey	Lo	Sweet Bakery Products
53420410	Sopaipilla with syrup or honey	Lo	Sweet Bakery Products
53430000	Crepe, NS as to filling	Lo	Quick Breads and Bread Products
53430100	Crepe, chocolate filled	Lo	Quick Breads and Bread Products
53430200	Crepe, fruit filled	Lo	Quick Breads and Bread Products
53430250	Crepe suzette	Lo	Quick Breads and Bread Products
53430700	Tamale, sweet	Lo	Sweet Bakery Products
53430750	Tamale, sweet, with fruit	Lo	Sweet Bakery Products
53440000	Strudel, apple	Lo	Sweet Bakery Products
53440300	Strudel, berry	Lo	Sweet Bakery Products
53440500	Strudel, cherry	Lo	Sweet Bakery Products
53440600	Strudel, cheese	Lo	Sweet Bakery Products
53440800	Strudel, cheese and fruit	Lo	Sweet Bakery Products
53441110	Baklava	Lo	Sweet Bakery Products
53441210	Basbousa	Lo	Sweet Bakery Products
53450000	Turnover or dumpling, apple	Lo	Sweet Bakery Products
53450300	Turnover or dumpling, berry	Lo	Sweet Bakery Products
53450500	Turnover or dumpling, cherry	Lo	Sweet Bakery Products
53450800	Turnover or dumpling, lemon	Lo	Sweet Bakery Products
53451000	Turnover or dumpling, peach	Lo	Sweet Bakery Products
53451500	Turnover, guava	Lo	Sweet Bakery Products
53451750	Turnover, pumpkin	Lo	Sweet Bakery Products
53452100	Pastry, fruit-filled	Lo	Sweet Bakery Products
53452120	Pastry, made with bean or lotus see	Lo	Sweet Bakery Products
53452130	Pastry, made with bean paste and s	Lo	Sweet Bakery Products
53452150	Pastry, Chinese, made with rice flou	Lo	Sweet Bakery Products
53452170	Pastry, cookie type, fried	Lo	Sweet Bakery Products
53452200	Pastry, Italian, with cheese	Lo	Sweet Bakery Products
53452400	Pastry, puff	Lo	Sweet Bakery Products
53452420	Pastry, puff, custard or cream filled,	Lo	Sweet Bakery Products
53452450	Cheese pastry puffs	Lo	Sweet Bakery Products

53452500	Pastry, mainly flour and water, fried	Lo	Sweet Bakery Products
53453150	Empanada, Mexican turnover, fruit-	Lo	Sweet Bakery Products
53453170	Empanada, Mexican turnover, pum	Lo	Sweet Bakery Products
53500100	Breakfast pastry, NFS	Lo	Sweet Bakery Products
53510000	Danish pastry, plain or spice	Lo	Sweet Bakery Products
53510100	Danish pastry, with fruit	Lo	Sweet Bakery Products
53510200	Danish pastry, with nuts	Lo	Sweet Bakery Products
53511000	Danish pastry, with cheese	Lo	Sweet Bakery Products
53511500	Danish pastry, with cheese, fat free	Lo	Sweet Bakery Products
53520000	Doughnut, NFS	Lo	Sweet Bakery Products
53520100	Doughnut, cake type, plain	Lo	Sweet Bakery Products
53520110	Doughnut, cake type	Lo	Sweet Bakery Products
53520120	Doughnut, chocolate	Lo	Sweet Bakery Products
53520130	Doughnut, cake type, powdered su	Lo	Sweet Bakery Products
53520135	Doughnut, cake type, with icing	Lo	Sweet Bakery Products
53520140	Doughnut, cake type, chocolate icin	Lo	Sweet Bakery Products
53520150	Doughnut, cake type, chocolate cov	Lo	Sweet Bakery Products
53520160	Doughnut, chocolate, with chocolat	Lo	Sweet Bakery Products
53520170	Doughnut holes	Lo	Sweet Bakery Products
53520200	Churros	Lo	Sweet Bakery Products
53520500	Doughnut, Asian	Lo	Sweet Bakery Products
53520510	Beignet	Lo	Sweet Bakery Products
53520600	Cruller, NFS	Lo	Sweet Bakery Products
53520700	French cruller	Lo	Sweet Bakery Products
53521100	Doughnut, chocolate, raised or yea	Lo	Sweet Bakery Products
53521110	Doughnut, yeast type	Lo	Sweet Bakery Products
53521120	Doughnut, chocolate, raised or yea	Lo	Sweet Bakery Products
53521130	Doughnut, yeast type, with chocolat	Lo	Sweet Bakery Products
53521140	Doughnut, jelly	Lo	Sweet Bakery Products
53521210	Doughnut, custard-filled	Lo	Sweet Bakery Products
53521220	Doughnut, chocolate cream-filled	Lo	Sweet Bakery Products
53521230	Doughnut, custard-filled, with icing	Lo	Sweet Bakery Products
53521250	Doughnut, wheat	Lo	Sweet Bakery Products
53521300	Doughnut, wheat, chocolate covere	Lo	Sweet Bakery Products
53530000	Breakfast tart	Lo	Sweet Bakery Products
53530010	Breakfast tart, lowfat	Lo	Sweet Bakery Products
53540000	Breakfast bar, NFS	Lo	Snack/Meal Bars
53540200	Breakfast bar, cereal crust with fruit	Lo	Snack/Meal Bars
53540250	Breakfast bar, cereal crust with fruit	Lo	Snack/Meal Bars
53540300	Fiber One Chewy Bar	Lo	Snack/Meal Bars
53540400	Kellogg's Nutri-Grain Cereal Bar	Lo	Snack/Meal Bars
53540402	Kellogg's Nutri-Grain Yogurt Bar	Lo	Snack/Meal Bars
53540404	Kellogg's Nutri-Grain Fruit and Nut	Lo	Snack/Meal Bars
53540500	Breakfast bar, date, with yogurt coa	Lo	Snack/Meal Bars
53540600	Milk 'n Cereal bar	Lo	Snack/Meal Bars
53540700	Kellogg's Special K bar	Lo	Snack/Meal Bars
53540800	Kashi GOLEAN Chewy Bars	Lo	Snack/Meal Bars
53540802	Kashi TLC Chewy Granola Bar	Lo	Snack/Meal Bars
53540804	Kashi GOLEAN Crunchy Bars	Lo	Snack/Meal Bars
53540806	Kashi TLC Crunchy Granola Bar	Lo	Snack/Meal Bars
53540900	Nature Valley Chewy Trail Mix Gran	Lo	Snack/Meal Bars

53540902	Nature Valley Chewy Granola Bar w	Lo	Snack/Meal Bars
53540904	Nature Valley Sweet and Salty Nut (	Lo	Snack/Meal Bars
53540906	Nature Valley Crunchy Granola Bar	Lo	Snack/Meal Bars
53541000	Quaker Chewy Granola Bar	Lo	Snack/Meal Bars
53541002	Quaker Chewy 90 Calorie Granola	Lo	Snack/Meal Bars
53541004	Quaker Chewy 25% Less Sugar Gr	Lo	Snack/Meal Bars
53541006	Quaker Chewy Dipps Granola Bar	Lo	Snack/Meal Bars
53541200	Meal replacement bar	Lo	Snack/Meal Bars
53541300	Slim Fast Original Meal Bar	Lo	Snack/Meal Bars
53542000	Snack bar, oatmeal	Lo	Snack/Meal Bars
53542100	Granola bar, NFS	Lo	Snack/Meal Bars
53542200	Granola bar, lowfat, NFS	Lo	Snack/Meal Bars
53542210	Granola bar, nonfat	Lo	Snack/Meal Bars
53543000	Granola bar, reduced sugar, NFS	Lo	Snack/Meal Bars
53543100	Granola bar, peanuts, oats, sugar, v	Lo	Snack/Meal Bars
53544200	Granola bar, chocolate-coated, NFS	Lo	Snack/Meal Bars
53544210	Granola bar, with coconut, chocolat	Lo	Snack/Meal Bars
53544220	Granola bar with nuts, chocolate-co	Lo	Snack/Meal Bars
53544230	Granola bar, oats, nuts, coated with	Lo	Snack/Meal Bars
53544250	Granola bar, coated with non-choco	Lo	Snack/Meal Bars
53544300	Granola bar, high fiber, coated with	Lo	Snack/Meal Bars
53544400	Granola bar, with rice cereal	Lo	Snack/Meal Bars
53544410	Quaker Granola Bites	Lo	Snack/Meal Bars
53544450	PowerBar (fortified high energy bar)	Lo	Snack/Meal Bars
53610000	Coffee cake, NFS	Lo	Sweet Bakery Products
53610100	Coffee cake, crumb or quick-bread	Lo	Sweet Bakery Products
53610120	Coffee cake, crumb or quick-bread	Lo	Sweet Bakery Products
53610170	Coffee cake, crumb or quick-bread	Lo	Sweet Bakery Products
53610200	Coffee cake, crumb or quick-bread	Lo	Sweet Bakery Products
53610250	Coffee cake, crumb or quick-bread	Lo	Sweet Bakery Products
53710400	Cereal or granola bar (General Mills	Lo	Snack/Meal Bars
53710500	Cereal or granola bar (Kellogg's Nu	Lo	Snack/Meal Bars
53710502	Cereal or granola bar (Kellogg's Nu	Lo	Snack/Meal Bars
53710504	Cereal or granola bar (Kellogg's Nu	Lo	Snack/Meal Bars
53710600	Milk 'n Cereal bar	Lo	Snack/Meal Bars
53710700	Cereal or granola bar (Kellogg's Sp	Lo	Snack/Meal Bars
53710800	Cereal or granola bar (Kashi Chewy	Lo	Snack/Meal Bars
53710802	Cereal or granola bar (Kashi Crunch	Lo	Snack/Meal Bars
53710804	Kashi GOLEAN Crunchy Bars	Lo	Snack/Meal Bars
53710806	Kashi TLC Crunchy Granola Bar	Lo	Snack/Meal Bars
53710810	Cereal or granola bar (KIND Fruit a	Lo	Snack/Meal Bars
53710900	Cereal or granola bar (General Mills	Lo	Snack/Meal Bars
53710902	Cereal or granola bar, with yogurt c	Lo	Snack/Meal Bars
53710904	Cereal or granola bar (General Mills	Lo	Snack/Meal Bars
53710906	Cereal or granola bar (General Mills	Lo	Snack/Meal Bars
53711000	Cereal or granola bar (Quaker Chew	Lo	Snack/Meal Bars
53711002	Cereal or granola bar (Quaker Chew	Lo	Snack/Meal Bars
53711004	Cereal or granola bar (Quaker Chew	Lo	Snack/Meal Bars
53711006	Cereal or granola bar (Quaker Chew	Lo	Snack/Meal Bars
53711100	Cereal or granola bar (Quaker Gran	Lo	Snack/Meal Bars
53712000	Snack bar, oatmeal	Lo	Snack/Meal Bars

53712100	Cereal or Granola bar, NFS	Lo	Snack/Meal Bars
53712200	Cereal or granola bar, lowfat, NFS	Lo	Snack/Meal Bars
53712210	Cereal or granola bar, nonfat	Lo	Snack/Meal Bars
53713010	Cereal or granola bar, fruit and nut	Lo	Snack/Meal Bars
53713100	Cereal or granola bar, peanuts , oat	Lo	Snack/Meal Bars
53714200	Cereal or granola bar, chocolate co	Lo	Snack/Meal Bars
53714210	Cereal or granola bar, with coconut	Lo	Snack/Meal Bars
53714220	Cereal or granola bar with nuts, cho	Lo	Snack/Meal Bars
53714230	Cereal or granola bar, oats, nuts, co	Lo	Snack/Meal Bars
53714250	Cereal or granola bar, coated with n	Lo	Snack/Meal Bars
53714300	Cereal or granola bar, high fiber, co	Lo	Snack/Meal Bars
53714400	Cereal or granola bar, with rice cere	Lo	Snack/Meal Bars
53714500	Breakfast bar, NFS	Lo	Snack/Meal Bars
53714520	Breakfast bar, cereal crust with fruit	Lo	Snack/Meal Bars
53720100	Nutrition bar (Balance Original Bar)	Lo	Snack/Meal Bars
53720200	Nutrition bar (Clif Bar)	Lo	Snack/Meal Bars
53720210	Nutrition bar (Clif Kids Organic Zbar	Lo	Snack/Meal Bars
53720300	Nutrition bar (PowerBar)	Lo	Snack/Meal Bars
53720400	Nutrition bar (Slim Fast Original Me	Lo	Snack/Meal Bars
53720500	Nutrition bar (Snickers Marathon Pr	Lo	Snack/Meal Bars
53720600	Nutrition bar (South Beach Living M	Lo	Snack/Meal Bars
53720610	Nutrition bar (South Beach Living H	Lo	Snack/Meal Bars
53720700	Nutrition bar (Tiger's Milk)	Lo	Snack/Meal Bars
53720800	Nutrition bar (Zone Perfect Classic	Lo	Snack/Meal Bars
53729000	Nutrition bar or meal replacement b	Lo	Snack/Meal Bars
53801000	Cereal bar with fruit filling, baby foo	Lo	Baby Foods
53803050	Cookie, fruit, baby food	Lo	Baby Foods
53803100	Cookie, baby food	Lo	Baby Foods
53803250	Cookie, teething, baby	Lo	Baby Foods
53803300	Cookie, rice, baby	Lo	Baby Foods
54001000	Crackers, NFS	Lo	Crackers
54101010	Cracker, animal	Lo	Sweet Bakery Products
54102010	Graham crackers	Lo	Sweet Bakery Products
54102015	Graham crackers (Teddy Grahams)	Lo	Sweet Bakery Products
54102020	Graham crackers, chocolate covere	Lo	Sweet Bakery Products
54102050	Crackers, oatmeal	Lo	Crackers
54102060	Crackers, Cuban	Lo	Sweet Bakery Products
54102100	Graham crackers, reduced fat	Lo	Sweet Bakery Products
54102110	Crackers, graham, fat free	Lo	Sweet Bakery Products
54102200	Graham crackers, sandwich, with fi	Lo	Sweet Bakery Products
54103000	Crackers, breakfast biscuit	Lo	Crackers
54200100	Crackers, butter, reduced sodium	Lo	Crackers
54201010	Crackers, matzo, reduced sodium	Lo	Crackers
54202010	Crackers, saltine, low sodium	Lo	Crackers
54202020	Crackers, saltine, reduced sodium	Lo	Crackers
54202050	Crackers, saltine, fat free, low sodiu	Lo	Crackers
54203010	Crackers, toast thins (rye, wheat, w	Lo	Crackers
54204010	Cracker, 100% whole wheat, low so	Lo	Crackers
54204020	Crackers, wheat, reduced sodium	Lo	Crackers
54204030	Crackers, woven wheat, reduced so	Lo	Crackers
54205010	Cracker, snack, low sodium	Lo	Crackers

54205030	Cracker, cheese, low sodium	Lo	Crackers
54205100	Cracker, snack, reduced fat, reduce	Lo	Crackers
54206010	Puffed rice cake without salt	Lo	Crackers
54207010	Crispbread, wheat, low sodium	Lo	Crackers
54210010	Cracker, multigrain, low sodium	Lo	Crackers
54222000	Crispbread, rye, low sodium	Lo	Crackers
54301000	Cracker, snack	Lo	Crackers
54301010	Crackers, butter, plain	Lo	Crackers
54301020	Crackers, butter, flavored	Lo	Crackers
54301030	Crackers, butter (Ritz)	Lo	Crackers
54301100	Crackers, butter, reduced fat	Lo	Crackers
54301200	Cracker, snack, fat free	Lo	Crackers
54304000	Crackers, cheese	Lo	Crackers
54304005	Crackers, cheese (Cheez-It)	Lo	Crackers
54304020	Crackers, cheese (Goldfish)	Lo	Crackers
54304100	Crackers, cheese, reduced fat	Lo	Crackers
54304110	Crackers, cheese, reduced sodium	Lo	Crackers
54304150	Crackers, cheese, whole grain	Lo	Crackers
54304500	Cracker, high fiber, no added fat	Lo	Crackers
54305000	Crispbread, wheat, no added fat	Lo	Crackers
54305010	Crackers, crispbread	Lo	Crackers
54305020	Crackers, flatbread	Lo	Crackers
54305500	Crispbread, wheat or rye, extra cris	Lo	Crackers
54307000	Crackers, matzo	Lo	Crackers
54308000	Crackers, milk	Lo	Crackers
54309000	Crackers, oat	Lo	Crackers
54313000	Crackers, oyster	Lo	Crackers
54318000	Chips, rice	Lo	Savory Snacks
54318500	Rice cake	Lo	Crackers
54319000	Crackers, rice	Lo	Crackers
54319005	Crackers, rice and nuts	Lo	Crackers
54319010	Puffed rice cake	Lo	Crackers
54319020	Popcorn cake	Lo	Crackers
54319500	Rice paper	Lo	Crackers
54322000	Crispbread, rye, no added fat	Lo	Crackers
54325000	Crackers, saltine	Lo	Crackers
54325010	Crackers, saltine, reduced fat	Lo	Crackers
54325050	Crackers, saltine, whole wheat	Lo	Crackers
54325060	Crackers, saltine, multigrain	Lo	Crackers
54326000	Crackers, multigrain	Lo	Crackers
54327950	Crackers, cylindrical, peanut-butter	Lo	Crackers
54328000	Crackers, sandwich	Lo	Crackers
54328100	Crackers, sandwich, peanut butter f	Lo	Crackers
54328105	Crackers, sandwich, peanut butter f	Lo	Crackers
54328110	Crackers, sandwich, reduced fat, pe	Lo	Crackers
54328120	Crackers, whole grain, sandwich, pe	Lo	Crackers
54328200	Crackers, sandwich, cheese filled	Lo	Crackers
54328210	Crackers, sandwich, cheese filled (I	Lo	Crackers
54334000	Crackers, toast thins (rye, pumpern	Lo	Crackers
54336000	Crackers, water	Lo	Crackers
54336100	Crackers, wonton	Lo	Crackers

54337000	Cracker, 100% whole wheat	Lo	Crackers
54337010	Crackers, woven wheat	Lo	Crackers
54337020	Crackers, woven wheat, plain (Trisc	Lo	Crackers
54337030	Crackers, woven wheat, flavored (T	Lo	Crackers
54337050	Cracker, 100% whole wheat, reduc	Lo	Crackers
54337060	Crackers, woven wheat, reduced fa	Lo	Crackers
54337100	Crackers, whole wheat and bran	Lo	Crackers
54338000	Crackers, wheat	Lo	Crackers
54338010	Crackers, wheat, plain (Wheat Thin	Lo	Crackers
54338020	Crackers, wheat, flavored (Wheat T	Lo	Crackers
54338100	Crackers, wheat, reduced fat	Lo	Crackers
54339000	Crackers, corn	Lo	Crackers
54340100	Crackers, gluten free, plain	Lo	Crackers
54340110	Crackers, gluten free, flavored	Lo	Crackers
54350000	Crackers, baby food	Lo	Baby Foods
54350010	Gerber Finger Foods, Puffs, baby fo	Lo	Baby Foods
54350020	Finger Foods, Puffs, baby food	Lo	Baby Foods
54360000	Crunchy snacks, corn based, baby	Lo	Baby Foods
54401010	Salty snacks, corn or cornmeal bas	Lo	Savory Snacks
54401011	Corn nuts	Lo	Savory Snacks
54401020	Salty snacks, corn or cornmeal bas	Lo	Savory Snacks
54401021	Corn chips, plain	Lo	Savory Snacks
54401026	Corn chips, flavored	Lo	Savory Snacks
54401031	Corn chips, plain (Fritos)	Lo	Savory Snacks
54401035	Corn chips, flavored (Fritos)	Lo	Savory Snacks
54401050	Salty snacks, corn or cornmeal bas	Lo	Savory Snacks
54401055	Cheese flavored corn snacks	Lo	Savory Snacks
54401065	Cheese flavored corn snacks, redu	Lo	Savory Snacks
54401075	Tortilla chips, plain	Lo	Savory Snacks
54401080	Salty snacks, corn or cornmeal bas	Lo	Savory Snacks
54401081	Cheese flavored corn snacks (Chee	Lo	Savory Snacks
54401085	Tortilla chips, flavored	Lo	Savory Snacks
54401090	Corn chips, reduced sodium	Lo	Savory Snacks
54401100	Salty snacks, corn or cornmeal bas	Lo	Savory Snacks
54401110	Tortilla chips, nacho cheese flavor (	Lo	Savory Snacks
54401111	Tortilla chips, cool ranch flavor (Dor	Lo	Savory Snacks
54401112	Tortilla chips, other flavors (Doritos)	Lo	Savory Snacks
54401120	Salty snacks, corn or cornmeal bas	Lo	Savory Snacks
54401121	Tortilla chips, reduced fat, plain	Lo	Savory Snacks
54401122	Tortilla chips, reduced fat, flavored	Lo	Savory Snacks
54401150	Salty snacks, corn or cornmeal bas	Lo	Savory Snacks
54401170	Tortilla chips, low fat, unsalted	Lo	Savory Snacks
54401210	Salty snacks, corn based puffs and	Lo	Savory Snacks
54402080	Tortilla chips, reduced sodium	Lo	Savory Snacks
54402200	Snack mix	Lo	Savory Snacks
54402500	Salty snacks, wheat- and corn-base	Lo	Savory Snacks
54402600	Salty snacks, multigrain, whole grai	Lo	Savory Snacks
54402610	Potato chips, restructured, multigra	Lo	Savory Snacks
54402700	Pita chips	Lo	Crackers
54403000	Popcorn, popped in oil, unbuttered	Lo	Savory Snacks
54403001	Popcorn, NFS	Lo	Savory Snacks

54403005	Popcorn, movie theater, with added	Lo	Savory Snacks
54403006	Popcorn, movie theater, unbuttered	Lo	Savory Snacks
54403010	Popcorn, air-popped, unbuttered	Lo	Savory Snacks
54403020	Popcorn, popped in oil, buttered	Lo	Savory Snacks
54403040	Popcorn, air-popped, with added bu	Lo	Savory Snacks
54403045	Popcorn, popped in oil, unbuttered	Lo	Savory Snacks
54403046	Popcorn, popped in oil, with added	Lo	Savory Snacks
54403050	Popcorn, flavored	Lo	Savory Snacks
54403051	Popcorn, microwave, NFS	Lo	Savory Snacks
54403052	Popcorn, microwave, plain	Lo	Savory Snacks
54403053	Popcorn, microwave, plain, light	Lo	Savory Snacks
54403054	Popcorn, microwave, low sodium	Lo	Savory Snacks
54403055	Popcorn, microwave, unsalted	Lo	Savory Snacks
54403056	Popcorn, microwave, butter flavored	Lo	Savory Snacks
54403057	Popcorn, microwave, butter flavored	Lo	Savory Snacks
54403058	Popcorn, microwave, cheese flavor	Lo	Savory Snacks
54403059	Popcorn, microwave, kettle corn	Lo	Savory Snacks
54403060	Popcorn, popped in oil, lowfat, redu	Lo	Savory Snacks
54403061	Popcorn, microwave, kettle corn, lig	Lo	Savory Snacks
54403062	Popcorn, microwave, other flavored	Lo	Savory Snacks
54403070	Popcorn, popped in oil, lowfat	Lo	Savory Snacks
54403080	Popcorn, ready-to-eat packaged, N	Lo	Savory Snacks
54403081	Popcorn, ready-to-eat packaged, pl	Lo	Savory Snacks
54403082	Popcorn, ready-to-eat packaged, pl	Lo	Savory Snacks
54403083	Popcorn, ready-to-eat packaged, lo	Lo	Savory Snacks
54403085	Popcorn, ready-to-eat packaged, bu	Lo	Savory Snacks
54403086	Popcorn, ready-to-eat packaged, bu	Lo	Savory Snacks
54403087	Popcorn, ready-to-eat packaged, ch	Lo	Savory Snacks
54403088	Popcorn, ready-to-eat packaged, ch	Lo	Savory Snacks
54403089	Popcorn, ready-to-eat-packaged, ke	Lo	Savory Snacks
54403090	Popcorn, popped in oil, unsalted	Lo	Savory Snacks
54403091	Popcorn, ready-to-eat packaged, ke	Lo	Savory Snacks
54403092	Popcorn, ready-to-eat packaged, ot	Lo	Savory Snacks
54403110	Popcorn, caramel coated	Lo	Savory Snacks
54403120	Popcorn, caramel coated, with nuts	Lo	Savory Snacks
54403150	Popcorn, sugar syrup or caramel-co	Lo	Savory Snacks
54403160	Popcorn, chocolate coated	Lo	Savory Snacks
54404000	Popcorn chips, plain	Lo	Savory Snacks
54404010	Popcorn chips, other flavors	Lo	Savory Snacks
54404020	Popcorn chips, sweet flavors	Lo	Savory Snacks
54406010	Onion flavored rings	Lo	Savory Snacks
54406200	Shrimp chips	Lo	Savory Snacks
54408000	Pretzels, NFS	Lo	Savory Snacks
54408010	Pretzels, hard	Lo	Savory Snacks
54408015	Pretzels, hard, NFS	Lo	Savory Snacks
54408016	Pretzels, hard, plain, salted	Lo	Savory Snacks
54408017	Pretzels, hard, plain, lightly salted	Lo	Savory Snacks
54408020	Pretzels, soft	Lo	Savory Snacks
54408030	Pretzels, hard, plain, unsalted	Lo	Savory Snacks
54408035	Pretzels, hard, flavored	Lo	Savory Snacks
54408040	Pretzels, soft, unsalted	Lo	Savory Snacks

54408070	Pretzels, hard, multigrain	Lo	Savory Snacks
54408080	Pretzel, gluten free	Lo	Savory Snacks
54408081	Pretzels, hard, plain, gluten free	Lo	Savory Snacks
54408082	Pretzels, hard, flavored, gluten free	Lo	Savory Snacks
54408105	Pretzel chips, hard, plain	Lo	Savory Snacks
54408110	Pretzel chips, hard, flavored	Lo	Savory Snacks
54408190	Pretzels, hard, coated, NFS	Lo	Savory Snacks
54408200	Pretzels, hard, chocolate coated	Lo	Savory Snacks
54408210	Pretzels, hard, white chocolate coat	Lo	Savory Snacks
54408250	Pretzels, hard, yogurt coated	Lo	Savory Snacks
54408290	Pretzels, hard, filled, NFS	Lo	Savory Snacks
54408300	Pretzels, hard, cheese filled	Lo	Savory Snacks
54408310	Pretzels, hard, peanut butter filled	Lo	Savory Snacks
54408400	Pretzels, soft, NFS	Lo	Savory Snacks
54408405	Pretzels, soft, ready-to-eat, NFS	Lo	Savory Snacks
54408410	Pretzels, soft, ready-to-eat, salted,	Lo	Savory Snacks
54408411	Pretzels, soft, ready-to-eat, unsalte	Lo	Savory Snacks
54408415	Pretzels, soft, ready-to-eat, salted,	Lo	Savory Snacks
54408416	Pretzels, soft, ready-to-eat, unsalte	Lo	Savory Snacks
54408420	Pretzels, soft, ready-to-eat, cinnam	Lo	Savory Snacks
54408422	Pretzels, soft, ready-to-eat, coated	Lo	Savory Snacks
54408430	Pretzels, soft, ready-to-eat, topped	Lo	Savory Snacks
54408432	Pretzels, soft, ready-to-eat, topped	Lo	Savory Snacks
54408456	Pretzels, soft, from frozen, unsalted	Lo	Savory Snacks
54408470	Pretzels, soft, filled with cheese	Lo	Savory Snacks
54408475	Pretzels, soft, from school lunch	Lo	Savory Snacks
54408480	Pretzels, soft, multigrain	Lo	Savory Snacks
54408485	Pretzels, soft, gluten free	Lo	Savory Snacks
54408487	Pretzels, soft, gluten free, coated o	Lo	Savory Snacks
54420010	Multigrain mixture, pretzels, cereal	Lo	Savory Snacks
54420100	Oriental party mix, with peanuts, se	Lo	Savory Snacks
54420200	Multigrain mixture, bread sticks, ses	Lo	Savory Snacks
54420210	Multigrain chips (Sun Chips)	Lo	Savory Snacks
54420220	Snack mix, plain (Chex Mix)	Lo	Savory Snacks
54430010	Yogurt chips	Lo	Crackers
54440010	Bagel chips	Lo	Crackers
54440020	Cracker chips	Lo	Savory Snacks
55100005	Pancakes, NFS	Lo	Quick Breads and Bread Products
55100010	Pancakes, plain, from frozen	Lo	Quick Breads and Bread Products
55100015	Pancakes, plain, reduced fat, from	Lo	Quick Breads and Bread Products
55100020	Pancakes, with fruit, from frozen	Lo	Quick Breads and Bread Products
55100025	Pancakes, with chocolate, from froz	Lo	Quick Breads and Bread Products
55100030	Pancakes, whole grain, from frozen	Lo	Quick Breads and Bread Products
55100035	Pancakes, whole grain, reduced fat	Lo	Quick Breads and Bread Products
55100040	Pancakes, gluten free, from frozen	Lo	Quick Breads and Bread Products
55100050	Pancakes, plain, from fast food / re	Lo	Quick Breads and Bread Products
55100055	Pancakes, with fruit, from fast food	Lo	Quick Breads and Bread Products
55100060	Pancakes, with chocolate, from fast	Lo	Quick Breads and Bread Products
55100065	Pancakes, whole grain, from fast fo	Lo	Quick Breads and Bread Products
55100080	Pancakes, from school, NFS	Lo	Quick Breads and Bread Products
55101000	Pancakes, plain	Lo	Quick Breads and Bread Products

55101010	Pancakes, reduced calorie, high fib	Lo	Quick Breads and Bread Products
55101015	Pancakes, plain, reduced fat	Lo	Quick Breads and Bread Products
55101020	Pancakes, plain, fat free	Lo	Quick Breads and Bread Products
55103000	Pancakes, with fruit	Lo	Quick Breads and Bread Products
55103020	Pancakes, pumpkin	Lo	Quick Breads and Bread Products
55103100	Pancakes, with chocolate	Lo	Quick Breads and Bread Products
55105000	Pancakes, buckwheat	Lo	Quick Breads and Bread Products
55105100	Pancakes, cornmeal	Lo	Quick Breads and Bread Products
55105200	Pancakes, whole grain	Lo	Quick Breads and Bread Products
55105205	Pancakes, whole grain, reduced fat	Lo	Quick Breads and Bread Products
55105210	Pancakes, whole wheat, fat free	Lo	Quick Breads and Bread Products
55105300	Pancakes, sour dough	Lo	Quick Breads and Bread Products
55105400	Pancakes, rye	Lo	Quick Breads and Bread Products
55106000	Pancakes, gluten free	Lo	Quick Breads and Bread Products
55200010	Waffle, NFS	Lo	Quick Breads and Bread Products
55200020	Waffle, plain, from frozen	Lo	Quick Breads and Bread Products
55200030	Waffle, plain, reduced fat, from froz	Lo	Quick Breads and Bread Products
55200040	Waffle, fruit, from frozen	Lo	Quick Breads and Bread Products
55200050	Waffle, chocolate, from frozen	Lo	Quick Breads and Bread Products
55200060	Waffle, whole grain, from frozen	Lo	Quick Breads and Bread Products
55200070	Waffle, whole grain, reduced fat, fro	Lo	Quick Breads and Bread Products
55200080	Waffle, whole grain, fruit, from froze	Lo	Quick Breads and Bread Products
55200090	Waffle, gluten free, from frozen	Lo	Quick Breads and Bread Products
55200100	Waffle, plain, from fast food / restau	Lo	Quick Breads and Bread Products
55200110	Waffle, chocolate, from fast food / r	Lo	Quick Breads and Bread Products
55200120	Waffle, fruit, from fast food / restaur	Lo	Quick Breads and Bread Products
55200130	Waffle, whole grain, from fast food /	Lo	Quick Breads and Bread Products
55200200	Waffle, from school, NFS	Lo	Quick Breads and Bread Products
55201000	Waffle, plain	Lo	Quick Breads and Bread Products
55202000	Waffle, wheat, bran, or multigrain	Lo	Quick Breads and Bread Products
55203000	Waffle, fruit	Lo	Quick Breads and Bread Products
55203500	Waffle, nut and honey	Lo	Quick Breads and Bread Products
55203600	Waffle, chocolate	Lo	Quick Breads and Bread Products
55203700	Waffle, cinnamon	Lo	Quick Breads and Bread Products
55204000	Waffle, cornmeal	Lo	Quick Breads and Bread Products
55205000	Waffle, whole grain	Lo	Quick Breads and Bread Products
55206000	Waffle, oat bran	Lo	Quick Breads and Bread Products
55207000	Waffle, multi-bran	Lo	Quick Breads and Bread Products
55208000	Waffle, gluten free	Lo	Quick Breads and Bread Products
55211000	Waffle, plain, fat free	Lo	Quick Breads and Bread Products
55211050	Waffle, plain, reduced fat	Lo	Quick Breads and Bread Products
55212000	Waffle, whole grain, reduced fat	Lo	Quick Breads and Bread Products
55300010	French toast, NFS	Lo	Quick Breads and Bread Products
55300020	French toast, plain, from frozen	Lo	Quick Breads and Bread Products
55300050	French toast, plain, from fast food /	Lo	Quick Breads and Bread Products
55300055	French toast, whole grain, from fast	Lo	Quick Breads and Bread Products
55300060	French toast, from school, NFS	Lo	Quick Breads and Bread Products
55301000	French toast, plain	Lo	Quick Breads and Bread Products
55301015	French toast, whole grain	Lo	Quick Breads and Bread Products
55301020	French toast, whole grain, reduced	Lo	Quick Breads and Bread Products
55301025	French toast, gluten free	Lo	Quick Breads and Bread Products

55301030	French toast sticks, NFS	Lo	Quick Breads and Bread Products
55301031	French toast sticks, plain, from froz	Lo	Quick Breads and Bread Products
55301040	French toast sticks, plain, from fast	Lo	Quick Breads and Bread Products
55301048	French toast sticks, from school, NF	Lo	Quick Breads and Bread Products
55301050	French toast sticks, plain	Lo	Quick Breads and Bread Products
55301055	French toast sticks, whole grain	Lo	Quick Breads and Bread Products
55310100	Fried bread, Puerto Rican style	Lo	Quick Breads and Bread Products
55401000	Crepe, plain	Lo	Quick Breads and Bread Products
55501000	Chinese pancake	Lo	Quick Breads and Bread Products
55502000	Flour and water gravy	Lo	Condiments and Sauces
55610200	Dumpling, fried, Puerto Rican style	Lo	Mixed Dishes - Grain-based
55610300	Dumpling, plain	Lo	Quick Breads and Bread Products
55701000	Cake made with glutinous rice	Lo	Mixed Dishes - Grain-based
55702000	Cake or pancake made with rice flo	Lo	Mixed Dishes - Grain-based
55702100	Dosa (Indian), plain	Lo	Quick Breads and Bread Products
55703000	Cake made with glutinous rice and	Lo	Mixed Dishes - Grain-based
55801000	Funnel cake with sugar	Lo	Sweet Bakery Products
55801010	Funnel cake with sugar and fruit	Lo	Sweet Bakery Products
56101000	Macaroni, cooked, NS as to fat add	Lo	Cooked Grains
56101010	Macaroni, cooked, fat not added in	Lo	Cooked Grains
56101030	Macaroni, cooked, fat added in coo	Lo	Cooked Grains
56102000	Macaroni, whole wheat, cooked, NS	Lo	Cooked Grains
56102010	Macaroni, whole wheat, cooked, fat	Lo	Cooked Grains
56102020	Macaroni, whole wheat, cooked, fat	Lo	Cooked Grains
56103010	Macaroni, cooked, spinach, fat not	Lo	Cooked Grains
56104000	Pasta, vegetable, cooked	Lo	Cooked Grains
56104010	Macaroni, cooked, vegetable, fat no	Lo	Cooked Grains
56104020	Macaroni, cooked, vegetable, fat ad	Lo	Cooked Grains
56112000	Noodles, cooked	Lo	Cooked Grains
56112010	Noodles, cooked, fat not added in c	Lo	Cooked Grains
56112030	Noodles, cooked, fat added in cook	Lo	Cooked Grains
56113000	Noodles, whole grain, cooked	Lo	Cooked Grains
56113010	Noodles, cooked, whole wheat, fat	Lo	Cooked Grains
56113990	Noodles, vegetable, cooked	Lo	Cooked Grains
56114000	Noodles, cooked, spinach, fat not a	Lo	Cooked Grains
56114020	Noodles, cooked, spinach, fat adde	Lo	Cooked Grains
56116000	Noodles, chow mein	Lo	Crackers
56116990	Long rice noodles, made from mun	Lo	Cooked Grains
56117000	Long rice noodles (made from mun	Lo	Cooked Grains
56117010	Long rice noodles (made from mun	Lo	Cooked Grains
56117090	Rice noodles, cooked	Lo	Cooked Grains
56117100	Chow fun rice noodles, cooked, fat	Lo	Cooked Grains
56117110	Chow fun rice noodles, cooked, fat	Lo	Cooked Grains
56130000	Pasta, cooked	Lo	Cooked Grains
56130010	Spaghetti, cooked, fat not added in	Lo	Cooked Grains
56131000	Spaghetti, cooked, fat added in coo	Lo	Cooked Grains
56132990	Pasta, whole grain, cooked	Lo	Cooked Grains
56133000	Spaghetti, cooked, whole wheat, fat	Lo	Cooked Grains
56133010	Spaghetti, cooked, whole wheat, fat	Lo	Cooked Grains
56140100	Pasta, gluten free	Lo	Cooked Grains
56200300	Cereal, cooked, NFS	Lo	Cooked Cereals

56200350	Cereal, cooked, instant, NS as to gr	Lo	Cooked Cereals
56200390	Barley, NS as to fat	Lo	Cooked Grains
56200400	Barley, no added fat	Lo	Cooked Grains
56200490	Buckwheat groats, NS as to fat	Lo	Cooked Grains
56200500	Buckwheat groats, no added fat	Lo	Cooked Grains
56200510	Buckwheat groats, fat added	Lo	Cooked Grains
56200990	Grits, NS as to regular, quick, or ins	Lo	Cooked Cereals
56201000	Grits, NS as to regular, quick, or ins	Lo	Cooked Cereals
56201010	Grits, cooked, corn or hominy, regu	Lo	Cooked Cereals
56201020	Grits, cooked, corn or hominy, regu	Lo	Cooked Cereals
56201030	Grits, cooked, corn or hominy, regu	Lo	Cooked Cereals
56201040	Grits, NS as to regular, quick, or ins	Lo	Cooked Cereals
56201050	Grits, regular or quick, made with w	Lo	Cooked Cereals
56201051	Grits, regular or quick, made with w	Lo	Cooked Cereals
56201052	Grits, regular or quick, made with w	Lo	Cooked Cereals
56201055	Grits, regular or quick, made with m	Lo	Cooked Cereals
56201056	Grits, regular or quick, made with m	Lo	Cooked Cereals
56201057	Grits, regular or quick, made with m	Lo	Cooked Cereals
56201060	Grits, cooked, corn or hominy, with	Lo	Cooked Cereals
56201061	Grits, cooked, corn or hominy, with	Lo	Cooked Cereals
56201062	Grits, cooked, corn or hominy, with	Lo	Cooked Cereals
56201070	Grits, cooked, corn or hominy, with	Lo	Cooked Cereals
56201071	Grits, cooked, corn or hominy, with	Lo	Cooked Cereals
56201072	Grits, cooked, corn or hominy, with	Lo	Cooked Cereals
56201081	Grits, cooked, corn or hominy, with	Lo	Cooked Cereals
56201082	Grits, cooked, corn or hominy, with	Lo	Cooked Cereals
56201090	Grits, with cheese, NS as to fat	Lo	Cooked Cereals
56201091	Grits, with cheese, no added fat	Lo	Cooked Cereals
56201092	Grits, with cheese, fat added	Lo	Cooked Cereals
56201110	Grits, cooked, corn or hominy, quic	Lo	Cooked Cereals
56201120	Grits, cooked, corn or hominy, quic	Lo	Cooked Cereals
56201130	Grits, cooked, corn or hominy, quic	Lo	Cooked Cereals
56201210	Grits, instant, made with water, no a	Lo	Cooked Cereals
56201220	Grits, instant, made with water, fat a	Lo	Cooked Cereals
56201230	Grits, instant, made with water, NS	Lo	Cooked Cereals
56201240	Grits, cooked, flavored, corn or hom	Lo	Cooked Cereals
56201298	Grits, cooked, corn or hominy, NS a	Lo	Cooked Cereals
56201300	Grits, cooked, corn or hominy, NS a	Lo	Cooked Cereals
56201320	Grits, cooked, corn or hominy, regu	Lo	Cooked Cereals
56201322	Grits, cooked, corn or hominy, regu	Lo	Cooked Cereals
56201324	Grits, cooked, corn or hominy, regu	Lo	Cooked Cereals
56201330	Grits, cooked, corn or hominy, quic	Lo	Cooked Cereals
56201332	Grits, cooked, corn or hominy, quic	Lo	Cooked Cereals
56201340	Grits, instant, made with milk, fat ac	Lo	Cooked Cereals
56201342	Grits, instant, made with milk, no ac	Lo	Cooked Cereals
56201360	Grits, instant, made with non-dairy r	Lo	Cooked Cereals
56201510	Cornmeal mush, made with water	Lo	Cooked Cereals
56201515	Cornmeal mush, NS as to fat	Lo	Cooked Cereals
56201516	Cornmeal mush, no added fat	Lo	Cooked Cereals
56201517	Cornmeal mush, fat added	Lo	Cooked Cereals
56201520	Cornmeal mush, fried	Lo	Cooked Cereals

56201530	Cornmeal mush, made with milk	Lo	Cooked Cereals
56201540	Cornmeal, Puerto Rican Style	Lo	Cooked Cereals
56201550	Cornmeal dumpling	Lo	Quick Breads and Bread Products
56201600	Masa harina, cooked	Lo	Cooked Cereals
56201700	Cornstarch with milk, eaten as a ce	Lo	Cooked Cereals
56201750	Cornstarch, dry	Lo	Other
56201800	Cornstarch, hydrolyzed powder	Lo	Other
56201990	Millet, NS as to fat	Lo	Cooked Grains
56202000	Millet, no added fat	Lo	Cooked Grains
56202100	Millet, fat added	Lo	Cooked Grains
56202900	Oatmeal, from fast food, plain	Lo	Cooked Cereals
56202905	Oatmeal, from fast food, maple flav	Lo	Cooked Cereals
56202910	Oatmeal, from fast food, fruit flavor	Lo	Cooked Cereals
56202920	Oatmeal, from fast food, other flavo	Lo	Cooked Cereals
56202960	Oatmeal, NS as to regular, quick, o	Lo	Cooked Cereals
56202970	Oatmeal, cooked, quick (1 or 3 min	Lo	Cooked Cereals
56202980	Oatmeal, cooked, regular, NS as to	Lo	Cooked Cereals
56203000	Oatmeal, NS as to regular, quick, o	Lo	Cooked Cereals
56203010	Oatmeal, cooked, regular, fat not ad	Lo	Cooked Cereals
56203020	Oatmeal, cooked, quick (1 or 3 min	Lo	Cooked Cereals
56203030	Oatmeal, cooked, instant, fat not ad	Lo	Cooked Cereals
56203040	Oatmeal, NS as to regular, quick, o	Lo	Cooked Cereals
56203050	Oatmeal, cooked, regular, fat added	Lo	Cooked Cereals
56203055	Oatmeal, regular or quick, made wi	Lo	Cooked Cereals
56203056	Oatmeal, regular or quick, made wi	Lo	Cooked Cereals
56203057	Oatmeal, regular or quick, made wi	Lo	Cooked Cereals
56203060	Oatmeal, cooked, quick (1 or 3 min	Lo	Cooked Cereals
56203065	Oatmeal, regular or quick, made wi	Lo	Cooked Cereals
56203066	Oatmeal, regular or quick, made wi	Lo	Cooked Cereals
56203067	Oatmeal, regular or quick, made wi	Lo	Cooked Cereals
56203070	Oatmeal, cooked, instant, fat added	Lo	Cooked Cereals
56203075	Oatmeal, regular or quick, made wi	Lo	Cooked Cereals
56203076	Oatmeal, regular or quick, made wi	Lo	Cooked Cereals
56203077	Oatmeal, regular or quick, made wi	Lo	Cooked Cereals
56203080	Oatmeal, cooked, instant, NS as to	Lo	Cooked Cereals
56203086	Oatmeal, instant, plain, made with v	Lo	Cooked Cereals
56203087	Oatmeal, instant, plain, made with v	Lo	Cooked Cereals
56203096	Oatmeal, instant, plain, made with r	Lo	Cooked Cereals
56203097	Oatmeal, instant, plain, made with	Lo	Cooked Cereals
56203106	Oatmeal, instant, plain, made with r	Lo	Cooked Cereals
56203125	Oatmeal, instant, maple flavored, N	Lo	Cooked Cereals
56203130	Oatmeal, instant, maple flavored, no	Lo	Cooked Cereals
56203135	Oatmeal, instant, maple flavored, fa	Lo	Cooked Cereals
56203150	Oatmeal, instant, fruit flavored, NS	Lo	Cooked Cereals
56203155	Oatmeal, instant, fruit flavored, no a	Lo	Cooked Cereals
56203160	Oatmeal, instant, fruit flavored, fat a	Lo	Cooked Cereals
56203175	Oatmeal, instant, other flavors, no a	Lo	Cooked Cereals
56203180	Oatmeal, instant, other flavors, fat a	Lo	Cooked Cereals
56203200	Oatmeal with fruit, cooked	Lo	Cooked Cereals
56203210	Oatmeal, NS as to regular, quick, o	Lo	Cooked Cereals
56203211	Oatmeal, cooked, regular, made wi	Lo	Cooked Cereals

56203212	Oatmeal, cooked, quick (1 or 3 min	Lo	Cooked Cereals
56203213	Oatmeal, cooked, instant, made wit	Lo	Cooked Cereals
56203220	Oatmeal, NS as to regular, quick, o	Lo	Cooked Cereals
56203221	Oatmeal, cooked, regular, made wi	Lo	Cooked Cereals
56203222	Oatmeal, cooked, quick (1 or 3 min	Lo	Cooked Cereals
56203223	Oatmeal, cooked, instant, made wit	Lo	Cooked Cereals
56203230	Oatmeal, NS as to regular, quick, o	Lo	Cooked Cereals
56203231	Oatmeal, cooked, regular, made wi	Lo	Cooked Cereals
56203232	Oatmeal, cooked, quick (1 or 3 min	Lo	Cooked Cereals
56203233	Oatmeal, cooked, instant, made wit	Lo	Cooked Cereals
56203510	Oatmeal, reduced sugar, plain, no a	Lo	Cooked Cereals
56203540	Oatmeal, made with milk and sugar	Lo	Cooked Cereals
56203550	Oatmeal, reduced sugar, flavored, I	Lo	Cooked Cereals
56203555	Oatmeal, reduced sugar, flavored, r	Lo	Cooked Cereals
56203560	Oatmeal, reduced sugar, flavored, f	Lo	Cooked Cereals
56203600	Oatmeal, multigrain, NS as to fat	Lo	Cooked Cereals
56203610	Oatmeal, multigrain, no added fat	Lo	Cooked Cereals
56203620	Oatmeal, multigrain, fat added	Lo	Cooked Cereals
56204000	Quinoa, NS as to fat	Lo	Cooked Grains
56204005	Quinoa, no added fat	Lo	Cooked Grains
56204010	Quinoa, fat added	Lo	Cooked Grains
56204980	Rice, white, cooked, converted, NS	Lo	Cooked Grains
56204990	Rice, white, cooked, regular, NS as	Lo	Cooked Grains
56205000	Rice, cooked, NFS	Lo	Cooked Grains
56205001	Rice, white, cooked, NS as to fat	Lo	Cooked Grains
56205002	Rice, white, cooked, made with oil	Lo	Cooked Grains
56205004	Rice, white, cooked, made with butt	Lo	Cooked Grains
56205006	Rice, white, cooked, made with mar	Lo	Cooked Grains
56205007	Rice, white, cooked, fat added, NS	Lo	Cooked Grains
56205008	Rice, white, cooked, no added fat	Lo	Cooked Grains
56205010	Rice, white, cooked, regular, fat not	Lo	Cooked Grains
56205011	Rice, brown, cooked, NS as to fat	Lo	Cooked Grains
56205012	Rice, brown, cooked, fat added, ma	Lo	Cooked Grains
56205014	Rice, brown, cooked, made with bu	Lo	Cooked Grains
56205016	Rice, brown, cooked, made with ma	Lo	Cooked Grains
56205017	Rice, brown, cooked, fat added, NS	Lo	Cooked Grains
56205018	Rice, brown, cooked, no added fat	Lo	Cooked Grains
56205020	Rice, white, cooked, instant, NS as	Lo	Cooked Grains
56205030	Rice, white, cooked, instant, fat not	Lo	Cooked Grains
56205040	Rice, white, cooked, converted, fat	Lo	Cooked Grains
56205050	Rice, cream of, cooked, no added f	Lo	Cooked Cereals
56205060	Rice, cooked, with milk	Lo	Cooked Grains
56205070	Rice, sweet, cooked with honey	Lo	Cooked Grains
56205080	Rice, creamed, made with milk and	Lo	Cooked Cereals
56205090	Rice, cream of, cooked, fat added	Lo	Cooked Cereals
56205092	Rice, cream of, cooked, NS as to fa	Lo	Cooked Cereals
56205094	Rice, cream of, cooked, made with	Lo	Cooked Cereals
56205101	Congee	Lo	Cooked Grains
56205110	Rice, brown, cooked, regular, fat no	Lo	Cooked Grains
56205120	Rice, brown, cooked, regular, NS as	Lo	Cooked Grains
56205130	Yellow rice, cooked, NS as to fat	Lo	Cooked Grains

56205150	Yellow rice, cooked, no added fat	Lo	Cooked Grains
56205170	Yellow rice, cooked, fat added	Lo	Cooked Grains
56205190	Rice, white, cooked, glutinous	Lo	Cooked Grains
56205200	Rice, frozen dessert, nondairy, flavo	Lo	Other Desserts
56205205	Rice, wild, 100%, cooked, NS as to	Lo	Cooked Grains
56205210	Rice, wild, 100%, cooked, no added	Lo	Cooked Grains
56205215	Rice, wild, 100%, cooked, fat added	Lo	Cooked Grains
56205230	Rice dessert bar, frozen, flavors oth	Lo	Other Desserts
56205300	Rice, white and wild, cooked, no ad	Lo	Cooked Grains
56205310	Rice, brown and wild, cooked, no a	Lo	Cooked Grains
56205320	Rice, white and wild, cooked, fat ad	Lo	Cooked Grains
56205330	Rice, white and wild, cooked, NS as	Lo	Cooked Grains
56205340	Rice, brown and wild, cooked, fat a	Lo	Cooked Grains
56205350	Rice, brown and wild, cooked, NS a	Lo	Cooked Grains
56205400	Rice, cooked, NS as to type, fat add	Lo	Cooked Grains
56205410	Rice, white, cooked with fat, Puerto	Lo	Cooked Grains
56205420	Rice, white, cooked, regular, fat add	Lo	Cooked Grains
56205430	Rice, white, cooked, instant, fat add	Lo	Cooked Grains
56205440	Rice, white, cooked, converted, fat	Lo	Cooked Grains
56205510	Rice, brown, cooked, regular, fat ad	Lo	Cooked Grains
56205530	Rice, brown, cooked, instant, NS as	Lo	Cooked Grains
56205540	Rice, brown, cooked, instant, fat no	Lo	Cooked Grains
56205550	Rice, brown, cooked, instant, fat ad	Lo	Cooked Grains
56206970	Wheat, cream of, cooked, quick, NS	Lo	Cooked Cereals
56206990	Cream of wheat, NS as to regular, c	Lo	Cooked Cereals
56207000	Cream of wheat, NS as to regular, c	Lo	Cooked Cereals
56207005	Cream of wheat, NS as to regular, c	Lo	Cooked Cereals
56207010	Wheat, cream of, cooked, regular, f	Lo	Cooked Cereals
56207016	Cream of wheat, regular or quick, m	Lo	Cooked Cereals
56207017	Cream of wheat, regular or quick, m	Lo	Cooked Cereals
56207020	Wheat, cream of, cooked, quick, fat	Lo	Cooked Cereals
56207022	Cream of wheat, regular or quick, m	Lo	Cooked Cereals
56207023	Cream of wheat, regular or quick, m	Lo	Cooked Cereals
56207027	Cream of wheat, regular or quick, m	Lo	Cooked Cereals
56207030	Cream of wheat, instant, made with	Lo	Cooked Cereals
56207040	Wheat, cream of, cooked, made wit	Lo	Cooked Cereals
56207060	Cream of wheat, instant, made with	Lo	Cooked Cereals
56207080	Wheat, cream of, cooked, NS as to	Lo	Cooked Cereals
56207086	Wheat, cream of, cooked, regular, r	Lo	Cooked Cereals
56207087	Wheat, cream of, cooked, regular, r	Lo	Cooked Cereals
56207092	Wheat, cream of, cooked, quick, ma	Lo	Cooked Cereals
56207094	Cream of wheat, instant, made with	Lo	Cooked Cereals
56207095	Cream of wheat, instant, made with	Lo	Cooked Cereals
56207102	Cream of wheat, instant, made with	Lo	Cooked Cereals
56207110	Bulgur, no added fat	Lo	Cooked Grains
56207120	Bulgur, fat added	Lo	Cooked Grains
56207130	Bulgur, NS as to fat	Lo	Cooked Grains
56207150	Couscous, plain, cooked, fat not ad	Lo	Cooked Grains
56207160	Couscous, plain, cooked	Lo	Cooked Grains
56207180	Couscous, plain, cooked, fat added	Lo	Cooked Grains
56207190	Whole wheat cereal, cooked, NS as	Lo	Cooked Cereals

56207200	Whole wheat cereal, cooked, no ad	Lo	Cooked Cereals
56207210	Whole wheat cereal, cooked, fat ad	Lo	Cooked Cereals
56207212	Whole wheat cereal, cooked, made	Lo	Cooked Cereals
56207220	Wheat, cream of, cooked, regular, f	Lo	Cooked Cereals
56207230	Wheat, cream of, cooked, quick, fa	Lo	Cooked Cereals
56207300	Whole wheat cereal, wheat and bar	Lo	Cooked Cereals
56207330	Whole wheat cereal, wheat and bar	Lo	Cooked Cereals
56207340	Whole wheat cereal, wheat and bar	Lo	Cooked Cereals
56207342	Whole wheat cereal, wheat and bar	Lo	Cooked Cereals
56207350	Wheat cereal, chocolate flavored, c	Lo	Cooked Cereals
56207360	Wheat cereal, chocolate flavored, c	Lo	Cooked Cereals
56207370	Wheat cereal, chocolate flavored, c	Lo	Cooked Cereals
56208500	Oat bran cereal, cooked, no added	Lo	Cooked Cereals
56208510	Oat bran cereal, cooked, fat added	Lo	Cooked Cereals
56208520	Oat bran cereal, cooked, NS as to f	Lo	Cooked Cereals
56208530	Oat bran cereal, cooked, made with	Lo	Cooked Cereals
56209000	Cream of rye	Lo	Cooked Cereals
56210000	Cereal, nestum	Lo	Baby Foods
57000000	Cereal, NFS	Lo	Ready-to-Eat Cereals
57000050	Kashi cereal, NS as to ready to eat	Lo	Ready-to-Eat Cereals
57000100	Cereal, oat, NFS	Lo	Ready-to-Eat Cereals
57100100	Cereal, ready-to-eat, NFS	Lo	Ready-to-Eat Cereals
57100400	Character cereals, TV or movie, Ge	Lo	Ready-to-Eat Cereals
57100500	Character cereals, TV or movie, Ke	Lo	Ready-to-Eat Cereals
57101000	Cereal (Kellogg's All-Bran)	Lo	Ready-to-Eat Cereals
57101020	All-Bran with Extra Fiber	Lo	Ready-to-Eat Cereals
57102000	Cereal (Alpen)	Lo	Ready-to-Eat Cereals
57103000	Cereal (Post Alpha-Bits)	Lo	Ready-to-Eat Cereals
57103020	Alpha-bits with marshmallows	Lo	Ready-to-Eat Cereals
57103050	Amaranth Flakes	Lo	Ready-to-Eat Cereals
57103100	Cereal (General Mills Cheerios App	Lo	Ready-to-Eat Cereals
57103500	Apple Cinnamon Squares Mini-Whe	Lo	Ready-to-Eat Cereals
57104000	Cereal (Kellogg's Apple Jacks)	Lo	Ready-to-Eat Cereals
57106050	Cereal (Post Great Grains Banana	Lo	Ready-to-Eat Cereals
57106060	Cereal (General Mills Cheerios Ban	Lo	Ready-to-Eat Cereals
57106100	Cereal (General Mills Basic 4)	Lo	Ready-to-Eat Cereals
57106250	Cereal (General Mills Kix Berry Ber	Lo	Ready-to-Eat Cereals
57106260	Cereal (General Mills Cheerios Ber	Lo	Ready-to-Eat Cereals
57106530	Cereal (Post Selects Blueberry Mor	Lo	Ready-to-Eat Cereals
57107000	Cereal (General Mills Boo Berry)	Lo	Ready-to-Eat Cereals
57110000	Cereal (Kellogg's All-Bran Bran Buc	Lo	Ready-to-Eat Cereals
57111000	Bran Chex	Lo	Ready-to-Eat Cereals
57117000	Cereal (Quaker Cap'n Crunch)	Lo	Ready-to-Eat Cereals
57117500	Cereal (Quaker Christmas Crunch)	Lo	Ready-to-Eat Cereals
57119000	Cereal (Quaker Cap'n Crunch's Cru	Lo	Ready-to-Eat Cereals
57120000	Cereal (Quaker Cap'n Crunch's Pea	Lo	Ready-to-Eat Cereals
57123000	Cereal (General Mills Cheerios)	Lo	Ready-to-Eat Cereals
57124000	Chex cereal, NFS	Lo	Ready-to-Eat Cereals
57124030	Cereal (General Mills Chex Chocola	Lo	Ready-to-Eat Cereals
57124050	Cereal (General Mills Chex Cinnam	Lo	Ready-to-Eat Cereals
57124100	Cereal (General Mills Cheerios Cho	Lo	Ready-to-Eat Cereals

57124200	Cereal, chocolate flavored, frosted,	Lo	Ready-to-Eat Cereals
57124300	Cereal (General Mills Lucky Charm	Lo	Ready-to-Eat Cereals
57124500	Cinnamon Grahams, General Mills	Lo	Ready-to-Eat Cereals
57124900	Cereal (Kellogg's Cinnabon)	Lo	Ready-to-Eat Cereals
57125000	Cereal (General Mills Cinnamon To	Lo	Ready-to-Eat Cereals
57125010	Cereal (General Mills 25% Less Su	Lo	Ready-to-Eat Cereals
57125900	Cereal (General Mills Honey Nut Cl	Lo	Ready-to-Eat Cereals
57126000	Cereal (Kellogg's Cocoa Krispies)	Lo	Ready-to-Eat Cereals
57126500	Cocoa Blasts, Quaker	Lo	Ready-to-Eat Cereals
57127000	Cereal (Post Cocoa Pebbles)	Lo	Ready-to-Eat Cereals
57128000	Cereal (General Mills Cocoa Puffs)	Lo	Ready-to-Eat Cereals
57128005	Cereal (General Mills 25% Less Su	Lo	Ready-to-Eat Cereals
57128880	Complete Oat Bran Flakes, Kellogg	Lo	Ready-to-Eat Cereals
57130000	Cereal (General Mills Cookie Crisp)	Lo	Ready-to-Eat Cereals
57131000	Cereal (Quaker Corn Bran Crunch)	Lo	Ready-to-Eat Cereals
57132000	Cereal (General Mills Chex Corn)	Lo	Ready-to-Eat Cereals
57134000	Cereal, corn flakes	Lo	Ready-to-Eat Cereals
57134090	Corn flakes, low sodium	Lo	Ready-to-Eat Cereals
57135000	Cereal (Kellogg's Corn Flakes)	Lo	Ready-to-Eat Cereals
57137000	Cereal, corn puffs	Lo	Ready-to-Eat Cereals
57138000	Total Corn Flakes	Lo	Ready-to-Eat Cereals
57139000	Cereal (General Mills Count Chocul	Lo	Ready-to-Eat Cereals
57143000	Cereal (Kellogg's Cracklin' Oat Brar	Lo	Ready-to-Eat Cereals
57143500	Cereal (Post Great Grains, Cranber	Lo	Ready-to-Eat Cereals
57144000	Crisp Crunch	Lo	Ready-to-Eat Cereals
57148000	Cereal (Kellogg's Crispix)	Lo	Ready-to-Eat Cereals
57148500	Cereal, crispy brown rice	Lo	Ready-to-Eat Cereals
57148600	Harmony cereal, General Mills	Lo	Ready-to-Eat Cereals
57151000	Cereal, crispy rice	Lo	Ready-to-Eat Cereals
57152000	Crispy Wheats'n Raisins	Lo	Ready-to-Eat Cereals
57160000	Curves Fruit and Nut Crunch Cerea	Lo	Ready-to-Eat Cereals
57201800	Disney cereals, Kellogg's	Lo	Ready-to-Eat Cereals
57201900	Cereal (General Mills Dora The Exp	Lo	Ready-to-Eat Cereals
57206000	Cereal (Famila)	Lo	Ready-to-Eat Cereals
57206700	Cereal (General Mills Fiber One)	Lo	Ready-to-Eat Cereals
57206705	Cereal (General Mills Fiber One Ca	Lo	Ready-to-Eat Cereals
57206710	Cereal (General Mills Fiber One Ho	Lo	Ready-to-Eat Cereals
57206715	Cereal (General Mills Fiber One Ra	Lo	Ready-to-Eat Cereals
57206800	Cereal (Healt Valley Fiber 7 Flakes)	Lo	Ready-to-Eat Cereals
57207000	Cereal, bran flakes	Lo	Ready-to-Eat Cereals
57208000	Cereal (Kellogg's All-Bran Complete	Lo	Ready-to-Eat Cereals
57209000	Cereal (Post Bran Flakes)	Lo	Ready-to-Eat Cereals
57211000	Cereal (General Mills Frankenberry	Lo	Ready-to-Eat Cereals
57212100	French Toast Crunch, General Mills	Lo	Ready-to-Eat Cereals
57213000	Cereal (Kellogg's Froot Loops)	Lo	Ready-to-Eat Cereals
57213005	Froot Loops Cereal Straws	Lo	Ready-to-Eat Cereals
57213010	Cereal (Kellogg's Froot Loops Mars	Lo	Ready-to-Eat Cereals
57213850	Cereal (General Mills Cheerios Fros	Lo	Ready-to-Eat Cereals
57214000	Cereal (Kellogg's Frosted Mini-Whe	Lo	Ready-to-Eat Cereals
57214100	Frosted Wheat Bites	Lo	Ready-to-Eat Cereals
57215000	Frosty O's	Lo	Ready-to-Eat Cereals

57216000	Cereal, frosted rice	Lo	Ready-to-Eat Cereals
57218000	Cereal (Kellogg's Frosted Krispies)	Lo	Ready-to-Eat Cereals
57219000	Cereal, fruit and fiber	Lo	Ready-to-Eat Cereals
57221000	Cereal, fiber and fruit	Lo	Ready-to-Eat Cereals
57221650	Fruit Harvest cereal, Kellogg's	Lo	Ready-to-Eat Cereals
57221700	Cereal, fruit rings	Lo	Ready-to-Eat Cereals
57221800	Cereal, fruit whirls	Lo	Ready-to-Eat Cereals
57221810	Cereal (General Mills Cheerios Frui	Lo	Ready-to-Eat Cereals
57223000	Cereal (Post Fruity Pebbles)	Lo	Ready-to-Eat Cereals
57224000	Cereal (General Mills Golden Graha	Lo	Ready-to-Eat Cereals
57227000	Cereal, granola	Lo	Ready-to-Eat Cereals
57228000	Granola, homemade	Lo	Ready-to-Eat Cereals
57229000	Cereal (Kellogg's Low Fat Granola)	Lo	Ready-to-Eat Cereals
57229500	Cereal (Kellogg's Low Fat Granola w	Lo	Ready-to-Eat Cereals
57230000	Cereal (Post Grape-Nuts)	Lo	Ready-to-Eat Cereals
57231000	Cereal (Post Grape-Nuts Flakes)	Lo	Ready-to-Eat Cereals
57231200	Cereal (Post Great Grains Raisins,	Lo	Ready-to-Eat Cereals
57231250	Cereal (Post Great Grains Double P	Lo	Ready-to-Eat Cereals
57232100	Healthy Choice Almond Crunch with	Lo	Ready-to-Eat Cereals
57237100	Cereal (Post Honey Bunches of Oa	Lo	Ready-to-Eat Cereals
57237200	Cereal (Post Honey Bunches of Oa	Lo	Ready-to-Eat Cereals
57237300	Cereal (Post Honey Bunches of Oa	Lo	Ready-to-Eat Cereals
57237310	Cereal (Post Honey Bunches of Oa	Lo	Ready-to-Eat Cereals
57237900	Cereal (Post Honey Bunches of Oa	Lo	Ready-to-Eat Cereals
57238000	Cereal (Post Honeycomb)	Lo	Ready-to-Eat Cereals
57239000	Honeycomb, strawberry	Lo	Ready-to-Eat Cereals
57239100	Cereal (Kellogg's Honey Crunch Co	Lo	Ready-to-Eat Cereals
57240100	Cereal (General Mills Chex Honey N	Lo	Ready-to-Eat Cereals
57241000	Cereal (General Mills Cheerios Hon	Lo	Ready-to-Eat Cereals
57241200	Cereal (Post Shredded Wheat Hone	Lo	Ready-to-Eat Cereals
57243000	Cereal (Kellogg's Honey Smacks)	Lo	Ready-to-Eat Cereals
57245000	Just Right Fruit and Nut (formerly Ju	Lo	Ready-to-Eat Cereals
57301100	Kaboom	Lo	Ready-to-Eat Cereals
57301500	Cereal (Kashi 7 Whole Grain Puffs)	Lo	Ready-to-Eat Cereals
57301505	Cereal (Kashi Autumn Wheat)	Lo	Ready-to-Eat Cereals
57301510	Cereal (Kashi GOLEAN)	Lo	Ready-to-Eat Cereals
57301511	Cereal (Kashi GOLEAN Crunch)	Lo	Ready-to-Eat Cereals
57301512	Cereal (Kashi GOLEAN Crunch Ho	Lo	Ready-to-Eat Cereals
57301520	Cereal (Kashi Good Friends)	Lo	Ready-to-Eat Cereals
57301530	Cereal (Kashi Heart to Heart Honey	Lo	Ready-to-Eat Cereals
57301535	Cereal (Kashi Heart to Heart Oat Fl	Lo	Ready-to-Eat Cereals
57301540	Cereal (Kashi Honey Sunshine Squ	Lo	Ready-to-Eat Cereals
57302100	Cereal (Quaker King Vitaman)	Lo	Ready-to-Eat Cereals
57303100	Cereal (General Mills Kix)	Lo	Ready-to-Eat Cereals
57303105	Cereal (General Mills Honey Kix)	Lo	Ready-to-Eat Cereals
57303200	Cereal (Kellogg's Krave)	Lo	Ready-to-Eat Cereals
57304100	Cereal (Quaker Life)	Lo	Ready-to-Eat Cereals
57305100	Cereal (General Mills Lucky Charms	Lo	Ready-to-Eat Cereals
57305150	Cereal, frosted oat cereal with mars	Lo	Ready-to-Eat Cereals
57305160	Cereal (Malt-O-Meal Blueberry Muf	Lo	Ready-to-Eat Cereals
57305165	Cereal (Malt-O-Meal Cinnamon Toa	Lo	Ready-to-Eat Cereals

57305170	Cereal (Malt-O-Meal Coco-Roos)	Lo	Ready-to-Eat Cereals
57305174	Cereal (Malt-O-Meal Colossal Crun	Lo	Ready-to-Eat Cereals
57305175	Cereal (Malt-O-Meal Cocoa Dyno-B	Lo	Ready-to-Eat Cereals
57305180	Cereal (Malt-O-Meal Corn Bursts)	Lo	Ready-to-Eat Cereals
57305200	Cereal (Malt-O-Meal Crispy Rice)	Lo	Ready-to-Eat Cereals
57305210	Cereal (Malt-O-Meal Frosted Flakes	Lo	Ready-to-Eat Cereals
57305215	Cereal (Malt-O-Meal Frosted Mini S	Lo	Ready-to-Eat Cereals
57305300	Cereal (Malt-O-Meal Fruity Dyno-Bi	Lo	Ready-to-Eat Cereals
57305400	Cereal (Malt-O-Meal Honey Grahan	Lo	Ready-to-Eat Cereals
57305500	Cereal (Malt-O-Meal Honey Nut To	Lo	Ready-to-Eat Cereals
57305600	Cereal (Malt-O-Meal Marshmallow	Lo	Ready-to-Eat Cereals
57306100	Malt-O-Meal Puffed Rice	Lo	Ready-to-Eat Cereals
57306120	Malt-O-Meal Puffed Wheat	Lo	Ready-to-Eat Cereals
57306130	Cereal (Malt-O-Meal Raisin Bran)	Lo	Ready-to-Eat Cereals
57306500	Cereal (Malt-O-Meal Golden Puffs)	Lo	Ready-to-Eat Cereals
57306700	Cereal (Malt-O-Meal Toasted Oat C	Lo	Ready-to-Eat Cereals
57306800	Cereal (Malt-O-Meal Tootie Fruities	Lo	Ready-to-Eat Cereals
57307010	Cereal (Post Maple Pecan Crunch)	Lo	Ready-to-Eat Cereals
57307150	Marshmallow Safari, Quaker	Lo	Ready-to-Eat Cereals
57307500	Cereal, millet, puffed	Lo	Ready-to-Eat Cereals
57307600	Mini-Swirlz Cinnamon Bun Cereal, I	Lo	Ready-to-Eat Cereals
57308150	Mueslix cereal, NFS	Lo	Ready-to-Eat Cereals
57308190	Cereal, muesli	Lo	Ready-to-Eat Cereals
57308300	Multi Bran Chex	Lo	Ready-to-Eat Cereals
57308400	Cereal (General Mills Cheerios Mul	Lo	Ready-to-Eat Cereals
57309100	Cereal (Nature Valley Granola)	Lo	Ready-to-Eat Cereals
57316200	Cereal, nutty nuggets	Lo	Ready-to-Eat Cereals
57316300	Cereal (Health Valley Oat Bran Flak	Lo	Ready-to-Eat Cereals
57316380	Cereal (General Mills Cheerios Oat	Lo	Ready-to-Eat Cereals
57316385	Cereal (General Mills Cheerios Prot	Lo	Ready-to-Eat Cereals
57316410	Oatmeal Crisp, Apple Cinnamon (fo	Lo	Ready-to-Eat Cereals
57316450	Cereal (General Mills Oatmeal Cris	Lo	Ready-to-Eat Cereals
57316500	Cereal (General Mills Oatmeal Cris	Lo	Ready-to-Eat Cereals
57316710	Cereal (Quaker Honey Graham Oh'	Lo	Ready-to-Eat Cereals
57316750	Oh's, Fruitangy, Quaker	Lo	Ready-to-Eat Cereals
57318000	100% Bran	Lo	Ready-to-Eat Cereals
57319000	100% Natural Cereal, plain, Quaker	Lo	Ready-to-Eat Cereals
57319500	Sun Country 100% Natural Granola	Lo	Ready-to-Eat Cereals
57320500	Cereal (Quaker Granola with Oats,	Lo	Ready-to-Eat Cereals
57321500	100 % Natural Wholegrain Cereal w	Lo	Ready-to-Eat Cereals
57321700	Optimum, Nature's Path	Lo	Ready-to-Eat Cereals
57321800	Optimum Slim, Nature's Path	Lo	Ready-to-Eat Cereals
57321900	Cereal (Nature's Path Organic Flax	Lo	Ready-to-Eat Cereals
57321905	Organic Flax Plus, Pumpkin Granol	Lo	Ready-to-Eat Cereals
57322500	Oreo O's cereal, Post	Lo	Ready-to-Eat Cereals
57323000	Cereal (Quaker Sweet Crunch)	Lo	Ready-to-Eat Cereals
57323050	Sweet Puffs, Quaker	Lo	Ready-to-Eat Cereals
57324000	Peanut Butter Toast Crunch, Gener	Lo	Ready-to-Eat Cereals
57325000	Cereal (Kellogg's Product 19)	Lo	Ready-to-Eat Cereals
57326000	Cereal (Barbara's Puffins)	Lo	Ready-to-Eat Cereals
57327450	Cereal (Quaker Toasted Oat Bran)	Lo	Ready-to-Eat Cereals

57327500	Cereal (Quaker Oatmeal Squares)	Lo	Ready-to-Eat Cereals
57328000	Cereal (Quaker Quisp)	Lo	Ready-to-Eat Cereals
57329000	Cereal, raisin bran	Lo	Ready-to-Eat Cereals
57330000	Cereal (Kellogg's Raisin Bran)	Lo	Ready-to-Eat Cereals
57330010	Cereal (Kellogg's Raisin Bran Crunc	Lo	Ready-to-Eat Cereals
57331000	Cereal (Post Raisin Bran)	Lo	Ready-to-Eat Cereals
57332050	Cereal (General Mills Total Raisin B	Lo	Ready-to-Eat Cereals
57332100	Cereal (General Mills Raisin Nut Br	Lo	Ready-to-Eat Cereals
57335550	Cereal (General Mills Reese's Puffs	Lo	Ready-to-Eat Cereals
57336000	Cereal (General Mills Chex Rice)	Lo	Ready-to-Eat Cereals
57337000	Cereal, rice flakes	Lo	Ready-to-Eat Cereals
57339000	Cereal (Kellogg's Rice Krispies)	Lo	Ready-to-Eat Cereals
57339100	Rice Krispies with Real Strawberrie	Lo	Ready-to-Eat Cereals
57339500	Cereal (Kellogg's Rice Krispies Trea	Lo	Ready-to-Eat Cereals
57340000	Cereal, puffed rice	Lo	Ready-to-Eat Cereals
57340700	Scooby Doo cereal, Kellogg's	Lo	Ready-to-Eat Cereals
57341000	Cereal (Post Shredded Wheat'n Bra	Lo	Ready-to-Eat Cereals
57341200	Cereal (Kellogg's Smart Start Stron	Lo	Ready-to-Eat Cereals
57341300	Cereal (Kellogg's Smorz)	Lo	Ready-to-Eat Cereals
57342010	Smorz, Kellogg's	Lo	Ready-to-Eat Cereals
57344000	Cereal (Kellogg's Special K)	Lo	Ready-to-Eat Cereals
57344001	Cereal (Kellogg's Special K Blueber	Lo	Ready-to-Eat Cereals
57344005	Cereal (Kellogg's Special K Chocola	Lo	Ready-to-Eat Cereals
57344007	Cereal (Kellogg's Special K Low Fa	Lo	Ready-to-Eat Cereals
57344010	Cereal (Kellogg's Special K Red Be	Lo	Ready-to-Eat Cereals
57344015	Cereal (Kellogg's Special K Fruit &	Lo	Ready-to-Eat Cereals
57344020	Cereal (Kellogg's Special K Vanilla	Lo	Ready-to-Eat Cereals
57344025	Cereal (Kellogg's Special K Cinnam	Lo	Ready-to-Eat Cereals
57346500	Oatmeal Honey Nut Heaven, Quake	Lo	Ready-to-Eat Cereals
57347000	Cereal (Kellogg's Corn Pops)	Lo	Ready-to-Eat Cereals
57347500	Strawberry Squares Mini-Wheats, K	Lo	Ready-to-Eat Cereals
57348000	Cereal, frosted corn flakes	Lo	Ready-to-Eat Cereals
57349000	Cereal (Kellogg's Frosted Flakes)	Lo	Ready-to-Eat Cereals
57349020	Cereal (Kellogg's Frosted Flakes, R	Lo	Ready-to-Eat Cereals
57355000	Cereal (Post Golden Crisp)	Lo	Ready-to-Eat Cereals
57401100	Cereal, toasted oat	Lo	Ready-to-Eat Cereals
57403100	Toasties, Post	Lo	Ready-to-Eat Cereals
57404100	Malt-O-Meal Toasty O's	Lo	Ready-to-Eat Cereals
57404200	Malt-O-Meal Apple and Cinnamon T	Lo	Ready-to-Eat Cereals
57406100	Cereal (General Mills Total)	Lo	Ready-to-Eat Cereals
57406105	Total Cranberry Crunch	Lo	Ready-to-Eat Cereals
57407100	Cereal (General Mills Trix)	Lo	Ready-to-Eat Cereals
57407110	Cereal (General Mills 25% Less Su	Lo	Ready-to-Eat Cereals
57408100	Cereal (Uncle Sam)	Lo	Ready-to-Eat Cereals
57409100	Cereal (Post Waffle Crisp)	Lo	Ready-to-Eat Cereals
57410000	Cereal (Weetabix Whole Grain)	Lo	Ready-to-Eat Cereals
57411000	Cereal (General Mills Chex Wheat)	Lo	Ready-to-Eat Cereals
57412000	Wheat germ, plain	Lo	Other
57416000	Cereal, puffed wheat, plain	Lo	Ready-to-Eat Cereals
57416010	Cereal, puffed wheat, sweetened	Lo	Ready-to-Eat Cereals
57417000	Cereal (Post Shredded Wheat)	Lo	Ready-to-Eat Cereals

57418000	Cereal (General Mills Wheaties)	Lo	Ready-to-Eat Cereals
57419000	Cereal (General Mills Cheerios Yog	Lo	Ready-to-Eat Cereals
57601100	Wheat bran, unprocessed	Lo	Other
57603100	Rice polishings	Lo	Other
57604100	Whole wheat, cracked	Lo	Other
57801000	Barley cereal, baby food, dry, instar	Lo	Baby Foods
57803000	Mixed cereal, baby food, dry, instan	Lo	Baby Foods
57804000	Oatmeal cereal, baby food, dry, inst	Lo	Baby Foods
57805000	Rice cereal, baby food, dry, instant	Lo	Baby Foods
57805080	Rice cereal with apples, baby food,	Lo	Baby Foods
57805090	Rice cereal with mixed fruits, baby f	Lo	Baby Foods
57805100	Rice cereal with bananas, baby foo	Lo	Baby Foods
57805500	Brown rice cereal, baby food, dry, ir	Lo	Baby Foods
57806000	Mixed cereal with bananas, baby fo	Lo	Baby Foods
57806050	Multigrain, whole grain cereal, baby	Lo	Baby Foods
57806100	Oatmeal cereal with bananas, baby	Lo	Baby Foods
57806200	Oatmeal cereal with fruit, baby food	Lo	Baby Foods
57807010	Whole wheat cereal with apples, ba	Lo	Baby Foods
57820000	Cereal, baby food, jarred, NFS	Lo	Baby Foods
57820100	Rice cereal, baby food, jarred, NFS	Lo	Baby Foods
57822000	Mixed cereal with applesauce and b	Lo	Baby Foods
57823000	Oatmeal with applesauce and bana	Lo	Baby Foods
57824000	Rice cereal with applesauce and ba	Lo	Baby Foods
57824500	Rice cereal with mixed fruit, baby fo	Lo	Baby Foods
57830100	Gerber Graduates Finger Snacks C	Lo	Baby Foods
58100000	Burrito, taco, or quesadilla with egg	Lo	Mixed Dishes - Sandwiches (single code)
58100005	Burrito, taco, or quesadilla with egg	Lo	Mixed Dishes - Sandwiches (single code)
58100010	Burrito, taco, or quesadilla with egg	Lo	Mixed Dishes - Sandwiches (single code)
58100013	Burrito, taco, or quesadilla with egg	Lo	Mixed Dishes - Sandwiches (single code)
58100015	Burrito, taco, or quesadilla with egg	Lo	Mixed Dishes - Sandwiches (single code)
58100017	Burrito, taco, or quesadilla with egg	Lo	Mixed Dishes - Sandwiches (single code)
58100020	Burrito, taco, or quesadilla with egg	Lo	Mixed Dishes - Sandwiches (single code)
58100100	Burrito with meat	Lo	Mixed Dishes - Mexican
58100110	Burrito with beef and beans	Lo	Mixed Dishes - Mexican
58100120	Burrito with meat and beans	Lo	Mixed Dishes - Mexican
58100125	Burrito with meat and beans, from f	Lo	Mixed Dishes - Mexican
58100130	Burrito with beef and cheese, no be	Lo	Mixed Dishes - Mexican
58100135	Burrito with meat and sour cream	Lo	Mixed Dishes - Mexican
58100140	Burrito with meat, beans, and sour	Lo	Mixed Dishes - Mexican
58100145	Burrito with meat, beans, and sour	Lo	Mixed Dishes - Mexican
58100150	Burrito with beef and potato, no bea	Lo	Mixed Dishes - Mexican
58100155	Burrito with beef, rice, and cheese	Lo	Mixed Dishes - Mexican
58100160	Burrito with meat, beans, and rice	Lo	Mixed Dishes - Mexican
58100165	Burrito with meat, beans, rice, and s	Lo	Mixed Dishes - Mexican
58100180	Burrito with pork and beans	Lo	Mixed Dishes - Mexican
58100200	Burrito with chicken	Lo	Mixed Dishes - Mexican
58100210	Burrito with chicken and beans	Lo	Mixed Dishes - Mexican
58100220	Burrito with chicken and beans	Lo	Mixed Dishes - Mexican
58100230	Burrito with chicken and cheese	Lo	Mixed Dishes - Mexican
58100235	Burrito with chicken and sour crean	Lo	Mixed Dishes - Mexican
58100240	Burrito with chicken, NFS	Lo	Mixed Dishes - Mexican

58100245	Burrito with chicken, beans, and so	Lo	Mixed Dishes - Mexican
58100250	Burrito with chicken, rice, and chee	Lo	Mixed Dishes - Mexican
58100255	Burrito with chicken, beans, and ric	Lo	Mixed Dishes - Mexican
58100260	Burrito with chicken, beans, rice, ar	Lo	Mixed Dishes - Mexican
58100300	Burrito with beans and rice, meatle	Lo	Mixed Dishes - Mexican
58100310	Burrito with beans, meatless	Lo	Mixed Dishes - Mexican
58100320	Burrito with beans, meatless	Lo	Mixed Dishes - Mexican
58100325	Burrito with beans, meatless, from f	Lo	Mixed Dishes - Mexican
58100330	Burrito with beans, rice, and sour cr	Lo	Mixed Dishes - Mexican
58100340	Burrito with eggs, sausage, cheese	Lo	Mixed Dishes - Sandwiches (single code)
58100350	Burrito with eggs and cheese, no be	Lo	Mixed Dishes - Sandwiches (single code)
58100360	Chilaquiles, tortilla casserole with s	Lo	Mixed Dishes - Mexican
58100370	Chilaquiles, tortilla casserole with s	Lo	Mixed Dishes - Mexican
58100400	Enchilada with beef, no beans	Lo	Mixed Dishes - Mexican
58100410	Burrito with beef, cheese, and sour	Lo	Mixed Dishes - Mexican
58100500	Enchilada, no sauce	Lo	Mixed Dishes - Mexican
58100510	Enchilada with beef and beans	Lo	Mixed Dishes - Mexican
58100520	Enchilada with meat and beans, red	Lo	Mixed Dishes - Mexican
58100525	Enchilada with meat and beans, gre	Lo	Mixed Dishes - Mexican
58100530	Enchilada with meat, red-chile or er	Lo	Mixed Dishes - Mexican
58100535	Enchilada with meat, green-chile or	Lo	Mixed Dishes - Mexican
58100560	Enchilada with ham and cheese, no	Lo	Mixed Dishes - Mexican
58100600	Enchilada with chicken, tomato-bas	Lo	Mixed Dishes - Mexican
58100610	Enchilada with chicken and beans,	Lo	Mixed Dishes - Mexican
58100620	Enchilada with chicken and beans,	Lo	Mixed Dishes - Mexican
58100625	Enchilada with chicken and beans,	Lo	Mixed Dishes - Mexican
58100630	Enchilada with chicken, red-chile or	Lo	Mixed Dishes - Mexican
58100635	Enchilada with chicken, green-chile	Lo	Mixed Dishes - Mexican
58100710	Enchilada with beans, meatless	Lo	Mixed Dishes - Mexican
58100720	Enchilada with beans, meatless, red	Lo	Mixed Dishes - Mexican
58100725	Enchilada with beans, green-chile o	Lo	Mixed Dishes - Mexican
58100800	Enchilada, just cheese, meatless, n	Lo	Mixed Dishes - Mexican
58100805	Enchilada, just cheese, meatless, n	Lo	Mixed Dishes - Mexican
58100900	Enchilada with seafood, tomato-bas	Lo	Mixed Dishes - Mexican
58101200	Flauta, NFS	Lo	Mixed Dishes - Mexican
58101230	Flauta with beef	Lo	Mixed Dishes - Mexican
58101240	Flauta with chicken	Lo	Mixed Dishes - Mexican
58101300	Taco or tostada with beef, cheese a	Lo	Mixed Dishes - Mexican
58101310	Taco or tostada with beef, lettuce, t	Lo	Mixed Dishes - Mexican
58101320	Taco or tostada with meat	Lo	Mixed Dishes - Mexican
58101323	Taco or tostada with meat, from fas	Lo	Mixed Dishes - Mexican
58101325	Taco or tostada with meat and sour	Lo	Mixed Dishes - Mexican
58101345	Soft taco with meat	Lo	Mixed Dishes - Mexican
58101347	Soft taco with meat, from fast food	Lo	Mixed Dishes - Mexican
58101350	Soft taco with meat and sour cream	Lo	Mixed Dishes - Mexican
58101357	Soft taco with meat and sour cream	Lo	Mixed Dishes - Mexican
58101400	Soft taco with beef, cheese, and let	Lo	Mixed Dishes - Mexican
58101450	Soft taco with chicken	Lo	Mixed Dishes - Mexican
58101457	Soft taco with chicken, from fast foo	Lo	Mixed Dishes - Mexican
58101460	Soft taco with chicken and sour cre	Lo	Mixed Dishes - Mexican
58101510	Taco or tostada with chicken or turk	Lo	Mixed Dishes - Mexican

58101520	Taco or tostada with chicken	Lo	Mixed Dishes - Mexican
58101525	Taco or tostada with chicken and so	Lo	Mixed Dishes - Mexican
58101530	Soft taco with beef, cheese, lettuce,	Lo	Mixed Dishes - Mexican
58101540	Taco or tostada with fish	Lo	Mixed Dishes - Mexican
58101555	Soft taco with fish	Lo	Mixed Dishes - Mexican
58101600	Soft taco with bean, cheese, and le	Lo	Mixed Dishes - Mexican
58101610	Soft taco with beans	Lo	Mixed Dishes - Mexican
58101615	Soft taco with beans and sour crea	Lo	Mixed Dishes - Mexican
58101620	Soft taco with meat and beans	Lo	Mixed Dishes - Mexican
58101625	Soft taco with chicken and beans	Lo	Mixed Dishes - Mexican
58101630	Soft taco with meat, beans, and sou	Lo	Mixed Dishes - Mexican
58101635	Soft taco with chicken, beans, and s	Lo	Mixed Dishes - Mexican
58101710	Taco or tostada with beans, meatle	Lo	Mixed Dishes - Mexican
58101720	Taco or tostada with beans	Lo	Mixed Dishes - Mexican
58101725	Taco or tostada with beans and sou	Lo	Mixed Dishes - Mexican
58101730	Taco or tostada with meat and bea	Lo	Mixed Dishes - Mexican
58101733	Taco or tostada with meat and bea	Lo	Mixed Dishes - Mexican
58101735	Taco or tostada with chicken and b	Lo	Mixed Dishes - Mexican
58101740	Soft taco with egg and potato	Lo	Mixed Dishes - Sandwiches (single code)
58101745	Taco or tostada with meat, beans, a	Lo	Mixed Dishes - Mexican
58101750	Taco or tostada with chicken, beans	Lo	Mixed Dishes - Mexican
58101800	Ground beef with tomato sauce and	Lo	Mixed Dishes - Mexican
58101820	Mexican casserole made with grou	Lo	Mixed Dishes - Mexican
58101830	Mexican casserole made with grou	Lo	Mixed Dishes - Mexican
58101910	Taco or tostada salad with beef and	Lo	Mixed Dishes - Mexican
58101930	Taco or tostada salad with meat	Lo	Mixed Dishes - Mexican
58101935	Taco or tostada salad with chicken	Lo	Mixed Dishes - Mexican
58101940	Taco or tostada salad, meatless	Lo	Mixed Dishes - Mexican
58101945	Taco or tostada salad with meat an	Lo	Mixed Dishes - Mexican
58101950	Taco or tostada salad with chicken	Lo	Mixed Dishes - Mexican
58101955	Taco or tostada salad, meatless wit	Lo	Mixed Dishes - Mexican
58103110	Tamale with meat and/or poultry	Lo	Mixed Dishes - Mexican
58103120	Tamale with meat	Lo	Mixed Dishes - Mexican
58103130	Tamale with chicken	Lo	Mixed Dishes - Mexican
58103200	Tamale, plain, meatless, no sauce,	Lo	Mixed Dishes - Mexican
58103210	Tamale, meatless, with sauce, Pue	Lo	Mixed Dishes - Mexican
58103250	Tamale, plain, meatless, no sauce,	Lo	Mixed Dishes - Mexican
58103310	Tamale casserole with meat	Lo	Mixed Dishes - Mexican
58104080	Nachos with beef, beans, cheese, a	Lo	Mixed Dishes - Mexican
58104090	Nachos with cheese and sour crea	Lo	Mixed Dishes - Mexican
58104100	Nachos with cheese, meatless, no	Lo	Mixed Dishes - Mexican
58104110	Nachos with beans, no cheese	Lo	Mixed Dishes - Mexican
58104120	Nachos with cheese	Lo	Mixed Dishes - Mexican
58104130	Nachos with meat and cheese	Lo	Mixed Dishes - Mexican
58104140	Nachos with beef and cheese	Lo	Mixed Dishes - Mexican
58104150	Nachos with chicken and cheese	Lo	Mixed Dishes - Mexican
58104160	Nachos with chili	Lo	Mixed Dishes - Mexican
58104180	Nachos with meat, cheese, and sou	Lo	Mixed Dishes - Mexican
58104190	Nachos with chicken, cheese, and s	Lo	Mixed Dishes - Mexican
58104250	Nachos with chicken or turkey and	Lo	Mixed Dishes - Mexican
58104260	Gordita, sope, or chalupa with bear	Lo	Mixed Dishes - Mexican

58104270	Gordita, sope, or chalupa with bean	Lo	Mixed Dishes - Mexican
58104280	Gordita, sope, or chalupa with meat	Lo	Mixed Dishes - Mexican
58104290	Gordita, sope, or chalupa with meat	Lo	Mixed Dishes - Mexican
58104310	Chalupa with beans, chicken, chees	Lo	Mixed Dishes - Mexican
58104320	Gordita, sope, or chalupa with chick	Lo	Mixed Dishes - Mexican
58104340	Gordita, sope, or chalupa with chick	Lo	Mixed Dishes - Mexican
58104450	Chimichanga with beef and tomato	Lo	Mixed Dishes - Mexican
58104500	Chimichanga with meat	Lo	Mixed Dishes - Mexican
58104510	Chimichanga with beef, cheese, lett	Lo	Mixed Dishes - Mexican
58104520	Chimichanga, meatless	Lo	Mixed Dishes - Mexican
58104530	Chimichanga with chicken	Lo	Mixed Dishes - Mexican
58104535	Chimichanga with meat and sour cr	Lo	Mixed Dishes - Mexican
58104550	Chimichanga with chicken and sour	Lo	Mixed Dishes - Mexican
58104600	Chimichanga with beef and rice	Lo	Mixed Dishes - Mexican
58104710	Quesadilla, just cheese, meatless	Lo	Mixed Dishes - Mexican
58104720	Quesadilla, just cheese, from fast fo	Lo	Mixed Dishes - Mexican
58104730	Quesadilla with meat	Lo	Mixed Dishes - Mexican
58104740	Quesadilla with chicken	Lo	Mixed Dishes - Mexican
58104745	Quesadilla with chicken, from fast fo	Lo	Mixed Dishes - Mexican
58104750	Quesadilla with vegetables	Lo	Mixed Dishes - Mexican
58104760	Quesadilla with vegetables and mea	Lo	Mixed Dishes - Mexican
58104770	Quesadilla with vegetables and chic	Lo	Mixed Dishes - Mexican
58104800	Taquito or flauta with cheese	Lo	Mixed Dishes - Mexican
58104810	Taquitoes	Lo	Mixed Dishes - Mexican
58104820	Taquito or flauta with meat	Lo	Mixed Dishes - Mexican
58104825	Taquito or flauta with meat and che	Lo	Mixed Dishes - Mexican
58104830	Taquito or flauta with chicken	Lo	Mixed Dishes - Mexican
58104835	Taquito or flauta with chicken and c	Lo	Mixed Dishes - Mexican
58104900	Taquito or flauta with egg	Lo	Mixed Dishes - Sandwiches (single code)
58104905	Taquito or flauta with egg and break	Lo	Mixed Dishes - Sandwiches (single code)
58105000	Fajita with chicken and vegetables	Lo	Mixed Dishes - Mexican
58105050	Fajita with meat and vegetables	Lo	Mixed Dishes - Mexican
58105075	Fajita with vegetables	Lo	Mixed Dishes - Mexican
58105100	Pupusa, cheese-filled	Lo	Mixed Dishes - Mexican
58105105	Pupusa, bean-filled	Lo	Mixed Dishes - Mexican
58105110	Pupusa, meat-filled	Lo	Mixed Dishes - Mexican
58106200	Pizza, cheese, from frozen, thin cru	Lo	Mixed Dishes - Pizza
58106205	Pizza, cheese, from frozen, thick cr	Lo	Mixed Dishes - Pizza
58106210	Pizza, cheese, from restaurant or fa	Lo	Mixed Dishes - Pizza
58106220	Pizza, cheese, from restaurant or fa	Lo	Mixed Dishes - Pizza
58106225	Pizza, cheese, from restaurant or fa	Lo	Mixed Dishes - Pizza
58106230	Pizza, cheese, from restaurant or fa	Lo	Mixed Dishes - Pizza
58106233	Pizza, cheese, stuffed crust	Lo	Mixed Dishes - Pizza
58106234	Pizza, cheese, from school lunch, m	Lo	Mixed Dishes - Pizza
58106235	Pizza, cheese, from school lunch, th	Lo	Mixed Dishes - Pizza
58106236	Pizza, cheese, from school lunch, th	Lo	Mixed Dishes - Pizza
58106240	Pizza, extra cheese, NS as to type c	Lo	Mixed Dishes - Pizza
58106250	Pizza, extra cheese, thin crust	Lo	Mixed Dishes - Pizza
58106255	Pizza, extra cheese, regular crust	Lo	Mixed Dishes - Pizza
58106260	Pizza, extra cheese, thick crust	Lo	Mixed Dishes - Pizza
58106300	Pizza, cheese, with vegetables, from	Lo	Mixed Dishes - Pizza

58106305	Pizza, cheese with vegetables, from	Lo	Mixed Dishes - Pizza
58106310	Pizza, cheese, with vegetables, NS	Lo	Mixed Dishes - Pizza
58106320	Pizza, cheese, with vegetables, fron	Lo	Mixed Dishes - Pizza
58106325	Pizza, cheese, with vegetables, fron	Lo	Mixed Dishes - Pizza
58106330	Pizza, cheese, with vegetables, fron	Lo	Mixed Dishes - Pizza
58106345	Pizza with cheese and extra vegeta	Lo	Mixed Dishes - Pizza
58106347	Pizza with cheese and extra vegeta	Lo	Mixed Dishes - Pizza
58106350	Pizza with cheese and extra vegeta	Lo	Mixed Dishes - Pizza
58106358	Pizza, cheese, with fruit, thin crust	Lo	Mixed Dishes - Pizza
58106359	Pizza, cheese, with fruit, medium cr	Lo	Mixed Dishes - Pizza
58106360	Pizza, cheese, with fruit, thick crust	Lo	Mixed Dishes - Pizza
58106411	Pizza with chicken, thin crust	Lo	Mixed Dishes - Pizza
58106412	Pizza with chicken, regular crust	Lo	Mixed Dishes - Pizza
58106413	Pizza with chicken, thick crust	Lo	Mixed Dishes - Pizza
58106441	Pizza with chicken and vegetables,	Lo	Mixed Dishes - Pizza
58106442	Pizza with chicken and vegetables,	Lo	Mixed Dishes - Pizza
58106443	Pizza with chicken and vegetables,	Lo	Mixed Dishes - Pizza
58106462	Pizza with chicken and fruit, regular	Lo	Mixed Dishes - Pizza
58106500	Pizza with meat, prepared from froz	Lo	Mixed Dishes - Pizza
58106505	Pizza with meat, prepared from froz	Lo	Mixed Dishes - Pizza
58106510	Pizza with meat, NS as to type of cr	Lo	Mixed Dishes - Pizza
58106512	Pizza with pepperoni, from frozen, t	Lo	Mixed Dishes - Pizza
58106514	Pizza with pepperoni, from frozen, r	Lo	Mixed Dishes - Pizza
58106516	Pizza with pepperoni, from frozen, t	Lo	Mixed Dishes - Pizza
58106520	Pizza with meat, thin crust	Lo	Mixed Dishes - Pizza
58106530	Pizza with meat, thick crust	Lo	Mixed Dishes - Pizza
58106540	Pizza with pepperoni, from restaura	Lo	Mixed Dishes - Pizza
58106550	Pizza with pepperoni, from restaura	Lo	Mixed Dishes - Pizza
58106555	Pizza with pepperoni, from restaura	Lo	Mixed Dishes - Pizza
58106560	Pizza with pepperoni, from restaura	Lo	Mixed Dishes - Pizza
58106565	Pizza with pepperoni, stuffed crust	Lo	Mixed Dishes - Pizza
58106570	Pizza with pepperoni, from school lu	Lo	Mixed Dishes - Pizza
58106578	Pizza, with pepperoni, from school l	Lo	Mixed Dishes - Pizza
58106580	Pizza with pepperoni, from school lu	Lo	Mixed Dishes - Pizza
58106602	Pizza with meat other than peppero	Lo	Mixed Dishes - Pizza
58106604	Pizza with meat other than peppero	Lo	Mixed Dishes - Pizza
58106606	Pizza with meat other than peppero	Lo	Mixed Dishes - Pizza
58106610	Pizza with meat other than peppero	Lo	Mixed Dishes - Pizza
58106620	Pizza with meat other than peppero	Lo	Mixed Dishes - Pizza
58106625	Pizza with meat other than peppero	Lo	Mixed Dishes - Pizza
58106630	Pizza with meat other than peppero	Lo	Mixed Dishes - Pizza
58106633	Pizza, with meat other than pepperc	Lo	Mixed Dishes - Pizza
58106634	Pizza, with meat other than pepperc	Lo	Mixed Dishes - Pizza
58106635	Pizza, with meat other than pepperc	Lo	Mixed Dishes - Pizza
58106636	Pizza, with meat other than pepperc	Lo	Mixed Dishes - Pizza
58106640	Pizza with extra meat, NS as to type	Lo	Mixed Dishes - Pizza
58106650	Pizza with extra meat, thin crust	Lo	Mixed Dishes - Pizza
58106655	Pizza with extra meat, medium crus	Lo	Mixed Dishes - Pizza
58106660	Pizza with extra meat, thick crust	Lo	Mixed Dishes - Pizza
58106700	Pizza with meat and vegetables, fro	Lo	Mixed Dishes - Pizza
58106702	Pizza with meat and vegetables, fro	Lo	Mixed Dishes - Pizza

58106705	Pizza with meat and vegetables, fro	Lo	Mixed Dishes - Pizza
58106710	Pizza with meat and vegetables, NS	Lo	Mixed Dishes - Pizza
58106720	Pizza with meat and vegetables, fro	Lo	Mixed Dishes - Pizza
58106725	Pizza with meat and vegetables, fro	Lo	Mixed Dishes - Pizza
58106730	Pizza with meat and vegetables, fro	Lo	Mixed Dishes - Pizza
58106733	Pizza with extra meat and extra veg	Lo	Mixed Dishes - Pizza
58106734	Pizza with extra meat and extra veg	Lo	Mixed Dishes - Pizza
58106735	Pizza with extra meat and extra veg	Lo	Mixed Dishes - Pizza
58106736	Pizza with extra meat and extra veg	Lo	Mixed Dishes - Pizza
58106737	Pizza with extra meat and extra veg	Lo	Mixed Dishes - Pizza
58106738	Pizza with extra meat and extra veg	Lo	Mixed Dishes - Pizza
58106740	Pizza with meat and fruit, NS as to t	Lo	Mixed Dishes - Pizza
58106750	Pizza with meat and fruit, thin crust	Lo	Mixed Dishes - Pizza
58106755	Pizza with meat and fruit, medium c	Lo	Mixed Dishes - Pizza
58106760	Pizza with meat and fruit, thick crus	Lo	Mixed Dishes - Pizza
58106780	Pizza with meat and vegetables, pre	Lo	Mixed Dishes - Pizza
58106820	Pizza with beans and vegetables, th	Lo	Mixed Dishes - Pizza
58106830	Pizza with beans and vegetables, th	Lo	Mixed Dishes - Pizza
58106910	Pizza with seafood, thin crust	Lo	Mixed Dishes - Pizza
58106915	Pizza with seafood, regular crust	Lo	Mixed Dishes - Pizza
58106920	Pizza with seafood, thick crust	Lo	Mixed Dishes - Pizza
58107030	Pizza, no cheese, NS as to type of c	Lo	Mixed Dishes - Pizza
58107050	Pizza, no cheese, thin crust	Lo	Mixed Dishes - Pizza
58107060	Pizza, no cheese, regular crust	Lo	Mixed Dishes - Pizza
58107100	Pizza, no cheese, thick crust	Lo	Mixed Dishes - Pizza
58107205	White pizza, cheese, thin crust	Lo	Mixed Dishes - Pizza
58107210	White pizza, NS as to type of crust	Lo	Mixed Dishes - Pizza
58107212	White pizza, cheese, with vegetable	Lo	Mixed Dishes - Pizza
58107220	White pizza, thin crust	Lo	Mixed Dishes - Pizza
58107222	White pizza, cheese, with meat, thin	Lo	Mixed Dishes - Pizza
58107224	White pizza, cheese, with meat, thic	Lo	Mixed Dishes - Pizza
58107225	White pizza, regular crust	Lo	Mixed Dishes - Pizza
58107230	White pizza, thick crust	Lo	Mixed Dishes - Pizza
58107232	White pizza, cheese, with meat and	Lo	Mixed Dishes - Pizza
58108000	Calzone, with cheese, meatless	Lo	Mixed Dishes - Pizza
58108010	Calzone, with meat and cheese	Lo	Mixed Dishes - Pizza
58108050	Pizza rolls	Lo	Mixed Dishes - Pizza
58109000	Italian pie, meatless	Lo	Mixed Dishes - Pizza
58109015	Pizza, cheese, whole wheat thin cru	Lo	Mixed Dishes - Pizza
58109020	Pizza, cheese, whole wheat thick cr	Lo	Mixed Dishes - Pizza
58109030	Pizza, with meat, whole wheat thin c	Lo	Mixed Dishes - Pizza
58109040	Pizza, with meat, whole wheat thick	Lo	Mixed Dishes - Pizza
58109050	Pizza, cheese and vegetables, who	Lo	Mixed Dishes - Pizza
58109060	Pizza, cheese and vegetables, who	Lo	Mixed Dishes - Pizza
58109100	Pizza, cheese, gluten-free thin crus	Lo	Mixed Dishes - Pizza
58109120	Pizza, with meat, gluten-free thin cr	Lo	Mixed Dishes - Pizza
58109130	Pizza, with meat, gluten-free thick c	Lo	Mixed Dishes - Pizza
58109140	Pizza, cheese and vegetables, glute	Lo	Mixed Dishes - Pizza
58109150	Pizza, cheese and vegetables, glute	Lo	Mixed Dishes - Pizza
58109210	Breakfast pizza with egg	Lo	Mixed Dishes - Sandwiches (single code)
58110110	Egg roll, meatless	Lo	Mixed Dishes - Asian

58110120	Egg roll, with shrimp	Lo	Mixed Dishes - Asian
58110130	Egg roll, with beef and/or pork	Lo	Mixed Dishes - Asian
58110170	Egg roll, with chicken or turkey	Lo	Mixed Dishes - Asian
58110200	Roll with meat and/or shrimp, veget	Lo	Mixed Dishes - Asian
58111110	Wonton, fried, filled with meat, poul	Lo	Mixed Dishes - Asian
58111120	Wonton, fried, meatless	Lo	Mixed Dishes - Asian
58111130	Wonton, fried, filled with meat, poul	Lo	Mixed Dishes - Asian
58111200	Puffs, fried, crab meat and cream c	Lo	Mixed Dishes - Asian
58112110	Dim sum, meat filled (egg roll-type)	Lo	Mixed Dishes - Asian
58112510	Dumpling, steamed, filled with meat	Lo	Mixed Dishes - Asian
58113110	Dumpling, fried, pork	Lo	Mixed Dishes - Asian
58115110	Tamale casserole, Puerto Rican sty	Lo	Mixed Dishes - Mexican
58115210	Taco with crab meat, Puerto Rican	Lo	Mixed Dishes - Mexican
58116110	Meat turnover, Puerto Rican style	Lo	Mixed Dishes - Grain-based
58116115	Empanada, Mexican turnover, filled	Lo	Mixed Dishes - Grain-based
58116120	Empanada, Mexican turnover, filled	Lo	Mixed Dishes - Grain-based
58116130	Empanada, Mexican turnover, filled	Lo	Mixed Dishes - Grain-based
58116210	Meat pie, Puerto Rican style	Lo	Mixed Dishes - Grain-based
58116310	Cheese turnover, Puerto Rican style	Lo	Mixed Dishes - Grain-based
58117110	Cornmeal fritter, Puerto Rican style	Lo	Quick Breads and Bread Products
58117310	Kibby, Puerto Rican style	Lo	Mixed Dishes - Meat, Poultry, Seafood
58117410	Codfish fritter, Puerto Rican style	Lo	Mixed Dishes - Meat, Poultry, Seafood
58117510	Hayacas, Puerto Rican style	Lo	Mixed Dishes - Meat, Poultry, Seafood
58118210	Cornmeal coconut dessert, Puerto I	Lo	Other Desserts
58120110	Crepe, filled with meat, poultry, or s	Lo	Mixed Dishes - Grain-based
58120120	Crepe, filled with meat, poultry, or s	Lo	Mixed Dishes - Grain-based
58121510	Dumpling, meat-filled	Lo	Mixed Dishes - Grain-based
58121610	Dumpling, potato- or cheese-filled	Lo	Mixed Dishes - Grain-based
58121620	Dumpling, vegetable	Lo	Mixed Dishes - Grain-based
58122210	Gnocchi, cheese	Lo	Mixed Dishes - Grain-based
58122220	Gnocchi, potato	Lo	Mixed Dishes - Grain-based
58122310	Knish, potato	Lo	Mixed Dishes - Grain-based
58122320	Knish, cheese	Lo	Mixed Dishes - Grain-based
58122330	Knish, meat	Lo	Mixed Dishes - Grain-based
58123110	Sweet bread dough, filled with meat	Lo	Mixed Dishes - Asian
58123120	Sweet bread dough, filled with bean	Lo	Sweet Bakery Products
58124210	Pastry, cheese-filled	Lo	Sweet Bakery Products
58124220	Pastry, egg and cheese filled	Lo	Mixed Dishes - Grain-based
58124230	Pastry, meat / poultry-filled	Lo	Mixed Dishes - Grain-based
58124250	Spanakopitta	Lo	Mixed Dishes - Grain-based
58124500	Pastry, filled with potatoes and peas	Lo	Mixed Dishes - Grain-based
58125110	Quiche with meat, poultry or fish	Lo	Mixed Dishes - Grain-based
58125120	Spinach quiche, meatless	Lo	Mixed Dishes - Grain-based
58125180	Cheese quiche, meatless	Lo	Mixed Dishes - Grain-based
58126000	Turnover filled with ground beef and	Lo	Mixed Dishes - Grain-based
58126110	Turnover, meat-filled, no gravy	Lo	Mixed Dishes - Grain-based
58126130	Turnover, meat- and cheese-filled,	Lo	Mixed Dishes - Grain-based
58126140	Turnover, meat- and bean-filled, no	Lo	Mixed Dishes - Grain-based
58126150	Turnover, meat- and cheese-filled,	Lo	Mixed Dishes - Grain-based
58126160	Turnover, cheese-filled, tomato-bas	Lo	Mixed Dishes - Grain-based
58126170	Turnover filled with meat and veget	Lo	Mixed Dishes - Grain-based

58126180	Turnover, meat-, potato-, and veget	Lo	Mixed Dishes - Grain-based
58126270	Turnover, chicken- or turkey-, and c	Lo	Mixed Dishes - Grain-based
58126280	Turnover, chicken- or turkey-, and v	Lo	Mixed Dishes - Grain-based
58126290	Turnover, meat- and cheese-filled,	Lo	Mixed Dishes - Grain-based
58126300	Turnover, meat- and cheese-filled,	Lo	Mixed Dishes - Grain-based
58126310	Turnover, chicken, with gravy	Lo	Mixed Dishes - Grain-based
58126400	Turnover, filled with egg, meat and	Lo	Mixed Dishes - Grain-based
58126410	Turnover, filled with egg, meat, and	Lo	Mixed Dishes - Grain-based
58127110	Vegetables in pastry	Lo	Mixed Dishes - Grain-based
58127150	Vegetables and cheese in pastry	Lo	Mixed Dishes - Grain-based
58127210	Croissant sandwich, filled with ham	Lo	Mixed Dishes - Sandwiches (single code)
58127270	Croissant sandwich with sausage a	Lo	Mixed Dishes - Sandwiches (single code)
58127290	Croissant sandwich with bacon and	Lo	Mixed Dishes - Sandwiches (single code)
58127310	Croissant sandwich with ham, egg,	Lo	Mixed Dishes - Sandwiches (single code)
58127330	Croissant sandwich with sausage, e	Lo	Mixed Dishes - Sandwiches (single code)
58127350	Croissant sandwich with bacon, egg	Lo	Mixed Dishes - Sandwiches (single code)
58127500	Vegetable submarine sandwich, wit	Lo	Mixed Dishes - Sandwiches (single code)
58128000	Biscuit with gravy	Lo	Mixed Dishes - Sandwiches (single code)
58128120	Cornmeal dressing with chicken or	Lo	Mixed Dishes - Grain-based
58128210	Dressing with oysters	Lo	Mixed Dishes - Grain-based
58128220	Dressing with chicken or turkey and	Lo	Mixed Dishes - Grain-based
58128250	Dressing with meat and vegetables	Lo	Mixed Dishes - Grain-based
58130010	Lasagna with meat and/or poultry	Lo	Mixed Dishes - Grain-based
58130011	Lasagna with meat	Lo	Mixed Dishes - Grain-based
58130013	Lasagna with meat, canned	Lo	Mixed Dishes - Grain-based
58130014	Lasagna with meat, from restaurant	Lo	Mixed Dishes - Grain-based
58130015	Lasagna with meat, home recipe	Lo	Mixed Dishes - Grain-based
58130016	Lasagna with meat, frozen	Lo	Mixed Dishes - Grain-based
58130020	Lasagna with meat and spinach	Lo	Mixed Dishes - Grain-based
58130140	Lasagna with chicken or turkey	Lo	Mixed Dishes - Grain-based
58130150	Lasagna, with chicken or turkey, an	Lo	Mixed Dishes - Grain-based
58130310	Lasagna, meatless	Lo	Mixed Dishes - Grain-based
58130320	Lasagna, meatless, with vegetables	Lo	Mixed Dishes - Grain-based
58131100	Ravioli, NS as to filling, no sauce	Lo	Mixed Dishes - Grain-based
58131110	Ravioli, NS as to filling, with tomato	Lo	Mixed Dishes - Grain-based
58131120	Ravioli, NS as to filling, with cream	Lo	Mixed Dishes - Grain-based
58131310	Ravioli, meat-filled, no sauce	Lo	Mixed Dishes - Grain-based
58131320	Ravioli, meat-filled, with tomato sau	Lo	Mixed Dishes - Grain-based
58131323	Ravioli, meat-filled, with tomato sau	Lo	Mixed Dishes - Grain-based
58131330	Ravioli, meat-filled, with cream sauc	Lo	Mixed Dishes - Grain-based
58131510	Ravioli, cheese-filled, no sauce	Lo	Mixed Dishes - Grain-based
58131520	Ravioli, cheese-filled, with tomato s	Lo	Mixed Dishes - Grain-based
58131523	Ravioli, cheese-filled, with tomato s	Lo	Mixed Dishes - Grain-based
58131530	Ravioli, cheese-filled, with meat sau	Lo	Mixed Dishes - Grain-based
58131535	Ravioli, cheese-filled, with cream sa	Lo	Mixed Dishes - Grain-based
58131590	Ravioli, cheese and spinach-filled, r	Lo	Mixed Dishes - Grain-based
58131600	Ravioli, cheese and spinach-filled, v	Lo	Mixed Dishes - Grain-based
58131610	Ravioli, cheese and spinach filled, v	Lo	Mixed Dishes - Grain-based
58132110	Spaghetti with tomato sauce, meatl	Lo	Mixed Dishes - Grain-based
58132113	Pasta with tomato sauce and chees	Lo	Mixed Dishes - Grain-based
58132310	Spaghetti with tomato sauce and m	Lo	Mixed Dishes - Grain-based

58132313	Pasta with tomato sauce and meat	Lo	Mixed Dishes - Grain-based
58132340	Spaghetti with tomato sauce and ve	Lo	Mixed Dishes - Grain-based
58132350	Spaghetti with tomato sauce, meatl	Lo	Mixed Dishes - Grain-based
58132360	Spaghetti with tomato sauce and m	Lo	Mixed Dishes - Grain-based
58132450	Spaghetti with tomato sauce, meatl	Lo	Mixed Dishes - Grain-based
58132460	Spaghetti with tomato sauce and m	Lo	Mixed Dishes - Grain-based
58132710	Spaghetti with tomato sauce and fra	Lo	Mixed Dishes - Grain-based
58132713	Pasta with tomato sauce and frankf	Lo	Mixed Dishes - Grain-based
58132800	Spaghetti with clam sauce, NS as to	Lo	Mixed Dishes - Grain-based
58132810	Spaghetti with red clam sauce	Lo	Mixed Dishes - Grain-based
58132820	Spaghetti with white clam sauce	Lo	Mixed Dishes - Grain-based
58132910	Spaghetti with tomato sauce and po	Lo	Mixed Dishes - Grain-based
58133110	Manicotti, cheese-filled, no sauce	Lo	Mixed Dishes - Grain-based
58133120	Manicotti, cheese-filled, with tomato	Lo	Mixed Dishes - Grain-based
58133130	Manicotti, cheese-filled, with meat s	Lo	Mixed Dishes - Grain-based
58133140	Manicotti, vegetable- and cheese-fil	Lo	Mixed Dishes - Grain-based
58134110	Stuffed shells, cheese-filled, no sau	Lo	Mixed Dishes - Grain-based
58134120	Stuffed shells, cheese-filled, with to	Lo	Mixed Dishes - Grain-based
58134130	Stuffed shells, cheese-filled, with m	Lo	Mixed Dishes - Grain-based
58134160	Stuffed shells, cheese- and spinach	Lo	Mixed Dishes - Grain-based
58134210	Stuffed shells, with chicken, with tor	Lo	Mixed Dishes - Grain-based
58134310	Stuffed shells, with fish and/or shell	Lo	Mixed Dishes - Grain-based
58134610	Tortellini, meat-filled, with tomato sa	Lo	Mixed Dishes - Grain-based
58134613	Tortellini, meat-filled, with tomato sa	Lo	Mixed Dishes - Grain-based
58134620	Tortellini, cheese-filled, meatless, w	Lo	Mixed Dishes - Grain-based
58134623	Tortellini, cheese-filled, meatless, w	Lo	Mixed Dishes - Grain-based
58134640	Tortellini, cheese-filled, meatless, w	Lo	Mixed Dishes - Grain-based
58134650	Tortellini, meat-filled, no sauce	Lo	Mixed Dishes - Grain-based
58134660	Tortellini, cheese-filled, with cream	Lo	Mixed Dishes - Grain-based
58134680	Tortellini, cheese-filled, no sauce	Lo	Mixed Dishes - Grain-based
58134710	Tortellini, spinach-filled, with tomato	Lo	Mixed Dishes - Grain-based
58134720	Tortellini, spinach-filled, no sauce	Lo	Mixed Dishes - Grain-based
58134810	Cannelloni, cheese- and spinach-fil	Lo	Mixed Dishes - Grain-based
58135110	Chow fun noodles with meat and ve	Lo	Mixed Dishes - Asian
58135120	Chow fun noodles with vegetables,	Lo	Mixed Dishes - Asian
58136110	Lo mein, NFS	Lo	Mixed Dishes - Asian
58136120	Lo mein, meatless	Lo	Mixed Dishes - Asian
58136130	Lo mein, with shrimp	Lo	Mixed Dishes - Asian
58136140	Lo mein, with pork	Lo	Mixed Dishes - Asian
58136150	Lo mein, with beef	Lo	Mixed Dishes - Asian
58136160	Lo mein, with chicken	Lo	Mixed Dishes - Asian
58137210	Pad Thai, NFS	Lo	Mixed Dishes - Asian
58137220	Pad Thai, meatless	Lo	Mixed Dishes - Asian
58137230	Pad Thai with chicken	Lo	Mixed Dishes - Asian
58137240	Pad Thai with seafood	Lo	Mixed Dishes - Asian
58137250	Pad Thai with meat	Lo	Mixed Dishes - Asian
58137300	Adobo, with noodles	Lo	Mixed Dishes - Meat, Poultry, Seafood
58145110	Macaroni or noodles with cheese	Lo	Mixed Dishes - Grain-based
58145111	Macaroni or noodles with cheese, fr	Lo	Mixed Dishes - Grain-based
58145112	Macaroni or noodles with cheese, n	Lo	Mixed Dishes - Grain-based
58145113	Macaroni or noodles with cheese, c	Lo	Mixed Dishes - Grain-based

58145114	Macaroni or noodles with cheese, n	Lo	Mixed Dishes - Grain-based
58145115	Macaroni or noodles with cheese, fr	Lo	Mixed Dishes - Grain-based
58145117	Macaroni or noodles with cheese, E	Lo	Mixed Dishes - Grain-based
58145119	Macaroni or noodles with cheese, n	Lo	Mixed Dishes - Grain-based
58145120	Macaroni or noodles with cheese ar	Lo	Mixed Dishes - Grain-based
58145130	Macaroni or noodles with cheese ar	Lo	Mixed Dishes - Grain-based
58145135	Macaroni or noodles with cheese ar	Lo	Mixed Dishes - Grain-based
58145136	Macaroni or noodles with cheese ar	Lo	Mixed Dishes - Grain-based
58145140	Macaroni or noodles with cheese ar	Lo	Mixed Dishes - Grain-based
58145150	Macaroni or noodles with cheese ar	Lo	Mixed Dishes - Grain-based
58145160	Macaroni or noodles with cheese ar	Lo	Mixed Dishes - Grain-based
58145170	Macaroni or noodles with cheese ar	Lo	Mixed Dishes - Grain-based
58145190	Macaroni or noodles with cheese ar	Lo	Mixed Dishes - Grain-based
58145300	Macaroni or noodles with cheese, w	Lo	Mixed Dishes - Grain-based
58146100	Pasta with tomato sauce, meatless	Lo	Mixed Dishes - Grain-based
58146110	Pasta with meat sauce	Lo	Mixed Dishes - Grain-based
58146120	Pasta with tomato-based sauce, ch	Lo	Mixed Dishes - Grain-based
58146130	Pasta with carbonara sauce	Lo	Mixed Dishes - Grain-based
58146150	Pasta with tomato-based sauce and	Lo	Mixed Dishes - Grain-based
58146160	Pasta with vegetables, no sauce or	Lo	Mixed Dishes - Grain-based
58146200	Pasta, meat-filled, with gravy, cann	Lo	Mixed Dishes - Grain-based
58146210	Pasta with sauce, NFS	Lo	Mixed Dishes - Grain-based
58146215	Pasta with sauce, meatless, school	Lo	Mixed Dishes - Grain-based
58146221	Pasta with tomato-based sauce, res	Lo	Mixed Dishes - Grain-based
58146222	Pasta with tomato-based sauce, ho	Lo	Mixed Dishes - Grain-based
58146223	Pasta with tomato-based sauce, rea	Lo	Mixed Dishes - Grain-based
58146300	Pasta, whole wheat, with meat sauc	Lo	Mixed Dishes - Grain-based
58146301	Pasta with tomato-based sauce, an	Lo	Mixed Dishes - Grain-based
58146302	Pasta with tomato-based sauce, an	Lo	Mixed Dishes - Grain-based
58146303	Pasta with tomato-based sauce, an	Lo	Mixed Dishes - Grain-based
58146310	Pasta, whole wheat, with tomato sa	Lo	Mixed Dishes - Grain-based
58146315	Pasta with sauce and meat, from sc	Lo	Mixed Dishes - Grain-based
58146321	Pasta with tomato-based sauce and	Lo	Mixed Dishes - Grain-based
58146322	Pasta with tomato-based sauce and	Lo	Mixed Dishes - Grain-based
58146323	Pasta with tomato-based sauce and	Lo	Mixed Dishes - Grain-based
58146331	Pasta with tomato-based sauce, me	Lo	Mixed Dishes - Grain-based
58146332	Pasta with tomato-based sauce, me	Lo	Mixed Dishes - Grain-based
58146333	Pasta with tomato-based sauce, me	Lo	Mixed Dishes - Grain-based
58146341	Pasta with tomato-based sauce and	Lo	Mixed Dishes - Grain-based
58146342	Pasta with tomato-based sauce and	Lo	Mixed Dishes - Grain-based
58146343	Pasta with tomato-based sauce and	Lo	Mixed Dishes - Grain-based
58146351	Pasta with tomato-based sauce, po	Lo	Mixed Dishes - Grain-based
58146352	Pasta with tomato-based sauce, po	Lo	Mixed Dishes - Grain-based
58146353	Pasta with tomato-based sauce, po	Lo	Mixed Dishes - Grain-based
58146361	Pasta with tomato-based sauce and	Lo	Mixed Dishes - Grain-based
58146362	Pasta with tomato-based sauce and	Lo	Mixed Dishes - Grain-based
58146363	Pasta with tomato-based sauce and	Lo	Mixed Dishes - Grain-based
58146371	Pasta with tomato-based sauce, se	Lo	Mixed Dishes - Grain-based
58146372	Pasta with tomato-based sauce, se	Lo	Mixed Dishes - Grain-based
58146373	Pasta with tomato-based sauce, se	Lo	Mixed Dishes - Grain-based
58146381	Pasta with cream sauce, restaurant	Lo	Mixed Dishes - Grain-based

58146382	Pasta with cream sauce, home reci	Lo	Mixed Dishes - Grain-based
58146383	Pasta with cream sauce, ready-to-h	Lo	Mixed Dishes - Grain-based
58146391	Pasta with cream sauce and added	Lo	Mixed Dishes - Grain-based
58146392	Pasta with cream sauce and added	Lo	Mixed Dishes - Grain-based
58146393	Pasta with cream sauce and added	Lo	Mixed Dishes - Grain-based
58146401	Pasta with cream sauce and meat,	Lo	Mixed Dishes - Grain-based
58146402	Pasta with cream sauce and meat,	Lo	Mixed Dishes - Grain-based
58146403	Pasta with cream sauce and meat,	Lo	Mixed Dishes - Grain-based
58146411	Pasta with cream sauce, meat, and	Lo	Mixed Dishes - Grain-based
58146412	Pasta with cream sauce, meat, and	Lo	Mixed Dishes - Grain-based
58146413	Pasta with cream sauce, meat, and	Lo	Mixed Dishes - Grain-based
58146421	Pasta with cream sauce and poultry	Lo	Mixed Dishes - Grain-based
58146422	Pasta with cream sauce and poultry	Lo	Mixed Dishes - Grain-based
58146423	Pasta with cream sauce and poultry	Lo	Mixed Dishes - Grain-based
58146431	Pasta with cream sauce, poultry, ar	Lo	Mixed Dishes - Grain-based
58146432	Pasta with cream sauce, poultry, ar	Lo	Mixed Dishes - Grain-based
58146433	Pasta with cream sauce, poultry, ar	Lo	Mixed Dishes - Grain-based
58146441	Pasta with cream sauce and seafoo	Lo	Mixed Dishes - Grain-based
58146442	Pasta with cream sauce and seafoo	Lo	Mixed Dishes - Grain-based
58146443	Pasta with cream sauce and seafoo	Lo	Mixed Dishes - Grain-based
58146451	Pasta with cream sauce, seafood, a	Lo	Mixed Dishes - Grain-based
58146452	Pasta with cream sauce, seafood, a	Lo	Mixed Dishes - Grain-based
58146601	Pasta, whole grain, with tomato-bas	Lo	Mixed Dishes - Grain-based
58146602	Pasta, whole grain, with tomato-bas	Lo	Mixed Dishes - Grain-based
58146603	Pasta, whole grain, with tomato-bas	Lo	Mixed Dishes - Grain-based
58146612	Pasta, whole grain, with tomato-bas	Lo	Mixed Dishes - Grain-based
58146613	Pasta, whole grain, with tomato-bas	Lo	Mixed Dishes - Grain-based
58146622	Pasta, whole grain, with tomato-bas	Lo	Mixed Dishes - Grain-based
58146623	Pasta, whole grain, with tomato-bas	Lo	Mixed Dishes - Grain-based
58146632	Pasta, whole grain, with tomato-bas	Lo	Mixed Dishes - Grain-based
58146641	Pasta, whole grain, with tomato-bas	Lo	Mixed Dishes - Grain-based
58146642	Pasta, whole grain, with tomato-bas	Lo	Mixed Dishes - Grain-based
58146652	Pasta, whole grain, with tomato-bas	Lo	Mixed Dishes - Grain-based
58146653	Pasta, whole grain, with tomato-bas	Lo	Mixed Dishes - Grain-based
58146662	Pasta, whole grain, with tomato-bas	Lo	Mixed Dishes - Grain-based
58146672	Pasta, whole grain, with tomato-bas	Lo	Mixed Dishes - Grain-based
58146682	Pasta, whole grain, with cream sau	Lo	Mixed Dishes - Grain-based
58146683	Pasta, whole grain, with cream sau	Lo	Mixed Dishes - Grain-based
58146692	Pasta, whole grain, with cream sau	Lo	Mixed Dishes - Grain-based
58146693	Pasta, whole grain, with cream sau	Lo	Mixed Dishes - Grain-based
58146702	Pasta, whole grain, with cream sau	Lo	Mixed Dishes - Grain-based
58146713	Pasta, whole grain, with cream sau	Lo	Mixed Dishes - Grain-based
58146722	Pasta, whole grain, with cream sau	Lo	Mixed Dishes - Grain-based
58146723	Pasta, whole grain, with cream sau	Lo	Mixed Dishes - Grain-based
58146732	Pasta, whole grain, with cream sau	Lo	Mixed Dishes - Grain-based
58146733	Pasta, whole grain, with cream sau	Lo	Mixed Dishes - Grain-based
58146741	Pasta, whole grain, with cream sau	Lo	Mixed Dishes - Grain-based
58147100	Pasta with pesto sauce	Lo	Mixed Dishes - Grain-based
58147110	Pasta with tomato-based sauce and	Lo	Mixed Dishes - Grain-based
58147310	Macaroni, creamed	Lo	Mixed Dishes - Grain-based
58147330	Macaroni or noodles, creamed, with	Lo	Mixed Dishes - Grain-based

58147340	Macaroni or noodles, creamed, with	Lo	Mixed Dishes - Grain-based
58147350	Macaroni, creamed, with vegetables	Lo	Mixed Dishes - Grain-based
58147510	Flavored pasta	Lo	Mixed Dishes - Grain-based
58148110	Macaroni or pasta salad, made with	Lo	Mixed Dishes - Grain-based
58148111	Macaroni or pasta salad, made with	Lo	Mixed Dishes - Grain-based
58148112	Macaroni or pasta salad, made with	Lo	Mixed Dishes - Grain-based
58148114	Macaroni or pasta salad, made with	Lo	Mixed Dishes - Grain-based
58148115	Macaroni or pasta salad, made with	Lo	Mixed Dishes - Grain-based
58148116	Macaroni or pasta salad, made with	Lo	Mixed Dishes - Grain-based
58148117	Macaroni or pasta salad, made with	Lo	Mixed Dishes - Grain-based
58148118	Macaroni or pasta salad, made with	Lo	Mixed Dishes - Grain-based
58148120	Macaroni or pasta salad with egg	Lo	Mixed Dishes - Grain-based
58148130	Macaroni or pasta salad with tuna	Lo	Mixed Dishes - Grain-based
58148140	Macaroni or pasta salad with crab n	Lo	Mixed Dishes - Grain-based
58148150	Macaroni or pasta salad with shrim	Lo	Mixed Dishes - Grain-based
58148160	Macaroni or pasta salad with tuna a	Lo	Mixed Dishes - Grain-based
58148170	Macaroni or pasta salad with chicke	Lo	Mixed Dishes - Grain-based
58148550	Macaroni or pasta salad with meat	Lo	Mixed Dishes - Grain-based
58149110	Noodle pudding	Lo	Mixed Dishes - Grain-based
58149160	Noodle pudding, with milk	Lo	Other Desserts
58150110	Rice, fried, meatless	Lo	Mixed Dishes - Asian
58150310	Rice, fried, NFS	Lo	Mixed Dishes - Asian
58150320	Rice, fried, with chicken	Lo	Mixed Dishes - Asian
58150330	Rice, fried, with pork	Lo	Mixed Dishes - Asian
58150340	Rice, fried, with beef	Lo	Mixed Dishes - Asian
58150510	Rice, fried, with shrimp	Lo	Mixed Dishes - Asian
58150520	Dukboki or Tteokbokki, Korean	Lo	Mixed Dishes - Grain-based
58150530	Adobo, with rice	Lo	Mixed Dishes - Meat, Poultry, Seafood
58151100	Sushi, NFS	Lo	Mixed Dishes - Asian
58151110	Sushi, no vegetables, no seafood (r	Lo	Mixed Dishes - Asian
58151120	Sushi, with vegetables, no seafood	Lo	Mixed Dishes - Asian
58151130	Sushi, with vegetables and seafood	Lo	Mixed Dishes - Asian
58151140	Sushi, with vegetables, rolled in sea	Lo	Mixed Dishes - Asian
58151150	Sushi, with seafood, no vegetables	Lo	Mixed Dishes - Asian
58151170	Sushi roll, avocado	Lo	Mixed Dishes - Asian
58151180	Sushi roll, California	Lo	Mixed Dishes - Asian
58151190	Sushi roll, eel	Lo	Mixed Dishes - Asian
58151200	Sushi roll, salmon	Lo	Mixed Dishes - Asian
58151210	Sushi roll, shrimp	Lo	Mixed Dishes - Asian
58151220	Sushi roll tuna	Lo	Mixed Dishes - Asian
58151230	Sushi roll, vegetable	Lo	Mixed Dishes - Asian
58151400	Sushi, topped with crab	Lo	Mixed Dishes - Asian
58151410	Sushi, topped with eel	Lo	Mixed Dishes - Asian
58151420	Sushi, topped with salmon	Lo	Mixed Dishes - Asian
58151430	Sushi, topped with shrimp	Lo	Mixed Dishes - Asian
58151440	Sushi, topped with tuna	Lo	Mixed Dishes - Asian
58151450	Sushi, topped with egg	Lo	Mixed Dishes - Asian
58155110	Rice with chicken, Puerto Rican sty	Lo	Mixed Dishes - Grain-based
58155310	Paella with meat, Valenciana style	Lo	Mixed Dishes - Grain-based
58155320	Seafood paella, Puerto Rican style	Lo	Mixed Dishes - Grain-based
58155410	Soupy rice with chicken, Puerto Ric	Lo	Mixed Dishes - Soups

58155610	Rice meal fritter, Puerto Rican style	Lo	Mixed Dishes - Grain-based
58155810	Stewed rice, Puerto Rican style	Lo	Mixed Dishes - Grain-based
58156210	Rice with vienna sausage, Puerto R	Lo	Mixed Dishes - Grain-based
58156310	Rice with Spanish sausage, Puerto	Lo	Mixed Dishes - Grain-based
58156410	Rice with onions, Puerto Rican style	Lo	Mixed Dishes - Grain-based
58156610	Pigeon pea asopao, Asopao de gar	Lo	Mixed Dishes - Soups
58156710	Rice with stewed beans, Puerto Ric	Lo	Mixed Dishes - Grain-based
58157210	Rice pudding made with coconut m	Lo	Other Desserts
58157300	Congee, with meat, poultry, and/or	Lo	Mixed Dishes - Grain-based
58157310	Congee, with meat, poultry, and/or	Lo	Mixed Dishes - Grain-based
58157320	Congee, with vegetables	Lo	Mixed Dishes - Grain-based
58160000	Biryani with vegetables	Lo	Mixed Dishes - Grain-based
58160100	Beans and rice, from fast food / res	Lo	Mixed Dishes - Bean/Vegetable-based
58160102	Kidney beans and rice, from fast fo	Lo	Mixed Dishes - Bean/Vegetable-based
58160104	Black beans and rice, from fast foo	Lo	Mixed Dishes - Bean/Vegetable-based
58160106	Pinto beans and rice, from fast food	Lo	Mixed Dishes - Bean/Vegetable-based
58160110	Beans and white rice	Lo	Mixed Dishes - Bean/Vegetable-based
58160120	Beans and rice, with tomatoes	Lo	Mixed Dishes - Bean/Vegetable-based
58160130	Rice with beans and chicken	Lo	Mixed Dishes - Bean/Vegetable-based
58160132	Beans and rice, with meat	Lo	Mixed Dishes - Bean/Vegetable-based
58160135	Rice with beans and beef	Lo	Mixed Dishes - Bean/Vegetable-based
58160140	Rice with beans and pork	Lo	Mixed Dishes - Bean/Vegetable-based
58160150	Kidney beans and white rice	Lo	Mixed Dishes - Bean/Vegetable-based
58160154	Black beans and white rice	Lo	Mixed Dishes - Bean/Vegetable-based
58160156	Pinto beans and white rice	Lo	Mixed Dishes - Bean/Vegetable-based
58160160	Hopping John	Lo	Mixed Dishes - Grain-based
58160200	Rice with vegetables (including carr	Lo	Mixed Dishes - Grain-based
58160202	Rice with vegetables (including carr	Lo	Mixed Dishes - Grain-based
58160204	Rice with vegetables (including carr	Lo	Mixed Dishes - Grain-based
58160205	Rice with vegetables (excluding car	Lo	Mixed Dishes - Grain-based
58160207	Rice with vegetables (excluding car	Lo	Mixed Dishes - Grain-based
58160209	Rice with vegetables (excluding car	Lo	Mixed Dishes - Grain-based
58160210	Rice with vegetables, no sauce	Lo	Mixed Dishes - Grain-based
58160220	Rice with vegetables, tomato-based	Lo	Mixed Dishes - Grain-based
58160290	Rice with corn, NS as to fat added i	Lo	Mixed Dishes - Grain-based
58160292	Rice with corn, fat not added in coo	Lo	Mixed Dishes - Grain-based
58160294	Rice with corn, fat added in cooking	Lo	Mixed Dishes - Grain-based
58160300	Rice with peas, NS as to fat added	Lo	Mixed Dishes - Grain-based
58160302	Rice with peas, fat not added in coo	Lo	Mixed Dishes - Grain-based
58160304	Rice with peas, fat added in cooking	Lo	Mixed Dishes - Grain-based
58160310	Rice with peas and carrots, NS as t	Lo	Mixed Dishes - Grain-based
58160312	Rice with peas and carrots, fat not a	Lo	Mixed Dishes - Grain-based
58160314	Rice with peas and carrots, fat adde	Lo	Mixed Dishes - Grain-based
58160320	Rice with tomatoes, NS as to fat ad	Lo	Mixed Dishes - Grain-based
58160322	Rice with tomatoes, fat not added in	Lo	Mixed Dishes - Grain-based
58160324	Rice with tomatoes, fat added in co	Lo	Mixed Dishes - Grain-based
58160400	Rice, white, with corn, NS as to fat	Lo	Mixed Dishes - Grain-based
58160410	Rice, white, with corn, no added fat	Lo	Mixed Dishes - Grain-based
58160420	Rice, white, with corn, fat added	Lo	Mixed Dishes - Grain-based
58160430	Rice, white, with peas, NS as to fat	Lo	Mixed Dishes - Grain-based
58160440	Rice, white, with peas, no added fat	Lo	Mixed Dishes - Grain-based

58160450	Rice, white, with peas, fat added	Lo	Mixed Dishes - Grain-based
58160460	Rice, white, with carrots, NS as to fa	Lo	Mixed Dishes - Grain-based
58160470	Rice, white, with carrots, no added f	Lo	Mixed Dishes - Grain-based
58160480	Rice, white, with carrots, fat added	Lo	Mixed Dishes - Grain-based
58160490	Rice, white, with peas and carrots, N	Lo	Mixed Dishes - Grain-based
58160500	Rice, white, with peas and carrots, n	Lo	Mixed Dishes - Grain-based
58160510	Rice, white, with peas and carrots, f	Lo	Mixed Dishes - Grain-based
58160520	Rice, white, with tomatoes and/or to	Lo	Mixed Dishes - Grain-based
58160530	Rice, white, with tomatoes and/or to	Lo	Mixed Dishes - Grain-based
58160540	Rice, white, with tomatoes and/or to	Lo	Mixed Dishes - Grain-based
58160550	Rice, white, with dark green vegeta	Lo	Mixed Dishes - Grain-based
58160560	Rice, white, with dark green vegeta	Lo	Mixed Dishes - Grain-based
58160570	Rice, white, with dark green vegeta	Lo	Mixed Dishes - Grain-based
58160580	Rice, white, with carrots and tomato	Lo	Mixed Dishes - Grain-based
58160590	Rice, white, with carrots and tomato	Lo	Mixed Dishes - Grain-based
58160600	Rice, white, with carrots and tomato	Lo	Mixed Dishes - Grain-based
58160610	Rice, white, with dark green vegeta	Lo	Mixed Dishes - Grain-based
58160620	Rice, white, with dark green vegeta	Lo	Mixed Dishes - Grain-based
58160630	Rice, white, with dark green vegeta	Lo	Mixed Dishes - Grain-based
58160640	Rice, white, with carrots and dark g	Lo	Mixed Dishes - Grain-based
58160650	Rice, white, with carrots and dark g	Lo	Mixed Dishes - Grain-based
58160660	Rice, white, with carrots and dark g	Lo	Mixed Dishes - Grain-based
58160670	Rice, white, with carrots, dark greer	Lo	Mixed Dishes - Grain-based
58160690	Rice, white, with carrots, dark greer	Lo	Mixed Dishes - Grain-based
58160700	Rice, white, with other vegetables, N	Lo	Mixed Dishes - Grain-based
58160710	Rice, white, with other vegetables, n	Lo	Mixed Dishes - Grain-based
58160720	Rice, white, with other vegetables, f	Lo	Mixed Dishes - Grain-based
58160800	Rice, white, with lentils, NS as to fat	Lo	Mixed Dishes - Grain-based
58160805	Rice, white, with lentils, fat added	Lo	Mixed Dishes - Grain-based
58160810	Rice, white, with lentils, no added fa	Lo	Mixed Dishes - Grain-based
58161110	Rice casserole with cheese	Lo	Mixed Dishes - Grain-based
58161120	Brown rice casserole with cheese	Lo	Mixed Dishes - Grain-based
58161200	Rice, cooked with coconut milk	Lo	Mixed Dishes - Grain-based
58161300	White rice with tomato sauce	Lo	Mixed Dishes - Grain-based
58161310	Rice, brown, with tomato sauce	Lo	Mixed Dishes - Grain-based
58161320	Beans and brown rice	Lo	Mixed Dishes - Bean/Vegetable-based
58161321	Kidney beans and brown rice	Lo	Mixed Dishes - Bean/Vegetable-based
58161322	Black beans and brown rice	Lo	Mixed Dishes - Bean/Vegetable-based
58161323	Pinto beans and brown rice	Lo	Mixed Dishes - Bean/Vegetable-based
58161325	Rice, brown, with beans and tomato	Lo	Mixed Dishes - Grain-based
58161400	Rice, brown, with vegetables (incluc	Lo	Mixed Dishes - Grain-based
58161402	Rice, brown, with vegetables (incluc	Lo	Mixed Dishes - Grain-based
58161404	Rice, brown, with vegetables (incluc	Lo	Mixed Dishes - Grain-based
58161405	Rice, brown, with vegetables (exclu	Lo	Mixed Dishes - Grain-based
58161407	Rice, brown, with vegetables (exclu	Lo	Mixed Dishes - Grain-based
58161409	Rice, brown, with vegetables (exclu	Lo	Mixed Dishes - Grain-based
58161420	Rice, brown, with corn, NS as to fat	Lo	Mixed Dishes - Grain-based
58161422	Rice, brown, with corn, no added fa	Lo	Mixed Dishes - Grain-based
58161424	Rice, brown, with corn, fat added	Lo	Mixed Dishes - Grain-based
58161430	Rice, brown, with peas, NS as to fat	Lo	Mixed Dishes - Grain-based
58161432	Rice, brown, with peas, no added fa	Lo	Mixed Dishes - Grain-based

58161434	Rice, brown, with peas, fat added	Lo	Mixed Dishes - Grain-based
58161435	Rice, brown, with carrots, NS as to	Lo	Mixed Dishes - Grain-based
58161437	Rice, brown, with carrots, no added	Lo	Mixed Dishes - Grain-based
58161439	Rice, brown, with carrots, fat added	Lo	Mixed Dishes - Grain-based
58161440	Rice, brown, with peas and carrots,	Lo	Mixed Dishes - Grain-based
58161442	Rice, brown, with peas and carrots,	Lo	Mixed Dishes - Grain-based
58161444	Rice, brown, with peas and carrots,	Lo	Mixed Dishes - Grain-based
58161452	Rice, brown, with tomatoes, fat not	Lo	Mixed Dishes - Grain-based
58161454	Rice, brown, with tomatoes, fat add	Lo	Mixed Dishes - Grain-based
58161460	Rice, brown, with tomatoes and/or t	Lo	Mixed Dishes - Grain-based
58161462	Rice, brown, with tomatoes and/or t	Lo	Mixed Dishes - Grain-based
58161464	Rice, brown, with tomatoes and/or t	Lo	Mixed Dishes - Grain-based
58161470	Rice, brown, with dark green vegeta	Lo	Mixed Dishes - Grain-based
58161472	Rice, brown, with dark green vegeta	Lo	Mixed Dishes - Grain-based
58161474	Rice, brown, with dark green vegeta	Lo	Mixed Dishes - Grain-based
58161480	Rice, brown, with carrots and tomat	Lo	Mixed Dishes - Grain-based
58161490	Rice, brown, with dark green vegeta	Lo	Mixed Dishes - Grain-based
58161500	Rice, brown, with carrots and dark g	Lo	Mixed Dishes - Grain-based
58161502	Rice, brown, with carrots and dark g	Lo	Mixed Dishes - Grain-based
58161504	Rice, brown, with carrots and dark g	Lo	Mixed Dishes - Grain-based
58161510	Grape leaves stuffed with rice	Lo	Mixed Dishes - Grain-based
58161524	Rice, brown, with carrots, dark gree	Lo	Mixed Dishes - Grain-based
58161530	Rice, brown, with other vegetables,	Lo	Mixed Dishes - Grain-based
58161532	Rice, brown, with other vegetables,	Lo	Mixed Dishes - Grain-based
58161534	Rice, brown, with other vegetables,	Lo	Mixed Dishes - Grain-based
58161710	Rice croquette	Lo	Mixed Dishes - Grain-based
58162090	Stuffed pepper, with meat	Lo	Mixed Dishes - Bean/Vegetable-based
58162110	Stuffed pepper, with rice and meat	Lo	Mixed Dishes - Bean/Vegetable-based
58162120	Stuffed pepper, with rice, meatless	Lo	Mixed Dishes - Bean/Vegetable-based
58162130	Stuffed tomato, with rice and meat	Lo	Mixed Dishes - Bean/Vegetable-based
58162140	Stuffed tomato, with rice, meatless	Lo	Mixed Dishes - Bean/Vegetable-based
58162310	Rice pilaf	Lo	Mixed Dishes - Grain-based
58163110	Rice with gravy	Lo	Mixed Dishes - Grain-based
58163130	Dirty rice	Lo	Mixed Dishes - Grain-based
58163210	Rice, creamed	Lo	Mixed Dishes - Grain-based
58163310	Flavored rice mixture	Lo	Mixed Dishes - Grain-based
58163330	Flavored rice mixture with cheese	Lo	Mixed Dishes - Grain-based
58163360	Flavored rice, brown and wild	Lo	Mixed Dishes - Grain-based
58163380	Flavored rice and pasta mixture	Lo	Mixed Dishes - Grain-based
58163400	Flavored rice and pasta mixture, re	Lo	Mixed Dishes - Grain-based
58163405	Spanish rice, from restaurant	Lo	Mixed Dishes - Grain-based
58163410	Spanish rice, fat added	Lo	Mixed Dishes - Grain-based
58163420	Spanish rice, no added fat	Lo	Mixed Dishes - Grain-based
58163430	Spanish rice, NS as to fat	Lo	Mixed Dishes - Grain-based
58163450	Spanish rice with ground beef	Lo	Mixed Dishes - Grain-based
58163510	Rice dressing	Lo	Mixed Dishes - Grain-based
58163610	Rice-vegetable medley	Lo	Mixed Dishes - Grain-based
58164110	Rice with raisins	Lo	Mixed Dishes - Grain-based
58164500	Rice, white, with cheese and/or crea	Lo	Mixed Dishes - Grain-based
58164510	Rice, white, with cheese and/or crea	Lo	Mixed Dishes - Grain-based
58164520	Rice, white, with cheese and/or crea	Lo	Mixed Dishes - Grain-based

58164530	Rice, white, with gravy, NS as to fat	Lo	Mixed Dishes - Grain-based
58164540	Rice, white, with gravy, no added fa	Lo	Mixed Dishes - Grain-based
58164550	Rice, white, with gravy, fat added	Lo	Mixed Dishes - Grain-based
58164560	Rice, white, with soy-based sauce,	Lo	Mixed Dishes - Asian
58164570	Rice, white, with soy-based sauce,	Lo	Mixed Dishes - Asian
58164580	Rice, white, with soy-based sauce,	Lo	Mixed Dishes - Asian
58164800	Rice, brown, with cheese and/or cre	Lo	Mixed Dishes - Grain-based
58164820	Rice, brown, with cheese and/or cre	Lo	Mixed Dishes - Grain-based
58164830	Rice, brown, with gravy, NS as to fa	Lo	Mixed Dishes - Grain-based
58164840	Rice, brown, with gravy, no added f	Lo	Mixed Dishes - Grain-based
58164850	Rice, brown, with gravy, fat added	Lo	Mixed Dishes - Grain-based
58164870	Rice, brown, with soy-based sauce,	Lo	Mixed Dishes - Asian
58164880	Rice, brown, with soy-based sauce,	Lo	Mixed Dishes - Asian
58165000	Rice, white, with vegetables, chees	Lo	Mixed Dishes - Grain-based
58165010	Rice, white, with vegetables, chees	Lo	Mixed Dishes - Grain-based
58165020	Rice, white, with vegetables, chees	Lo	Mixed Dishes - Grain-based
58165030	Rice, white, with vegetables and gra	Lo	Mixed Dishes - Grain-based
58165040	Rice, white, with vegetables and gra	Lo	Mixed Dishes - Grain-based
58165050	Rice, white, with vegetables and gra	Lo	Mixed Dishes - Grain-based
58165060	Rice, white, with vegetables, soy-ba	Lo	Mixed Dishes - Asian
58165070	Rice, white, with vegetables, soy-ba	Lo	Mixed Dishes - Asian
58165080	Rice, white, with vegetables, soy-ba	Lo	Mixed Dishes - Asian
58165400	Rice, brown, with vegetables, chees	Lo	Mixed Dishes - Grain-based
58165410	Rice, brown, with vegetables, chees	Lo	Mixed Dishes - Grain-based
58165420	Rice, brown, with vegetables, chees	Lo	Mixed Dishes - Grain-based
58165430	Rice, brown, with vegetables and gr	Lo	Mixed Dishes - Grain-based
58165440	Rice, brown, with vegetables and gr	Lo	Mixed Dishes - Grain-based
58165460	Rice, brown, with vegetables, soy-b	Lo	Mixed Dishes - Asian
58165470	Rice, brown, with vegetables, soy-b	Lo	Mixed Dishes - Asian
58165480	Rice, brown, with vegetables, soy-b	Lo	Mixed Dishes - Asian
58174000	Upma, Indian breakfast dish	Lo	Cooked Cereals
58174100	Dosa (Indian), with filling	Lo	Mixed Dishes - Grain-based
58175000	Vada, fried dumpling	Lo	Mixed Dishes - Grain-based
58200100	Wrap sandwich, filled with meat, po	Lo	Mixed Dishes - Sandwiches (single code)
58200250	Wrap sandwich, filled with vegetabl	Lo	Mixed Dishes - Sandwiches (single code)
58200300	Wrap sandwich, filled with meat, po	Lo	Mixed Dishes - Sandwiches (single code)
58201005	Jelly sandwich, regular jelly, on whit	Lo	Mixed Dishes - Sandwiches (single code)
58201015	Jelly sandwich, regular jelly, on whe	Lo	Mixed Dishes - Sandwiches (single code)
58201025	Jelly sandwich, regular jelly, on who	Lo	Mixed Dishes - Sandwiches (single code)
58201035	Jelly sandwich, reduced sugar jelly,	Lo	Mixed Dishes - Sandwiches (single code)
58201045	Jelly sandwich, reduced sugar jelly,	Lo	Mixed Dishes - Sandwiches (single code)
58301020	Lasagna with cheese and sauce, di	Lo	Mixed Dishes - Grain-based
58301030	Veal lasagna, diet frozen meal	Lo	Mixed Dishes - Grain-based
58301050	Lasagna with cheese and meat sau	Lo	Mixed Dishes - Grain-based
58301080	Lasagna with cheese and meat sau	Lo	Mixed Dishes - Grain-based
58301110	Vegetable lasagna, frozen meal	Lo	Mixed Dishes - Grain-based
58301150	Zucchini lasagna, diet frozen meal	Lo	Mixed Dishes - Grain-based
58302000	Macaroni and cheese, diet frozen m	Lo	Mixed Dishes - Grain-based
58302010	Macaroni and cheese with apples, v	Lo	Mixed Dishes - Grain-based
58302050	Beef and noodles with meat sauce	Lo	Mixed Dishes - Meat, Poultry, Seafood
58302060	Spaghetti or noodles with beef in to	Lo	Mixed Dishes - Grain-based

58302080	Noodles with vegetables in tomato-	Lo	Mixed Dishes - Grain-based
58303100	Rice, with broccoli, cheese sauce, f	Lo	Mixed Dishes - Grain-based
58304010	Spaghetti and meatballs dinner, NF	Lo	Mixed Dishes - Grain-based
58304050	Spaghetti with meat and mushroom	Lo	Mixed Dishes - Grain-based
58304060	Spaghetti with meat sauce, diet froz	Lo	Mixed Dishes - Grain-based
58304200	Ravioli, cheese-filled, with tomato s	Lo	Mixed Dishes - Grain-based
58304220	Rigatoni with meat sauce and chee	Lo	Mixed Dishes - Grain-based
58304230	Ravioli, cheese-filled, with vegetabl	Lo	Mixed Dishes - Grain-based
58304250	Manicotti, cheese-filled, with tomato	Lo	Mixed Dishes - Grain-based
58304400	Linguini with vegetables and seafoo	Lo	Mixed Dishes - Grain-based
58305250	Pasta with vegetable and cheese sa	Lo	Mixed Dishes - Grain-based
58306010	Beef enchilada dinner, NFS, frozen	Lo	Mixed Dishes - Mexican
58306020	Beef enchilada, chili gravy, rice, ref	Lo	Mixed Dishes - Mexican
58306050	Cheese enchilada with beans and r	Lo	Mixed Dishes - Mexican
58306070	Cheese enchilada, frozen meal	Lo	Mixed Dishes - Mexican
58306100	Chicken enchilada, diet frozen mea	Lo	Mixed Dishes - Mexican
58306150	Chicken enchilada with salsa, rice,	Lo	Mixed Dishes - Mexican
58310210	Sausage and french toast, frozen m	Lo	Mixed Dishes - Meat, Poultry, Seafood
58310310	Pancakes and sausage, frozen mea	Lo	Mixed Dishes - Meat, Poultry, Seafood
58400000	Soup, NFS	Lo	Mixed Dishes - Soups
58400100	Noodle soup, NFS	Lo	Mixed Dishes - Soups
58400200	Rice soup, NFS	Lo	Mixed Dishes - Soups
58401010	Barley soup, home recipe, canned,	Lo	Mixed Dishes - Soups
58401200	Barley soup, sweet, with or without	Lo	Mixed Dishes - Soups
58402010	Beef noodle soup, canned or ready-	Lo	Mixed Dishes - Soups
58402020	Beef dumpling soup, home recipe,	Lo	Mixed Dishes - Soups
58402030	Beef rice soup, home recipe, canne	Lo	Mixed Dishes - Soups
58402100	Beef noodle soup, home recipe	Lo	Mixed Dishes - Soups
58403010	Chicken or turkey noodle soup, can	Lo	Mixed Dishes - Soups
58403020	Chicken noodle soup, canned, undi	Lo	Mixed Dishes - Soups
58403030	Chicken noodle soup, canned, low	Lo	Mixed Dishes - Soups
58403040	Chicken or turkey noodle soup, hom	Lo	Mixed Dishes - Soups
58403050	Chicken or turkey noodle soup, crea	Lo	Mixed Dishes - Soups
58403060	Chicken or turkey noodle soup, red	Lo	Mixed Dishes - Soups
58403100	Noodle and potato soup, Puerto Ric	Lo	Mixed Dishes - Soups
58404010	Chicken or turkey rice soup, canne	Lo	Mixed Dishes - Soups
58404030	Chicken or turkey rice soup, home	Lo	Mixed Dishes - Soups
58404040	Chicken or turkey rice soup, reduce	Lo	Mixed Dishes - Soups
58404100	Rice and potato soup, Puerto Rican	Lo	Mixed Dishes - Soups
58404500	Matzo ball soup	Lo	Mixed Dishes - Soups
58404510	Chicken or turkey soup with dumpli	Lo	Mixed Dishes - Soups
58404520	Chicken or turkey soup with dumpli	Lo	Mixed Dishes - Soups
58406010	Turkey noodle soup	Lo	Mixed Dishes - Soups
58406020	Turkey noodle soup, home recipe	Lo	Mixed Dishes - Soups
58407000	Instant soup, NFS	Lo	Mixed Dishes - Soups
58407010	Instant soup, noodle	Lo	Mixed Dishes - Soups
58407030	Soup, mostly noodles	Lo	Mixed Dishes - Soups
58407035	Soup, mostly noodles, reduced sod	Lo	Mixed Dishes - Soups
58407040	Instant soup, rice	Lo	Mixed Dishes - Soups
58407050	Instant soup, noodle with egg, shrin	Lo	Mixed Dishes - Soups
58408010	Wonton soup	Lo	Mixed Dishes - Soups

58408500	Noodle soup with vegetables, Asian	Lo	Mixed Dishes - Soups
58409000	Noodle soup, with fish ball, shrimp,	Lo	Mixed Dishes - Soups
58421000	Sopa seca, Mexican style, NFS	Lo	Mixed Dishes - Soups
58421010	Sopa Seca de Fideo, Mexican style	Lo	Mixed Dishes - Soups
58421020	Sopa de Fideo Aguada, Mexican sty	Lo	Mixed Dishes - Soups
58421060	Sopa seca de arroz, home recipe, M	Lo	Mixed Dishes - Soups
58421080	Sopa de tortilla, Mexican style tortill	Lo	Mixed Dishes - Soups
58450300	Noodle soup, made with milk	Lo	Mixed Dishes - Soups
58503010	Macaroni, tomatoes, and beef, baby	Lo	Baby Foods
58503020	Macaroni, tomatoes, and beef, baby	Lo	Baby Foods
58503050	Macaroni with beef and tomato sau	Lo	Baby Foods
58508000	Macaroni and cheese, baby food, st	Lo	Baby Foods
58508300	Macaroni and cheese, baby food, to	Lo	Baby Foods
58509020	Spaghetti, tomato sauce, and beef,	Lo	Baby Foods
58509100	Ravioli, cheese-filled, with tomato s	Lo	Baby Foods
58509200	Macaroni with vegetables, baby foo	Lo	Baby Foods
59003000	Meat substitute, cereal- and vegeta	Lo	Plant-based Protein Foods
61100600	Clementine, raw	Lo	Fruits
61101010	Grapefruit, raw	Lo	Fruits
61101200	Grapefruit, canned	Lo	Fruits
61101220	Grapefruit, canned or frozen, unswe	Lo	Fruits
61101230	Grapefruit, canned or frozen, in ligh	Lo	Fruits
61104010	Grapefruit and orange sections, raw	Lo	Fruits
61113010	Lemon, raw	Lo	Fruits
61113500	Lemon pie filling	Lo	Sugars
61116010	Lime, raw	Lo	Fruits
61119010	Orange, raw	Lo	Fruits
61119020	Orange, sections, canned, juice pac	Lo	Fruits
61122300	Orange, canned, NFS	Lo	Fruits
61122320	Orange, canned, juice pack	Lo	Fruits
61122330	Orange, canned, in syrup	Lo	Fruits
61122350	Orange, mandarin, canned or froze	Lo	Fruits
61125000	Tangelo, raw	Lo	Fruits
61125010	Tangerine, raw	Lo	Fruits
61201000	Grapefruit juice, NFS	Lo	100% Juice
61201010	Grapefruit juice, 100%, freshly sque	Lo	100% Juice
61201020	Grapefruit juice, 100%, NS as to for	Lo	100% Juice
61201220	Grapefruit juice, 100%, canned, bot	Lo	100% Juice
61201225	Grapefruit juice, 100%, with calcium	Lo	100% Juice
61201620	Grapefruit juice,100%, frozen, recon	Lo	100% Juice
61204000	Lemon juice, 100%, NS as to form	Lo	Condiments and Sauces
61204010	Lemon juice, 100%, freshly squeeze	Lo	Condiments and Sauces
61204200	Lemon juice, 100%, canned or bottl	Lo	Condiments and Sauces
61204600	Lemon juice, frozen	Lo	Condiments and Sauces
61207000	Lime juice, 100%, NS as to form	Lo	Condiments and Sauces
61207010	Lime juice, 100%, freshly squeezed	Lo	Condiments and Sauces
61207200	Lime juice, 100%, canned or bottled	Lo	Condiments and Sauces
61210000	Orange juice, 100%, NFS	Lo	100% Juice
61210010	Orange juice, 100%, freshly squee	Lo	100% Juice
61210220	Orange juice, 100%, canned, bottle	Lo	100% Juice
61210230	Orange juice, canned, bottled or in	Lo	100% Juice

61210250	Orange juice, 100%, with calcium a	Lo	100% Juice
61210620	Orange juice, 100%, frozen, recons	Lo	100% Juice
61210720	Orange juice, 100%, frozen, not rec	Lo	100% Juice
61210820	Orange juice, 100%, with calcium a	Lo	100% Juice
61213000	Tangerine juice, NFS	Lo	100% Juice
61213220	Tangerine juice, 100%	Lo	100% Juice
61213620	Tangerine juice, frozen (reconstitute	Lo	100% Juice
61213800	Fruit juice blend, citrus, 100% juice	Lo	100% Juice
61213900	Fruit juice blend, citrus, 100% juice,	Lo	100% Juice
61214000	Grape-tangerine-lemon juice	Lo	100% Juice
61216010	Grapefruit and orange juice, fresh	Lo	100% Juice
61216220	Grapefruit and orange juice, cannec	Lo	100% Juice
61219000	Orange and banana juice	Lo	100% Juice
61219100	Pineapple-orange-banana juice	Lo	100% Juice
61219150	Orange-white grape-peach juice	Lo	100% Juice
61222000	Pineapple-grapefruit juice, NFS	Lo	100% Juice
61225000	Pineapple-orange juice, NFS	Lo	100% Juice
61225200	Pineapple-orange juice, canned, NS	Lo	100% Juice
61225220	Pineapple-orange juice, canned, bo	Lo	100% Juice
61225600	Pineapple-orange juice, frozen (rec	Lo	100% Juice
61226000	Strawberry-banana-orange juice	Lo	100% Juice
62101000	Fruit, dried, NFS, uncooked	Lo	Fruits
62101050	Fruit mixture, dried	Lo	Fruits
62101100	Apple, dried	Lo	Fruits
62101200	Apple, dried, cooked, NS as to swe	Lo	Fruits
62101220	Apple, dried, cooked, unsweetened	Lo	Fruits
62101230	Apple, dried, cooked, with sugar	Lo	Fruits
62101300	Apple chips	Lo	Fruits
62104100	Apricot, dried	Lo	Fruits
62104200	Apricot, dried, cooked, NS as to sw	Lo	Fruits
62104220	Apricot, dried, cooked, unsweetene	Lo	Fruits
62104230	Apricot, dried, cooked, with sugar	Lo	Fruits
62105000	Blueberries, dried	Lo	Fruits
62106000	Cherries, dried	Lo	Fruits
62107200	Banana chips	Lo	Fruits
62108100	Currants, dried	Lo	Fruits
62109100	Cranberries, dried	Lo	Fruits
62110100	Date	Lo	Fruits
62113100	Fig, dried	Lo	Fruits
62113220	Fig, dried, cooked, unsweetened	Lo	Fruits
62114000	Lychee, dried	Lo	Fruits
62114050	Mango, dried	Lo	Fruits
62114110	Papaya, dried	Lo	Fruits
62116100	Peach, dried	Lo	Fruits
62116220	Peach, dried, cooked, unsweetened	Lo	Fruits
62116230	Peach, dried, cooked, with sugar	Lo	Fruits
62119100	Pear, dried	Lo	Fruits
62119200	Pear, dried, cooked, NS as to swee	Lo	Fruits
62119230	Pear, dried, cooked, with sugar	Lo	Fruits
62120000	Persimmon, dried	Lo	Fruits
62120100	Pineapple, dried	Lo	Fruits

62121100	Plum, rock salt, dried	Lo	Fruits
62122100	Prune, dried	Lo	Fruits
62122200	Prune, dried, cooked, NS as to swe	Lo	Fruits
62122220	Prune, dried, cooked, unsweetened	Lo	Fruits
62122230	Prune, dried, cooked, with sugar	Lo	Fruits
62125100	Raisins	Lo	Fruits
62125110	Raisins, cooked	Lo	Fruits
62126000	Tamarind, dried	Lo	Fruits
63101110	Applesauce, regular	Lo	Fruits
63101120	Applesauce, unsweetened	Lo	Fruits
63101130	Applesauce, stewed apples, with su	Lo	Fruits
63101140	Applesauce, stewed apples, sweete	Lo	Fruits
63101150	Applesauce, flavored	Lo	Fruits
63101210	Apple pie filling	Lo	Fruits
63101310	Apple, baked	Lo	Fruits
63101320	Apple, baked, unsweetened	Lo	Fruits
63101330	Apple, baked, with sugar	Lo	Fruits
63101410	Apple rings, fried	Lo	Fruits
63101420	Apple, pickled	Lo	Fruits
63101500	Apple, fried	Lo	Fruits
63103110	Apricot, canned	Lo	Fruits
63103120	Apricot, cooked or canned, unswee	Lo	Fruits
63103130	Apricot, cooked or canned, in heavy	Lo	Fruits
63103140	Apricot, cooked or canned, in light s	Lo	Fruits
63103150	Apricot, cooked or canned, drained	Lo	Fruits
63103170	Apricot, cooked or canned, juice pa	Lo	Fruits
63105010	Avocado, raw	Lo	Vegetables, excluding Potatoes
63107010	Banana, raw	Lo	Fruits
63107050	Banana, white, Guineo blanco mad	Lo	Fruits
63107070	Banana, Chinese, raw	Lo	Fruits
63107110	Banana, baked	Lo	Fruits
63107210	Banana, ripe, fried	Lo	Fruits
63107310	Banana, ripe, boiled	Lo	Fruits
63107410	Banana, fried	Lo	Fruits
63109010	Cantaloupe, raw	Lo	Fruits
63109700	Starfruit, raw	Lo	Fruits
63110010	Cassaba melon, raw	Lo	Fruits
63111010	Cherries, maraschino	Lo	Fruits
63113030	Cherry pie filling	Lo	Sugars
63113110	Cherries, sour, red, cooked, unswe	Lo	Fruits
63115110	Cherries, canned	Lo	Fruits
63115120	Cherries, sweet, cooked, unsweete	Lo	Fruits
63115130	Cherries, sweet, cooked or canned,	Lo	Fruits
63115140	Cherries, sweet, cooked or canned,	Lo	Fruits
63115150	Cherries, sweet, cooked or canned,	Lo	Fruits
63115170	Cherries, sweet, cooked or canned,	Lo	Fruits
63119110	Fig, canned	Lo	Fruits
63119130	Fig, cooked or canned, in heavy syr	Lo	Fruits
63119140	Figs, cooked or canned, in light syru	Lo	Fruits
63123110	Grapes, seedless, cooked or canne	Lo	Fruits
63125100	Guava shell, canned in heavy syrup	Lo	Fruits

63126500	Kiwi fruit, raw	Lo	Fruits
63126510	Lychee	Lo	Fruits
63126600	Lychee, cooked or canned, in sugar	Lo	Fruits
63126700	Longans, raw	Lo	Fruits
63127010	Honeydew melon, raw	Lo	Fruits
63127610	Honeydew, frozen	Lo	Fruits
63129010	Mango, raw	Lo	Fruits
63129030	Mango, canned	Lo	Fruits
63129050	Mango, frozen	Lo	Fruits
63131110	Nectarine, cooked	Lo	Fruits
63133010	Papaya, raw	Lo	Fruits
63133050	Papaya, green, cooked	Lo	Fruits
63133100	Papaya, canned	Lo	Fruits
63134010	Passion fruit, raw	Lo	Fruits
63135110	Peach, canned, NFS	Lo	Fruits
63135120	Peach, cooked or canned, unsweet	Lo	Fruits
63135130	Peach, cooked or canned, in heavy	Lo	Fruits
63135140	Peach, canned, in syrup	Lo	Fruits
63135150	Peach, cooked or canned, drained s	Lo	Fruits
63135170	Peach, canned, juice pack	Lo	Fruits
63135660	Peach, spiced	Lo	Fruits
63137110	Pear, canned, NFS	Lo	Fruits
63137120	Pear, cooked or canned, unsweeter	Lo	Fruits
63137130	Pear, cooked or canned, in heavy s	Lo	Fruits
63137140	Pear, canned, in syrup	Lo	Fruits
63137150	Pear, cooked or canned, drained so	Lo	Fruits
63137170	Pear, canned, juice pack	Lo	Fruits
63141010	Pineapple, raw	Lo	Fruits
63141110	Pineapple, canned, NFS	Lo	Fruits
63141120	Pineapple, cooked or canned, unsw	Lo	Fruits
63141130	Pineapple, cooked or canned, in he	Lo	Fruits
63141140	Pineapple, canned, in syrup	Lo	Fruits
63141150	Pineapple, cooked or canned, drain	Lo	Fruits
63141170	Pineapple, canned, juice pack	Lo	Fruits
63141200	Pineapple, frozen	Lo	Fruits
63143110	Plum, canned	Lo	Fruits
63143130	Plum, cooked or canned, in heavy s	Lo	Fruits
63143140	Plum, cooked or canned, in light syr	Lo	Fruits
63143150	Plum, cooked or canned, drained s	Lo	Fruits
63143170	Plum, cooked or canned, juice pack	Lo	Fruits
63145010	Pomegranate, raw	Lo	Fruits
63147010	Rhubarb, raw	Lo	Fruits
63147110	Rhubarb	Lo	Fruits
63147120	Rhubarb, cooked or canned, unswe	Lo	Fruits
63147130	Rhubarb, cooked or canned, in hea	Lo	Fruits
63147140	Rhubarb, cooked or canned, in light	Lo	Fruits
63147600	Rhubarb, frozen, NS as to sweeten	Lo	Fruits
63147620	Rhubarb, frozen, with sugar	Lo	Fruits
63148750	Tamarind	Lo	Fruits
63149010	Watermelon, raw	Lo	Fruits
63201110	Blackberries, cooked or canned, NS	Lo	Fruits

63201800	Blackberries, frozen, sweetened, NS	Lo	Fruits
63203110	Bluberries, canned	Lo	Fruits
63203120	Blueberries, cooked or canned, uns	Lo	Fruits
63203130	Blueberries, cooked or canned, in h	Lo	Fruits
63203700	Blueberry pie filling	Lo	Sugars
63207000	Cranberries, NS as to raw, cooked,	Lo	Fruits
63207110	Cranberry sauce	Lo	Fruits
63219110	Raspberries, cooked or canned, NS	Lo	Fruits
63219120	Raspberries, cooked or canned, un	Lo	Fruits
63219130	Raspberries, cooked or canned, in	Lo	Fruits
63223110	Strawberries, canned	Lo	Fruits
63223120	Strawberries, cooked or canned, ur	Lo	Fruits
63223130	Strawberries, cooked or canned, in	Lo	Fruits
63301010	Ambrosia	Lo	Fruits
63307010	Cranberry-orange relish, uncooked	Lo	Fruits
63307100	Cranberry-raspberry Sauce	Lo	Fruits
63311110	Fruit cocktail, canned, NFS	Lo	Fruits
63311120	Fruit cocktail, cooked or canned, ur	Lo	Fruits
63311130	Fruit cocktail, cooked or canned, in	Lo	Fruits
63311140	Fruit cocktail, canned, in syrup	Lo	Fruits
63311145	Tropical fruit cocktail, cooked or car	Lo	Fruits
63311150	Fruit cocktail, cooked or canned, dr	Lo	Fruits
63311170	Fruit cocktail, canned, juice pack	Lo	Fruits
63401060	Apple, candied	Lo	Fruits
63401070	Fruit, chocolate covered	Lo	Fruits
63401990	Banana, chocolate-covered with nu	Lo	Fruits
63402045	Fried dwarf banana, Puerto Rican s	Lo	Fruits
63402990	Fruit salad, including citrus fruits, w	Lo	Fruits
63403000	Fruit salad, excluding citrus fruits, w	Lo	Fruits
63403100	Fruit dessert with cream and/or pud	Lo	Fruits
63408010	Guacamole with tomatoes	Lo	Condiments and Sauces
63408200	Guacamole with tomatoes and chili	Lo	Condiments and Sauces
63409010	Guacamole, NFS	Lo	Condiments and Sauces
63409015	Guacamole with tomatoes	Lo	Condiments and Sauces
63409020	Chutney	Lo	Condiments and Sauces
63411010	Cranberry salad, congealed	Lo	Fruits
63420100	Fruit juice bar, frozen, orange flavor	Lo	Other Desserts
63420105	Frozen fruit juice bar	Lo	Other Desserts
63420110	Fruit juice bar, frozen, flavor other t	Lo	Other Desserts
63420200	Fruit juice bar, frozen, sweetened w	Lo	Other Desserts
63420205	Frozen fruit juice bar, no sugar add	Lo	Other Desserts
63430100	Sorbet, fruit, noncitrus flavor	Lo	Other Desserts
63430110	Sorbet, fruit, citrus flavor	Lo	Other Desserts
63430150	Sorbet	Lo	Other Desserts
63430500	Fruit juice bar with cream, frozen	Lo	Other Desserts
64100100	Fruit juice, NFS	Lo	100% Juice
64100110	Fruit juice blend, 100% juice	Lo	100% Juice
64100200	Cranberry juice blend, 100% juice	Lo	100% Juice
64100220	Cranberry juice blend, 100% juice, v	Lo	100% Juice
64101010	Apple cider	Lo	100% Juice
64104010	Apple juice, 100%	Lo	100% Juice

64104030	Apple juice, 100%, with calcium add	Lo	100% Juice
64104050	Apple juice, with added vitamin C	Lo	100% Juice
64104090	Apple juice with added vitamin C ar	Lo	100% Juice
64104150	Apple-cherry juice	Lo	100% Juice
64104200	Apple-pear juice	Lo	100% Juice
64104450	Apple-raspberry juice	Lo	100% Juice
64104500	Apple-grape juice	Lo	100% Juice
64104600	Blackberry juice, 100%	Lo	100% Juice
64104610	Blueberry juice	Lo	100% Juice
64105400	Cranberry juice, 100%, not a blend	Lo	100% Juice
64105500	Cranberry-white grape juice mixture	Lo	100% Juice
64116010	Grape juice, NFS	Lo	100% Juice
64116020	Grape juice, 100%	Lo	100% Juice
64116030	Grape juice, with sugar	Lo	100% Juice
64116040	Grape juice, low-calorie sweetener	Lo	100% Juice
64116050	Grape juice, NS as to sweetened or	Lo	100% Juice
64116060	Grape juice, 100%, with calcium ad	Lo	100% Juice
64116100	Grape juice, unsweetened, with add	Lo	100% Juice
64120010	Papaya juice, 100%	Lo	100% Juice
64121000	Passion fruit juice, 100%	Lo	100% Juice
64122030	Peach juice, with sugar	Lo	100% Juice
64124010	Pineapple juice, NS as to sweetene	Lo	100% Juice
64124020	Pineapple juice, 100%	Lo	100% Juice
64124060	Pineapple juice, unsweetened, with	Lo	100% Juice
64124200	Pineapple-apple-guava juice, with a	Lo	100% Juice
64125000	Pineapple juice-non-citrus juice bler	Lo	100% Juice
64126000	Pomegranate juice, 100%	Lo	100% Juice
64132010	Prune juice, 100%	Lo	100% Juice
64132020	Prune juice, unsweetened	Lo	100% Juice
64132500	Strawberry juice, 100%	Lo	100% Juice
64133100	Watermelon juice, 100%	Lo	100% Juice
64134000	Fruit smoothie drink, made with frui	Lo	Sweetened Beverages
64134030	Fruit smoothie juice drink, no dairy	Lo	Sweetened Beverages
64134100	Fruit smoothie, light	Lo	Sweetened Beverages
64134200	Fruit smoothie, bottled	Lo	Sweetened Beverages
64200100	Fruit nectar, NFS	Lo	Sweetened Beverages
64201010	Apricot nectar	Lo	Sweetened Beverages
64201500	Banana nectar	Lo	Sweetened Beverages
64202010	Cantaloupe nectar	Lo	Sweetened Beverages
64203020	Guava nectar	Lo	Sweetened Beverages
64204010	Mango nectar	Lo	Sweetened Beverages
64205010	Peach nectar	Lo	Sweetened Beverages
64210010	Papaya nectar	Lo	Sweetened Beverages
64213010	Passion fruit nectar	Lo	Sweetened Beverages
64215010	Pear nectar	Lo	Sweetened Beverages
64221010	Soursop, nectar	Lo	Sweetened Beverages
64401000	Vinegar	Lo	Condiments and Sauces
67100100	Fruit, baby food, NFS	Lo	Baby Foods
67100110	Fruit bar, with added vitamin C, bab	Lo	Baby Foods
67100200	Tropical fruit medley, baby food, str	Lo	Baby Foods
67100300	Apples, baby food, toddler	Lo	Baby Foods

67101000	Apple-raspberry, baby food, NS as t	Lo	Baby Foods
67101010	Apple-raspberry, baby food, straine	Lo	Baby Foods
67101020	Apple-raspberry, baby food, junior	Lo	Baby Foods
67102000	Applesauce, baby food, NS as to st	Lo	Baby Foods
67102010	Applesauce, baby food, strained	Lo	Baby Foods
67102020	Applesauce, baby food, junior	Lo	Baby Foods
67104000	Applesauce and apricots, baby food	Lo	Baby Foods
67104010	Applesauce and apricots, baby food	Lo	Baby Foods
67104020	Applesauce and apricots, baby food	Lo	Baby Foods
67104030	Applesauce with bananas, baby foo	Lo	Baby Foods
67104040	Applesauce with bananas, baby foo	Lo	Baby Foods
67104060	Applesauce with bananas, baby foo	Lo	Baby Foods
67104070	Applesauce with cherries, baby food	Lo	Baby Foods
67104080	Applesauce with cherries, baby food	Lo	Baby Foods
67104090	Applesauce with cherries, baby food	Lo	Baby Foods
67105030	Bananas, baby food, strained	Lo	Baby Foods
67106010	Bananas with apples and pears, ba	Lo	Baby Foods
67106030	Bananas with orange, baby food, st	Lo	Baby Foods
67106050	Banana with mixed berries, baby fo	Lo	Baby Foods
67108000	Peaches, baby food, NS as to strain	Lo	Baby Foods
67108010	Peaches, baby food, strained	Lo	Baby Foods
67108020	Peaches, baby food, junior	Lo	Baby Foods
67108030	Peaches, baby food, toddler	Lo	Baby Foods
67109000	Pears, baby food, NS as to strained	Lo	Baby Foods
67109010	Pears, baby food, strained	Lo	Baby Foods
67109020	Pears, baby food, junior	Lo	Baby Foods
67109030	Pears, baby food, toddler	Lo	Baby Foods
67110000	Prunes, baby food, strained	Lo	Baby Foods
67113010	Apples and pears, baby food, strain	Lo	Baby Foods
67113020	Apples and pears, baby food, junior	Lo	Baby Foods
67114000	Pears and pineapple, baby food, NS	Lo	Baby Foods
67114010	Pears and pineapple, baby food, str	Lo	Baby Foods
67114020	Pears and pineapple, baby food, jur	Lo	Baby Foods
67202000	Apple juice, baby food	Lo	Baby Beverages
67202010	Apple juice, with added calcium, ba	Lo	Baby Beverages
67203000	Apple-fruit juice blend, baby food	Lo	Baby Beverages
67203200	Apple-banana juice, baby food	Lo	Baby Beverages
67203400	Apple-cherry juice, baby food	Lo	Baby Beverages
67203450	Apple-cranberry juice, baby food	Lo	Baby Beverages
67203500	Apple-grape juice, baby food	Lo	Baby Beverages
67203510	Apple-grape juice, with added calciu	Lo	Baby Beverages
67203600	Apple-peach juice, baby food	Lo	Baby Beverages
67203700	Apple-prune juice, baby food	Lo	Baby Beverages
67203800	Grape juice, baby food	Lo	Baby Beverages
67204000	Mixed fruit juice, not citrus, baby foo	Lo	Baby Beverages
67204100	Mixed fruit juice, not citrus, with add	Lo	Baby Beverages
67205000	Orange juice, baby food	Lo	Baby Beverages
67211000	Orange-apple-banana juice, baby fo	Lo	Baby Beverages
67212000	Pear juice, baby food	Lo	Baby Beverages
67230000	Apple-sweet potato juice, baby food	Lo	Baby Beverages
67230500	Orange-carrot juice, baby food	Lo	Baby Beverages

67250100	Banana juice with lowfat yogurt, bab	Lo	Baby Foods
67250150	Mixed fruit juice with lowfat yogurt, l	Lo	Baby Foods
67260000	Fruit juice and water drink, with high	Lo	Baby Beverages
67304000	Plums, baby food, NS as to strained	Lo	Baby Foods
67304010	Plums, baby food, strained	Lo	Baby Foods
67304020	Plums, baby food, junior	Lo	Baby Foods
67304030	Plums, bananas, and rice, baby foo	Lo	Baby Foods
67304500	Prunes with oatmeal, baby food, str	Lo	Baby Foods
67307010	Apricots, baby food, strained	Lo	Baby Foods
67307020	Apricots, baby food, junior	Lo	Baby Foods
67308000	Bananas, baby food, NS as to strai	Lo	Baby Foods
67308020	Bananas, baby food, junior	Lo	Baby Foods
67309000	Bananas and pineapple, baby food,	Lo	Baby Foods
67309010	Bananas and pineapple, baby food,	Lo	Baby Foods
67309020	Bananas and pineapple, baby food,	Lo	Baby Foods
67309030	Bananas and strawberry, baby food	Lo	Baby Foods
67404010	Fruit dessert, baby food, strained	Lo	Baby Foods
67404020	Fruit dessert, baby food, junior	Lo	Baby Foods
67404070	Apple yogurt dessert, baby food, str	Lo	Baby Foods
67404110	Banana apple dessert, baby food, s	Lo	Baby Foods
67404300	Blueberry yogurt dessert, baby food	Lo	Baby Foods
67404500	Mixed fruit yogurt dessert, baby foo	Lo	Baby Foods
67405000	Peach cobbler, baby food, NS as to	Lo	Baby Foods
67405010	Peach cobbler, baby food, strained	Lo	Baby Foods
67405020	Peach cobbler, baby food, junior	Lo	Baby Foods
67408010	Banana pudding, baby food, straine	Lo	Baby Foods
67408500	Banana yogurt dessert, baby food, s	Lo	Baby Foods
67412010	Dutch apple dessert, baby food, stra	Lo	Baby Foods
67412020	Dutch apple dessert, baby food, jun	Lo	Baby Foods
67413700	Peach yogurt dessert, baby food, st	Lo	Baby Foods
67414010	Pineapple dessert, baby food, strain	Lo	Baby Foods
67414100	Mango dessert, baby food	Lo	Baby Foods
67415010	Tutti-fruitti pudding, baby food, strai	Lo	Baby Foods
67415020	Tutti-fruitti pudding, baby food, junio	Lo	Baby Foods
67430000	Fruit flavored snack, baby food	Lo	Baby Foods
67430500	Yogurt and fruit snack, baby food	Lo	Baby Foods
67501000	Apples and chicken, baby food, stra	Lo	Baby Foods
67501100	Apples with ham, baby food, straine	Lo	Baby Foods
67600100	Apples and sweet potatoes, baby fo	Lo	Baby Foods
71000100	Potato, NFS	Lo	White Potatoes
71001000	White potato, raw, with or without p	Lo	White Potatoes
71100100	Potato, baked, NFS	Lo	White Potatoes
71101000	Potato, baked, peel not eaten	Lo	White Potatoes
71101100	White potato, baked, peel eaten, NS	Lo	White Potatoes
71101110	White potato, baked, peel eaten, fa	Lo	White Potatoes
71101120	White potato, baked, peel eaten, fa	Lo	White Potatoes
71101150	White potato skins, with adhering fl	Lo	White Potatoes
71102980	Potato, boiled, NFS	Lo	White Potatoes
71102990	Potato, boiled, ready-to-heat	Lo	White Potatoes
71103000	Potato, boiled, from fresh, peel not	Lo	White Potatoes
71103010	Potato, boiled, from fresh, peel not	Lo	White Potatoes

71103020	Potato, boiled, from fresh, peel not	Lo	White Potatoes
71103030	Potato, boiled, from fresh, peel not	Lo	White Potatoes
71103040	Potato, boiled, from fresh, peel not	Lo	White Potatoes
71103050	Potato, boiled, from fresh, peel not	Lo	White Potatoes
71103100	White potato, boiled with peel, peel	Lo	White Potatoes
71103105	Potato, boiled, from fresh, peel eate	Lo	White Potatoes
71103110	White potato, boiled with peel, peel	Lo	White Potatoes
71103115	Potato, boiled, from fresh, peel eate	Lo	White Potatoes
71103120	White potato, boiled with peel, peel	Lo	White Potatoes
71103125	Potato, boiled, from fresh, peel eat	Lo	White Potatoes
71103135	Potato, boiled, from fresh, peel eate	Lo	White Potatoes
71103140	Potato, boiled, from fresh, peel eate	Lo	White Potatoes
71103150	Potato, boiled, from fresh, peel eate	Lo	White Potatoes
71103210	White potato, boiled, without peel, c	Lo	White Potatoes
71103220	White potato, boiled, without peel, c	Lo	White Potatoes
71103310	Potato, canned, fat added, NS as to	Lo	White Potatoes
71103320	Potato, canned, no added fat	Lo	White Potatoes
71104000	White potato, roasted, NS as to fat	Lo	White Potatoes
71104010	White potato, roasted, fat not addec	Lo	White Potatoes
71104020	White potato, roasted, fat added in	Lo	White Potatoes
71104030	Potato, roasted, NFS	Lo	White Potatoes
71104040	Potato, roasted, from fresh, peel ea	Lo	White Potatoes
71104050	Potato, roasted, from fresh, peel ea	Lo	White Potatoes
71104060	Potato, roasted, from fresh, peel ea	Lo	White Potatoes
71104070	Potato, roasted, from fresh, peel ea	Lo	White Potatoes
71104080	Potato, roasted, from fresh, peel ea	Lo	White Potatoes
71104090	Potato, roasted, from fresh, peel ea	Lo	White Potatoes
71104100	Potato, roasted, from fresh, peel no	Lo	White Potatoes
71104120	Potato, roasted, from fresh, peel no	Lo	White Potatoes
71104130	Potato, roasted, from fresh, peel no	Lo	White Potatoes
71104140	Potato, roasted, from fresh, peel no	Lo	White Potatoes
71104150	Potato, roasted, from fresh, peel no	Lo	White Potatoes
71104200	Potato, roasted, ready-to-heat	Lo	White Potatoes
71106000	Stewed potatoes, Puerto Rican styl	Lo	White Potatoes
71200010	Potato chips, NFS	Lo	Savory Snacks
71200100	Potato chips, plain	Lo	Savory Snacks
71200110	Potato chips, barbecue flavored	Lo	Savory Snacks
71200120	Potato chips, sour cream and onion	Lo	Savory Snacks
71200130	Potato chips, cheese flavored	Lo	Savory Snacks
71200140	Potato chips, other flavored	Lo	Savory Snacks
71200200	Potato chips, ruffled, plain	Lo	Savory Snacks
71200210	Potato chips, ruffled, barbecue flav	Lo	Savory Snacks
71200220	Potato chips, ruffled, sour cream ar	Lo	Savory Snacks
71200230	Potato chips, ruffled, cheese flavore	Lo	Savory Snacks
71200240	Potato chips, ruffled, other flavored	Lo	Savory Snacks
71200300	Potato chips, restructured, plain	Lo	Savory Snacks
71200310	Potato chips, restructured, flavored	Lo	Savory Snacks
71200400	Potato chips, baked, plain	Lo	Savory Snacks
71200410	Potato chips, baked, flavored	Lo	Savory Snacks
71201010	White potato, chips	Lo	Savory Snacks
71201015	White potato chips, regular cut	Lo	Savory Snacks

71201020	White potato chips, ruffled, rippled,	Lo	Savory Snacks
71201050	Potato chips, reduced fat	Lo	Savory Snacks
71201060	Potato chips, fat free	Lo	Savory Snacks
71201080	White potato, chips, fat free	Lo	Savory Snacks
71201090	White potato, chips, fat free, made	Lo	Savory Snacks
71201100	White potato, chips, restructured	Lo	Savory Snacks
71201200	Potato chips, restructured, reduced	Lo	Savory Snacks
71201210	Potato chips, restructured, fat free	Lo	Savory Snacks
71201250	White potato, chips, restructured, b	Lo	Savory Snacks
71202000	Potato chips, unsalted	Lo	Savory Snacks
71202100	Potato chips, reduced fat, unsalted	Lo	Savory Snacks
71202500	Potato chips, lightly salted	Lo	Savory Snacks
71202510	Potato chips, restructured, lightly sa	Lo	Savory Snacks
71203010	Potato chips, popped, plain	Lo	Savory Snacks
71203020	Potato chips, popped, flavored	Lo	Savory Snacks
71204000	Potato puffs, cheese-filled	Lo	Savory Snacks
71205000	White potato, sticks	Lo	Savory Snacks
71205020	Potato sticks, plain	Lo	Savory Snacks
71205030	Potato sticks, flavored	Lo	Savory Snacks
71205040	Potato sticks, fry shaped	Lo	Savory Snacks
71211000	White potato skins, chips	Lo	Savory Snacks
71220000	Vegetable chips	Lo	Savory Snacks
71301000	White potato, cooked, with sauce, N	Lo	White Potatoes
71301020	White potato, cooked, with cheese	Lo	White Potatoes
71301021	White potato, cooked, with cheese,	Lo	White Potatoes
71301120	White potato, cooked, with ham and	Lo	White Potatoes
71305010	White potato, scalloped	Lo	White Potatoes
71305011	White potato, scalloped, fat not add	Lo	White Potatoes
71305015	Potato, scalloped, NFS	Lo	White Potatoes
71305020	Potato, scalloped, from fast food or	Lo	White Potatoes
71305030	Potato, scalloped, from fresh	Lo	White Potatoes
71305040	Potato, scalloped, from fresh, with r	Lo	White Potatoes
71305050	Potato, scalloped, from dry mix	Lo	White Potatoes
71305060	Potato, scalloped, from dry mix, wit	Lo	White Potatoes
71305070	Potato, scalloped, ready-to-heat	Lo	White Potatoes
71305110	White potato, scalloped, with ham	Lo	White Potatoes
71400990	Potato, french fries, NFS	Lo	White Potatoes
71401000	Potato, french fries, NS as to fresh	Lo	White Potatoes
71401010	Potato, french fries, from fresh, frie	Lo	White Potatoes
71401015	Potato, french fries, from fresh, bak	Lo	White Potatoes
71401020	Potato, french fries, from frozen, ba	Lo	White Potatoes
71401030	Potato, french fries, fast food	Lo	White Potatoes
71401031	Potato, french fries, restaurant	Lo	White Potatoes
71401032	Potato, french fries, from frozen, fri	Lo	White Potatoes
71401033	Potato, french fries, school	Lo	White Potatoes
71401035	White potato, french fries, from froz	Lo	White Potatoes
71401039	Potato, french fries, with cheese, fa	Lo	White Potatoes
71401041	Potato, french fries, with cheese, sc	Lo	White Potatoes
71401045	Potato, french fries, with chili, fast fo	Lo	White Potatoes
71401050	Potato, french fries, with chili and ch	Lo	White Potatoes
71402040	White potato, french fries, breaded	Lo	White Potatoes

71402500	Potato, french fries, with cheese	Lo	White Potatoes
71402505	White potato, french fries, with chee	Lo	White Potatoes
71402510	Potato, french fries, with chili and ch	Lo	White Potatoes
71402520	Potato, french fries, with chili	Lo	White Potatoes
71403000	White potato, home fries	Lo	White Potatoes
71403010	White potato, home fries, fat not ad	Lo	White Potatoes
71403020	Potato, home fries, NFS	Lo	White Potatoes
71403030	Potato, home fries, from restaurant	Lo	White Potatoes
71403040	Potato, home fries, from fresh	Lo	White Potatoes
71403050	Potato, home fries, ready-to-heat	Lo	White Potatoes
71403500	Potato, home fries, with vegetables	Lo	White Potatoes
71404000	Potato, hash brown, NFS	Lo	White Potatoes
71404010	Potato, hash brown, from fast food	Lo	White Potatoes
71404020	Potato, hash brown, from fast food,	Lo	White Potatoes
71404030	Potato, hash brown, from restauran	Lo	White Potatoes
71404040	Potato, hash brown, from restauran	Lo	White Potatoes
71404050	Potato, hash brown, from school lun	Lo	White Potatoes
71405000	White potato, hash brown, NS as to	Lo	White Potatoes
71405010	Potato, hash brown, from fresh	Lo	White Potatoes
71405019	Potato, hash brown, from fresh, with	Lo	White Potatoes
71405020	White potato, hash brown, from froz	Lo	White Potatoes
71405030	Potato, hash brown, from dry mix	Lo	White Potatoes
71405040	Potato, hash brown, ready-to-heat	Lo	White Potatoes
71405050	Potato, hash brown, ready-to-heat,	Lo	White Potatoes
71405100	White potato, hash brown, with che	Lo	White Potatoes
71410000	Potato skins without topping	Lo	White Potatoes
71410500	Potato skins, with cheese	Lo	White Potatoes
71411000	Potato skins, with cheese and baco	Lo	White Potatoes
71501000	Potato, mashed, NFS	Lo	White Potatoes
71501005	Potato, mashed, from fast food	Lo	White Potatoes
71501006	Potato, mashed, from fast food, with	Lo	White Potatoes
71501007	Potato, mashed, ready-to-heat	Lo	White Potatoes
71501010	Potato, mashed, from fresh, made w	Lo	White Potatoes
71501011	Potato, mashed, from fresh, made w	Lo	White Potatoes
71501012	Potato, mashed, from fresh, made w	Lo	White Potatoes
71501013	Potato, mashed, from fresh, NFS	Lo	White Potatoes
71501015	White potato, from fresh, mashed, m	Lo	White Potatoes
71501016	Potato, mashed, from restaurant	Lo	White Potatoes
71501017	Potato, mashed, from restaurant, w	Lo	White Potatoes
71501018	Potato, mashed, from school lunch	Lo	White Potatoes
71501020	White potato, from fresh, mashed, m	Lo	White Potatoes
71501025	White potato, from fresh, mashed, m	Lo	White Potatoes
71501030	White potato, from fresh, mashed, m	Lo	White Potatoes
71501035	Potato, mashed, from dry mix, NFS	Lo	White Potatoes
71501040	Potato, mashed, from dry mix, mad	Lo	White Potatoes
71501045	Potato, mashed, from dry mix, mad	Lo	White Potatoes
71501050	White potato, from fresh, mashed, m	Lo	White Potatoes
71501054	Potato, mashed, from dry mix, mad	Lo	White Potatoes
71501055	White potato, from fresh, mashed, m	Lo	White Potatoes
71501060	White potato, from dry, mashed, ma	Lo	White Potatoes
71501061	Potato, mashed, ready-to-heat, NFS	Lo	White Potatoes

71501070	White potato, from dry, mashed, ma	Lo	White Potatoes
71501071	Potato, mashed, ready-to-heat, with	Lo	White Potatoes
71501075	Potato, mashed, ready-to-heat, with	Lo	White Potatoes
71501080	White potato, from fresh, mashed, r	Lo	White Potatoes
71501090	White potato, from dry, mashed, ma	Lo	White Potatoes
71501200	White potato, from complete dry mi	Lo	White Potatoes
71501300	White potato, from dry, mashed, NS	Lo	White Potatoes
71501310	White potato, from fresh, mashed, I	Lo	White Potatoes
71503010	Potato patty	Lo	White Potatoes
71505000	Potato tots, NFS	Lo	White Potatoes
71505010	Potato tots, fast food / restaurant	Lo	White Potatoes
71505020	Potato tots, school	Lo	White Potatoes
71505030	Potato tots, from fresh, fried or bak	Lo	White Potatoes
71505040	Potato tots, frozen, baked	Lo	White Potatoes
71505050	Potato tots, frozen, fried	Lo	White Potatoes
71505060	Potato tots, frozen, NS as to fried o	Lo	White Potatoes
71507000	White potato, stuffed, baked, peel n	Lo	White Potatoes
71507005	Potato, baked, peel not eaten, with	Lo	White Potatoes
71507010	Potato, baked, peel not eaten, with	Lo	White Potatoes
71507020	Potato, baked, peel not eaten, with	Lo	White Potatoes
71507025	Potato, baked, peel not eaten, with	Lo	White Potatoes
71507030	Potato, baked, peel not eaten, with	Lo	White Potatoes
71507035	Potato, baked, peel not eaten, with	Lo	White Potatoes
71507040	White potato, stuffed, baked, peel n	Lo	White Potatoes
71507050	White potato, stuffed, baked, peel n	Lo	White Potatoes
71508000	White potato, stuffed, baked, peel e	Lo	White Potatoes
71508001	Potato, baked, peel eaten	Lo	White Potatoes
71508005	Potato, baked, peel eaten, with butt	Lo	White Potatoes
71508010	Potato, baked, peel eaten, with sou	Lo	White Potatoes
71508020	Potato, baked, peel eaten, with che	Lo	White Potatoes
71508025	Potato, baked, peel eaten, with mea	Lo	White Potatoes
71508030	Potato, baked, peel eaten, with chili	Lo	White Potatoes
71508035	Potato, baked, peel eaten, with veg	Lo	White Potatoes
71508040	White potato, stuffed, baked, peel e	Lo	White Potatoes
71508050	White potato, stuffed, baked, peel e	Lo	White Potatoes
71508060	White potato, stuffed, baked, peel e	Lo	White Potatoes
71508070	White potato, stuffed, baked, peel n	Lo	White Potatoes
71508120	White potato, stuffed with ham, broc	Lo	White Potatoes
71600950	Potato salad with egg, from restaura	Lo	White Potatoes
71601010	Potato salad with egg, made with m	Lo	White Potatoes
71601015	Potato salad with egg, made with lig	Lo	White Potatoes
71601020	Potato salad with egg, made with m	Lo	White Potatoes
71601025	Potato salad with egg, made with lig	Lo	White Potatoes
71601035	Potato salad with egg, made with lig	Lo	White Potatoes
71601040	Potato salad with egg, made with Ita	Lo	White Potatoes
71601050	Potato salad with egg, made with ar	Lo	White Potatoes
71602010	Potato salad, German style	Lo	White Potatoes
71602950	Potato salad, from restaurant	Lo	White Potatoes
71603010	Potato salad, made with mayonnais	Lo	White Potatoes
71603015	Potato salad, made with light mayor	Lo	White Potatoes
71603020	Potato salad, made with mayonnais	Lo	White Potatoes

71603025	Potato salad, made with light mayo	Lo	White Potatoes
71603030	Potato salad, made with creamy dre	Lo	White Potatoes
71603040	Potato salad, made with Italian dres	Lo	White Potatoes
71603050	Potato salad, made with any type of	Lo	White Potatoes
71701000	Potato pancake	Lo	White Potatoes
71701500	Lefse (Norwegian)	Lo	White Potatoes
71702000	Potato pudding	Lo	White Potatoes
71703000	Stewed potatoes, Mexican style (Pa	Lo	White Potatoes
71703040	Stewed potatoes with tomatoes, Me	Lo	White Potatoes
71703990	Stewed potatoes	Lo	White Potatoes
71704000	Stewed potatoes with tomatoes	Lo	White Potatoes
71801000	Potato soup, NS as to made with m	Lo	Mixed Dishes - Soups
71801010	Potato soup, cream of, prepared wi	Lo	Mixed Dishes - Soups
71801020	Potato soup, prepared with water	Lo	Mixed Dishes - Soups
71801040	Potato soup, instant, made from dry	Lo	Mixed Dishes - Soups
71801100	Potato and cheese soup	Lo	Mixed Dishes - Soups
71802010	Macaroni and potato soup	Lo	Mixed Dishes - Soups
71803010	Potato chowder	Lo	Mixed Dishes - Soups
71851010	Plantain soup, Puerto Rican style	Lo	Mixed Dishes - Soups
71900100	Plantain, cooked, no added fat	Lo	Vegetables, excluding Potatoes
71900200	Plantain, cooked with oil	Lo	Vegetables, excluding Potatoes
71901010	Green plantains, boiled	Lo	Vegetables, excluding Potatoes
71901110	Fried green plantain, Puerto Rican s	Lo	Vegetables, excluding Potatoes
71905000	Ripe plantain, raw	Lo	Vegetables, excluding Potatoes
71905008	Plantain, cooked, fat added, NS as	Lo	Vegetables, excluding Potatoes
71905010	Ripe plantain, boiled	Lo	Vegetables, excluding Potatoes
71905100	Plantain, cooked with butter or marg	Lo	Vegetables, excluding Potatoes
71905110	Fried ripe plantain, Puerto Rican sty	Lo	Vegetables, excluding Potatoes
71905120	Plantain, ripe, rolled in flour, fried	Lo	Vegetables, excluding Potatoes
71905410	Plantain chips	Lo	Savory Snacks
71910110	Green banana, cooked in salt water	Lo	Vegetables, excluding Potatoes
71910210	Green banana, fried	Lo	Vegetables, excluding Potatoes
71910310	Pickled green bananas, Puerto Rica	Lo	Condiments and Sauces
71930090	Cassava, cooked, NS as to fat adde	Lo	Vegetables, excluding Potatoes
71930100	Cassava, cooked, fat not added in c	Lo	Vegetables, excluding Potatoes
71930120	Cassava, cooked	Lo	Vegetables, excluding Potatoes
71930130	Cassava, cooked, made with oil	Lo	Vegetables, excluding Potatoes
71930190	Yuca fries	Lo	Vegetables, excluding Potatoes
71930200	Casabe, cassava bread	Lo	Quick Breads and Bread Products
71931010	Cassava with creole sauce, Puerto	Lo	Mixed Dishes - Bean/Vegetable-based
71941120	Sweet potatoes, white, Puerto Rican	Lo	Vegetables, excluding Potatoes
71945010	Yam, cooked, Puerto Rican	Lo	Vegetables, excluding Potatoes
71945020	Yam buns; Puerto Rican style	Lo	Quick Breads and Bread Products
71950010	Tannier, cooked	Lo	Vegetables, excluding Potatoes
71961010	Celeriac, cooked	Lo	Vegetables, excluding Potatoes
71962010	Dasheen, boiled	Lo	Vegetables, excluding Potatoes
71962020	Dasheen, cooked	Lo	Vegetables, excluding Potatoes
71962040	Taro, cooked	Lo	Vegetables, excluding Potatoes
71970120	Starchy vegetables, including tannie	Lo	Vegetables, excluding Potatoes
71970130	Starchy vegetables, including tannie	Lo	Vegetables, excluding Potatoes
71970200	Fufu	Lo	Vegetables, excluding Potatoes

71980100	Poi	Lo	Vegetables, excluding Potatoes
71980200	Taro chips	Lo	Savory Snacks
72101200	Beet greens, cooked, NS as to fat a	Lo	Vegetables, excluding Potatoes
72101210	Beet greens, cooked, fat not added	Lo	Vegetables, excluding Potatoes
72101220	Beet greens, cooked	Lo	Vegetables, excluding Potatoes
72103020	Broccoli raab, cooked, fat not addec	Lo	Vegetables, excluding Potatoes
72103030	Broccoli raab, cooked	Lo	Vegetables, excluding Potatoes
72103040	Broccoli raab, cooked, made with o	Lo	Vegetables, excluding Potatoes
72103060	Broccoli raab, cooked, made with m	Lo	Vegetables, excluding Potatoes
72104210	Chard, cooked, fat not added in coo	Lo	Vegetables, excluding Potatoes
72104220	Chard, cooked	Lo	Vegetables, excluding Potatoes
72104230	Chard, cooked, made with oil	Lo	Vegetables, excluding Potatoes
72107200	Collards, cooked, NS as to form, NS	Lo	Vegetables, excluding Potatoes
72107201	Collards, cooked, from fresh, NS as	Lo	Vegetables, excluding Potatoes
72107202	Collards, cooked, from frozen, NS a	Lo	Vegetables, excluding Potatoes
72107203	Collards, cooked, from canned, NS	Lo	Vegetables, excluding Potatoes
72107210	Collards, cooked, NS as to form, fa	Lo	Vegetables, excluding Potatoes
72107211	Collards, fresh, cooked, no added f	Lo	Vegetables, excluding Potatoes
72107212	Collards, frozen, cooked, no added	Lo	Vegetables, excluding Potatoes
72107213	Collards, canned, cooked, no addec	Lo	Vegetables, excluding Potatoes
72107220	Collards, NS as to form, cooked	Lo	Vegetables, excluding Potatoes
72107221	Collards, fresh, cooked, fat added,	Lo	Vegetables, excluding Potatoes
72107222	Collards, frozen, cooked, fat added	Lo	Vegetables, excluding Potatoes
72107223	Collards, canned, cooked, fat addec	Lo	Vegetables, excluding Potatoes
72107227	Collards, fresh, cooked with oil	Lo	Vegetables, excluding Potatoes
72107228	Collards, fresh, cooked with butter c	Lo	Vegetables, excluding Potatoes
72107230	Collards, frozen, cooked with oil	Lo	Vegetables, excluding Potatoes
72107231	Collards, frozen, cooked with butter	Lo	Vegetables, excluding Potatoes
72107233	Collards, canned, cooked with oil	Lo	Vegetables, excluding Potatoes
72107234	Collards, canned, cooked with butte	Lo	Vegetables, excluding Potatoes
72110221	Cress, cooked	Lo	Vegetables, excluding Potatoes
72113210	Dandelion greens, cooked, fat not a	Lo	Vegetables, excluding Potatoes
72116200	Escarole, cooked, NS as to fat adde	Lo	Vegetables, excluding Potatoes
72116210	Escarole, cooked, fat not added in c	Lo	Vegetables, excluding Potatoes
72116220	Escarole, cooked	Lo	Vegetables, excluding Potatoes
72116221	Escarole, cooked, made with oil	Lo	Vegetables, excluding Potatoes
72118200	Greens, cooked, NS as to form, NS	Lo	Vegetables, excluding Potatoes
72118201	Greens, cooked, from fresh, NS as	Lo	Vegetables, excluding Potatoes
72118202	Greens, cooked, from frozen, NS as	Lo	Vegetables, excluding Potatoes
72118210	Greens, cooked, NS as to form, fat	Lo	Vegetables, excluding Potatoes
72118211	Greens, fresh, cooked, no added fa	Lo	Vegetables, excluding Potatoes
72118212	Greens, frozen, cooked, no added f	Lo	Vegetables, excluding Potatoes
72118213	Greens, canned, cooked, no added	Lo	Vegetables, excluding Potatoes
72118220	Greens, NS as to form, cooked	Lo	Vegetables, excluding Potatoes
72118221	Greens, fresh, cooked, fat added	Lo	Vegetables, excluding Potatoes
72118222	Greens, frozen, cooked, fat added	Lo	Vegetables, excluding Potatoes
72118223	Greens, canned, cooked, fat added	Lo	Vegetables, excluding Potatoes
72118224	Greens, cooked, NS as to form, ma	Lo	Vegetables, excluding Potatoes
72118227	Greens, cooked, from fresh, made v	Lo	Vegetables, excluding Potatoes
72118228	Greens, cooked, from fresh, made v	Lo	Vegetables, excluding Potatoes
72118230	Greens, cooked, from frozen, made	Lo	Vegetables, excluding Potatoes

72118233	Greens, cooked, from canned, mad	Lo	Vegetables, excluding Potatoes
72118305	Chamnamul, cooked, fat not added	Lo	Vegetables, excluding Potatoes
72119200	Kale, cooked, NS as to form, NS as	Lo	Vegetables, excluding Potatoes
72119201	Kale, cooked, from fresh, NS as to	Lo	Vegetables, excluding Potatoes
72119202	Kale, cooked, from frozen, NS as to	Lo	Vegetables, excluding Potatoes
72119210	Kale, cooked, NS as to form, fat no	Lo	Vegetables, excluding Potatoes
72119211	Kale, fresh, cooked, no added fat	Lo	Vegetables, excluding Potatoes
72119212	Kale, frozen, cooked, no added fat	Lo	Vegetables, excluding Potatoes
72119213	Kale, canned, cooked, no added fat	Lo	Vegetables, excluding Potatoes
72119220	Kale, NS as to form, cooked	Lo	Vegetables, excluding Potatoes
72119221	Kale, fresh, cooked, fat added	Lo	Vegetables, excluding Potatoes
72119222	Kale, frozen, cooked, fat added	Lo	Vegetables, excluding Potatoes
72119224	Kale, cooked, NS as to form, made	Lo	Vegetables, excluding Potatoes
72119227	Kale, cooked, from fresh, made with	Lo	Vegetables, excluding Potatoes
72119228	Kale, cooked, from fresh, made with	Lo	Vegetables, excluding Potatoes
72119230	Kale, cooked, from frozen, made wi	Lo	Vegetables, excluding Potatoes
72119231	Kale, cooked, from frozen, made wi	Lo	Vegetables, excluding Potatoes
72121210	Mustard cabbage, cooked, fat not a	Lo	Vegetables, excluding Potatoes
72122200	Mustard greens, cooked, NS as to f	Lo	Vegetables, excluding Potatoes
72122201	Mustard greens, cooked, from fresh	Lo	Vegetables, excluding Potatoes
72122202	Mustard greens, cooked, from froze	Lo	Vegetables, excluding Potatoes
72122203	Mustard greens, cooked, from cann	Lo	Vegetables, excluding Potatoes
72122211	Mustard greens, fresh, cooked, no a	Lo	Vegetables, excluding Potatoes
72122212	Mustard greens, frozen, cooked, no	Lo	Vegetables, excluding Potatoes
72122213	Mustard greens, canned, cooked, n	Lo	Vegetables, excluding Potatoes
72122220	Mustard greens, NS as to form, coo	Lo	Vegetables, excluding Potatoes
72122221	Mustard greens, fresh, cooked, fat a	Lo	Vegetables, excluding Potatoes
72122222	Mustard greens, frozen, cooked, fat	Lo	Vegetables, excluding Potatoes
72122223	Mustard greens, canned, cooked, fa	Lo	Vegetables, excluding Potatoes
72122224	Mustard greens, cooked, NS as to f	Lo	Vegetables, excluding Potatoes
72122225	Mustard greens, cooked, NS as to f	Lo	Vegetables, excluding Potatoes
72122227	Mustard greens, cooked, from fresh	Lo	Vegetables, excluding Potatoes
72122228	Mustard greens, cooked, from fresh	Lo	Vegetables, excluding Potatoes
72122229	Mustard greens, cooked, from fresh	Lo	Vegetables, excluding Potatoes
72122234	Mustard greens, cooked, from cann	Lo	Vegetables, excluding Potatoes
72123010	Poke greens, cooked, fat not added	Lo	Vegetables, excluding Potatoes
72123020	Poke greens, cooked	Lo	Vegetables, excluding Potatoes
72123030	Poke greens, cooked, made with oi	Lo	Vegetables, excluding Potatoes
72125200	Spinach, cooked, NS as to form, NS	Lo	Vegetables, excluding Potatoes
72125201	Spinach, cooked, from fresh, NS as	Lo	Vegetables, excluding Potatoes
72125202	Spinach, cooked, from frozen, NS a	Lo	Vegetables, excluding Potatoes
72125203	Spinach, cooked, from canned, NS	Lo	Vegetables, excluding Potatoes
72125210	Spinach, cooked, NS as to form, fat	Lo	Vegetables, excluding Potatoes
72125211	Spinach, fresh, cooked, no added fa	Lo	Vegetables, excluding Potatoes
72125212	Spinach, frozen, cooked, no added	Lo	Vegetables, excluding Potatoes
72125213	Spinach, canned, cooked, no added	Lo	Vegetables, excluding Potatoes
72125214	Spinach, cooked, NS as to form, ma	Lo	Vegetables, excluding Potatoes
72125217	Spinach, fresh, cooked with oil	Lo	Vegetables, excluding Potatoes
72125218	Spinach, fresh, cooked with butter o	Lo	Vegetables, excluding Potatoes
72125219	Spinach, cooked, from fresh, made	Lo	Vegetables, excluding Potatoes
72125220	Spinach, NS as to form, cooked	Lo	Vegetables, excluding Potatoes

72125221	Spinach, fresh, cooked, fat added, I	Lo	Vegetables, excluding Potatoes
72125222	Spinach, frozen, cooked, fat added,	Lo	Vegetables, excluding Potatoes
72125223	Spinach, canned, cooked, fat added	Lo	Vegetables, excluding Potatoes
72125224	Spinach, frozen, cooked with oil	Lo	Vegetables, excluding Potatoes
72125225	Spinach, frozen, cooked with butter	Lo	Vegetables, excluding Potatoes
72125226	Spinach, cooked, from frozen, mad	Lo	Vegetables, excluding Potatoes
72125227	Spinach, canned, cooked with oil	Lo	Vegetables, excluding Potatoes
72125228	Spinach, canned, cooked with butte	Lo	Vegetables, excluding Potatoes
72125229	Spinach, cooked, from canned, ma	Lo	Vegetables, excluding Potatoes
72125230	Spinach, creamed	Lo	Vegetables, excluding Potatoes
72125231	Spinach, from fresh, creamed	Lo	Vegetables, excluding Potatoes
72125232	Spinach, from frozen, creamed	Lo	Vegetables, excluding Potatoes
72125233	Spinach, from canned, creamed	Lo	Vegetables, excluding Potatoes
72125240	Spinach souffle	Lo	Mixed Dishes - Bean/Vegetable-based
72125250	Spinach, cooked, NS as to form, wi	Lo	Vegetables, excluding Potatoes
72125251	Spinach, cooked, from fresh, with c	Lo	Vegetables, excluding Potatoes
72125252	Spinach, cooked, from frozen, with	Lo	Vegetables, excluding Potatoes
72125253	Spinach, cooked, from canned, with	Lo	Vegetables, excluding Potatoes
72125260	Spinach and cheese casserole	Lo	Mixed Dishes - Bean/Vegetable-based
72125310	Palak Paneer	Lo	Mixed Dishes - Bean/Vegetable-based
72125500	Channa Saag	Lo	Mixed Dishes - Bean/Vegetable-based
72126000	Taro leaves, cooked, fat not added	Lo	Vegetables, excluding Potatoes
72126001	Taro leaves, cooked	Lo	Vegetables, excluding Potatoes
72128200	Turnip greens, cooked, NS as to fo	Lo	Vegetables, excluding Potatoes
72128201	Turnip greens, cooked, from fresh,	Lo	Vegetables, excluding Potatoes
72128202	Turnip greens, cooked, from frozen	Lo	Vegetables, excluding Potatoes
72128203	Turnip greens, cooked, from canne	Lo	Vegetables, excluding Potatoes
72128210	Turnip greens, cooked, NS as to fo	Lo	Vegetables, excluding Potatoes
72128211	Turnip greens, fresh, cooked, no ac	Lo	Vegetables, excluding Potatoes
72128212	Turnip greens, frozen, cooked, no a	Lo	Vegetables, excluding Potatoes
72128213	Turnip greens, canned, cooked, no	Lo	Vegetables, excluding Potatoes
72128220	Turrnip greens, NS as to form, cook	Lo	Vegetables, excluding Potatoes
72128221	Turnip greens, fresh, cooked, fat ac	Lo	Vegetables, excluding Potatoes
72128222	Turnip greens, frozen, cooked, fat a	Lo	Vegetables, excluding Potatoes
72128223	Turnip greens, canned, cooked, fat	Lo	Vegetables, excluding Potatoes
72128227	Turnip greens, cooked, from fresh,	Lo	Vegetables, excluding Potatoes
72128228	Turnip greens, cooked, from fresh,	Lo	Vegetables, excluding Potatoes
72128230	Turnip greens, cooked, from frozen	Lo	Vegetables, excluding Potatoes
72128237	Turnip greens, canned, reduced so	Lo	Vegetables, excluding Potatoes
72128510	Turnip greens, canned, low sodium	Lo	Vegetables, excluding Potatoes
72128520	Turnip greens, canned, low sodium	Lo	Vegetables, excluding Potatoes
72130199	Watercress, cooked, NS as to fat a	Lo	Vegetables, excluding Potatoes
72130200	Watercress, cooked, fat not added	Lo	Vegetables, excluding Potatoes
72130201	Watercress, cooked	Lo	Vegetables, excluding Potatoes
72132199	Bitter melon leaves, horseradish lea	Lo	Vegetables, excluding Potatoes
72132200	Bitter melon leaves, horseradish lea	Lo	Vegetables, excluding Potatoes
72132201	Bitter melon, horseradish, jute, or ra	Lo	Vegetables, excluding Potatoes
72133199	Sweet potato leaves, squash leaves	Lo	Vegetables, excluding Potatoes
72133200	Sweet potato leaves, squash leaves	Lo	Vegetables, excluding Potatoes
72133201	Sweet potato, squash, pumpkin, ch	Lo	Vegetables, excluding Potatoes
72201190	Broccoli, cooked, from restaurant	Lo	Vegetables, excluding Potatoes

72201200	Broccoli, cooked, NS as to form, NS	Lo	Vegetables, excluding Potatoes
72201201	Broccoli, cooked, from fresh, NS as	Lo	Vegetables, excluding Potatoes
72201202	Broccoli, cooked, from frozen, NS a	Lo	Vegetables, excluding Potatoes
72201210	Broccoli, cooked, NS as to form, fat	Lo	Vegetables, excluding Potatoes
72201211	Broccoli, fresh, cooked, no added fa	Lo	Vegetables, excluding Potatoes
72201212	Broccoli, frozen, cooked, no added	Lo	Vegetables, excluding Potatoes
72201213	Broccoli, cooked, NS as to form, ma	Lo	Vegetables, excluding Potatoes
72201214	Broccoli, cooked, NS as to form, ma	Lo	Vegetables, excluding Potatoes
72201220	Broccoli, NS as to form, cooked	Lo	Vegetables, excluding Potatoes
72201221	Broccoli, fresh, cooked, fat added, I	Lo	Vegetables, excluding Potatoes
72201222	Broccoli, frozen, cooked, fat added,	Lo	Vegetables, excluding Potatoes
72201223	Broccoli, fresh, cooked with oil	Lo	Vegetables, excluding Potatoes
72201224	Broccoli, fresh, cooked with butter c	Lo	Vegetables, excluding Potatoes
72201225	Broccoli, cooked, from fresh, made	Lo	Vegetables, excluding Potatoes
72201226	Broccoli, frozen, cooked with oil	Lo	Vegetables, excluding Potatoes
72201227	Broccoli, frozen, cooked with butter	Lo	Vegetables, excluding Potatoes
72201228	Broccoli, cooked, from frozen, made	Lo	Vegetables, excluding Potatoes
72201230	Broccoli, cooked, NS as to form, wi	Lo	Vegetables, excluding Potatoes
72201231	Broccoli, cooked, from fresh, with c	Lo	Vegetables, excluding Potatoes
72201232	Broccoli, cooked, from frozen, with	Lo	Vegetables, excluding Potatoes
72201240	Broccoli, cooked, NS as to form, wi	Lo	Vegetables, excluding Potatoes
72201241	Broccoli, cooked, from fresh, with m	Lo	Vegetables, excluding Potatoes
72201242	Broccoli, cooked, from frozen, with	Lo	Vegetables, excluding Potatoes
72201250	Broccoli, cooked, NS as to form, wi	Lo	Vegetables, excluding Potatoes
72201251	Broccoli, cooked, from fresh, with c	Lo	Vegetables, excluding Potatoes
72201252	Broccoli, cooked, from frozen, with	Lo	Vegetables, excluding Potatoes
72202010	Broccoli casserole with noodles	Lo	Mixed Dishes - Bean/Vegetable-based
72202020	Broccoli casserole with rice	Lo	Mixed Dishes - Bean/Vegetable-based
72202030	Fried broccoli	Lo	Vegetables, excluding Potatoes
72203010	Broccoli, chinese, cooked, NS as to	Lo	Vegetables, excluding Potatoes
72203050	Broccoli, chinese cooked, from fres	Lo	Vegetables, excluding Potatoes
72203070	Broccoli, Chinese, cooked	Lo	Vegetables, excluding Potatoes
72203080	Broccoli, chinese, cooked, NS as to	Lo	Vegetables, excluding Potatoes
72203120	Broccoli, chinese, cooked, from fres	Lo	Vegetables, excluding Potatoes
72302000	Broccoli soup, prepared with milk, h	Lo	Mixed Dishes - Soups
72302020	Broccoli soup, prepared with water,	Lo	Mixed Dishes - Soups
72302100	Broccoli cheese soup, prepared wit	Lo	Mixed Dishes - Soups
72306000	Watercress broth with shrimp	Lo	Mixed Dishes - Soups
72307000	Spinach soup	Lo	Mixed Dishes - Soups
72308000	Dark-green leafy vegetable soup wi	Lo	Mixed Dishes - Soups
72308500	Dark-green leafy vegetable soup, m	Lo	Mixed Dishes - Soups
73102190	Carrots, cooked, from restaurant	Lo	Vegetables, excluding Potatoes
73102200	Carrots, cooked, NS as to form, NS	Lo	Vegetables, excluding Potatoes
73102201	Carrots, cooked, from fresh, NS as	Lo	Vegetables, excluding Potatoes
73102202	Carrots, cooked, from frozen, NS as	Lo	Vegetables, excluding Potatoes
73102203	Carrots, cooked, from canned, NS a	Lo	Vegetables, excluding Potatoes
73102210	Carrots, cooked, NS as to form, fat	Lo	Vegetables, excluding Potatoes
73102211	Carrots, fresh, cooked, no added fa	Lo	Vegetables, excluding Potatoes
73102212	Carrots, frozen, cooked, no added f	Lo	Vegetables, excluding Potatoes
73102213	Carrots, canned, cooked, no added	Lo	Vegetables, excluding Potatoes
73102214	Carrots, cooked, NS as to form, ma	Lo	Vegetables, excluding Potatoes

73102215	Carrots, cooked, NS as to form, ma	Lo	Vegetables, excluding Potatoes
73102217	Carrots, fresh, cooked with oil	Lo	Vegetables, excluding Potatoes
73102218	Carrots, fresh, cooked with butter o	Lo	Vegetables, excluding Potatoes
73102219	Carrots, cooked, from fresh, made	Lo	Vegetables, excluding Potatoes
73102220	Carrots, NS as to form, cooked	Lo	Vegetables, excluding Potatoes
73102221	Carrots, fresh, cooked, fat added, N	Lo	Vegetables, excluding Potatoes
73102222	Carrots, frozen, cooked, fat added,	Lo	Vegetables, excluding Potatoes
73102223	Carrots, canned, cooked, fat added	Lo	Vegetables, excluding Potatoes
73102224	Carrots, frozen, cooked with oil	Lo	Vegetables, excluding Potatoes
73102225	Carrots, frozen, cooked with butter	Lo	Vegetables, excluding Potatoes
73102226	Carrots, cooked, from frozen, made	Lo	Vegetables, excluding Potatoes
73102228	Carrots, canned, cooked with butter	Lo	Vegetables, excluding Potatoes
73102229	Carrots, cooked, from canned, mad	Lo	Vegetables, excluding Potatoes
73102230	Carrots, cooked, NS as to form, cre	Lo	Mixed Dishes - Bean/Vegetable-based
73102231	Carrots, cooked, from fresh, cream	Lo	Mixed Dishes - Bean/Vegetable-based
73102233	Carrots, cooked, from canned, crea	Lo	Mixed Dishes - Bean/Vegetable-based
73102240	Carrots, cooked, NS as to form, gla	Lo	Vegetables, excluding Potatoes
73102241	Carrots, glazed, cooked	Lo	Vegetables, excluding Potatoes
73102242	Carrots, cooked, from frozen, glaze	Lo	Vegetables, excluding Potatoes
73102243	Carrots, cooked, from canned, glaz	Lo	Vegetables, excluding Potatoes
73102251	Carrots, cooked, from fresh, with ch	Lo	Mixed Dishes - Bean/Vegetable-based
73102252	Carrots, cooked, from frozen, with c	Lo	Mixed Dishes - Bean/Vegetable-based
73103000	Carrots, canned, low sodium, NS as	Lo	Vegetables, excluding Potatoes
73103010	Carrots, canned, reduced sodium, c	Lo	Vegetables, excluding Potatoes
73103020	Carrots, canned, reduced sodium, c	Lo	Vegetables, excluding Potatoes
73103022	Carrots, canned, reduced sodium, c	Lo	Vegetables, excluding Potatoes
73103023	Carrots, canned, low sodium, made	Lo	Vegetables, excluding Potatoes
73111030	Peas and carrots, NS as to form, cr	Lo	Mixed Dishes - Bean/Vegetable-based
73111031	Peas and carrots, from fresh, crean	Lo	Mixed Dishes - Bean/Vegetable-based
73111032	Peas and carrots, from frozen, crea	Lo	Mixed Dishes - Bean/Vegetable-based
73111033	Peas and carrots, from canned, cre	Lo	Mixed Dishes - Bean/Vegetable-based
73111200	Peas and carrots, cooked, NS as to	Lo	Vegetables, excluding Potatoes
73111201	Peas and carrots, cooked, from fres	Lo	Vegetables, excluding Potatoes
73111202	Peas and carrots, cooked, from froz	Lo	Vegetables, excluding Potatoes
73111203	Peas and carrots, cooked, from car	Lo	Vegetables, excluding Potatoes
73111210	Peas and carrots, cooked, NS as to	Lo	Vegetables, excluding Potatoes
73111211	Peas and carrots, fresh, cooked, no	Lo	Vegetables, excluding Potatoes
73111212	Peas and carrots, frozen, cooked, n	Lo	Vegetables, excluding Potatoes
73111213	Peas and carrots, canned, cooked,	Lo	Vegetables, excluding Potatoes
73111220	Peas and carrots, cooked, NS as to	Lo	Vegetables, excluding Potatoes
73111221	Peas and carrots, fresh, cooked, fa	Lo	Vegetables, excluding Potatoes
73111222	Peas and carrots, frozen, cooked, f	Lo	Vegetables, excluding Potatoes
73111223	Peas and carrots, canned, cooked,	Lo	Vegetables, excluding Potatoes
73111224	Peas and carrots, cooked, NS as to	Lo	Vegetables, excluding Potatoes
73111227	Peas and carrots, cooked, from fres	Lo	Vegetables, excluding Potatoes
73111228	Peas and carrots, cooked, from fres	Lo	Vegetables, excluding Potatoes
73111231	Peas and carrots, cooked, from froz	Lo	Vegetables, excluding Potatoes
73111232	Peas and carrots, cooked, from froz	Lo	Vegetables, excluding Potatoes
73111234	Peas and carrots, cooked, from car	Lo	Vegetables, excluding Potatoes
73111235	Peas and carrots, cooked, from car	Lo	Vegetables, excluding Potatoes
73111260	Peas and carrots, canned, low sodi	Lo	Vegetables, excluding Potatoes

73111270	Peas and carrots, canned, low sodi	Lo	Vegetables, excluding Potatoes
73111400	Carrots in tomato sauce	Lo	Mixed Dishes - Bean/Vegetable-based
73201000	Pumpkin, cooked, NS as to form, N	Lo	Vegetables, excluding Potatoes
73201001	Pumpkin, cooked, from fresh, NS a	Lo	Vegetables, excluding Potatoes
73201010	Pumpkin, cooked, NS as to form, fa	Lo	Vegetables, excluding Potatoes
73201011	Pumpkin, cooked, from fresh, fat no	Lo	Vegetables, excluding Potatoes
73201012	Pumpkin, cooked, from frozen, fat n	Lo	Vegetables, excluding Potatoes
73201013	Pumpkin, canned, cooked	Lo	Vegetables, excluding Potatoes
73201020	Pumpkin, cooked	Lo	Vegetables, excluding Potatoes
73201021	Pumpkin, cooked, from fresh, fat ad	Lo	Vegetables, excluding Potatoes
73201024	Pumpkin, cooked, NS as to form, m	Lo	Vegetables, excluding Potatoes
73201027	Pumpkin, cooked, from fresh, made	Lo	Vegetables, excluding Potatoes
73210010	Calabaza, cooked	Lo	Vegetables, excluding Potatoes
73210110	Pumpkin fritters, Puerto Rican style	Lo	Mixed Dishes - Bean/Vegetable-based
73211110	Sweet potato and pumpkin cassero	Lo	Mixed Dishes - Bean/Vegetable-based
73301000	Squash, winter type, mashed, NS a	Lo	Vegetables, excluding Potatoes
73301010	Squash, winter type, mashed, no fa	Lo	Vegetables, excluding Potatoes
73301020	Squash, winter type, mashed, fat ad	Lo	Vegetables, excluding Potatoes
73302010	Winter squash, raw	Lo	Vegetables, excluding Potatoes
73303000	Squash, winter type, baked, NS as	Lo	Vegetables, excluding Potatoes
73303010	Winter squash, cooked, no added f	Lo	Vegetables, excluding Potatoes
73303020	Winter squash, cooked, fat added	Lo	Vegetables, excluding Potatoes
73303021	Squash, winter type, baked, no sug	Lo	Vegetables, excluding Potatoes
73303022	Squash, winter type, baked, no sug	Lo	Vegetables, excluding Potatoes
73303040	Squash, winter type, baked, no fat a	Lo	Vegetables, excluding Potatoes
73304010	Squash fritter or cake	Lo	Vegetables, excluding Potatoes
73305010	Squash, winter, baked with cheese	Lo	Mixed Dishes - Bean/Vegetable-based
73305020	Squash, winter, souffle	Lo	Mixed Dishes - Bean/Vegetable-based
73401000	Sweet potato, NFS	Lo	Vegetables, excluding Potatoes
73402000	Sweet potato, baked, peel eaten, N	Lo	Vegetables, excluding Potatoes
73402010	Sweet potato, baked, peel eaten, no	Lo	Vegetables, excluding Potatoes
73402020	Sweet potato, baked, peel eaten, fa	Lo	Vegetables, excluding Potatoes
73402021	Sweet potato, baked, peel eaten, m	Lo	Vegetables, excluding Potatoes
73402022	Sweet potato, baked, peel eaten, m	Lo	Vegetables, excluding Potatoes
73402023	Sweet potato, baked, peel eaten, m	Lo	Vegetables, excluding Potatoes
73403000	Sweet potato, baked, peel not eater	Lo	Vegetables, excluding Potatoes
73403010	Sweet potato, baked, peel not eater	Lo	Vegetables, excluding Potatoes
73403020	Sweet potato, baked, peel not eater	Lo	Vegetables, excluding Potatoes
73403021	Sweet potato, baked, peel not eater	Lo	Vegetables, excluding Potatoes
73403022	Sweet potato, baked, peel not eater	Lo	Vegetables, excluding Potatoes
73403023	Sweet potato, baked, peel not eater	Lo	Vegetables, excluding Potatoes
73405000	Sweet potato, boiled, NS as to fat	Lo	Vegetables, excluding Potatoes
73405010	Sweet potato, boiled, no added fat	Lo	Vegetables, excluding Potatoes
73405020	Sweet potato, boiled, fat added, NS	Lo	Vegetables, excluding Potatoes
73405021	Sweet potato, boiled, made with oil	Lo	Vegetables, excluding Potatoes
73405022	Sweet potato, boiled, made with bu	Lo	Vegetables, excluding Potatoes
73405023	Sweet potato, boiled, made with ma	Lo	Vegetables, excluding Potatoes
73405110	Sweet potato, boiled with peel, peel	Lo	Vegetables, excluding Potatoes
73405120	Sweet potato, boiled with peel, peel	Lo	Vegetables, excluding Potatoes
73406000	Sweet potato, candied	Lo	Vegetables, excluding Potatoes
73406010	Sweet potato with fruit	Lo	Vegetables, excluding Potatoes

73407000	Sweet potato, canned, NS as to fat	Lo	Vegetables, excluding Potatoes
73407010	Sweet potato, canned without syrup	Lo	Vegetables, excluding Potatoes
73407020	Sweet potato, canned in syrup	Lo	Vegetables, excluding Potatoes
73407030	Sweet potato, canned in syrup, with	Lo	Vegetables, excluding Potatoes
73407050	Sweet potato, canned, no added fat	Lo	Vegetables, excluding Potatoes
73407060	Sweet potato, canned, fat added	Lo	Vegetables, excluding Potatoes
73409000	Sweet potato, casserole or mashed	Lo	Vegetables, excluding Potatoes
73410110	Sweet potato, fried	Lo	Vegetables, excluding Potatoes
73410200	Sweet potato fries, NFS	Lo	Vegetables, excluding Potatoes
73410210	Sweet potato chips	Lo	Savory Snacks
73410300	Sweet potato fries, NS as to fresh o	Lo	Vegetables, excluding Potatoes
73410310	Sweet potato fries, frozen, fried	Lo	Vegetables, excluding Potatoes
73410320	Sweet potato fries, frozen, baked	Lo	Vegetables, excluding Potatoes
73410330	Sweet potato fries, from fresh, fried	Lo	Vegetables, excluding Potatoes
73410340	Sweet potato fries, from fresh, bake	Lo	Vegetables, excluding Potatoes
73410400	Sweet potato fries, fast food / restau	Lo	Vegetables, excluding Potatoes
73410500	Sweet potato fries, school	Lo	Vegetables, excluding Potatoes
73420000	Sweet potato tots, NFS	Lo	Vegetables, excluding Potatoes
73420020	Sweet potato tots, from frozen, bak	Lo	Vegetables, excluding Potatoes
73420200	Sweet potato tots, school	Lo	Vegetables, excluding Potatoes
73421000	Sweet potato, yellow, Puerto Rican,	Lo	Vegetables, excluding Potatoes
73501000	Carrot soup, cream of, prepared wit	Lo	Mixed Dishes - Soups
73501010	Carrot with rice soup, cream of, pre	Lo	Mixed Dishes - Soups
73502000	Squash, winter type, soup, home re	Lo	Mixed Dishes - Soups
74201000	Tomatoes, NS as to form, cooked	Lo	Vegetables, excluding Potatoes
74201001	Tomatoes, fresh, cooked	Lo	Vegetables, excluding Potatoes
74201003	Tomatoes, canned, cooked	Lo	Vegetables, excluding Potatoes
74202010	Tomatoes, NS as to form, broiled	Lo	Vegetables, excluding Potatoes
74202011	Tomatoes, from fresh, broiled	Lo	Vegetables, excluding Potatoes
74202050	Tomatoes, red, NS as to form, fried	Lo	Vegetables, excluding Potatoes
74202051	Tomatoes, red, from fresh, fried	Lo	Vegetables, excluding Potatoes
74203010	Tomatoes, scalloped	Lo	Mixed Dishes - Bean/Vegetable-based
74204010	Tomatoes, NS as to form, stewed	Lo	Vegetables, excluding Potatoes
74204011	Tomatoes, from fresh, stewed	Lo	Vegetables, excluding Potatoes
74204013	Tomatoes, from canned, stewed	Lo	Vegetables, excluding Potatoes
74204500	Tomatoes, canned, reduced sodium	Lo	Vegetables, excluding Potatoes
74205010	Fried green tomatoes	Lo	Vegetables, excluding Potatoes
74205011	Tomatoes, green, cooked, from fres	Lo	Vegetables, excluding Potatoes
74206000	Sun-dried tomatoes	Lo	Vegetables, excluding Potatoes
74301100	Tomato juice, 100%	Lo	100% Juice
74301150	Tomato juice, 100%, low sodium	Lo	100% Juice
74302000	Tomato juice cocktail	Lo	100% Juice
74303000	Tomato and vegetable juice, 100%	Lo	100% Juice
74303100	Tomato and vegetable juice, 100%,	Lo	100% Juice
74304000	Tomato juice with clam or beef juice	Lo	100% Juice
74401010	Ketchup	Lo	Condiments and Sauces
74401110	Ketchup, reduced sodium	Lo	Condiments and Sauces
74402010	Tomato chili sauce	Lo	Condiments and Sauces
74402150	Salsa, red, commercially-prepared	Lo	Condiments and Sauces
74402210	Taco sauce	Lo	Condiments and Sauces
74402250	Enchilada sauce, red	Lo	Condiments and Sauces

74402260	Enchilada sauce, green	Lo	Condiments and Sauces
74403010	Tomato sauce	Lo	Condiments and Sauces
74403110	Tomato paste	Lo	Vegetables, excluding Potatoes
74404010	Spaghetti sauce	Lo	Condiments and Sauces
74404020	Spaghetti sauce with added vegeta	Lo	Condiments and Sauces
74404030	Spaghetti sauce with meat, canned	Lo	Condiments and Sauces
74404050	Spaghetti sauce, reduced sodium	Lo	Condiments and Sauces
74404060	Spaghetti sauce, fat free	Lo	Condiments and Sauces
74404090	Vodka sauce with tomatoes and cre	Lo	Condiments and Sauces
74406010	Barbecue sauce	Lo	Condiments and Sauces
74406050	Barbecue sauce, reduced sodium	Lo	Condiments and Sauces
74406060	Buffalo sauce	Lo	Condiments and Sauces
74406100	Steak sauce	Lo	Condiments and Sauces
74406500	Cocktail sauce	Lo	Condiments and Sauces
74410110	Puerto Rican seasoning with ham	Lo	Condiments and Sauces
74415110	Puerto Rican seasoning with ham a	Lo	Condiments and Sauces
74420110	Puerto Rican seasoning without ha	Lo	Condiments and Sauces
74501010	Tomato aspic	Lo	Vegetables, excluding Potatoes
74502010	Tomato and lima beans, cooked, fa	Lo	Vegetables, excluding Potatoes
74503010	Tomato and corn, cooked, fat not a	Lo	Vegetables, excluding Potatoes
74504000	Tomato and okra, cooked, NS as to	Lo	Vegetables, excluding Potatoes
74504010	Tomato and okra, cooked, fat not a	Lo	Vegetables, excluding Potatoes
74504020	Tomato and okra, cooked, fat adde	Lo	Vegetables, excluding Potatoes
74504021	Tomato and okra, cooked, made wi	Lo	Vegetables, excluding Potatoes
74504100	Tomato and onion, cooked, NS as t	Lo	Vegetables, excluding Potatoes
74504110	Tomato and onion, cooked, fat not	Lo	Vegetables, excluding Potatoes
74504120	Tomato and onion, cooked, fat add	Lo	Vegetables, excluding Potatoes
74504121	Tomato and onion, cooked, made w	Lo	Vegetables, excluding Potatoes
74504122	Tomato and onion, cooked, made w	Lo	Vegetables, excluding Potatoes
74504150	Tomato and celery, cooked, fat not	Lo	Vegetables, excluding Potatoes
74505000	Tomato with corn and okra, cooked	Lo	Vegetables, excluding Potatoes
74505010	Tomato with corn and okra, cooked	Lo	Vegetables, excluding Potatoes
74505020	Tomato with corn and okra, cooked	Lo	Vegetables, excluding Potatoes
74505022	Tomato with corn and okra, cooked	Lo	Vegetables, excluding Potatoes
74601000	Tomato soup, NFS	Lo	Mixed Dishes - Soups
74601010	Tomato soup, cream of, prepared w	Lo	Mixed Dishes - Soups
74602010	Tomato soup, prepared with water,	Lo	Mixed Dishes - Soups
74602030	Tomato soup, canned, undiluted	Lo	Mixed Dishes - Soups
74602050	Tomato soup, instant type, prepare	Lo	Mixed Dishes - Soups
74602100	Tomato soup, canned, low sodium,	Lo	Mixed Dishes - Soups
74602200	Tomato soup, canned, reduced soc	Lo	Mixed Dishes - Soups
74602300	Tomato soup, canned, reduced soc	Lo	Mixed Dishes - Soups
74603010	Tomato beef soup, prepared with w	Lo	Mixed Dishes - Soups
74604010	Tomato beef noodle soup, prepared	Lo	Mixed Dishes - Soups
74604100	Tomato beef rice soup, prepared w	Lo	Mixed Dishes - Soups
74604500	Tomato noodle soup, canned, prep	Lo	Mixed Dishes - Soups
74604600	Tomato noodle soup, canned, prep	Lo	Mixed Dishes - Soups
74605010	Tomato rice soup, prepared with wa	Lo	Mixed Dishes - Soups
74606010	Tomato vegetable soup, prepared w	Lo	Mixed Dishes - Soups
74606020	Tomato vegetable soup with noodle	Lo	Mixed Dishes - Soups
74701000	Tomato sandwich	Lo	Mixed Dishes - Sandwiches (single code)

75111500	Garlic, raw	Lo	Condiments and Sauces
75132000	Mixed vegetable juice	Lo	100% Juice
75144100	Lettuce, wilted, with bacon dressing	Lo	Mixed Dishes - Bean/Vegetable-based
75200100	Vegetables, NS as to type, cooked,	Lo	Vegetables, excluding Potatoes
75200110	Vegetables, NS as to type, cooked,	Lo	Vegetables, excluding Potatoes
75200120	Vegetables, NS as to type, cooked,	Lo	Vegetables, excluding Potatoes
75200121	Vegetables, NS as to type, cooked,	Lo	Vegetables, excluding Potatoes
75200600	Algae, dried	Lo	Other
75200700	Aloe vera juice drink	Lo	100% Juice
75201000	Artichoke, cooked, NS as to form, N	Lo	Vegetables, excluding Potatoes
75201010	Artichoke, cooked, NS as to form, f	Lo	Vegetables, excluding Potatoes
75201011	Artichoke, fresh, cooked, no added	Lo	Vegetables, excluding Potatoes
75201012	Artichoke, frozen, cooked, no added	Lo	Vegetables, excluding Potatoes
75201013	Artichoke, canned, cooked, no adde	Lo	Vegetables, excluding Potatoes
75201020	Artichokes, NS as to form, cooked	Lo	Vegetables, excluding Potatoes
75201021	Artichoke, fresh, cooked, fat added	Lo	Vegetables, excluding Potatoes
75201023	Artichoke, canned, cooked, fat adde	Lo	Vegetables, excluding Potatoes
75201030	Artichoke salad in oil	Lo	Mixed Dishes - Bean/Vegetable-based
75201034	Artichoke, cooked, from fresh, mad	Lo	Vegetables, excluding Potatoes
75201040	Artichoke, cooked, from frozen, ma	Lo	Vegetables, excluding Potatoes
75202000	Asparagus, cooked, NS as to form,	Lo	Vegetables, excluding Potatoes
75202001	Asparagus, cooked, from fresh, NS	Lo	Vegetables, excluding Potatoes
75202010	Asparagus, cooked, NS as to form,	Lo	Vegetables, excluding Potatoes
75202011	Asparagus, fresh, cooked, no added	Lo	Vegetables, excluding Potatoes
75202012	Asparagus, frozen, cooked, no adde	Lo	Vegetables, excluding Potatoes
75202013	Asparagus, canned, cooked, no add	Lo	Vegetables, excluding Potatoes
75202020	Asparagus, NS as to form, cooked	Lo	Vegetables, excluding Potatoes
75202021	Asparagus, fresh, cooked, fat added	Lo	Vegetables, excluding Potatoes
75202022	Asparagus, frozen, cooked, fat adde	Lo	Vegetables, excluding Potatoes
75202023	Asparagus, canned, cooked, fat add	Lo	Vegetables, excluding Potatoes
75202024	Asparagus, cooked, NS as to form,	Lo	Vegetables, excluding Potatoes
75202025	Asparagus, cooked, NS as to form,	Lo	Vegetables, excluding Potatoes
75202026	Asparagus, cooked, NS as to form,	Lo	Vegetables, excluding Potatoes
75202027	Asparagus, fresh, cooked with oil	Lo	Vegetables, excluding Potatoes
75202028	Asparagus, fresh, cooked with butte	Lo	Vegetables, excluding Potatoes
75202029	Asparagus, cooked, from fresh, ma	Lo	Vegetables, excluding Potatoes
75202032	Asparagus, frozen, cooked with but	Lo	Vegetables, excluding Potatoes
75202035	Asparagus, canned, cooked with bu	Lo	Vegetables, excluding Potatoes
75202036	Asparagus, cooked, from canned, n	Lo	Vegetables, excluding Potatoes
75203000	Bamboo shoots, cooked, fat not add	Lo	Vegetables, excluding Potatoes
75203020	Bamboo shoots, cooked, fat added	Lo	Vegetables, excluding Potatoes
75203028	Bamboo shoots, cooked	Lo	Vegetables, excluding Potatoes
75204000	Beans, lima, immature, cooked, NS	Lo	Vegetables, excluding Potatoes
75204001	Beans, lima, immature, cooked, fro	Lo	Vegetables, excluding Potatoes
75204002	Beans, lima, immature, cooked, fro	Lo	Vegetables, excluding Potatoes
75204003	Beans, lima, immature, cooked, fro	Lo	Vegetables, excluding Potatoes
75204010	Beans, lima, immature, cooked, NS	Lo	Vegetables, excluding Potatoes
75204011	Beans, lima, immature, cooked, fro	Lo	Vegetables, excluding Potatoes
75204012	Lima beans, from frozen, no added	Lo	Vegetables, excluding Potatoes
75204013	Beans, lima, immature, cooked, fro	Lo	Vegetables, excluding Potatoes
75204020	Beans, lima, immature, cooked, NS	Lo	Vegetables, excluding Potatoes

75204021	Beans, lima, immature, cooked, fro	Lo	Vegetables, excluding Potatoes
75204022	Lima beans, from frozen, fat added	Lo	Vegetables, excluding Potatoes
75204023	Lima beans, from canned	Lo	Vegetables, excluding Potatoes
75204027	Beans, lima, immature, cooked, fro	Lo	Vegetables, excluding Potatoes
75204030	Beans, lima, immature, cooked, fro	Lo	Vegetables, excluding Potatoes
75204031	Beans, lima, immature, cooked, fro	Lo	Vegetables, excluding Potatoes
75204032	Beans, lima, immature, cooked, fro	Lo	Vegetables, excluding Potatoes
75204033	Beans, lima, immature, cooked, fro	Lo	Vegetables, excluding Potatoes
75204110	Beans, lima, immature, canned, low	Lo	Vegetables, excluding Potatoes
75204120	Beans, lima, immature, canned, low	Lo	Vegetables, excluding Potatoes
75204960	Beans, string, cooked, NS as to for	Lo	Vegetables, excluding Potatoes
75204962	Beans, string, cooked, NS as to for	Lo	Vegetables, excluding Potatoes
75204963	Beans, string, cooked, from fresh, N	Lo	Vegetables, excluding Potatoes
75204964	Beans, string, cooked, from fresh, N	Lo	Vegetables, excluding Potatoes
75204965	Beans, string, cooked, from fresh, N	Lo	Vegetables, excluding Potatoes
75204966	Beans, string, cooked, from frozen,	Lo	Vegetables, excluding Potatoes
75204967	Beans, string, cooked, from frozen,	Lo	Vegetables, excluding Potatoes
75204969	Beans, string, cooked, from canned	Lo	Vegetables, excluding Potatoes
75204970	Beans, string, cooked, from canned	Lo	Vegetables, excluding Potatoes
75204971	Beans, string, cooked, from canned	Lo	Vegetables, excluding Potatoes
75204980	Beans, string, cooked, NS as to for	Lo	Vegetables, excluding Potatoes
75204981	Beans, string, cooked, from fresh, N	Lo	Vegetables, excluding Potatoes
75204982	Beans, string, cooked, from frozen,	Lo	Vegetables, excluding Potatoes
75204983	Beans, string, cooked, from canned	Lo	Vegetables, excluding Potatoes
75204990	Beans, string, cooked, NS as to for	Lo	Vegetables, excluding Potatoes
75204991	Beans, string, cooked, from fresh, N	Lo	Vegetables, excluding Potatoes
75204992	Beans, string, cooked, from frozen,	Lo	Vegetables, excluding Potatoes
75204993	Beans, string, cooked, from canned	Lo	Vegetables, excluding Potatoes
75205000	Beans, string, cooked, NS as to for	Lo	Vegetables, excluding Potatoes
75205001	Beans, string, cooked, from fresh, N	Lo	Vegetables, excluding Potatoes
75205003	Beans, string, cooked, from canned	Lo	Vegetables, excluding Potatoes
75205005	Green beans, cooked, from restaur	Lo	Vegetables, excluding Potatoes
75205010	Beans, string, green, cooked, NS as	Lo	Vegetables, excluding Potatoes
75205011	Beans, string, green, cooked, from	Lo	Vegetables, excluding Potatoes
75205012	Beans, string, green, cooked, from	Lo	Vegetables, excluding Potatoes
75205013	Beans, string, green, cooked, from	Lo	Vegetables, excluding Potatoes
75205020	Beans, string, green, cooked, NS as	Lo	Vegetables, excluding Potatoes
75205021	Green beans, fresh, cooked, no add	Lo	Vegetables, excluding Potatoes
75205022	Green beans, frozen, cooked, no ad	Lo	Vegetables, excluding Potatoes
75205023	Green beans, canned, cooked, no a	Lo	Vegetables, excluding Potatoes
75205030	Green beans, NS as to form, cooke	Lo	Vegetables, excluding Potatoes
75205031	Green beans, fresh, cooked, fat add	Lo	Vegetables, excluding Potatoes
75205032	Green beans, frozen, cooked, fat ad	Lo	Vegetables, excluding Potatoes
75205033	Green beans, canned, cooked, fat a	Lo	Vegetables, excluding Potatoes
75205041	Beans, string, green, cooked, NS as	Lo	Vegetables, excluding Potatoes
75205042	Beans, string, green, cooked, NS as	Lo	Vegetables, excluding Potatoes
75205043	Beans, string, green, cooked, NS as	Lo	Vegetables, excluding Potatoes
75205044	Green beans, fresh, cooked with oil	Lo	Vegetables, excluding Potatoes
75205045	Green beans, fresh, cooked with bu	Lo	Vegetables, excluding Potatoes
75205046	Beans, string, green, cooked, from	Lo	Vegetables, excluding Potatoes
75205047	Green beans, frozen, cooked with c	Lo	Vegetables, excluding Potatoes

75205048	Green beans, frozen, cooked with b	Lo	Vegetables, excluding Potatoes
75205049	Beans, string, green, cooked, from	Lo	Vegetables, excluding Potatoes
75205050	Green beans, canned, cooked with	Lo	Vegetables, excluding Potatoes
75205051	Green beans, canned, cooked with	Lo	Vegetables, excluding Potatoes
75205052	Beans, string, green, cooked, from	Lo	Vegetables, excluding Potatoes
75205110	Beans, string, green, canned, low s	Lo	Vegetables, excluding Potatoes
75205120	Green beans, canned, reduced sod	Lo	Vegetables, excluding Potatoes
75205130	Green beans, canned, reduced sod	Lo	Vegetables, excluding Potatoes
75205131	Green beans, canned, reduced sod	Lo	Vegetables, excluding Potatoes
75205132	Green beans, canned, reduced sod	Lo	Vegetables, excluding Potatoes
75205133	Beans, string, green, canned, low s	Lo	Vegetables, excluding Potatoes
75205200	Fried green beans	Lo	Vegetables, excluding Potatoes
75206000	Beans, string, yellow, cooked, NS a	Lo	Vegetables, excluding Potatoes
75206003	Beans, string, yellow, cooked, from	Lo	Vegetables, excluding Potatoes
75206010	Beans, string, yellow, cooked, NS a	Lo	Vegetables, excluding Potatoes
75206011	Beans, string, yellow, cooked, from	Lo	Vegetables, excluding Potatoes
75206012	Beans, string, yellow, cooked, from	Lo	Vegetables, excluding Potatoes
75206013	Beans, string, yellow, cooked, from	Lo	Vegetables, excluding Potatoes
75206020	Yellow string beans, cooked	Lo	Vegetables, excluding Potatoes
75206021	Beans, string, yellow, cooked, from	Lo	Vegetables, excluding Potatoes
75206022	Beans, string, yellow, cooked, from	Lo	Vegetables, excluding Potatoes
75206023	Beans, string, yellow, cooked, from	Lo	Vegetables, excluding Potatoes
75206030	Beans, string, yellow, cooked, NS a	Lo	Vegetables, excluding Potatoes
75206037	Beans, string, yellow, cooked, from	Lo	Vegetables, excluding Potatoes
75207000	Bean sprouts, cooked, NS as to for	Lo	Vegetables, excluding Potatoes
75207001	Bean sprouts, cooked, from fresh, N	Lo	Vegetables, excluding Potatoes
75207010	Bean sprouts, cooked, NS as to for	Lo	Vegetables, excluding Potatoes
75207011	Bean sprouts, cooked, from fresh, f	Lo	Vegetables, excluding Potatoes
75207020	Bean sprouts, cooked, NS as to for	Lo	Vegetables, excluding Potatoes
75207021	Bean sprouts, cooked	Lo	Vegetables, excluding Potatoes
75208000	Beets, cooked, NS as to form, NS a	Lo	Vegetables, excluding Potatoes
75208001	Beets, cooked, from fresh, NS as to	Lo	Vegetables, excluding Potatoes
75208003	Beets, cooked, from canned, NS as	Lo	Vegetables, excluding Potatoes
75208010	Beets, cooked, NS as to form, fat n	Lo	Vegetables, excluding Potatoes
75208011	Beets, fresh, cooked, no added fat	Lo	Vegetables, excluding Potatoes
75208013	Beets, canned, cooked, no added fa	Lo	Vegetables, excluding Potatoes
75208020	Beets, NS as to form, cooked	Lo	Vegetables, excluding Potatoes
75208021	Beets, fresh, cooked, fat added	Lo	Vegetables, excluding Potatoes
75208023	Beets, canned, cooked, fat added	Lo	Vegetables, excluding Potatoes
75208110	Beets, canned, reduced sodium, co	Lo	Vegetables, excluding Potatoes
75208120	Beets, canned, low sodium, fat add	Lo	Vegetables, excluding Potatoes
75208124	Beets, cooked, from fresh, made w	Lo	Vegetables, excluding Potatoes
75208290	Bitter melon, cooked, NS as to fat a	Lo	Vegetables, excluding Potatoes
75208300	Bitter melon, cooked, fat not added	Lo	Vegetables, excluding Potatoes
75208310	Bitter melon, cooked	Lo	Vegetables, excluding Potatoes
75208500	Breadfruit, cooked, fat not added in	Lo	Vegetables, excluding Potatoes
75208520	Breadfruit, fried	Lo	Vegetables, excluding Potatoes
75208710	Broccoflower, cooked, fat not added	Lo	Vegetables, excluding Potatoes
75208720	Broccoflower, cooked	Lo	Vegetables, excluding Potatoes
75209000	Brussels sprouts, cooked, NS as to	Lo	Vegetables, excluding Potatoes
75209001	Brussels sprouts, cooked, from fres	Lo	Vegetables, excluding Potatoes

75209002	Brussels sprouts, cooked, from froz	Lo	Vegetables, excluding Potatoes
75209010	Brussels sprouts, cooked, NS as to	Lo	Vegetables, excluding Potatoes
75209011	Brussels sprouts, fresh, cooked, no	Lo	Vegetables, excluding Potatoes
75209012	Brussels sprouts, frozen, cooked, n	Lo	Vegetables, excluding Potatoes
75209020	Brussels sprouts, NS as to form, co	Lo	Vegetables, excluding Potatoes
75209021	Brussels sprouts, fresh, cooked, fat	Lo	Vegetables, excluding Potatoes
75209022	Brussels sprouts, frozen, cooked, fa	Lo	Vegetables, excluding Potatoes
75209030	Brussels sprouts, cooked, NS as to	Lo	Vegetables, excluding Potatoes
75209031	Brussels sprouts, cooked, NS as to	Lo	Vegetables, excluding Potatoes
75209032	Brussels sprouts, cooked, NS as to	Lo	Vegetables, excluding Potatoes
75209040	Brussels sprouts, cooked, from fres	Lo	Vegetables, excluding Potatoes
75209041	Brussels sprouts, cooked, from fres	Lo	Vegetables, excluding Potatoes
75209050	Brussels sprouts, cooked, from froz	Lo	Vegetables, excluding Potatoes
75209051	Brussels sprouts, cooked, from froz	Lo	Vegetables, excluding Potatoes
75209052	Brussels sprouts, cooked, from froz	Lo	Vegetables, excluding Potatoes
75210000	Cabbage, Chinese, cooked, NS as	Lo	Vegetables, excluding Potatoes
75210010	Cabbage, Chinese, cooked, no add	Lo	Vegetables, excluding Potatoes
75210020	Cabbage, Chinese, cooked, fat add	Lo	Vegetables, excluding Potatoes
75210021	Cabbage, Chinese, cooked, made v	Lo	Vegetables, excluding Potatoes
75210022	Cabbage, Chinese, cooked, made v	Lo	Vegetables, excluding Potatoes
75211010	Cabbage, green, cooked, NS as to	Lo	Vegetables, excluding Potatoes
75211020	Cabbage, green, cooked, no added	Lo	Vegetables, excluding Potatoes
75211030	Cabbage, green, cooked, fat added	Lo	Vegetables, excluding Potatoes
75211031	Cabbage, green, cooked with oil	Lo	Vegetables, excluding Potatoes
75211032	Cabbage, green, cooked with butte	Lo	Vegetables, excluding Potatoes
75211033	Cabbage, green, cooked, made wit	Lo	Vegetables, excluding Potatoes
75212000	Cabbage, red, cooked, NS as to fat	Lo	Vegetables, excluding Potatoes
75212010	Cabbage, red, cooked, fat not adde	Lo	Vegetables, excluding Potatoes
75212020	Cabbage, red, cooked	Lo	Vegetables, excluding Potatoes
75212021	Cabbage, red, cooked, made with c	Lo	Vegetables, excluding Potatoes
75213010	Cabbage, savoy, cooked, fat not ad	Lo	Vegetables, excluding Potatoes
75213100	Cactus, cooked, NS as to fat added	Lo	Vegetables, excluding Potatoes
75213110	Cactus, cooked, no added fat	Lo	Vegetables, excluding Potatoes
75213120	Cactus, cooked, fat added	Lo	Vegetables, excluding Potatoes
75213121	Cactus, cooked, made with oil	Lo	Vegetables, excluding Potatoes
75214000	Cauliflower, cooked, NS as to form,	Lo	Vegetables, excluding Potatoes
75214001	Cauliflower, cooked, from fresh, NS	Lo	Vegetables, excluding Potatoes
75214002	Cauliflower, cooked, from frozen, N	Lo	Vegetables, excluding Potatoes
75214010	Cauliflower, cooked, NS as to form,	Lo	Vegetables, excluding Potatoes
75214011	Cauliflower, fresh, cooked, no adde	Lo	Vegetables, excluding Potatoes
75214012	Cauliflower, frozen, cooked, no add	Lo	Vegetables, excluding Potatoes
75214013	Cauliflower, cooked, from canned, f	Lo	Vegetables, excluding Potatoes
75214020	Cauliflower, NS as to form, cooked	Lo	Vegetables, excluding Potatoes
75214021	Cauliflower, fresh, cooked, fat adde	Lo	Vegetables, excluding Potatoes
75214022	Cauliflower, frozen, cooked, fat add	Lo	Vegetables, excluding Potatoes
75214023	Cauliflower, cooked, from canned, f	Lo	Vegetables, excluding Potatoes
75214024	Cauliflower, cooked, NS as to form,	Lo	Vegetables, excluding Potatoes
75214025	Cauliflower, cooked, NS as to form,	Lo	Vegetables, excluding Potatoes
75214027	Cauliflower, fresh, cooked with oil	Lo	Vegetables, excluding Potatoes
75214028	Cauliflower, fresh, cooked with butt	Lo	Vegetables, excluding Potatoes
75214029	Cauliflower, cooked, from fresh, ma	Lo	Vegetables, excluding Potatoes

75214030	Cauliflower, frozen, cooked with oil	Lo	Vegetables, excluding Potatoes
75214031	Cauliflower, frozen, cooked with but	Lo	Vegetables, excluding Potatoes
75214032	Cauliflower, cooked, from frozen, m	Lo	Vegetables, excluding Potatoes
75215000	Celery, cooked, NS as to fat added	Lo	Vegetables, excluding Potatoes
75215010	Celery, cooked, fat not added in coo	Lo	Vegetables, excluding Potatoes
75215020	Celery, cooked	Lo	Vegetables, excluding Potatoes
75215030	Celery, cooked, made with oil	Lo	Vegetables, excluding Potatoes
75215040	Celery, cooked, made with butter	Lo	Vegetables, excluding Potatoes
75215100	Fennel bulb, cooked, NS as to fat a	Lo	Vegetables, excluding Potatoes
75215120	Fennel bulb, cooked	Lo	Vegetables, excluding Potatoes
75215121	Fennel bulb, cooked, made with oil	Lo	Vegetables, excluding Potatoes
75215500	Chives, dried or dehydrated	Lo	Other
75215509	Christophine, cooked, NS as to fat a	Lo	Vegetables, excluding Potatoes
75215510	Christophine, cooked, fat not added	Lo	Vegetables, excluding Potatoes
75215511	Christophine, cooked	Lo	Vegetables, excluding Potatoes
75215990	Corn, cooked, from restaurant	Lo	Vegetables, excluding Potatoes
75216000	Corn, cooked, NS as to form, NS as	Lo	Vegetables, excluding Potatoes
75216001	Corn, cooked, from fresh, NS as to	Lo	Vegetables, excluding Potatoes
75216002	Corn, cooked, from frozen, NS as to	Lo	Vegetables, excluding Potatoes
75216003	Corn, cooked, from canned, NS as	Lo	Vegetables, excluding Potatoes
75216010	Corn, cooked, NS as to form, NS as	Lo	Vegetables, excluding Potatoes
75216011	Corn, cooked, from fresh, NS as to	Lo	Vegetables, excluding Potatoes
75216012	Corn, cooked, from frozen, NS as to	Lo	Vegetables, excluding Potatoes
75216013	Corn, cooked, from canned, NS as	Lo	Vegetables, excluding Potatoes
75216020	Corn, cooked, NS as to form, NS as	Lo	Vegetables, excluding Potatoes
75216021	Corn, cooked, from fresh, NS as to	Lo	Vegetables, excluding Potatoes
75216022	Corn, cooked, from frozen, NS as to	Lo	Vegetables, excluding Potatoes
75216023	Corn, cooked, from canned, NS as	Lo	Vegetables, excluding Potatoes
75216024	Corn, cooked, NS as to form, NS as	Lo	Vegetables, excluding Potatoes
75216025	Corn, cooked, NS as to form, NS as	Lo	Vegetables, excluding Potatoes
75216026	Corn, cooked, NS as to form, NS as	Lo	Vegetables, excluding Potatoes
75216027	Corn, cooked, from fresh, NS as to	Lo	Vegetables, excluding Potatoes
75216028	Corn, cooked, from fresh, NS as to	Lo	Vegetables, excluding Potatoes
75216033	Corn, cooked, from frozen, NS as to	Lo	Vegetables, excluding Potatoes
75216035	Corn, cooked, from canned, NS as	Lo	Vegetables, excluding Potatoes
75216036	Corn, cooked, from canned, NS as	Lo	Vegetables, excluding Potatoes
75216050	Corn, NS as to form, NS as to color	Lo	Vegetables, excluding Potatoes
75216053	Corn, from canned, NS as to color,	Lo	Vegetables, excluding Potatoes
75216070	Corn, dried, cooked	Lo	Vegetables, excluding Potatoes
75216100	Corn, yellow, cooked, NS as to form	Lo	Vegetables, excluding Potatoes
75216101	Corn, yellow, cooked, from fresh, N	Lo	Vegetables, excluding Potatoes
75216102	Corn, yellow, cooked, from frozen, N	Lo	Vegetables, excluding Potatoes
75216103	Corn, yellow, cooked, from canned,	Lo	Vegetables, excluding Potatoes
75216110	Corn, yellow, cooked, NS as to form	Lo	Vegetables, excluding Potatoes
75216111	Corn, fresh, cooked, no added fat	Lo	Vegetables, excluding Potatoes
75216112	Corn, frozen, cooked, no added fat	Lo	Vegetables, excluding Potatoes
75216113	Corn, canned, cooked, no added fa	Lo	Vegetables, excluding Potatoes
75216120	Corn, NS as to form, cooked	Lo	Vegetables, excluding Potatoes
75216121	Corn, fresh, cooked, fat added, NS	Lo	Vegetables, excluding Potatoes
75216122	Corn, frozen, cooked, fat added, NS	Lo	Vegetables, excluding Potatoes
75216123	Corn, canned, cooked, fat added, N	Lo	Vegetables, excluding Potatoes

75216131	Corn, yellow, cooked, NS as to forn	Lo	Vegetables, excluding Potatoes
75216132	Corn, yellow, cooked, NS as to forn	Lo	Vegetables, excluding Potatoes
75216133	Corn, yellow, cooked, NS as to forn	Lo	Vegetables, excluding Potatoes
75216134	Corn, fresh, cooked with oil	Lo	Vegetables, excluding Potatoes
75216135	Corn, fresh, cooked with butter or m	Lo	Vegetables, excluding Potatoes
75216136	Corn, yellow, cooked, from fresh, m	Lo	Vegetables, excluding Potatoes
75216137	Corn, frozen, cooked with oil	Lo	Vegetables, excluding Potatoes
75216138	Corn, frozen, cooked with butter or	Lo	Vegetables, excluding Potatoes
75216139	Corn, yellow, cooked, from frozen, r	Lo	Vegetables, excluding Potatoes
75216141	Corn, canned, cooked with oil	Lo	Vegetables, excluding Potatoes
75216142	Corn, canned, cooked with butter or	Lo	Vegetables, excluding Potatoes
75216143	Corn, yellow, cooked, from canned,	Lo	Vegetables, excluding Potatoes
75216150	Corn, yellow, NS as to form, cream	Lo	Vegetables, excluding Potatoes
75216153	Corn, creamed	Lo	Vegetables, excluding Potatoes
75216160	Corn, yellow and white, cooked, NS	Lo	Vegetables, excluding Potatoes
75216161	Corn, yellow and white, cooked, fro	Lo	Vegetables, excluding Potatoes
75216162	Corn, yellow and white, cooked, fro	Lo	Vegetables, excluding Potatoes
75216163	Corn, yellow and white, cooked, fro	Lo	Vegetables, excluding Potatoes
75216170	Corn, yellow and white, cooked, NS	Lo	Vegetables, excluding Potatoes
75216171	Corn, yellow and white, cooked, fro	Lo	Vegetables, excluding Potatoes
75216172	Corn, yellow and white, cooked, fro	Lo	Vegetables, excluding Potatoes
75216173	Corn, yellow and white, cooked, fro	Lo	Vegetables, excluding Potatoes
75216177	Corn, yellow and white, cooked, fro	Lo	Vegetables, excluding Potatoes
75216178	Corn, yellow and white, cooked, fro	Lo	Vegetables, excluding Potatoes
75216179	Corn, yellow and white, cooked, fro	Lo	Vegetables, excluding Potatoes
75216180	Corn, yellow and white, cooked, NS	Lo	Vegetables, excluding Potatoes
75216181	Corn, yellow and white, cooked, fro	Lo	Vegetables, excluding Potatoes
75216182	Corn, yellow and white, cooked, fro	Lo	Vegetables, excluding Potatoes
75216183	Corn, yellow and white, cooked, fro	Lo	Vegetables, excluding Potatoes
75216188	Corn, yellow and white, cooked, fro	Lo	Vegetables, excluding Potatoes
75216190	Corn, yellow, NS as to form, cream	Lo	Vegetables, excluding Potatoes
75216193	Corn, yellow, from canned, cream s	Lo	Vegetables, excluding Potatoes
75216200	Corn, white, cooked, NS as to form	Lo	Vegetables, excluding Potatoes
75216201	Corn, white, cooked, from fresh, NS	Lo	Vegetables, excluding Potatoes
75216202	Corn, white, cooked, from frozen, N	Lo	Vegetables, excluding Potatoes
75216210	Corn, white, cooked, NS as to form	Lo	Vegetables, excluding Potatoes
75216211	Corn, white, cooked, from fresh, fat	Lo	Vegetables, excluding Potatoes
75216212	Corn, white, cooked, from frozen, fa	Lo	Vegetables, excluding Potatoes
75216213	Corn, white, cooked, from canned,	Lo	Vegetables, excluding Potatoes
75216220	Corn, white, cooked, NS as to form	Lo	Vegetables, excluding Potatoes
75216221	Corn, white, cooked, from fresh, fat	Lo	Vegetables, excluding Potatoes
75216222	Corn, white, cooked, from frozen, fa	Lo	Vegetables, excluding Potatoes
75216223	Corn, white, cooked, from canned,	Lo	Vegetables, excluding Potatoes
75216224	Corn, white, cooked, NS as to form	Lo	Vegetables, excluding Potatoes
75216226	Corn, white, cooked, NS as to form	Lo	Vegetables, excluding Potatoes
75216227	Corn, white, cooked, from fresh, ma	Lo	Vegetables, excluding Potatoes
75216228	Corn, white, cooked, from fresh, ma	Lo	Vegetables, excluding Potatoes
75216229	Corn, white, cooked, from fresh, ma	Lo	Vegetables, excluding Potatoes
75216232	Corn, white, cooked, from frozen, m	Lo	Vegetables, excluding Potatoes
75216233	Corn, white, cooked, from frozen, m	Lo	Vegetables, excluding Potatoes
75216250	Corn, white, NS as to form, cream s	Lo	Vegetables, excluding Potatoes

75216253	Corn, white, from canned, cream st	Lo	Vegetables, excluding Potatoes
75216300	Corn, yellow, canned, low sodium, N	Lo	Vegetables, excluding Potatoes
75216310	Corn, canned, reduced sodium, coo	Lo	Vegetables, excluding Potatoes
75216320	Corn, canned, reduced sodium, coo	Lo	Vegetables, excluding Potatoes
75216322	Corn, canned, reduced sodium, coo	Lo	Vegetables, excluding Potatoes
75216323	Corn, yellow, canned, low sodium, r	Lo	Vegetables, excluding Potatoes
75216700	Cucumber, cooked, NS as to fat ad	Lo	Vegetables, excluding Potatoes
75216710	Cucumber, cooked, fat not added ir	Lo	Vegetables, excluding Potatoes
75216720	Cucumber, cooked	Lo	Vegetables, excluding Potatoes
75216721	Cucumber, cooked, made with oil	Lo	Vegetables, excluding Potatoes
75216722	Cucumber, cooked, made with butte	Lo	Vegetables, excluding Potatoes
75217000	Eggplant, cooked, NS as to fat adde	Lo	Vegetables, excluding Potatoes
75217010	Eggplant, cooked, no added fat	Lo	Vegetables, excluding Potatoes
75217020	Eggplant, cooked, fat added	Lo	Vegetables, excluding Potatoes
75217021	Eggplant, cooked, made with oil	Lo	Vegetables, excluding Potatoes
75217022	Eggplant, cooked, made with butter	Lo	Vegetables, excluding Potatoes
75217300	Flowers or blossoms of sesbania, s	Lo	Vegetables, excluding Potatoes
75217301	Flowers or blossoms of sesbania, s	Lo	Vegetables, excluding Potatoes
75217400	Garlic, cooked	Lo	Condiments and Sauces
75217490	Hominy, cooked, NS as to fat addec	Lo	Cooked Cereals
75217500	Hominy, cooked, fat not added in co	Lo	Cooked Cereals
75217520	Hominy, cooked	Lo	Cooked Cereals
75218010	Kohlrabi, cooked, fat not added in c	Lo	Vegetables, excluding Potatoes
75218011	Kohlrabi, cooked	Lo	Vegetables, excluding Potatoes
75218400	Leek, cooked	Lo	Vegetables, excluding Potatoes
75218499	Lotus root, cooked, NS as to fat adc	Lo	Vegetables, excluding Potatoes
75218500	Lotus root, cooked, fat not added in	Lo	Vegetables, excluding Potatoes
75218501	Lotus root, cooked	Lo	Vegetables, excluding Potatoes
75219000	Mushrooms, cooked, NS as to form	Lo	Vegetables, excluding Potatoes
75219001	Mushrooms, cooked, from fresh, NS	Lo	Vegetables, excluding Potatoes
75219002	Mushrooms, cooked, from frozen, N	Lo	Vegetables, excluding Potatoes
75219003	Mushrooms, cooked, from canned,	Lo	Vegetables, excluding Potatoes
75219010	Mushrooms, cooked, NS as to form	Lo	Vegetables, excluding Potatoes
75219011	Mushrooms, fresh, cooked, no adde	Lo	Vegetables, excluding Potatoes
75219012	Mushrooms, cooked, from frozen, fa	Lo	Vegetables, excluding Potatoes
75219013	Mushrooms, cooked, from canned,	Lo	Vegetables, excluding Potatoes
75219020	Mushrooms, NS as to form, cooked	Lo	Vegetables, excluding Potatoes
75219021	Mushrooms, fresh, cooked, fat adde	Lo	Vegetables, excluding Potatoes
75219022	Mushrooms, cooked, from frozen, fa	Lo	Vegetables, excluding Potatoes
75219023	Mushrooms, canned, cooked	Lo	Vegetables, excluding Potatoes
75219030	Mushrooms, cooked, NS as to form	Lo	Vegetables, excluding Potatoes
75219031	Mushrooms, cooked, NS as to form	Lo	Vegetables, excluding Potatoes
75219033	Mushrooms, fresh, cooked with oil	Lo	Vegetables, excluding Potatoes
75219034	Mushrooms, fresh, cooked with butt	Lo	Vegetables, excluding Potatoes
75219035	Mushrooms, cooked, from fresh, m	Lo	Vegetables, excluding Potatoes
75219036	Mushrooms, cooked, from frozen, n	Lo	Vegetables, excluding Potatoes
75219038	Mushrooms, cooked, from frozen, n	Lo	Vegetables, excluding Potatoes
75219100	Mushroom, Asian, cooked, from dri	Lo	Vegetables, excluding Potatoes
75220000	Okra, cooked, NS as to form, NS as	Lo	Vegetables, excluding Potatoes
75220001	Okra, cooked, from fresh, NS as to	Lo	Vegetables, excluding Potatoes
75220002	Okra, cooked, from frozen, NS as to	Lo	Vegetables, excluding Potatoes

75220010	Okra, cooked, NS as to form, fat no	Lo	Vegetables, excluding Potatoes
75220011	Okra, fresh, cooked, no added fat	Lo	Vegetables, excluding Potatoes
75220012	Okra, frozen, cooked, no added fat	Lo	Vegetables, excluding Potatoes
75220013	Okra, cooked, from canned, fat not	Lo	Vegetables, excluding Potatoes
75220020	Okra, NS as to form, cooked	Lo	Vegetables, excluding Potatoes
75220021	Okra, fresh, cooked, fat added	Lo	Vegetables, excluding Potatoes
75220022	Okra, frozen, cooked, fat added	Lo	Vegetables, excluding Potatoes
75220023	Okra, cooked, from canned, fat add	Lo	Vegetables, excluding Potatoes
75220027	Okra, cooked, from fresh, made wit	Lo	Vegetables, excluding Potatoes
75220028	Okra, cooked, from fresh, made wit	Lo	Vegetables, excluding Potatoes
75220031	Okra, cooked, from frozen, made w	Lo	Vegetables, excluding Potatoes
75220032	Okra, cooked, from frozen, made w	Lo	Vegetables, excluding Potatoes
75220033	Okra, cooked, from frozen, made w	Lo	Vegetables, excluding Potatoes
75220049	Lettuce, cooked, NS as to fat addec	Lo	Vegetables, excluding Potatoes
75220050	Lettuce, cooked, fat not added in cc	Lo	Vegetables, excluding Potatoes
75220051	Lettuce, cooked	Lo	Vegetables, excluding Potatoes
75220100	Luffa, cooked, fat not added in cook	Lo	Vegetables, excluding Potatoes
75220101	Luffa, cooked	Lo	Vegetables, excluding Potatoes
75221000	Onions, cooked, NS as to form, NS	Lo	Vegetables, excluding Potatoes
75221001	Onions, cooked, from fresh, NS as	Lo	Vegetables, excluding Potatoes
75221002	Onions, cooked, from frozen, NS as	Lo	Vegetables, excluding Potatoes
75221010	Onions, cooked, NS as to form, fat	Lo	Vegetables, excluding Potatoes
75221011	Onions, cooked, no added fat	Lo	Vegetables, excluding Potatoes
75221012	Onions, cooked, from frozen, fat no	Lo	Vegetables, excluding Potatoes
75221014	Onions, cooked, NS as to form, ma	Lo	Vegetables, excluding Potatoes
75221015	Onions, cooked, NS as to form, ma	Lo	Vegetables, excluding Potatoes
75221017	Onions, cooked, from fresh, made w	Lo	Vegetables, excluding Potatoes
75221018	Onions, cooked, from fresh, made w	Lo	Vegetables, excluding Potatoes
75221019	Onions, cooked, from fresh, made w	Lo	Vegetables, excluding Potatoes
75221020	Onions, cooked, NS as to form, fat	Lo	Vegetables, excluding Potatoes
75221021	Onions, cooked, fat added	Lo	Vegetables, excluding Potatoes
75221022	Onions, cooked, from frozen, fat ad	Lo	Vegetables, excluding Potatoes
75221023	Onions, cooked, from frozen, made	Lo	Vegetables, excluding Potatoes
75221030	Onions, pearl, cooked	Lo	Vegetables, excluding Potatoes
75221031	Onions, pearl, cooked, from fresh	Lo	Vegetables, excluding Potatoes
75221032	Onions, pearl, cooked, from frozen	Lo	Vegetables, excluding Potatoes
75221033	Onions, pearl, cooked, from cannec	Lo	Vegetables, excluding Potatoes
75221040	Onions, green, cooked, NS as to fo	Lo	Vegetables, excluding Potatoes
75221051	Onions, green, cooked, from fresh,	Lo	Vegetables, excluding Potatoes
75221060	Onions, green, cooked, NS as to fo	Lo	Vegetables, excluding Potatoes
75221061	Onions, green, cooked	Lo	Vegetables, excluding Potatoes
75221160	Palm hearts, cooked	Lo	Vegetables, excluding Potatoes
75221210	Parsley, cooked (assume fat not ad	Lo	Other
75222000	Parsnips, cooked, NS as to fat adde	Lo	Vegetables, excluding Potatoes
75222010	Parsnips, cooked, fat not added in c	Lo	Vegetables, excluding Potatoes
75222020	Parsnips, cooked	Lo	Vegetables, excluding Potatoes
75222022	Parsnips, cooked, made with butter	Lo	Vegetables, excluding Potatoes
75223000	Peas, cowpeas, field peas, or black	Lo	Vegetables, excluding Potatoes
75223001	Peas, cowpeas, field peas, or black	Lo	Vegetables, excluding Potatoes
75223002	Peas, cowpeas, field peas, or black	Lo	Vegetables, excluding Potatoes
75223003	Peas, cowpeas, field peas, or black	Lo	Vegetables, excluding Potatoes

75223010	Peas, cowpeas, field peas, or black	Lo	Vegetables, excluding Potatoes
75223011	Peas, cowpeas, field peas, or black	Lo	Vegetables, excluding Potatoes
75223012	Peas, cowpeas, field peas, or black	Lo	Vegetables, excluding Potatoes
75223013	Peas, cowpeas, field peas, or black	Lo	Vegetables, excluding Potatoes
75223020	Peas, cowpeas, field peas, or black	Lo	Vegetables, excluding Potatoes
75223021	Peas, cowpeas, field peas, or black	Lo	Vegetables, excluding Potatoes
75223022	Blackeyed peas, from frozen	Lo	Vegetables, excluding Potatoes
75223023	Blackeyed peas, from canned	Lo	Vegetables, excluding Potatoes
75223024	Peas, cowpeas, field peas, or black	Lo	Vegetables, excluding Potatoes
75223027	Peas, cowpeas, field peas, or black	Lo	Vegetables, excluding Potatoes
75223028	Peas, cowpeas, field peas, or black	Lo	Vegetables, excluding Potatoes
75223030	Peas, cowpeas, field peas, or black	Lo	Vegetables, excluding Potatoes
75223031	Peas, cowpeas, field peas, or black	Lo	Vegetables, excluding Potatoes
75223033	Peas, cowpeas, field peas, or black	Lo	Vegetables, excluding Potatoes
75223034	Peas, cowpeas, field peas, or black	Lo	Vegetables, excluding Potatoes
75224000	Green peas, cooked, from restaura	Lo	Vegetables, excluding Potatoes
75224010	Peas, green, cooked, NS as to form	Lo	Vegetables, excluding Potatoes
75224011	Peas, green, cooked, from fresh, N	Lo	Vegetables, excluding Potatoes
75224012	Peas, green, cooked, from frozen, N	Lo	Vegetables, excluding Potatoes
75224013	Peas, green, cooked, from canned,	Lo	Vegetables, excluding Potatoes
75224020	Peas, green, cooked, NS as to form	Lo	Vegetables, excluding Potatoes
75224021	Green peas, fresh, cooked, no adde	Lo	Vegetables, excluding Potatoes
75224022	Green peas, frozen, cooked, no add	Lo	Vegetables, excluding Potatoes
75224023	Green peas, canned, cooked, no ad	Lo	Vegetables, excluding Potatoes
75224030	Green peas, NS as to form, cooked	Lo	Vegetables, excluding Potatoes
75224031	Green peas, fresh, cooked, fat adde	Lo	Vegetables, excluding Potatoes
75224032	Green peas, frozen, cooked, fat add	Lo	Vegetables, excluding Potatoes
75224033	Green peas, canned, cooked, fat ad	Lo	Vegetables, excluding Potatoes
75224040	Peas, green, cooked, NS as to form	Lo	Vegetables, excluding Potatoes
75224041	Peas, green, cooked, NS as to form	Lo	Vegetables, excluding Potatoes
75224043	Green peas, fresh, cooked with oil	Lo	Vegetables, excluding Potatoes
75224044	Green peas, fresh, cooked with butt	Lo	Vegetables, excluding Potatoes
75224046	Green peas, frozen, cooked with oil	Lo	Vegetables, excluding Potatoes
75224047	Green peas, frozen, cooked with bu	Lo	Vegetables, excluding Potatoes
75224048	Peas, green, cooked, from frozen, n	Lo	Vegetables, excluding Potatoes
75224050	Green peas, canned, cooked with b	Lo	Vegetables, excluding Potatoes
75224051	Peas, green, cooked, from canned,	Lo	Vegetables, excluding Potatoes
75224120	Green peas, canned, reduced sodiu	Lo	Vegetables, excluding Potatoes
75224130	Green peas, canned, reduced sodiu	Lo	Vegetables, excluding Potatoes
75224131	Green peas, canned, reduced sodiu	Lo	Vegetables, excluding Potatoes
75224132	Green peas, canned, reduced sodiu	Lo	Vegetables, excluding Potatoes
75224133	Peas, green, canned, low sodium, n	Lo	Vegetables, excluding Potatoes
75225010	Pigeon peas, cooked, NS as to forn	Lo	Vegetables, excluding Potatoes
75225011	Pigeon peas, cooked, from fresh, fa	Lo	Vegetables, excluding Potatoes
75225013	Pigeon peas, cooked, from canned,	Lo	Vegetables, excluding Potatoes
75225014	Pigeon peas, cooked, NS as to forn	Lo	Vegetables, excluding Potatoes
75225015	Pigeon peas, cooked, NS as to forn	Lo	Vegetables, excluding Potatoes
75226000	Peppers, green, cooked, NS as to f	Lo	Vegetables, excluding Potatoes
75226010	Peppers, green, cooked, fat not add	Lo	Vegetables, excluding Potatoes
75226020	Peppers, green, cooked	Lo	Vegetables, excluding Potatoes
75226021	Peppers, green, cooked, made with	Lo	Vegetables, excluding Potatoes

75226022	Peppers, green, cooked, made with	Lo	Vegetables, excluding Potatoes
75226023	Peppers, green, cooked, made with	Lo	Vegetables, excluding Potatoes
75226040	Peppers, red, cooked, NS as to fat	Lo	Vegetables, excluding Potatoes
75226050	Peppers, red, cooked, fat not added	Lo	Vegetables, excluding Potatoes
75226060	Peppers, red, cooked	Lo	Vegetables, excluding Potatoes
75226061	Peppers, red, cooked, made with oi	Lo	Vegetables, excluding Potatoes
75226062	Peppers, red, cooked, made with bu	Lo	Vegetables, excluding Potatoes
75226090	Peppers, hot, cooked, NS as to forr	Lo	Condiments and Sauces
75226091	Peppers, hot, cooked, from fresh, N	Lo	Condiments and Sauces
75226093	Peppers, hot, cooked, from canned	Lo	Condiments and Sauces
75226100	Peppers, hot, cooked, NS as to forr	Lo	Condiments and Sauces
75226101	Peppers, hot, cooked, from fresh, fa	Lo	Condiments and Sauces
75226102	Peppers, hot, cooked, from frozen,	Lo	Condiments and Sauces
75226103	Peppers, hot, cooked, from canned	Lo	Condiments and Sauces
75226110	Peppers, hot, cooked, NS as to forr	Lo	Condiments and Sauces
75226111	Hot peppers, cooked	Lo	Condiments and Sauces
75226112	Peppers, hot, cooked, from frozen,	Lo	Condiments and Sauces
75226113	Peppers, hot, cooked, from canned	Lo	Condiments and Sauces
75226700	Pimiento	Lo	Condiments and Sauces
75227099	Radish, daikon, cooked, NS as to fa	Lo	Vegetables, excluding Potatoes
75227100	Radish, daikon, cooked, fat not add	Lo	Vegetables, excluding Potatoes
75227110	Daikon radish, cooked	Lo	Vegetables, excluding Potatoes
75228000	Rutabaga, cooked, NS as to fat add	Lo	Vegetables, excluding Potatoes
75228010	Rutabaga, cooked, fat not added in	Lo	Vegetables, excluding Potatoes
75228020	Rutabaga, cooked	Lo	Vegetables, excluding Potatoes
75228021	Rutabaga, cooked, made with oil	Lo	Vegetables, excluding Potatoes
75228022	Rutabaga, cooked, made with butte	Lo	Vegetables, excluding Potatoes
75230010	Sauerkraut, cooked, fat not added i	Lo	Condiments and Sauces
75230020	Sauerkraut, cooked, fat added in co	Lo	Condiments and Sauces
75230100	Sauerkraut, canned, low sodium	Lo	Condiments and Sauces
75231000	Snowpea, cooked, NS as to form, N	Lo	Vegetables, excluding Potatoes
75231001	Snowpea, cooked, from fresh, NS a	Lo	Vegetables, excluding Potatoes
75231010	Snowpea, cooked, NS as to form, fa	Lo	Vegetables, excluding Potatoes
75231011	Snowpea, fresh, cooked, no added	Lo	Vegetables, excluding Potatoes
75231012	Snowpea, frozen, cooked, no added	Lo	Vegetables, excluding Potatoes
75231020	Snowpea, NS as to form, cooked	Lo	Vegetables, excluding Potatoes
75231021	Snowpea, fresh, cooked, fat added	Lo	Vegetables, excluding Potatoes
75231022	Snowpea, frozen, cooked, fat added	Lo	Vegetables, excluding Potatoes
75231025	Snowpea, cooked, NS as to form, n	Lo	Vegetables, excluding Potatoes
75231026	Snowpea, cooked, from fresh, mad	Lo	Vegetables, excluding Potatoes
75231027	Snowpea, cooked, from fresh, mad	Lo	Vegetables, excluding Potatoes
75231029	Snowpea, cooked, from frozen, ma	Lo	Vegetables, excluding Potatoes
75231031	Snowpea, cooked, from frozen, ma	Lo	Vegetables, excluding Potatoes
75232000	Seaweed, dried	Lo	Other
75232050	Seaweed, prepared with soy sauce	Lo	Mixed Dishes - Asian
75232100	Seaweed, cooked, NS as to fat add	Lo	Vegetables, excluding Potatoes
75232110	Seaweed, cooked, no added fat	Lo	Vegetables, excluding Potatoes
75232120	Seaweed, cooked, fat added	Lo	Vegetables, excluding Potatoes
75232121	Seaweed, cooked, made with oil	Lo	Vegetables, excluding Potatoes
75233000	Squash, summer, yellow or green, c	Lo	Vegetables, excluding Potatoes
75233001	Squash, summer, yellow or green, c	Lo	Vegetables, excluding Potatoes

75233002	Squash, summer, yellow or green, c	Lo	Vegetables, excluding Potatoes
75233010	Squash, summer, yellow or green, c	Lo	Vegetables, excluding Potatoes
75233011	Summer squash, yellow or green, fr	Lo	Vegetables, excluding Potatoes
75233012	Summer squash, yellow or green, fr	Lo	Vegetables, excluding Potatoes
75233013	Summer squash, yellow or green, c	Lo	Vegetables, excluding Potatoes
75233020	Summer squash, yellow or green, N	Lo	Vegetables, excluding Potatoes
75233021	Summer squash, yellow or green, fr	Lo	Vegetables, excluding Potatoes
75233022	Summer squash, yellow or green, fr	Lo	Vegetables, excluding Potatoes
75233023	Summer squash, yellow or green, c	Lo	Vegetables, excluding Potatoes
75233024	Squash, summer, yellow or green, c	Lo	Vegetables, excluding Potatoes
75233025	Squash, summer, yellow or green, c	Lo	Vegetables, excluding Potatoes
75233027	Summer squash, yellow or green, fr	Lo	Vegetables, excluding Potatoes
75233028	Summer squash, yellow or green, fr	Lo	Vegetables, excluding Potatoes
75233029	Squash, summer, yellow or green, c	Lo	Vegetables, excluding Potatoes
75233030	Summer squash, yellow or green, fr	Lo	Vegetables, excluding Potatoes
75233031	Summer squash, yellow or green, fr	Lo	Vegetables, excluding Potatoes
75233210	Squash, spaghetti, cooked, fat adde	Lo	Vegetables, excluding Potatoes
75233220	Spaghetti squash, cooked	Lo	Vegetables, excluding Potatoes
75233222	Squash, spaghetti, cooked, made w	Lo	Vegetables, excluding Potatoes
75233223	Squash, spaghetti, cooked, made w	Lo	Vegetables, excluding Potatoes
75234000	Turnip, cooked, NS as to form, NS a	Lo	Vegetables, excluding Potatoes
75234001	Turnip, cooked, from fresh, NS as t	Lo	Vegetables, excluding Potatoes
75234003	Turnip, cooked, from canned, NS a	Lo	Vegetables, excluding Potatoes
75234011	Turnip, cooked, from fresh, fat not a	Lo	Vegetables, excluding Potatoes
75234012	Turnip, cooked, from frozen, fat not	Lo	Vegetables, excluding Potatoes
75234021	Turnip, cooked	Lo	Vegetables, excluding Potatoes
75234022	Turnip, cooked, from frozen, fat add	Lo	Vegetables, excluding Potatoes
75234027	Turnip, cooked, from fresh, made w	Lo	Vegetables, excluding Potatoes
75234028	Turnip, cooked, from fresh, made w	Lo	Vegetables, excluding Potatoes
75235000	Water Chesnut	Lo	Vegetables, excluding Potatoes
75235750	Winter melon, cooked	Lo	Vegetables, excluding Potatoes
75236000	Yeast	Lo	Other
75236500	Yeast extract spread	Lo	Condiments and Sauces
75301110	Lima beans and corn, cooked, no a	Lo	Vegetables, excluding Potatoes
75301120	Lima beans and corn, cooked, fat a	Lo	Vegetables, excluding Potatoes
75301121	Beans, lima and corn, cooked, mad	Lo	Vegetables, excluding Potatoes
75301123	Beans, lima and corn, cooked, mad	Lo	Vegetables, excluding Potatoes
75302009	Beans, string, green, with tomatoes	Lo	Vegetables, excluding Potatoes
75302010	Beans, string, green, with tomatoes	Lo	Vegetables, excluding Potatoes
75302011	Beans, string, green, with tomatoes	Lo	Vegetables, excluding Potatoes
75302020	Beans, string, green, with onions, c	Lo	Vegetables, excluding Potatoes
75302040	Beans, string, green, with almonds,	Lo	Vegetables, excluding Potatoes
75302045	Beans, string, green, with almonds,	Lo	Vegetables, excluding Potatoes
75302047	Beans, string, green, with almonds,	Lo	Vegetables, excluding Potatoes
75302050	Beans, string, green, and potatoes,	Lo	Vegetables, excluding Potatoes
75302060	Beans, string, green, with pinto bea	Lo	Vegetables, excluding Potatoes
75302080	Bean salad, yellow and/or green str	Lo	Vegetables, excluding Potatoes
75302200	Beans, string, green, with onions, N	Lo	Vegetables, excluding Potatoes
75302205	Beans, string, green, with onions, c	Lo	Vegetables, excluding Potatoes
75302210	Beans, string, green, with onions, c	Lo	Vegetables, excluding Potatoes
75302211	Beans, string, green, with onions, c	Lo	Vegetables, excluding Potatoes

75302212	Beans, string, green, with onions, c	Lo	Vegetables, excluding Potatoes
75302500	Beans, string, green, and potatoes,	Lo	Vegetables, excluding Potatoes
75302505	Beans, string, green, and potatoes,	Lo	Vegetables, excluding Potatoes
75302510	Beans, string, green, and potatoes,	Lo	Vegetables, excluding Potatoes
75302511	Beans, string, green, and potatoes,	Lo	Vegetables, excluding Potatoes
75302512	Beans, string, green, and potatoes,	Lo	Vegetables, excluding Potatoes
75302513	Beans, string, green, and potatoes,	Lo	Vegetables, excluding Potatoes
75303000	Corn with peppers, red or green, co	Lo	Vegetables, excluding Potatoes
75303010	Corn with peppers, red or green, co	Lo	Vegetables, excluding Potatoes
75303020	Corn with peppers, red or green, co	Lo	Vegetables, excluding Potatoes
75303021	Corn with peppers, red or green, co	Lo	Vegetables, excluding Potatoes
75303022	Corn with peppers, red or green, co	Lo	Vegetables, excluding Potatoes
75306010	Eggplant in tomato sauce, cooked,	Lo	Mixed Dishes - Bean/Vegetable-based
75306998	Peppers and onions, cooked, no ad	Lo	Vegetables, excluding Potatoes
75306999	Green peppers and onions, cooked	Lo	Vegetables, excluding Potatoes
75307000	Peppers and onions, cooked, fat ad	Lo	Vegetables, excluding Potatoes
75307001	Green peppers and onions, cooked	Lo	Vegetables, excluding Potatoes
75307002	Green peppers and onions, cooked	Lo	Vegetables, excluding Potatoes
75307003	Green peppers and onions, cooked	Lo	Vegetables, excluding Potatoes
75310990	Classic mixed vegetables, cooked,	Lo	Vegetables, excluding Potatoes
75311000	Mixed vegetables, cooked, NS as to	Lo	Vegetables, excluding Potatoes
75311002	Mixed vegetables, cooked, from fro	Lo	Vegetables, excluding Potatoes
75311003	Mixed vegetables, cooked, from ca	Lo	Vegetables, excluding Potatoes
75311010	Mixed vegetables, cooked, NS as to	Lo	Vegetables, excluding Potatoes
75311012	Classic mixed vegetables, frozen, c	Lo	Vegetables, excluding Potatoes
75311013	Classic mixed vegetables, canned,	Lo	Vegetables, excluding Potatoes
75311020	Classic mixed vegetables, NS as to	Lo	Vegetables, excluding Potatoes
75311022	Classic mixed vegetables, frozen, c	Lo	Vegetables, excluding Potatoes
75311023	Classic mixed vegetables, canned,	Lo	Vegetables, excluding Potatoes
75311024	Mixed vegetables, cooked, NS as to	Lo	Vegetables, excluding Potatoes
75311025	Mixed vegetables, cooked, NS as to	Lo	Vegetables, excluding Potatoes
75311026	Mixed vegetables, cooked, NS as to	Lo	Vegetables, excluding Potatoes
75311027	Classic mixed vegetables, frozen, c	Lo	Vegetables, excluding Potatoes
75311028	Classic mixed vegetables, frozen, c	Lo	Vegetables, excluding Potatoes
75311029	Mixed vegetables, cooked, from fro	Lo	Vegetables, excluding Potatoes
75311030	Classic mixed vegetables, canned,	Lo	Vegetables, excluding Potatoes
75311031	Classic mixed vegetables, canned,	Lo	Vegetables, excluding Potatoes
75311032	Mixed vegetables, cooked, from ca	Lo	Vegetables, excluding Potatoes
75311110	Classic mixed vegetables, canned,	Lo	Vegetables, excluding Potatoes
75311120	Classic mixed vegetables, canned,	Lo	Vegetables, excluding Potatoes
75315000	Peas and corn, cooked, NS as to fa	Lo	Vegetables, excluding Potatoes
75315010	Peas and corn, cooked, no added f	Lo	Vegetables, excluding Potatoes
75315020	Peas and corn, cooked, fat added	Lo	Vegetables, excluding Potatoes
75315022	Peas and corn, cooked, made with	Lo	Vegetables, excluding Potatoes
75315023	Peas and corn, cooked, made with	Lo	Vegetables, excluding Potatoes
75315100	Peas and onions, cooked, NS as to	Lo	Vegetables, excluding Potatoes
75315110	Peas and onions, cooked, fat not ad	Lo	Vegetables, excluding Potatoes
75315120	Peas and onions, cooked, fat added	Lo	Vegetables, excluding Potatoes
75315200	Peas and mushrooms, cooked, NS	Lo	Vegetables, excluding Potatoes
75315210	Peas and mushrooms, cooked, fat	Lo	Vegetables, excluding Potatoes
75315215	Peas and mushrooms, cooked, fat	Lo	Vegetables, excluding Potatoes

75315216	Peas and mushrooms, cooked, ma	Lo	Vegetables, excluding Potatoes
75315300	Peas and potatoes, cooked, fat not	Lo	Vegetables, excluding Potatoes
75315305	Peas and potatoes, cooked, NS as	Lo	Vegetables, excluding Potatoes
75315310	Peas and potatoes, cooked, fat add	Lo	Vegetables, excluding Potatoes
75315999	Squash, summer, yellow or green, a	Lo	Vegetables, excluding Potatoes
75316000	Squash, summer, yellow or green, a	Lo	Vegetables, excluding Potatoes
75316010	Zucchini with tomato sauce, cooked	Lo	Mixed Dishes - Bean/Vegetable-based
75316020	Squash, summer, yellow or green, a	Lo	Vegetables, excluding Potatoes
75316022	Squash, summer, yellow or green, a	Lo	Vegetables, excluding Potatoes
75316023	Squash, summer, yellow or green, a	Lo	Vegetables, excluding Potatoes
75316024	Squash, summer, yellow or green, a	Lo	Vegetables, excluding Potatoes
75316030	Squash, summer, yellow or green, v	Lo	Vegetables, excluding Potatoes
75316031	Squash, summer, yellow or green, v	Lo	Vegetables, excluding Potatoes
75316032	Squash, summer, yellow or green, v	Lo	Vegetables, excluding Potatoes
75316050	Ratatouille	Lo	Mixed Dishes - Bean/Vegetable-based
75317000	Vegetables, stew type, cooked, NS	Lo	Vegetables, excluding Potatoes
75317010	Vegetables, stew type, cooked, fat a	Lo	Vegetables, excluding Potatoes
75317011	Vegetables, stew type, cooked, mad	Lo	Vegetables, excluding Potatoes
75317012	Vegetables, stew type, cooked, mad	Lo	Vegetables, excluding Potatoes
75317020	Vegetables, stew type, cooked, no a	Lo	Vegetables, excluding Potatoes
75330050	Broccoli and cauliflower, cooked, no	Lo	Vegetables, excluding Potatoes
75330060	Broccoli and cauliflower, cooked, fa	Lo	Vegetables, excluding Potatoes
75330080	Broccoli, cauliflower and carrots, co	Lo	Vegetables, excluding Potatoes
75330090	Broccoli, cauliflower and carrots, co	Lo	Vegetables, excluding Potatoes
75330100	Vegetable combination, including ca	Lo	Vegetables, excluding Potatoes
75330110	Vegetable combination, including ca	Lo	Vegetables, excluding Potatoes
75330120	Vegetable combination, including ca	Lo	Vegetables, excluding Potatoes
75330121	Vegetable combination, including ca	Lo	Vegetables, excluding Potatoes
75330122	Vegetable combination, including ca	Lo	Vegetables, excluding Potatoes
75330123	Vegetable combination, including ca	Lo	Vegetables, excluding Potatoes
75330130	Vegetable combination, excluding c	Lo	Vegetables, excluding Potatoes
75330140	Vegetable combination, excluding c	Lo	Vegetables, excluding Potatoes
75330150	Vegetable combination, excluding c	Lo	Vegetables, excluding Potatoes
75330151	Vegetable combination, excluding c	Lo	Vegetables, excluding Potatoes
75330152	Vegetable combination, excluding c	Lo	Vegetables, excluding Potatoes
75330153	Vegetable combination, excluding c	Lo	Vegetables, excluding Potatoes
75340000	Vegetable combinations, Asian style	Lo	Vegetables, excluding Potatoes
75340010	Asian stir fry vegetables, cooked, no	Lo	Vegetables, excluding Potatoes
75340020	Asian stir fry vegetables, cooked, fa	Lo	Vegetables, excluding Potatoes
75340021	Vegetable combinations, Asian style	Lo	Vegetables, excluding Potatoes
75340100	Vegetable combinations (broccoli, c	Lo	Vegetables, excluding Potatoes
75340110	Vegetable combinations (broccoli, c	Lo	Vegetables, excluding Potatoes
75340120	Vegetable combinations (broccoli, c	Lo	Vegetables, excluding Potatoes
75340130	Vegetable combination (green bean	Lo	Vegetables, excluding Potatoes
75340140	Vegetable combination (green bean	Lo	Vegetables, excluding Potatoes
75340150	Vegetable combination (green bean	Lo	Vegetables, excluding Potatoes
75340160	Vegetable and pasta combinations	Lo	Mixed Dishes - Bean/Vegetable-based
75340200	Jai, Monk's Food	Lo	Vegetables, excluding Potatoes
75340300	Pinacbet	Lo	Vegetables, excluding Potatoes
75365000	Vegetable mixture, dried	Lo	Other
75400500	Artichokes, stuffed	Lo	Mixed Dishes - Bean/Vegetable-based

75401010	Asparagus, NS as to form, creamed	Lo	Mixed Dishes - Bean/Vegetable-based
75401011	Asparagus, from fresh, creamed or	Lo	Mixed Dishes - Bean/Vegetable-based
75401012	Asparagus, from frozen, creamed o	Lo	Mixed Dishes - Bean/Vegetable-based
75401013	Asparagus, from canned, creamed	Lo	Mixed Dishes - Bean/Vegetable-based
75402012	Beans, lima, immature, from frozen	Lo	Mixed Dishes - Bean/Vegetable-based
75402020	Beans, lima, immature, cooked, NS	Lo	Mixed Dishes - Bean/Vegetable-based
75403010	Beans, string, green, NS as to form	Lo	Mixed Dishes - Bean/Vegetable-based
75403011	Beans, string, green, from fresh, cre	Lo	Mixed Dishes - Bean/Vegetable-based
75403012	Beans, string, green, from frozen, c	Lo	Mixed Dishes - Bean/Vegetable-based
75403013	Beans, string, green, from canned,	Lo	Mixed Dishes - Bean/Vegetable-based
75403020	Green bean casserole	Lo	Mixed Dishes - Bean/Vegetable-based
75403022	Beans, string, green, cooked, from	Lo	Mixed Dishes - Bean/Vegetable-based
75403023	Beans, string, green, cooked, from	Lo	Mixed Dishes - Bean/Vegetable-based
75403200	Green beans, cooked, Szechuan-st	Lo	Mixed Dishes - Asian
75405010	Beets with Harvard sauce	Lo	Mixed Dishes - Bean/Vegetable-based
75406011	Brussels sprouts, from fresh, cream	Lo	Mixed Dishes - Bean/Vegetable-based
75407010	Cabbage, creamed	Lo	Mixed Dishes - Bean/Vegetable-based
75409010	Cauliflower, NS as to form, creame	Lo	Mixed Dishes - Bean/Vegetable-based
75409011	Cauliflower, from fresh, creamed	Lo	Mixed Dishes - Bean/Vegetable-based
75409012	Cauliflower, from frozen, creamed	Lo	Mixed Dishes - Bean/Vegetable-based
75409020	Fried cauliflower	Lo	Vegetables, excluding Potatoes
75410010	Celery, creamed	Lo	Mixed Dishes - Bean/Vegetable-based
75410500	Chiles rellenos, cheese-filled	Lo	Mixed Dishes - Mexican
75410530	Chiles rellenos, filled with meat and	Lo	Mixed Dishes - Mexican
75410550	Stuffed jalapeno pepper	Lo	Mixed Dishes - Grain-based
75411010	Corn, scalloped or pudding	Lo	Mixed Dishes - Bean/Vegetable-based
75411020	Corn fritter	Lo	Vegetables, excluding Potatoes
75411030	Corn, cooked, NS as to form, with c	Lo	Mixed Dishes - Bean/Vegetable-based
75411031	Corn, cooked, from fresh, with crea	Lo	Mixed Dishes - Bean/Vegetable-based
75411032	Corn, cooked, from frozen, with cre	Lo	Mixed Dishes - Bean/Vegetable-based
75411033	Corn, cooked, from canned, with cr	Lo	Mixed Dishes - Bean/Vegetable-based
75412010	Fried eggplant	Lo	Vegetables, excluding Potatoes
75412030	Eggplant dip	Lo	Condiments and Sauces
75412060	Eggplant parmesan casserole, regu	Lo	Mixed Dishes - Bean/Vegetable-based
75412070	Eggplant with cheese and tomato s	Lo	Mixed Dishes - Bean/Vegetable-based
75414010	Mushrooms, NS as to form, creame	Lo	Mixed Dishes - Bean/Vegetable-based
75414011	Mushrooms, from fresh, creamed	Lo	Mixed Dishes - Bean/Vegetable-based
75414013	Mushrooms, from canned, creamed	Lo	Mixed Dishes - Bean/Vegetable-based
75414020	Mushrooms, stuffed	Lo	Mixed Dishes - Bean/Vegetable-based
75414030	Fried mushrooms	Lo	Vegetables, excluding Potatoes
75414500	Fried okra	Lo	Vegetables, excluding Potatoes
75415010	Onions, NS as to form, creamed	Lo	Mixed Dishes - Bean/Vegetable-based
75415011	Onions, from fresh, creamed	Lo	Mixed Dishes - Bean/Vegetable-based
75415020	Onion rings, NS as to form, batter-d	Lo	Vegetables, excluding Potatoes
75415021	Onion rings, from fresh, batter-dipp	Lo	Vegetables, excluding Potatoes
75415022	Fried onion rings	Lo	Vegetables, excluding Potatoes
75416500	Pea salad	Lo	Vegetables, excluding Potatoes
75417010	Peas, NS as to form, creamed	Lo	Mixed Dishes - Bean/Vegetable-based
75417011	Peas, from fresh, creamed	Lo	Mixed Dishes - Bean/Vegetable-based
75417012	Peas, from frozen, creamed	Lo	Mixed Dishes - Bean/Vegetable-based
75417013	Peas, from canned, creamed	Lo	Mixed Dishes - Bean/Vegetable-based

75417021	Peas, cooked, from fresh, with mus	Lo	Mixed Dishes - Bean/Vegetable-based
75417022	Peas, cooked, from frozen, with mu	Lo	Mixed Dishes - Bean/Vegetable-based
75417030	Peas, cooked, NS as to form, with t	Lo	Mixed Dishes - Bean/Vegetable-based
75417032	Peas, cooked, from frozen, with ton	Lo	Mixed Dishes - Bean/Vegetable-based
75418000	Squash, summer, yellow or green, l	Lo	Mixed Dishes - Bean/Vegetable-based
75418010	Fried summer squash, yellow or gre	Lo	Vegetables, excluding Potatoes
75418020	Squash, summer, casserole with to	Lo	Mixed Dishes - Bean/Vegetable-based
75418030	Squash, summer, casserole, with ri	Lo	Mixed Dishes - Bean/Vegetable-based
75418040	Squash, summer, casserole, with c	Lo	Mixed Dishes - Bean/Vegetable-based
75418050	Squash, summer, NS as to form, cr	Lo	Mixed Dishes - Bean/Vegetable-based
75418051	Squash, summer, from fresh, crean	Lo	Mixed Dishes - Bean/Vegetable-based
75418053	Squash, summer, from canned, cre	Lo	Mixed Dishes - Bean/Vegetable-based
75418060	Squash, summer, souffle	Lo	Mixed Dishes - Bean/Vegetable-based
75418101	Turnips, from fresh, creamed	Lo	Mixed Dishes - Bean/Vegetable-based
75418220	Creamed christophine, Puerto Rica	Lo	Mixed Dishes - Bean/Vegetable-based
75439010	Vegetable stew without meat	Lo	Mixed Dishes - Bean/Vegetable-based
75439500	Chow mein or chop suey, meatless	Lo	Mixed Dishes - Asian
75440100	Vegetable combination, including ca	Lo	Mixed Dishes - Asian
75440110	Vegetable combination, excluding c	Lo	Mixed Dishes - Asian
75440170	Vegetable sticks, breaded (including	Lo	Mixed Dishes - Bean/Vegetable-based
75440200	Vegetable tempura	Lo	Vegetables, excluding Potatoes
75440300	Vegetable combinations, including c	Lo	Mixed Dishes - Bean/Vegetable-based
75440310	Vegetable combinations, excluding	Lo	Mixed Dishes - Bean/Vegetable-based
75440400	Pakora	Lo	Vegetables, excluding Potatoes
75440500	Vegetable combinations, including c	Lo	Mixed Dishes - Bean/Vegetable-based
75440510	Vegetable combinations, excluding	Lo	Mixed Dishes - Bean/Vegetable-based
75440600	Vegetable curry	Lo	Mixed Dishes - Bean/Vegetable-based
75440610	Vegetable curry with rice	Lo	Mixed Dishes - Bean/Vegetable-based
75450500	Vegetable combination, including ca	Lo	Mixed Dishes - Bean/Vegetable-based
75450510	Vegetable combination, excluding c	Lo	Mixed Dishes - Bean/Vegetable-based
75450600	Vegetable combination, including ca	Lo	Mixed Dishes - Bean/Vegetable-based
75460700	Vegetable combinations, including c	Lo	Mixed Dishes - Bean/Vegetable-based
75460710	Vegetable combinations, excluding	Lo	Mixed Dishes - Bean/Vegetable-based
75460800	Vegetable combinations, including c	Lo	Mixed Dishes - Bean/Vegetable-based
75460810	Vegetable combinations, excluding	Lo	Mixed Dishes - Bean/Vegetable-based
75460900	Chow mein or chop suey, meatless	Lo	Mixed Dishes - Asian
75501010	Relish, corn	Lo	Condiments and Sauces
75503020	Relish, pickle	Lo	Condiments and Sauces
75503090	Horseradish	Lo	Condiments and Sauces
75503110	Cucumber pickles, dill, reduced salt	Lo	Condiments and Sauces
75506010	Mustard	Lo	Condiments and Sauces
75506100	Honey mustard dip	Lo	Condiments and Sauces
75510020	Olives, black	Lo	Condiments and Sauces
75510050	Olive tapenade	Lo	Condiments and Sauces
75511300	Pickles, fried	Lo	Vegetables, excluding Potatoes
75515010	Vegetable relish	Lo	Condiments and Sauces
75534550	Wasabi paste	Lo	Condiments and Sauces
75600150	Soup, cream of, NFS	Lo	Mixed Dishes - Soups
75601000	Asparagus soup, cream of, NS as t	Lo	Mixed Dishes - Soups
75601010	Asparagus soup, cream of, prepare	Lo	Mixed Dishes - Soups
75601020	Asparagus soup, cream of, prepare	Lo	Mixed Dishes - Soups

75601100	Borscht	Lo	Mixed Dishes - Soups
75601200	Cabbage soup, home recipe, canne	Lo	Mixed Dishes - Soups
75601210	Cabbage with meat soup, home rec	Lo	Mixed Dishes - Soups
75602010	Cauliflower soup, cream of, prepare	Lo	Mixed Dishes - Soups
75603000	Celery soup, cream of, NS as to ma	Lo	Mixed Dishes - Soups
75603010	Celery soup, cream of, prepared wi	Lo	Mixed Dishes - Soups
75603020	Celery soup, cream of, prepared wi	Lo	Mixed Dishes - Soups
75603030	Celery soup, cream of, canned, und	Lo	Mixed Dishes - Soups
75604010	Corn soup, cream of, prepared with	Lo	Mixed Dishes - Soups
75604020	Corn soup, cream of, prepared with	Lo	Mixed Dishes - Soups
75604510	Cucumber soup, cream of, prepare	Lo	Mixed Dishes - Soups
75605010	Leek soup, cream of, prepared with	Lo	Mixed Dishes - Soups
75607000	Mushroom soup, NFS	Lo	Mixed Dishes - Soups
75607010	Mushroom soup, cream of, prepare	Lo	Mixed Dishes - Soups
75607020	Mushroom soup, cream of, prepare	Lo	Mixed Dishes - Soups
75607030	Mushroom soup, canned, undiluted	Lo	Mixed Dishes - Soups
75607040	Mushroom soup, with meat broth, p	Lo	Mixed Dishes - Soups
75607050	Mushroom soup, cream of, low sod	Lo	Mixed Dishes - Soups
75607060	Mushroom soup, cream of, NS as to	Lo	Mixed Dishes - Soups
75607080	Mushroom with chicken soup, crear	Lo	Mixed Dishes - Soups
75607090	Mushroom soup, cream of, canned,	Lo	Mixed Dishes - Soups
75607100	Mushroom soup, cream of, canned,	Lo	Mixed Dishes - Soups
75607130	Mushroom soup, made from dry mi	Lo	Mixed Dishes - Soups
75607140	Mushroom soup, cream of, canned,	Lo	Mixed Dishes - Soups
75607150	Mushroom soup, cream of, canned,	Lo	Mixed Dishes - Soups
75608010	Onion soup, cream of, prepared wit	Lo	Mixed Dishes - Soups
75608030	Onion soup, cream of, canned, und	Lo	Mixed Dishes - Soups
75608100	Onion soup, French	Lo	Mixed Dishes - Soups
75608200	Onion soup, made from dry mix	Lo	Mixed Dishes - Soups
75609000	Pea soup, NFS	Lo	Mixed Dishes - Soups
75609010	Pea soup, prepared with milk	Lo	Mixed Dishes - Soups
75609020	Pea soup, prepared with water	Lo	Mixed Dishes - Soups
75609050	Pea soup, canned, low sodium, pre	Lo	Mixed Dishes - Soups
75611010	Vegetable soup, cream of, preparec	Lo	Mixed Dishes - Soups
75612010	Zucchini soup, cream of, prepared v	Lo	Mixed Dishes - Soups
75646010	Shav soup	Lo	Mixed Dishes - Soups
75647000	Seaweed soup	Lo	Mixed Dishes - Soups
75649010	Vegetable soup, canned, prepared	Lo	Mixed Dishes - Soups
75649020	Vegetable soup, canned, undiluted	Lo	Mixed Dishes - Soups
75649030	Vegetable soup, canned, low sodiu	Lo	Mixed Dishes - Soups
75649040	Vegetable soup, reduced sodium, c	Lo	Mixed Dishes - Soups
75649050	Vegetable soup, made from dry mix	Lo	Mixed Dishes - Soups
75649070	Vegetable soup, made from dry mix	Lo	Mixed Dishes - Soups
75649100	Vegetable soup, cream of, made fro	Lo	Mixed Dishes - Soups
75649110	Vegetable soup, home recipe	Lo	Mixed Dishes - Soups
75649150	Vegetable noodle soup, home recip	Lo	Mixed Dishes - Soups
75650990	Minestrone soup, reduced sodium,	Lo	Mixed Dishes - Soups
75651000	Minestrone soup, home recipe	Lo	Mixed Dishes - Soups
75651010	Minestrone soup, canned, preparec	Lo	Mixed Dishes - Soups
75651020	Vegetable beef soup, canned, prep	Lo	Mixed Dishes - Soups
75651030	Vegetable beef noodle soup, prepa	Lo	Mixed Dishes - Soups

75651040	Vegetable noodle soup, canned, pr	Lo	Mixed Dishes - Soups
75651050	Vegetable chicken or turkey soup, p	Lo	Mixed Dishes - Soups
75651070	Vegetable rice soup, canned, prepa	Lo	Mixed Dishes - Soups
75651080	Vegetable beef soup with rice, cann	Lo	Mixed Dishes - Soups
75651090	Vegetable chicken soup, canned, lo	Lo	Mixed Dishes - Soups
75651110	Vegetable chicken rice soup, canne	Lo	Mixed Dishes - Soups
75651120	Vegetable chicken noodle soup, pre	Lo	Mixed Dishes - Soups
75651140	Vegetable soup with chicken broth,	Lo	Mixed Dishes - Soups
75651150	Vegetable noodle soup, reduced so	Lo	Mixed Dishes - Soups
75652010	Vegetable beef soup, home recipe	Lo	Mixed Dishes - Soups
75652020	Vegetable beef soup, canned, undil	Lo	Mixed Dishes - Soups
75652030	Vegetable beef soup, canned, prep	Lo	Mixed Dishes - Soups
75652040	Vegetable beef soup with noodles c	Lo	Mixed Dishes - Soups
75652050	Vegetable beef soup with rice, hom	Lo	Mixed Dishes - Soups
75654010	Vegetarian vegetable soup, prepare	Lo	Mixed Dishes - Soups
75654020	Vegetarian vegetable soup, undilute	Lo	Mixed Dishes - Soups
75656010	Vegetable soup, Spanish style, stev	Lo	Mixed Dishes - Soups
75656020	Vegetable soup, chunky style	Lo	Mixed Dishes - Soups
75656040	Vegetable soup, with pasta, chunky	Lo	Mixed Dishes - Soups
75656060	Vegetable beef soup, chunky style	Lo	Mixed Dishes - Soups
75657000	Vegetable broth, bouillon	Lo	Mixed Dishes - Soups
76102010	Spinach, creamed, baby food, strai	Lo	Baby Foods
76102030	Broccoli, carrots and cheese, baby	Lo	Baby Foods
76201000	Carrots, baby food, NS as to straine	Lo	Baby Foods
76201010	Carrots, baby food, strained	Lo	Baby Foods
76201020	Carrots, baby food, junior	Lo	Baby Foods
76201030	Carrots, baby food, toddler	Lo	Baby Foods
76202000	Carrots and peas, baby food, strain	Lo	Baby Foods
76205000	Squash, baby food, NS as to straine	Lo	Baby Foods
76205010	Squash, baby food, strained	Lo	Baby Foods
76205020	Squash, baby food, junior	Lo	Baby Foods
76205030	Squash and corn, baby food, straine	Lo	Baby Foods
76205060	Corn and sweet potatoes, baby food	Lo	Baby Foods
76209000	Sweet potatoes, baby food, NS as t	Lo	Baby Foods
76209010	Sweet potatoes, baby food, strained	Lo	Baby Foods
76209020	Sweet potatoes, baby food, junior	Lo	Baby Foods
76401000	Beans, green string, baby food, NS	Lo	Baby Foods
76401010	Beans, green string, baby food, stra	Lo	Baby Foods
76401020	Beans, green string, baby food, juni	Lo	Baby Foods
76401060	Beans, green string, baby food, tod	Lo	Baby Foods
76402000	Green beans and potatoes, baby fo	Lo	Baby Foods
76403010	Beets, baby food, strained	Lo	Baby Foods
76405000	Corn, creamed, baby food, NS as to	Lo	Baby Foods
76405010	Corn, creamed, baby food, strained	Lo	Baby Foods
76405020	Corn, creamed, baby food, junior	Lo	Baby Foods
76407000	Mixed vegetables, garden vegetable	Lo	Baby Foods
76407010	Mixed vegetables, garden vegetable	Lo	Baby Foods
76407020	Mixed vegetables, garden vegetable	Lo	Baby Foods
76409000	Peas, baby food, NS as to strained	Lo	Baby Foods
76409010	Peas, baby food, strained	Lo	Baby Foods
76409020	Peas, baby food, junior	Lo	Baby Foods

76409030	Peas, baby food, toddler	Lo	Baby Foods
76420000	Potatoes, baby food, toddler	Lo	Baby Foods
76501000	Vegetables and rice, baby food, stra	Lo	Baby Foods
76502000	Peas and brown rice, baby food	Lo	Baby Foods
76601010	Vegetable and bacon, baby food, st	Lo	Baby Foods
76602000	Carrots and beef, baby food, straine	Lo	Baby Foods
76603010	Vegetable and beef, baby food, stra	Lo	Baby Foods
76603020	Vegetable and beef, baby food, juni	Lo	Baby Foods
76604000	Broccoli and chicken, baby food, str	Lo	Baby Foods
76604500	Sweet potatoes and chicken, baby f	Lo	Baby Foods
76605000	Vegetable and chicken, baby food,	Lo	Baby Foods
76605010	Vegetable and chicken, baby food,	Lo	Baby Foods
76605020	Vegetable and chicken, baby food,	Lo	Baby Foods
76607010	Vegetable and ham, baby food, stra	Lo	Baby Foods
76607020	Vegetable and ham, baby food, juni	Lo	Baby Foods
76607030	Potatoes with cheese and ham, bab	Lo	Baby Foods
76607100	Potatoes with cheese and broccoli,	Lo	Baby Foods
76611000	Vegetable and turkey, baby food, N	Lo	Baby Foods
76611010	Vegetable and turkey, baby food, st	Lo	Baby Foods
76611020	Vegetable and turkey, baby food, ju	Lo	Baby Foods
77121010	Fried stuffed potatoes, Puerto Rica	Lo	White Potatoes
77121110	Potato and ham fritters, Puerto Rica	Lo	White Potatoes
77141010	Potato chicken pie, Puerto Rican st	Lo	Mixed Dishes - Meat, Poultry, Seafood
77201210	Green plantain with cracklings, Pue	Lo	Vegetables, excluding Potatoes
77205110	Ripe plantain fritters, Puerto Rican s	Lo	Vegetables, excluding Potatoes
77205610	Ripe plantain meat pie, Puerto Rica	Lo	Mixed Dishes - Meat, Poultry, Seafood
77230210	Cassava Pasteles, Puerto Rican sty	Lo	Mixed Dishes - Meat, Poultry, Seafood
77250110	Stuffed tannier fritters, Puerto Rica	Lo	Mixed Dishes - Meat, Poultry, Seafood
77250710	Tannier fritters, Puerto Rican style	Lo	Mixed Dishes - Bean/Vegetable-based
77272010	Puerto Rican pasteles	Lo	Mixed Dishes - Meat, Poultry, Seafood
77316010	Stuffed cabbage, with meat, Puerto	Lo	Mixed Dishes - Meat, Poultry, Seafood
77316510	Stuffed cabbage, with meat and rice	Lo	Mixed Dishes - Meat, Poultry, Seafood
77316600	Eggplant and meat casserole	Lo	Mixed Dishes - Meat, Poultry, Seafood
77513010	Spanish stew	Lo	Mixed Dishes - Meat, Poultry, Seafood
77563010	Puerto Rican stew	Lo	Mixed Dishes - Meat, Poultry, Seafood
78101000	Vegetable and fruit juice, 100% juic	Lo	100% Juice
78101120	Fruit and vegetable smoothie, bottle	Lo	Sweetened Beverages
81100000	Table fat, NFS	Lo	Fats and Oils
81100500	Butter, NFS	Lo	Fats and Oils
81101000	Butter, stick	Lo	Fats and Oils
81101010	Butter, tub	Lo	Fats and Oils
81101100	Butter, stick, unsalted	Lo	Fats and Oils
81101110	Butter, whipped, tub, unsalted	Lo	Fats and Oils
81101500	Light butter, stick, salted	Lo	Fats and Oils
81101510	Light butter, stick, unsalted	Lo	Fats and Oils
81101520	Butter, light	Lo	Fats and Oils
81102000	Margarine, NFS	Lo	Fats and Oils
81102010	Margarine, stick	Lo	Fats and Oils
81102020	Margarine, tub	Lo	Fats and Oils
81102030	Margarine, liquid, salted	Lo	Fats and Oils
81103020	Margarine, whipped, tub, salted	Lo	Fats and Oils

81103030	Margarine, stick, unsalted	Lo	Fats and Oils
81103035	Margarine-oil blend, NFS	Lo	Fats and Oils
81103040	Margarine-oil blend, stick	Lo	Fats and Oils
81103041	Margarine-like spread, made with y	Lo	Fats and Oils
81103060	Margarine, tub, unsalted	Lo	Fats and Oils
81103080	Margarine-oil blend, tub	Lo	Fats and Oils
81103090	Butter replacement, liquid	Lo	Fats and Oils
81103100	Margarine-like spread, stick, unsalt	Lo	Fats and Oils
81103120	Margarine-like spread, tub, unsalted	Lo	Fats and Oils
81103130	Margarine like spread, whipped, tub	Lo	Fats and Oils
81103140	Margarine-like spread, tub, sweeten	Lo	Fats and Oils
81104010	Margarine-oil blend, tub, light	Lo	Fats and Oils
81104011	Margarine like spread, reduced calc	Lo	Fats and Oils
81104020	Margarine-oil blend, stick, light	Lo	Fats and Oils
81104050	Margarine like spread, reduced calc	Lo	Fats and Oils
81104070	Margarine-like spread, reduced calc	Lo	Fats and Oils
81104100	Margarine like spread, fat free, tub,	Lo	Fats and Oils
81104110	Margarine like spread, fat free, liqui	Lo	Fats and Oils
81104490	Butter-oil blend, NFS	Lo	Fats and Oils
81104500	Butter-oil blend, stick	Lo	Fats and Oils
81104510	Butter-oil blend, tub	Lo	Fats and Oils
81104550	Butter-oil blend, light	Lo	Fats and Oils
81104560	Vegetable oil-butter spread, reduce	Lo	Fats and Oils
81105010	Butter-margarine blend, stick, salte	Lo	Fats and Oils
81105020	Butter-margarine blend, tub, salted	Lo	Fats and Oils
81105030	Butter-margarine blend, stick, unsa	Lo	Fats and Oils
81105500	Butter-vegetable oil blend	Lo	Fats and Oils
81106010	Butter replacement, powder	Lo	Fats and Oils
81201000	Animal fat or drippings	Lo	Fats and Oils
81203000	Shortening, NS as to vegetable or a	Lo	Fats and Oils
81204000	Ghee, clarified butter	Lo	Fats and Oils
81301000	Garlic sauce	Lo	Condiments and Sauces
81301020	Lemon-butter sauce	Lo	Condiments and Sauces
81302010	Hollandaise sauce	Lo	Condiments and Sauces
81302020	Bernaise sauce	Lo	Condiments and Sauces
81302030	Orange sauce (for duck)	Lo	Condiments and Sauces
81302040	Sandwich spread	Lo	Fats and Oils
81302050	Tartar sauce	Lo	Fats and Oils
81302060	Horseradish sauce	Lo	Condiments and Sauces
81302070	Pesto sauce	Lo	Condiments and Sauces
81308100	Fry sauce	Lo	Condiments and Sauces
81312100	Curry sauce	Lo	Condiments and Sauces
81322000	Honey butter	Lo	Fats and Oils
81324000	Lecithin	Lo	Other
81330210	Adobo fresco	Lo	Condiments and Sauces
82101000	Vegetable oil, NFS	Lo	Fats and Oils
82101300	Almond oil	Lo	Fats and Oils
82101500	Coconut oil	Lo	Fats and Oils
82102000	Corn oil	Lo	Fats and Oils
82103500	Flaxseed oil	Lo	Fats and Oils
82104000	Olive oil	Lo	Fats and Oils

82105000	Peanut oil	Lo	Fats and Oils
82105500	Canola oil	Lo	Fats and Oils
82106000	Safflower oil	Lo	Fats and Oils
82107000	Sesame oil	Lo	Fats and Oils
82108000	Soybean oil	Lo	Fats and Oils
82108500	Sunflower oil	Lo	Fats and Oils
82108700	Walnut oil	Lo	Fats and Oils
83100100	Salad dressing, NFS, for salads	Lo	Fats and Oils
83100200	Salad dressing, NFS, for sandwiche	Lo	Fats and Oils
83101000	Blue or roquefort cheese dressing	Lo	Fats and Oils
83101500	Bacon dressing (hot)	Lo	Fats and Oils
83101600	Bacon and tomato dressing	Lo	Fats and Oils
83102000	Caesar dressing	Lo	Fats and Oils
83103000	Coleslaw dressing	Lo	Fats and Oils
83103500	Feta Cheese Dressing	Lo	Fats and Oils
83104000	French or Catalina dressing	Lo	Fats and Oils
83105000	Fruit dressing, made with fruit juice	Lo	Fats and Oils
83105100	Fruit dressing, made with honey, oil	Lo	Fats and Oils
83105500	Honey mustard dressing	Lo	Fats and Oils
83106000	Italian dressing, made with vinegar	Lo	Fats and Oils
83107000	Mayonnaise, regular	Lo	Fats and Oils
83107200	Mayonnaise, made with tofu	Lo	Fats and Oils
83108000	Vegan mayonnaise	Lo	Fats and Oils
83108100	Mayonnaise, imitation, cholesterol f	Lo	Fats and Oils
83109000	Russian dressing	Lo	Fats and Oils
83110000	Mayonnaise-type salad dressing	Lo	Fats and Oils
83110010	Mayonnaise-type salad dressing, ch	Lo	Fats and Oils
83112000	Avocado dressing	Lo	Fats and Oils
83112500	Creamy dressing	Lo	Fats and Oils
83112600	Cream cheese dressing	Lo	Fats and Oils
83112900	Milk, vinegar, and sugar dressing	Lo	Fats and Oils
83112950	Poppy seed dressing	Lo	Fats and Oils
83112960	Peppercorn Dressing	Lo	Fats and Oils
83112980	Celery seed dressing	Lo	Fats and Oils
83112990	Sesame dressing	Lo	Fats and Oils
83113000	Sweet and sour dressing	Lo	Fats and Oils
83114000	Thousand Island dressing	Lo	Fats and Oils
83115000	Yogurt dressing	Lo	Fats and Oils
83200100	Salad dressing, light, NFS	Lo	Fats and Oils
83201000	Blue or roquefort cheese dressing,	Lo	Fats and Oils
83201050	Blue or roquefort cheese dressing,	Lo	Fats and Oils
83201200	Blue or roquefort cheese dressing,	Lo	Fats and Oils
83201400	Coleslaw dressing, light	Lo	Fats and Oils
83202000	French dressing, low-calorie	Lo	Fats and Oils
83202010	French dressing, reduced calorie, fa	Lo	Fats and Oils
83202020	French or Catalina dressing, light	Lo	Fats and Oils
83203000	Caesar dressing, light	Lo	Fats and Oils
83203250	Mayonnaise-type salad dressing, fa	Lo	Fats and Oils
83204000	Mayonnaise, light	Lo	Fats and Oils
83204020	Mayonnaise, reduced calorie or diet	Lo	Fats and Oils
83204030	Mayonnaise, reduced fat, with olive	Lo	Fats and Oils

83204050	Mayonnaise-type salad dressing, lig	Lo	Fats and Oils
83204060	Mayonnaise-type salad dressing, lo	Lo	Fats and Oils
83204500	Honey mustard dressing, light	Lo	Fats and Oils
83205000	Italian dressing, low calorie	Lo	Fats and Oils
83205450	Italian dressing, light	Lo	Fats and Oils
83205500	Italian dressing, reduced calorie, fat	Lo	Fats and Oils
83206000	Russian dressing, light	Lo	Fats and Oils
83206500	Sesame dressing, light	Lo	Fats and Oils
83207000	Thousand Island dressing, light	Lo	Fats and Oils
83207100	Thousand Island dressing, reduced	Lo	Fats and Oils
83208000	Vinegar, sugar, and water dressing	Lo	Fats and Oils
83208500	Korean dressing or marinade	Lo	Fats and Oils
83210000	Creamy dressing, made with sour c	Lo	Fats and Oils
83210050	Creamy dressing made with sour cr	Lo	Fats and Oils
83210100	Creamy dressing, light	Lo	Fats and Oils
83210200	Creamy dressing, made with sour c	Lo	Fats and Oils
83210250	Creamy dressing, made with sour c	Lo	Fats and Oils
83220000	Salad dressing, low calorie, oil-free	Lo	Fats and Oils
83300100	Blue or roquefort cheese dressing,	Lo	Fats and Oils
83300200	Caesar dressing, fat free	Lo	Fats and Oils
83300300	Creamy dressing, fat free	Lo	Fats and Oils
83300400	French or Catalina dressing, fat free	Lo	Fats and Oils
83300500	Honey mustard dressing, fat free	Lo	Fats and Oils
83300600	Italian dressing, fat free	Lo	Fats and Oils
83300700	Mayonnaise, fat free	Lo	Fats and Oils
83300900	Salad dressing, fat free, NFS	Lo	Fats and Oils
83301000	Thousand Island dressing, fat free	Lo	Fats and Oils
89901000	Bacon, for use with vegetables	Lo	Other
89901002	Ham, for use with vegetables	Lo	Other
89901004	Beef, for use with vegetables	Lo	Other
89901006	Chicken, for use with vegetables	Lo	Other
89901010	Cream sauce, for use with vegetabl	Lo	Other
89901020	Cheese sauce, for use with vegetab	Lo	Other
89901030	Gravy, for use with vegetables	Lo	Other
89901040	Soy based sauce, for use with vege	Lo	Other
89901050	Tomato sauce, for use with vegetab	Lo	Other
89902000	Avocado, for use on a sandwich	Lo	Vegetables, excluding Potatoes
89902010	Cucumber, for use on a sandwich	Lo	Vegetables, excluding Potatoes
89902040	Onions, for use on a sandwich	Lo	Vegetables, excluding Potatoes
89902100	Bacon, for use on a sandwich	Lo	Other
91101000	Sugar, NFS	Lo	Sugars
91101010	Sugar, white, granulated or lump	Lo	Sugars
91101020	Sugar, white, confectioner's, powde	Lo	Sugars
91102010	Sugar, brown	Lo	Sugars
91104100	Sugar, cinnamon	Lo	Sugars
91104200	Sugar, raw	Lo	Sugars
91105010	Fructose sweetener, sugar substitu	Lo	Sugars
91106000	Sugar substitute, sugar-aspartame	Lo	Sugars
91106010	Sugar substitute and sugar blend	Lo	Sugars
91107000	Sugar substitute, sucralose, powde	Lo	Sugars
91108000	Sugar substitute, stevia, powder	Lo	Sugars

91108010	Sugar substitute, stevia, liquid	Lo	Sugars
91108020	Sugar substitute, monk fruit, powde	Lo	Sugars
91109000	Blue Agave liquid sweetener, sugar	Lo	Sugars
91200000	Sugar substitute, powder, NFS	Lo	Sugars
91200005	Sugar substitute, liquid, NFS	Lo	Sugars
91200020	Sugar substitute, saccharin-based,	Lo	Sugars
91200030	Brown sugar substitute, saccharin-b	Lo	Sugars
91200040	Sugar substitute, saccharin, powde	Lo	Sugars
91200110	Sugar substitute, saccharin, liquid	Lo	Sugars
91201010	Sugar substitute, aspartame, powde	Lo	Sugars
91300010	Syrup, NFS	Lo	Sugars
91300100	Pancake syrup	Lo	Sugars
91301020	Cane and corn pancake syrup	Lo	Sugars
91301030	Corn syrup	Lo	Sugars
91301040	Buttered blends syrup	Lo	Sugars
91301050	Blueberry syrup	Lo	Sugars
91301060	Maple syrup	Lo	Sugars
91301080	Chocolate syrup	Lo	Sugars
91301081	Chocolate syrup, light	Lo	Sugars
91301082	Chocolate syrup, thin type, sugar fre	Lo	Sugars
91301090	Sorghum syrup	Lo	Sugars
91301100	Simple syrup	Lo	Sugars
91301130	Strawberry drink syrup	Lo	Sugars
91301200	Sugar, brown, and water syrup	Lo	Sugars
91301250	Maple and corn and/or cane pancak	Lo	Sugars
91301510	Pancake syrup, light	Lo	Sugars
91302010	Honey	Lo	Sugars
91302020	Agave liquid sweetener	Lo	Sugars
91303000	Molasses	Lo	Sugars
91304010	Topping, butterscotch or caramel	Lo	Sugars
91304020	Topping, chocolate	Lo	Sugars
91304030	Topping, fruit	Lo	Sugars
91304040	Topping, marshmallow	Lo	Sugars
91304060	Topping, nuts and syrup	Lo	Sugars
91304070	Topping, peanut butter, thick, fudge	Lo	Sugars
91304090	Topping, chocolate flavored hazelnu	Lo	Sugars
91304250	Topping, milk chocolate with cereal	Lo	Sugars
91304300	Topping, chocolate, hard coating	Lo	Sugars
91305010	Icing, chocolate	Lo	Sugars
91305020	Icing, white	Lo	Sugars
91306020	Caramel dip, regular	Lo	Sugars
91306025	Caramel dip, light	Lo	Sugars
91306030	Chocolate dip	Lo	Sugars
91306040	Dessert dip	Lo	Sugars
91351010	Syrup, dietetic	Lo	Sugars
91351020	Topping, dietetic	Lo	Sugars
91361010	Sweet and sour sauce	Lo	Condiments and Sauces
91361020	Fruit sauce	Lo	Sugars
91361040	Dessert sauce	Lo	Sugars
91361050	Duck sauce	Lo	Condiments and Sauces
91361070	Plum sauce, Asian style	Lo	Condiments and Sauces

91401000	Jelly, all flavors	Lo	Sugars
91402000	Jam, preserve, all flavors	Lo	Sugars
91403000	Fruit butter, all flavors	Lo	Sugars
91404000	Marmalade, all flavors	Lo	Sugars
91405000	Jelly, sugar free, all flavors	Lo	Sugars
91405500	Jelly, reduced sugar, all flavors	Lo	Sugars
91406000	Jam, preserve, marmalade, sugar f	Lo	Sugars
91406500	Jam, preserve, marmalade, sweete	Lo	Sugars
91406600	Jam, preserve, marmalade, reduce	Lo	Sugars
91407100	Guava paste	Lo	Sugars
91407120	Sweet potato paste	Lo	Sugars
91407150	Bean paste, sweetened	Lo	Sugars
91500200	Gelatin powder, sweetened, dry	Lo	Other
91501010	Gelatin dessert	Lo	Other Desserts
91501015	Gelatin snacks	Lo	Other Desserts
91501020	Gelatin dessert with fruit	Lo	Other Desserts
91501030	Gelatin dessert with whipped cream	Lo	Other Desserts
91501040	Gelatin dessert with fruit and whipp	Lo	Other Desserts
91501050	Gelatin dessert with cream cheese	Lo	Other Desserts
91501060	Gelatin dessert with sour cream	Lo	Other Desserts
91501070	Gelatin dessert with fruit and sour c	Lo	Other Desserts
91501080	Gelatin dessert with fruit and cream	Lo	Other Desserts
91501090	Gelatin dessert with fruit, vegetable	Lo	Other Desserts
91501100	Gelatin salad with vegetables	Lo	Other Desserts
91501110	Gelatin dessert with fruit and whipp	Lo	Other Desserts
91501120	Gelatin dessert with fruit and vegeta	Lo	Other Desserts
91510100	Gelatin powder, dietetic, sweetened	Lo	Other
91511010	Gelatin dessert, sugar free	Lo	Other Desserts
91511020	Gelatin dessert, sugar free, with fru	Lo	Other Desserts
91511030	Gelatin dessert, dietetic, with whipp	Lo	Other Desserts
91511050	Gelatin dessert, dietetic, with cream	Lo	Other Desserts
91511060	Gelatin dessert, dietetic, with sour c	Lo	Other Desserts
91511070	Gelatin dessert, dietetic, with fruit a	Lo	Other Desserts
91511080	Gelatin dessert, dietetic, with fruit a	Lo	Other Desserts
91511090	Gelatin dessert, dietetic, with fruits a	Lo	Other Desserts
91511100	Gelatin salad, dietetic, with vegetab	Lo	Other Desserts
91511110	Gelatin dessert, dietetic, with fruit a	Lo	Other Desserts
91520100	Yokan	Lo	Other Desserts
91550100	Coconut cream cake, Puerto Rican	Lo	Sweet Bakery Products
91550300	Pineapple custard, Puerto Rican sty	Lo	Other Desserts
91560100	Haupia	Lo	Other Desserts
91601000	Italian Ice	Lo	Other Desserts
91610900	Popsicle, NFS	Lo	Other Desserts
91611000	Popsicle	Lo	Other Desserts
91611050	Ice pop filled with ice cream, all flav	Lo	Other Desserts
91611100	Popsicle, no sugar added	Lo	Other Desserts
91612000	Freezer pop	Lo	Other Desserts
91621000	Snow cone	Lo	Other Desserts
91700010	Candy, NFS	Lo	Candy
91700500	M&M's Almond Chocolate Candies	Lo	Candy
91701010	Almonds, chocolate covered	Lo	Candy

91701020	Almonds, sugar-coated	Lo	Candy
91701030	Almonds, yogurt-covered	Lo	Candy
91702010	Butterscotch morsels	Lo	Candy
91703010	Caramel, chocolate-flavored roll	Lo	Candy
91703020	Caramel, flavor other than chocolat	Lo	Candy
91703030	Caramel, with nuts	Lo	Candy
91703040	Caramel candy, chocolate covered	Lo	Candy
91703050	Caramel with nuts and cereal, choc	Lo	Candy
91703060	Caramel with nuts, chocolate cover	Lo	Candy
91703070	Rolo	Lo	Candy
91703080	Caramel, all flavors, sugar free	Lo	Candy
91703150	Toblerone, milk chocolate with hone	Lo	Candy
91703200	TWIX Caramel Cookie Bars	Lo	Candy
91703250	TWIX Chocolate Fudge Cookie Bar	Lo	Candy
91703300	TWIX Peanut Butter Cookie Bars	Lo	Candy
91703350	Bar None candy bar	Lo	Candy
91703400	Whatchamacallit	Lo	Candy
91703500	Nuts, carob-coated	Lo	Candy
91703600	Espresso coffee beans, chocolate-c	Lo	Candy
91705010	Milk chocolate candy, plain	Lo	Candy
91705020	Milk chocolate candy, with cereal	Lo	Candy
91705030	Kit Kat	Lo	Candy
91705040	Chocolate, milk, with nuts, not almo	Lo	Candy
91705050	Milk chocolate candy, with fruit and	Lo	Candy
91705060	Milk chocolate candy, with almonds	Lo	Candy
91705070	Chocolate, milk, with peanuts	Lo	Candy
91705090	Chocolate candy with fondant and c	Lo	Candy
91705200	Chocolate, semi-sweet morsel	Lo	Candy
91705300	Chocolate, sweet or dark	Lo	Candy
91705310	Chocolate, sweet or dark, with almo	Lo	Candy
91705400	Chocolate, white	Lo	Candy
91705410	Chocolate, white, with almonds	Lo	Candy
91705420	Chocolate, white, with cereal	Lo	Candy
91705430	Kit Kat White	Lo	Candy
91705500	Mexican chocolate, tablet	Lo	Candy
91706000	Coconut candy, chocolate covered	Lo	Candy
91706100	Coconut candy, no chocolate cover	Lo	Candy
91706400	Coconut candy, Puerto Rican style	Lo	Candy
91707000	Fondant	Lo	Candy
91707010	Fondant, chocolate covered	Lo	Candy
91708000	Fruit peel, candied	Lo	Candy
91708010	Date candy	Lo	Candy
91708020	Soft fruit confections	Lo	Candy
91708030	Fruit leather and fruit snacks candy	Lo	Candy
91708040	Fun Fruits Creme Supremes	Lo	Candy
91708070	Tamarind candy	Lo	Candy
91708100	Fruit snacks candy, with high vitami	Lo	Candy
91708150	Yogurt covered fruit snacks candy,	Lo	Candy
91709000	Gumdrops, chocolate covered	Lo	Candy
91713010	Fudge, chocolate, chocolate-coatec	Lo	Candy
91713020	Fudge, chocolate, chocolate-coatec	Lo	Candy

91713030	Fudge, chocolate	Lo	Candy
91713040	Fudge, chocolate, with nuts	Lo	Candy
91713050	Fudge, peanut butter	Lo	Candy
91713060	Fudge, peanut butter, with nuts	Lo	Candy
91713070	Fudge, vanilla	Lo	Candy
91713080	Fudge, vanilla, with nuts	Lo	Candy
91713090	Fudge, divinity	Lo	Candy
91715000	Fudge, caramel and nut, chocolate-	Lo	Candy
91715100	SNICKERS Bar	Lo	Candy
91715200	Baby Ruth	Lo	Candy
91715300	100 GRAND Bar	Lo	Candy
91716010	Halvah, plain	Lo	Candy
91718000	Honey-combed hard candy with pea	Lo	Candy
91718050	Honey-combed hard candy with pea	Lo	Candy
91718100	Butterfinger	Lo	Candy
91718110	Butterfinger Crisp	Lo	Candy
91718200	Chocolate-flavored sprinkles	Lo	Candy
91718300	Ladoo, round ball, Asian-Indian des	Lo	Candy
91721000	Licorice	Lo	Candy
91723000	Marshmallow	Lo	Candy
91723010	Marshmallow, chocolate covered	Lo	Candy
91723020	Marshmallow, candy-coated	Lo	Candy
91723050	Marshmallow, coconut-coated	Lo	Candy
91726000	Nougat, plain	Lo	Candy
91726110	Nougat, with caramel, chocolate co	Lo	Candy
91726130	MILKY WAY Bar	Lo	Candy
91726140	MILKY WAY MIDNIGHT Bar	Lo	Candy
91726150	MARS Almond Bar	Lo	Candy
91726410	Nougat, chocolate covered	Lo	Candy
91726420	3 MUSKETEERS Bar	Lo	Candy
91726425	3 Musketeers Truffle Crisp Bar	Lo	Candy
91727010	Nuts, chocolate covered, not almon	Lo	Candy
91728000	Nut roll, fudge or nougat, caramel a	Lo	Candy
91728500	Sugared pecans, sugar and egg wh	Lo	Candy
91731000	Peanuts, chocolate covered	Lo	Candy
91731010	M&M's Peanut Chocolate Candies	Lo	Candy
91731060	M&M's Peanut Butter Chocolate Ca	Lo	Candy
91731100	Peanuts, sugar-coated	Lo	Candy
91731150	Peanuts, yogurt covered	Lo	Candy
91732000	Peanut bar	Lo	Candy
91732100	Planters Peanut Bar	Lo	Candy
91733000	Peanut brittle	Lo	Candy
91733200	Peanut Bar, chocolate covered can	Lo	Candy
91734000	Peanut butter, chocolate covered	Lo	Candy
91734100	Reese's Peanut Butter Cup	Lo	Candy
91734200	Reese's Pieces	Lo	Candy
91734300	Reese's Sticks	Lo	Candy
91734400	Reese's Fast Break	Lo	Candy
91734450	Reese's Crispy Crunchy Bar	Lo	Candy
91734500	Peanut butter morsels	Lo	Candy
91735000	Pralines	Lo	Candy

91736000	Pineapple candy, Puerto Rican style	Lo	Candy
91739010	Raisins, chocolate covered	Lo	Candy
91739600	Raisins, yogurt covered	Lo	Candy
91742010	Sesame Crunch, Sahadi	Lo	Candy
91745010	Gumdrops	Lo	Candy
91745020	Hard candy	Lo	Candy
91745040	Butterscotch hard candy	Lo	Candy
91745100	Skittles	Lo	Candy
91746010	Sugar-coated chocolate discs	Lo	Candy
91746100	M&M's Milk Chocolate Candies	Lo	Candy
91746120	Sixlets	Lo	Candy
91746150	Easter egg, candy coated chocolate	Lo	Candy
91746200	M&M's Pretzel Chocolate Candies	Lo	Candy
91750000	Taffy	Lo	Candy
91760000	Toffee, plain	Lo	Candy
91760100	Toffee, chocolate covered	Lo	Candy
91760200	Toffee, chocolate-coated, with nuts	Lo	Candy
91760500	Truffles	Lo	Candy
91760700	Wax candy, liquid filled	Lo	Candy
91770000	Dietetic or low calorie candy, NFS	Lo	Candy
91770010	Dietetic or low calorie gumdrops	Lo	Candy
91770020	Dietetic or low calorie hard candy	Lo	Candy
91770030	Dietetic or low calorie candy, choco	Lo	Candy
91770050	Dietetic or low calorie mints	Lo	Candy
91780010	Snickers Marathon Energy bar	Lo	Snack/Meal Bars
91781010	Snickers Marathon Protein bar	Lo	Snack/Meal Bars
91800100	Chewing gum, NFS	Lo	Candy
91801000	Chewing gum, regular	Lo	Candy
91802000	Chewing gum, sugar free	Lo	Candy
92100000	Coffee, NS as to type	Lo	Coffee and Tea
92100500	Coffee, NS as to brewed or instant	Lo	Coffee and Tea
92101000	Coffee, brewed	Lo	Coffee and Tea
92101500	Coffee, brewed, blend of regular an	Lo	Coffee and Tea
92101600	Coffee, Turkish	Lo	Coffee and Tea
92101610	Coffee, espresso	Lo	Coffee and Tea
92101630	Coffee, espresso, decaffeinated	Lo	Coffee and Tea
92101700	Coffee, brewed, flavored	Lo	Coffee and Tea
92101800	Coffee, Cuban	Lo	Coffee and Tea
92101810	Coffee, macchiato	Lo	Coffee and Tea
92101820	Coffee, macchiato, sweetened	Lo	Coffee and Tea
92101850	Coffee, cafe con leche	Lo	Coffee and Tea
92101851	Coffee, cafe con leche, decaffeinate	Lo	Coffee and Tea
92101900	Coffee, Latte	Lo	Coffee and Tea
92101901	Coffee, Latte, nonfat	Lo	Coffee and Tea
92101903	Coffee, Latte, with non-dairy milk	Lo	Coffee and Tea
92101904	Coffee, Latte, flavored	Lo	Coffee and Tea
92101905	Coffee, Latte, nonfat, flavored	Lo	Coffee and Tea
92101906	Coffee, Latte, with non-dairy milk, fl	Lo	Coffee and Tea
92101910	Coffee, Latte, decaffeinated	Lo	Coffee and Tea
92101911	Coffee, Latte, decaffeinated, nonfat	Lo	Coffee and Tea
92101917	Coffee, Latte, decaffeinated, flavore	Lo	Coffee and Tea

92101918	Coffee, Latte, decaffeinated, nonfat	Lo	Coffee and Tea
92101920	Frozen coffee drink	Lo	Coffee and Tea
92101921	Frozen coffee drink, nonfat	Lo	Coffee and Tea
92101923	Frozen coffee drink, with non-dairy	Lo	Coffee and Tea
92101925	Frozen coffee drink, with whipped c	Lo	Coffee and Tea
92101926	Frozen coffee drink, nonfat, with wh	Lo	Coffee and Tea
92101930	Frozen coffee drink, decaffeinated	Lo	Coffee and Tea
92101931	Frozen coffee drink, decaffeinated,	Lo	Coffee and Tea
92101935	Frozen coffee drink, decaffeinated,	Lo	Coffee and Tea
92101936	Frozen coffee drink, decaffeinated,	Lo	Coffee and Tea
92101950	Coffee, Cafe Mocha	Lo	Coffee and Tea
92101955	Coffee, Cafe Mocha, nonfat	Lo	Coffee and Tea
92101960	Coffee, Cafe Mocha, with non-dairy	Lo	Coffee and Tea
92101965	Coffee, Cafe Mocha, decaffeinated	Lo	Coffee and Tea
92101970	Coffee, Cafe Mocha, decaffeinated,	Lo	Coffee and Tea
92101975	Coffee, Cafe Mocha, decaffeinated,	Lo	Coffee and Tea
92102000	Frozen mocha coffee drink	Lo	Coffee and Tea
92102010	Frozen mocha coffee drink, nonfat	Lo	Coffee and Tea
92102020	Frozen mocha coffee drink, with no	Lo	Coffee and Tea
92102030	Frozen mocha coffee drink, with wh	Lo	Coffee and Tea
92102040	Frozen mocha coffee drink, nonfat,	Lo	Coffee and Tea
92102050	Frozen mocha coffee drink, with no	Lo	Coffee and Tea
92102090	Frozen mocha coffee drink, decaffe	Lo	Coffee and Tea
92102400	Iced Coffee, brewed	Lo	Coffee and Tea
92102401	Iced Coffee, brewed, decaffeinated	Lo	Coffee and Tea
92102450	Iced Coffee, pre-lightened and pre-s	Lo	Coffee and Tea
92102500	Coffee, Iced Latte	Lo	Coffee and Tea
92102501	Coffee, Iced Latte, nonfat	Lo	Coffee and Tea
92102502	Coffee, Iced Latte, with non-dairy m	Lo	Coffee and Tea
92102503	Coffee, Iced Latte, flavored	Lo	Coffee and Tea
92102505	Coffee, Iced Latte, with non-dairy m	Lo	Coffee and Tea
92102600	Coffee, Iced Cafe Mocha	Lo	Coffee and Tea
92102602	Coffee, Iced Cafe Mocha, with non-	Lo	Coffee and Tea
92103000	Coffee, instant, reconstituted	Lo	Coffee and Tea
92104000	Coffee, instant, 50% less caffeine, r	Lo	Coffee and Tea
92105000	Coffee, liquid concentrate	Lo	Coffee and Tea
92105010	Coffee, made from liquid concentra	Lo	Coffee and Tea
92106000	Coffee, acid neutralized, from powd	Lo	Coffee and Tea
92111000	Coffee, NS as to brewed or instant,	Lo	Coffee and Tea
92111010	Coffee, brewed, decaffeinated	Lo	Coffee and Tea
92114000	Coffee, instant, decaffeinated, reco	Lo	Coffee and Tea
92121000	Coffee, instant, pre-lightened and p	Lo	Coffee and Tea
92121001	Coffee, instant, decaffeinated, pre-l	Lo	Coffee and Tea
92121010	Coffee, instant, pre-sweetened with	Lo	Coffee and Tea
92121020	Coffee, mocha, instant, pre-lightene	Lo	Coffee and Tea
92121030	Coffee, mocha, instant, pre-lightene	Lo	Coffee and Tea
92121040	Coffee, instant, pre-lightened and p	Lo	Coffee and Tea
92121041	Coffee, instant, decaffeinated, pre-l	Lo	Coffee and Tea
92121050	Coffee, mocha, instant, decaffeinate	Lo	Coffee and Tea
92130000	Coffee, pre-lightened and pre-swee	Lo	Coffee and Tea
92130001	Coffee, decaffeinated, pre-lightened	Lo	Coffee and Tea

92130005	Coffee, pre-lightened and pre-swee	Lo	Coffee and Tea
92130006	Coffee, decaffeinated, pre-lightened	Lo	Coffee and Tea
92130010	Coffee, pre-lightened	Lo	Coffee and Tea
92130011	Coffee, decaffeinated, pre-lightened	Lo	Coffee and Tea
92130020	Coffee, pre-sweetened with sugar	Lo	Coffee and Tea
92130021	Coffee, decaffeinated, pre-sweeten	Lo	Coffee and Tea
92130030	Coffee, pre-sweetened with low cal	Lo	Coffee and Tea
92130031	Coffee, decaffeinated, pre-sweeten	Lo	Coffee and Tea
92152000	Coffee and chicory, brewed	Lo	Coffee and Tea
92153100	Coffee, decaffeinated, with cereal	Lo	Coffee and Tea
92161000	Coffee, Cappuccino	Lo	Coffee and Tea
92161001	Coffee, Cappuccino, nonfat	Lo	Coffee and Tea
92161002	Coffee, Cappuccino, with non-dairy	Lo	Coffee and Tea
92161005	Cappuccino, sweetened	Lo	Coffee and Tea
92162000	Coffee, Cappuccino, decaffeinated	Lo	Coffee and Tea
92162001	Coffee, Cappuccino, decaffeinated,	Lo	Coffee and Tea
92162005	Cappuccino, decaffeinated, sweete	Lo	Coffee and Tea
92171000	Coffee, bottled/canned	Lo	Coffee and Tea
92171010	Coffee, bottled/canned, light	Lo	Coffee and Tea
92191000	Coffee, dry instant powder, NS as t	Lo	Other
92191100	Coffee, instant, not reconstituted	Lo	Other
92191200	Coffee, instant, decaffeinated, not r	Lo	Other
92191250	Coffee, dry, acid neutralized	Lo	Other
92191400	Coffee, instant, pre-sweetened with	Lo	Other
92192000	Coffee, mocha, instant, pre-lightene	Lo	Other
92192040	Coffee, mocha, instant, decaffeinat	Lo	Other
92193000	Coffee, instant, pre-lightened and p	Lo	Other
92193005	Coffee, instant, decaffeinated, pre-l	Lo	Other
92193020	Coffee, instant, pre-lightened and p	Lo	Other
92201010	Coffee substitute	Lo	Coffee and Tea
92202010	Chicory beverage	Lo	Coffee and Tea
92203000	Cereal beverage	Lo	Coffee and Tea
92203110	Cereal beverage with beet roots, fro	Lo	Coffee and Tea
92204000	Mate, sweetened beverage made fr	Lo	Coffee and Tea
92205000	Rice beverage	Lo	Dairy Drinks and Substitutes
92291300	Coffee substitute, dry powder	Lo	Other
92301000	Tea, NS as to type, unsweetened	Lo	Coffee and Tea
92301060	Tea, NS as to type, presweetened w	Lo	Coffee and Tea
92301080	Tea, NS as to type, presweetened w	Lo	Coffee and Tea
92301100	Tea, NS as to type, decaffeinated, u	Lo	Coffee and Tea
92301130	Tea, NS as to type, presweetened,	Lo	Coffee and Tea
92301160	Tea, NS as to type, decaffeinated, p	Lo	Coffee and Tea
92301180	Tea, NS as to type, decaffeinated, p	Lo	Coffee and Tea
92301190	Tea, NS as to type, decaffeinated, p	Lo	Coffee and Tea
92302000	Tea, hot, leaf, black	Lo	Coffee and Tea
92302200	Tea, leaf, presweetened with sugar	Lo	Coffee and Tea
92302300	Tea, leaf, presweetened with low ca	Lo	Coffee and Tea
92302400	Tea, leaf, presweetened, NS as to s	Lo	Coffee and Tea
92302500	Tea, hot, leaf, black, decaffeinated	Lo	Coffee and Tea
92302600	Tea, leaf, decaffeinated, presweete	Lo	Coffee and Tea
92302700	Tea, leaf, decaffeinated, presweete	Lo	Coffee and Tea

92302800	Tea, leaf, decaffeinated, presweete	Lo	Coffee and Tea
92303010	Tea, hot, leaf, green	Lo	Coffee and Tea
92303100	Tea, hot, leaf, green, decaffeinated	Lo	Coffee and Tea
92304000	Tea, made from frozen concentrate	Lo	Coffee and Tea
92304100	Tea, hot, leaf, oolong	Lo	Coffee and Tea
92305000	Tea, made from powdered instant,	Lo	Coffee and Tea
92305010	Tea, iced, instant, black, unsweeten	Lo	Coffee and Tea
92305040	Tea, iced, instant, black, pre-sweete	Lo	Coffee and Tea
92305050	Tea, iced, instant, black, decaffeina	Lo	Coffee and Tea
92305090	Tea, iced, instant, black, pre-sweete	Lo	Coffee and Tea
92305110	Tea, iced, instant, black, decaffeina	Lo	Coffee and Tea
92305180	Tea, iced, instant, black, decaffeina	Lo	Coffee and Tea
92305800	Tea, made from powdered instant,	Lo	Coffee and Tea
92305900	Tea, iced, instant, green, unsweete	Lo	Coffee and Tea
92305910	Tea, iced, instant, green, pre-sweet	Lo	Coffee and Tea
92305920	Tea, iced, instant, green, pre-sweet	Lo	Coffee and Tea
92306000	Tea, hot, herbal	Lo	Coffee and Tea
92306020	Tea, herbal, presweetened with sug	Lo	Coffee and Tea
92306030	Tea, herbal, presweetened with low	Lo	Coffee and Tea
92306040	Tea, herbal, presweetened, NS as t	Lo	Coffee and Tea
92306090	Tea, hot, hibiscus	Lo	Coffee and Tea
92306100	Corn beverage	Lo	Sweetened Beverages
92306700	Tea, hot, chamomile	Lo	Coffee and Tea
92306800	Tea, hot, chai, with milk	Lo	Coffee and Tea
92307000	Tea, iced, instant, black, unsweeten	Lo	Other
92307400	Tea, iced, instant, black, pre-sweete	Lo	Other
92307500	Iced Tea / Lemonade juice drink	Lo	Coffee and Tea
92307510	Iced Tea / Lemonade juice drink, lig	Lo	Coffee and Tea
92307520	Iced Tea / Lemonade juice drink, di	Lo	Coffee and Tea
92308000	Tea, iced, brewed, black, pre-sweet	Lo	Coffee and Tea
92308010	Tea, iced, brewed, black, pre-sweet	Lo	Coffee and Tea
92308020	Tea, iced, brewed, black, unsweete	Lo	Coffee and Tea
92308030	Tea, iced, brewed, black, decaffeina	Lo	Coffee and Tea
92308040	Tea, iced, brewed, black, decaffeina	Lo	Coffee and Tea
92308050	Tea, iced, brewed, black, decaffeina	Lo	Coffee and Tea
92308500	Tea, iced, brewed, green, pre-swee	Lo	Coffee and Tea
92308510	Tea, iced, brewed, green, pre-swee	Lo	Coffee and Tea
92308520	Tea, iced, brewed, green, unsweete	Lo	Coffee and Tea
92308530	Tea, iced, brewed, green, decaffein	Lo	Coffee and Tea
92308540	Tea, iced, brewed, green, decaffein	Lo	Coffee and Tea
92308550	Tea, iced, brewed, green, decaffein	Lo	Coffee and Tea
92309000	Tea, iced, bottled, black	Lo	Coffee and Tea
92309010	Tea, iced, bottled, black, decaffeina	Lo	Coffee and Tea
92309020	Tea, iced, bottled, black, diet	Lo	Coffee and Tea
92309030	Tea, iced, bottled, black, decaffeina	Lo	Coffee and Tea
92309040	Tea, iced, bottled, black, unsweeten	Lo	Coffee and Tea
92309050	Tea, iced, bottled, black, decaffeina	Lo	Coffee and Tea
92309500	Tea, iced, bottled, green	Lo	Coffee and Tea
92309510	Tea, iced, bottled, green, diet	Lo	Coffee and Tea
92309520	Tea, iced, bottled, green, unsweete	Lo	Coffee and Tea
92400000	Soft drink, NFS	Lo	Sweetened Beverages

92400100	Soft drink, NFS, diet	Lo	Diet Beverages
92410110	Carbonated water, sweetened	Lo	Flavored or Enhanced Water
92410210	Carbonated water, unsweetened	Lo	Flavored or Enhanced Water
92410250	Carbonated water, sweetened, with	Lo	Flavored or Enhanced Water
92410310	Soft drink, cola	Lo	Sweetened Beverages
92410315	Soft drink, cola, reduced sugar	Lo	Sweetened Beverages
92410320	Soft drink, cola, diet	Lo	Diet Beverages
92410330	Soft drink, cola-type, with higher ca	Lo	Sweetened Beverages
92410340	Soft drink, cola, decaffeinated	Lo	Sweetened Beverages
92410350	Soft drink, cola, decaffeinated, diet	Lo	Diet Beverages
92410360	Soft drink, pepper type	Lo	Sweetened Beverages
92410370	Soft drink, pepper type, diet	Lo	Diet Beverages
92410390	Soft drink, pepper type, decaffeinate	Lo	Sweetened Beverages
92410400	Soft drink, pepper type, decaffeinate	Lo	Diet Beverages
92410410	Soft drink, cream soda	Lo	Sweetened Beverages
92410420	Soft drink, cream soda, diet	Lo	Diet Beverages
92410510	Soft drink, fruit flavored, caffeine fre	Lo	Sweetened Beverages
92410520	Soft drink, fruit flavored, diet, caffei	Lo	Diet Beverages
92410550	Soft drink, fruit flavored, caffeine co	Lo	Sweetened Beverages
92410560	Soft drink, fruit flavored, caffeine co	Lo	Diet Beverages
92410610	Soft drink, ginger ale	Lo	Sweetened Beverages
92410620	Soft drink, ginger ale, diet	Lo	Diet Beverages
92410710	Soft drink, root beer	Lo	Sweetened Beverages
92410720	Soft drink, root beer, diet	Lo	Diet Beverages
92410810	Soft drink, chocolate flavored	Lo	Sweetened Beverages
92410820	Soft drink, chocolate flavored, diet	Lo	Diet Beverages
92411510	Soft drink, cola, fruit or vanilla flavo	Lo	Sweetened Beverages
92411610	Soft drink, cola, fruit or vanilla flavo	Lo	Diet Beverages
92416010	Mavi drink	Lo	Sweetened Beverages
92417010	Soft drink, ale type	Lo	Sweetened Beverages
92431000	Carbonated juice drink, NS as to ty	Lo	Sweetened Beverages
92432000	Fruit juice drink, citrus, carbonated	Lo	Sweetened Beverages
92433000	Fruit juice drink, noncitrus, carbona	Lo	Sweetened Beverages
92510120	Apple-cherry drink	Lo	Sweetened Beverages
92510150	Apple juice drink	Lo	Sweetened Beverages
92510170	Apple-cranberry-grape juice drink	Lo	Sweetened Beverages
92510200	Apple-orange-pineapple juice drink	Lo	Sweetened Beverages
92510310	Banana-orange drink	Lo	Sweetened Beverages
92510410	Black cherry drink	Lo	Sweetened Beverages
92510610	Fruit juice drink	Lo	Sweetened Beverages
92510630	Fruit juice drink, NFS	Lo	Sweetened Beverages
92510650	Tamarind drink	Lo	Sweetened Beverages
92510720	Fruit punch, made with fruit juice ar	Lo	Sweetened Beverages
92510730	Fruit punch, made with soda, fruit ju	Lo	Sweetened Beverages
92510810	Grapeade and grape drink	Lo	Sweetened Beverages
92510820	Grape juice drink	Lo	Sweetened Beverages
92510910	Grapefruit juice drink	Lo	Sweetened Beverages
92510950	Guava juice drink	Lo	Sweetened Beverages
92510955	Lemonade, fruit juice drink	Lo	Sweetened Beverages
92510960	Lemonade, fruit flavored drink	Lo	Sweetened Beverages
92511000	Lemonade, frozen concentrate, not	Lo	Sweetened Beverages

92511010	Fruit flavored drink (formerly lemon	Lo	Sweetened Beverages
92511015	Fruit flavored drink	Lo	Sweetened Beverages
92511020	Lemon-limeade	Lo	Sweetened Beverages
92511110	Limeade	Lo	Sweetened Beverages
92511200	Orange-mango juice drink	Lo	Sweetened Beverages
92511220	Orange drink	Lo	Sweetened Beverages
92511240	Orange-lemon drink	Lo	Sweetened Beverages
92511250	Fruit juice beverage, 40-50% juice,	Lo	Sweetened Beverages
92511260	Orange-cranberry juice drink	Lo	Sweetened Beverages
92511270	Orange-peach juice drink	Lo	Sweetened Beverages
92511280	Orange-grape-banana juice drink	Lo	Sweetened Beverages
92511290	Papaya juice drink	Lo	Sweetened Beverages
92511310	Pineapple-grapefruit juice drink	Lo	Sweetened Beverages
92511340	Pineapple-orange juice drink	Lo	Sweetened Beverages
92511350	Orange-raspberry juice drink	Lo	Sweetened Beverages
92511400	Raspberry-flavored drink	Lo	Sweetened Beverages
92511510	Strawberry-flavored drink	Lo	Sweetened Beverages
92512040	Frozen daiquiri mix, frozen concent	Lo	Sweetened Beverages
92512050	Frozen daiquiri mix, from frozen co	Lo	Sweetened Beverages
92512090	Pina Colada, nonalcoholic	Lo	Sweetened Beverages
92512110	Margarita mix, nonalcoholic	Lo	Sweetened Beverages
92513000	Slush frozen drink	Lo	Sweetened Beverages
92513010	Slush frozen drink, no sugar added	Lo	Diet Beverages
92520410	Fruit drink, low calorie	Lo	Sweetened Beverages
92520810	Grape drink, low calorie	Lo	Sweetened Beverages
92520910	Lemonade, low calorie	Lo	Sweetened Beverages
92530310	Cherry drink with vitamin C added	Lo	Sweetened Beverages
92530410	Fruit flavored drink, with high vitami	Lo	Sweetened Beverages
92530510	Cranberry juice drink, with high vita	Lo	Sweetened Beverages
92530520	Cranberry-apple juice drink with vita	Lo	Sweetened Beverages
92530610	Fruit juice drink, with high vitamin C	Lo	Sweetened Beverages
92530710	Grape drink with vitamin C added	Lo	Sweetened Beverages
92530810	Grapefruit juice drink with vitamin C	Lo	Sweetened Beverages
92530840	Guava juice drink with vitamin C ad	Lo	Sweetened Beverages
92530910	Lemonade with vitamin C added	Lo	Sweetened Beverages
92530950	Vegetable and fruit juice drink, with	Lo	Sweetened Beverages
92531010	Orange drink and orangeade with v	Lo	Sweetened Beverages
92531020	Orange breakfast drink, made from	Lo	Sweetened Beverages
92531030	Fruit juice drink (Sunny D)	Lo	Sweetened Beverages
92531120	Pineapple-orange juice drink with vi	Lo	Sweetened Beverages
92541010	Fruit flavored drink, powdered, reco	Lo	Sweetened Beverages
92541020	Lemonade-flavored drink, made fro	Lo	Sweetened Beverages
92541040	Lemonade-flavored drink, made fro	Lo	Sweetened Beverages
92541100	Apple cider-flavored drink, made fro	Lo	Sweetened Beverages
92542000	Fruit flavored drink, with high vitami	Lo	Sweetened Beverages
92544000	Fruit-flavored drink, made from uns	Lo	Sweetened Beverages
92550030	Fruit juice drink, with high vitamin C	Lo	Sweetened Beverages
92550035	Fruit juice drink, light	Lo	Sweetened Beverages
92550040	Fruit juice drink, diet	Lo	Sweetened Beverages
92550050	Apple-white grape juice drink, low c	Lo	Sweetened Beverages
92550110	Cranberry juice drink, with high vita	Lo	Sweetened Beverages

92550200	Grape juice drink, light	Lo	Sweetened Beverages
92550210	Cranberry-apple juice drink, low cal	Lo	Sweetened Beverages
92550300	Grapefruit juice drink, low calorie, w	Lo	Sweetened Beverages
92550350	Orange juice beverage, 40-50% juic	Lo	Sweetened Beverages
92550360	Apple juice beverage, 40-50% juice	Lo	Sweetened Beverages
92550370	Lemonade, fruit juice drink, light	Lo	Sweetened Beverages
92550380	Pomegranate juice beverage, 40-50	Lo	Sweetened Beverages
92550400	Vegetable and fruit juice drink, with	Lo	Diet Beverages
92550405	Vegetable and fruit juice drink, with	Lo	Sweetened Beverages
92550610	Fruit flavored drink, with high vitami	Lo	Diet Beverages
92550620	Fruit flavored drink, diet	Lo	Diet Beverages
92551700	Juice drink, low calorie	Lo	Diet Beverages
92552000	Fruit flavored drink, with high vitami	Lo	Diet Beverages
92552010	Fruit flavored drink, powdered, reco	Lo	Diet Beverages
92552020	Fruit juice drink, reduced sugar (Su	Lo	Sweetened Beverages
92552030	Fruit juice drink (Capri Sun)	Lo	Sweetened Beverages
92552100	Orange-cranberry juice drink, low ca	Lo	Sweetened Beverages
92553000	Fruit-flavored thirst quencher bever	Lo	Diet Beverages
92560000	Fruit-flavored thirst quencher bever	Lo	Sweetened Beverages
92560100	Gatorade Thirst Quencher sports dr	Lo	Sweetened Beverages
92560200	Powerade sports drink	Lo	Sweetened Beverages
92565000	Fruit-flavored sports drink or thirst q	Lo	Diet Beverages
92565100	Gatorade G2 thirst quencher sports	Lo	Diet Beverages
92565200	Powerade Zero sports drink, low ca	Lo	Diet Beverages
92570100	Fluid replacement, electrolyte soluti	Lo	Sweetened Beverages
92570500	Fluid replacement, 5% glucose in w	Lo	Sweetened Beverages
92582100	Fruit juice drink, with high vitamin C	Lo	Sweetened Beverages
92582110	Fruit juice drink, added calcium (Su	Lo	Sweetened Beverages
92582120	Fruit flavored drink, reduced sugar,	Lo	Sweetened Beverages
92610010	Horchata beverage, made with almo	Lo	Sweetened Beverages
92610020	Horchata beverage, made with wate	Lo	Sweetened Beverages
92610030	Horchata beverage, made with milk	Lo	Sweetened Beverages
92610110	Coconut beverage, Puerto Rican	Lo	Sweetened Beverages
92611010	Oatmeal beverage with water	Lo	Sweetened Beverages
92611100	Oatmeal beverage with milk	Lo	Sweetened Beverages
92611510	Horchata beverage, made with rice	Lo	Sweetened Beverages
92611600	Horchata beverage, NFS	Lo	Sweetened Beverages
92612010	Sugar cane beverage	Lo	Sweetened Beverages
92613010	Cornmeal beverage	Lo	Sweetened Beverages
92613510	Cornmeal beverage with chocolate	Lo	Sweetened Beverages
92650000	Red Bull Energy Drink	Lo	Sweetened Beverages
92650005	Red Bull Energy Drink, sugar-free	Lo	Diet Beverages
92650100	Full Throttle Energy Drink	Lo	Sweetened Beverages
92650200	Monster Energy Drink	Lo	Sweetened Beverages
92650205	Mountain Dew AMP Energy Drink	Lo	Sweetened Beverages
92650210	Mountain Dew AMP Energy Drink, s	Lo	Diet Beverages
92650700	Rockstar Energy Drink	Lo	Sweetened Beverages
92650705	Rockstar Energy Drink, sugar-free	Lo	Diet Beverages
92650800	Vault Energy Drink	Lo	Sweetened Beverages
92650805	Vault Zero Energy drink	Lo	Diet Beverages
92651000	Energy drink	Lo	Sweetened Beverages

92731000	Fruit-flavored drink, non-carbonatec	Lo	Sweetened Beverages
92741000	Fruit-flavored drink, non-carbonatec	Lo	Sweetened Beverages
92801000	Wine, nonalcoholic	Lo	Sweetened Beverages
92803000	Nonalcoholic malt beverage	Lo	Sweetened Beverages
92804000	Shirley Temple	Lo	Sweetened Beverages
92900100	Fruit flavored drink, with high vitami	Lo	Other
92900110	Fruit flavored drink, powdered, not r	Lo	Other
92900200	Fruit flavored drink, powdered, not r	Lo	Other
92900300	Sports drink, dry concentrate, not re	Lo	Other
93101000	Beer	Lo	Alcoholic Beverages
93102000	Beer, light	Lo	Alcoholic Beverages
93102100	Beer, low carb	Lo	Alcoholic Beverages
93102200	Beer, light, higher alcohol	Lo	Alcoholic Beverages
93102300	Beer, higher alcohol	Lo	Alcoholic Beverages
93106000	Alcoholic malt beverage, sweetenec	Lo	Alcoholic Beverages
93106100	Alcoholic malt beverage	Lo	Alcoholic Beverages
93106500	Hard cider	Lo	Alcoholic Beverages
93201000	Cordial or liqueur	Lo	Alcoholic Beverages
93202000	Cordial or liqueur, coffee flavored	Lo	Alcoholic Beverages
93301000	Cocktail, NFS	Lo	Alcoholic Beverages
93301010	Alexander	Lo	Alcoholic Beverages
93301020	Bacardi cocktail	Lo	Alcoholic Beverages
93301030	Bloody Mary	Lo	Alcoholic Beverages
93301031	Canadian Club and soda	Lo	Alcoholic Beverages
93301032	Cape Cod	Lo	Alcoholic Beverages
93301040	Daiquiri	Lo	Alcoholic Beverages
93301045	Gelatin shot, alcoholic	Lo	Alcoholic Beverages
93301050	Gimlet	Lo	Alcoholic Beverages
93301060	Gin and Tonic	Lo	Alcoholic Beverages
93301075	Greyhound	Lo	Alcoholic Beverages
93301080	High ball	Lo	Alcoholic Beverages
93301083	Jagerbomb	Lo	Alcoholic Beverages
93301085	Kamikaze	Lo	Alcoholic Beverages
93301090	Manhattan	Lo	Alcoholic Beverages
93301100	Margarita	Lo	Alcoholic Beverages
93301110	Martini	Lo	Alcoholic Beverages
93301111	Martini, flavored	Lo	Alcoholic Beverages
93301115	Mimosa	Lo	Alcoholic Beverages
93301120	Mint julep	Lo	Alcoholic Beverages
93301125	Mojito	Lo	Alcoholic Beverages
93301130	Old fashioned	Lo	Alcoholic Beverages
93301132	Orange Blossom	Lo	Alcoholic Beverages
93301135	Rob Roy	Lo	Alcoholic Beverages
93301139	Salty Dog	Lo	Alcoholic Beverages
93301140	Screwdriver	Lo	Alcoholic Beverages
93301141	Seabreeze	Lo	Alcoholic Beverages
93301142	Seven and Seven	Lo	Alcoholic Beverages
93301150	Tom Collins	Lo	Alcoholic Beverages
93301160	Whiskey sour	Lo	Alcoholic Beverages
93301170	Whiskey and soda	Lo	Alcoholic Beverages
93301180	Mixed Drinks (for recipe modificatio	Lo	Alcoholic Beverages

93301181	Whiskey and water	Lo	Alcoholic Beverages
93301182	Whiskey and cola	Lo	Alcoholic Beverages
93301183	Whiskey and diet cola	Lo	Alcoholic Beverages
93301184	Whiskey and ginger ale	Lo	Alcoholic Beverages
93301190	Rum and cola	Lo	Alcoholic Beverages
93301191	Rum and diet cola	Lo	Alcoholic Beverages
93301200	Pina Colada	Lo	Alcoholic Beverages
93301205	Brandy and cola	Lo	Alcoholic Beverages
93301211	Vodka and soda	Lo	Alcoholic Beverages
93301213	Vodka and lemonade	Lo	Alcoholic Beverages
93301214	Vodka and cola	Lo	Alcoholic Beverages
93301215	Vodka and diet cola	Lo	Alcoholic Beverages
93301216	Vodka and energy drink	Lo	Alcoholic Beverages
93301217	Vodka and water	Lo	Alcoholic Beverages
93301218	Vodka and tonic	Lo	Alcoholic Beverages
93301220	Coquito, Puerto Rican (coconut, rur	Lo	Alcoholic Beverages
93301240	Black Russian	Lo	Alcoholic Beverages
93301250	White Russian	Lo	Alcoholic Beverages
93301270	Fruit punch, alcoholic	Lo	Alcoholic Beverages
93301275	Champagne punch	Lo	Alcoholic Beverages
93301310	Mai Tai	Lo	Alcoholic Beverages
93301320	Tequila Sunrise	Lo	Alcoholic Beverages
93301330	Gin Rickey	Lo	Alcoholic Beverages
93301360	Long Island iced tea	Lo	Alcoholic Beverages
93301370	Fuzzy Navel	Lo	Alcoholic Beverages
93301400	Irish Coffee	Lo	Alcoholic Beverages
93301450	Liqueur with cream	Lo	Alcoholic Beverages
93301500	Frozen daiquiri	Lo	Alcoholic Beverages
93301510	Frozen margarita	Lo	Alcoholic Beverages
93301550	Eggnog, alcoholic	Lo	Alcoholic Beverages
93301600	Gin fizz	Lo	Alcoholic Beverages
93401000	Wine, table, dry	Lo	Alcoholic Beverages
93401010	Wine, table, red	Lo	Alcoholic Beverages
93401020	Wine, table, white	Lo	Alcoholic Beverages
93401030	Wine, table, rose	Lo	Alcoholic Beverages
93401100	Wine, rice	Lo	Alcoholic Beverages
93401300	Wine, cooking (assume cooked)	Lo	Alcoholic Beverages
93402000	Wine, dessert, sweet	Lo	Alcoholic Beverages
93403000	Wine, light	Lo	Alcoholic Beverages
93404000	Wine cooler	Lo	Alcoholic Beverages
93404500	Sangria	Lo	Alcoholic Beverages
93404550	Sangria, red	Lo	Alcoholic Beverages
93404560	Sangria, white	Lo	Alcoholic Beverages
93404600	Sangria, Puerto Rican style	Lo	Alcoholic Beverages
93405000	Wine spritzer	Lo	Alcoholic Beverages
93501000	Brandy	Lo	Alcoholic Beverages
93502000	Whiskey	Lo	Alcoholic Beverages
93502100	Scotch	Lo	Alcoholic Beverages
93503000	Gin	Lo	Alcoholic Beverages
93504000	Rum	Lo	Alcoholic Beverages
93504100	Rum cooler	Lo	Alcoholic Beverages

93505000	Vodka	Lo	Alcoholic Beverages
93505100	Tequila	Lo	Alcoholic Beverages
94000000	Water as an ingredient	Lo	Plain Water
94000100	Water, tap	Lo	Plain Water
94100100	Water, bottled, unsweetened	Lo	Plain Water
94100200	Water, bottled, sweetened, with low	Lo	Flavored or Enhanced Water
94100300	Water, bottled, flavored (Capri Sun	Lo	Flavored or Enhanced Water
94210100	Water, bottled, flavored (Propel Wa	Lo	Flavored or Enhanced Water
94210200	Water, bottled, flavored (Glaceau V	Lo	Flavored or Enhanced Water
94210300	Water, bottled, flavored (SoBe Life	Lo	Flavored or Enhanced Water
94220100	Propel Zero Water	Lo	Flavored or Enhanced Water
94220200	Glaceau Water, low calorie	Lo	Flavored or Enhanced Water
94220215	Water, bottled, flavored, sugar free	Lo	Flavored or Enhanced Water
94220310	Water, bottled, flavored, sugar free	Lo	Flavored or Enhanced Water
94300100	Water, baby, bottled, unsweetened	Lo	Baby Beverages
95101000	Nutritional drink or shake, ready-to-	Lo	Sweetened Beverages
95101010	Nutritional drink or shake, ready-to-	Lo	Sweetened Beverages
95102000	Nutritional drink or shake, ready-to-	Lo	Sweetened Beverages
95103000	Nutritional drink or shake, ready-to-	Lo	Sweetened Beverages
95103010	Nutritional drink or shake, ready-to-	Lo	Sweetened Beverages
95104000	Nutritional drink or shake, ready-to-	Lo	Sweetened Beverages
95105000	Nutritional drink or shake, ready-to-	Lo	Sweetened Beverages
95106000	Nutritional drink or shake, ready-to-	Lo	Sweetened Beverages
95106010	Nutritional drink or shake, ready-to-	Lo	Sweetened Beverages
95110000	Nutritional drink or shake, ready-to-	Lo	Sweetened Beverages
95110010	Nutritional drink or shake, ready-to-	Lo	Sweetened Beverages
95110020	Nutritional drink or shake, high prot	Lo	Sweetened Beverages
95120000	Nutritional drink or shake, ready-to-	Lo	Sweetened Beverages
95120010	Nutritional drink or shake, high prot	Lo	Sweetened Beverages
95120020	Nutritional drink or shake, high prot	Lo	Sweetened Beverages
95120050	Nutritional drink or shake, liquid, so	Lo	Sweetened Beverages
95201000	Nutritional powder mix (Carnation In	Lo	Protein and Nutritional Powders
95201010	Nutritional powder mix, sugar free (	Lo	Protein and Nutritional Powders
95201200	Nutritional powder mix (EAS Whey	Lo	Protein and Nutritional Powders
95201300	Nutritional powder mix (EAS Soy Pr	Lo	Protein and Nutritional Powders
95201500	Nutritional powder mix, high protein	Lo	Protein and Nutritional Powders
95201600	Nutritional powder mix (Isopure)	Lo	Protein and Nutritional Powders
95201700	Nutritional powder mix (Kellogg's Sp	Lo	Protein and Nutritional Powders
95202000	Nutritional powder mix (Muscle Milk	Lo	Protein and Nutritional Powders
95202010	Nutritional powder mix, light (Muscle	Lo	Protein and Nutritional Powders
95210000	Nutritional powder mix (Slim Fast)	Lo	Protein and Nutritional Powders
95210020	Nutritional powder mix, high protein	Lo	Protein and Nutritional Powders
95220000	Nutritional powder mix, NFS	Lo	Protein and Nutritional Powders
95220010	Nutritional powder mix, high protein	Lo	Protein and Nutritional Powders
95230000	Nutritional powder mix, whey based	Lo	Protein and Nutritional Powders
95230010	Nutritional powder mix, protein, soy	Lo	Protein and Nutritional Powders
95230020	Nutritional powder mix, protein, ligh	Lo	Protein and Nutritional Powders
95230030	Nutritional powder mix, protein, NFS	Lo	Protein and Nutritional Powders
95310200	Energy drink (Full Throttle)	Lo	Sweetened Beverages
95310400	Energy drink (Monster)	Lo	Sweetened Beverages
95310500	Energy drink (Mountain Dew AMP)	Lo	Sweetened Beverages

95310560	Energy drink (NOS)	Lo	Sweetened Beverages
95310600	Energy drink (Red Bull)	Lo	Sweetened Beverages
95310700	Energy drink (Rockstar)	Lo	Sweetened Beverages
95310750	Energy drink (SoBe Energize Energ	Lo	Sweetened Beverages
95310800	Energy drink (Vault)	Lo	Sweetened Beverages
95311000	Energy Drink	Lo	Sweetened Beverages
95312400	Energy drink, low calorie (Monster)	Lo	Diet Beverages
95312410	Energy drink, sugar free (Monster)	Lo	Diet Beverages
95312500	Energy drink, sugar free (Mountain	Lo	Diet Beverages
95312550	Energy drink, sugar free (No Fear)	Lo	Diet Beverages
95312560	Energy drink (Ocean Spray Cran-Er	Lo	Sweetened Beverages
95312600	Energy drink, sugar-free (Red Bull)	Lo	Diet Beverages
95312700	Energy drink, sugar free (Rockstar)	Lo	Diet Beverages
95312800	Energy drink, sugar free (Vault)	Lo	Diet Beverages
95312900	Energy drink (XS)	Lo	Sweetened Beverages
95313200	Energy drink, sugar free	Lo	Diet Beverages
95320200	Sports drink (Gatorade G)	Lo	Sweetened Beverages
95320500	Sports drink (Powerade)	Lo	Sweetened Beverages
95321000	Sports drink, NFS	Lo	Sweetened Beverages
95322200	Sports drink, low calorie (Gatorade	Lo	Diet Beverages
95322500	Sports drink, low calorie (Powerade	Lo	Diet Beverages
95323000	Sports drink, low calorie	Lo	Diet Beverages
95330100	Fluid replacement, electrolyte soluti	Lo	Sweetened Beverages
95341000	FUZE Slenderize fortified low calori	Lo	Diet Beverages
95342000	Fruit juice, acai blend	Lo	100% Juice
42500000	Trail mix, NFS	Lo	Plant-based Protein Foods
42500100	Trail mix with nuts	Lo	Plant-based Protein Foods
42501000	Trail mix with nuts and fruit	Lo	Plant-based Protein Foods
42501500	Trail mix with chocolate	Lo	Plant-based Protein Foods
43103200	Sesame paste (sesame butter mad	Lo	Plant-based Protein Foods
43103300	Tahini	Lo	Plant-based Protein Foods
11459990	Frozen yogurt, NFS	Med	Other Desserts
11460000	Frozen yogurt, vanilla	Med	Other Desserts
11460100	Frozen yogurt, chocolate	Med	Other Desserts
11460150	Yogurt, frozen, NS as to flavor, lowf	Med	Other Desserts
11460160	Yogurt, frozen, chocolate, lowfat mi	Med	Other Desserts
11460170	Yogurt, frozen, flavors other than ch	Med	Other Desserts
11460190	Yogurt, frozen, NS as to flavor, non	Med	Other Desserts
11460200	Yogurt, frozen, chocolate, nonfat m	Med	Other Desserts
11460250	Yogurt, frozen, flavors other than ch	Med	Other Desserts
11460300	Yogurt, frozen, flavors other than ch	Med	Other Desserts
11460400	Yogurt, frozen, chocolate, nonfat m	Med	Other Desserts
11460410	Yogurt, frozen, flavors other than ch	Med	Other Desserts
11460430	Yogurt, frozen, chocolate, whole mi	Med	Other Desserts
11460440	Yogurt, frozen, flavors other than ch	Med	Other Desserts
11460500	Frozen yogurt, soft serve, vanilla	Med	Other Desserts
11460510	Frozen yogurt, soft serve, chocolate	Med	Other Desserts
11461000	Yogurt, frozen, chocolate-coated	Med	Other Desserts
11461200	Frozen yogurt sandwich	Med	Other Desserts
11461210	Frozen yogurt bar, vanilla	Med	Other Desserts
11461250	Frozen yogurt cone, chocolate	Med	Other Desserts

11461260	Frozen yogurt cone, vanilla	Med	Other Desserts
11461270	Yogurt, frozen, cone, flavors other t	Med	Other Desserts
11461280	Yogurt, frozen, cone, chocolate, low	Med	Other Desserts
11461300	Frozen yogurt cone, vanilla, waffle c	Med	Other Desserts
11553000	Fruit smoothie drink, made with frui	Med	Sweetened Beverages
11553100	Fruit smoothie, NFS	Med	Sweetened Beverages
11553110	Fruit smoothie, with whole fruit and	Med	Sweetened Beverages
11553120	Fruit smoothie, with whole fruit and	Med	Sweetened Beverages
12350000	Dip, sour cream base	Med	Condiments and Sauces
12350010	Dip, NFS	Med	Condiments and Sauces
12350020	Dip, sour cream base, reduced calo	Med	Condiments and Sauces
12350100	Spinach dip	Med	Condiments and Sauces
12350110	Spinach and artichoke dip	Med	Condiments and Sauces
12350200	Chipotle dip, regular	Med	Condiments and Sauces
12350210	Dill dip, regular	Med	Condiments and Sauces
12350220	Onion dip, regular	Med	Condiments and Sauces
12350225	Onion dip, light	Med	Condiments and Sauces
12350230	Ranch dip, regular	Med	Condiments and Sauces
12350235	Ranch dip, light	Med	Condiments and Sauces
12350240	Spinach dip, regular	Med	Condiments and Sauces
12350245	Spinach dip, light	Med	Condiments and Sauces
12350250	Vegetable dip, regular	Med	Condiments and Sauces
12350255	Vegetable dip, light	Med	Condiments and Sauces
14010100	Cheese, Cheddar or American type	Med	Cheese
14104010	Cheese, natural, Cheddar or Americ	Med	Cheese
14104015	Cheese, natural, Cheddar or Americ	Med	Cheese
14104020	Cheese, Cheddar or American type	Med	Cheese
14107010	Cheese, Mozzarella, NFS	Med	Cheese
14107020	Cheese, Mozzarella, whole milk	Med	Cheese
14107030	Cheese, Mozzarella, part skim	Med	Cheese
14107040	Cheese, Mozzarella, reduced sodiu	Med	Cheese
14107060	Cheese, Mozzarella, nonfat or fat fr	Med	Cheese
14108010	Cheese, Parmesan, dry grated	Med	Cheese
14108015	Cheese, Parmesan, dry grated, red	Med	Cheese
14108020	Cheese, Parmesan, hard	Med	Cheese
14108050	Cheese, Parmesan, low sodium	Med	Cheese
14108060	Cheese, Parmesan, dry grated, fat f	Med	Cheese
14410620	Cheese, with wine	Med	Cheese
14620100	Dip, cream cheese base	Med	Condiments and Sauces
14620115	Spinach and artichoke dip	Med	Condiments and Sauces
14620120	Shrimp dip, cream cheese base	Med	Condiments and Sauces
14640000	Cheese sandwich, NFS	Med	Mixed Dishes - Sandwiches (single code)
14640008	Cheese sandwich, Cheddar cheese	Med	Mixed Dishes - Sandwiches (single code)
14640010	Cheese sandwich, Cheddar cheese	Med	Mixed Dishes - Sandwiches (single code)
14640012	Cheese sandwich, Cheddar cheese	Med	Mixed Dishes - Sandwiches (single code)
14640020	Cheese sandwich, reduced fat Che	Med	Mixed Dishes - Sandwiches (single code)
14640024	Cheese sandwich, reduced fat Che	Med	Mixed Dishes - Sandwiches (single code)
14640032	Cheese sandwich, Cheddar cheese	Med	Mixed Dishes - Sandwiches (single code)
14640034	Cheese sandwich, Cheddar cheese	Med	Mixed Dishes - Sandwiches (single code)
14640036	Cheese sandwich, Cheddar cheese	Med	Mixed Dishes - Sandwiches (single code)
14640046	Cheese sandwich, reduced fat Che	Med	Mixed Dishes - Sandwiches (single code)

14640048	Cheese sandwich, reduced fat Che	Med	Mixed Dishes - Sandwiches (single code)
14640056	Cheese sandwich, Cheddar cheese	Med	Mixed Dishes - Sandwiches (single code)
14640058	Cheese sandwich, Cheddar cheese	Med	Mixed Dishes - Sandwiches (single code)
14640060	Cheese sandwich, Cheddar cheese	Med	Mixed Dishes - Sandwiches (single code)
14640068	Cheese sandwich, reduced fat Che	Med	Mixed Dishes - Sandwiches (single code)
14670000	Mozzarella cheese, tomato, and ba	Med	Vegetables, excluding Potatoes
21500000	Ground beef, raw	Med	Meats
26153100	Tuna, fresh, raw	Med	Seafood
26153170	Tuna, fresh, dried	Med	Seafood
26315100	Oysters, raw	Med	Seafood
27116400	Steak tartare	Med	Mixed Dishes - Meat, Poultry, Seafood
27151030	Ceviche	Med	Mixed Dishes - Meat, Poultry, Seafood
27351020	Codfish salad, Puerto Rican style (	Med	Mixed Dishes - Meat, Poultry, Seafood
27446300	Chicken or turkey garden salad, chi	Med	Mixed Dishes - Meat, Poultry, Seafood
27446310	Chicken or turkey garden salad, chi	Med	Mixed Dishes - Meat, Poultry, Seafood
27446315	Chicken or turkey garden salad with	Med	Mixed Dishes - Meat, Poultry, Seafood
27446320	Chicken or turkey, breaded, fried, g	Med	Mixed Dishes - Meat, Poultry, Seafood
27446330	Chicken or turkey garden salad with	Med	Mixed Dishes - Meat, Poultry, Seafood
27446350	Asian chicken or turkey garden sala	Med	Mixed Dishes - Meat, Poultry, Seafood
27446355	Asian chicken or turkey garden sala	Med	Mixed Dishes - Meat, Poultry, Seafood
27446360	Chicken or turkey caesar garden sa	Med	Mixed Dishes - Meat, Poultry, Seafood
27446362	Chicken or turkey, breaded, fried, c	Med	Mixed Dishes - Meat, Poultry, Seafood
27450030	Salmon salad	Med	Mixed Dishes - Meat, Poultry, Seafood
27450090	Tuna salad with cheese	Med	Mixed Dishes - Meat, Poultry, Seafood
27450100	Tuna salad with egg	Med	Mixed Dishes - Meat, Poultry, Seafood
27450110	Shrimp garden salad, shrimp, lettuc	Med	Mixed Dishes - Meat, Poultry, Seafood
27450120	Shrimp garden salad, shrimp, lettuc	Med	Mixed Dishes - Meat, Poultry, Seafood
27450180	Seafood garden salad with seafood	Med	Mixed Dishes - Meat, Poultry, Seafood
27450190	Seafood garden salad with seafood	Med	Mixed Dishes - Meat, Poultry, Seafood
27450310	Lomi salmon	Med	Mixed Dishes - Meat, Poultry, Seafood
27451070	Codfish salad, Puerto Rican style, S	Med	Mixed Dishes - Meat, Poultry, Seafood
27460490	Julienne salad, meat, cheese, eggs	Med	Mixed Dishes - Meat, Poultry, Seafood
41420100	Miso sauce	Med	Condiments and Sauces
41420110	Miso	Med	Condiments and Sauces
57602100	Oats, raw	Med	Other
57602500	Oat bran, uncooked	Med	Other
58148180	Macaroni or pasta salad with chees	Med	Mixed Dishes - Grain-based
58148500	Pasta or macaroni salad with oil an	Med	Mixed Dishes - Grain-based
58150100	Bibimbap, Korean	Med	Mixed Dishes - Grain-based
58175110	Tabbouleh	Med	Mixed Dishes - Grain-based
61110010	Kumquat, raw	Med	Fruits
61119100	Orange peel	Med	Other
63100100	Fruit, NFS	Med	Fruits
63100110	Fruit, pickled	Med	Fruits
63101000	Apple, raw	Med	Fruits
63103010	Apricot, raw	Med	Fruits
63115010	Cherries, raw	Med	Fruits
63115200	Cherries, frozen	Med	Fruits
63119010	Fig, raw	Med	Fruits
63123000	Grapes, raw	Med	Fruits
63123010	Grapes, European type, adherent s	Med	Fruits

63123020	Grapes, American type, slip skin, ra	Med	Fruits
63125010	Guava, raw	Med	Fruits
63129020	Mango, pickled	Med	Fruits
63131010	Nectarine, raw	Med	Fruits
63135010	Peach, raw	Med	Fruits
63135610	Peach, frozen, NS as to added swe	Med	Fruits
63135620	Peach, frozen	Med	Fruits
63135630	Peach, frozen, with sugar	Med	Fruits
63137010	Pear, raw	Med	Fruits
63137050	Pear, Asian, raw	Med	Fruits
63139010	Persimmon, raw	Med	Fruits
63143010	Plum, raw	Med	Fruits
63143650	Plum, pickled	Med	Fruits
63200100	Berries, NFS	Med	Fruits
63200200	Berries, frozen, NFS	Med	Fruits
63201010	Blackberries, raw	Med	Fruits
63201600	Blackberries, frozen	Med	Fruits
63203010	Blueberries, raw	Med	Fruits
63203550	Blueberries, frozen, sweetened	Med	Fruits
63203570	Blueberries, frozen, NS as to sweet	Med	Fruits
63203600	Blueberries, frozen	Med	Fruits
63205010	Boysenberries, raw	Med	Fruits
63205600	Boysenberries, frozen	Med	Fruits
63207010	Cranberries, raw	Med	Fruits
63214000	Huckleberries, raw	Med	Fruits
63215010	Loganberries, raw	Med	Fruits
63217010	Mulberries, raw	Med	Fruits
63219000	Raspberries, raw	Med	Fruits
63219020	Raspberries, red, raw	Med	Fruits
63219600	Raspberries, frozen, NS as to adde	Med	Fruits
63219610	Raspberries, frozen	Med	Fruits
63219620	Raspberries, frozen, with sugar	Med	Fruits
63223020	Strawberries, raw	Med	Fruits
63223030	Strawberries, raw, with sugar	Med	Fruits
63223600	Strawberries, frozen, NS as to adde	Med	Fruits
63223610	Strawberries, frozen	Med	Fruits
63223620	Strawberries, frozen, with sugar	Med	Fruits
63311000	Fruit salad, fresh or raw, excluding	Med	Fruits
63311050	Fruit salad, fresh or raw, including c	Med	Fruits
63311080	Fruit cocktail or mix, frozen	Med	Fruits
63311180	Fruit mixture, frozen	Med	Fruits
63320100	Fruit salad, Puerto Rican style	Med	Fruits
63401010	Apple salad with dressing	Med	Fruits
63401015	Apple and grape salad with yogurt a	Med	Fruits
63401020	Apple and cabbage salad with dress	Med	Fruits
63401030	Apple and fruit salad with dressing	Med	Fruits
63402950	Fruit salad, excluding citrus fruits, w	Med	Fruits
63402960	Fruit salad, excluding citrus fruits, w	Med	Fruits
63402970	Fruit salad, excluding citrus fruits, w	Med	Fruits
63402980	Fruit salad, excluding citrus fruits, w	Med	Fruits
63403010	Fruit salad, including citrus fruits, w	Med	Fruits

63403020	Fruit salad, including citrus fruit, wit	Med	Fruits
63403030	Fruit salad, including citrus fruits, w	Med	Fruits
63403040	Fruit salad, including citrus fruits, w	Med	Fruits
63412010	Pear salad with dressing	Med	Fruits
63413010	Pineapple salad with dressing	Med	Fruits
63415100	Soup, fruit	Med	Mixed Dishes - Soups
64134015	Fruit smoothie, with whole fruit, no d	Med	Sweetened Beverages
64134020	Fruit smoothie, with whole fruit, no d	Med	Sweetened Beverages
64134025	Fruit smoothie, with whole fruit, non	Med	Sweetened Beverages
72101100	Beet greens, raw	Med	Vegetables, excluding Potatoes
72104100	Chard, raw	Med	Vegetables, excluding Potatoes
72107100	Collards, raw	Med	Vegetables, excluding Potatoes
72110100	Cress, raw	Med	Vegetables, excluding Potatoes
72113100	Dandelion greens, raw	Med	Vegetables, excluding Potatoes
72116000	Romaine lettuce, raw	Med	Vegetables, excluding Potatoes
72116140	Caesar salad (with romaine)	Med	Vegetables, excluding Potatoes
72116150	Caesar salad, with romaine, no dres	Med	Vegetables, excluding Potatoes
72119190	Kale, raw	Med	Vegetables, excluding Potatoes
72122100	Mustard greens, raw	Med	Vegetables, excluding Potatoes
72124100	Radicchio, raw	Med	Vegetables, excluding Potatoes
72125100	Spinach, raw	Med	Vegetables, excluding Potatoes
72130100	Watercress, raw	Med	Vegetables, excluding Potatoes
72201100	Broccoli, raw	Med	Vegetables, excluding Potatoes
73101010	Carrots, raw	Med	Vegetables, excluding Potatoes
73101110	Carrots, raw, salad	Med	Vegetables, excluding Potatoes
73101210	Carrots, raw, salad with apples	Med	Vegetables, excluding Potatoes
73105000	Beet juice	Med	100% Juice
73105010	Carrot juice, 100%	Med	100% Juice
74101000	Tomatoes, raw	Med	Vegetables, excluding Potatoes
74102000	Tomatoes, green, raw	Med	Vegetables, excluding Potatoes
74205020	Tomato, green, pickled	Med	Condiments and Sauces
74402100	Salsa, NFS	Med	Condiments and Sauces
74402110	Salsa, pico de gallo	Med	Condiments and Sauces
74402200	Salsa, red, homemade	Med	Condiments and Sauces
74402300	Salsa made with fruit	Med	Condiments and Sauces
74402310	Green tomato-chile sauce, raw (Sal	Med	Condiments and Sauces
74402350	Salsa verde or salsa, green	Med	Condiments and Sauces
74403000	Hot Thai sauce	Med	Condiments and Sauces
74405010	Tomato relish	Med	Condiments and Sauces
74506000	Tomato and cucumber salad made	Med	Vegetables, excluding Potatoes
75100250	Raw vegetable, NFS	Med	Vegetables, excluding Potatoes
75100750	Artichoke, raw	Med	Vegetables, excluding Potatoes
75100800	Asparagus, raw	Med	Vegetables, excluding Potatoes
75101800	Green beans, raw	Med	Vegetables, excluding Potatoes
75102000	Beans, lima, raw	Med	Vegetables, excluding Potatoes
75102500	Beets, raw	Med	Vegetables, excluding Potatoes
75102600	Broccoflower, raw	Med	Vegetables, excluding Potatoes
75102750	Brussels sprouts, raw	Med	Vegetables, excluding Potatoes
75103000	Cabbage, green, raw	Med	Vegetables, excluding Potatoes
75104000	Cabbage, Chinese, raw	Med	Vegetables, excluding Potatoes
75105000	Cabbage, red, raw	Med	Vegetables, excluding Potatoes

75105500	Cactus, raw	Med	Vegetables, excluding Potatoes
75107000	Cauliflower, raw	Med	Vegetables, excluding Potatoes
75109000	Celery, raw	Med	Vegetables, excluding Potatoes
75109010	Fennel bulb, raw	Med	Vegetables, excluding Potatoes
75109400	Basil, raw	Med	Other
75109500	Chives, raw	Med	Other
75109550	Cilantro, raw	Med	Other
75109600	Corn, raw	Med	Vegetables, excluding Potatoes
75111000	Cucumber, raw	Med	Vegetables, excluding Potatoes
75111200	Eggplant, raw	Med	Vegetables, excluding Potatoes
75111800	Jicama, raw	Med	Vegetables, excluding Potatoes
75112000	Kohlrabi, raw	Med	Vegetables, excluding Potatoes
75112500	Leek, raw	Med	Vegetables, excluding Potatoes
75113000	Lettuce, raw	Med	Vegetables, excluding Potatoes
75113060	Lettuce, Boston, raw	Med	Vegetables, excluding Potatoes
75113080	Lettuce, arugula, raw	Med	Vegetables, excluding Potatoes
75114000	Mixed salad greens, raw	Med	Vegetables, excluding Potatoes
75115000	Mushrooms, raw	Med	Vegetables, excluding Potatoes
75117010	Onions, green, raw	Med	Vegetables, excluding Potatoes
75117020	Onions, raw	Med	Vegetables, excluding Potatoes
75119000	Parsley, raw	Med	Other
75120000	Green peas, raw	Med	Vegetables, excluding Potatoes
75121000	Pepper, hot chili, raw	Med	Condiments and Sauces
75121400	Pepper, poblano, raw	Med	Vegetables, excluding Potatoes
75121500	Pepper, Serrano, raw	Med	Vegetables, excluding Potatoes
75122000	Pepper, raw, NFS	Med	Vegetables, excluding Potatoes
75122100	Pepper, sweet, green, raw	Med	Vegetables, excluding Potatoes
75122200	Pepper, sweet, red, raw	Med	Vegetables, excluding Potatoes
75124000	Pepper, banana, raw	Med	Vegetables, excluding Potatoes
75125000	Radish, raw	Med	Vegetables, excluding Potatoes
75127000	Rutabaga, raw	Med	Vegetables, excluding Potatoes
75127500	Seaweed, raw	Med	Vegetables, excluding Potatoes
75127750	Snowpeas, raw	Med	Vegetables, excluding Potatoes
75128000	Summer squash, yellow, raw	Med	Vegetables, excluding Potatoes
75128010	Summer squash, green, raw	Med	Vegetables, excluding Potatoes
75129000	Turnip, raw	Med	Vegetables, excluding Potatoes
75132100	Celery juice	Med	100% Juice
75140500	Broccoli salad with cauliflower, chee	Med	Vegetables, excluding Potatoes
75140510	Broccoli slaw salad	Med	Vegetables, excluding Potatoes
75140990	Cabbage salad or coleslaw, from fa	Med	Vegetables, excluding Potatoes
75141000	Cabbage salad or coleslaw, made v	Med	Vegetables, excluding Potatoes
75141005	Cabbage salad or coleslaw, made v	Med	Vegetables, excluding Potatoes
75141020	Cabbage salad or coleslaw, made v	Med	Vegetables, excluding Potatoes
75141025	Cabbage salad or coleslaw, made v	Med	Vegetables, excluding Potatoes
75141030	Cabbage salad or coleslaw, made v	Med	Vegetables, excluding Potatoes
75141035	Cabbage salad or coleslaw, made v	Med	Vegetables, excluding Potatoes
75141040	Cabbage salad or coleslaw, made v	Med	Vegetables, excluding Potatoes
75141100	Cabbage salad or coleslaw with app	Med	Vegetables, excluding Potatoes
75141200	Cabbage salad or coleslaw with pin	Med	Vegetables, excluding Potatoes
75142000	Cucumber and vegetable namasu	Med	Vegetables, excluding Potatoes
75142500	Cucumber salad, made with sour cr	Med	Vegetables, excluding Potatoes

75142550	Cucumber salad, made with Italian	Med	Vegetables, excluding Potatoes
75142600	Cucumber salad made with cucumb	Med	Vegetables, excluding Potatoes
75143000	Lettuce, salad with assorted vegeta	Med	Vegetables, excluding Potatoes
75143050	Lettuce, salad with assorted vegeta	Med	Vegetables, excluding Potatoes
75143100	Lettuce, salad with avocado, tomato	Med	Vegetables, excluding Potatoes
75143200	Lettuce, salad with cheese, tomato	Med	Vegetables, excluding Potatoes
75143300	Lettuce, salad with egg, tomato, and	Med	Vegetables, excluding Potatoes
75143350	Lettuce, salad with egg, cheese, tor	Med	Vegetables, excluding Potatoes
75145000	Seven-layer salad, lettuce salad ma	Med	Mixed Dishes - Bean/Vegetable-based
75146000	Greek Salad, no dressing	Med	Mixed Dishes - Bean/Vegetable-based
75147000	Spinach salad, no dressing	Med	Mixed Dishes - Bean/Vegetable-based
75148010	Cobb salad, no dressing	Med	Mixed Dishes - Bean/Vegetable-based
75230000	Sauerkraut	Med	Condiments and Sauces
75416600	Pea salad with cheese	Med	Vegetables, excluding Potatoes
75500110	Green beans, pickled	Med	Condiments and Sauces
75500210	Beets, pickled	Med	Condiments and Sauces
75500510	Celery, pickled	Med	Condiments and Sauces
75502010	Cauliflower, pickled	Med	Condiments and Sauces
75502500	Cabbage, fresh, pickled, Japanese	Med	Condiments and Sauces
75502510	Cabbage, red, pickled	Med	Condiments and Sauces
75503010	Pickles, dill	Med	Condiments and Sauces
75503030	Cucumber pickles, sour	Med	Condiments and Sauces
75503040	Pickles, sweet	Med	Condiments and Sauces
75503080	Eggplant, pickled	Med	Condiments and Sauces
75503085	Ginger root, pickled	Med	Condiments and Sauces
75503100	Mustard pickles	Med	Condiments and Sauces
75503140	Cucumber pickles, sweet, reduced	Med	Condiments and Sauces
75505000	Mushrooms, pickled	Med	Condiments and Sauces
75507000	Okra, pickled	Med	Condiments and Sauces
75510000	Olives, NFS	Med	Condiments and Sauces
75510010	Olives, green	Med	Condiments and Sauces
75511010	Hot pepper sauce	Med	Condiments and Sauces
75511020	Peppers, pickled	Med	Condiments and Sauces
75511040	Pepper, hot, pickled	Med	Condiments and Sauces
75511100	Pickles, NFS	Med	Condiments and Sauces
75511200	Pickles, mixed	Med	Condiments and Sauces
75512010	Radishes, pickled, Hawaiian style	Med	Condiments and Sauces
75513010	Seaweed, pickled	Med	Condiments and Sauces
75515100	Vegetables, pickled	Med	Condiments and Sauces
75534030	Turnip, pickled	Med	Condiments and Sauces
75534500	Tsukemono, Japanese pickles	Med	Condiments and Sauces
75535000	Zucchini, pickled	Med	Condiments and Sauces
75604600	Gazpacho	Med	Mixed Dishes - Soups
78101100	Fruit and vegetable smoothie, with	Med	Sweetened Beverages
78101110	Fruit and vegetable smoothie, adde	Med	Sweetened Beverages
78101115	Fruit and vegetable smoothie, non-	Med	Sweetened Beverages
78101118	Fruit and vegetable smoothie, non-	Med	Sweetened Beverages
78101125	Fruit and vegetable smoothie, no da	Med	Sweetened Beverages
78101130	Vegetable smoothie	Med	Sweetened Beverages
89902020	Lettuce, for use on a sandwich	Med	Vegetables, excluding Potatoes
89902030	Mushrooms, for use on a sandwich	Med	Vegetables, excluding Potatoes

89902050	Pepper, for use on a sandwich	Med	Vegetables, excluding Potatoes
89902060	Spinach, for use on a sandwich	Med	Vegetables, excluding Potatoes
89902070	Tomatoes, for use on a sandwich	Med	Vegetables, excluding Potatoes
91408100	Chinese preserved sweet vegetable	Med	Condiments and Sauces
11112120	Milk, acidophilus, low fat (1%)	Hi	Milk
11112130	Milk, acidophilus, reduced fat (2%)	Hi	Milk
11115000	Buttermilk, fat free (skim)	Hi	Milk
11115100	Buttermilk, low fat (1%)	Hi	Milk
11115200	Buttermilk, reduced fat (2%)	Hi	Milk
11115300	Buttermilk, whole	Hi	Milk
11115400	Kefir, NS as to fat content	Hi	Milk
11400000	Yogurt, NFS	Hi	Yogurt
11400010	Yogurt, Greek, NS as to type of mil	Hi	Yogurt
11410000	Yogurt, NS as to type of milk or flav	Hi	Yogurt
11411010	Yogurt, NS as to type of milk, plain	Hi	Yogurt
11411100	Yogurt, whole milk, plain	Hi	Yogurt
11411200	Yogurt, low fat milk, plain	Hi	Yogurt
11411300	Yogurt, nonfat milk, plain	Hi	Yogurt
11411390	Yogurt, Greek, NS as to type of mil	Hi	Yogurt
11411400	Yogurt, Greek, whole milk, plain	Hi	Yogurt
11411410	Yogurt, Greek, low fat milk, plain	Hi	Yogurt
11411420	Yogurt, Greek, nonfat milk, plain	Hi	Yogurt
11420000	Yogurt, vanilla, NS as to type of mil	Hi	Yogurt
11421000	Yogurt, vanilla, whole milk	Hi	Yogurt
11422000	Yogurt, vanilla, low fat milk	Hi	Yogurt
11422100	Yogurt, vanilla, low fat milk, light	Hi	Yogurt
11423000	Yogurt, vanilla, nonfat milk	Hi	Yogurt
11424000	Yogurt, vanilla, nonfat milk, light	Hi	Yogurt
11424500	Yogurt, Greek, vanilla, whole milk	Hi	Yogurt
11424510	Yogurt, Greek, vanilla, low fat	Hi	Yogurt
11424520	Yogurt, Greek, vanilla, nonfat	Hi	Yogurt
11425000	Yogurt, chocolate, NS as to type of	Hi	Yogurt
11426000	Yogurt, chocolate, whole milk	Hi	Yogurt
11427000	Yogurt, chocolate, nonfat milk	Hi	Yogurt
11428000	Yogurt, Greek, chocolate, nonfat	Hi	Yogurt
11430000	Yogurt, NS as to type of milk, fruit	Hi	Yogurt
11431000	Yogurt, whole milk, fruit	Hi	Yogurt
11432000	Yogurt, low fat milk, fruit	Hi	Yogurt
11432500	Yogurt, fruit, low fat milk, light	Hi	Yogurt
11433000	Yogurt, nonfat milk, fruit	Hi	Yogurt
11433500	Yogurt, fruit, nonfat milk, light	Hi	Yogurt
11433990	Yogurt, Greek, NS as to type of mil	Hi	Yogurt
11434000	Yogurt, Greek, whole milk, fruit	Hi	Yogurt
11434010	Yogurt, Greek, low fat milk, fruit	Hi	Yogurt
11434020	Yogurt, Greek, nonfat milk, fruit	Hi	Yogurt
11434090	Yogurt, NS as to type of milk, flavor	Hi	Yogurt
11434100	Yogurt, whole milk, flavors other th	Hi	Yogurt
11434200	Yogurt, low fat milk, flavors other th	Hi	Yogurt
11434300	Yogurt, nonfat milk, flavors other th	Hi	Yogurt
11435000	Yogurt, Greek, NS as to type of mil	Hi	Yogurt
11435010	Yogurt, Greek, whole milk, flavors o	Hi	Yogurt

11435020	Yogurt, Greek, low fat milk, flavors o	Hi	Yogurt
11435030	Yogurt, Greek, nonfat milk, flavors o	Hi	Yogurt
11435100	Yogurt, Greek, with oats	Hi	Yogurt
11436000	Yogurt, liquid	Hi	Yogurt
11440010	Chipotle dip, yogurt based	Hi	Condiments and Sauces
11440020	Dill dip, yogurt based	Hi	Condiments and Sauces
11440040	Ranch dip, yogurt based	Hi	Condiments and Sauces
11440050	Spinach dip, yogurt based	Hi	Condiments and Sauces
11440060	Tzatziki dip	Hi	Condiments and Sauces
11440070	Vegetable dip, yogurt based	Hi	Condiments and Sauces
11445000	Yogurt, fruit and nuts, lowfat milk	Hi	Yogurt
11446000	Yogurt parfait, low fat, with fruit	Hi	Yogurt
11480010	Yogurt, whole milk, baby food	Hi	Baby Foods
11480020	Yogurt, whole milk, baby food, with	Hi	Baby Foods
11480040	Yogurt, whole milk, baby food, with	Hi	Baby Foods
12310100	Sour cream, regular	Hi	Fats and Oils
12310300	Sour cream, reduced fat	Hi	Fats and Oils
12310350	Sour cream, light	Hi	Fats and Oils
12310370	Sour cream, fat free	Hi	Fats and Oils
12320100	Sour cream, imitation	Hi	Fats and Oils
14010000	Cheese, NFS	Hi	Cheese
14100100	Cheese, natural, NFS	Hi	Cheese
14101010	Cheese, Blue or Roquefort	Hi	Cheese
14102010	Cheese, Brick	Hi	Cheese
14103010	Cheese, Camembert	Hi	Cheese
14103020	Cheese, Brie	Hi	Cheese
14104100	Cheese, Cheddar	Hi	Cheese
14104110	Cheese, Cheddar, reduced fat	Hi	Cheese
14104115	Cheese, Cheddar, nonfat or fat free	Hi	Cheese
14104200	Cheese, Colby	Hi	Cheese
14104250	Cheese, Colby Jack	Hi	Cheese
14104400	Cheese, Feta	Hi	Cheese
14104600	Cheese, Fontina	Hi	Cheese
14104700	Cheese, goat	Hi	Cheese
14105010	Cheese, Gouda or Edam	Hi	Cheese
14105200	Cheese, Gruyere	Hi	Cheese
14106200	Cheese, Monterey	Hi	Cheese
14106500	Cheese, Monterey, reduced fat	Hi	Cheese
14107200	Cheese, Muenster	Hi	Cheese
14107250	Cheese, Muenster, reduced fat	Hi	Cheese
14108200	Cheese, Port du Salut	Hi	Cheese
14108400	Cheese, Provolone	Hi	Cheese
14108420	Cheese, provolone, reduced fat	Hi	Cheese
14109010	Cheese, Swiss	Hi	Cheese
14109020	Cheese, Swiss, reduced sodium	Hi	Cheese
14109030	Cheese, Swiss, reduced fat	Hi	Cheese
14109040	Cheese, Swiss, nonfat or fat free	Hi	Cheese
14110010	Cheese, Cheddar, reduced sodium	Hi	Cheese
14110030	Cheese, Cheddar or Colby, lowfat	Hi	Cheese
41420200	Natto	Hi	Condiments and Sauces
41420380	Yogurt, soy	Lo	Plant-based Protein Foods

75100300	Sprouts, NFS	Hi	Vegetables, excluding Potatoes
75100500	Alfalfa sprouts, raw	Hi	Vegetables, excluding Potatoes
75101000	Bean sprouts, raw	Hi	Vegetables, excluding Potatoes
75502520	Kimchi	Hi	Condiments and Sauces
75503050	Cucumber pickles, fresh	Hi	Condiments and Sauces
75510030	Olives, stuffed	Hi	Condiments and Sauces
