# Supplementary material for: Association between high dietary intake of live microbes from food and all-cause and cause-specific mortality in cancer patients: A prospective cohort study
Source: Medicine (Baltimore). 2026 Jul 10;105(28):e49649. doi: 10.1097/MD.0000000000049649 (PMC13363040; doi:10.1097/MD.0000000000049649)
Supplement: Supplementary file 2 [file medi-105-e49649-s002.docx]

**Supplementary Table 2 Standardized Mean Differences of Baseline Characteristics Across MedHi Intake Groups**

| **Variables** | **G1 vs G2** | **G1 vs G3** | **G2 vs G3** |
| --- | --- | --- | --- |
| **Age** | 0.035 | 0.072 | 0.037 |
| **Sex** | 0.154 | 0.111 | 0.043 |
| **Race** | 0.190 | 0.245 | 0.054 |
| **Marital status** | 0.149 | 0.180 | 0.031 |
| **BMI** | 0.031 | 0.015 | 0.016 |
| **PIR group** | 0.270 | 0.421 | 0.179 |
| **Education level** | 0.210 | 0.391 | 0.183 |
| **HEI-2015** | 0.337 | 0.854 | 0.500 |
| **Physical activity** | 0.174 | 0.325 | 0.150 |
| **Smoking status** | 0.193 | 0.426 | 0.236 |
| **Alcohol intake** | 0.186 | 0.234 | 0.065 |
| **CVD** | 0.114 | 0.189 | 0.075 |
| **Hypertension** | 0.076 | 0.125 | 0.049 |
| **Hyperlipidemia** | 0.008 | 0.042 | 0.050 |
| **Diabetes** | 0.072 | 0.077 | 0.005 |

Abbreviations: BMI, body mass index; HEI-2015, healthy eating index-2015; PIR, poverty income ratio; CVD, cardiovascular disease; MedHi, medium-to-high microbial content food.
