# Supplementary material for: Association between high dietary intake of live microbes from food and all-cause and cause-specific mortality in cancer patients: A prospective cohort study
Source: Medicine (Baltimore). 2026 Jul 10;105(28):e49649. doi: 10.1097/MD.0000000000049649 (PMC13363040; doi:10.1097/MD.0000000000049649)
Supplement: Supplementary file 3 [file medi-105-e49649-s003.docx]

**Supplementary Table 3 Adjusted hazard ratios of three levels of MedHi food intake with risk of all-cause and cause-specific mortality after additional adjustment for total energy intake**

| **Characteristics** | **Levels of MedHi food intake** | | | ***P*-trend** |
| --- | --- | --- | --- | --- |
|  | **G1(MedHi=0)** | **G2(0<MedHi<132.7)** | **G3(MedHi≥132.7)** |  |
| **All-cause mortality** |  |  |  |  |
| Model 1 | 1 (reference) | 0.75 (0.65-0.85) | 0.59 (0.52-0.68) | <0.001 |
| Model 2 | 1 (reference) | 0.89 (0.78-1.02) | 0.79 (0.68-0.91) | 0.001 |
| Model 3 | 1 (reference) | 0.91 (0.79-1.04) | 0.80 (0.69-0.92) | 0.002 |
|  |  |  |  |  |
| **Cancer-specific mortality** |  |  |  |  |
| Model 1 | 1 (reference) | 0.76 (0.60-0.95) | 0.63 (0.50-0.80) | <0.001 |
| Model 2 | 1 (reference) | 0.95 (0.75-1.21) | 0.95 (0.73-1.22) | 0.668 |
| Model 3 | 1 (reference) | 0.95 (0.75-1.20) | 0.93 (0.72-1.20) | 0.569 |
|  |  |  |  |  |
| **Non-cancer mortality** |  |  |  |  |
| Model 1 | 1 (reference) | 0.74 (0.63-0.87) | 0.57 (0.48-0.68) | <0.001 |
| Model 2 | 1 (reference) | 0.86 (0.73-1.02) | 0.72 (0.60-0.86) | <0.001 |
| Model 3 | 1 (reference) | 0.88 (0.74-1.04) | 0.73 (0.61-0.88) | 0.001 |

Model 1 was adjusted for age and sex. Model 2 was additionally adjusted for race, marital status, BMI, PIR group, educational level, HEI-2015, physical activity, smoking status, alcohol intake, and total energy intake. Model 3 was additionally adjusted for CVD, hypertension, hyperlipidemia, and diabetes.

Abbreviations: BMI, body mass index; HEI-2015, healthy eating index-2015; PIR, poverty income ratio; CVD, cardiovascular disease; MedHi, medium-to-high microbial content food.
