# Supplementary material for: Association between high dietary intake of live microbes from food and all-cause and cause-specific mortality in cancer patients: A prospective cohort study
Source: Medicine (Baltimore). 2026 Jul 10;105(28):e49649. doi: 10.1097/MD.0000000000049649 (PMC13363040; doi:10.1097/MD.0000000000049649)
Supplement: Supplementary file 4 [file medi-105-e49649-s004.docx]

**Supplementary Table 4 Survey-weighted Cox proportional hazards models for the association between MedHi food intake and all-cause and cause-specific mortality among cancer survivors**

| **Characteristics** | **Levels of MedHi food intake** | | | ***P*-trend** |
| --- | --- | --- | --- | --- |
|  | **G1(MedHi=0)** | **G2(0<MedHi<132.7)** | **G3(MedHi≥132.7)** |  |
| **All-cause mortality** |  |  |  |  |
| Model 1 | 1 (reference) | 0.77 (0.66-0.89) | 0.60 (0.51-0.70) | <0.001 |
| Model 2 | 1 (reference) | 0.94 (0.80-1.11) | 0.83 (0.69-0.98) | 0.028 |
| Model 3 | 1 (reference) | 0.96 (0.81-1.14) | 0.84 (0.70-1.00) | 0.045 |
|  |  |  |  |  |
| **Cancer-specific mortality** |  |  |  |  |
| Model 1 | 1 (reference) | 0.91 (0.67-1.23) | 1.05 (0.79-1.40) | 0.715 |
| Model 2 | 1 (reference) | 0.90 (0.67-1.22) | 1.06 (0.80-1.40) | 0.679 |
| Model 3 | 1 (reference) | 0.91 (0.67-1.23) | 1.05 (0.79-1.40) | 0.715 |
|  |  |  |  |  |
| **Non-cancer mortality** |  |  |  |  |
| Model 1 | 1 (reference) | 0.79 (0.66-0.95) | 0.55 (0.45-0.68) | <0.001 |
| Model 2 | 1 (reference) | 0.95 (0.78-1.16) | 0.72 (0.58-0.89) | 0.002 |
| Model 3 | 1 (reference) | 0.98 (0.80-1.20) | 0.74 (0.59-0.92) | 0.005 |

Model 1 was adjusted for age and sex. Model 2 was additionally adjusted for race, marital status, BMI, PIR group, educational level, HEI-2015, physical activity, smoking status, and alcohol intake. Model 3 was additionally adjusted for CVD, hypertension, hyperlipidemia, and diabetes.

Abbreviations: BMI, body mass index; HEI-2015, healthy eating index-2015; PIR, poverty income ratio; CVD, cardiovascular disease; MedHi, medium-to-high microbial content food.
